# Supplementary material for: Multicomponent reactions provide key molecules for secret communication
Source: Nat Commun. 2018 Apr 12;9:1439. doi: 10.1038/s41467-018-03784-x (PMC5897361; doi:10.1038/s41467-018-03784-x)
Supplement: Supplementary file 1 — Supplementary Information [file 41467_2018_3784_MOESM1_ESM.pdf]

SUPPLEMENTARY INFORMATION

Multicomponent reactions provide key molecules for secret communication

Meier et al.

Table of Contents

Supplementary Figures 4

Supplementary Methods 13

Exemplarily encoded messages and tandem-MS spectra of the respective molecular keys 13

Encoded messages and file container 13

Tandem-MS spectra for decrypting example 1 14

Tandem-MS spectra for decrypting example 2 17

Tandem-MS spectra for decrypting example 3 20

Experimental Part 23

General 23

Synthetic procedures 26

Ugi reaction of perfluorononanoic acid, benzaldehyde, *tert*-butylisocyanide and butylamine 26

Ugi reaction of perfluoropentanoic acid benzaldehyde, *tert*-butylisocyanide and butylamine 31

Ugi reaction of perfluorononanoic acid, *p*-anisaldehyde, cyclohexylisocyanide and propargylamine ... 35

Ugi reaction of perfluorononanoic acid, *p*-anisaldehyde, cyclohexylisocyanide and allylamine 40

Ugi reaction of perfluorononanoic acid, benzaldehyde, cyclohexylisocyanide and propargylamine 44

Ugi reaction of perfluorononanoic acid, benzaldehyde, cyclohexylisocyanide and octylamine 49

Ugi reaction of pefluornonanoic acid, *p*-anisaldehyde, *4*-methoxyphenyl-isocyanide and propargylamine 54

Ugi reaction of perfluorononanoic acid, *p*-anisaldehyde, 2,6-dimethylphenyl-isocyanide and propargylamine 59

Ugi reaction of perfluorononanoic acid, *p*-anisaldehyde, cyclohexylisocyanide and heptylamine 64

Ugi reaction of perfluorononanoic acid, *p*-anisaldehyde, ethyl-2-isocyanoacetate and propargylamine 69

Ugi reaction of perfluorononanoic acid, valeraldehyde, *tert*-butylisocyanide and pentylamine 74

Ugi reaction of perfluorononanoic acid, valeraldehyde, *tert*-butylisocyanide and cyclohexylamine..... 79

Ugi reaction of perfluorononanoic acid, valeraldehyde, *tert*-butylisocyanide and *tert*-butylamine 83

Ugi reaction of perfluorononanoic acid, isobutyraldehyde, cyclohexylisocyanide and pentylamine..... 87

Ugi reaction of perfluorononanoic acid, valeraldehyde, cyclohexylisocyanide and pentylamine 92

Ugi reaction of perfluorononanoic acid, cyclohexancarboxaldehyde, cyclohexylisocyanide and pentylamine 96

Ugi reaction of perfluorononanoic acid, isobutyraldehyde, *tert*-butylisocyanide and cyclohexylamine 101

Ugi reaction of perfluorononanoic acid, isobutyraldehyde, pentylisocyanide and cyclohexylamine... 106

Ugi reaction of perfluorononanoic acid, isobutyraldehyde, cyclohexylisocyanide and cyclohexylamine 110

Ugi reaction of perfluoropentanoic acid valeraldehyde, *4*-methoxyphenylisocyanide and butylamine 114

Ugi reaction of perfluorotetradecanoic acid, benzaldehyde, *tert*-butylisocyanide and butylamine 119

Ugi reaction of perfluorononanoic acid, dodecanal, pentylisocyanide and benzylamine 128
Ugi reaction of perfluorononanoic acid, undec-10-enal, cyclohexylamine and benzylisocyanide 133
Ugi reaction of perfluorononanoic acid, 4-hydroxybenzaldehyde, cyclohexylisocyanide and propargylamine 138
Ugi reaction of perfluorononanoic acid, heptanal, 4-methoxyphenylisocyanide and butylamine....... 143
Ugi reaction of perfluorononanoic acid, benzaldehyde, cyclohexylisocyanide and pentylamine 148
Ugi reaction of perfluorononanoic acid, cyclamen aldehyde, *tert*-butylisocyanide and 2-pentylamine 154
Influence of stereochemistry.. 159

Supplementary Note 1 161

Solutions for encrypted messages... 161
Solution for example 1 161
Solution for example 2 165
Solution for example 3 168
Solution for deciphering the filecontainer 170

Supplementary References 170

Supplementary Figures

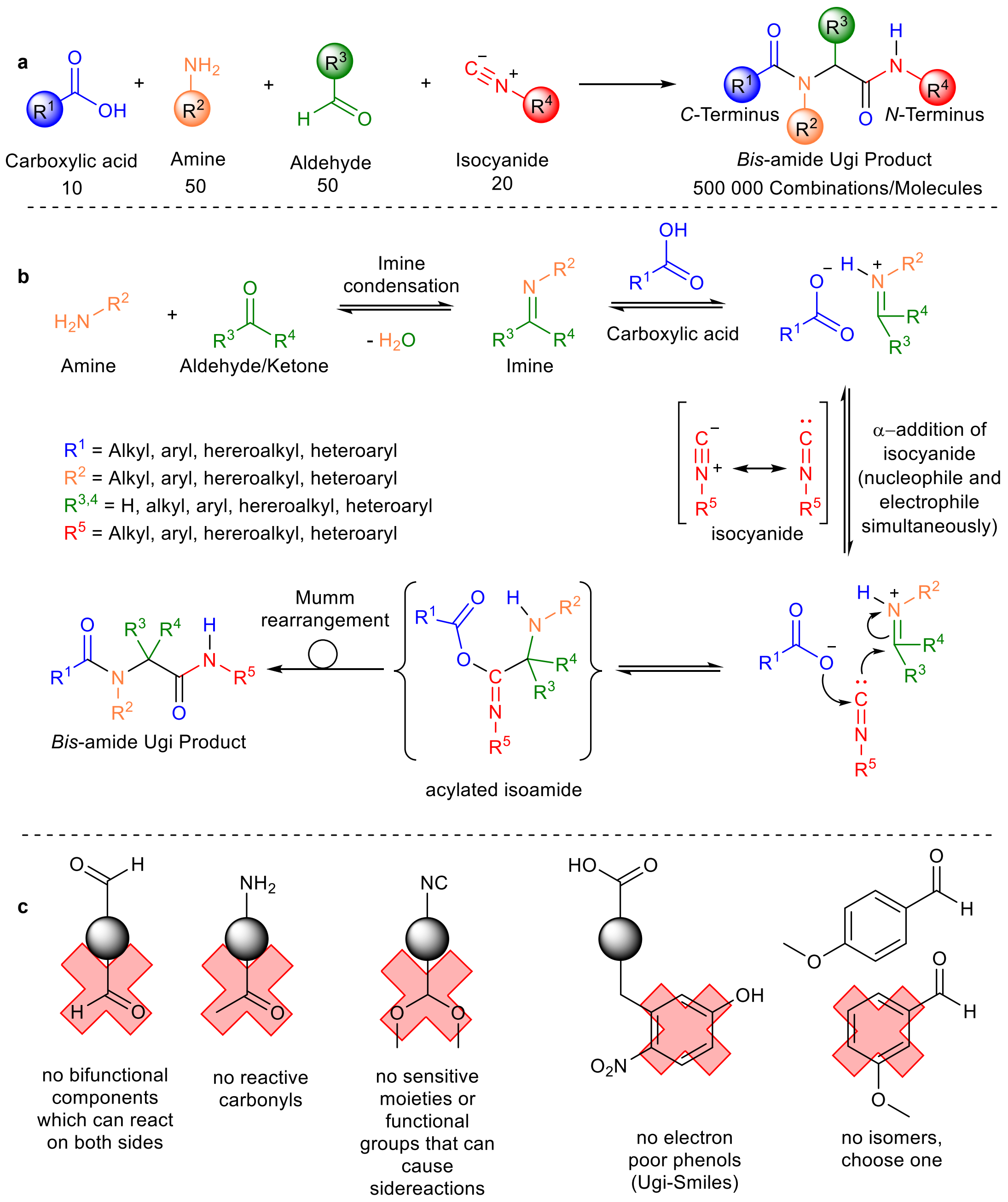

Supplementary Figure 1 | The Ugi reaction. a, Reaction equation of the Ugi reaction. **b**, Generally accepted reaction mechanism of the Ugi reaction. **c**, Illustration of limitations for setting up the list of components.

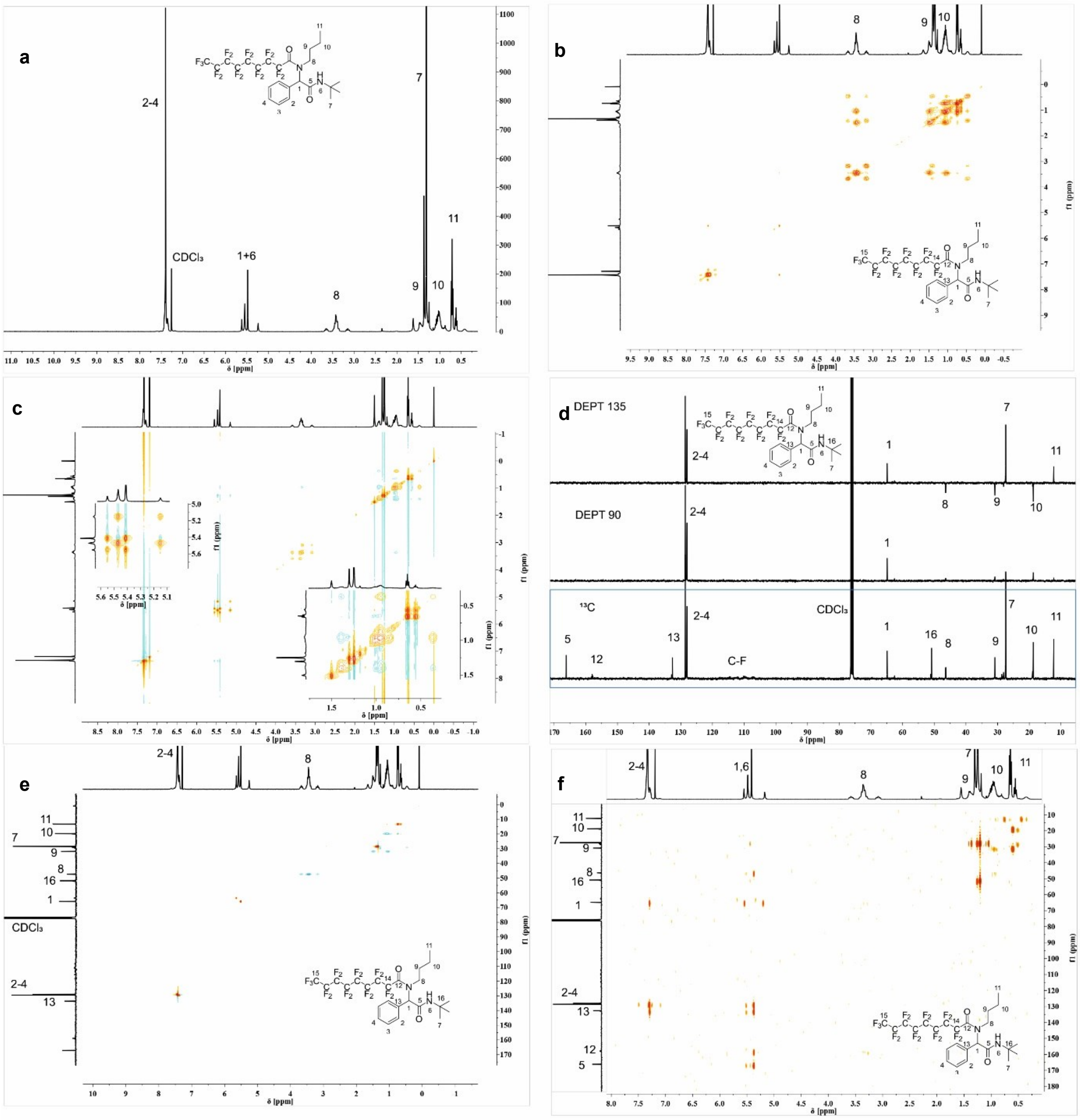

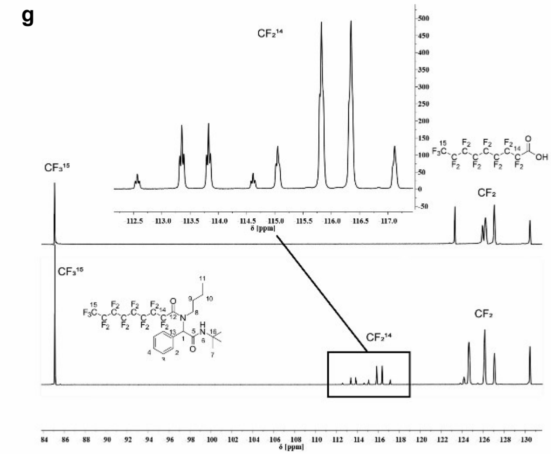

Supplementary Figure 2 | NMR evaluation of an exemplary molecular key. a, ^{1}H-NMR: Signal assignment was performed with additional information from 2D NMR experiments. Noteworthy, the NH^{6}, CH^{1} and the CH_3^{11} protons appear as split signals due to restricted rotation. The CH_2^{8} signal is split into a signal of higher order. **b**, COSY: The cross signals of the CH_2^{8} protons confirm the scalar coupling with the CH_2^{9} and CH_2^{10} protons. **c**, NOESY: The signals with the same phase (color) as the diagonal indicate chemical exchange (restricted rotation). **d**, ^{13}C-NMR stack: Top: DEPT135 experiment CH and CH_3 positive, CH_2 negative. Middle: DEPT90 experiment CH positive. Bottom: ^{13}C NMR: Signal C^{12} is weak in intensity due to ^{3}J(C-F) coupling with the vicinal CF_2^{14}. The ^{13}CF signals of the perfluorinated chain are weak in intensity due to the numerous C-F couplings. **e**, HSQC: Confirms the molecular structure. **f**, HMBC: Confirms the signal assignment for the carbon and proton signals. **g**, ^{19}F-NMR: Stack of the molecular key with the precursor perfluorinated acid**.** Top: Precursor perfluorononaic acid. Bottom: Molecular key with an expansion for the signals of the CF_2^{14} group next to the newly formed amide bond. Two AB signals (two species caused by restricted rotation) can be overserved.

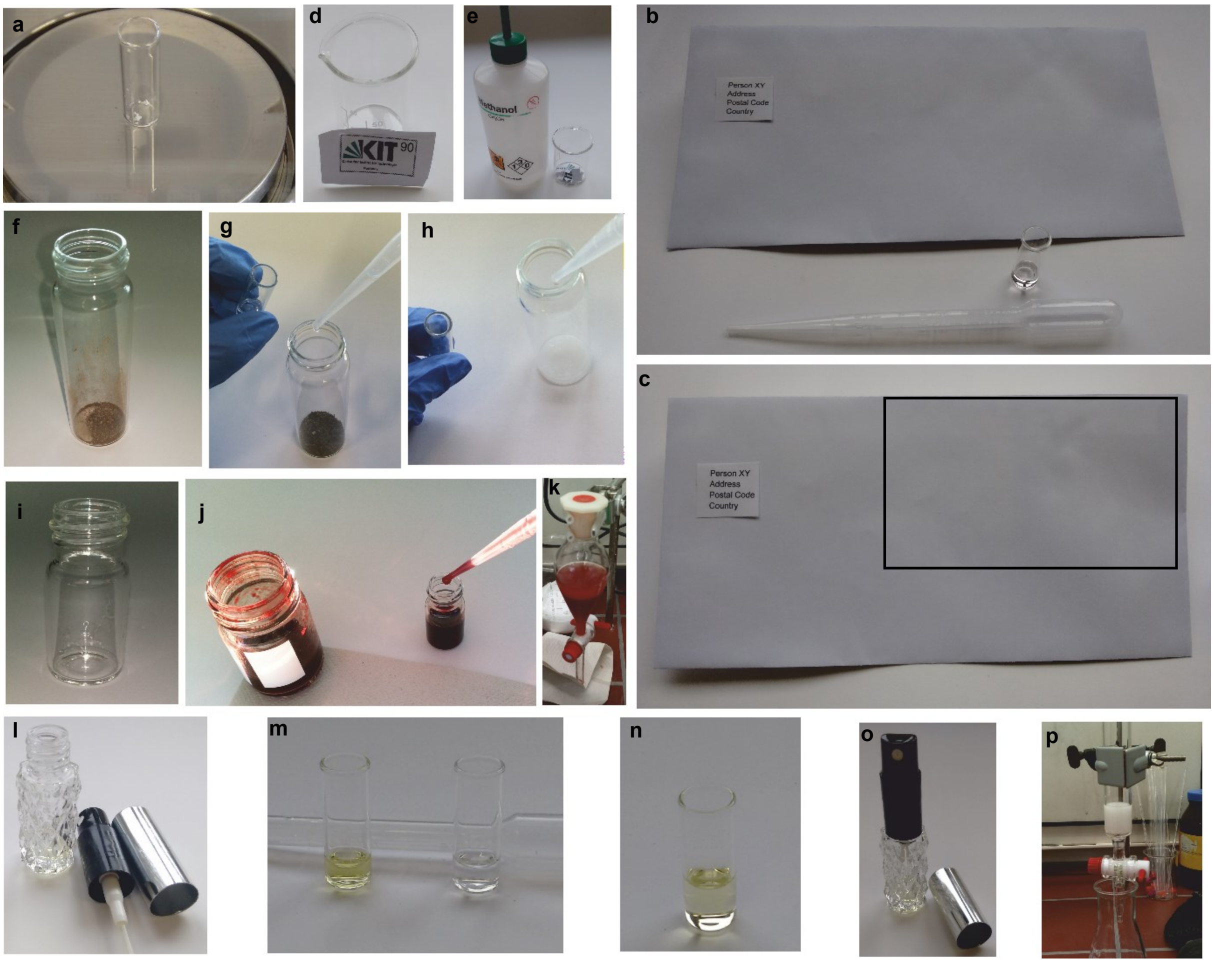

Supplementary Figure 3 | Hiding of molecular keys. a, Powdered molecular key on a scale. **b,** Envelope before application. **c,** Envelope after application of the molecular key. The black box indicates the area of interest, however the adsorbed molecular key was not visible with bare eyes. **d,** Cut piece of the envelope containing the molecular key. **e,** Shredded paper before the extraction with methanol. **f,** Molecular key adsorbed onto instant coffee powder after grinding. **g,** Molecular key on green tea. **h,** adsorption onto sugar. **i,** in this vial the molecular key was dissolved in ethanol and evaporated until a transparent film was obtained before mixing with blood. **j,** mixing of the molecular key with pig blood. **k,** Extraction of the molecular key from blood. **l,** Perfume bottle. **m,** left vial (yellow) contains the perfume, right vial (colorless) contains the molecular key dissolved in methanol. **n,** Resulting solution after mixing the perfume with the dissolved molecular key. **o,** Perfume bottle with the resulting mixture ready for transportation. Although the perfume is more diluted after mixing with the molecular key, it cannot easily be distinguished with bare eye or by the smell from the original perfume. **p,** F-SPE column for purification of the molecular keys.

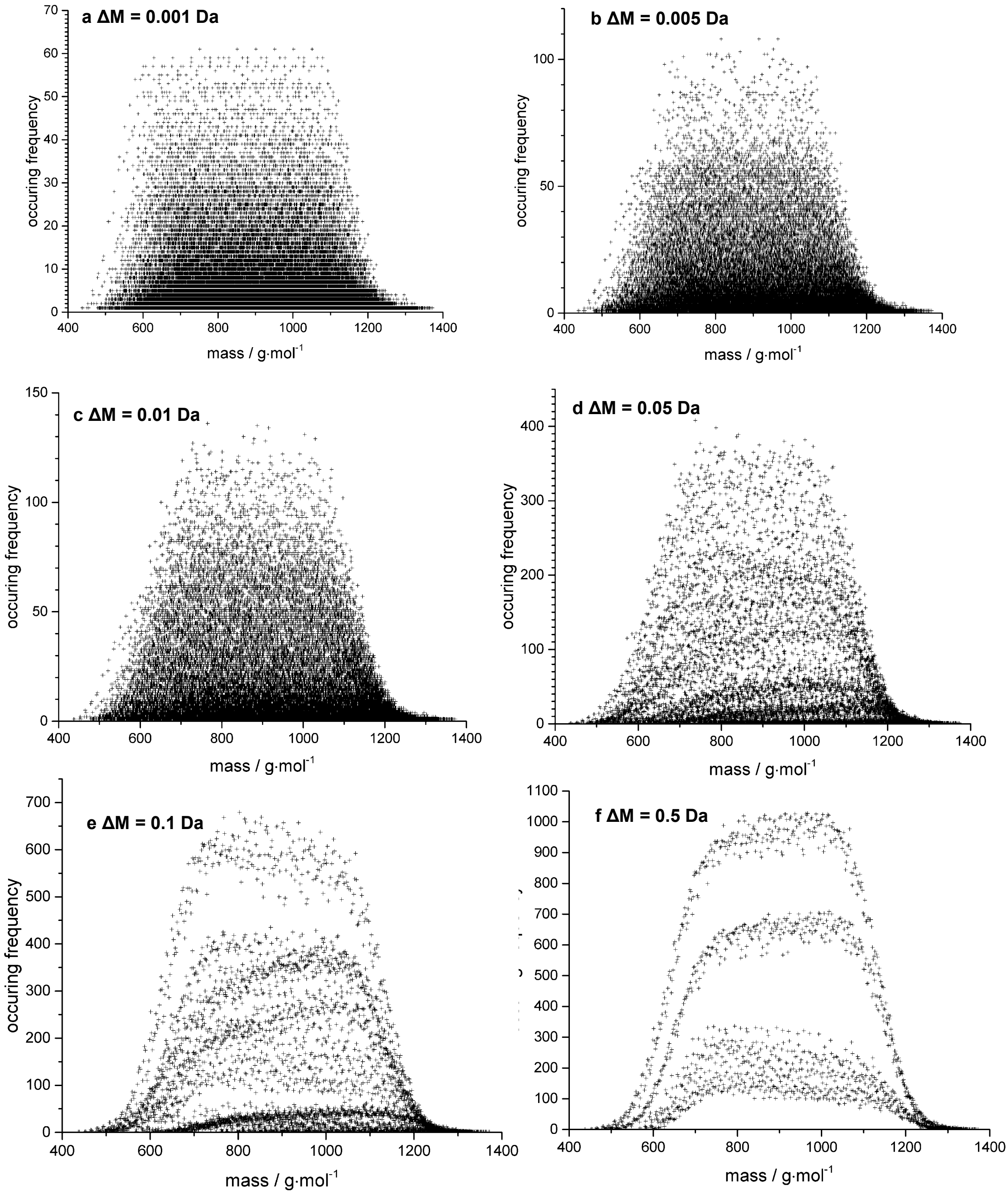

Supplementary Figure 4 | Database evaluation of the list of components. Regarding the occurring masses within certain thresholds. **a**, ΔM = 0.001 Da. **b**, ΔM = 0.005 Da. **c**, ΔM = 0.01 Da. **d**, ΔM = 0.05 Da. **e**, ΔM = 0.1 Da. **f**, ΔM = 0.5 Da. The List of components can be found in Supplementary Data 1.

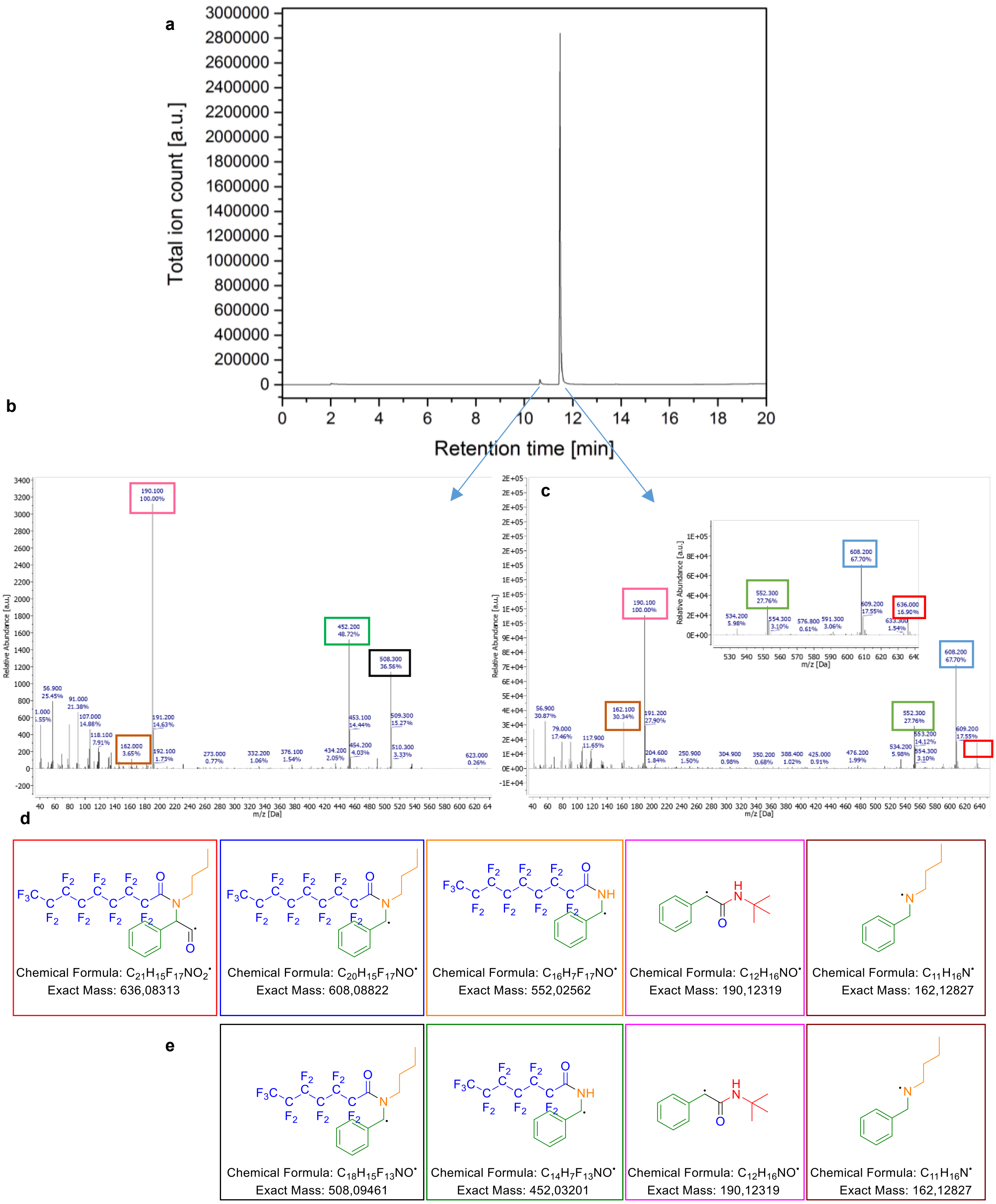

Supplementary Figure 5 | Purity determination *via* GC-MS. a, GC-MS chromatogram of a representative molecular key. The respective masses of the intensive signal at 11.5 min retention time are analyzed in **c.** The masses of the weak signal at 10.6 min retention time are analyzed in **b. b**, MS spectrum of the weak signal (1%) at 10.6 min retention time. The masses of the analyzed fragments in **e. c**, MS spectrum of the intense signal (99%) at 11.5 min retention time. The masses are assigned in **d. d**, Fragment assignment of the intense signal at 11.5 min. Interestingly, a similar fragmentation pattern as for the tandem-MS spectra can be observed. **e**, Fragment assignment of the weak signal at 10.6 min indicates the presence of a Ugi product with a shorter perfluorinated side chain. This impurity (originating from a shorter perfluorinated acid) was already present in the starting material and did not interfere with other analytical methods and the readout.

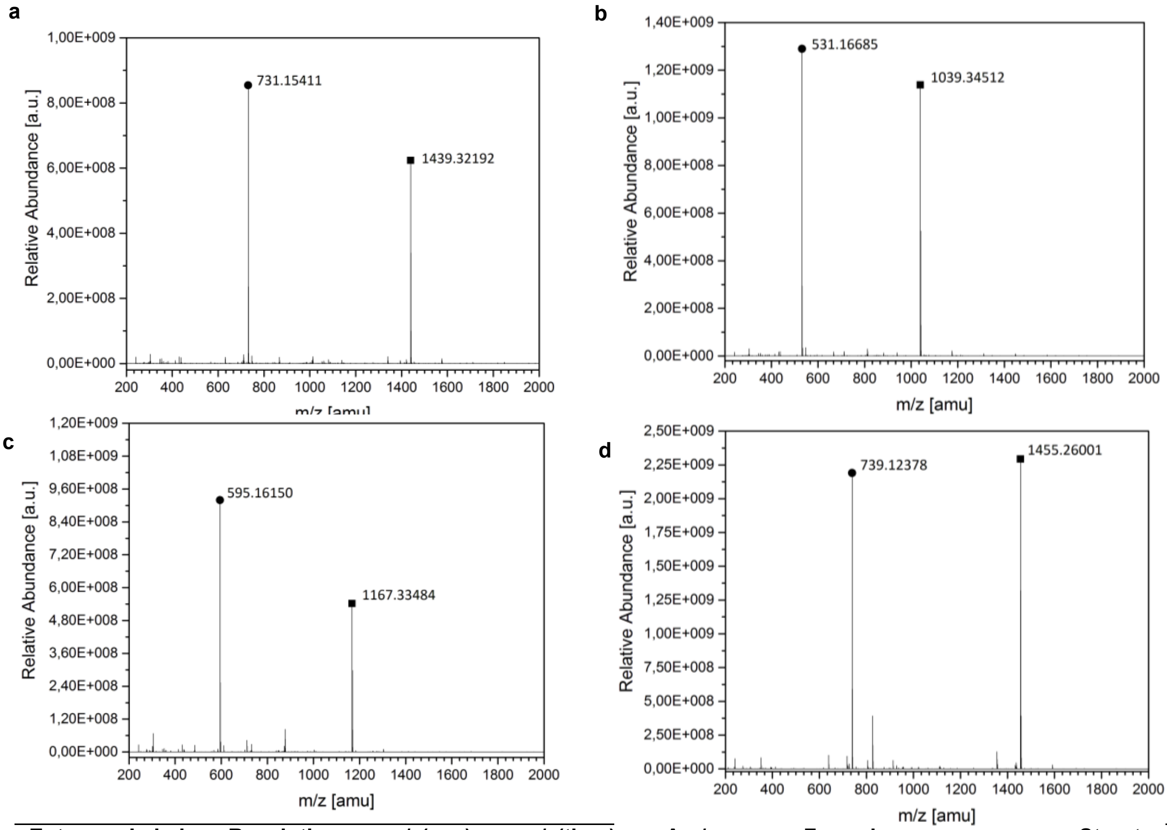

Entry	Label	Resolution	*m/z*(exp)	*m/z*(theo)	Δ*m/z*	Formula	Structure
1 in **a**	●	77000	731.1541	731.1537	0.0004	$C_{25}H_{25}O_2N_2F_{17}Na$	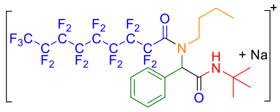
2 in **a**	■	56000	1439.3219	1439.3181	0.0038	$C_{50}H_{50}O_4N_4F_{34}Na$	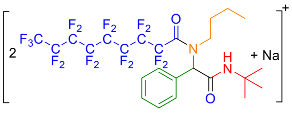
3 in **b**	●	90000	531.1669	531.1665	0.0004	$C_{21}H_{25}O_2N_2F_9Na$	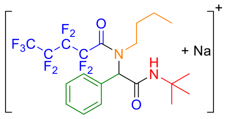
4 in **b**	■	66000	1039.3451	1039.3437	0.0014	$C_{42}H_{50}O_4N_4F_{18}Na$	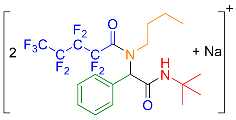
5 in **c**	●	93000	595.1615	595.1619	0.0004	$C_{25}H_{25}O_3N_2F_9Na$	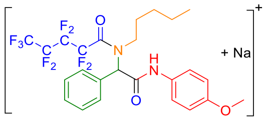
6 in **c**	■	66000	1167.3384	1167.3341	0.0043	$C_{50}H_{50}O_6N_4F_{18}Na$	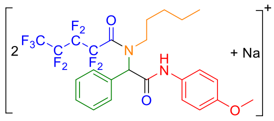
7 in **d**	●	83000	739.1238	739.1229	0.0009	$C_{42}H_{50}O_4N_4F_{18}Na$	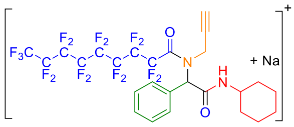
8 in **d**	■	59000	1455.2600	1455.2561	0.0039	$C_{52}H_{42}O_4N_4F_{34}Na$	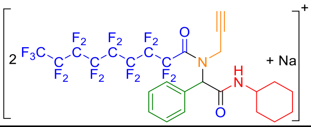

Supplementary Figure 6 | ESI-MS of different molecular keys. Top: ESI-MS spectra of four representative molecular keys. Recoded in positive mode in the range from 200 – 2000 *m/z*. The two predominant signals correspond to the [M + Na]$^+$ (●) and the [2 M + Na]$^+$ (■) adducts. **Table (bottom)**: Peak assignment of the ESI-MS spectra presented in above. The resolution (obtained by the Xcalibur software), the experimental *m/z* vs. the theoretical *m/z* values and Δ*m/z* for the proposed structure.

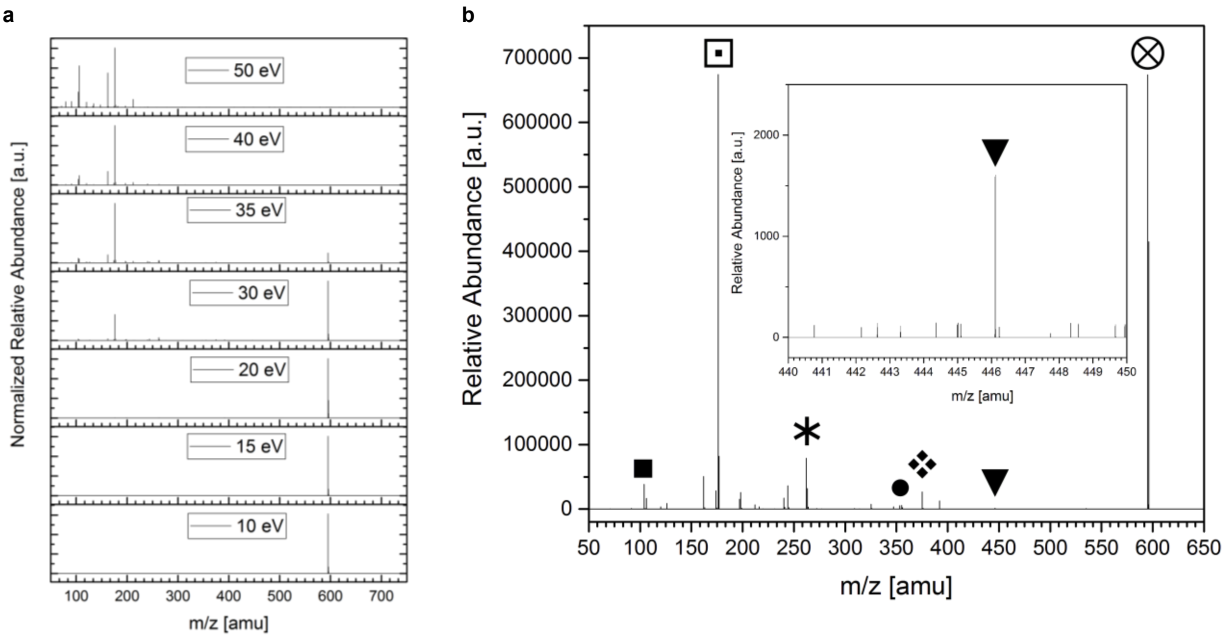

Entry	Label	Resolution	*m/z*(exp)	*m/z*(theo)	Δ*m/z*	Formula	Structure
1	⊗	89000	595.1612	595.1619	0.0007	$C_{25}H_{25}O_3N_2F_9Na$	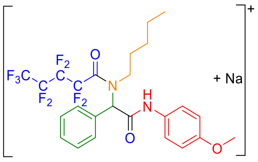
2	▼	97000	446.1137	446.1142	0.0009	$C_{17}H_{18}ONF_9Na$	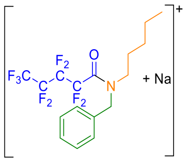
3	❖	112000	375.0274	375.0282	0.0008	$C_{12}H_7ONF_9Na$	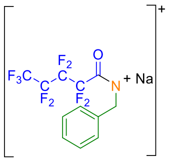
4	●	125000	325.1911	325.1916	0.0005	$C_{20}H_{25}O_2N_2$	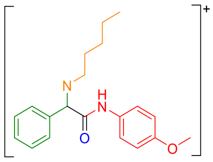
5	✱	135000	262.0839	262.0840	0.0001	$C_{15}H_{13}O_2NNa$	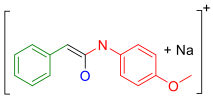
6	⊡	162000	176.1435	176.1439	0.0004	$C_{12}H_{18}N$	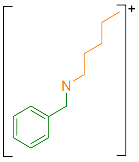
7	■	202000	104.0499	104.0500	0.0001	C_7H_6N	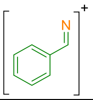

Supplementary Figure 7 | Fragmentation energy screening. a, Stacked tandem-MS spectra of a single charged species at 595 *m/z* (⊗). Recoded in positive mode with different higher-energy collision dissociation (HCD) energy levels in the relevant range from 50 – 750 *m/z*. **b**, Tandem-MS of a single charged species at 595 *m/z* (⊗). Recoded in positive mode with a higher-energy collision dissociation (HCD) of 30 eV in the relevant range from 50 – 650 *m/z*. The expansion visualizes the fragment ion (▼). **Table (bottom)**: includes peak assignment of the ESI-MS/MS spectrum presented in b at 595 *m/z* with a higher-energy collision dissociation (HCD) of 30 eV. The resolution (obtained by the Xcalibur software), the experimental *m/z* vs. the theoretical *m/z* values, and Δ*m/z* for the proposed structure.

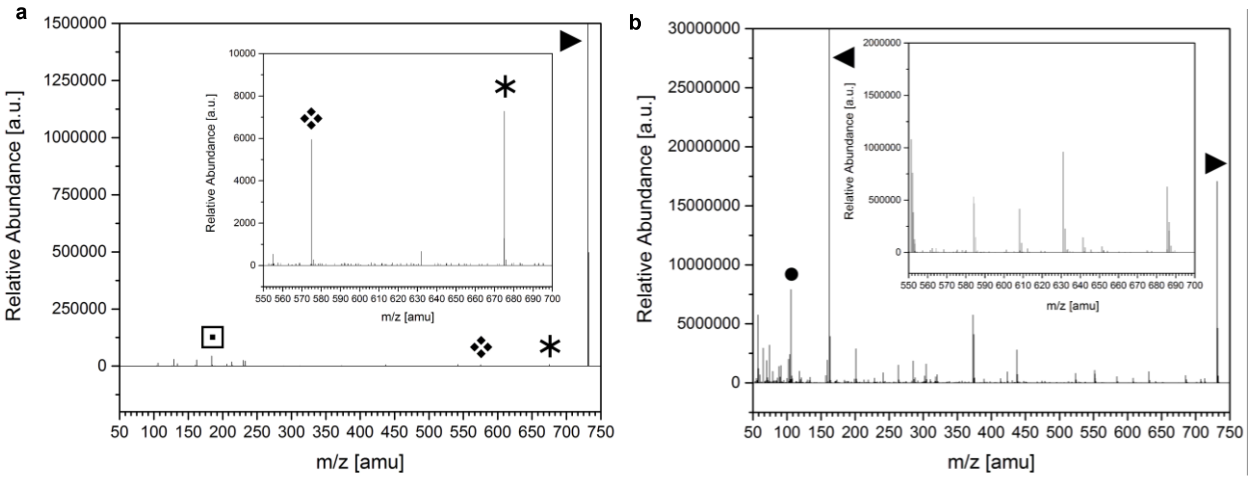

Entry	Label	Resolution	*m*/*z*(exp)	*m*/*z*(theo)	Δ*m*/*z*	Formula	Structure
1	▶	82000	731.1534	731.1542	0.0008	$C_{25}H_{25}O_2N_2F_{17}Na$	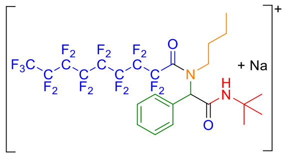
**a:** 35 eV top							
2 in **a**	✱	82000	675.0910	675.0916	0.0006	$C_{21}H_{17}O_2N_2F_{17}Na$	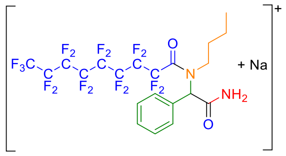
3 in **a**	❖	93000	575.0148	575.0154	0.0006	$C_{16}H_7ONF_{17}Na$	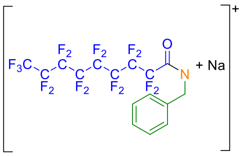
4 in **a**	⊡	157000	184.1098	184.1102	0.0004	$C_{11}H_{15}NNa$	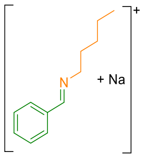
**b**: 50 eV bottom							
5 in **b**	◀	169000	162.1278	162.1283	0.0005	$C_{11}H_{16}N$	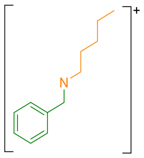
6 in **b**	●	208000	106.0660	106.0657	0.0003	C_7H_8N	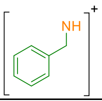

Supplementary Figure 8 | Fragmentation at different energies a, Tandem-MS of a single charged species at 731 *m/z* (▶). Recoded in positive mode with a higher-energy collision dissociation (HCD) of 35 eV (top left) and **b**, 50 eV (top right) in the relevant range from 50 – 750 *m/z*. The expansions visualize the range from 550 – 700 *m/z*. The heavier fragments (❖and✱) are observed in the lower energy spectrum (a) and the smaller fragments (●and◀) in the higher energy spectrum (b) exclusively. **Table (bottom):** includes peak assignment of the ESI-MS/MS spectrum at 731 *m/z* (▶) with a higher-energy collision dissociation (HCD) of 35 eV (a) compared to 50 eV (b). The resolution (obtained by the Xcalibur software), the experimental *m/z* vs. the theoretical *m/z* values, and Δ*m/z* for the proposed structure.

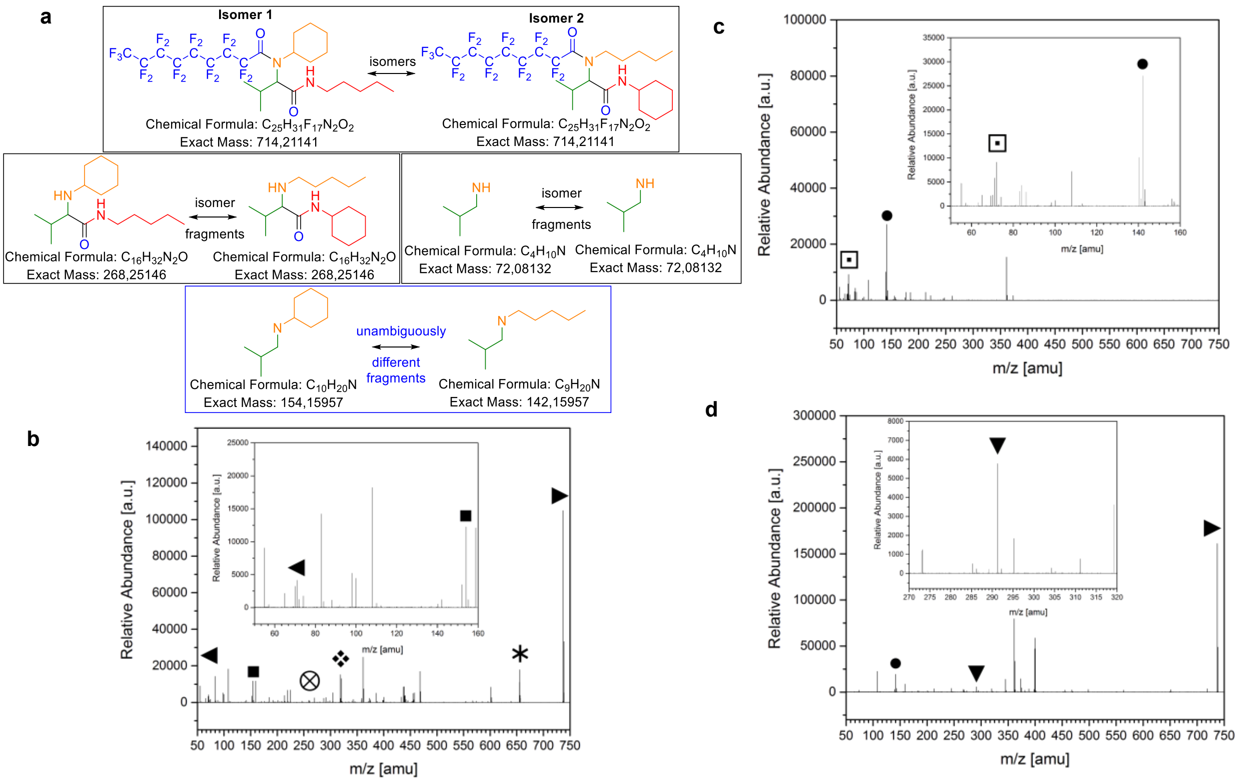

Entry	Label	Resolution	m/z(exp)	m/z(theo)	$\Delta m/z$	Formula	Structure
1 in **c**	▶	75000	737.2000	737.2011	0.0011	$C_{25}H_{31}O_2N_2F_{17}Na$	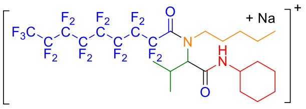
2 in **c**	▼	124000	291.2405	291.2412	0.0007	$C_{16}H_{32}ON_2Na$	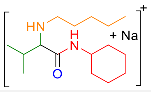
3 in **c**	●	172000	142.1591	142.1596	0.0005	$C_9H_{20}N$	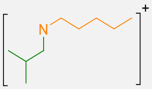
4 in **d**	⊡	252000	72.0815	72.0813	0.0002	$C_4H_{10}N$	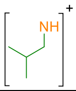
5 in **b**	▶	78000	737.2000	737.2011	0.0011	$C_{25}H_{31}O_2N_2F_{17}Na$	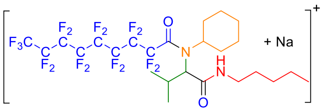
6 in **b**	∗	86000	655.1220	655.1229	0.0009	$C_{19}H_{21}O_2N_2F_{17}Na$	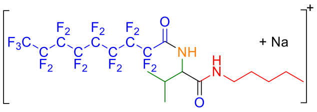
7 in **b**	❖	117000	318.2402	318.2401	0.0001	$C_{18}H_{33}O_2NNa$	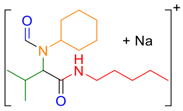
8 in **b**	⊗	137000	269.2588	269.2593	0.0005	$C_{16}H_{32}ON_2$	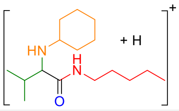
9 in **b**	■	177000	154.1592	154.1596	0.0004	$C_{10}H_{20}N$	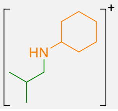
10 in **b**	◀	247000	72.0815	72.0813	0.0002	C_7H_8N	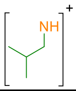

Supplementary Figure 9 | Differentiation of isomers. a, Two isomeric molecular keys and their respective fragments allowing differentiation. **b**, ESI-MS/MS of a single charged species at 737 *m/z* (▶) (Isomer 1). **c** and **d**, ESI-MS/MS of a single charged species at 737 *m/z* (▶) (Isomer 2). HCD = 25 eV (top) and 50 eV (bottom). The larger fragment (▼) is observed in the 25 eV spectrum (top) and the smaller fragments (⊡and●) in the 50 eV spectrum (bottom). **Table (bottom)**: includes fragment assignment.

Supplementary Methods

Exemplarily encoded messages and tandem-MS spectra of the respective molecular keys

Encoded messages and file container

The encrypted messages for following examples are included as ciphertext files (Supplementary Data 2) and can be decrypted utilizing the molecular encryption script (Supplementary Software 2). The respective decryption keys can be obtained by analyzing the corresponding tandem-MS spectra below with the analysis script (Supplementary Software 1). For this purpose start with the $[M+Na]^+$ ion from the ESI-MS spectra (intense signal at smaller *m/z*) and choose a ΔM of 0.002, then proceed by entering the pronounced fragment masses of the higher energy spectra, as previously described in the methods section. After determining the molecular key enter the alphanumerical codes into the molecular encryption script, the decrypted file can be exported as a *.txt file. The filecontainer included in the SI can be decrypted by utilizing all three molecular keys in sequential order. For accessing the files, save the decrypted version as a *.zip file.

Tandem-MS spectra for decrypting example 1

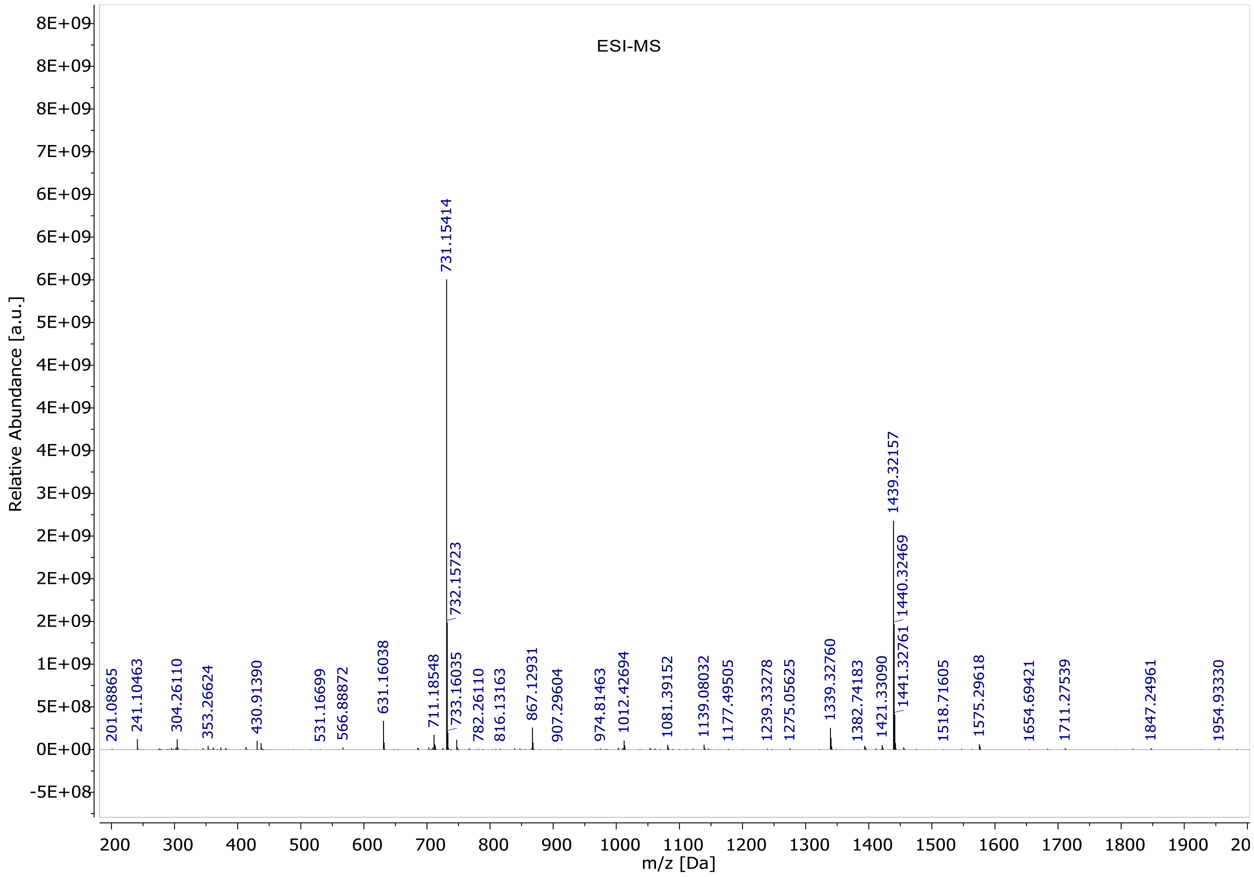

Supplementary Figure 10 | ESI-MS spectrum for example 1.

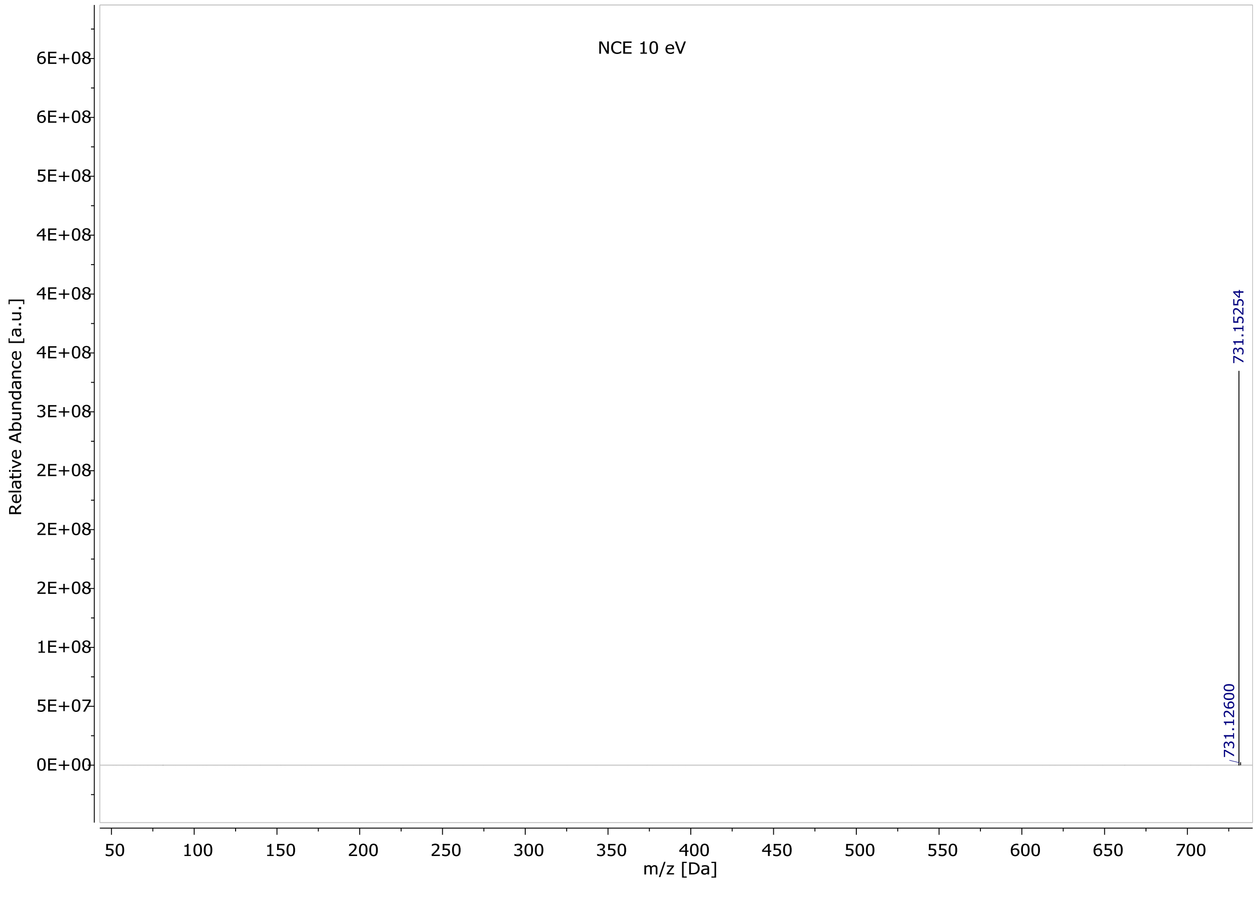

Supplementary Figure 11 | ESI-MS/MS spectrum of example 1. NCE =10 eV.

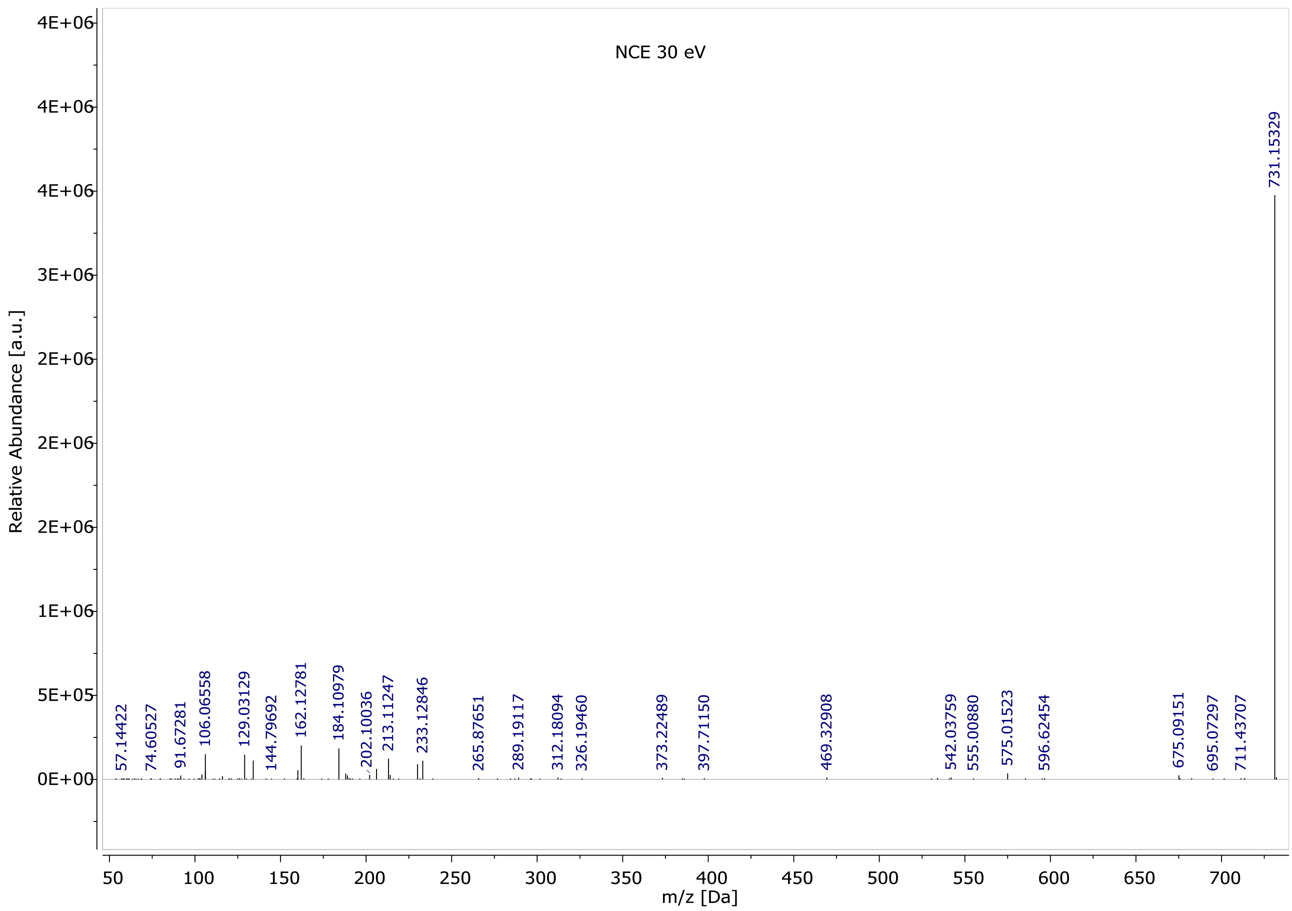

Supplementary Figure 12 | ESI-MS/MS spectrum of example 1. NCE =30 eV.

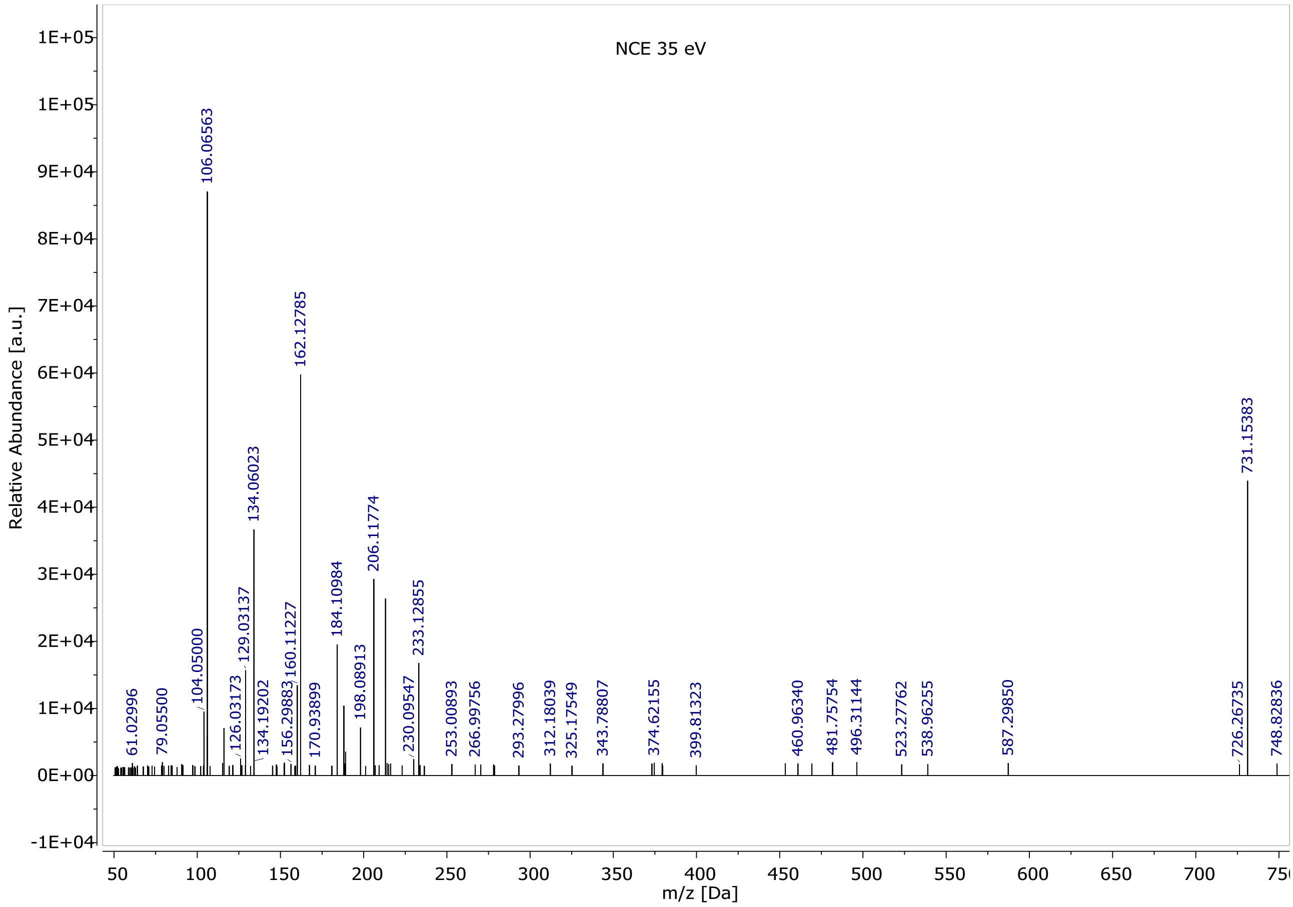

Supplementary Figure 13 | ESI-MS/MS spectrum of example 1. NCE =35 eV.

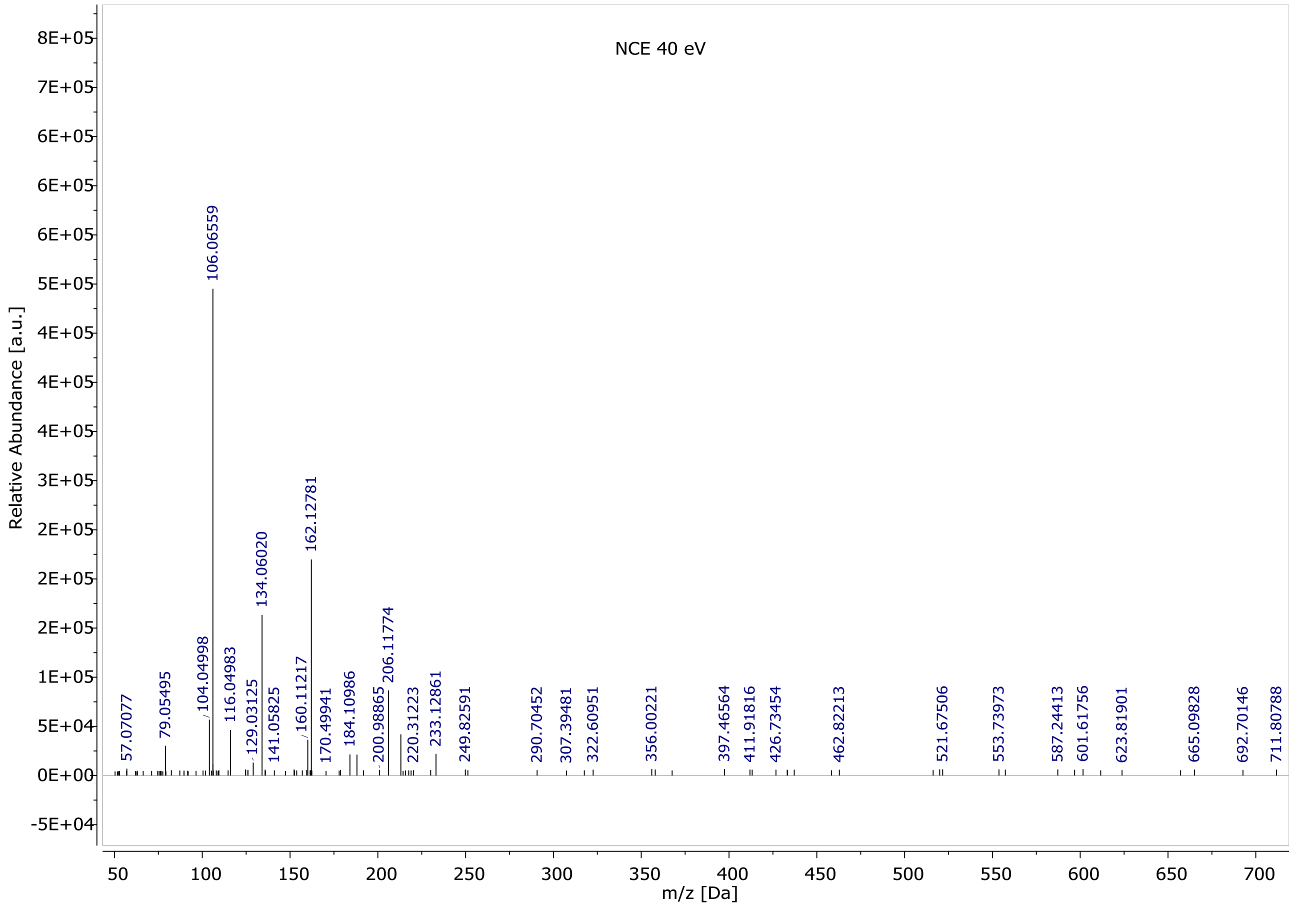

Supplementary Figure 14 | ESI-MS/MS spectrum of example 1. NCE =40 eV.

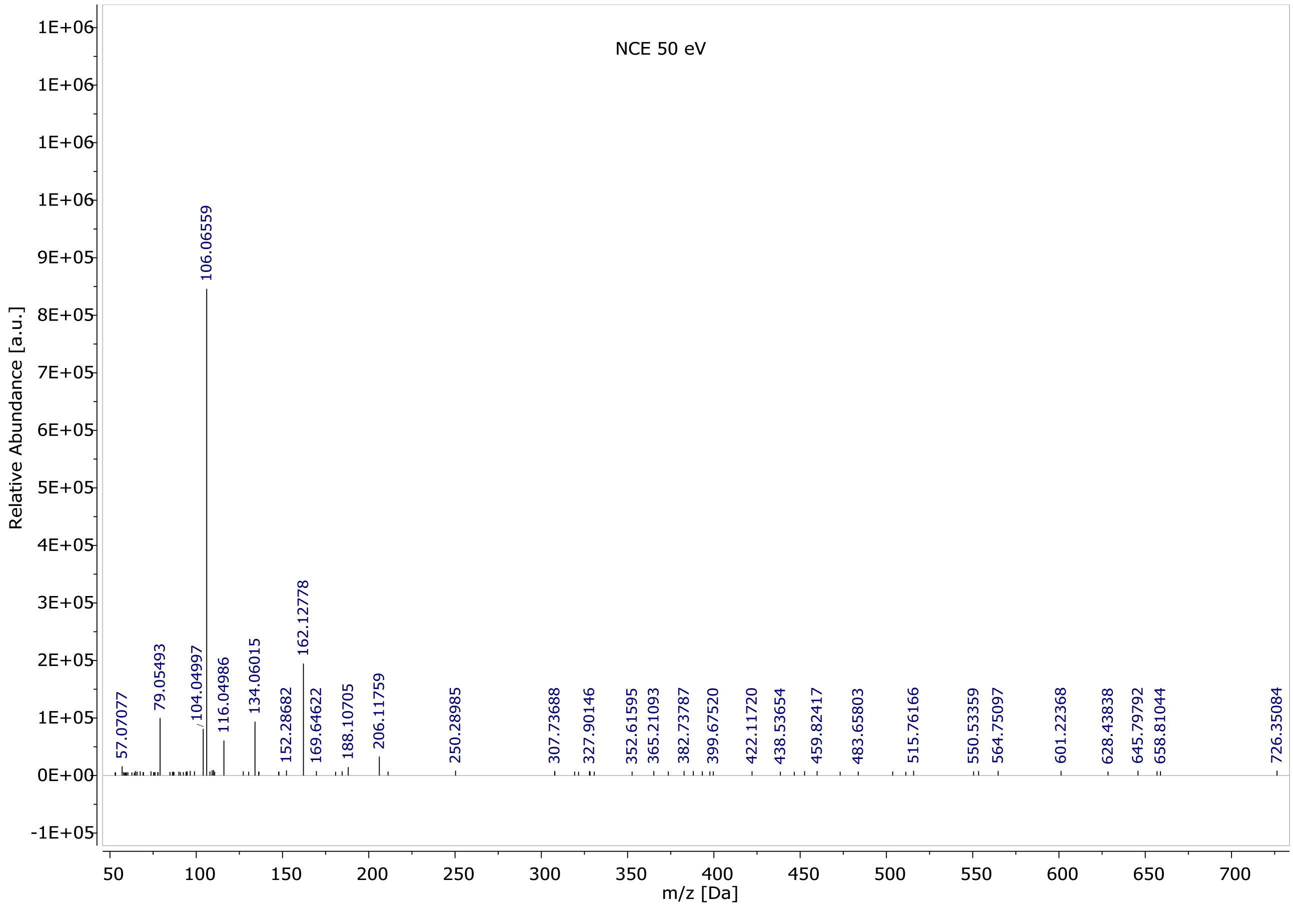

Supplementary Figure 15 | ESI-MS/MS spectrum of example 1. NCE =50 eV.

Tandem-MS spectra for decrypting example 2

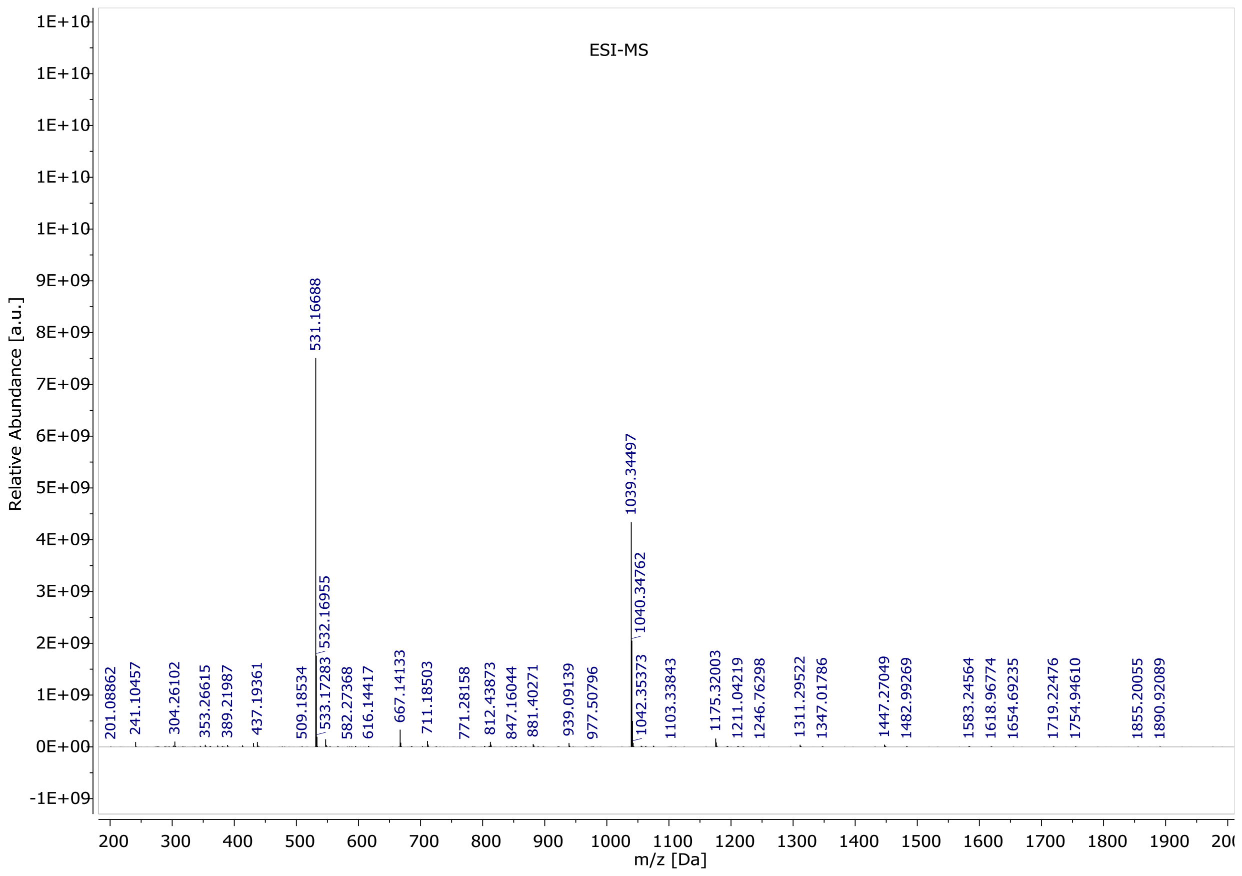

Supplementary Figure 16 | ESI-MS spectrum for example 2.

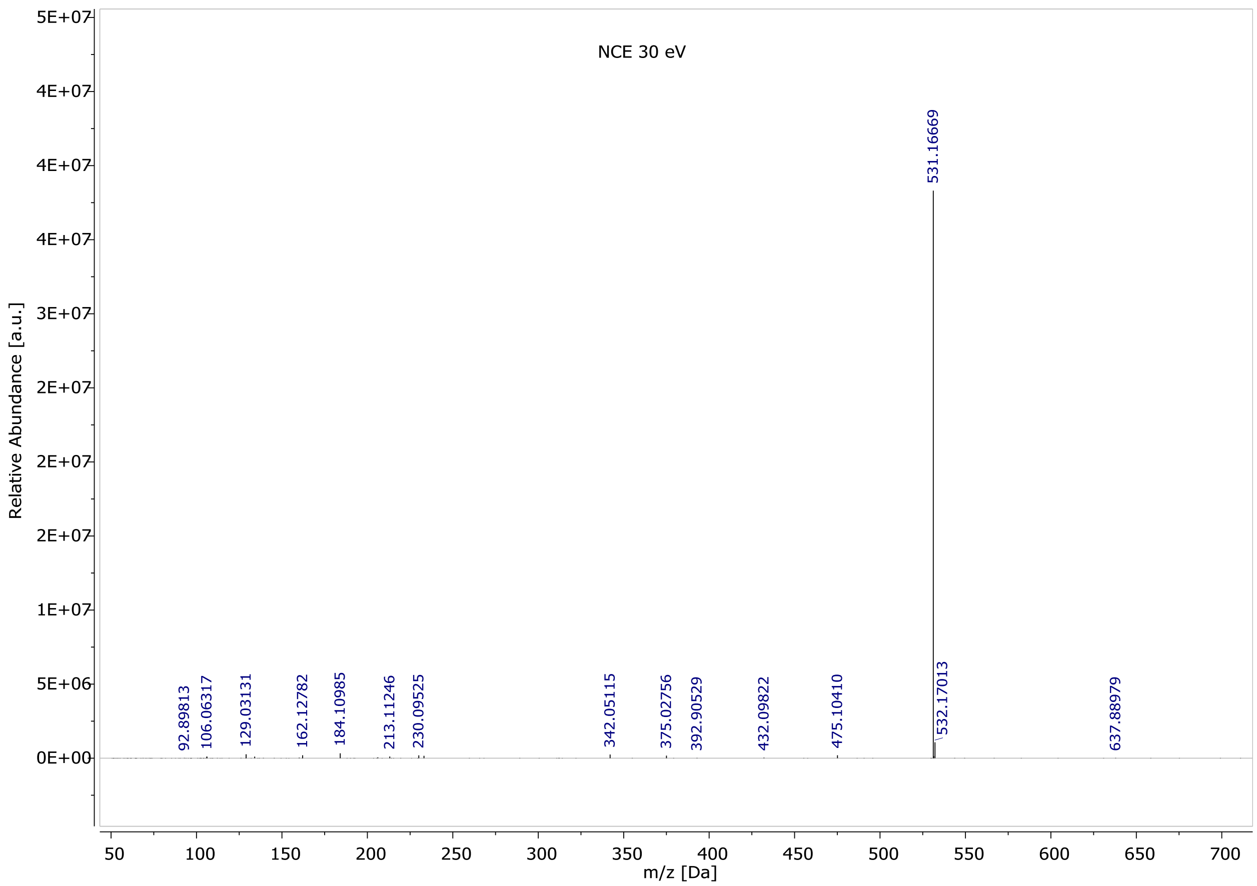

Supplementary Figure 17 | ESI-MS/MS spectrum of example 2. NCE =30 eV.

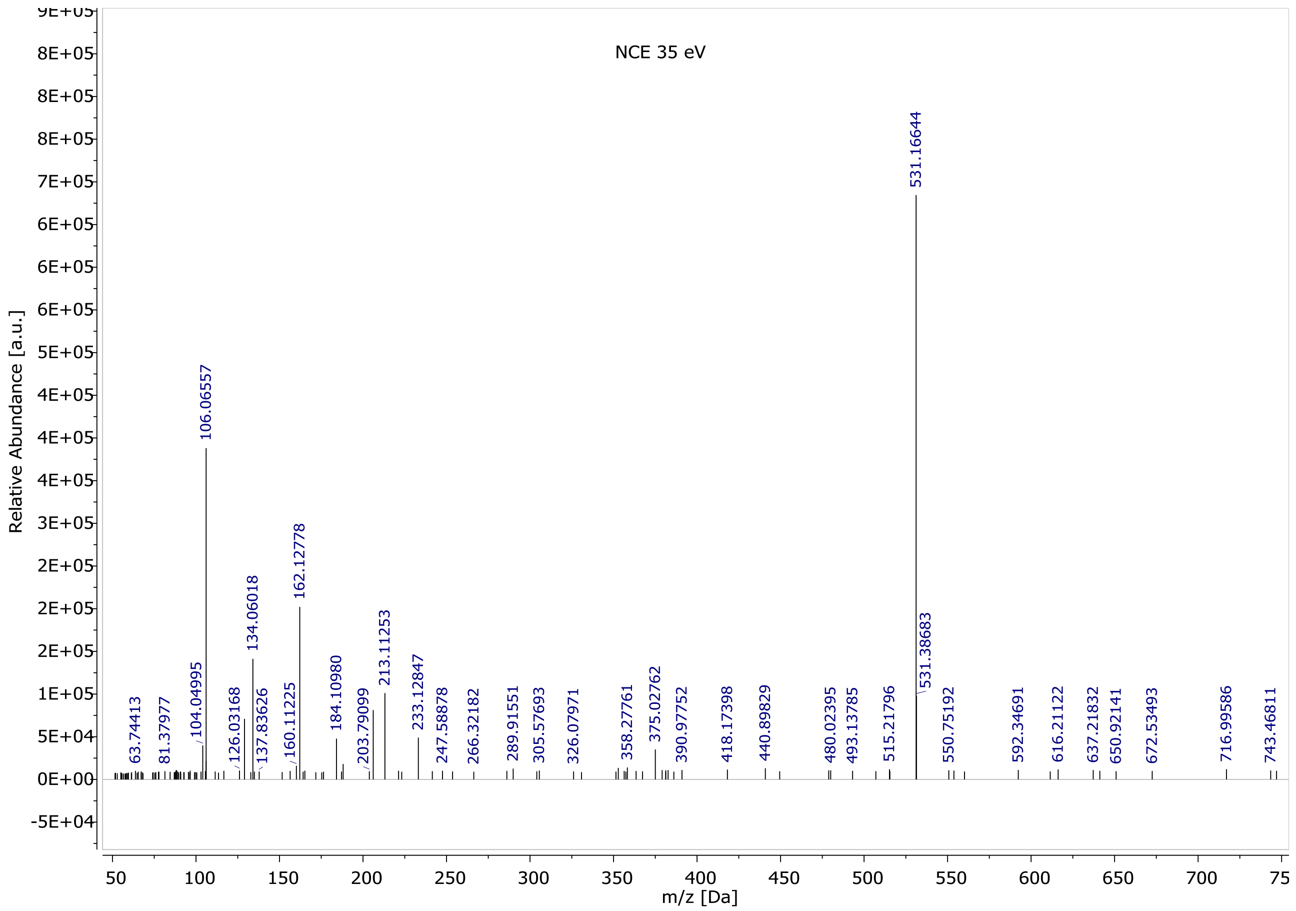

Supplementary Figure 18 | ESI-MS/MS spectrum of example 2. NCE =35 eV.

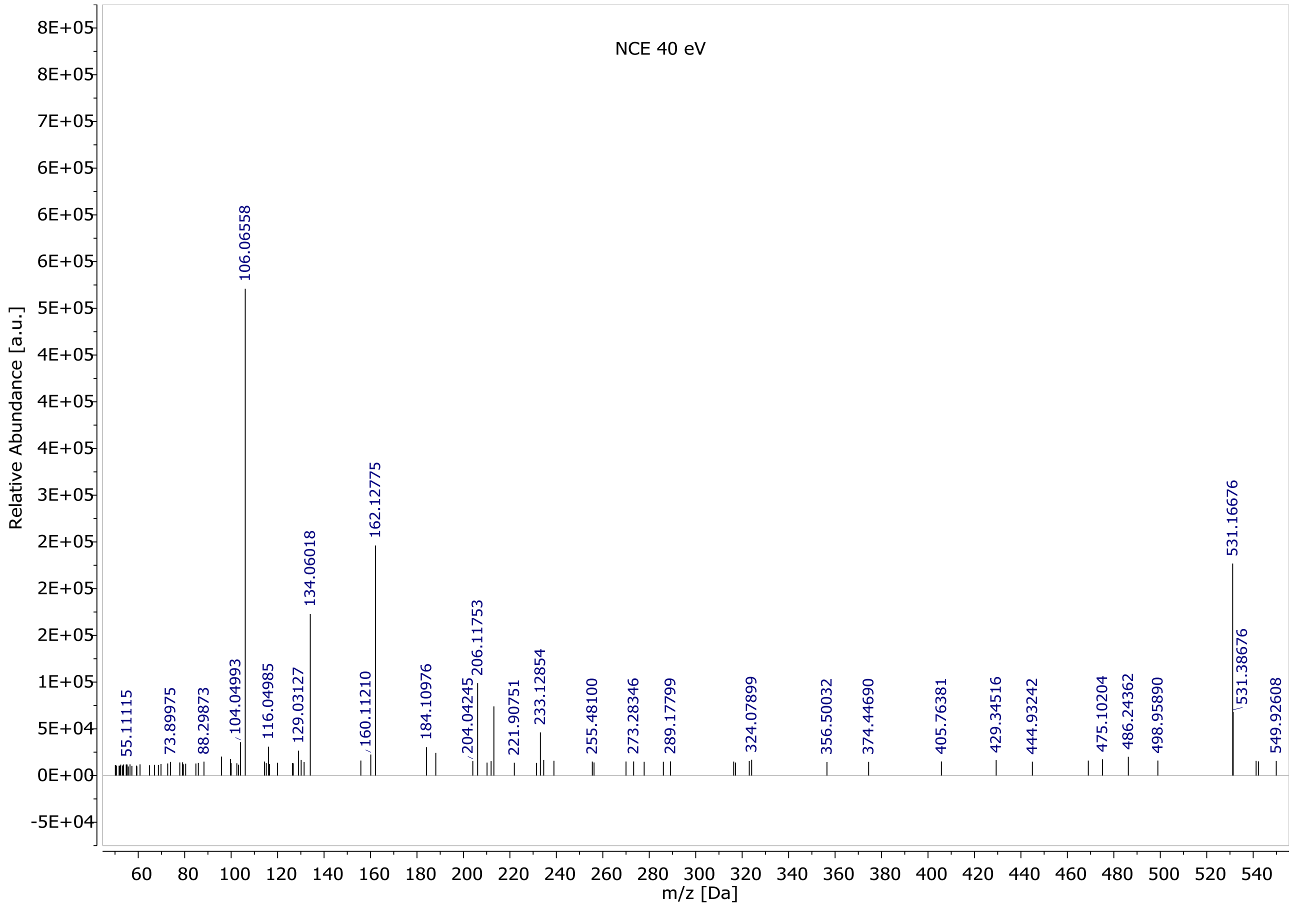

Supplementary Figure 19 | ESI-MS/MS spectrum of example 2. NCE =40 eV.

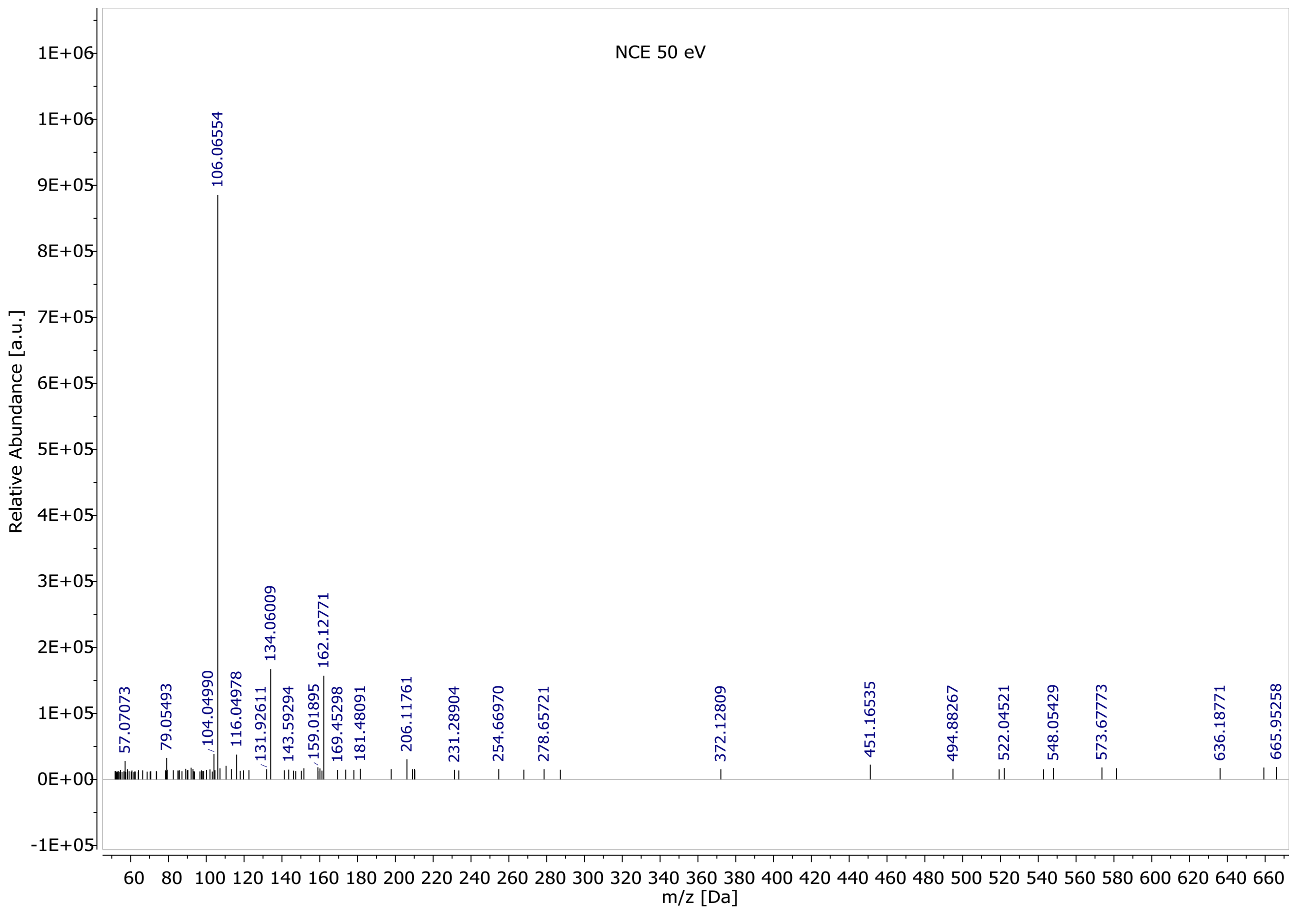

Supplementary Figure 20 | ESI-MS/MS spectrum of example 2. NCE =50 eV.

Tandem-MS spectra for decrypting example 3

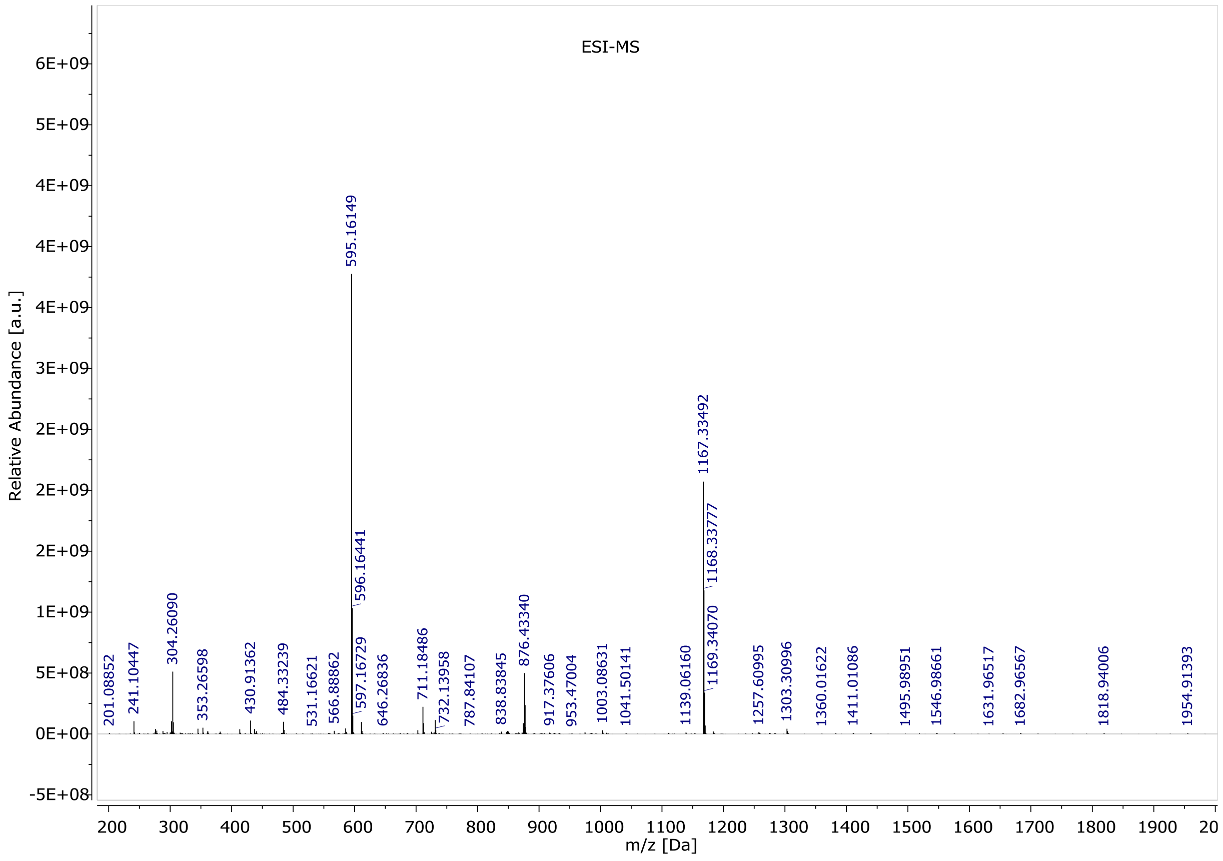

Supplementary Figure 21 | ESI-MS spectrum for example 3.

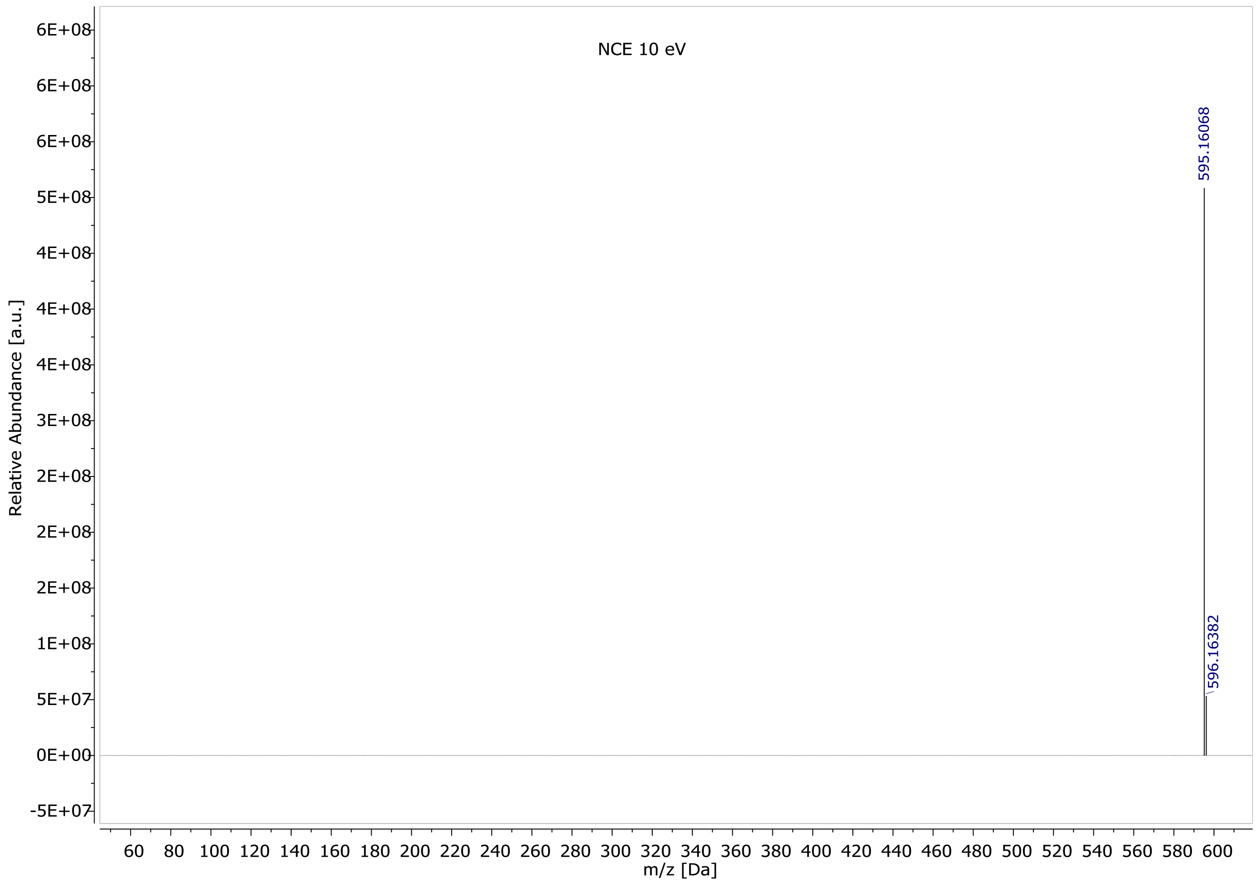

Supplementary Figure 22 | ESI-MS/MS spectrum of example 3. NCE =10 eV.

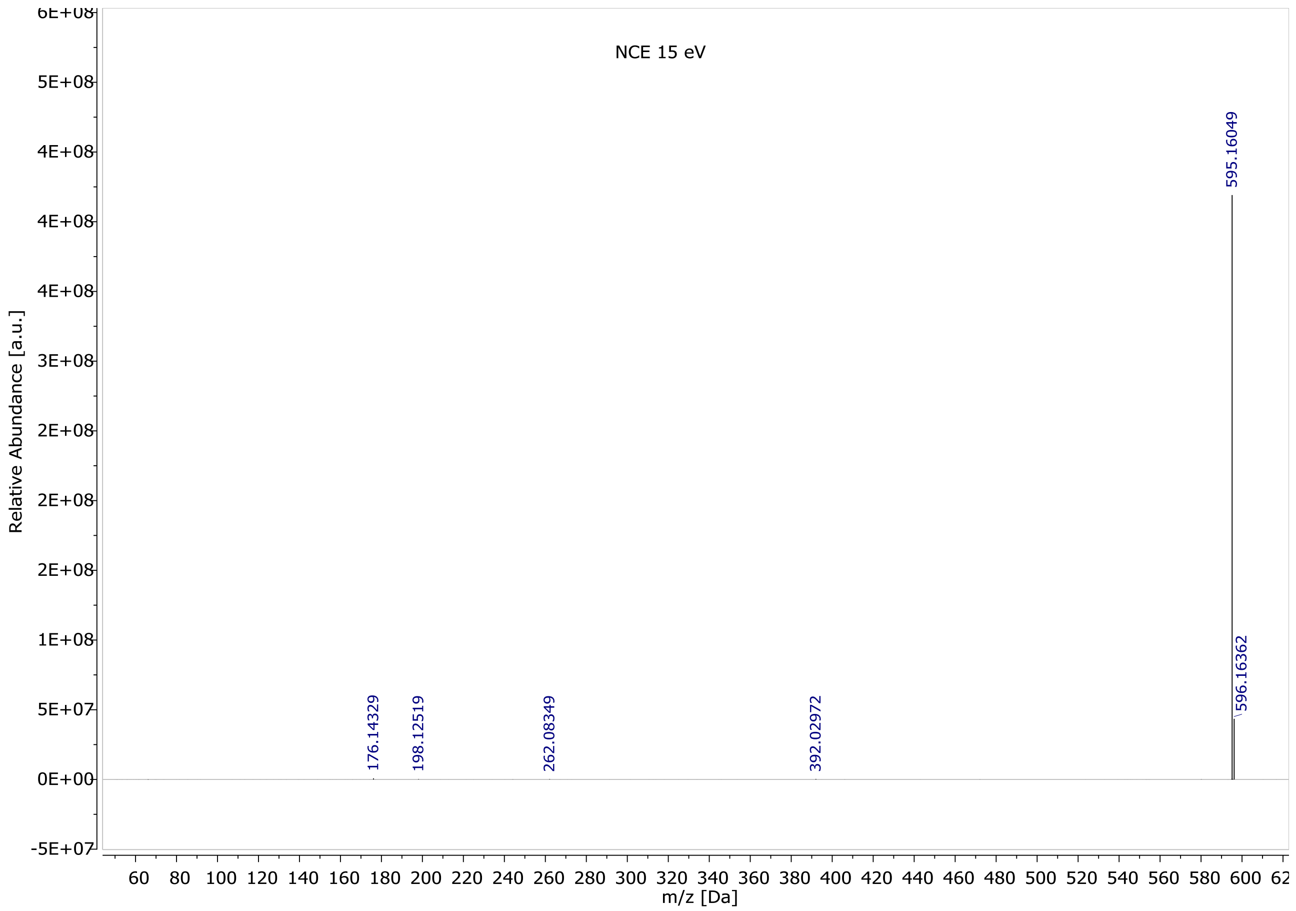

Supplementary Figure 23 | ESI-MS/MS spectrum of example 3. NCE =15 eV.

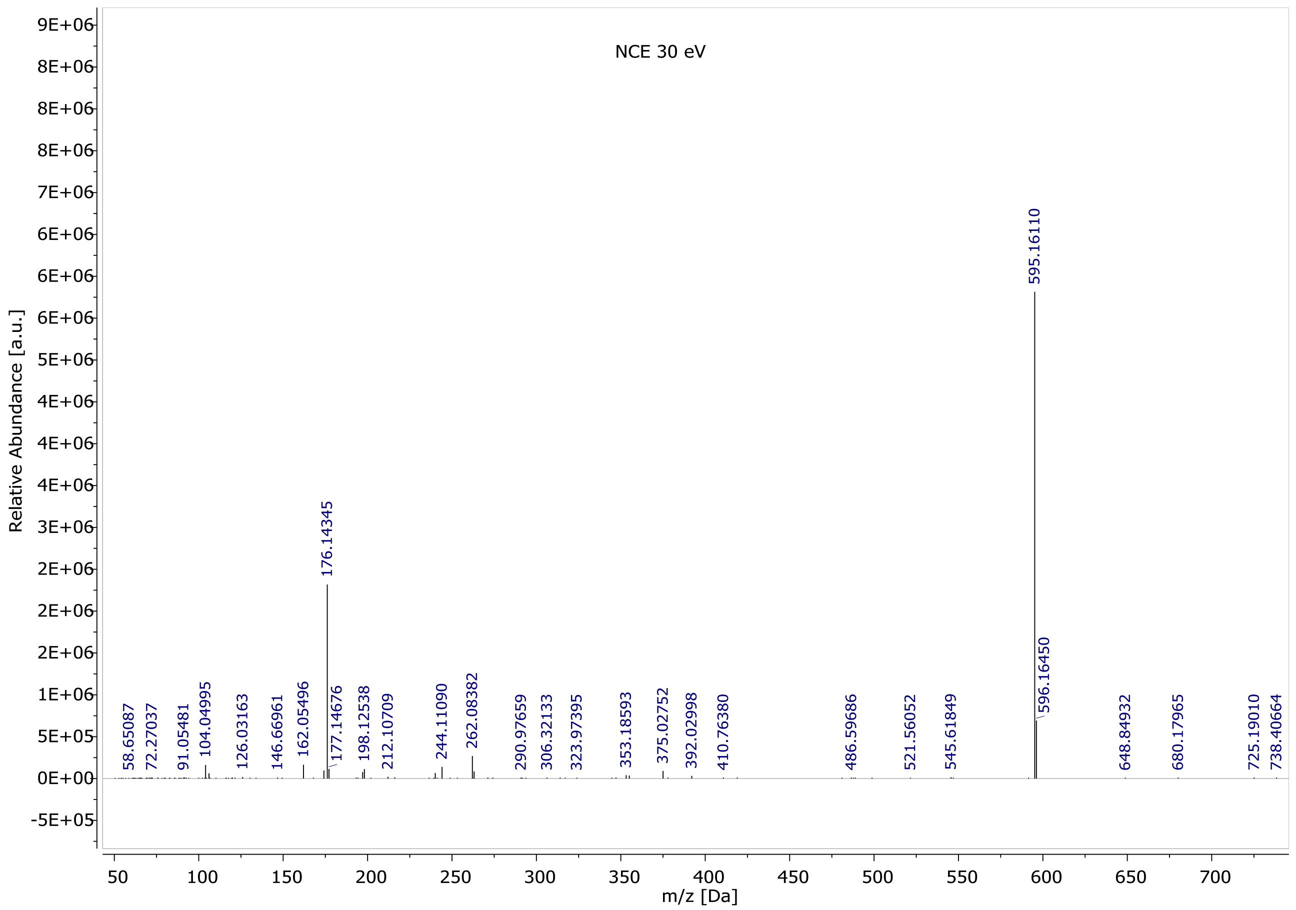

Supplementary Figure 24 | ESI-MS/MS spectrum of example 3. NCE =30 eV.

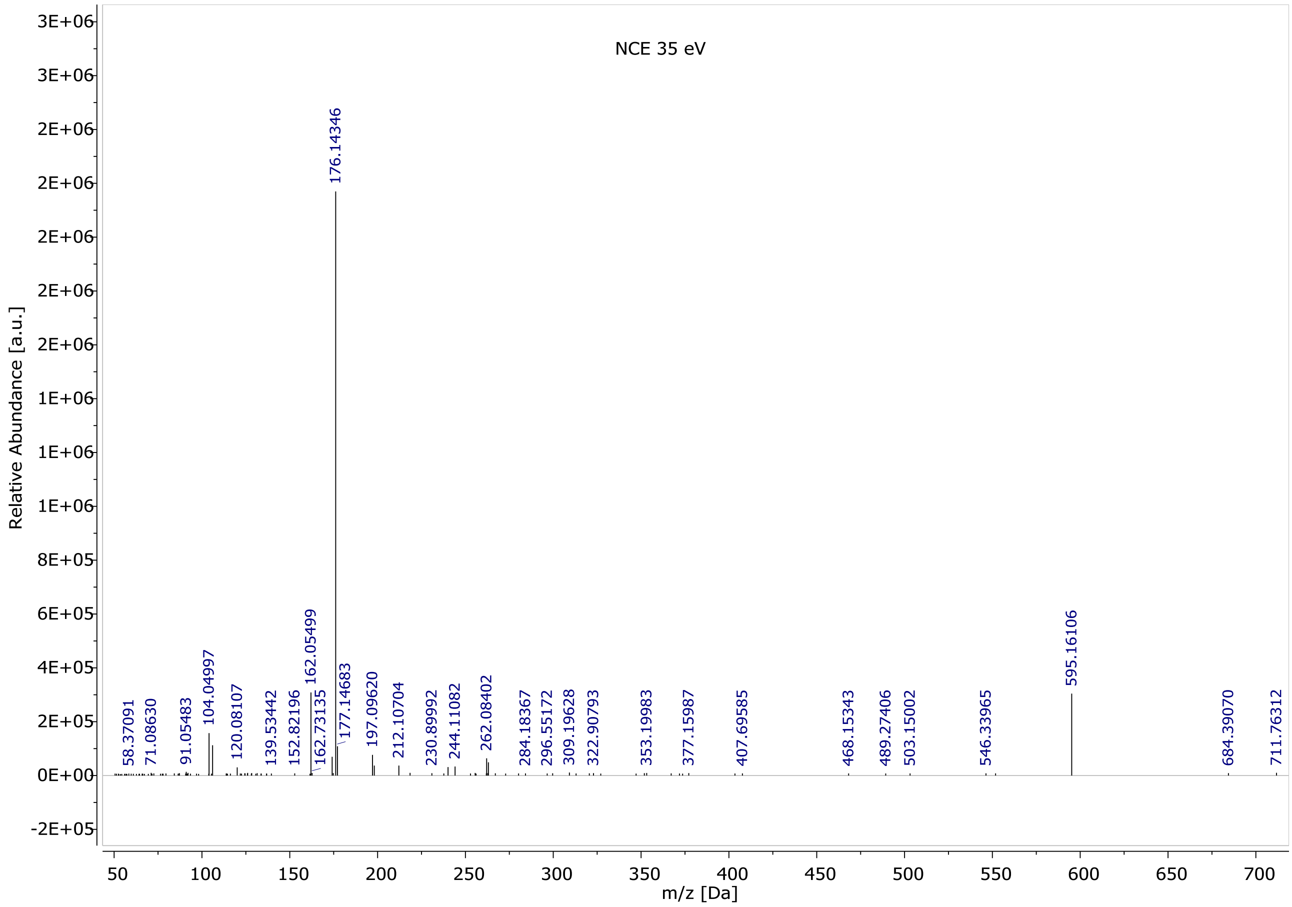

Supplementary Figure 25 | ESI-MS/MS spectrum of example 3. NCE =35 eV.

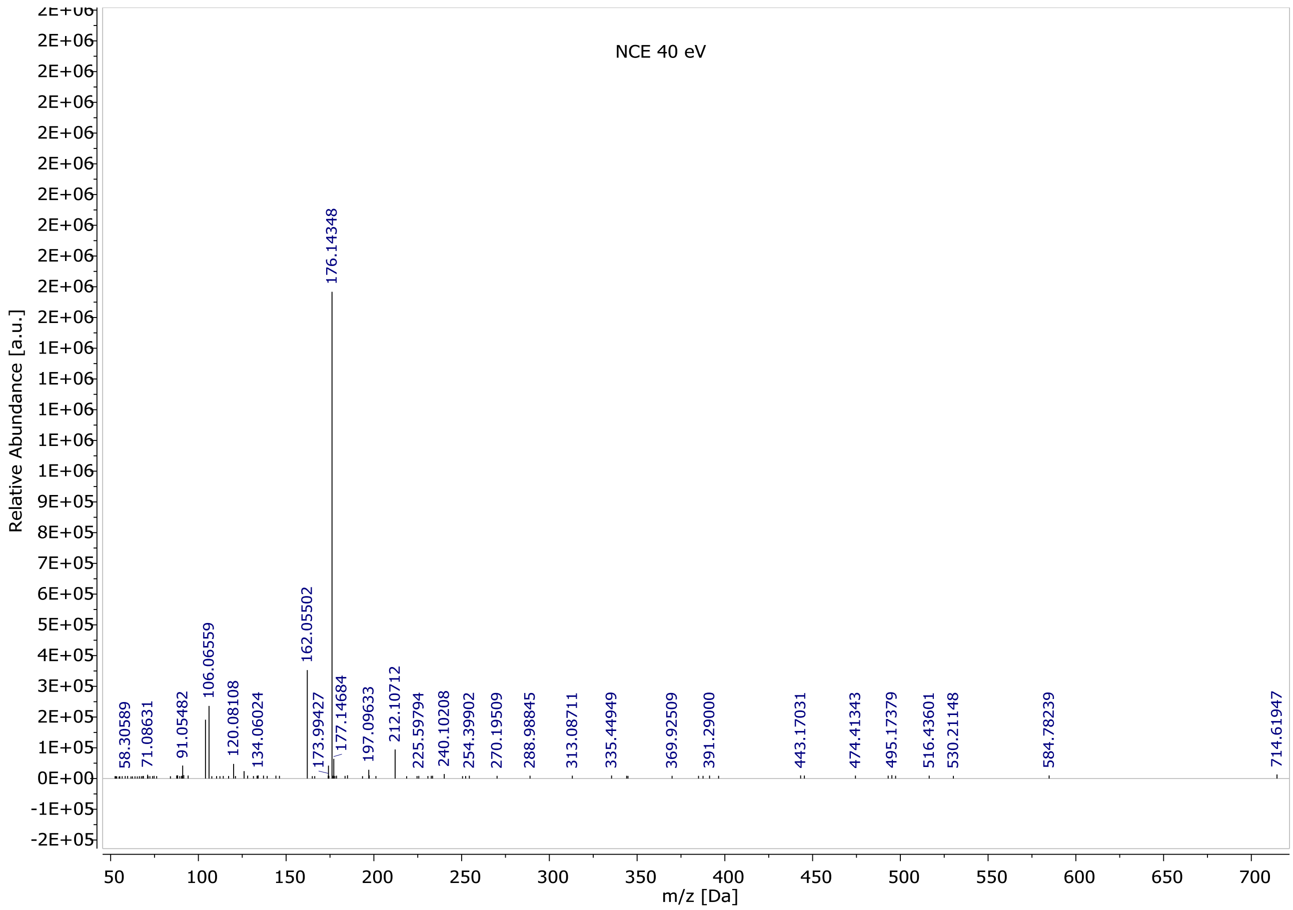

Supplementary Figure 26 | ESI-MS/MS spectrum of example 3. NCE =40 eV.

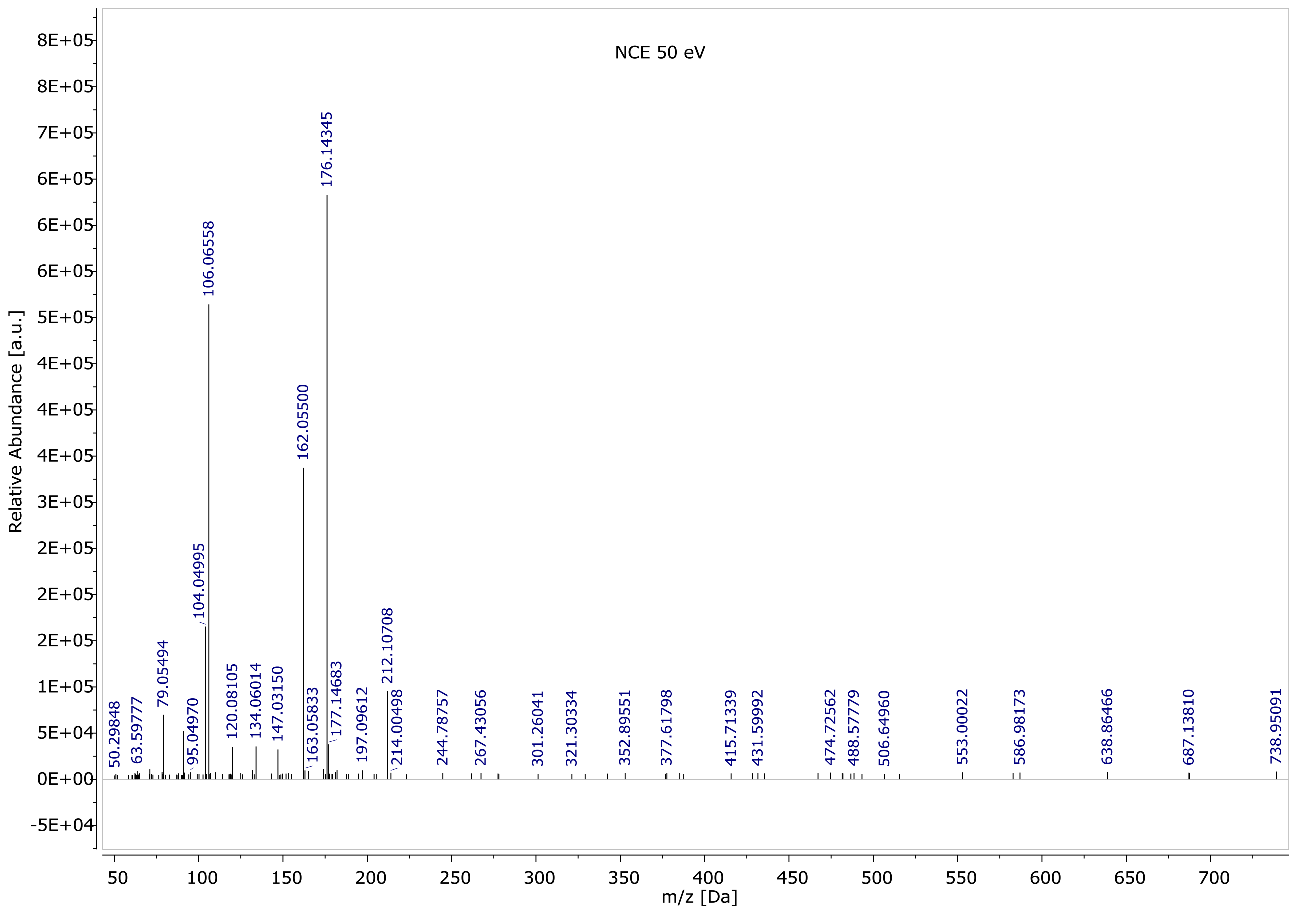

Supplementary Figure 27 | ESI-MS/MS spectrum of example 3. NCE =50 eV.

Experimental Part

General

All technical solvents were used, if not explicitly described otherwise, without further purification. Ethyl acetate, tetrahydrofuran, acetone and hexanes were pre-distilled. All commercially available chemicals were used, unless otherwise stated, without further purification and purchased from SIGMA ALDRICH at the highest commercial quality. Aldehydes were tested for oxidative contaminations (carboxylic acids) before use *via* TLC and ^{1}H NMR. Flash column chromatography was performed utilizing Merck SiO_2 60 (230 − 400 mesh);[1] for TLC analysis, precoated aluminum foils with fluorescence indicator from MERCK (TLC Silica gel 60, F_{254}, layer thickness: 0.25 mm) were employed as stationary phase. The spots were firstly visualized by fluorescence quenching under UV-light (λ = 254 nm), fluorescence (λ = 365 nm), and afterwards by staining with Seebach reagent: solution of 2.50 g cerium(IV) sulfate tetrahydrate ($Ce(SO_4)_2 \cdot 4H_2O$), 6.25 g ammonium heptamolybdate tetrahydrate ($(NH_4)_6Mo_7O_{24} \cdot 4H_2O$), 225 mL water and 25.0 mL concentrated sulfuric acid or potassium permanganate: solution of 3.00 g potassium permanganate ($KMnO_4$), 20.0 g potassium carbonate (K_2CO_3) and 5.00 mL of a 5 wt.% sodium hydroxide (NaOH)-solution in 300 mL water.

^{1}H and ^{13}C NMR spectra were recorded on BRUKER Avance DPX spectrometers (Billerica, MA) with a 5-mm dual proton/carbon probe (300 and 400 MHz), on a Bruker Avance III with a 5 mm z-gradient cryogenically cooled probe head (CPTCI, 600 MHz ^{1}H/75.5 MHz) or on a 500 MHz WB Bruker Avance I spectrometer with a proton frequency of 499.97 MHz, ^{13}C frequency of 125.72 MHz on a 8 mm TXI probe head with actively shielded z-gradients (at $\Theta = 0°$) and on a 4 mm triple HCX MAS probe head (at ca. $\Theta = 65°$) at 298 K, regulated with a Bruker VTU-3000. Unless otherwise stated, all spectra were measured at ambient temperature. The chemical shift for ^{1}H-NMR spectra was reported in parts per million (ppm) referenced to characteristic solvent signals of partly deuterated solvents *e.g.* $CDCl_3$ at 7.26 ppm or the centroid peak of the DMSO-d^6 quintet at 2.50 ppm. ^{13}C-NMR spectra were reported in ppm relative to characteristic signals of partly deuterated solvents, *e.g.* the centroid peak of the $CDCl_3$ triplet at 77.00 ppm or the DMSO-d^6 septet at 39.52 ppm. All ^{13}C spectra are decoupled from ^{1}H signals. The signals were listed from low field (large ppm) to high filed (small ppm) with the following notation: NMR-active nucleus (frequency [MHz], deuterated solvent): δ [ppm] = chemical shift (spin multiplicity, scalar coupling constant J [Hz], integral/number of nuclei, assignment $^{Atom\ position}$). The spin multiplicity and corresponding signal patterns were abbreviated as follows: s = singlet, d = doublet, t = triplet, q = quartet, quint. = quintet, m = multiplet, br s = brought singlet. Coupling constants J were noted in Hz. 2D NMR methods *i.e.* heteronuclear multiple quantum coherence (HMQC), heteronuclear single quantum coherence (HSQC), heteronuclear multiple bond correlation (HMBC), correlated spectroscopy (COSY) or nuclear overhauser enhancement spectroscopy (NOESY) were carried out, if necessary, for signal assignment and structure elucidation.

Fast-atom-bombardment (FAB) and electron ionization (EI) spectra were recorded utilizing a Finnigan MAT 95 mass spectrometer. Molecule fragmentations observed in FAB or EI measurements were formally denoted as homolytic bond cleavage to allow a simple illustration of the observed *m/z* species, but a radical mechanism (or formation) was not proven.

Infrared (IR) spectra were recorded on a BRUKER Alpha-p instrument applying ATR-technology. The signals were noted from large to smaller wavenumbers with the following notation: IR (Type of measurement) ν [cm^{-1}] = Wave number (signal intensity, molecular oscillation assignment). The signal shape and intensity is reported relative to the signal of highest intensity and was abbreviated in the following pattern: br = brought, vs = very strong, s = strong, m = medium, w = weak, vw = very weak.

GC-MS (electron impact (EI)) analyses were conducted using a Varian 431-GC instrument with a capillary column FactorFour^{TM} VF-5ms (30 m · 0.25 mm · 0.25 µm) and a Varian 210-MS ion trap mass detector. Scans were performed from 40 to 650 m/z at rate of 1 scan per second. The oven temperature program applied during the analysis was: initial temperature 95 °C, hold for 1 min, ramp

at 15 °C·min^{-1} to 200 °C, hold for 2 min., ramp at 15 °C·min^{-1} to 300 °C, hold for 5 min. The injector transfer line temperature was set to 250 °C. Measurements were performed in the split-split mode (split ratio 50:1) using helium as carrier gas (flow rate 1.0 mL·min^{-1}).

ESI-MS and ESI-MS-MS spectra were recorded on a Q Exactive (Orbitrap) mass spectrometer (Thermo Fisher Scientific, San Jose, CA, USA) equipped with a HESI II probe to record high resolution electrospray ionization–MS (ESI-MS). Calibration was carried out in the *m/z* range 74–1.822 using premixed calibration solutions (Thermo Fisher Scientific). A constant spray voltage of 4.7 kV and a dimensionless sheath gas of 5 were employed. The S-lens RF level was set to 62.0, while the capillary temperature was set to 250 °C. All samples were dissolved at a concentration range of 0.05 – 0.01 mg mL^{-1} in a mixture of THF and MeOH (3:2) doped with 100 µmol sodium trifluoroacetate and injected with a flow of 5 µL min^{-1}.

Synthetic procedures

Ugi reaction of perfluorononanoic acid, benzaldehyde, *tert*-butylisocyanide and butylamine

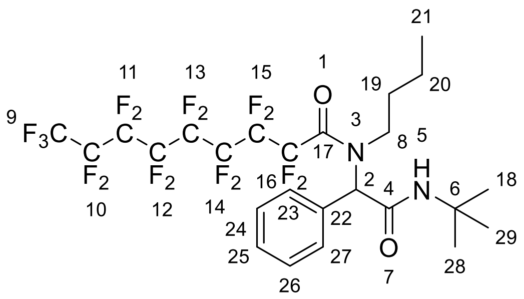

In a 25 mL round bottom flask benzaldehyde (50.0 µL, 52.0 mg, 490 µmol, 1.30 eq.) was dissolved in 1.5 mL methanol, subsequently butylamine (48.5 µL, 35.9 mg, 490 µmol, 1.30 eq.) was added and the resulting mixture was stirred for 60 min over sodium sulfate. Afterwards, the mixture was filtrated. The solid was washed with 10 mL methanol three times. Subsequently, the filtrate was concentrated under reduced pressure. Perfluorononanoic acid (175 mg, 377 µmol, 1.00 eq.) dissolved in 1 mL methanol was added to the imine at room temperature and the resulting mixture was stirred for 2 min. Subsequently, *tert*-butylisocyanide (51.2 µL, 37.6 mg, 453 µmol, 1.20 eq.) was added to the stirring mixture. The reaction was stirred for 18 h at room temperature. The crude reaction mixture was dried under reduced pressure and purified *via* column chromatography employing Fluoro*Flash*® silica gel. The fluorous fraction was concentrated and the residue was adsorbed onto celite® and purified *via* column chromatography employing silica gel and eluting with a gradual solvent mixture of ethyl acetate and *c*-hexane (0:1 → 1:1) to yield the Ugi product as a pale highly viscous oil (59.4 mg, 83.7 µmol, 22.2%).

R_f = 0.50 in *c*-hexane/ethyl acetate (6:1). Visualized *via* fluorescent quench and Seebach staining solution.

IR (ATR): ν [cm^{-1}] = 3320.6 (w, ν(N-H)), 2968.3 (w, ν(C-H)), 1675.7 (m, ν(C=O)), 1654.1 (m, ν(C=O)), 1553.5 (m), 1477.9 (vw), 1453.2 (w), 1429.2 (w), 1369.4 (w), 1330.5 (w), 1234.3 (m), 1202.1 (vs), 1148.3 (vs), 1111.3 (m), 987.4 (w), 968.1 (vw), 928.5 (w), 806.6 (vw), 772.64 (vw), 736.7 (w), 697.8 (w), 655.0 (m), 631.3 (m), 611.6 (w), 564.8 (w), 519.9 (s), 496.1 (w), 439.3 (vw).

^{1}H NMR (400 MHz, $CDCl_3$): δ [ppm] = 7.64 – 7.31 (m, 5 H, $CH_{Ar}^{23\text{-}27}$), 5.74 – 5.36 (m, 2 H, NH^{5} + CH^{2}), 3.78 – 3.00 (m, 2 H, CH_2^{8}), 1.47 – 1.18 (m, 9 H, $CH_3^{18,28,29}$), 1.14 – 0.96 (m, 4 H, $CH_2^{19,20}$), 0.67 (t, J = 7.2 Hz, 3 H, CH_3^{21}).

^{13}C NMR (101 MHz, $CDCl_3$): δ [ppm] = 166.2 (s, $CONR^{4}$), 158.0 (s, $CONR^{17}$), 132.9 (s, C_{Ar}^{22}), 132.6 (s, CH_{Ar}), 128.6 (s, CH_{Ar}), 128.1 (s, CH_{Ar}), 128.1 (s, CH_{Ar}), 64.9 (s, CH^{2}), 51.2 (s, C^{6}),47.2 (s, CH_2^{8}), 30.9 (s, $CH_2^{20\ or\ 19}$), 27.9 (s, $CH_3^{18,\ 28,\ 29}$), 27.5 (s, $CH_3^{18,\ 28,\ 29}$), 18.9 (s, $CH_2^{20\ or\ 19}$), 12.3 (s, CH_3^{21}).

^{19}F NMR (376 MHz, CDCl$_3$): δ [ppm] = -85.11 (t, J = 10.3 Hz, 3 F, CF$_3$^{9}), AB-signal (δ_A = -113.09, δ_B = -114.08, J_{AB} = 297.4 Hz, A and B are split into t, J = 13.1 Hz, CF$_2$^{16a}), AB-signal (δ_A = -115.56, δ_B = -116.60, J_{AB} = 291.8 Hz, CF$_2$^{16b}, additional coupling not resolved, signals broadened), -124.62 (s, CF$_2$), -126.11 (s, CF$_2$), -127.05 (s, CF$_2$), -130.44 (s, CF$_2$^{10}). Total integral of CF$_2$ region normalized with respect to the CF$_3$^{9} group = 14.

FAB – MS [m/z] (relative intensity): 709.2 (35%) [M + H]$^+$, 637.1 (40%) [Fragment A – H]$^+$, 608.1 (55%) [Fragment A – CO]$^+$, 552.1 (20%) [Fragment A – CO – C_5H_9]$^+$, 191.1 (12%), [Fragment B + H]$^+$.

HRMS – FAB [m/z]: [M + H]$^+$ calculated for $^{12}C_{25}{}^{1}H_{26}{}^{16}O_2{}^{14}N_2{}^{19}F_{17}$, 709.1717; found, 709.1715; Δ = 0.19 mmu.

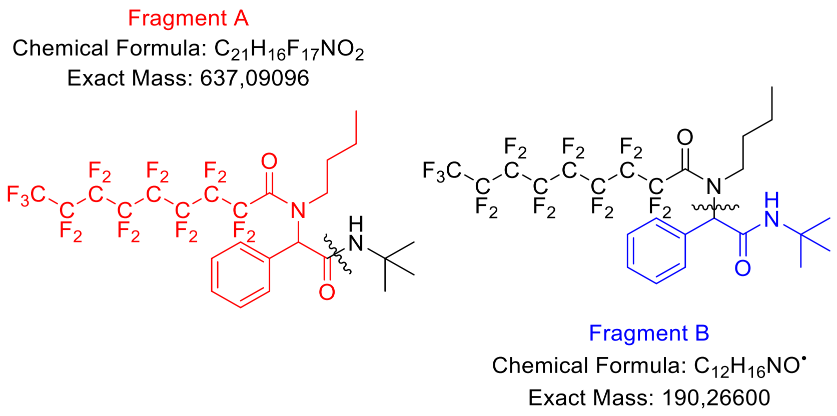

Supplementary Figure 28 | Proposed fragments observed in FAB-MS.

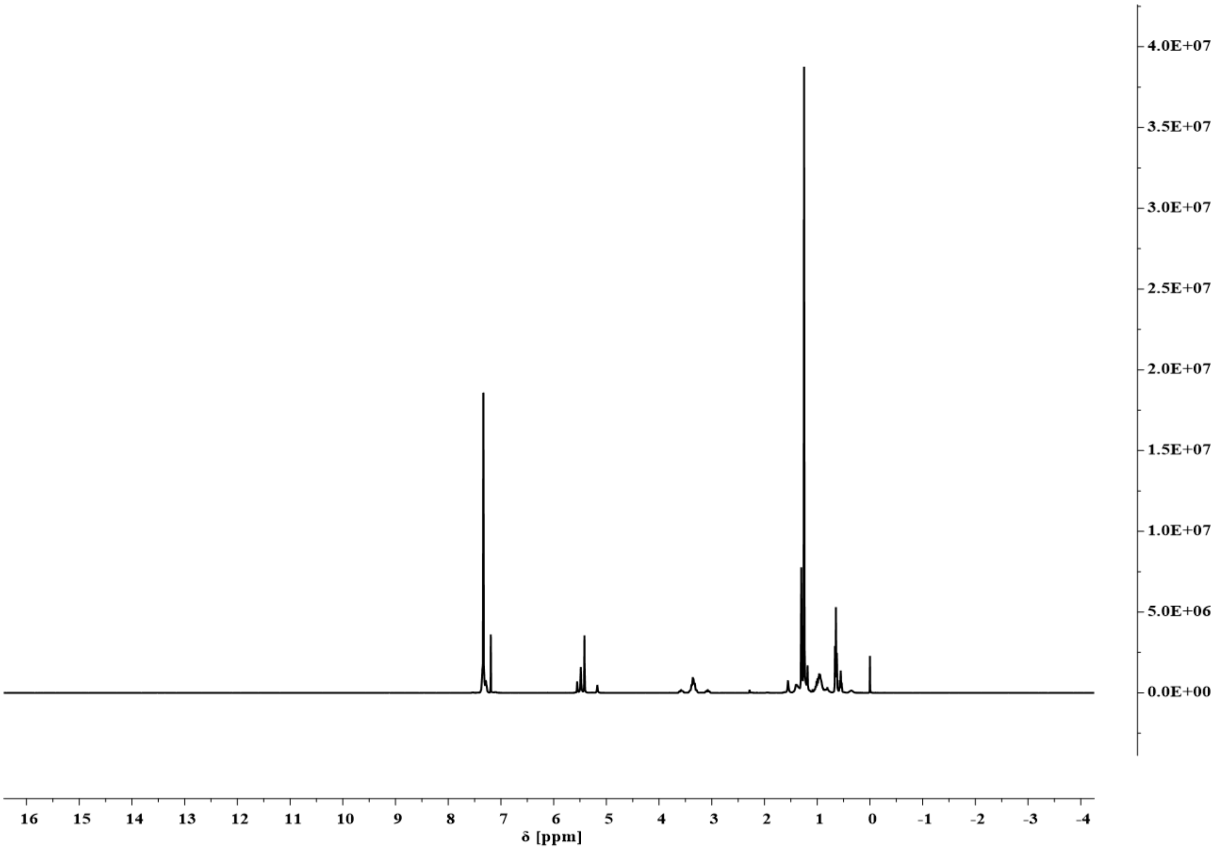

Supplementary Figure 29 | ^{1}H NMR of the title compound recorded in $CDCl_3$.

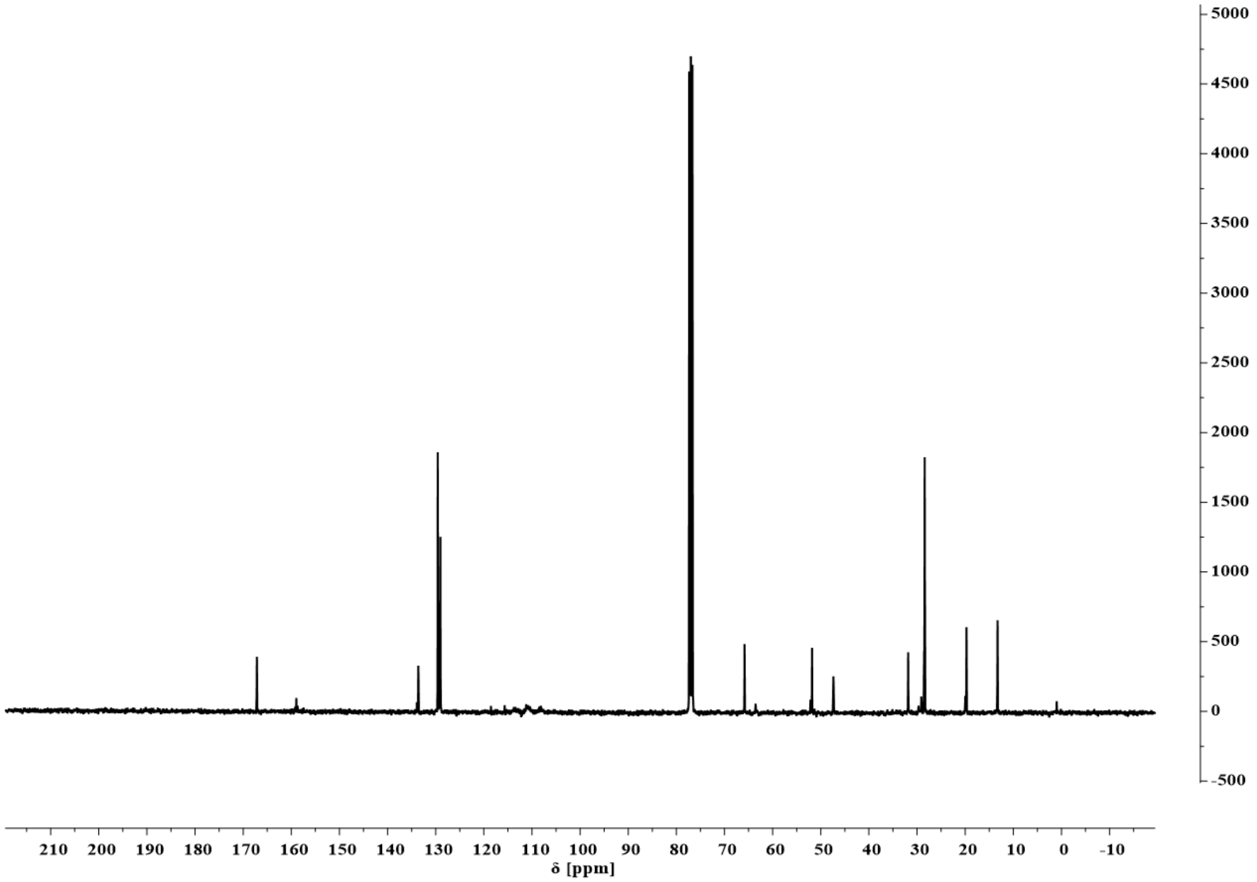

Supplementary Figure 30 | ^{13}C NMR of the title compound recorded in $CDCl_3$.

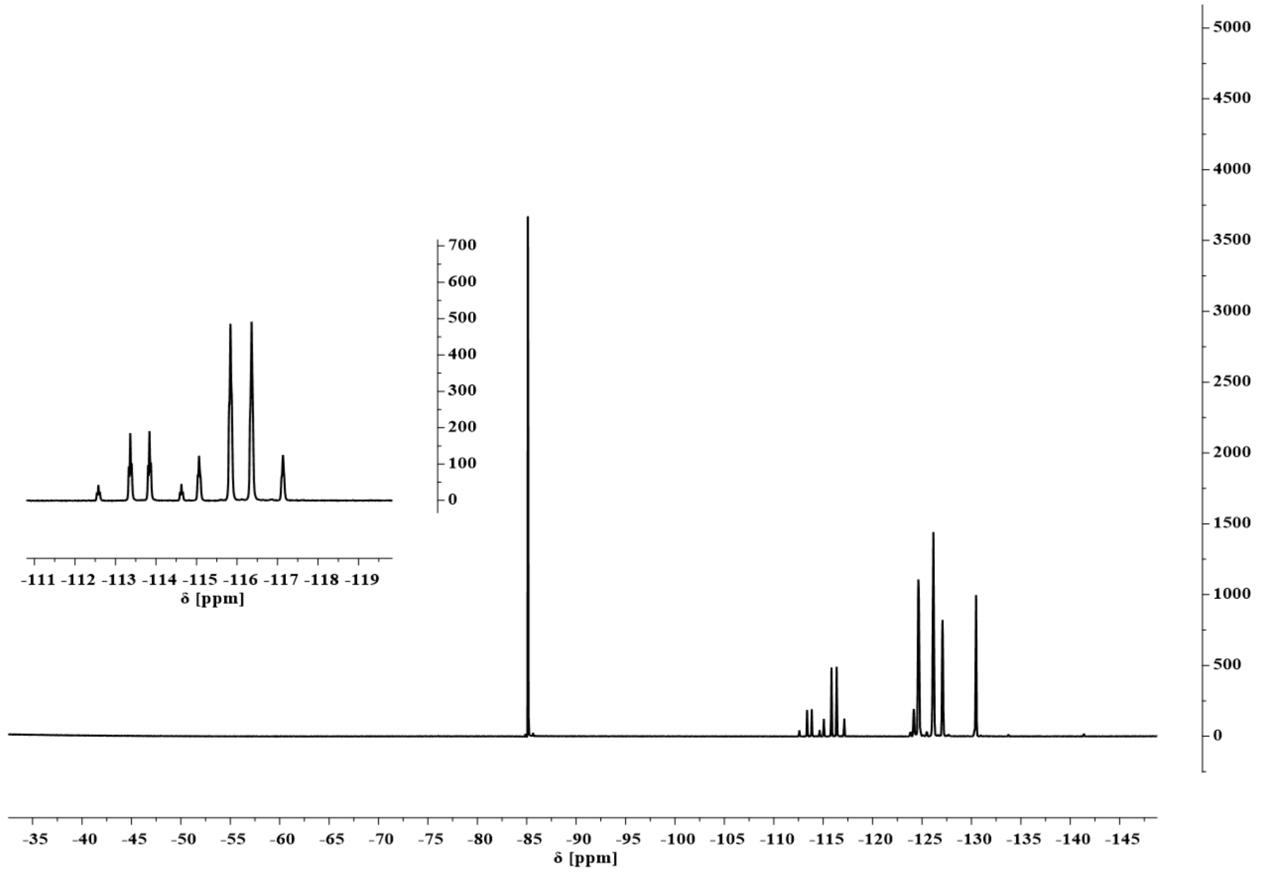

Supplementary Figure 31 | ^{19}F NMR of the title compound recorded in $CDCl_3$.

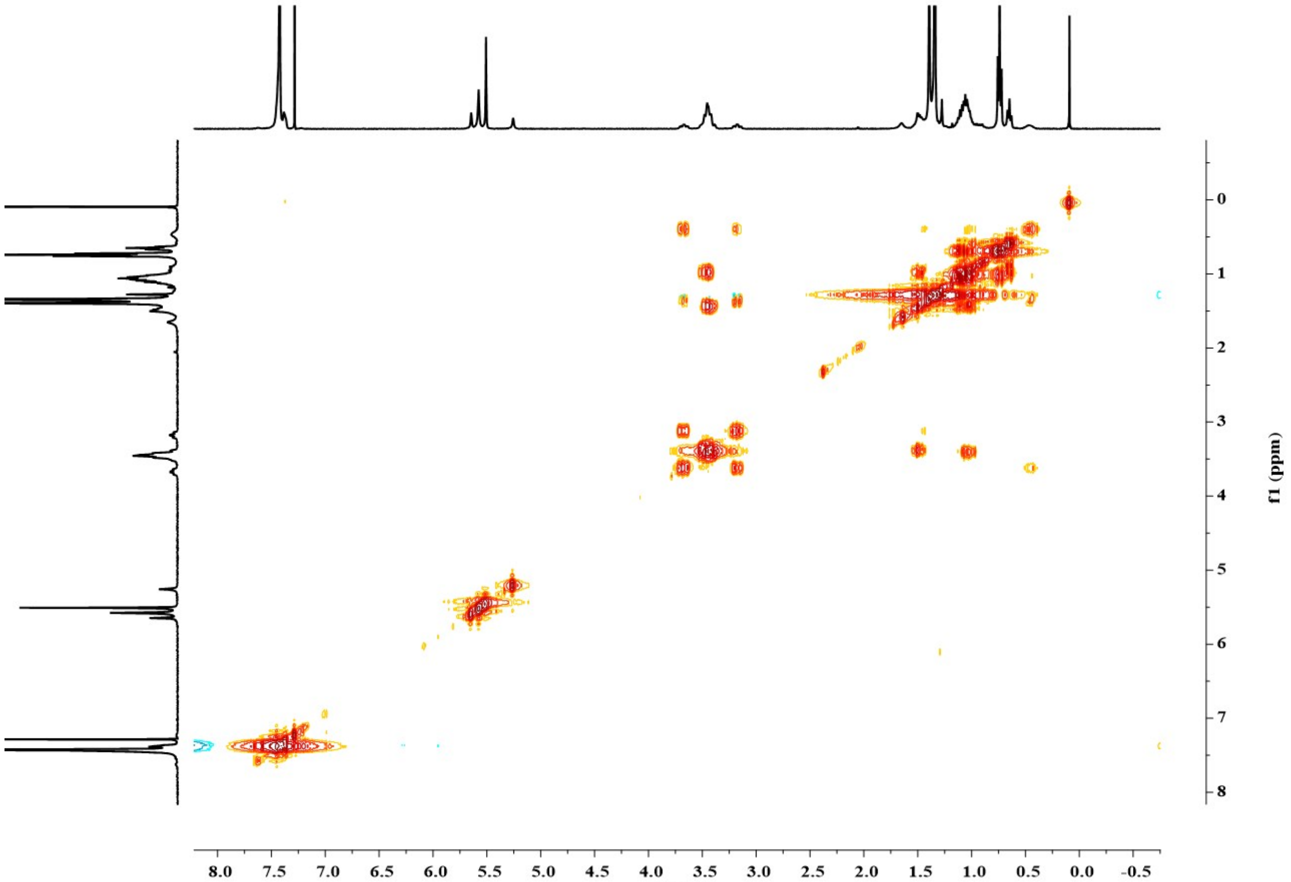

Supplementary Figure 32 | COSY experiment of the title compound recorded in $CDCl_3$.

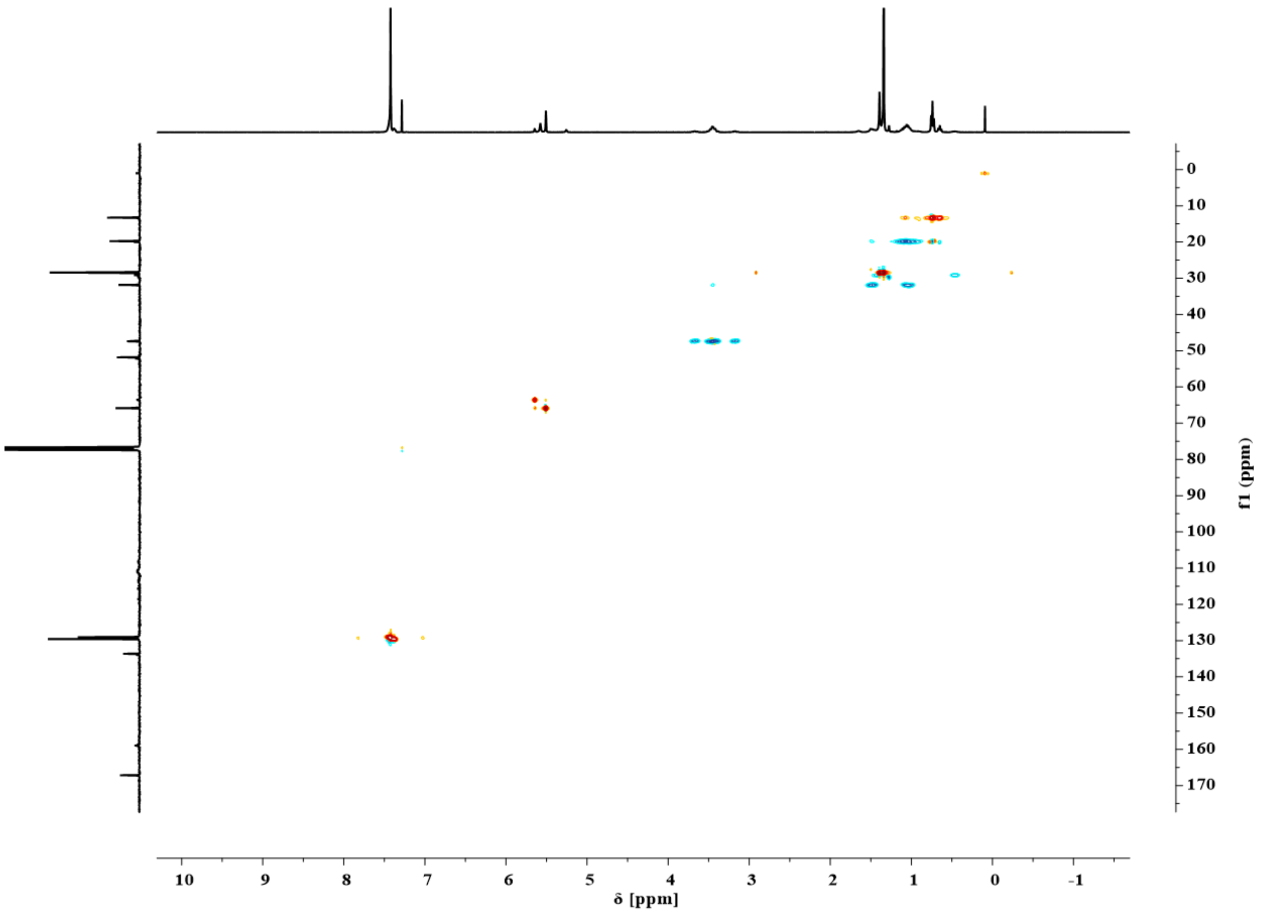

Supplementary Figure 33 | Multiplicity-edited HSQC experiment of the title compound recorded in $CDCl_3$.

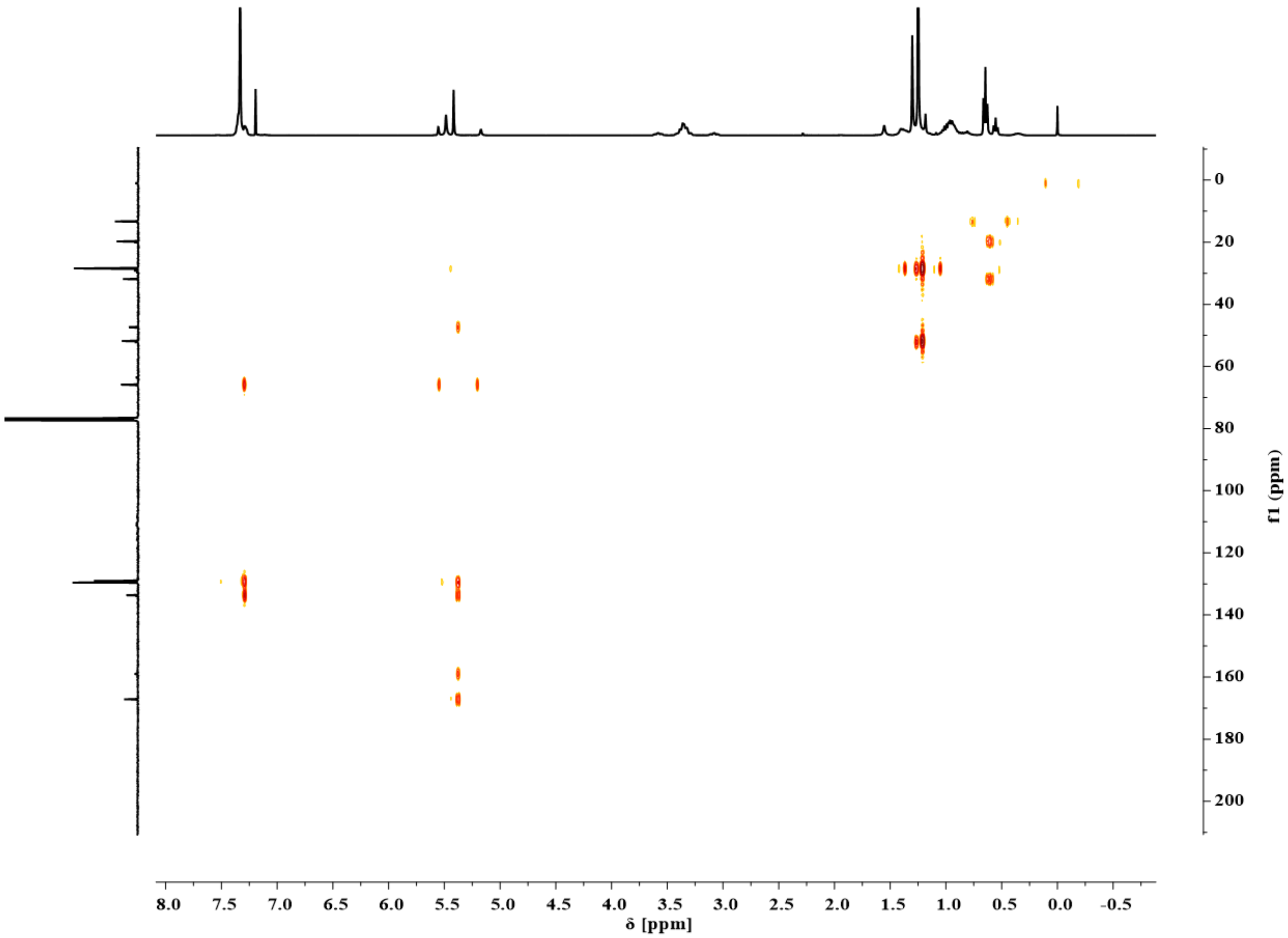

Supplementary Figure 34 | HMBC experiment of the title compound recorded in $CDCl_3$.

Ugi reaction of perfluoropentanoic acid benzaldehyde, *tert*-butylisocyanide and butylamine

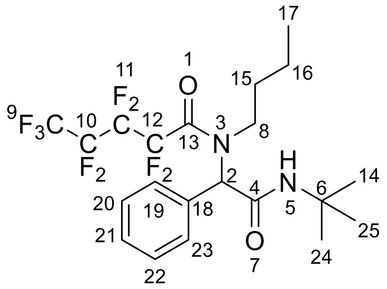

In a 25 mL round bottom flask benzaldehyde (115 µL, 119 mg, 1.12 mmol, 1.70 eq.) was dissolved in 1.5 mL methanol, subsequently butylamine (114 µL, 82.4 mg, 1.12 mmol, 1.70 eq.) was added and the resulting mixture was stirred for 60 min over sodium sulfate. Afterwards, the mixture was filtrated and the solid was washed with 10 mL methanol three times. Subsequently, the filtrate was concentrated under reduced pressure. Perfluoropentanoic acid (175 mg, 663 µmol, 1.00 eq.) dissolved in 1 mL methanol was added to the imine at room temperature and the resulting mixture was stirred for 2 min. Subsequently, *tert*-butylisocyanide (127 µL, 93.7 mg, 1.12 mmol, 1.70 eq.) was added to the stirring mixture. The reaction was stirred for 3 d at room temperature. The crude reaction mixture was dried under reduced pressure and purified *via* column chromatography employing Fluoro*Flash*® silica gel. The fluorous fraction was tested for purity *via* TLC and concentrated under reduced pressure. The remaining perfluoro acid was removed with a short silica gel filter column, eluting with *c*-hexane/ethyl acetate (3:1) to yield the Ugi product as a yellow powder (259 mg, 562 µmol, 85.1%).

R_f = 0.50 in *c*-hexane/ethyl acetate (5:1). Visualized *via* fluorescent quench and Seebach staining solution.

IR (ATR): ν [cm^{-1}] = 3317.9 (w, ν(N-H)), 2963.9 (w, ν(C-H)), 1679.7 (m, ν(C=O)), 1654.9 (s, ν(C=O)), 1556.6 (m), 1475.8 (w), 1454.4 (w), 1429.8 (m), 1355.4 (w), 1305.9 (w), 1233.5 (s), 1214.1 (s), 1187.4 (s), 1137.4 (s), 1125.2 (w), 1110.5 (w), 1029.5 (w), 959.2 (w), 869.7 (w), 855.6 (w), 805.5 (w), 787.1 (w), 764.8 (w), 748.8 (w), 728.1 (w), 699.2 (w), 648.3 (m), 634.3 (s), 610.4 (w), 522.6 (s), 498.2 (w), 435.9 (w).

^{1}H NMR (400 MHz, CD_3OD): δ [ppm] = 7.67 – 7.06 (m, 5 H, $CH_{Ar}^{19\text{-}23}$), 6.09 – 5.59 (m, 2 H, CH^2 + NH^5), 3.72 – 2.94 (m, 2 H, CH_2^{8}), 1.33 (d, J = 15.2 Hz, 9 H, $CH_3^{14,24,25}$), 1.03 – 0.76 (m, 4 H, CH_2^{15+16}), 0.68 – 0.55 (m, 3 H, CH_3^{17}).

^{13}C NMR (126 MHz, CD_3OD): δ [ppm] = 168.8 (s, $CONR^4$), 158.0 (s, $CONR^{13}$), 134.2 (s, C_{Ar}^{18}), 130.2 (s, CH_{Ar}), 129.6 (s, CH_{Ar}), 128.9 (s, CH_{Ar}), 128.8 (s, CH_{Ar}), 128.6 (s, CH_{Ar}), 64.2 (s, CH^2), 51.0

(s, C^6), 45.8 (s, CH_2^{8}), 32.1 (s, $CH_2^{15\ or\ 16}$), 29.0 (s, $CH_3^{14,\ 24,\ 25}$), 27.3 (s, $CH_3^{14,\ 24,\ 25}$), 19.5 (s, $CH_2^{15\ or\ 16}$), 12.3 (s, CH_3^{17}).

^{19}F NMR (376 MHz, CD_3OD): δ [ppm] = -82.77 (dt, J = 23.6, 11.7 Hz, 3 F, CF_3^{9}), AB-signal (δ_A = -110.63, δ_B = -111.89, J_{AB} = 240.9 Hz, A and B are split into t, J = 14.0 Hz, CF_2^{12a}), AB-signal (δ_A = -112.48, δ_B = -113.57, J_{AB} = 235.3 Hz, A and B are split into t, J = 14.4 Hz, CF_2^{12b}), -122.01 – -122.31 (m, CF_2), -124.75 (s, CF_2), -125.33 (s, CF_2), -125.44 (s, CF_2^{10}). Total integral of CF_2 region normalized with respect to the CF_3^{9} group = 6.

ESI-MS [*m/z*]: [M + Na]$^+$ calculated for $^{12}C_{21}{}^{1}H_{25}{}^{16}O_2{}^{14}N_2{}^{19}F_9{}^{23}Na$, 531.1665; found, 531.1669, Δ = 0.42 mmu.

ESI-MS [*m/z*]: [2 M + Na]$^+$ calculated for $^{12}C_{42}{}^{1}H_{50}{}^{16}O_4{}^{14}N_4{}^{19}F_{18}{}^{23}Na_2$, 1039.3437; found, 1039.3450, Δ = 1.29 mmu.

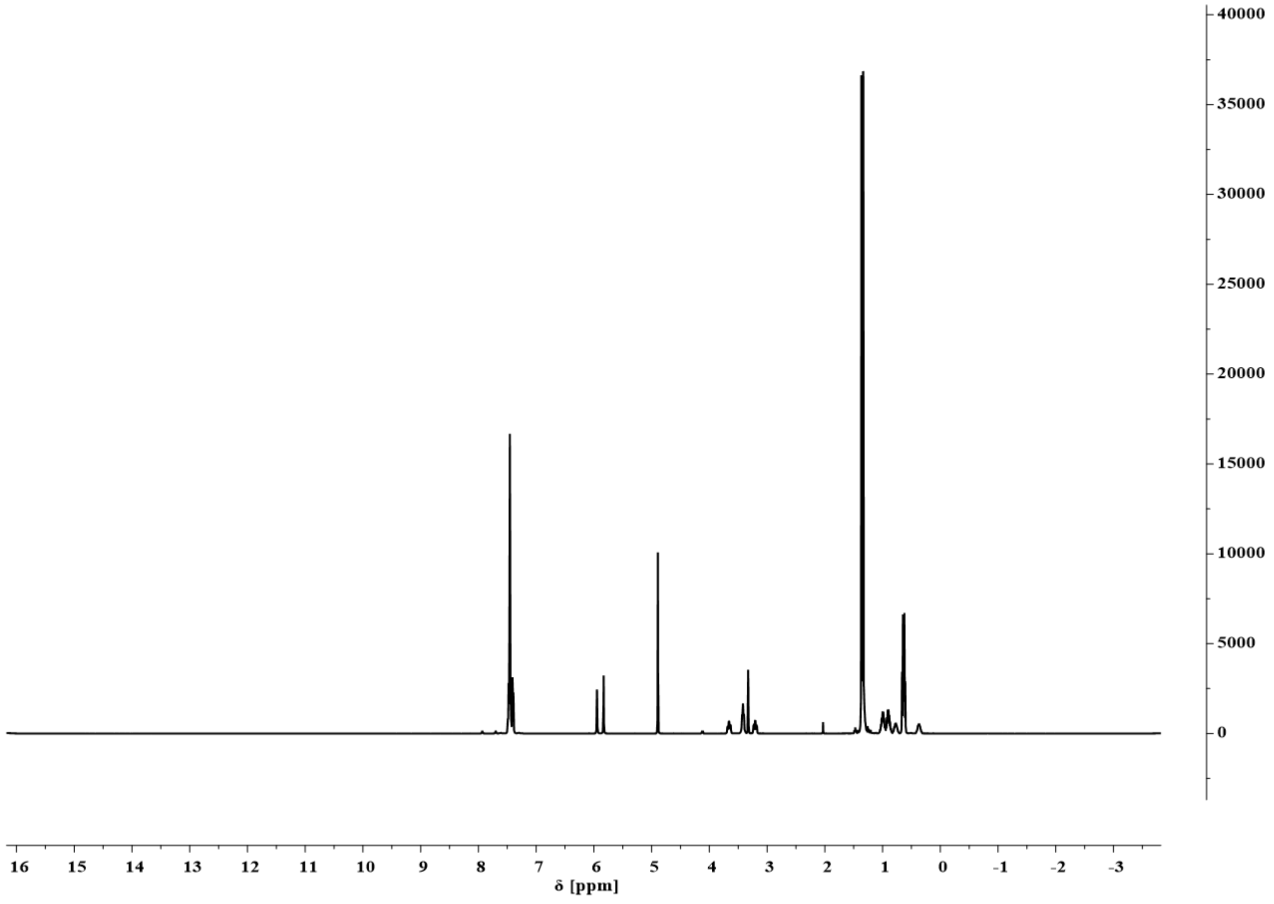

Supplementary Figure 35 | ^{1}H NMR of the title compound recorded in CD_3OD.

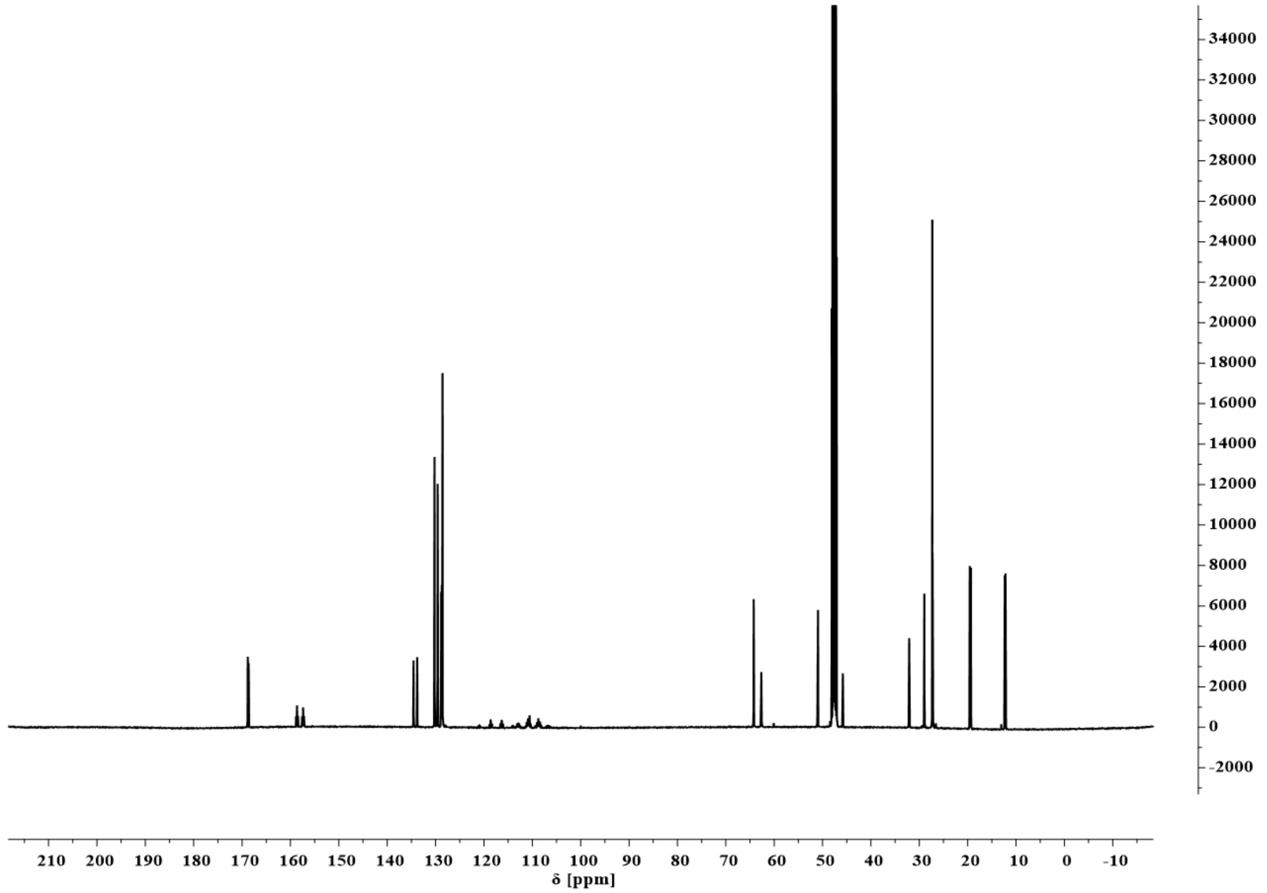

Supplementary Figure 36 | ^{13}C NMR of the title compound recorded in CD_3OD.

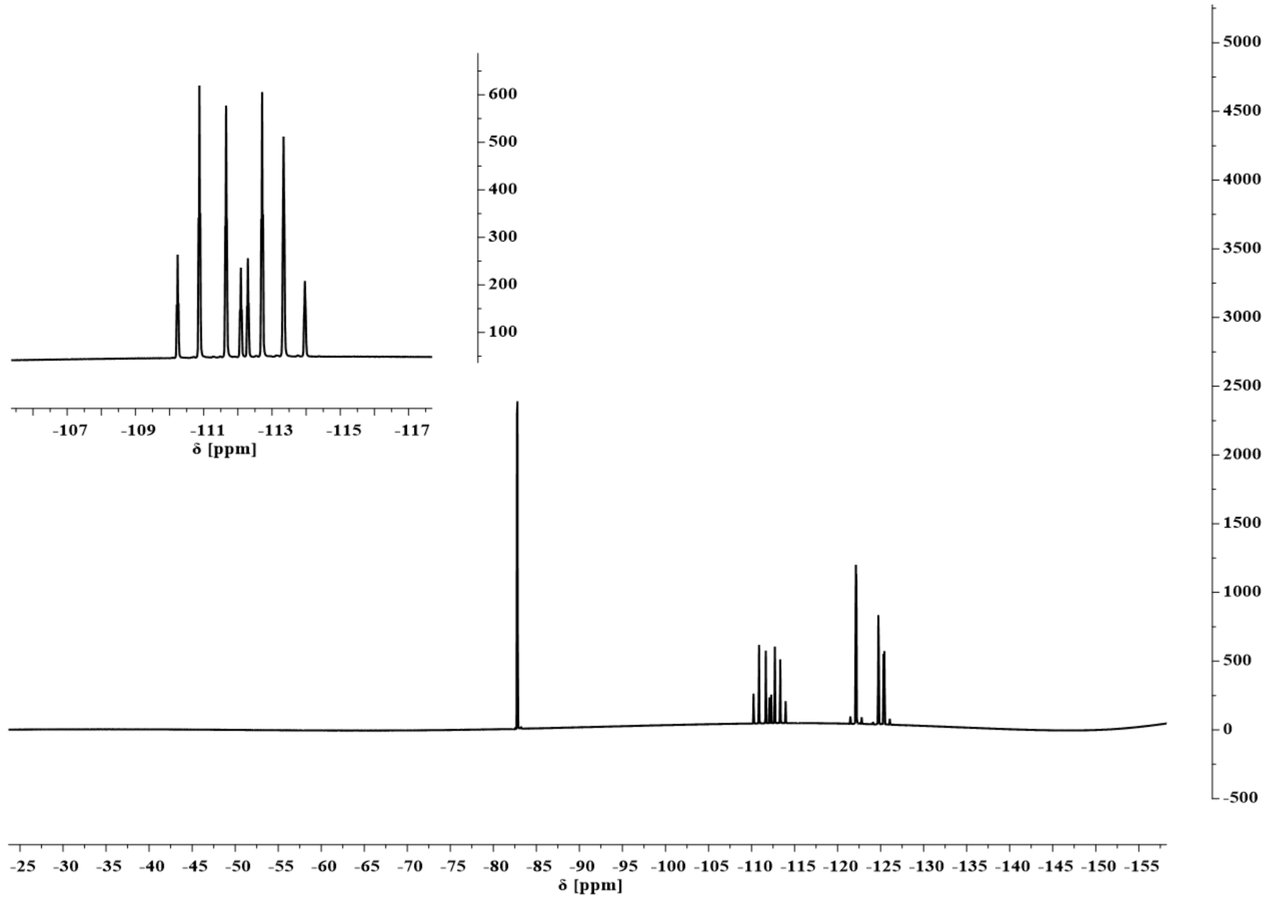

Supplementary Figure 37 | ^{19}F NMR of the title compound recorded in CD_3OD.

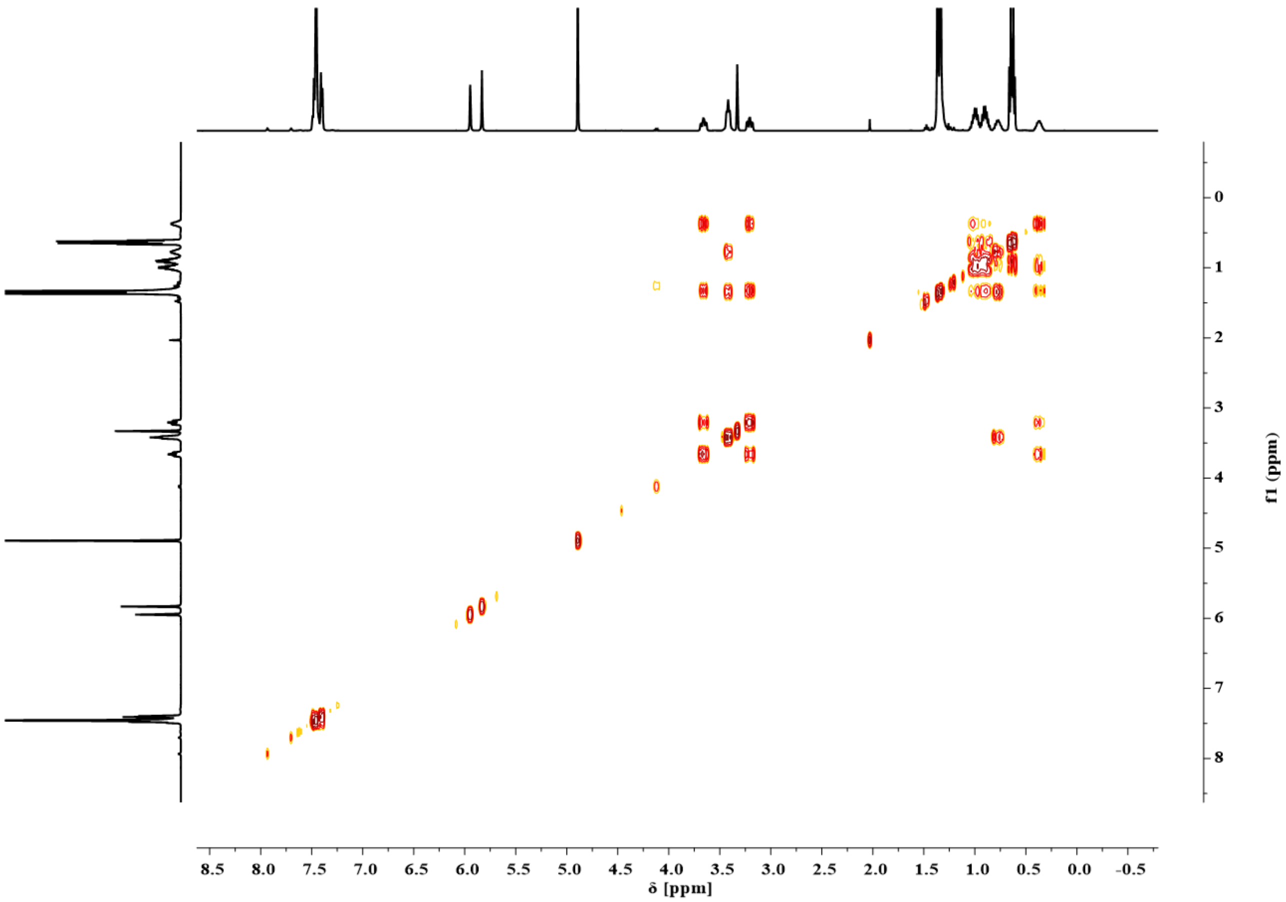

Supplementary Figure 38 | COSY experiment of the title compound recorded in CD_3OD.

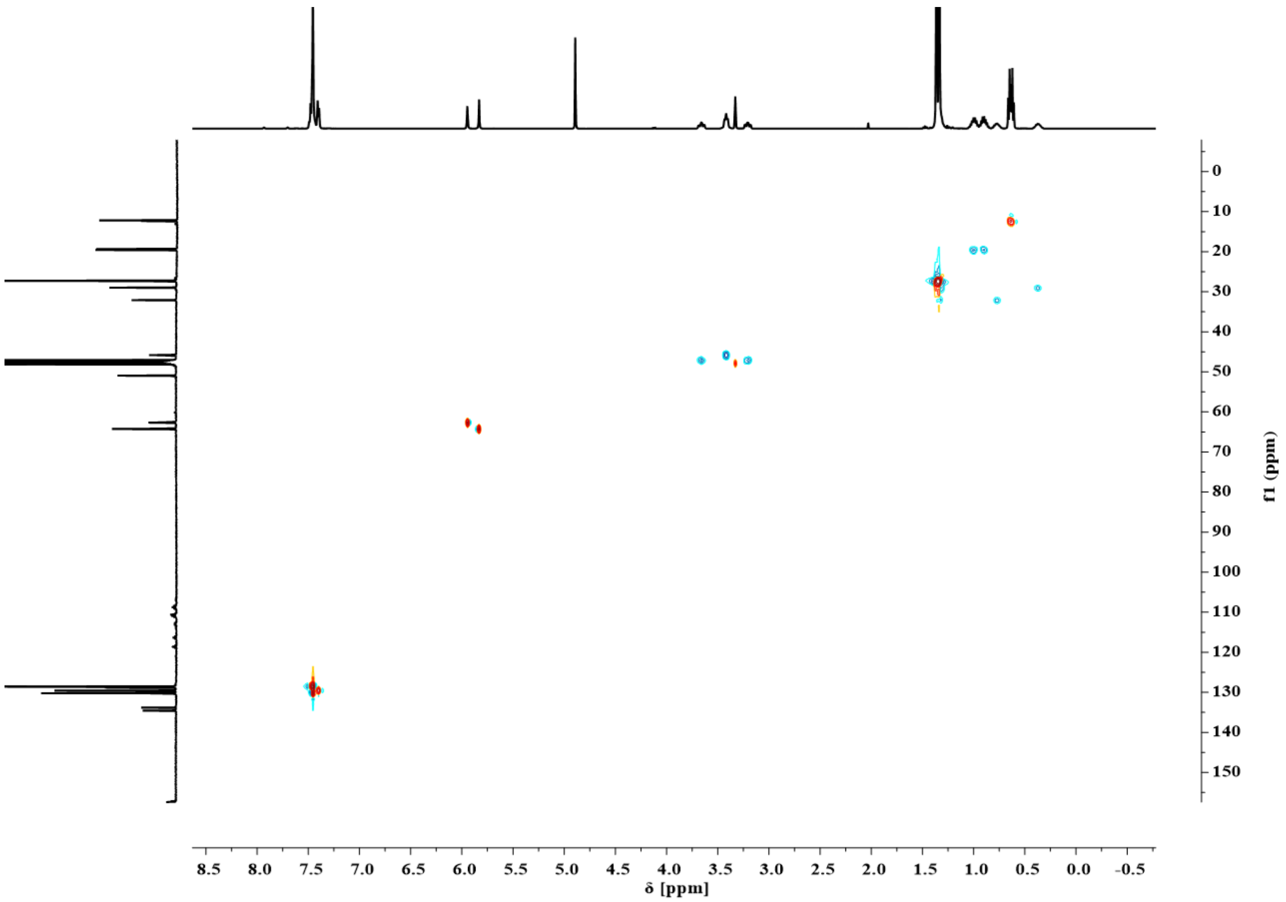

Supplementary Figure 39 | Multiplicity-edited HSQC experiment of the title compound recorded in $CDCl_3$.

Ugi reaction of perfluorononanoic acid, *p*-anisaldehyde, cyclohexylisocyanide and propargylamine

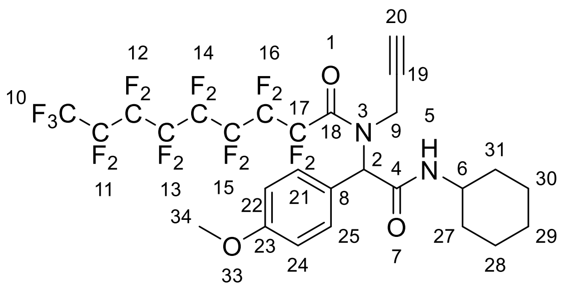

In a 25 mL round bottom flask *p*-anisaldehyde (77.9 µL, 87.3 mg, 641 µmol, 1.70 eq.) isobutyraldehyde and propargylamine (41.4 µL, 35.3 mg, 641 µmol, 1.70 eq.) were added. The resulting mixture was stirred for 60 min over sodium sulfate. Perfluorononanoic acid (175 mg, 377 µmol, 1.00 eq.) dissolved in 0.5 mL methanol was added to the imine at room temperature and the resulting mixture was stirred for 2 min. Subsequently, cyclohexylisocyanide (79.7 µL, 70.0 mg, 641 µmol, 1.70 eq.) was added to the stirring mixture. The reaction was stirred for 3 d at room temperature. The crude reaction mixture was dried under reduced pressure and purified *via* column chromatography employing Fluoro*Flash*® silica gel. The fluorous fraction was tested for purity *via* TLC and concentrated under reduced pressure. The remaining perfluorononanoic acid was removed with a short silica gel filter column, eluting with *c*-hexane/ethyl acetate (3:1) to yield the Ugi product as a yellow oil (158 mg, 212 µmol, 56.3%).

R_f = 0.36 in *c*-hexane/ethyl acetate (6:1). Visualized *via* fluorescent quench and Seebach staining solution.

IR (ATR): ν [cm^{-1}] = 3314.9 (m, ν(N-H)), 2937.2 (m, ν(C-H)), 2862.0 (w), 1687.5 (vs, ν(C=O)), 1655.8 (vs), 1612.7 (m), 1550.2 (s), 1518.1 (s), 1440.1 (s), 1405.5 (m), 1368.2 (m), 1326.9 (m), 1307.5 (m), 1286.6 (s), 1243.1 (s), 1021.5 (s), 1150.3 (s), 1119.4 (vs), 1079.4 (vs), 1034.4 (vs), 1011.4 (vs), 986.9 (s), 939.2 (m), 894.0 (m), 874.4 (m), 838.6 (m), 776.3 (m), 755.3 (m), 736.6 (m), 707.6 (m), 653.7 (vs), 642.8 (s), 629.0 (vs), 618.8 (vs), 560.0 (s), 527.0 (vs), 441.5 (w), 424.2 (m).

^{1}H NMR (500 MHz, CD_3OD): δ [ppm] = 7.41 – 7.19 (m, 2 H, $CH_{Ar}^{21,25}$), 7.03 – 6.81 (m, 2 H, $CH_{Ar}^{22,24}$), 5.91 (s, 1 H, CH^{2}), 4.40 – 4.18 (m, 2 H, CH_2^{9}), 3.80 (s, 3 H, CH_3^{34}), 3.69 (s, 1 H, CH^{6}), 3.34 – 3.25 (m, 1 H, CH^{20}), 1.94 – 1.10 (m, 10 H, CH_2).

^{13}C NMR (126 MHz, CD_3OD): δ [ppm] = 173.0 (s, $CONR^{4}$), 170.0 (s, $CONR^{18}$), 162.0 (s, C_{Ar}^{23}), 133.1 (s, $CH_{Ar}^{21,25}$), 132.1 (s, C_{Ar}^{8}), 115.3 (s, $CH_{Ar}^{22,24}$), 61.5 (s, CH^{2}), 55.8 (s, CH_3^{34}), 50.0 (s, CH^{6} or CH^{20}), 50.0 (s, CH^{6} or CH^{20}), 36.9 (s, CH_2^{9}), 33.5 (s, CH_2), 33.5 (s, CH_2), 27.0 (s, CH_2), 26.6 (s, CH_2), 26.0 (s, CH_2).

^{19}F NMR (376 MHz, CD_3OD): δ [ppm] = -88.24 (t, J = 10.3 Hz, 3 F, CF_3[10]), AB-signal (δ_A = -116.45, δ_B = -117.92, J_{AB} = 301.2 Hz, A and B are split into t, additional coupling not resolved, signals broadened, CF_2[17a]), AB-signal (δ_A = -117.79, δ_B = -119.06, J_{AB} = 293.6 Hz, A and B are split into t, additional coupling not resolved, signals broadened, CF_2[17b]), -126.39 – -127.61 (m, CF_2), -128.66 (s, CF_2), -129.62 (s, CF_2), -133.16 (s, CF_2[11]). Total integral of CF_2 region normalized with respect to the CF_3[10] group = 14.

FAB – MS [*m/z*] (relative intensity): 747.2 (25%) $[M-H]^+$, 621.0 (30%) $[\text{Fragment A} + H]^+$, 620.0 (45%) $[\text{Fragment A}]^+$, 582.0 (34%) $[\text{Fragment A} + H - C_3H_3]^+$, 247.1 (33%) $[\text{Fragment B} + H]^+$.

HRMS – FAB [*m/z*]: $[M + H]^+$ calculated for $^{12}C_{27}{}^{1}H_{24}{}^{16}O_{3}{}^{14}N_{2}{}^{19}F_{17}$, 747.1510; found, 747.1509; Δ = 0.06 mmu.

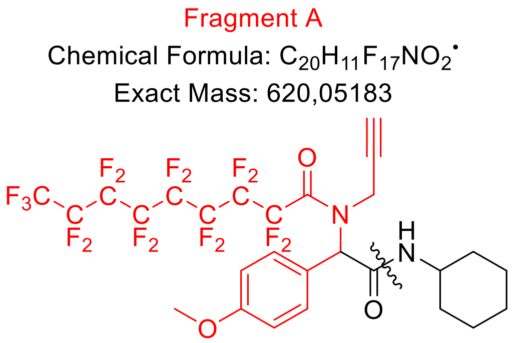

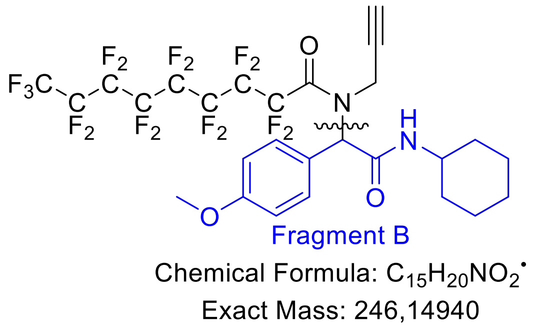

Supplementary Figure 40 | Proposed fragments observed in FAB-MS.

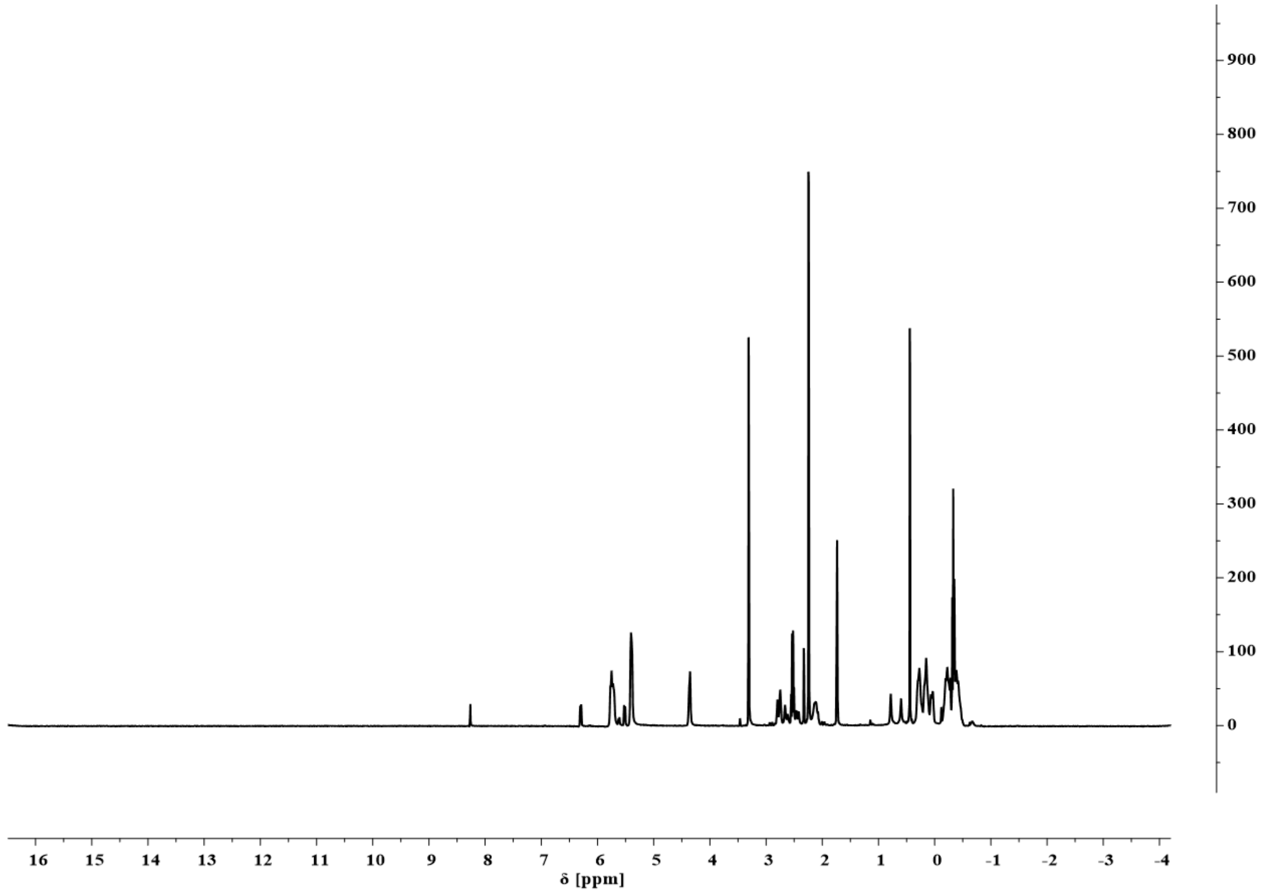

Supplementary Figure 41 | ^{1}H NMR of the title compound recorded in CD_3OD.

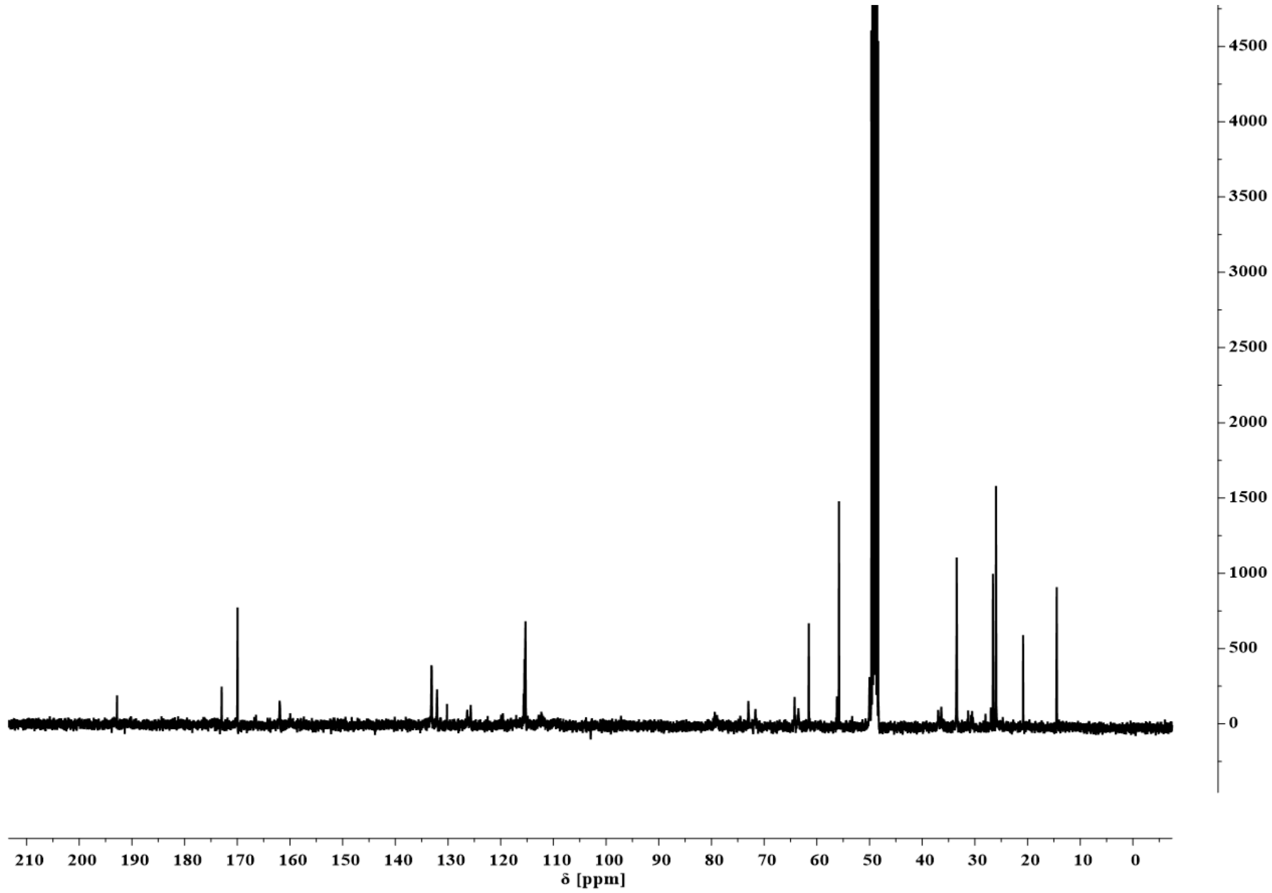

Supplementary Figure 42 | ^{13}C NMR of the title compound recorded in CD_3OD.

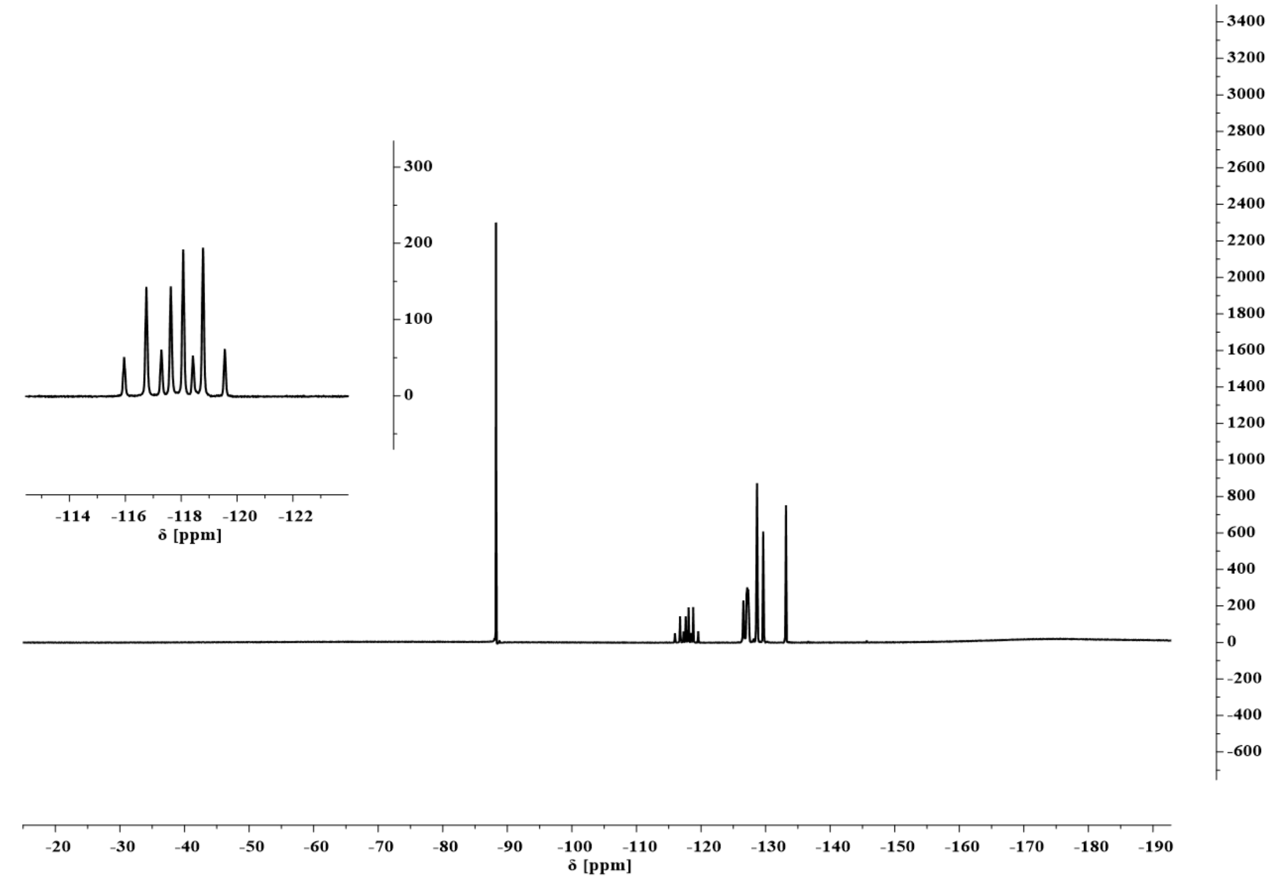

Supplementary Figure 43 | ^{19}F NMR of the title compound recorded in CD_3OD.

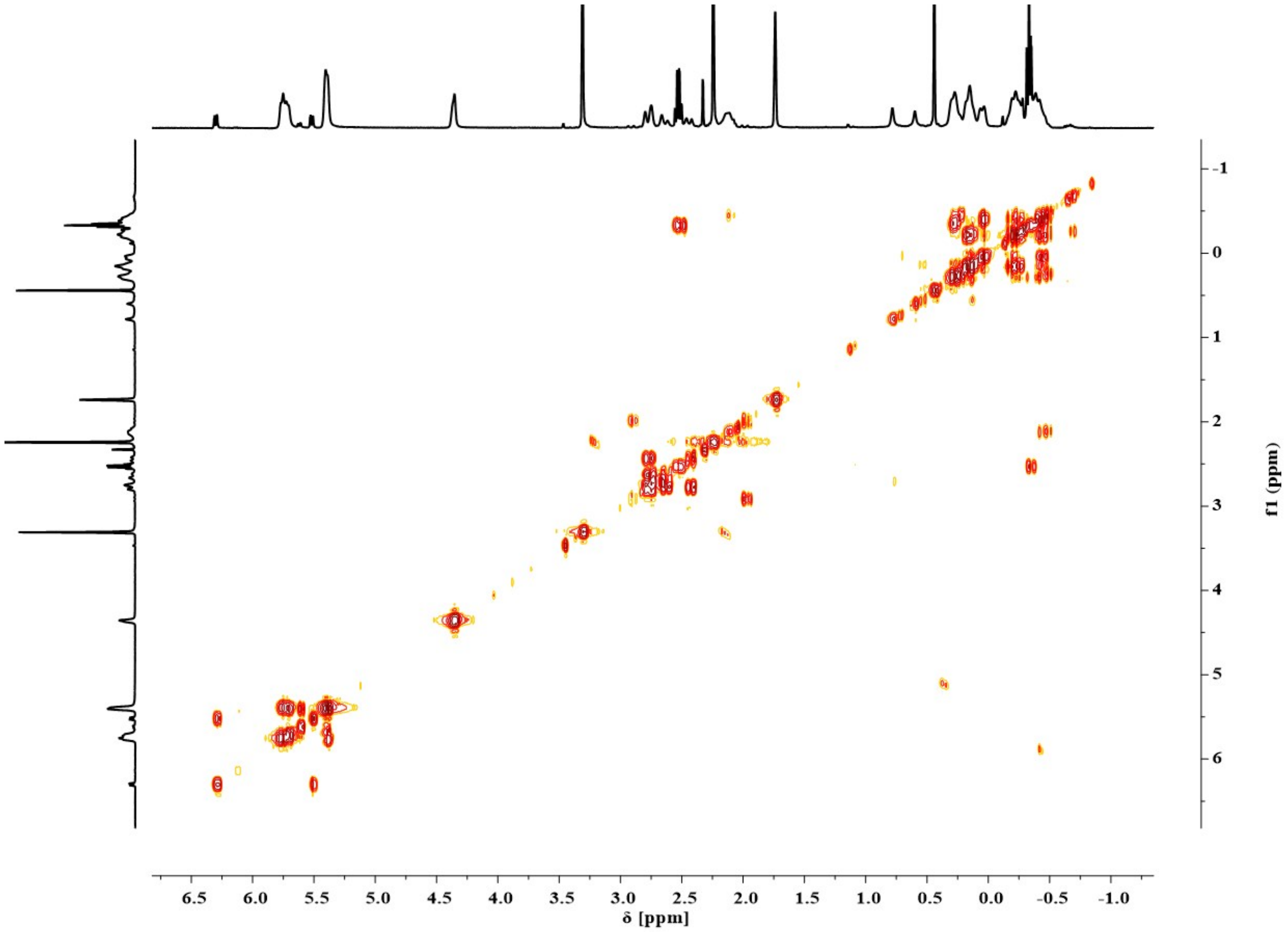

Supplementary Figure 44 | COSY experiment of the title compound recorded in CD_3OD.

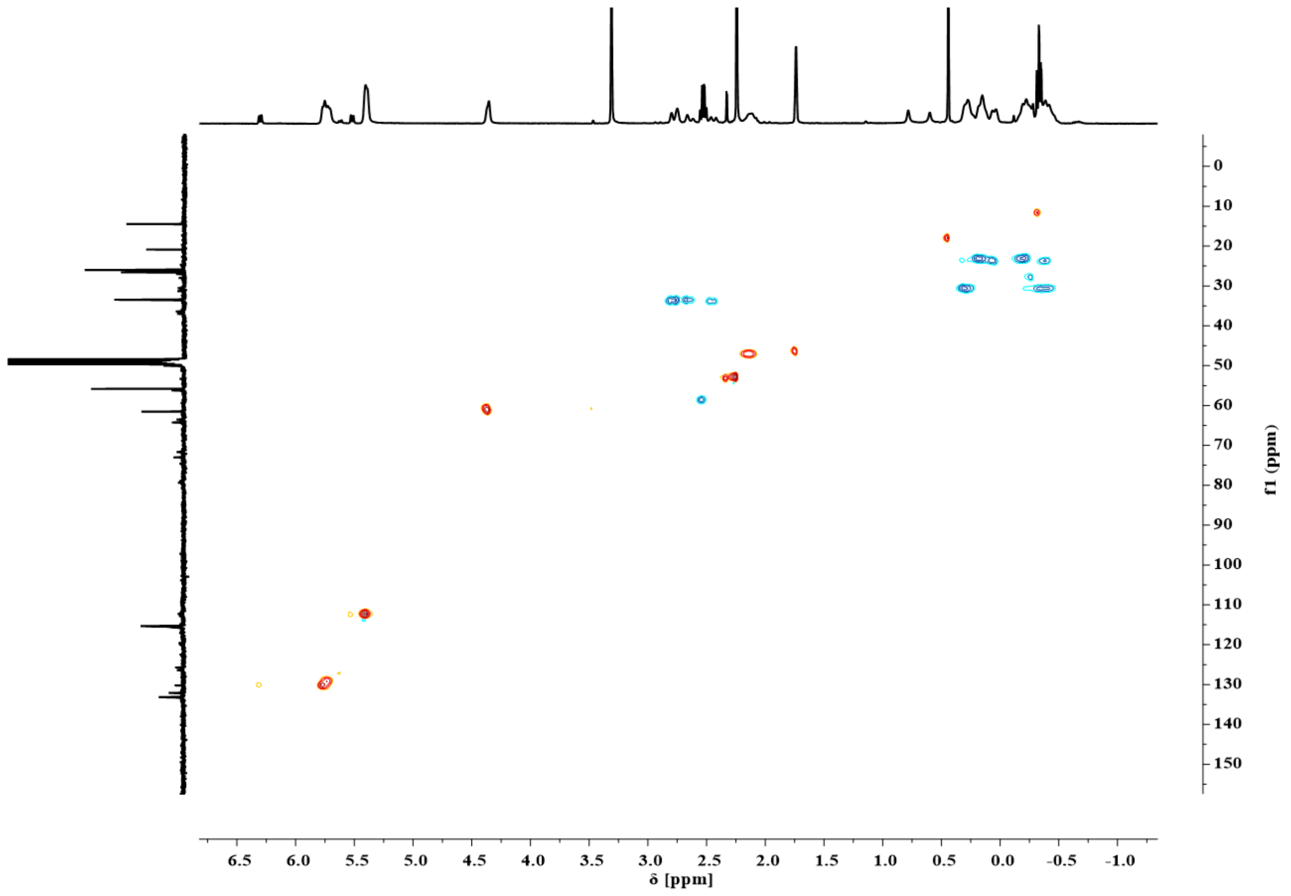

Supplementary Figure 45 | Multiplicity-edited HSQC experiment of the title compound recorded in $CDCl_3$.

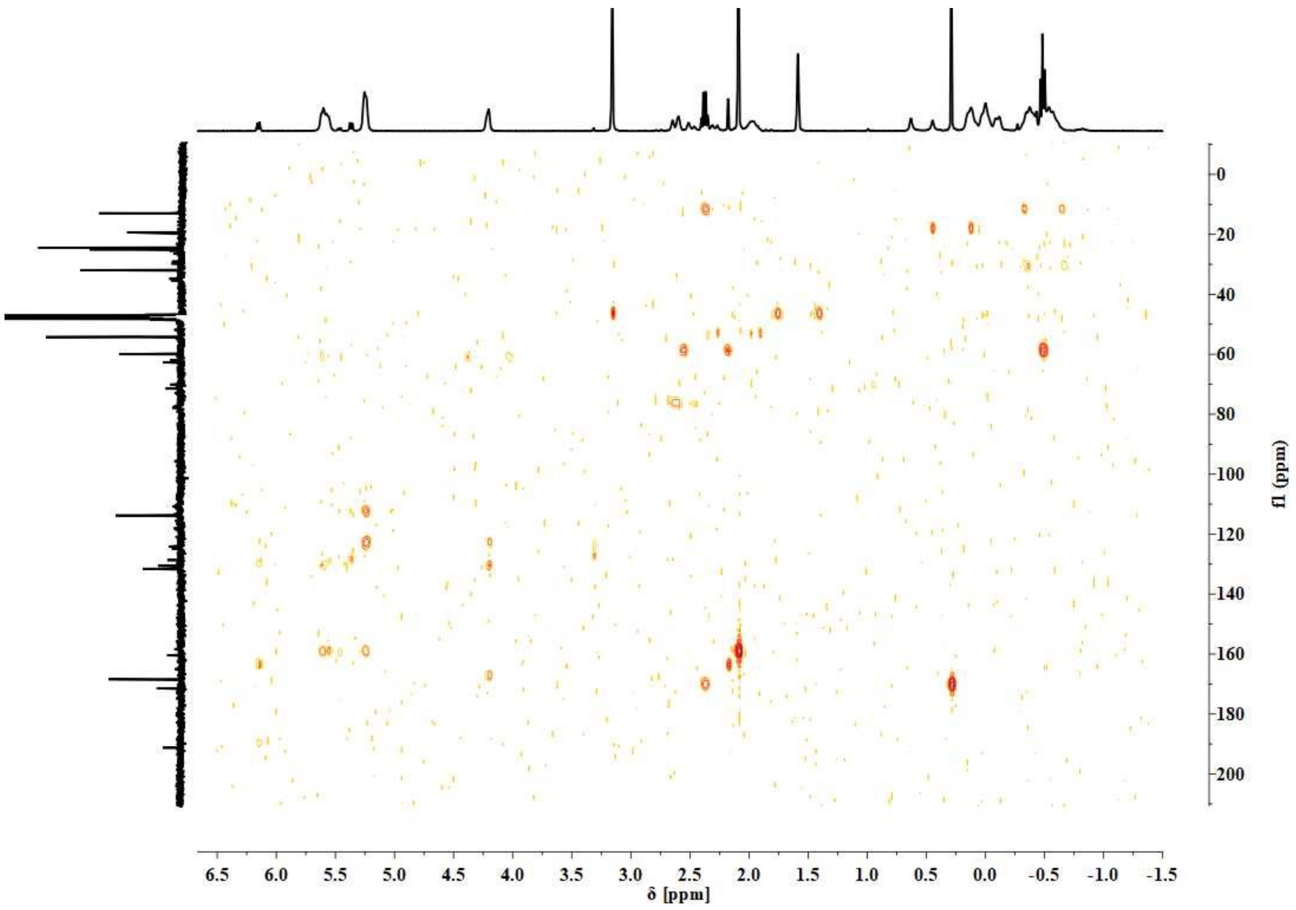

Supplementary Figure 46 | HMBC experiment of the title compound recorded in $CDCl_3$.

Ugi reaction of perfluorononanoic acid, *p*-anisaldehyde, cyclohexylisocyanide and allylamine

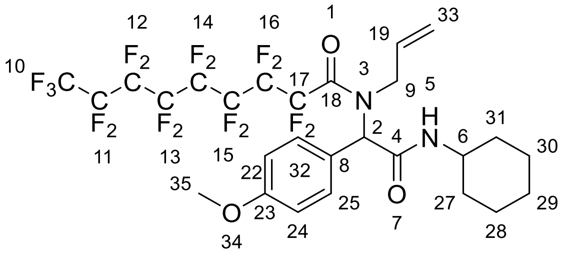

In a 25 mL round bottom flask *p*-anisaldehyde (77.9 µL, 87.3 mg, 641 µmol, 1.70 eq.) and allylamine (48.1 µL, 36.6 mg, 641 µmol, 1.70 eq.) were added. The resulting mixture was stirred for 60 min over sodium sulfate. Perfluorononanoic acid (175 mg, 377 µmol, 1.00 eq.) dissolved in 0.5 mL methanol was added to the solution at room temperature and the resulting mixture was stirred for 2 min. Subsequently, cyclohexylisocyanide (79.7 µL, 70.0 mg, 641 µmol, 1.70 eq.) was added to the stirring mixture. The reaction was stirred for 3 d at room temperature. The crude reaction mixture was dried under reduced pressure and purified *via* column chromatography employing Fluoro*Flash*® silica gel. The fluorous fraction was tested for purity *via* TLC and concentrated under reduced pressure. The remaining perfluorononanoic acid was removed with a short silica gel filter column, eluting with *c*-hexane/ethyl acetate (3:1) to yield the Ugi product as a yellow oil (193 mg, 259 µmol, 68.9%).

R_f = 0.43 in *c*-hexane/ethyl acetate (6:1). Visualized *via* fluorescent quench and Seebach staining solution.

IR (ATR): ν [cm^{-1}] = 3286.2 (br, ν(N-H)), 3083.6 (w), 2926.3 (m, ν(C-H)), 2849.5 (m), 1675.0 (s, ν(C=O)), 1655.9 (s), 1616.8 (m), 1555.6 (s), 1515.9 (s), 1417.2 (m), 1369.6 (m), 1330.1 (w), 1308.9 (w), 1248.4 (w), 1195.6 (s), 1143.4 (vs), 1116.4 (vs), 1042.2 (s), 989.9 (m), 943.3 (m), 927.1 (m), 889.1 (m), 863.4 (m), 840.1 (m), 805.7 (m), 760.8 (m), 716.6 (m), 681.4 (m), 649.5 (m), 633.9 (m), 615.3 (m), 564.7 (m), 549.9 (s), 519.5 (s), 481.4 (s), 450.5 (w).

^{1}H NMR (400 MHz, CD_3OD): δ [ppm] = 7.30 – 7.22 (m, 2 H, $CH_{Ar}^{25,32}$), 6.97 – 6.89 (m, 2 H, $CH_{Ar}^{22,24}$), 5.90 (s, 1 H, CH^{2}), 5.85 – 5.75 (m, 2 H, CH_2^{33}), 5.45 – 5.04 (m, 1 H, CH^{19}), 4.81 – 4.58 (m, 2 H, CH_2^{9}), 3.79 (s, 3 H, CH_3^{35}), 3.77 – 3.60 (m, 1 H, CH^{6}), 1.95 – 1.01 (m, 10 H, CH_2).

^{13}C NMR (126 MHz, CD_3OD): δ [ppm] = 173.0 (s, $CONR^{4}$), 170.2 (s, C_{Ar}^{23}), 162.0 (s, $CONR^{18}$), 135.2 (s C_{Ar}^{8}), 133.2 (s, CH^{19}), 132.3 (s, $CH_{Ar}^{25,32}$), 116.6 (s, CH_2^{9}), 115.2 (s, $C_{Ar}^{22,24}$), 65.0 (s, CH^{2}), 61.5 (s, CH_2^{33}), 55.8 (s, CH_3^{35}), 50.1 (s, CH^{6}), 33.6 (s, CH_2), 33.5 (s, CH_2), 26.6 (s, CH_2), 26.0 (s, CH_2), 20.9 (s, CH_2).

^{19}F NMR (376 MHz, CD_3OD): δ [ppm] = -88.26 (t, J = 10.5 Hz, 3 F, CF_3^{10}), AB-signal (δ_A = -116.14, δ_B = -117.58, J_{AB} =299.3 Hz, J_{AB} = 297.4 Hz, A and B are split into t, J = 12.3 Hz, CF_2^{17a}), AB-signal

(δ_A = 117.23, δ_B = -118.91, J_{AB} = 295.5 Hz, A and B are split into t, J = 12.6 Hz, CF_2[17b]), -126.44 (s, CF_2), -126.96 – -127.37 (m, CF_2), -128.69 (s, CF_2), -129.65 (s, CF_2), -133.16(s, CF_2[11]). Total integral of CF_2 region normalized with respect to the CF_3[10] group = 14.

FAB – MS [*m/z*] (relative intensity): 749.1 (25%) $[M + H]^+$, 622.0 (68%) [Fragment A $]^+$, 582.0 (52%) [Fragment A + H – $C_3H_5]^+$, 247.1 (28%) [Fragment B + H$]^+$.

HRMS – FAB [*m/z*]: $[M + H]^+$ calculated for $^{12}C_{27}{}^{1}H_{26}{}^{16}O_3{}^{14}N_2{}^{19}F_{17}$, 749.1665; found, 749.1666; Δ = 0.18 mmu.

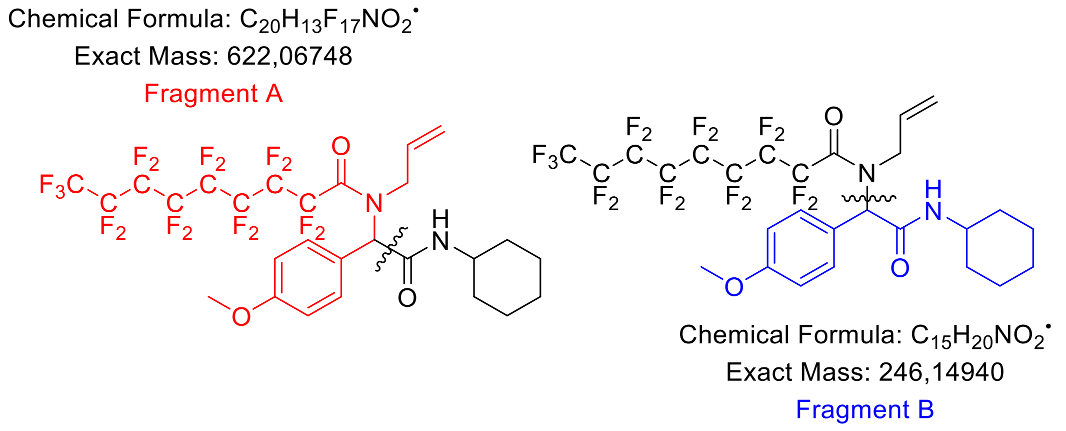

Supplementary Figure 47 | Proposed fragments observed in FAB-MS.

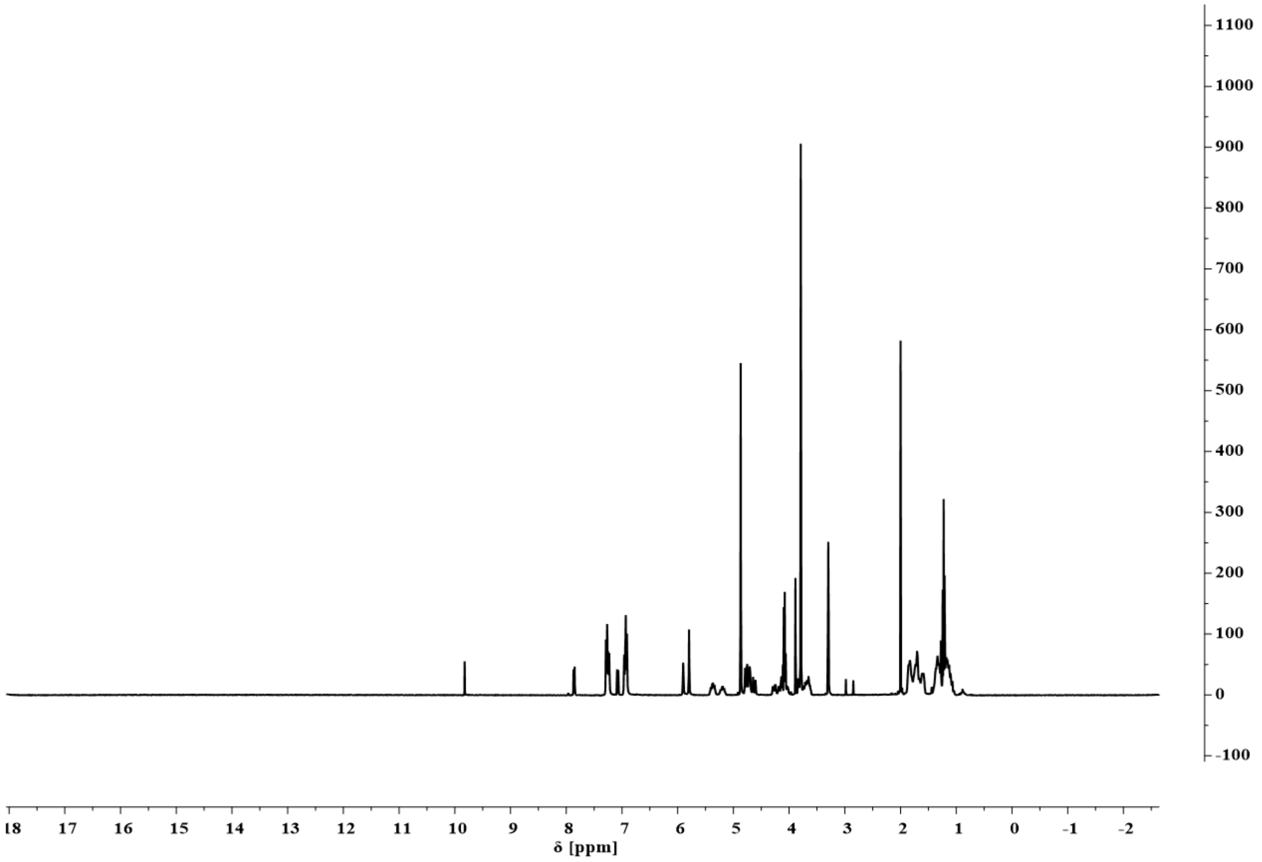

Supplementary Figure 48 | ^{1}H NMR of the title compound recorded in CD_3OD.

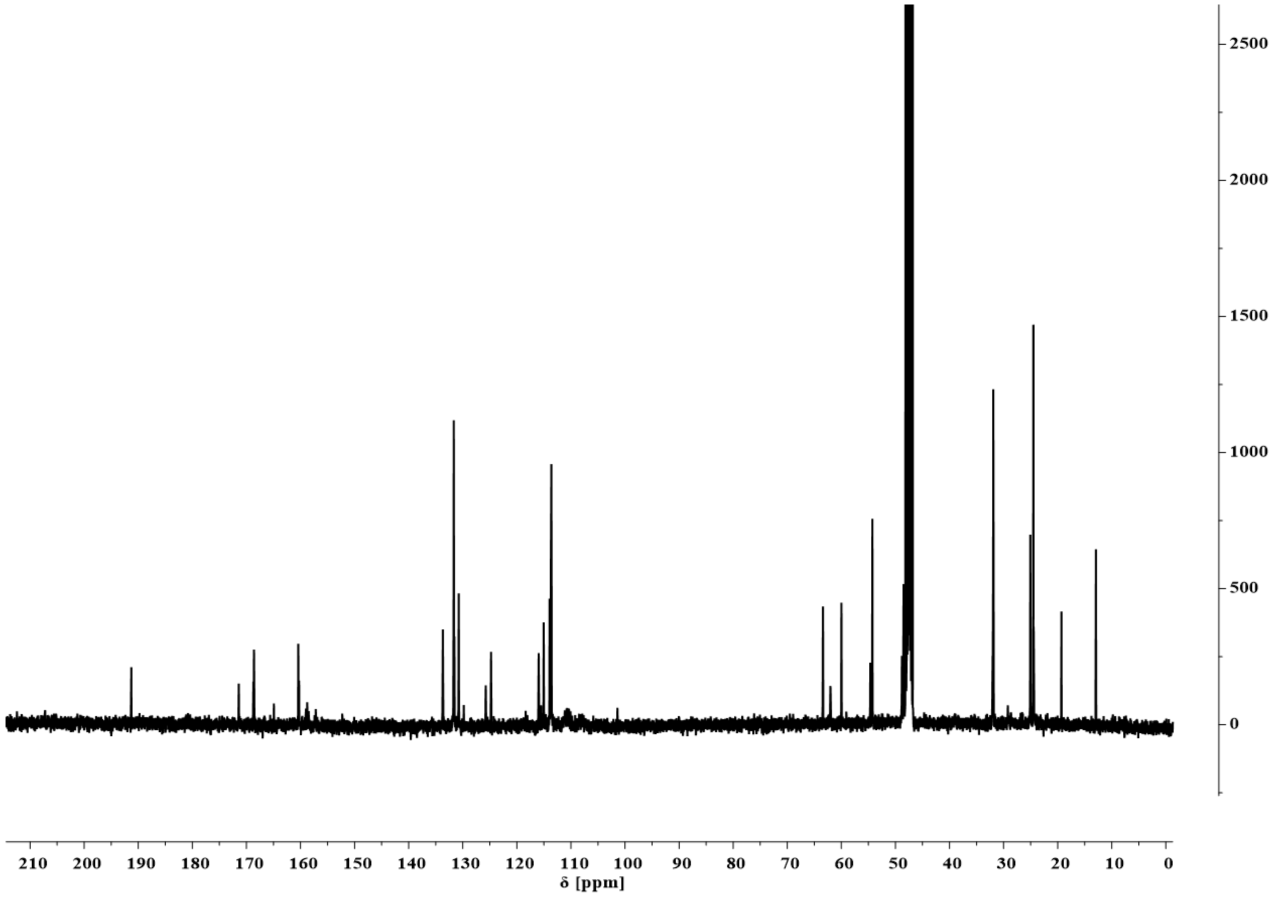

Supplementary Figure 49 | ^{13}C NMR of the title compound recorded in CD_3OD.

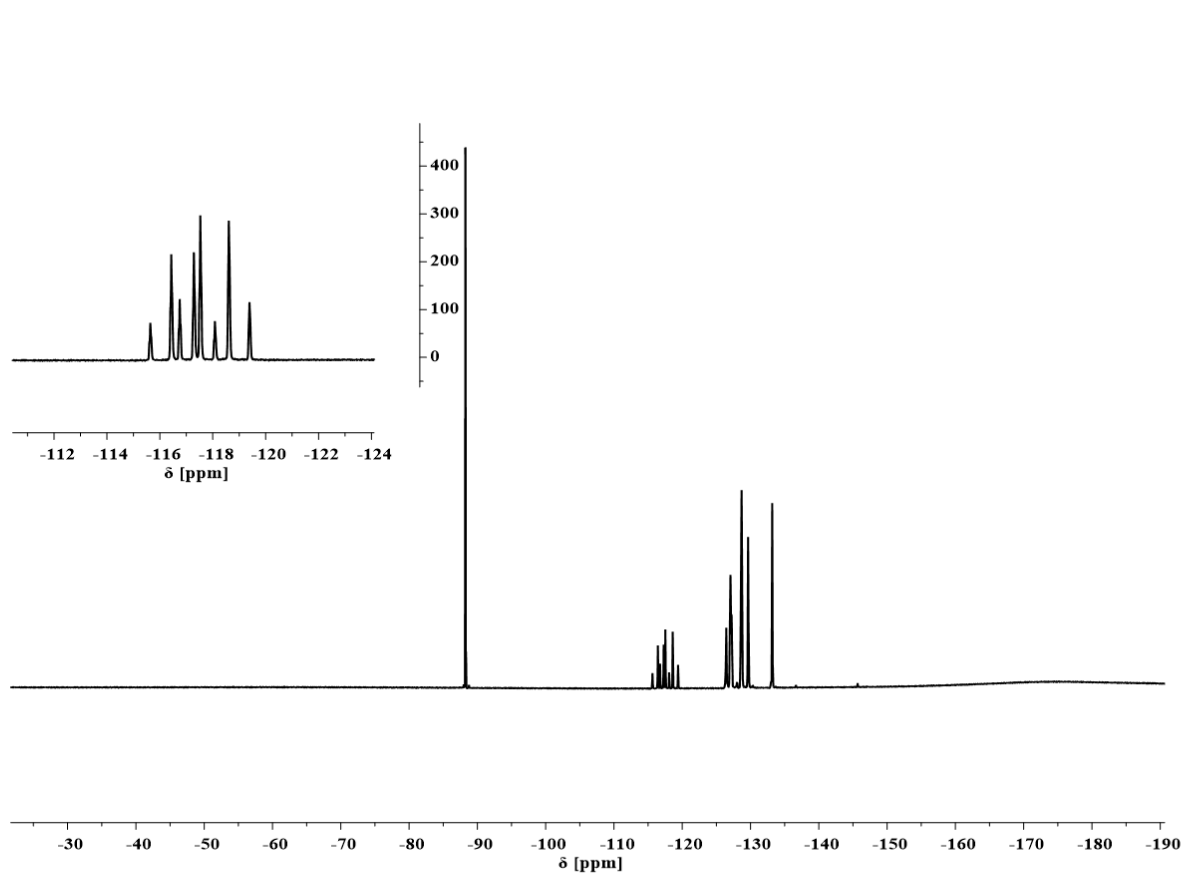

Supplementary Figure 50 | ^{19}F NMR of the title compound recorded in CD_3OD.

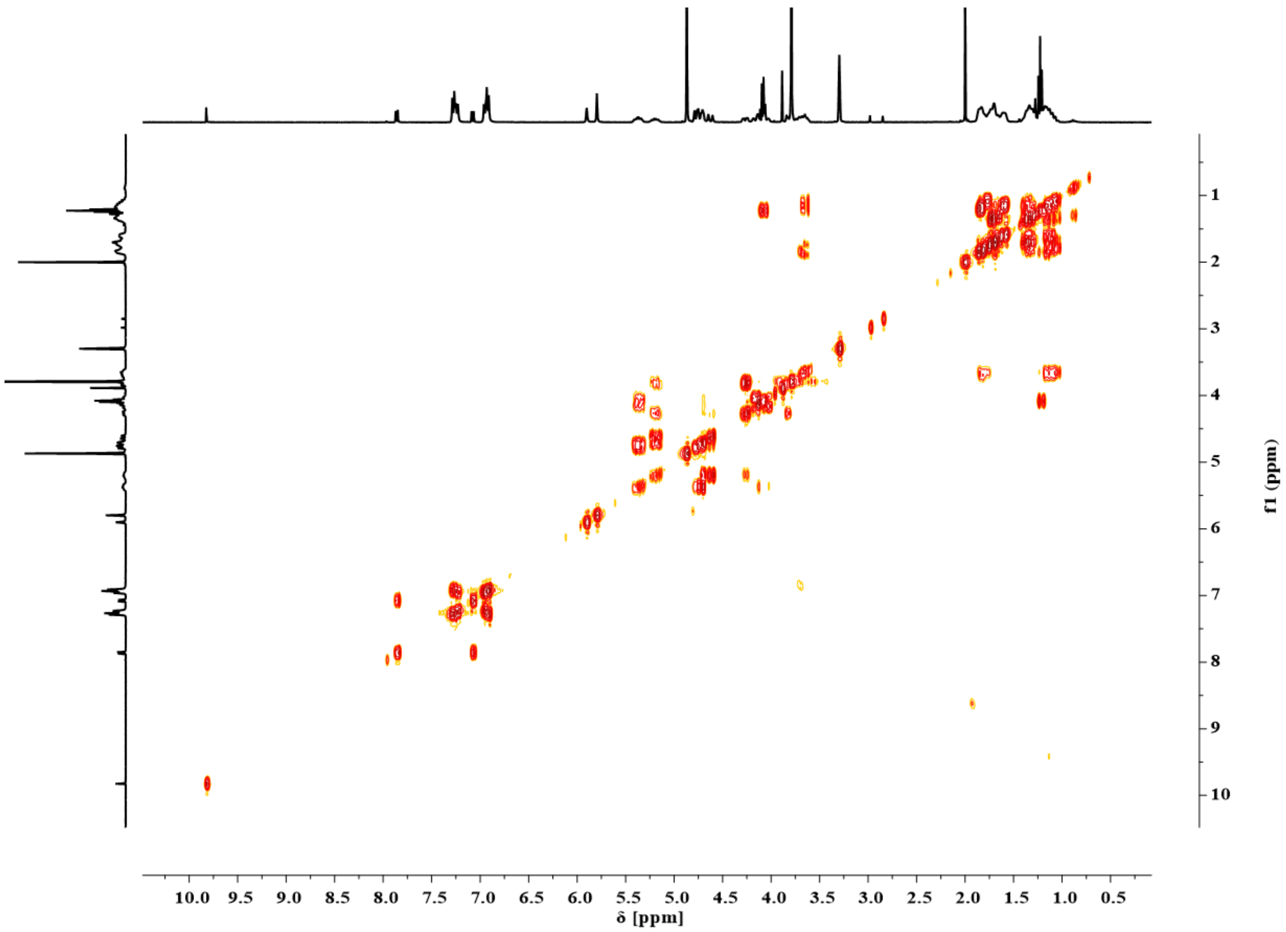

Supplementary Figure 51 | COSY experiment of the title compound recorded in CD_3OD.

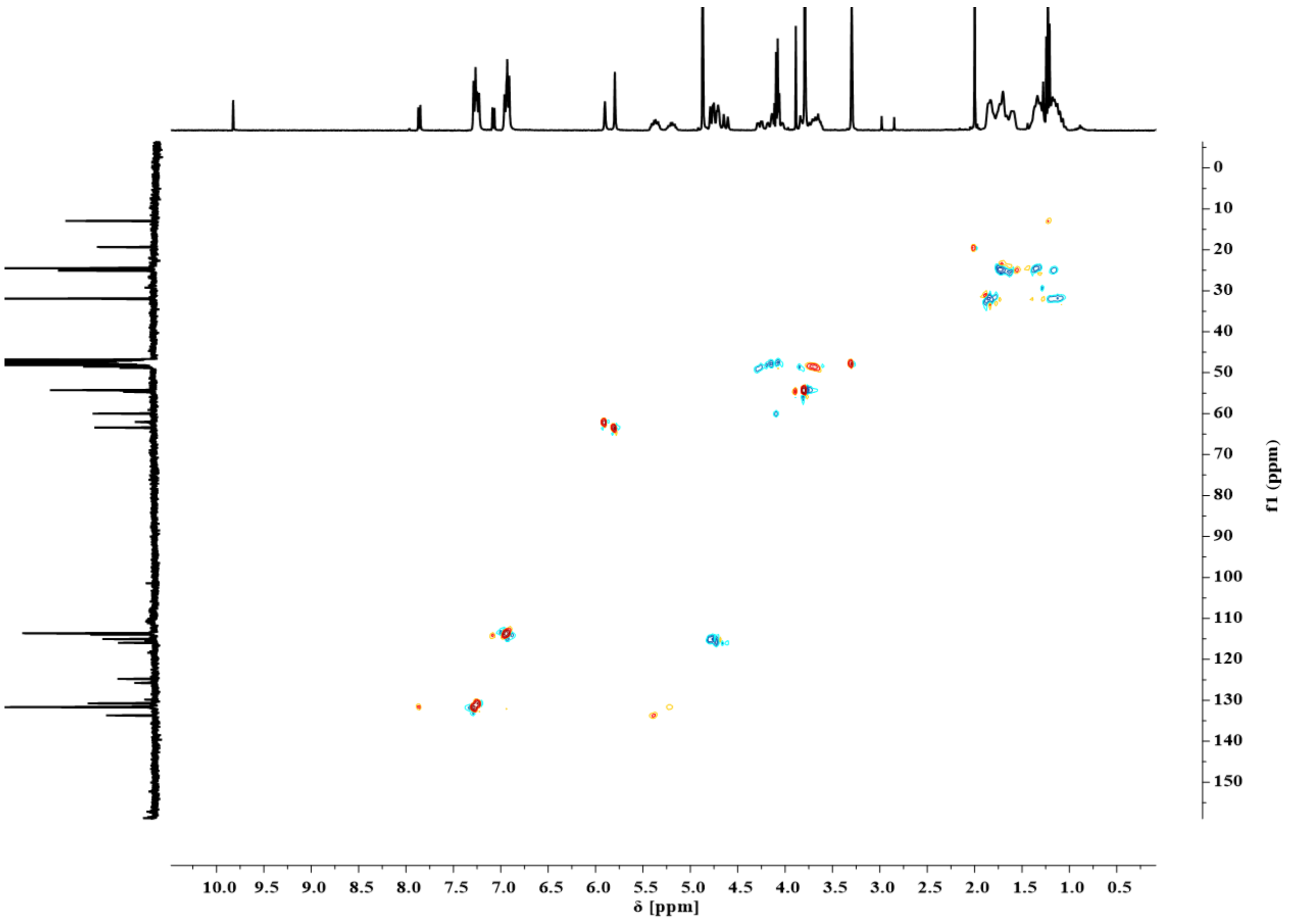

Supplementary Figure 52 | Multiplicity-edited HSQC experiment of the title compound recorded in $CDCl_3$.

Ugi reaction of perfluorononanoic acid, benzaldehyde, cyclohexylisocyanide and propargylamine

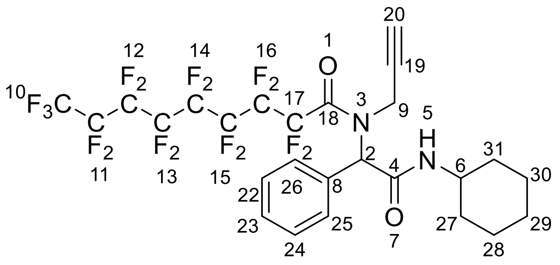

In a 25 mL round bottom flask benzaldehyde (65.4 µL, 68.0 mg, 641 µmol, 1.70 eq.) and propargylamine (41.4 µL, 35.3 mg, 641 µmol, 1.70 eq.) were added. The resulting mixture was stirred for 60 min over sodium sulfate. Perfluorononanoic acid (175 mg, 377 µmol, 1.00 eq.) dissolved in 0.5 mL methanol was added to the solution at room temperature and the resulting mixture was stirred for 2 min. Subsequently, cyclohexylisocyanide (79.7 µL, 70.0 mg, 641 µmol, 1.70 eq.) was added to the stirring mixture. The reaction was stirred for 3 d at room temperature. The crude reaction mixture was dried under reduced pressure and purified *via* column chromatography employing Fluoro*Flash*® silica gel. The fluorous fraction was tested for purity *via* TLC and concentrated under reduced pressure. The remaining perfluorononanoic acid was removed with a short silica gel filter column, eluting with *c*-hexane/ethyl acetate (3:1) to yield the Ugi product as a yellow oil (41.3 mg, 57.8 µmol, 15.3%).

R_f = 0.43 in *c*-hexane/ethyl acetate (6:1). Visualized *via* fluorescent quench and Seebach staining solution.

IR (ATR): ν [cm^{-1}] = 3292.6 (m, ν(N-H)), 2937.3 (m, ν(C-H)), 2857.2 (w, ν(C-H)), 1681.4 (s, ν(C=O)), 1645.5 (m, ν(N-H)), 1549.9 (m), 1494.1 (w), 1456.2 (m), 1421.6 (m), 1365.9 (m), 1329.4 (m), 1202.6 (vs), 1147.9 (vs), 1081.6 (m), 1029.4 (s), 1002.3 (m), 940.7 (m), 926.3 (m), 895.4 (w), 870.3 (w), 805.7 (m), 789.9 (m), 769.1 (s), 744.5 (s), 699.2 (vs), 669.8 (s), 633.2 (s), 559.7 (s), 520.3 (vs), 464.3 (w).

^{1}H NMR (500 MHz, CD_3OD): δ [ppm] = 7.40 (s, 5 H, CH_{Ar}^{22-26}), 5.99 (s, 1 H, CH^{2}), 4.52 – 4.20 (m, 2 H, CH_2^{9}), 4.09 – 3.85 (m, 1 H, CH^{6}), 3.77 – 3.53 (m, 1 H, CH^{20}), 1.92 – 1.54 (m, 4 H, CH_2), 1.40 – 1.06 (m, 6 H, CH_2).

^{13}C NMR (126 MHz, CD_3OD): δ [ppm] = 169.6 (s, $CONR^{4}$), 157.5 (s, $CONR^{18}$), 131.7 (s, C_{Ar}^{8}), 130.7 (s, CH_{Ar}), 130.3 (s, CH_{Ar}), 130.0 (s, CH_{Ar}), 64.2 (s, CH^{2}), 53.4 (s, CH^{6}), 50.0 (s, CH^{20}), 36.6 (s, CH_2^{9}), 33.5 (s, CH_2), 33.4 (s, CH_2), 27.0 (s, CH_2), 26.6 (s, CH_2), 26.0 (s, CH_2).

^{19}F NMR (376 MHz, CD_3OD): δ [ppm] = -88.26 (t, J = 10.2 Hz, 3 F, CF_3^{10}), AB-signal (δ_A = -116.51, δ_B = -117.81, J_{AB} = 301.2 Hz, A and B are split into t, CF_2^{17a}, additional coupling not resolved, signals broadened), AB-signal (δ_A = -117.81, δ_B = -119.02, J_{AB} = 295.5 Hz, A and B are split into t, CF_2^{17b}, additional coupling not resolved, signals broadened), -126.28 – -127.72 (m, CF_2), -128.68 (s, CF_2), -129.64 (s, CF_2), -133.18 (s, CF_2^{11}). Total integral of CF_2 region normalized with respect to the CF_3^{10} group = 14.

FAB – MS [m/z] (relative intensity): 717.2 (87%) $[M + H]^+$, 591.0 (17%) $[\text{Fragment A} + H]^+$, 590.0 (60%) $[\text{Fragment A }]^+$, 552.0 (15%) $[\text{Fragment A} + H – C_3H_3]^+$, 217.1 (23%) $[\text{Fragment B} + H]^+$.

HRMS – FAB [m/z]: $[M + H]^+$ calculated for $^{12}C_{26}{}^{1}H_{22}{}^{16}O_2{}^{14}N_2{}^{19}F_{17}$, 717.1403; found, 717.1404; Δ = 0.11 mmu.

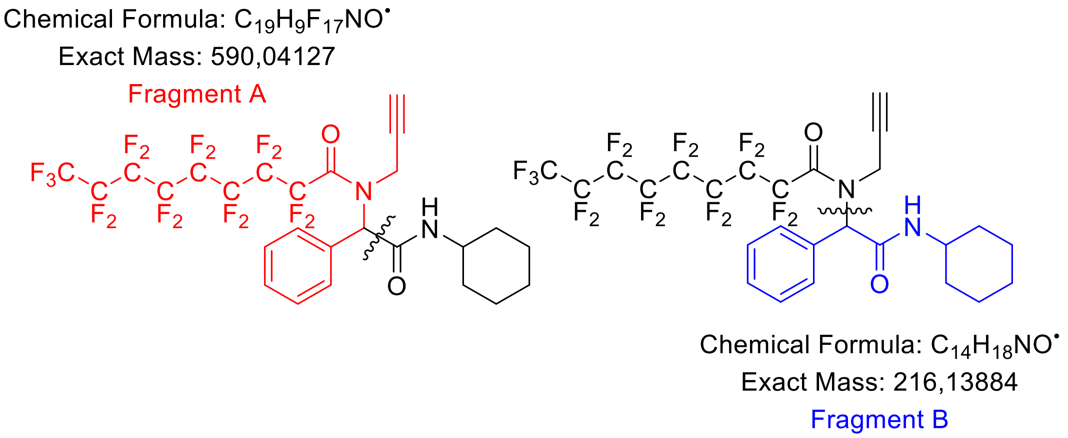

Supplementary Figure 53 | Proposed fragments observed in FAB-MS.

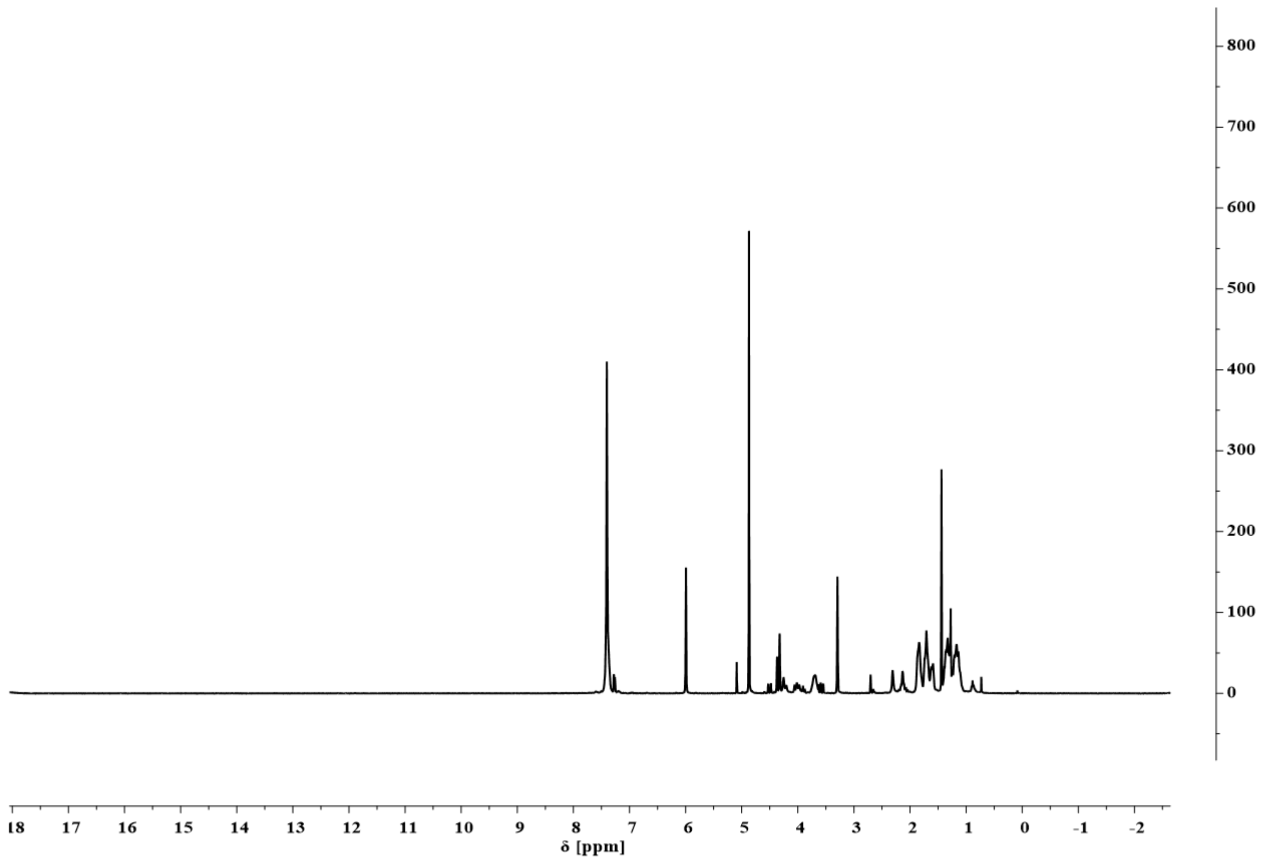

Supplementary Figure 54 | ^{1}H NMR of the title compound recorded in CD_3OD.

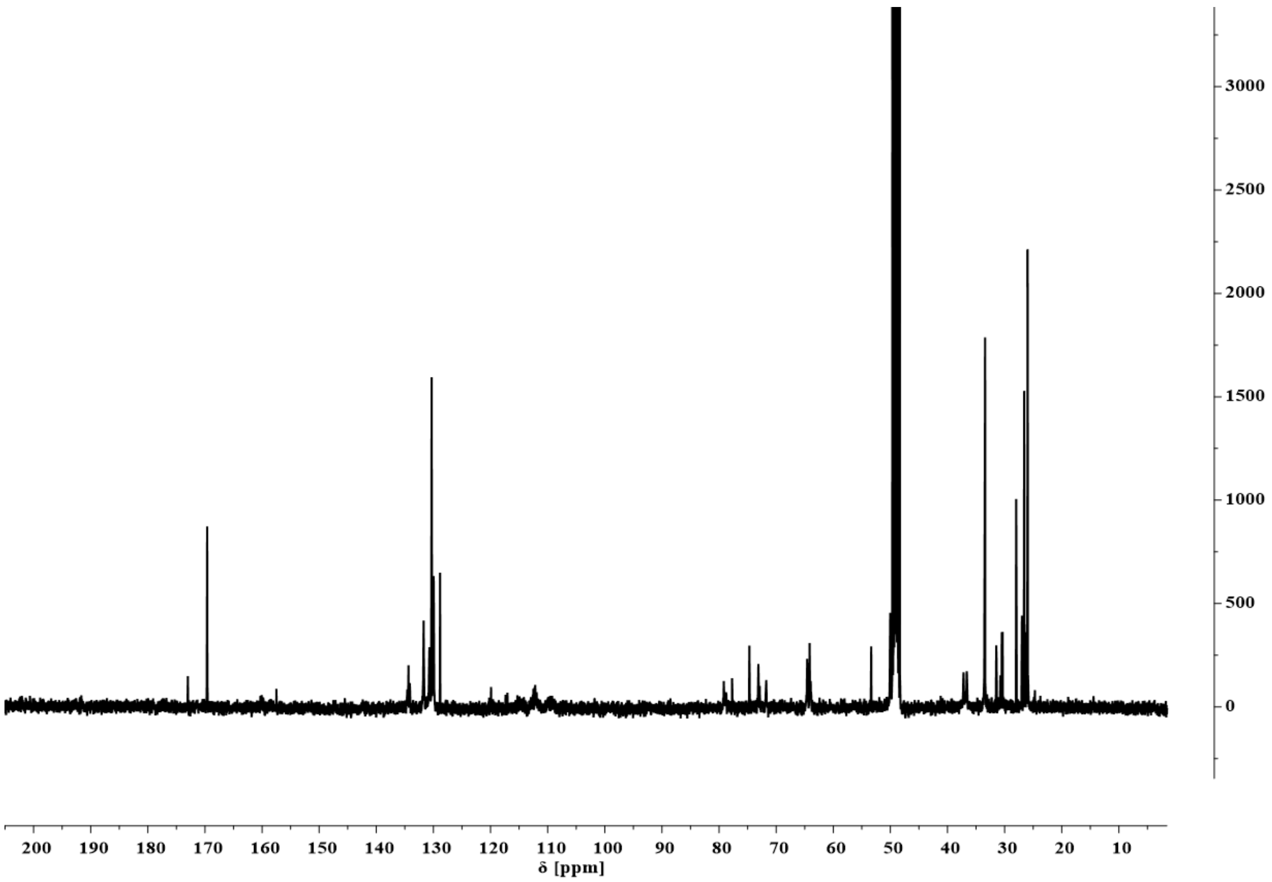

Supplementary Figure 55 | ^{1}H NMR of the title compound recorded in CD_3OD.

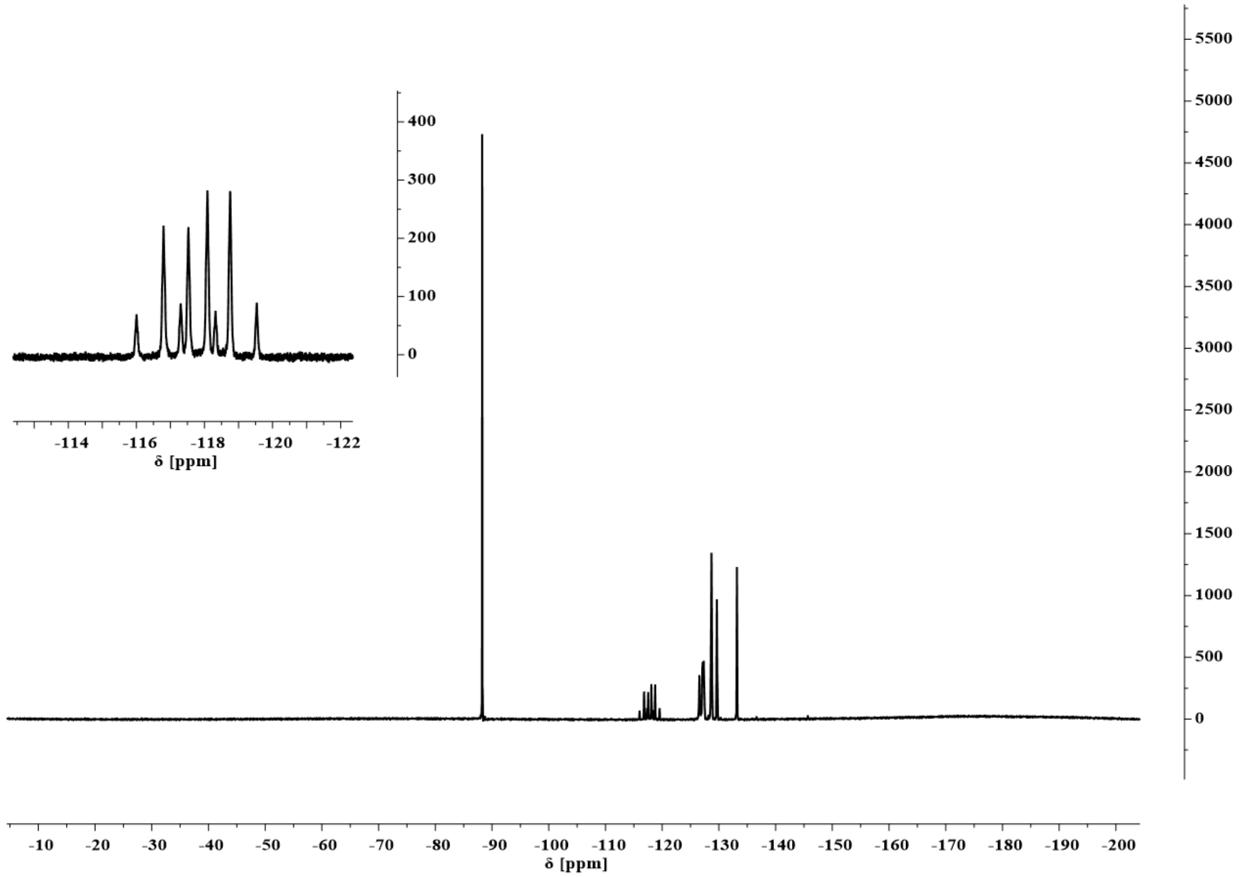

Supplementary Figure 56 | ^{19}F NMR of the title compound recorded in CD_3OD.

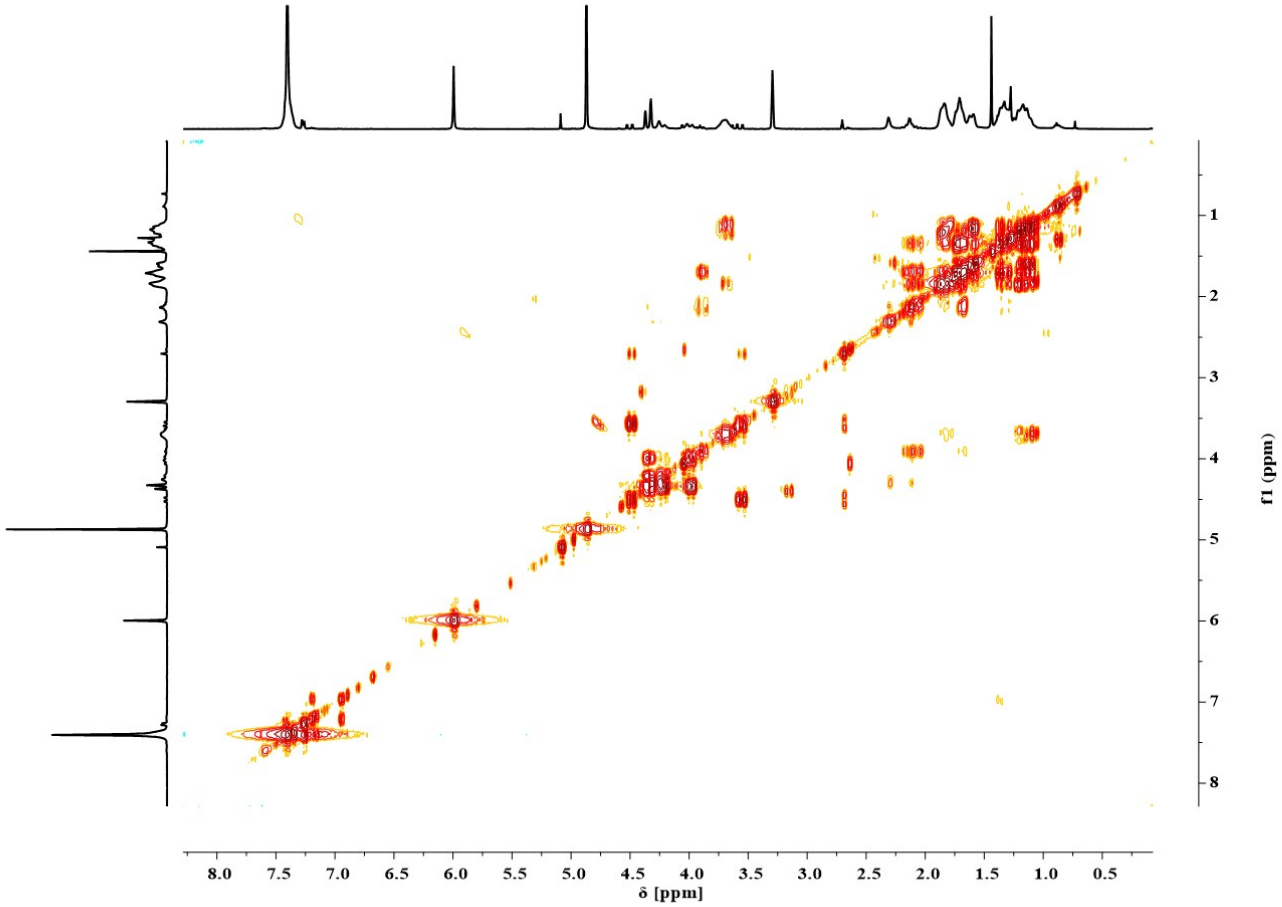

Supplementary Figure 57 | COSY experiment of the title compound recorded in CD_3OD.

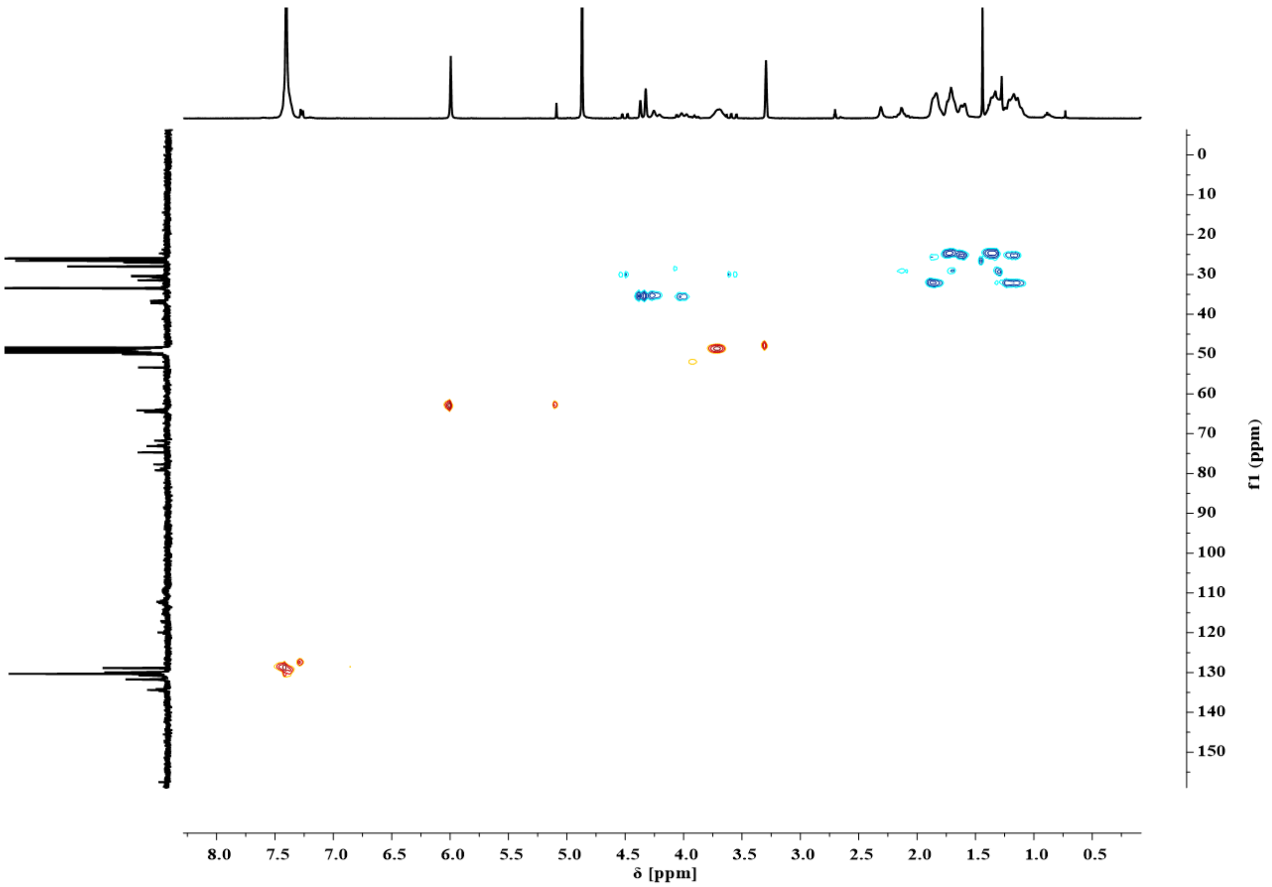

Supplementary Figure 58 | Multiplicity-edited HSQC experiment of the title compound recorded in CD_3OD.

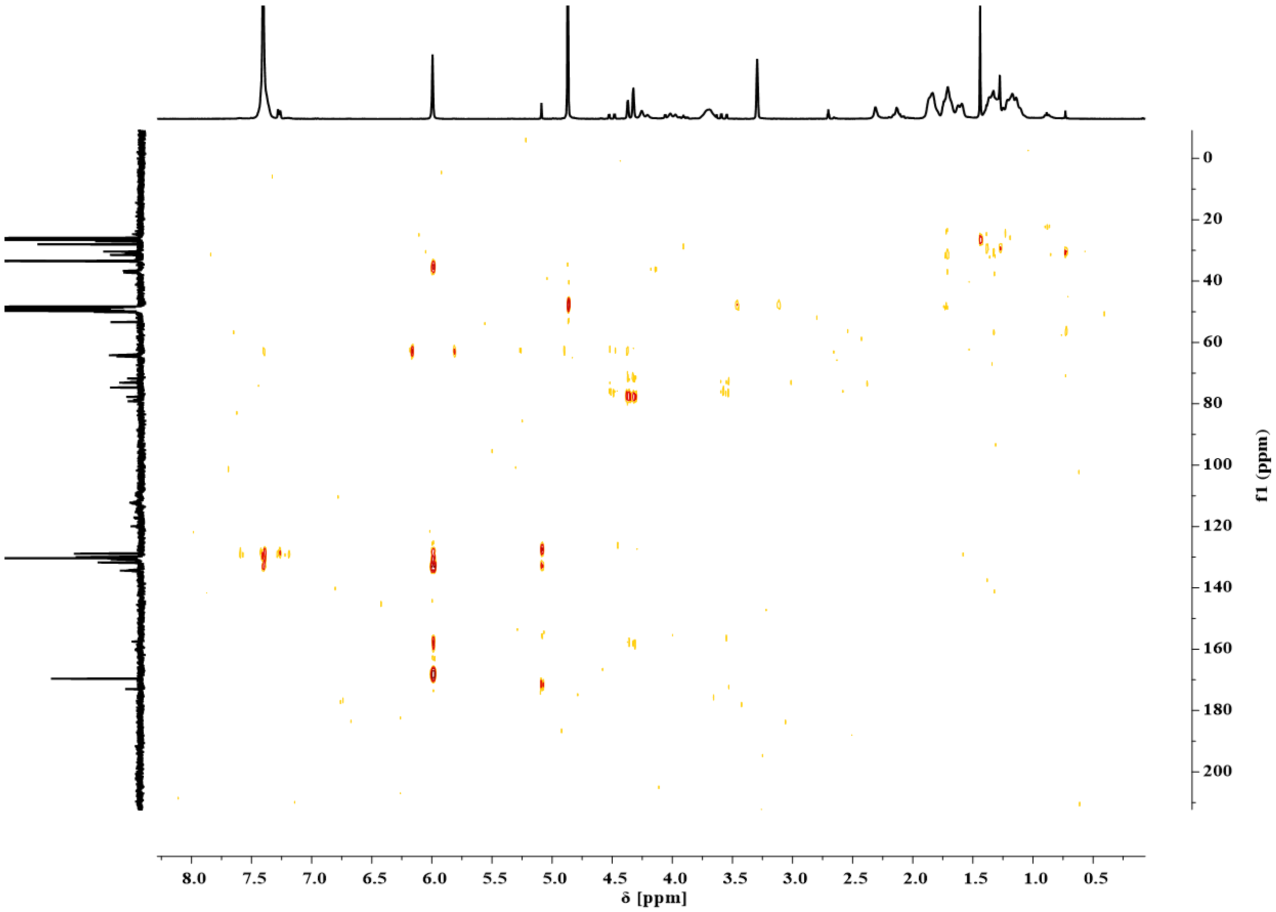

Supplementary Figure 59 | HMBC experiment of the title compound recorded in CD_3OD.

Ugi reaction of perfluorononanoic acid, benzaldehyde, cyclohexylisocyanide and octylamine

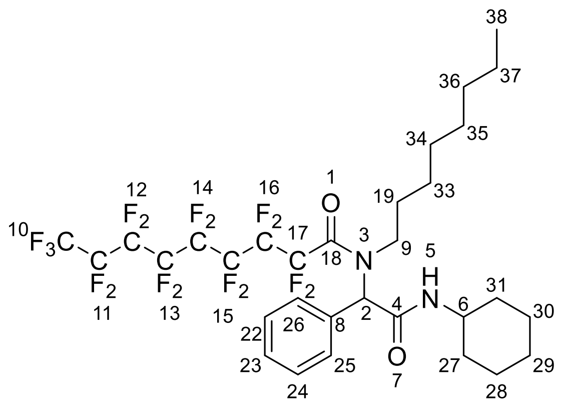

In a 25 mL round bottom flask benzaldehyde (65.4 µL, 68.0 mg, 641 µmol, 1.70 eq.) and octylamine (106 µL, 82.9 mg, 641 µmol, 1.70 eq.) were added. The resulting mixture was stirred for 60 min over sodium sulfate. Pefluornonanoic acid (175 mg, 377 µmol, 1.00 eq.) dissolved in 0.5 mL methanol was added to the solution at room temperature and the resulting mixture was stirred for 2 min. Subsequently, cyclohexylisocyanide (79.7 µL, 70.0 mg, 641 µmol, 1.70 eq.) was added to the stirring mixture. The reaction was stirred for 3 d at room temperature. The crude reaction mixture was dried under reduced pressure and purified *via* column chromatography employing Fluoro*Flash*® silica gel. The fluorous fraction was tested for purity *via* TLC and concentrated under reduced pressure. The remaining perfluorononanoic acid was removed with a short silica gel filter column, eluting with *c*-hexane/ethyl acetate (3:1) to yield the Ugi product as a yellow oil (41.8 mg, 52.8 µmol, 14.0%).

R_f = 0.36 in *c*-hexane/ethyl acetate (6:1). Visualized *via* fluorescent quench and Seebach staining solution.

IR (ATR): ν[cm^{-1}] = 2924.8 (s, ν(C-H)), 2853.3 (s, ν(C-H)), 1768.8 (w, ν(C=O)), 1712.5 (s, ν(C=O)), 1646.9 (s, ν(C=O)), 1450.9 (m), 1375.6 (m), 1240.5 (s), 1214.7 (s), 1150.2 (m), 753.2 (s), 693.2 (s), 496.5 (w).

^{1}H NMR (500 MHz, CDCl$_3$): δ [ppm] = 6.99 – 6.90 (m, 5 H, $CH_{Ar}^{25\text{-}26}$), 4.30 (s, 1 H, CH^{2}), 3.26 – 3.08 (m, 3 H, CH^{6} + CH_2^{9}), 1.28 – 1.16 (m, 4 H, CH_2), 0.96 – 0.68 (m, 16 H, CH_2), 0.49 – 0.28 (m, 3 H, CH_3^{38}).

^{13}C NMR (126 MHz, CDCl$_3$): δ [ppm] = 171.6 (s, $CONR^{4}$), 160.8 (s, $CONR^{18}$), 136.5 (s, C_{Ar}^{8}), 128.7 (s, CH_{Ar}), 128.1 (s, CH_{Ar}), 127.4 (s, CH_{Ar}), 65.5 (s, CH^{2}), 63.4 (s, CH_2), 62.0 (s, CH^{6}), 32.0 (s, CH_2), 31.1 (s, CH_2), 29.8 (s, CH_2), 29.6 (s, CH_2), 29.4 (s, CH_2), 27.5 (s, CH_2), 26.8(s, CH_2), 25.2 (s, CH_2), 22.8 (s, CH_2), 14.2 (s, CH_3^{8}).

^{19}F NMR (376 MHz, CDCl$_3$): δ [ppm] = -85.11 (t, J = 9.0 Hz, 3 F, CF_3^{10}), AB-signal (δ_A = -112.92, δ_B = -114.05, J_{AB} =295.5 Hz, A and B are split into t, CF_2^{17a}, additional coupling not resolved, signals

broadened), AB-signal (δ_A = -115.47, δ_B = -116.59, J_{AB} = 291.8 Hz, A and B are split into t, CF_2[17b], additional coupling not resolved, signals broadened), -123.73 – -124.95 (m, CF_2), -126.12 (s, CF_2), -127.05 (s, CF_2), -130.46 (s, CF_2[11]). Total integral of CF_2 region normalized with respect to the CF_3[10] group = 14.

FAB – MS [*m/z*] (relative intensity): 791.3 (40%) $[M + H]^+$, 552.0 (22%) $[\text{Fragment A} + H]^+$, 118.0 (23%) $[\text{Fragment B} – H]^+$, 98.0 (31%) $[\text{Fragment C}]^+$.

HRMS – FAB [*m/z*]: $[M + H]^+$ calculated for $^{12}C_{31}{}^{1}H_{36}{}^{16}O_2{}^{14}N_2{}^{19}F_{17}$, 797.2500; found, 791.2501; Δ = 0.14 mmu.

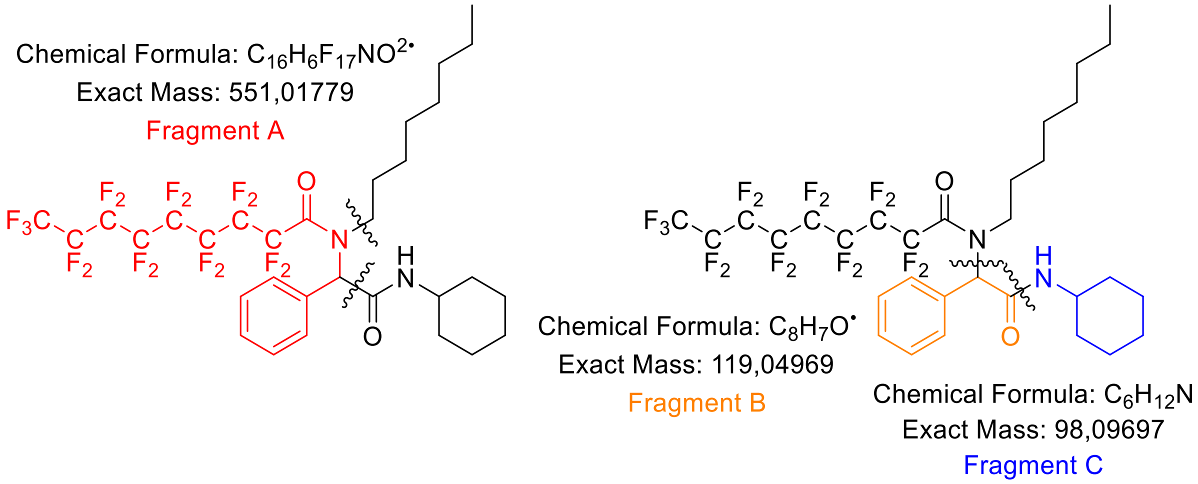

Supplementary Figure 60 | Proposed fragments observed in FAB-MS.

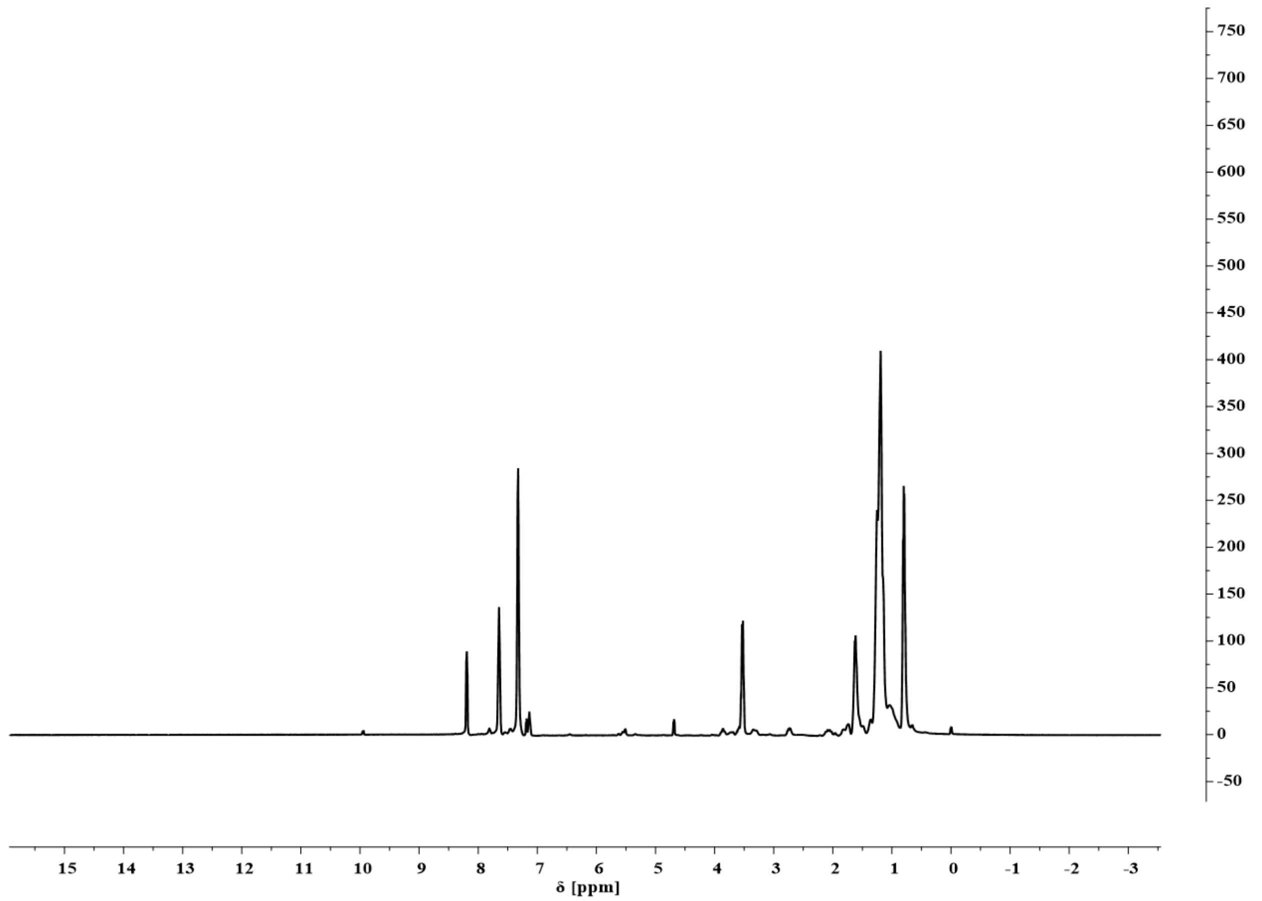

Supplementary Figure 61 | ^{1}H NMR of the title compound recorded in $CDCl_3$

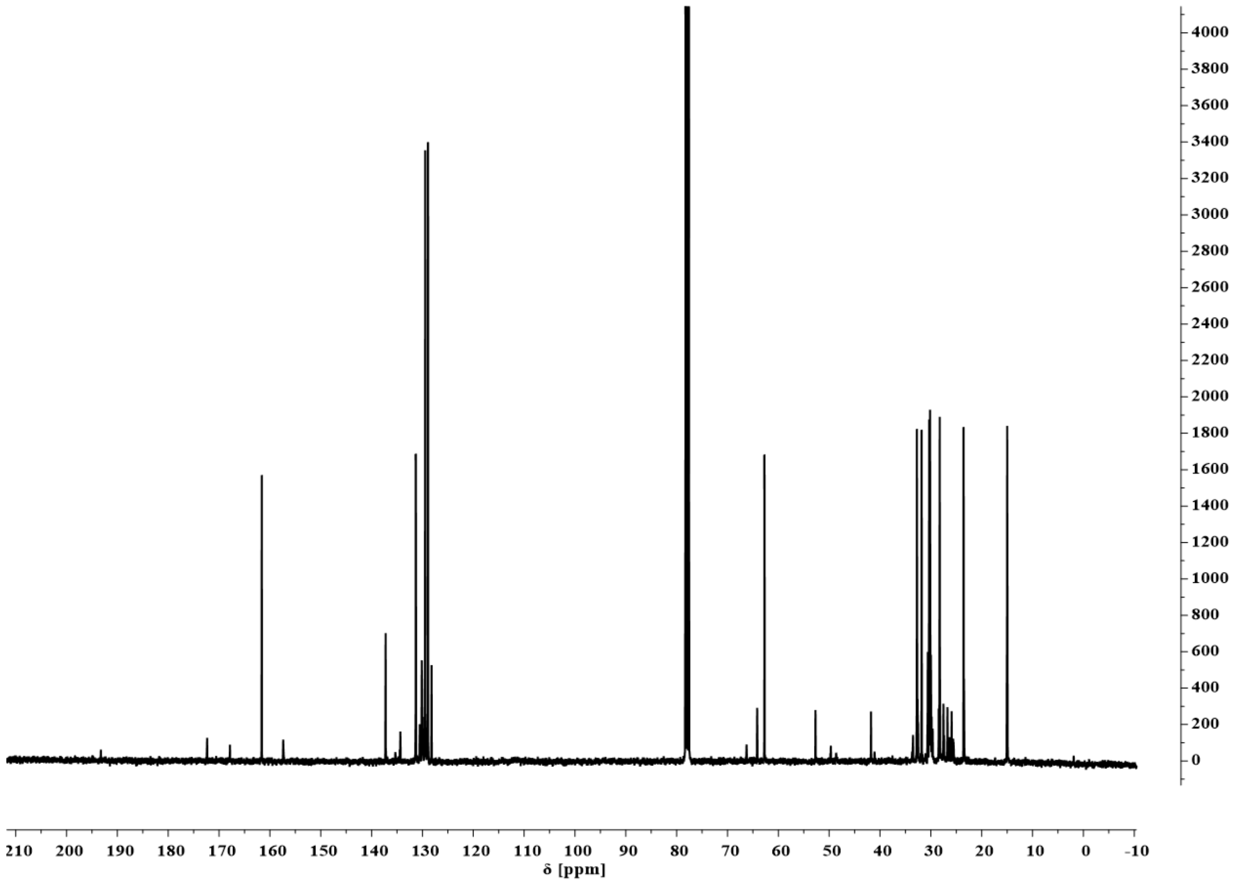

Supplementary Figure 62 | ^{13}C NMR of the title compound recorded in $CDCl_3$.

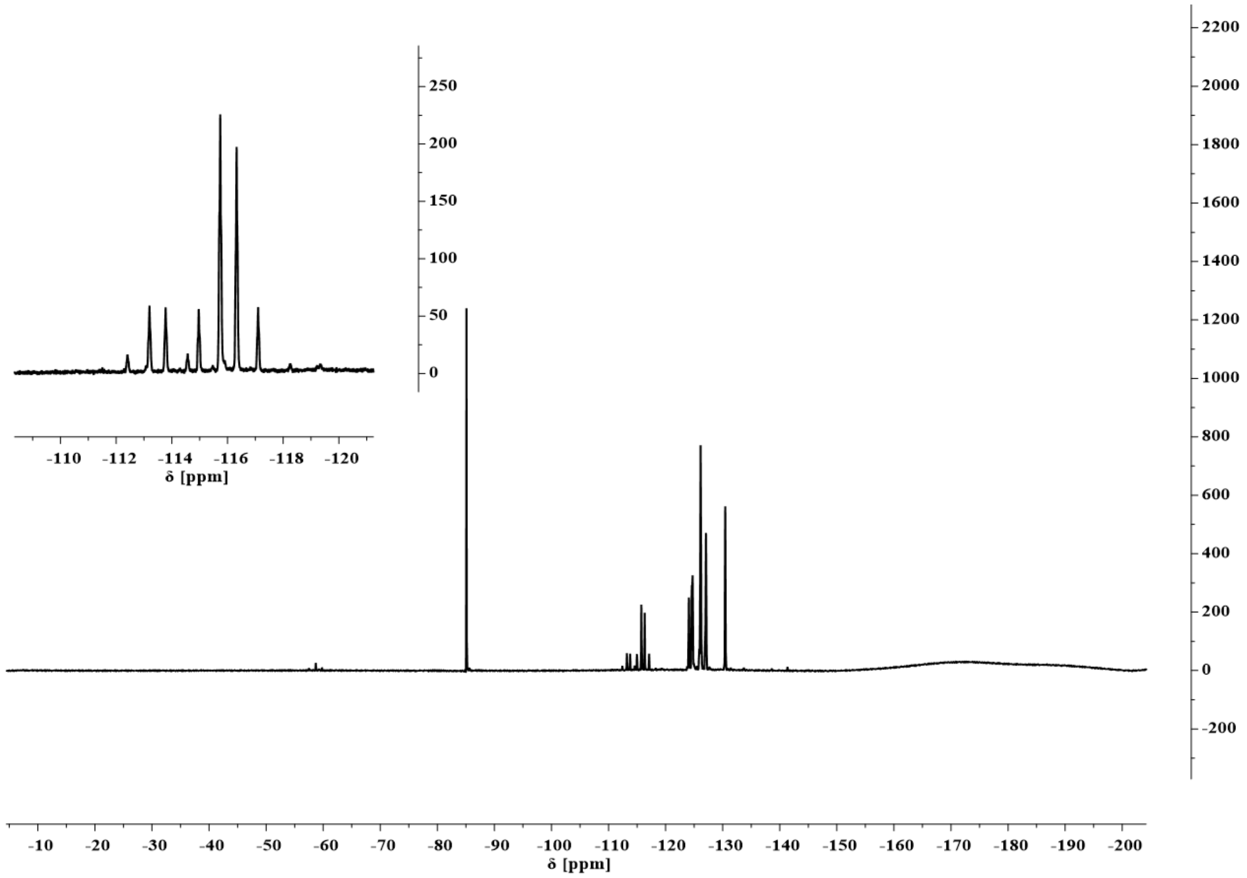

Supplementary Figure 63 | ^{19}F NMR of the title compound recorded in $CDCl_3$.

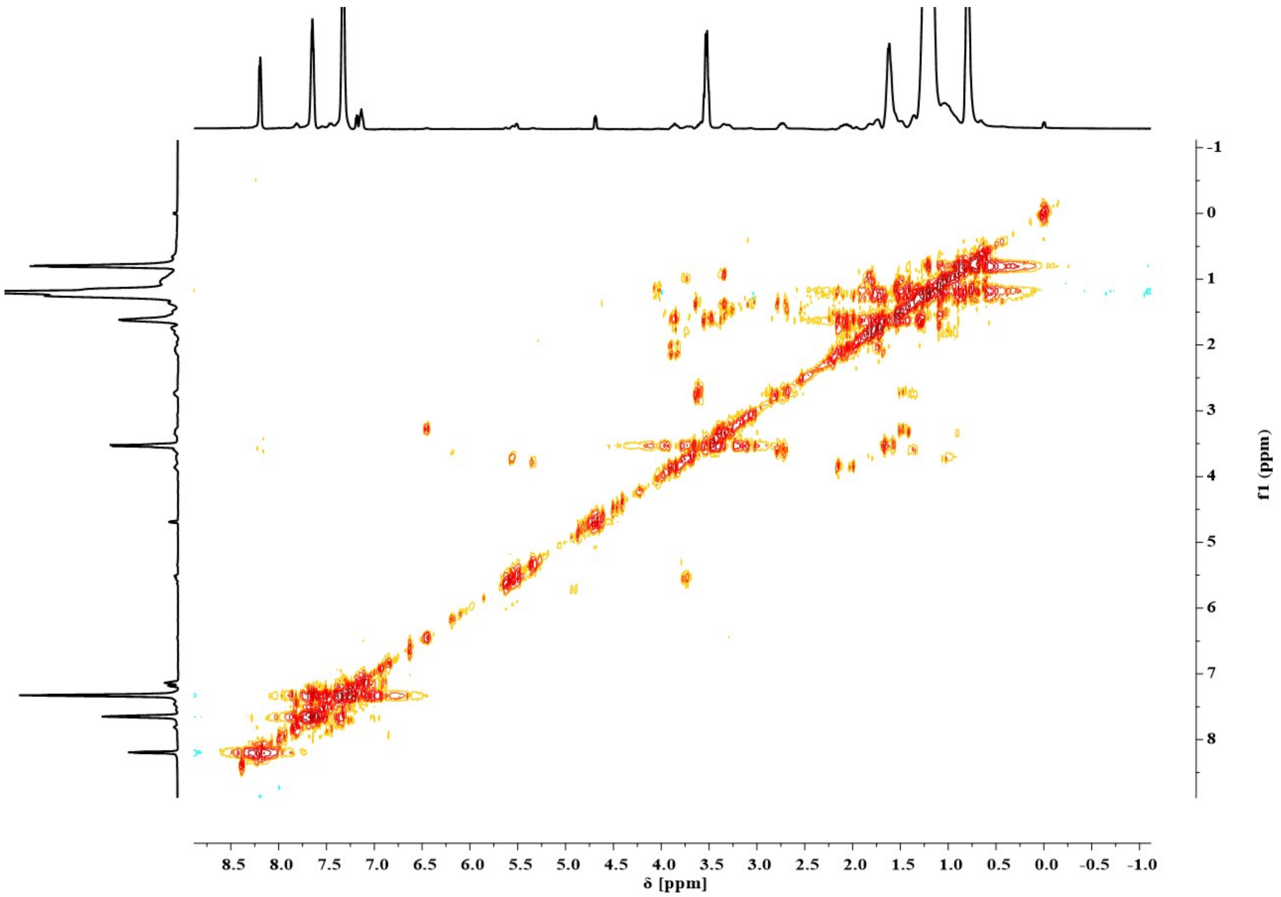

Supplementary Figure 64 | COSY experiment of the title compound recorded in $CDCl_3$.

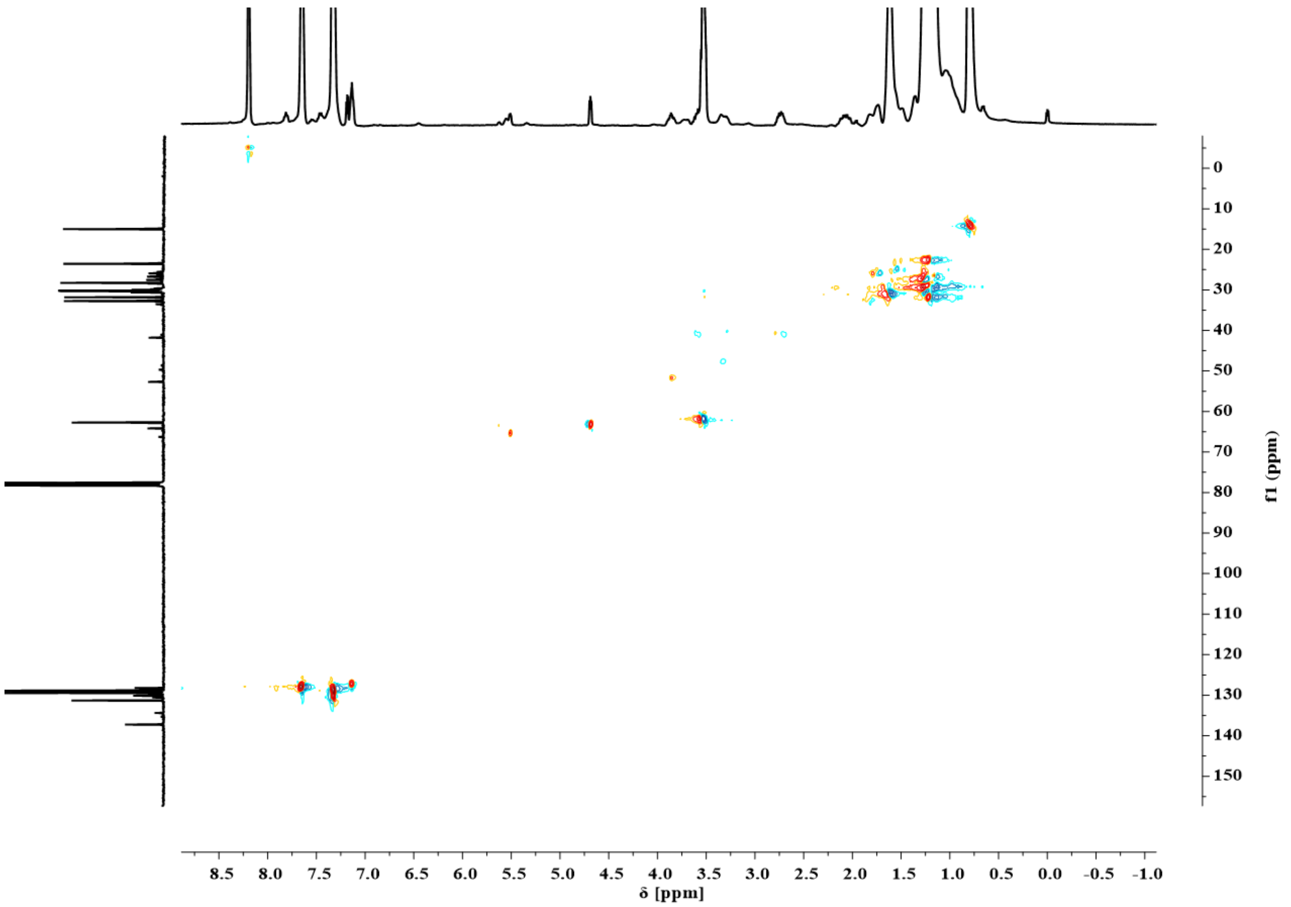

Supplementary Figure 65 | Multiplicity-edited HSQC experiment of the title compound recorded in $CDCl_3$.

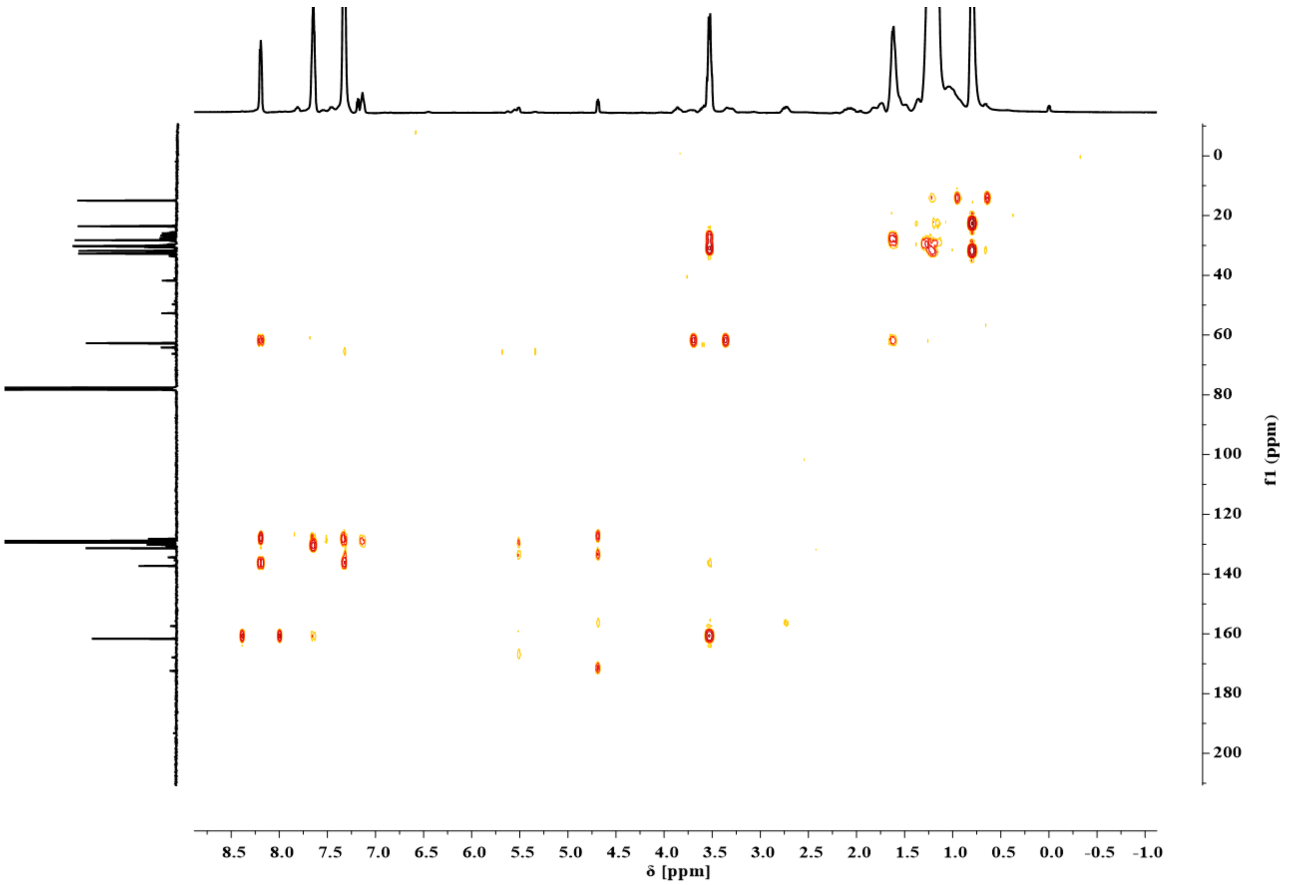

Supplementary Figure 66 | HMBC experiment of the title compound recorded in $CDCl_3$

Ugi reaction of pefluornonanoic acid, *p*-anisaldehyde, *4*-methoxyphenyl-isocyanide and propargylamine

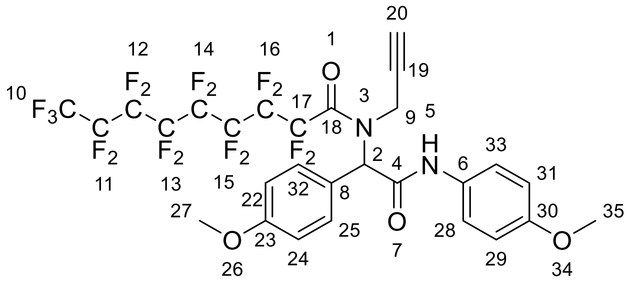

In a 25 mL round bottom flask *p*-anisaldehyde (77.9 µL, 87.3 mg, 641 µmol, 1.70 eq.) and propargylamine (41.1 µL, 35.3 mg, 641 µmol, 1.70 eq.) were added. The resulting mixture was stirred for 60 min over sodium sulfate. Perfluorononanoic acid (175 mg, 377 µmol, 1.00 eq.) dissolved in 0.5 mL methanol was added to the solution at room temperature and the resulting mixture was stirred for 2 min. Subsequently, *4*-methoxyphenyl-isocyanide (85.4 mg, 641 µmol, 1.70 eq.) was added to the stirring mixture. The reaction was stirred for 3 d at room temperature. The crude reaction mixture was dried under reduced pressure and purified *via* column chromatography employing Fluoro*Flash*® silica gel. The fluorous fraction was tested for purity *via* TLC and concentrated under reduced pressure. The remaining perfluorononanoic acid was removed with a short silica gel filter column, eluting with *c*-hexane/ethyl acetate (3:1) to yield the Ugi product as a yellow oil (190 mg, 247 µmol, 65.4%).

R_f = 0.30 in *c*-hexane/ethyl acetate (4:1). Visualized *via* fluorescent quench and Seebach staining solution.

IR (ATR): ν [cm^{-1}] = 3299.7 (br, ν(N-H)), 1680.0 (s, ν(C=O)), 1656.2 (s), 1606.8 (m), 1549.2 (m), 1510.5 (vs), 1462.2 (m), 1418.1 (m), 1300.9 (m), 1202.6 (vs), 1143.7 (vs), 1034.2 (s), 1004.1 (m), 945.7 (m), 828.0 (s), 781.1 (m), 719.5 (m), 657.6 (s), 632.1 (s), 526.1 (s), 441.3 (w).

^{1}H NMR (400 MHz, CDCl$_3$): δ [ppm] = 7.59 – 7.29 (m, 4 H, $CH_{Ar}^{25,32,28,33}$), 6.93 (d, J = 8.7 Hz, 2 H, $CH_{Ar}^{22,24\ or\ 29,31}$), 6.81 (d, J = 8.4 Hz, 2 H, $CH_{Ar}^{22,24\ or\ 29,31}$), 6.00 (s, 1 H, CH^{2}), 4.45-4.32 (m, 2 H, CH_2^{9}), 3.89 (s, 1 H, CH^{20}), 3.83 (s, 3 H, $CH_3^{27\ or\ 35}$), 3.77 (s, 3 H, $CH_3^{27\ or\ 3}$).

^{13}C NMR (101 MHz, CDCl$_3$): δ [ppm] = 171.4 (s, $CONR^{4}$), 166.2 (s, $CONR^{18}$), 160.8 (s, $C_{Ar}^{23\ or\ 30}$), 156.9 (s, $C_{Ar}^{23\ or\ 30}$), 132.0 (s, $CH_{Ar}^{25,32\ or\ 28,33}$), 130.3 (s, C_{Ar}^{8}), 123.9 (s, C_{Ar}^{6}), 122.0 (s, $CH_{Ar}^{25,32\ or\ 28,33}$), 114.8 (s, $CH_{Ar}^{22,24\ or\ 29,31}$), 114.3 $CH_{Ar}^{22,24\ or\ 29,31}$), 64.0 (s, CH^{2}), 55.6 (s, $CH_3^{27\ or\ 3}$), 55.5 (s, $CH_3^{27\ or\ 3}$), 36.0 (s, CH_2^{9}).

^{19}F NMR (376 MHz, $CDCl_3$): δ [ppm] = -85.13 (t, J = 9.9 Hz, 3 F, $CF_3{}^{10}$), AB-signal (δ_A = -113.30, δ_B = -114.73, J_{AB} = 299.3 Hz, additional coupling not resolved, signals broadened, $CF_2{}^{17a}$), AB-signal (δ_A = -115.21, δ_B = -116.14, J_{AB} = 293.6 Hz, additional coupling not resolved, signals broadened, $CF_2{}^{17b}$), -124.78 (s, CF_2), -126.13 (s, CF_2), -127.07 (s, CF_2), -130.46 (s, $CF_2{}^{11}$). Total integral of CF_2 region normalized with respect to the $CF_3{}^{10}$ group = 14.

FAB – MS [*m/z*] (relative intensity): 771.2 (33%) $[M + H]^+$, 770.1 (65%) $[M]^+$, 620.1 (65%) [Fragment A $]^+$, 271.1 (33%) [Fragment B + $H]^+$.

HRMS – FAB [*m/z*]: $[M]^+$ calculated for $^{12}C_{28}{}^{1}H_{19}{}^{16}O_4{}^{14}N_2{}^{19}F_{17}$, 770.1068; found, 770.1070; Δ = 0.22 mmu.

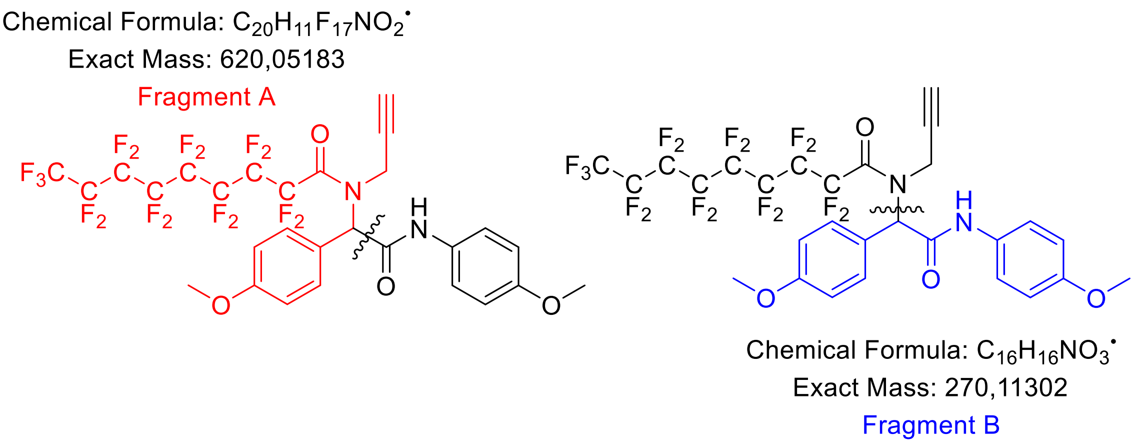

Supplementary Figure 67 | Proposed fragments observed in FAB-MS.

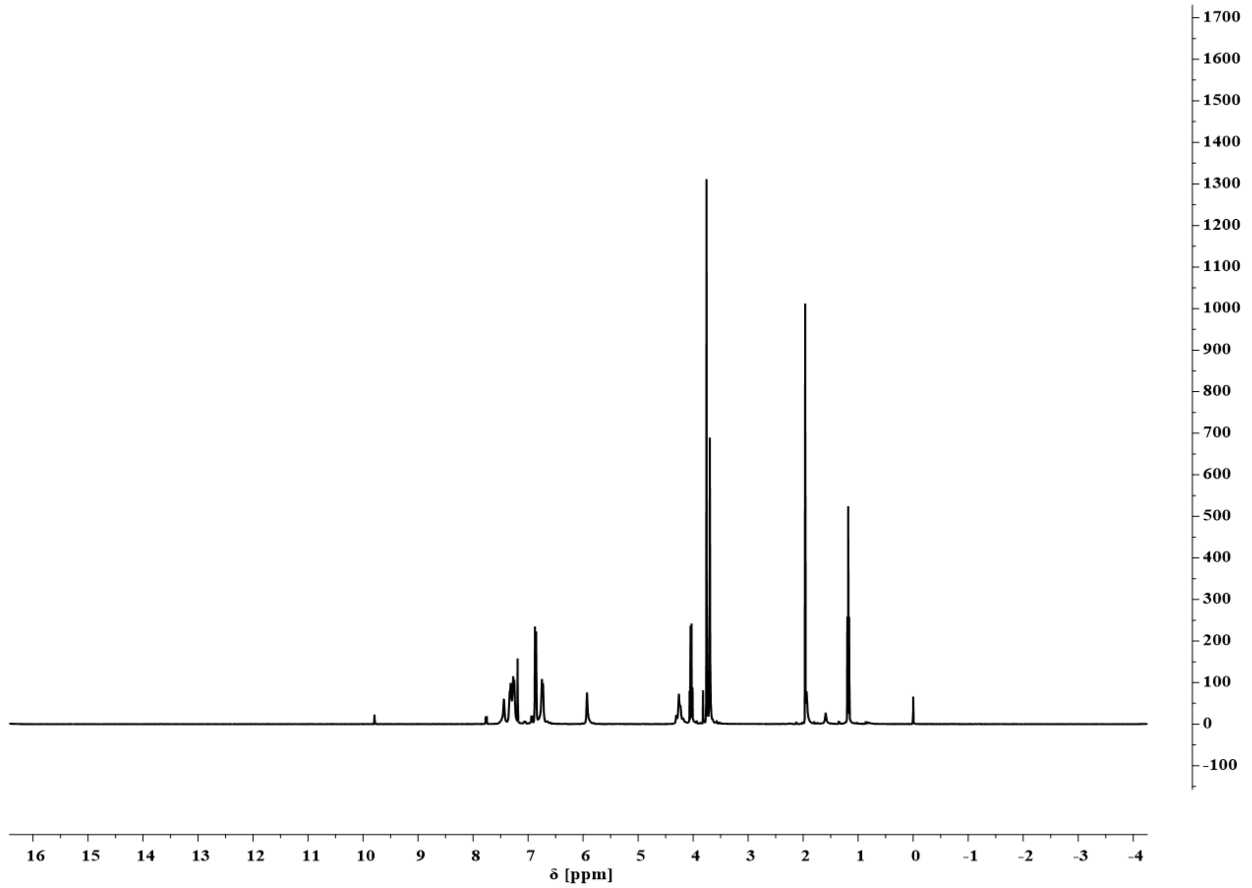

Supplementary Figure 68 | ^{1}H NMR of the title compound recorded in $CDCl_3$.

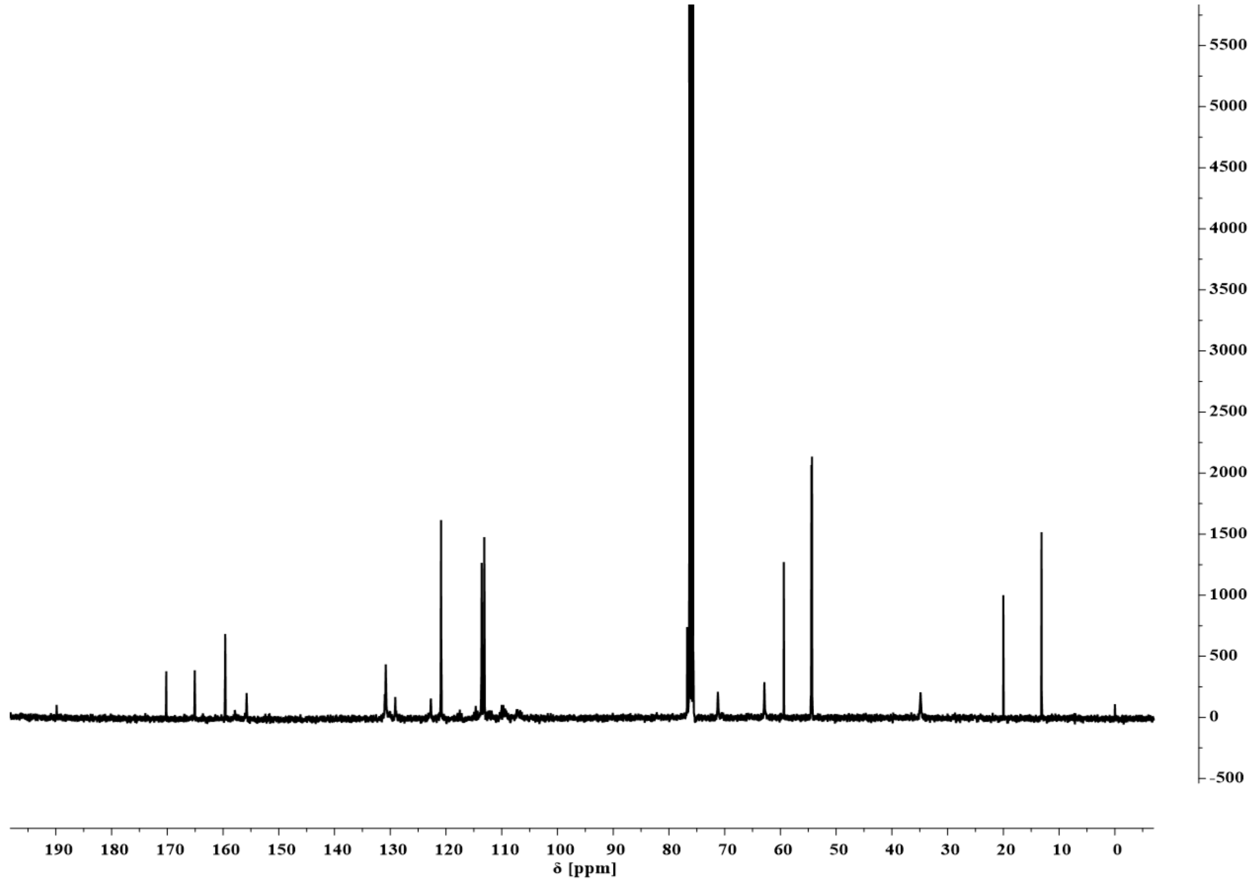

Supplementary Figure 70 | ^{13}C NMR of the title compound recorded in $CDCl_3$.

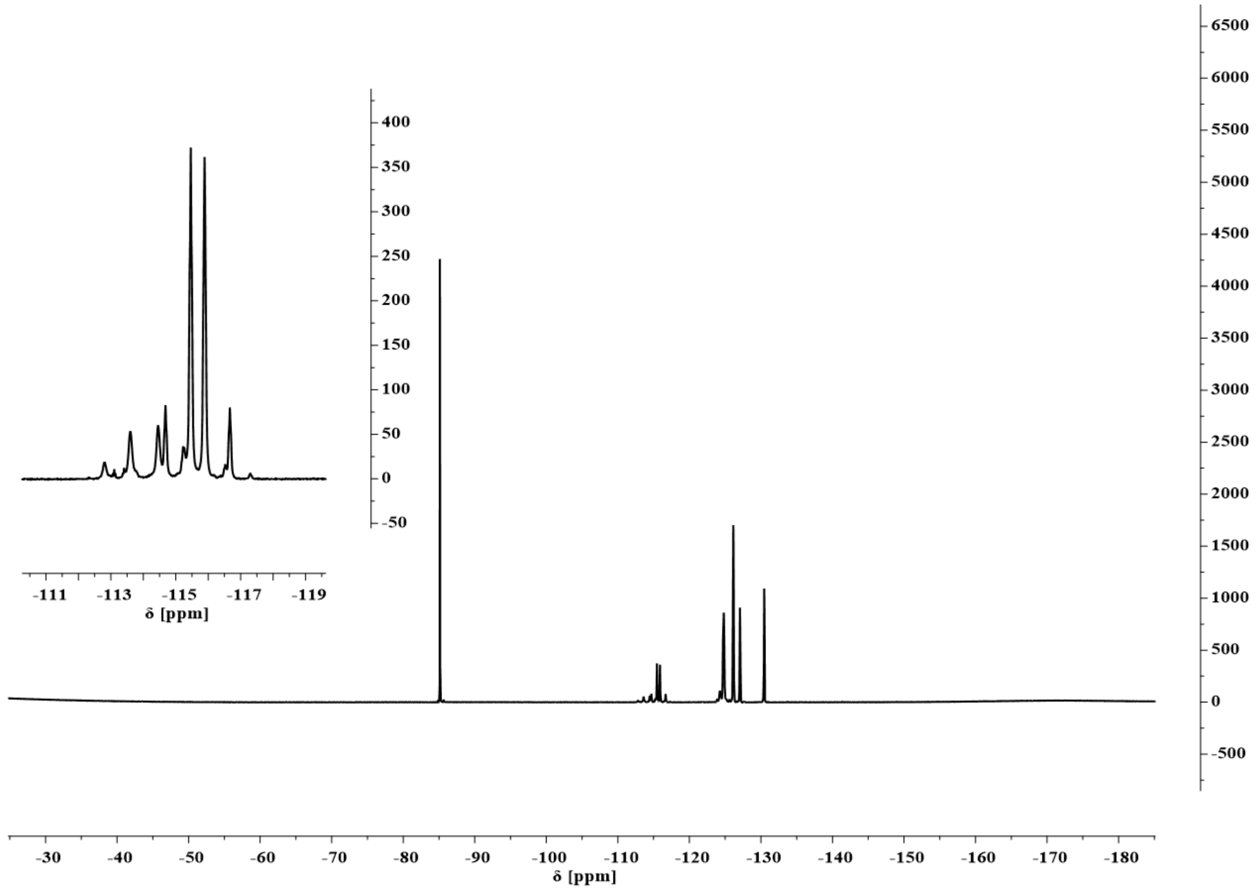

Supplementary Figure 71 | ^{19}F NMR of the title compound recorded in $CDCl_3$.

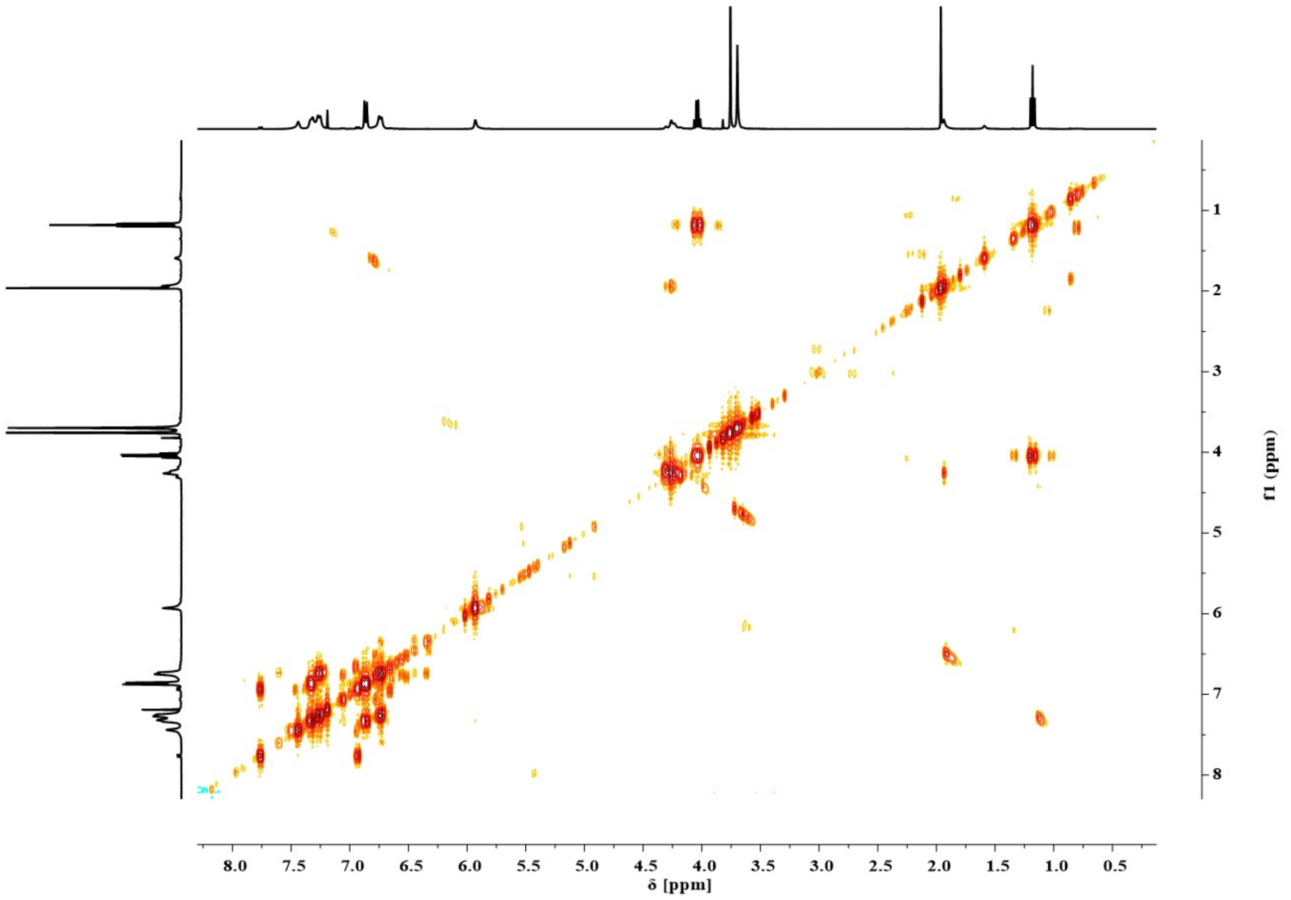

Supplementary Figure 72 | COSY experiment of the title compound recorded in $CDCl_3$.

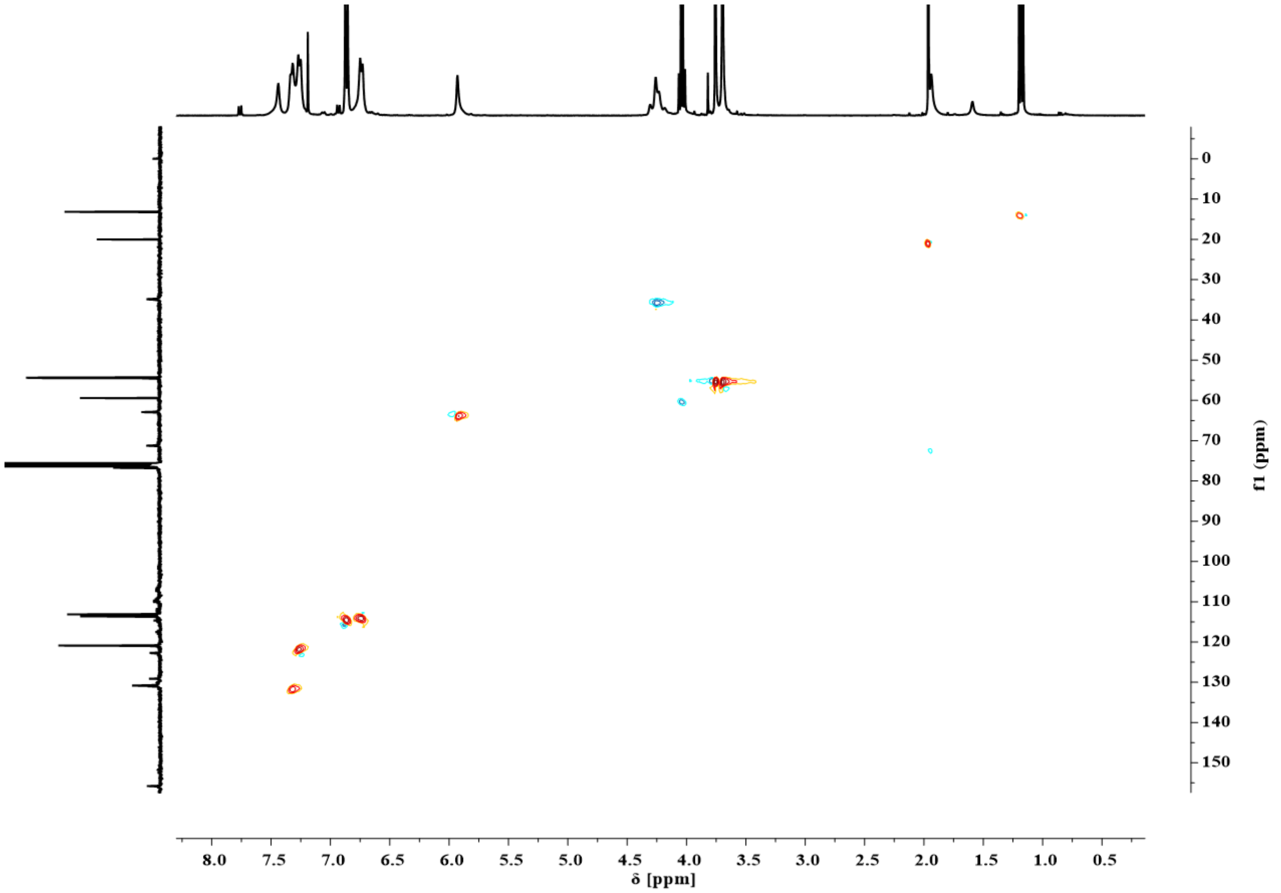

Supplementary Figure 73 | Multiplicity-edited HSQC experiment of the title compound recorded in $CDCl_3$.

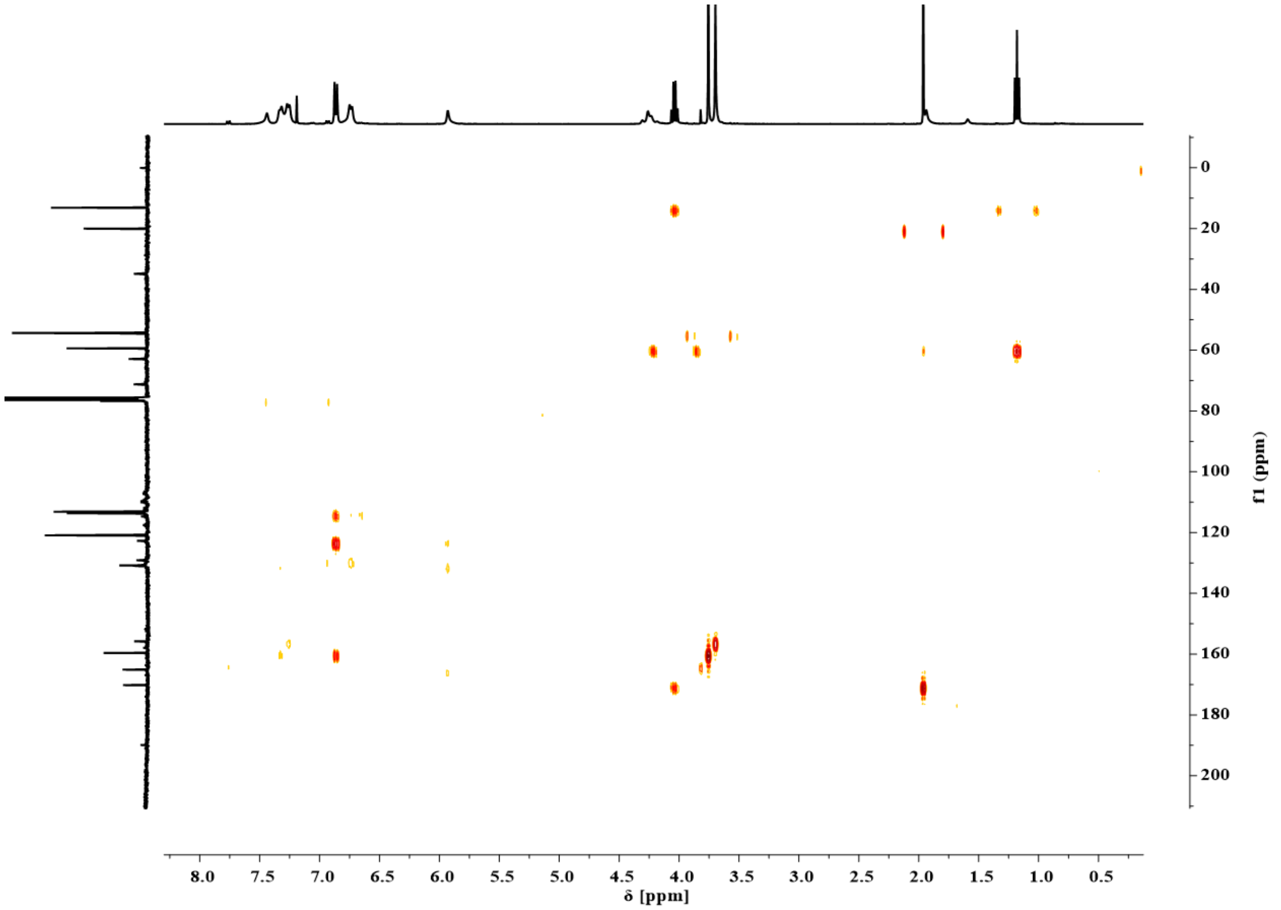

Supplementary Figure 74 | HMBC experiment of the title compound recorded in $CDCl_3$.

Ugi reaction of perfluorononanoic acid, *p*-anisaldehyde, 2,6-dimethylphenyl-isocyanide and propargylamine

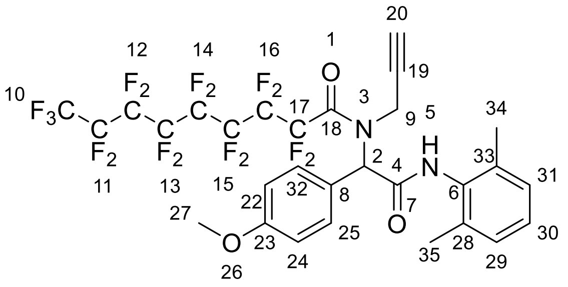

In a 25 mL round bottom flask *p*-anisaldehyde (77.9 µL, 87.3 mg, 641 µmol, 1.70 eq.) and propargylamine (41.1 µL, 35.3 mg, 641 µmol, 1.70 eq.) were added. The resulting mixture was stirred for 60 min over sodium sulfate. Perfluorononanoic acid (175 mg, 377 µmol, 1.00 eq.) dissolved in 0.5 mL methanol was added to the solution at room temperature and the resulting mixture was stirred for 2 min. Subsequently, 2,6-dimethylphenyl-isocyanide (84.1 mg, 641 µmol, 1.70 eq.) was added to the stirring mixture. The reaction was stirred for 3 d at room temperature. The crude reaction mixture was dried under reduced pressure and purified *via* column chromatography employing Fluoro*Flash*® silica gel. The fluorous fraction was tested for purity *via* TLC and concentrated under reduced pressure. The remaining perfluorononanoic acid was removed with a short silica gel filter column, eluting with *c*-hexane/ethyl acetate (3:1) to yield the Ugi product as a yellow oil (114 mg, 149 µmol, 39.5%).

R_f = 0.30 in *c*-hexane/ethyl acetate (4:1). Visualized *via* fluorescent quench and Seebach staining solution.

IR (ATR): ν [cm^{-1}] = 3330.2 (m, ν(N-H)), 1694.4 (m), 1665.2 (s), 1609.3 (w), 1534.7 (m), 1513.8 (m), 1426.0 (w), 1362.7 (w), 1205.8 (w), 1178.3 (vs), 1144.8 (vs), 1109.7 (vs), 1072.8 (s), 1027.5 (m), 1003.6 (s), 937.2 (s), 920.3 (m), 831.6 (m), 802.9 (m), 771.0 (m), 765.8 (m), 703.0 (s), 663.7 (vs), 633.2 (s), 587.1 (m), 559.7 (m), 559.7 (s), 525.1 (s), 444.7 (w).

^{1}H NMR (400 MHz, CDCl$_3$): δ [ppm] = 7.51 (d, J = 9.5 Hz, 2 H, $CH_{Ar}^{25,32}$), 7.11 – 7.01 (m, 3 H, $CH_{Ar}^{29,30,31}$), 6.97 (d, J = 8.7 Hz, 2 H, $CH_{Ar}^{22,24}$), 5.99 (s, 1 H, CH^{2}), 4.32 (s, 2 H, CH_2^{9}), 3.85 (s, 3 H, CH_3^{37}), 2.16 (s, 6 H, $CH_3^{34,35}$).

^{13}C NMR (101 MHz, CDCl$_3$): δ [ppm] = 166.7 (s, $CONR^{4}$), 160.9 (s, C_{Ar}^{23}), 135.6 (s, $C_{Ar}^{8\ or\ 6}$), 132.2 (s, $CH_{Ar}^{25,32}$), 128.4 (s, $C_{Ar}^{8\ or\ 6}$), 127.8 (s, $CH_{Ar}^{29,30,31}$), 114.7 (s, $CH_{Ar}^{22,24}$), 55.5 (CH_3^{27}), 36.0 (s, CH_2^{9}), 18.6 ($CH_3^{34,35}$).

^{19}F NMR (376 MHz, $CDCl_3$): δ [ppm] = -85.10 (t, J = 9.9 Hz, 3 F, $CF_3{}^{10}$), AB-signal (δ_A = -112.63, δ_B = -114.35, J_{AB} = 297.4 Hz, additional coupling not resolved, signals broadened, $CF_2{}^{17a}$), AB-signal (δ_A = -115.23, δ_B = -116.38, J_{AB} = 293.6 Hz, additional coupling not resolved, signals broadened, $CF_2{}^{17b}$), -124.82 (s, CF_2), -126.13 (s, CF_2), -127.06 (s, CF_2), -130.44 (s, $CF_2{}^{11}$). Total integral of CF_2 region normalized with respect to the $CF_3{}^{10}$ group = 14.

FAB – MS [m/z] (relative intensity): 769.1 (60%) $[M + H]^+$, 620.1 (85%) [Fragment A $]^+$.

HRMS – FAB [m/z]: $[M]^+$ calculated for $^{12}C_{29}{}^{1}H_{22}{}^{16}O_3{}^{14}N_2{}^{19}F_{17}$, 769.1353; found, 769.1355; Δ = 0.18 mmu.

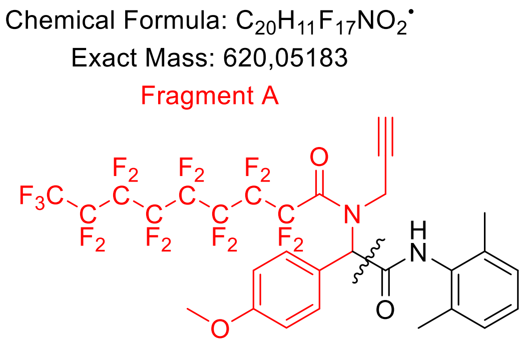

Supplementary Figure 75 | Proposed fragments observed in FAB-MS.

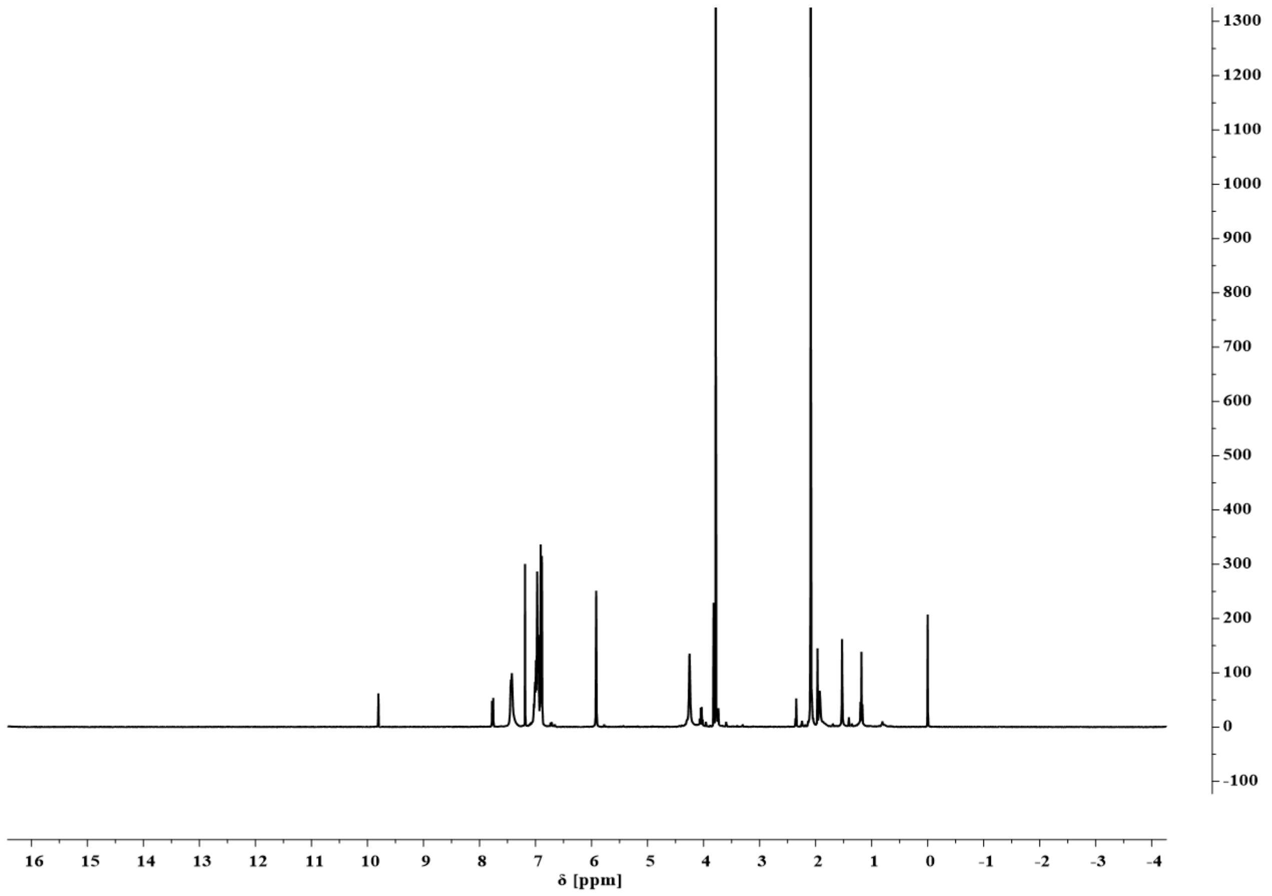

Supplementary Figure 76 | ^{1}H NMR of the title compound recorded in $CDCl_3$.

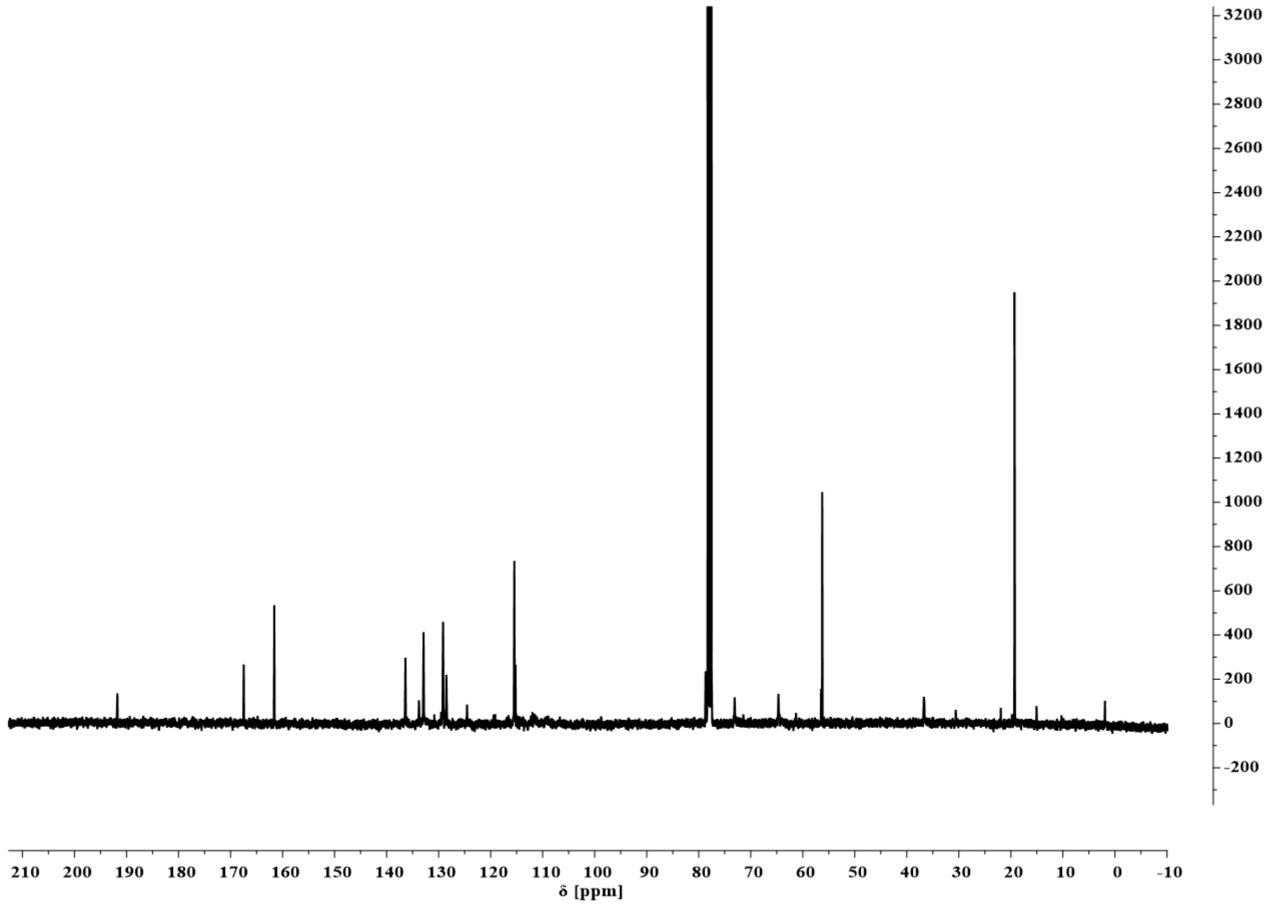

Supplementary Figure 77 | ^{13}C NMR of the title compound recorded in $CDCl_3$.

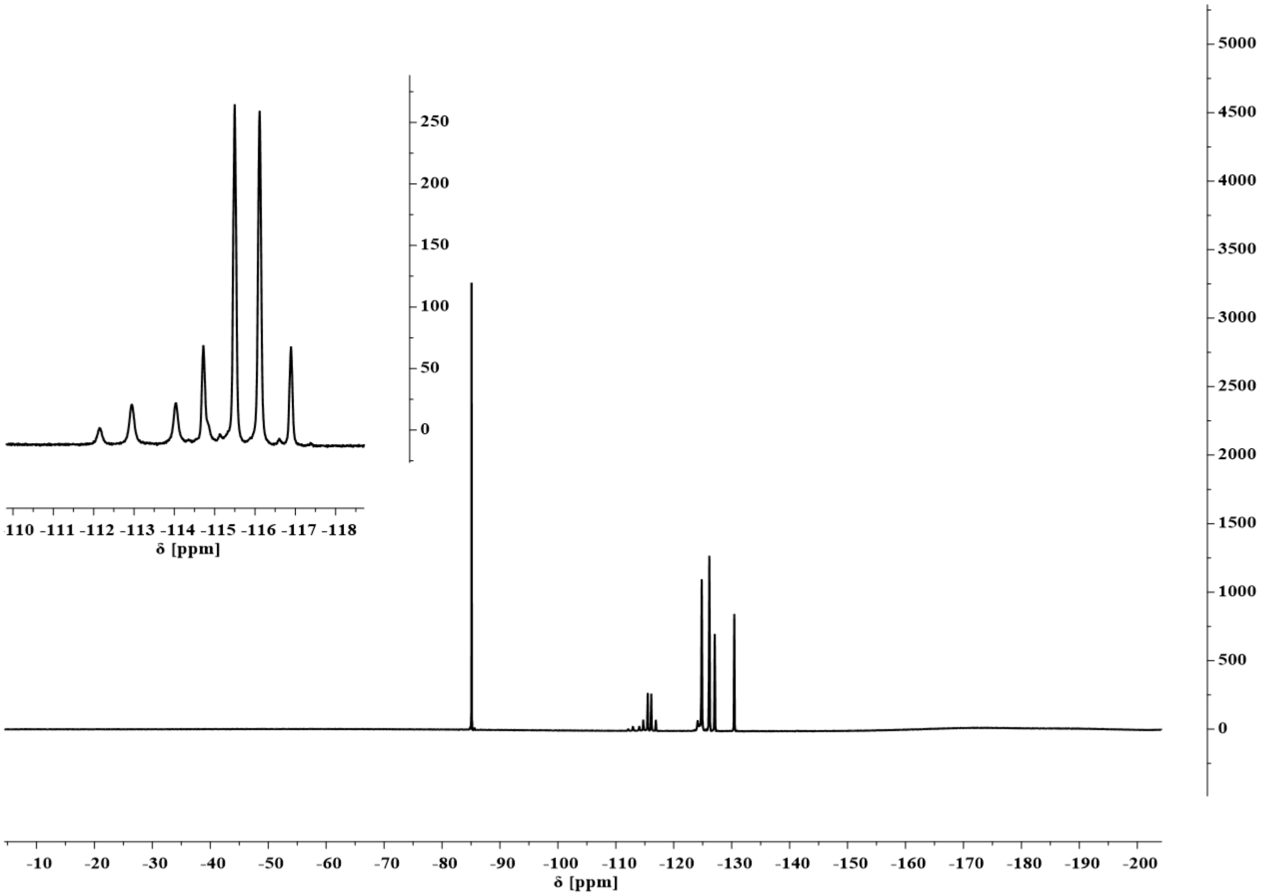

Supplementary Figure 78 | ^{19}F NMR of the title compound recorded in $CDCl_3$.

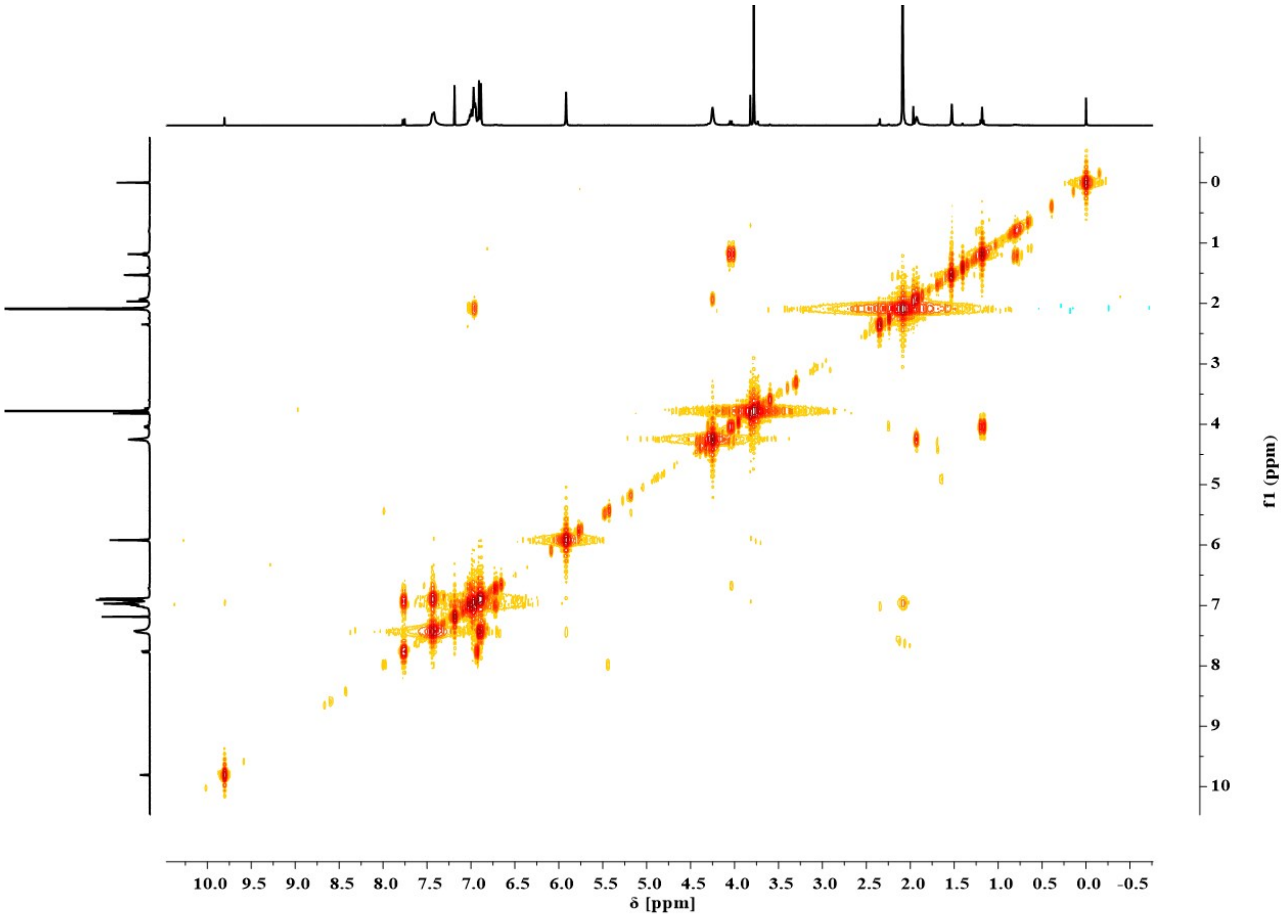

Supplementary Figure 79 | COSY experiment of the title compound recorded in $CDCl_3$.

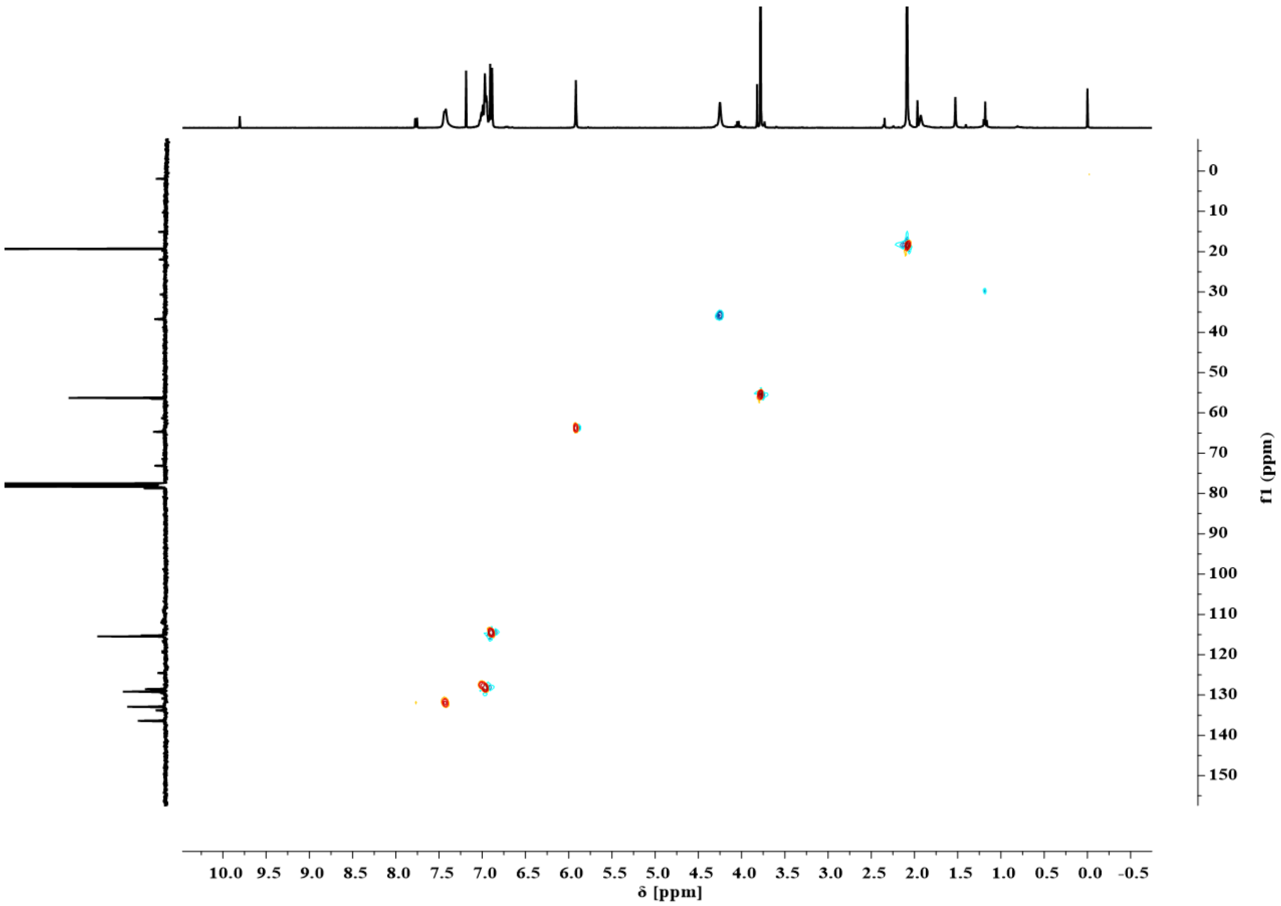

Supplementary Figure 80 | Multiplicity-edited HSQC experiment of the title compound recorded in $CDCl_3$.

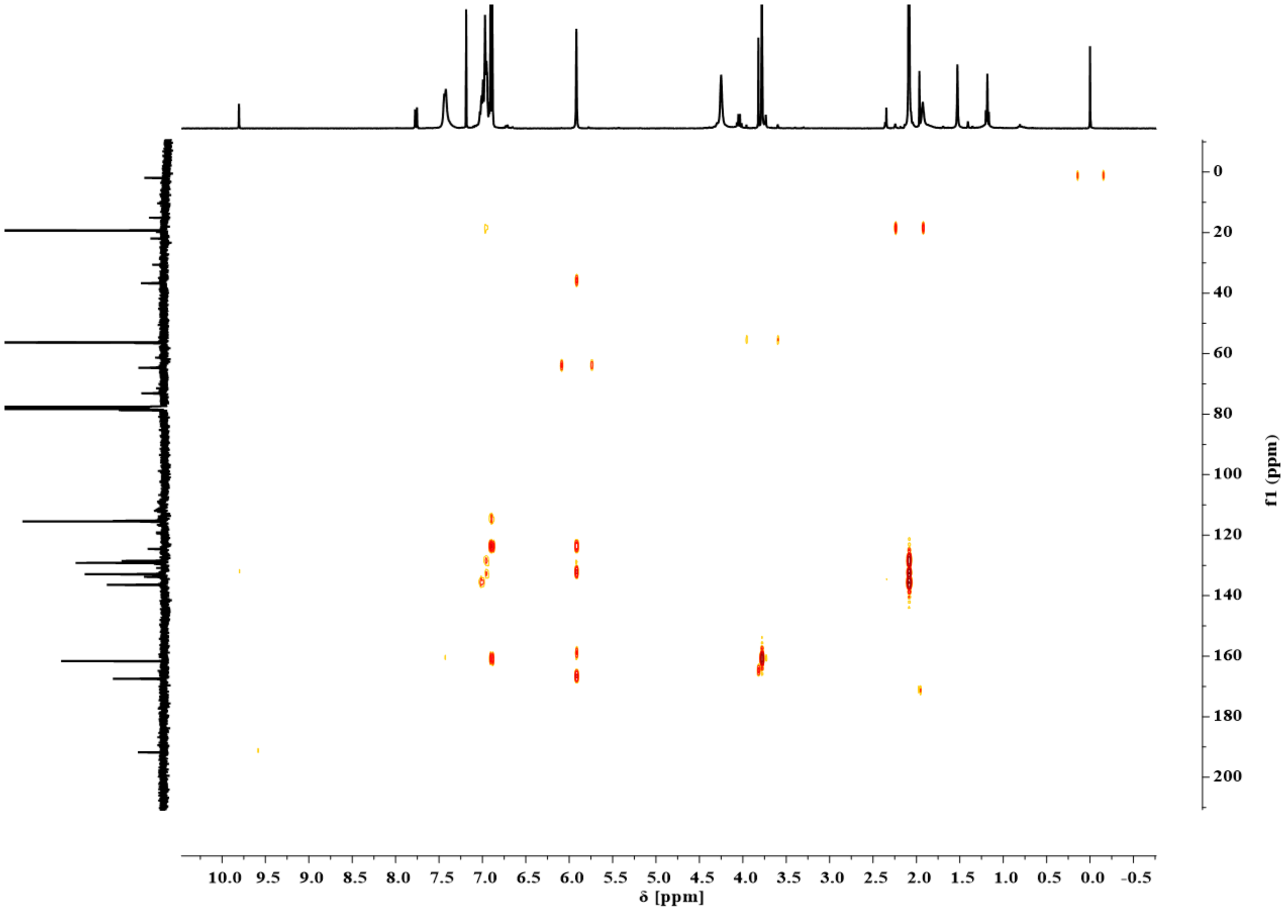

Supplementary Figure 81 | HMBC experiment of the title compound recorded in $CDCl_3$.

Ugi reaction of perfluorononanoic acid, *p*-anisaldehyde, cyclohexylisocyanide and heptylamine

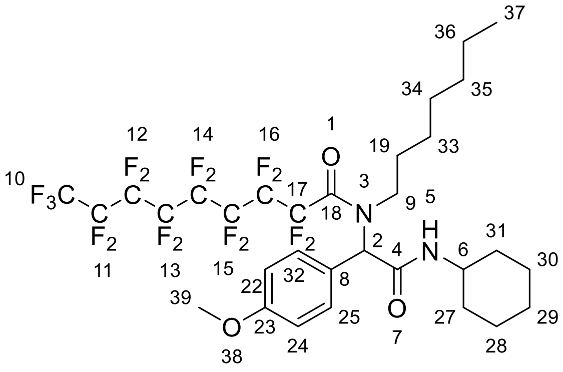

In a 25 mL round bottom flask *p*-anisaldehyde (77.9 µL, 87.3 mg, 641 µmol, 1.70 eq.) and heptylamine (95.0 µL, 73.8 mg, 641 µmol, 1.70 eq.) were mixed. The resulting mixture was stirred for 60 min over sodium sulfate. Perfluorononanoic acid (175 mg, 377 µmol, 1.00 eq.) dissolved in 0.5 mL methanol was added to the solution at room temperature and the resulting mixture was stirred for 2 min. Subsequently, cyclohexylisocyanide (79.9 µL, 70.0 mg, 641 µmol, 1.70 eq.) was added to the stirring mixture. The reaction was stirred for 3 d at room temperature. The crude reaction mixture was dried under reduced pressure and purified *via* column chromatography employing Fluoro*Flash*® silica gel. The fluorous fraction was tested for purity *via* TLC and concentrated under reduced pressure. The remaining perfluorononanoic acid was removed with a short silica gel filter column, eluting with *c*-hexane/ethyl acetate (3:1) to yield the Ugi product as a yellow oil (76.9 mg, 95.4 µmol, 25.3%).

R_f = 0.36 in *c*-hexane/ethyl acetate (4:1). Visualized *via* fluorescent quench and Seebach staining solution.

IR (ATR): ν [cm^{-1}] = 3295.4 (br, ν(N-H)), 2927.7 (m, ν(C-H)), 2853.7 (w, ν(C-H)), 1675.3 (s, ν(C=O)), 1645.9 (vs, ν(C=O)), 1612.2 (w), 1555.6 (m), 1513.0 (m), 1437.6 (w), 1200.7 (vs), 1144.6 (vs), 1028.7 (m), 977.1 (m), 918.3 (w), 822.1 (m), 773.1 (m), 703.4 (m), 662.7 (m), 561.6 (m), 527.0 (s), 443.0 (w).

^{1}H NMR (400 MHz, CDCl$_3$): δ [ppm] = 7.33 (d, J = 8.7 Hz, 2 H, $CH_{Ar}^{25,32}$), 6.91 (d, J = 8.8 Hz, 2 H, $CH_{Ar}^{22,24}$), 5.54 (s, 1 H, CH^{2}), 3.83 (s, 3 H, OCH_3^{39}), 3.81 – 3.75 (m, 1 H, CH^{6}), 3.38 (s, 2 H, CH_2^{9}), 1.94 – 1.84 (m, 2 H, CH_2), 1.69 – 1.55 (m, 4 H, CH_2), 1.38 – 0.94 (m, 16 H, CH_2), 0.83 (t, J = 7.2 Hz, 3 H, CH_3^{37}).

^{13}C NMR (101 MHz, CDCl$_3$): δ [ppm] = 167.4 (s, $CONR^{4}$), 160.3 (s, C_{Ar}^{23}), 159.0 (s, $CONR^{10}$), 131.3 (s, $CH_{Ar}^{25,32}$), 125.5 (s, C_{Ar}^{8}), 114.6 (s, $C_{Ar}^{22,24}$), 64.8 (s, CH^{2}), 55.5 (s, OCH_3^{39}), 48.9 (s, CH^{6}), 47.5

(s, CH_2^{9}), 32.8 (s, CH_2), 31.7 (s, CH_2), 30.0 (s, CH_2), 28.6 (s, CH_2), 27.1 (s, CH_2), 26.7 (s, CH_2), 25.6 (s, CH_2), 24.9 (s, CH_2), 22.6 (s, CH_2), 14.1 (s, CH_3^{37}).

^{19}F NMR (376 MHz, $CDCl_3$): δ [ppm] = -85.11 (t, J = 9.9 Hz, 3 F, CF_3^{10}), AB-signal (δ_A = -112.88, δ_B = -114.15, J_{AB} = 295.5 Hz, A and B are split into t, J = 13.3 Hz, CF_2^{17a}), AB-signal (δ_A = -115.47, δ_B = -116.63, J_{AB} = 291.8 Hz, A and B are split into t, J = 13.7 Hz, CF_2^{17b}), -123.87 – 125.00 (m, CF_2), -126.13 (s, CF_2), -127.06 (s, CF_2), -130.45 (s, CF_2^{11}). Total integral of CF_2 region normalized with respect to the CF_3^{10} group = 14.

FAB – MS [m/z] (relative intensity): 807.3 (25%) $[M + H]^+$, 708.1 (23%) [Fragment A +H $]^+$, 681.2 [Fragment B +H $]^+$.

HRMS – FAB [m/z]: $[M + H]^+$ calculated for $^{12}C_{31}{}^{1}H_{36}{}^{16}O_3{}^{14}N_2{}^{19}F_{17}$, 807.2449; found, 807.2449; Δ = 0.03 mmu.

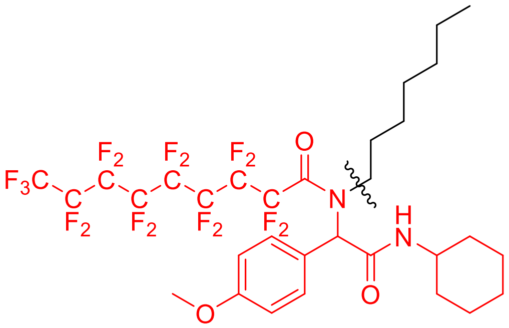

Chemical Formula: $C_{24}H_{20}F_{17}N_2O_3^{\bullet}$
Exact Mass: 707,12025
Fragment A

Chemical Formula: $C_{24}H_{23}F_{17}NO_2^{\bullet}$
Exact Mass: 680,14573
Fragment B

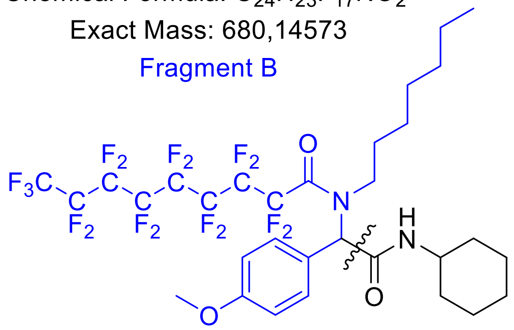

Supplementary Figure 82 | Proposed fragments observed in FAB-MS.

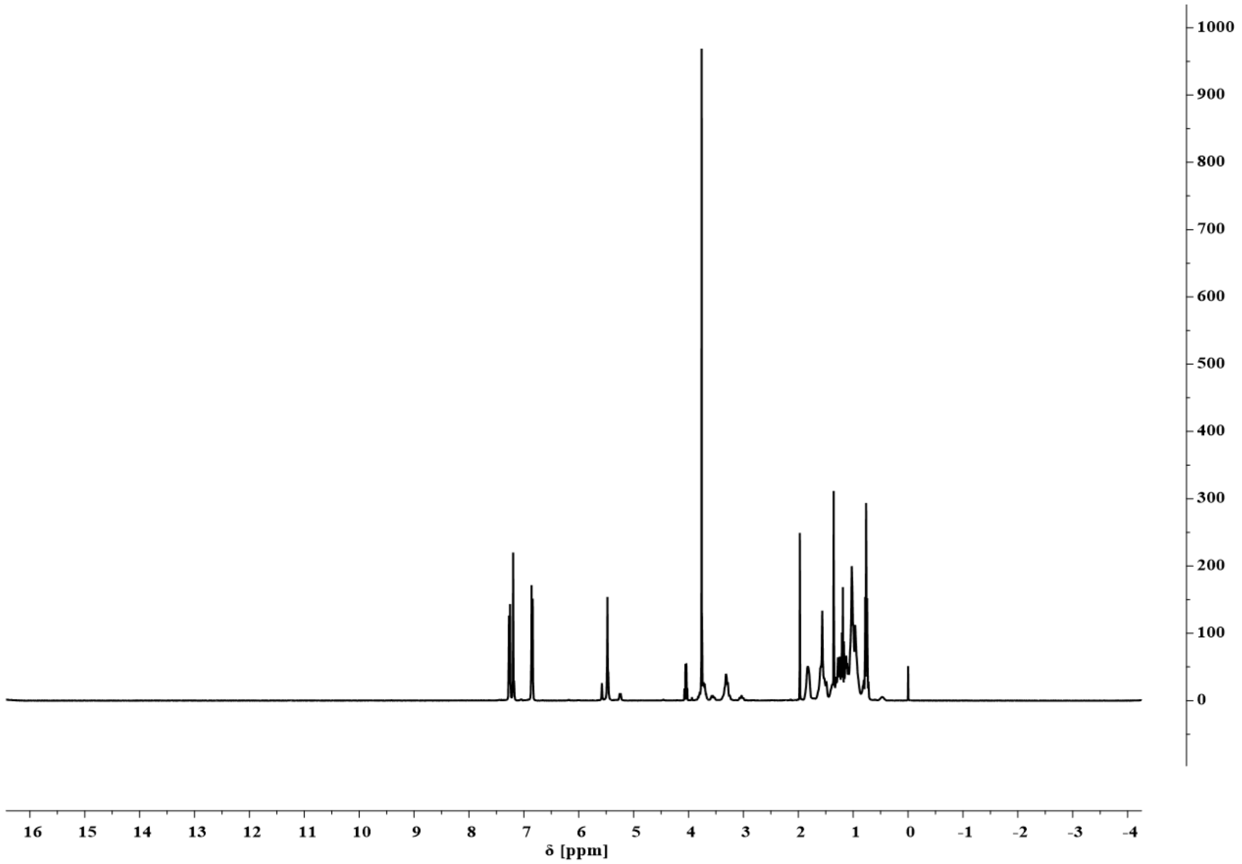

Supplementary Figure 83 | ^{1}H NMR of the title compound recorded in $CDCl_3$.

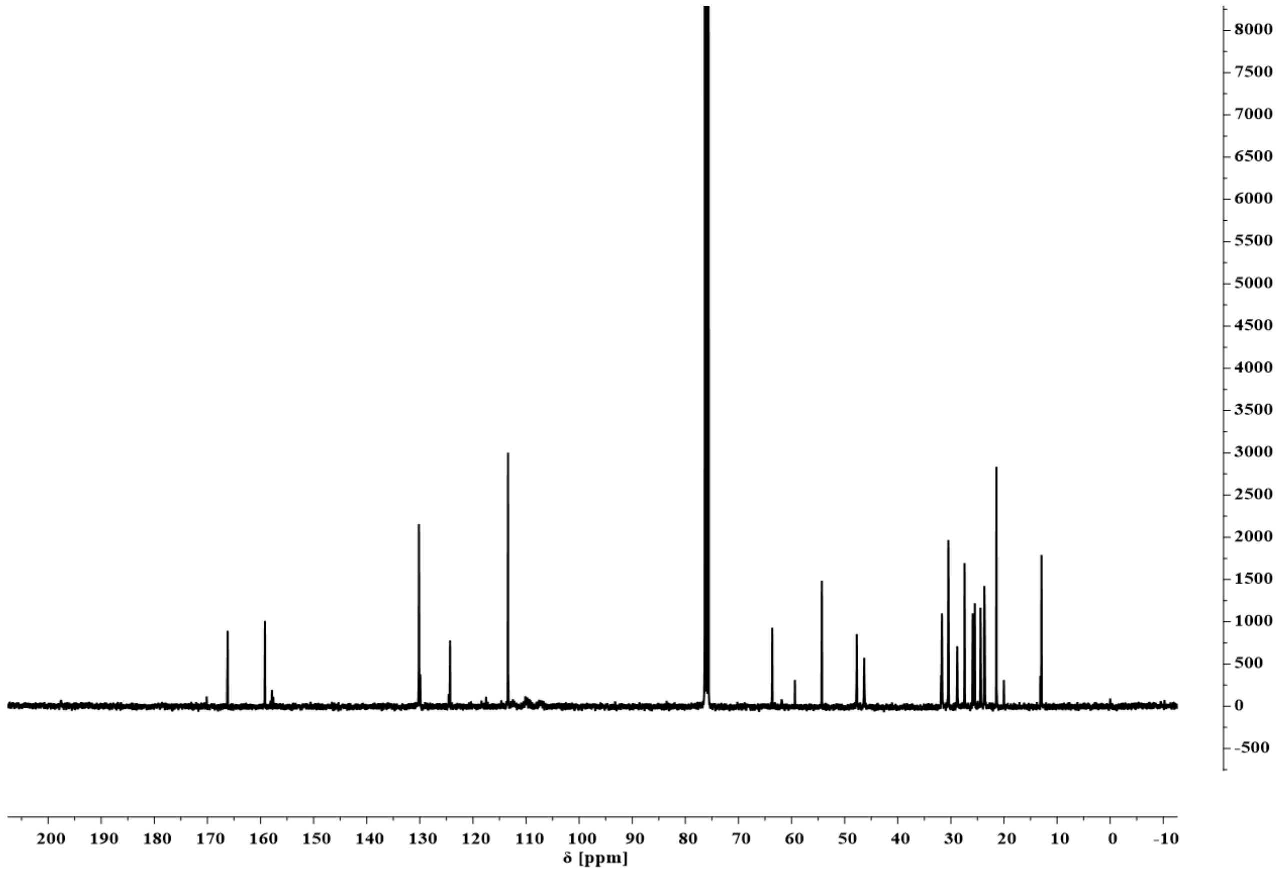

Supplementary Figure 84 | ^{13}C NMR of the title compound recorded in $CDCl_3$.

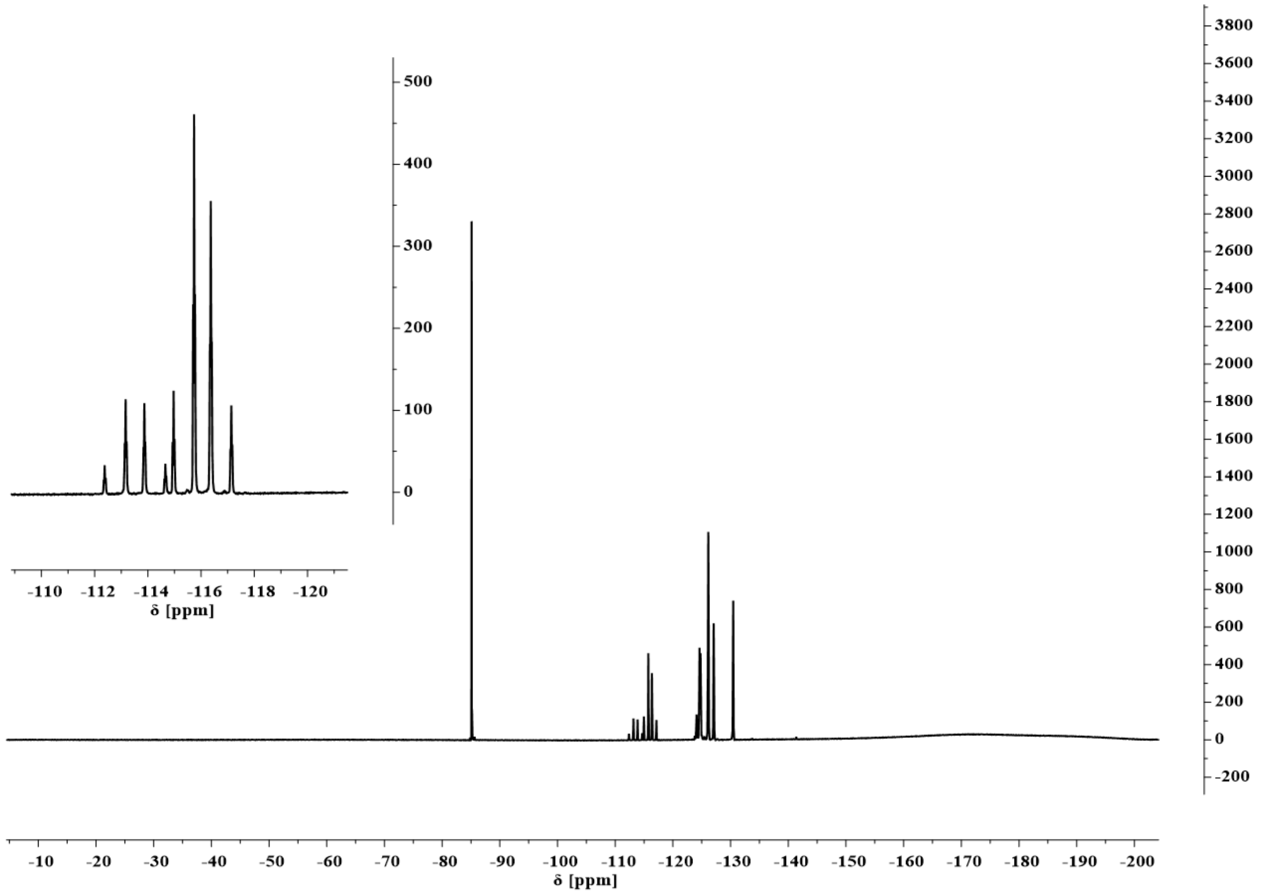

Supplementary Figure 85 | ^{19}F NMR of the title compound recorded in $CDCl_3$.

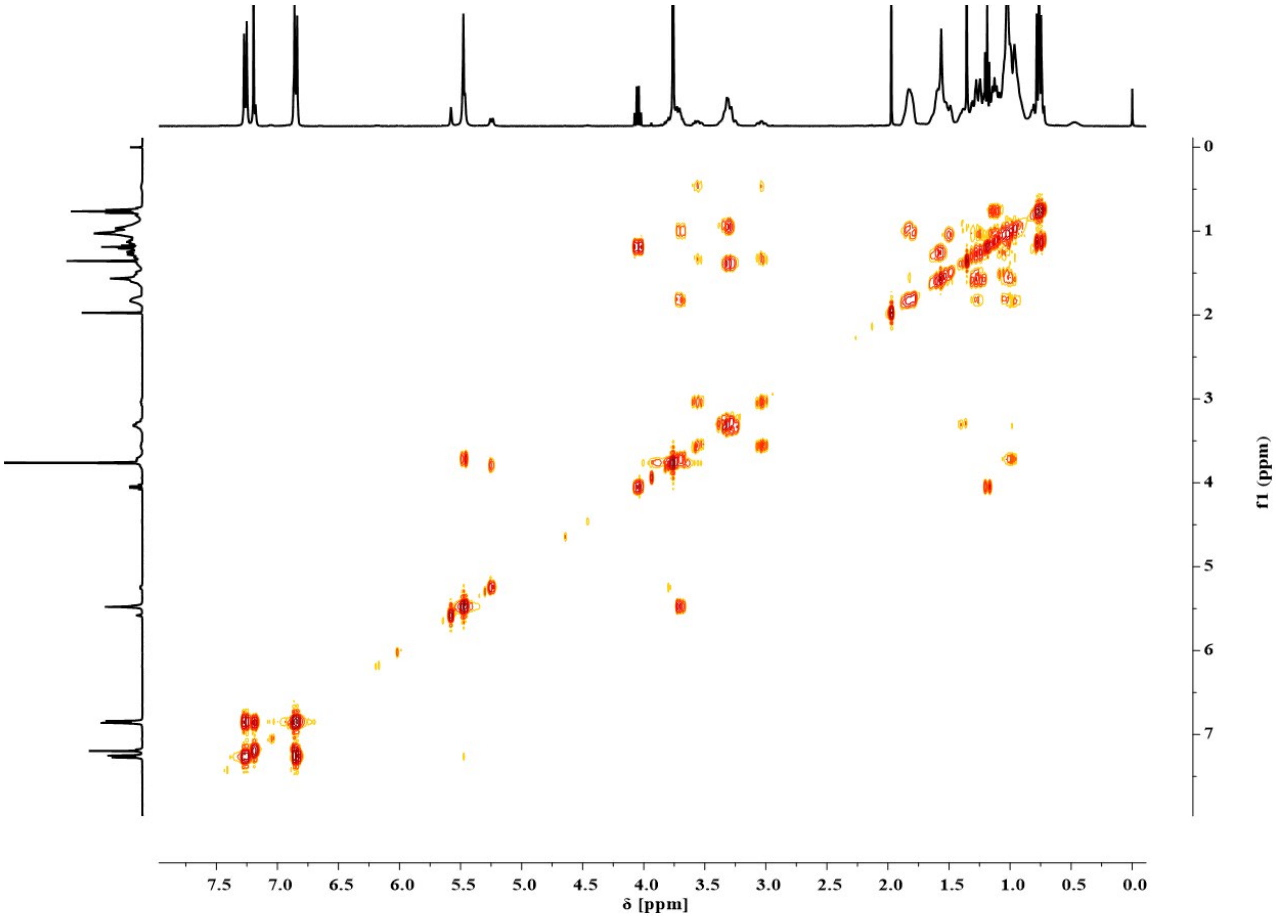

Supplementary Figure 86 | COSY experiment of the title compound recorded in $CDCl_3$.

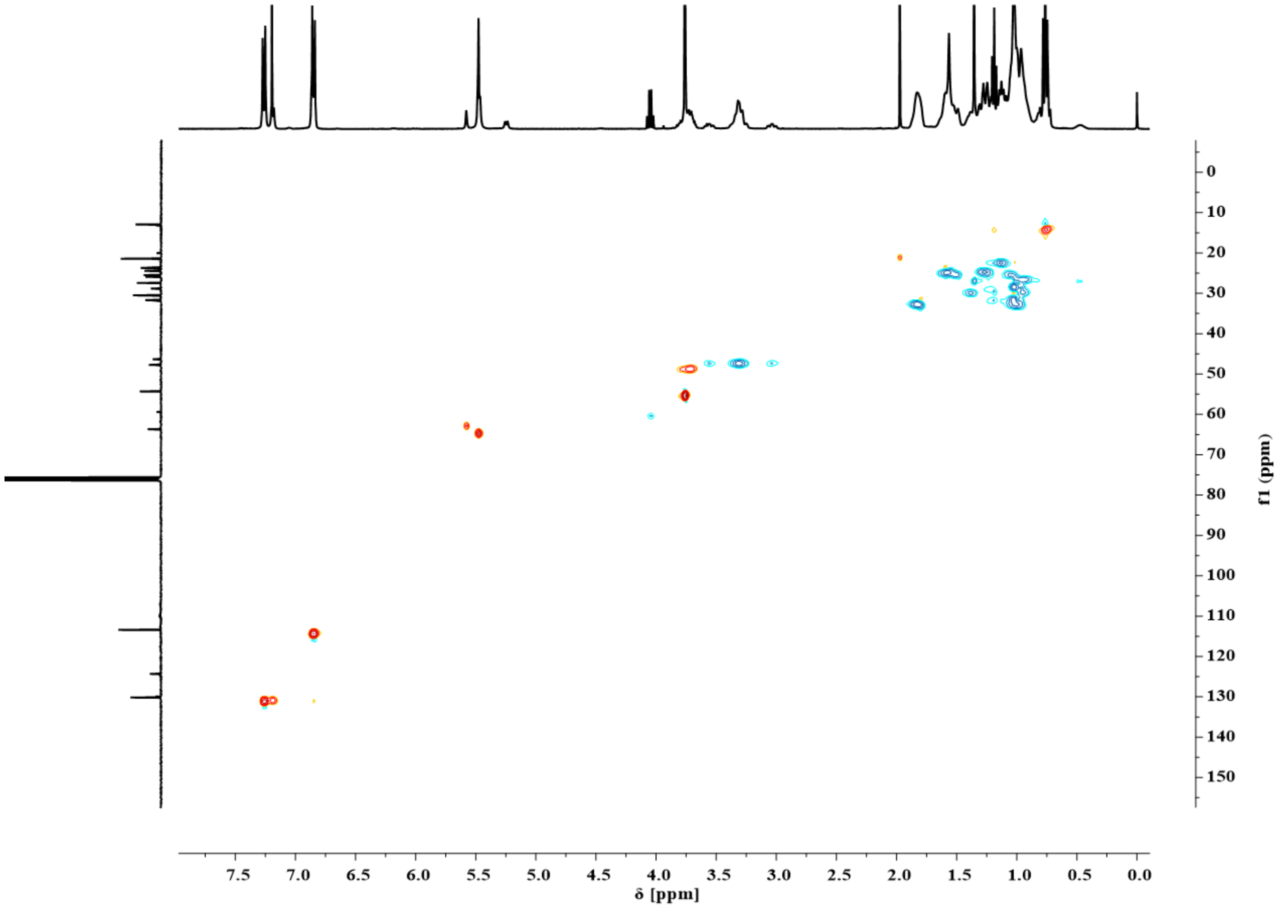

Supplementary Figure 87 | Multiplicity-edited HSQC experiment of the title compound recorded in $CDCl_3$.

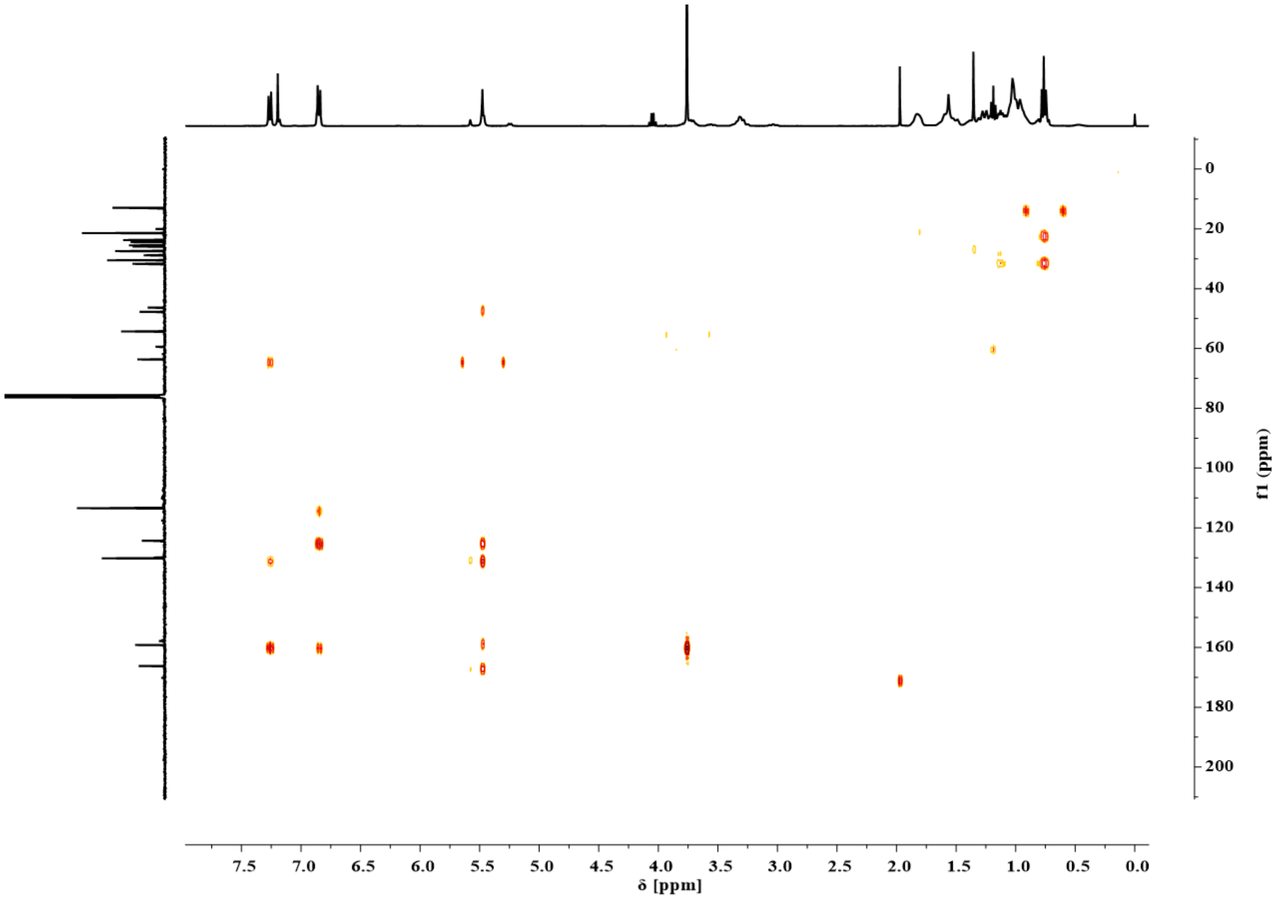

Supplementary Figure 88 | HMBC experiment of the title compound recorded in $CDCl_3$.

Ugi reaction of perfluorononanoic acid, *p*-anisaldehyde, ethyl-2-isocyanoacetate and propargylamine

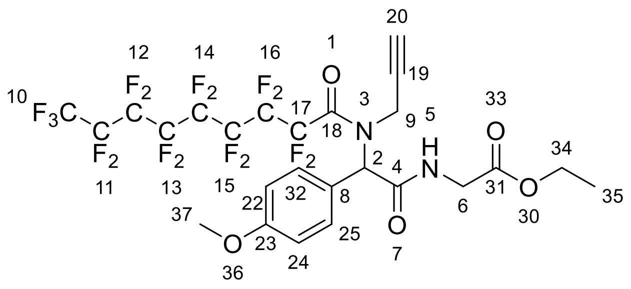

In a 25 mL round bottom flask *p*-anisaldehyde (77.9 µL, 87.3 mg, 641 µmol, 1.70 eq.) and propargylamine (41.1 µL, 35.3 mg, 641 µmol, 1.70 eq.) were added. The resulting mixture was stirred for 60 min over sodium sulfate. Perfluorononanoic acid (175 mg, 377 µmol, 1.00 eq.) dissolved in 0.5 mL methanol was added to the solution at room temperature and the resulting mixture was stirred for 2 min. Subsequently, ethyl-2-isocyanoacetate (82.6 µL, 72.5 mg, 641 µmol, 1.70 eq.) was added to the stirring mixture. The reaction was stirred for 3 d at room temperature. The crude reaction mixture was dried under reduced pressure and purified *via* column chromatography employing Fluoro*Flash*® silica gel. The fluorous fraction was tested for purity *via* TLC and concentrated under reduced pressure. The remaining perfluorononanoic acid was removed with a short silica gel filter column, eluting with *c*-hexane/ethyl acetate (3:1) to yield the Ugi product as a yellow oil (65.8 mg, 87.8 µmol, 23.3%).

R_f = 0.30 in *c*-hexane/ethyl acetate (4:1). Visualized *via* fluorescent quench and Seebach staining solution.

IR (ATR): ν[cm^{-1}] = 3269.5 (m, ν(N-H)), 2924.6 (w, ν(C-H)), 1746.7 (s, ν(C=O)), 1691.8 (s), 1666.2 (s), 1613.2 (w), 1563.9 (m), 1514.8 (m), 1449.1 (m), 1412.8 (m), 1200.9 (vs), 1145.4 (vs), 1106.1 (s), 1036.6 (m), 1004.3 (m), 950.1 (m), 828.4 (m), 768.2 (m), 702.4 (s), 672.2 (s), 636.9 (s), 558.2 (s), 529.1 (s), 430.2 (w), 390.0 (w).

^{1}H NMR (400 MHz, CDCl$_3$): δ [ppm] = 7.39 (d, J = 8.2 Hz, 2 H, $CH_{Ar}^{25,32}$), 6.93 (d, J = 8.7 Hz, 2 H, $CH_{Ar}^{22,24}$), 5.92 (s, 1 H, CH^{2}), 4.46 – 3.96 (m, 6 H, $CH_2^{34,6,9}$), 3.89 (s, 1 H, CH^{20}), 3.83 (s, 3 H, CH_3^{37}), 1.42 – 1.09 (m, 3 H, CH_3^{35}).

^{13}C NMR (101 MHz, CDCl$_3$): δ [ppm] = 169.4 (s, $CONR^{4}$), 164.8 (s, $CONR^{18}$), 160.7 (s, C_{Ar}^{23}), 132.1 (s, $CH_{Ar}^{25,32}$), 129.6 (s, C_{Ar}^{8}), 114.7 (s, $C_{Ar}^{22,24}$), 61.8 (s, CH^{2}), 55.7 (s, CH^{20}), 55.5 (s, OCH_3^{37}), 41.8 (s, CH_2^{6}), 36.0 (s, CH_2^{9}), 14.2 (CH_3^{35}).

^{19}F NMR (376 MHz, CDCl$_3$): δ [ppm] = -85.10 (t, J = 9.9 Hz, 3 F, CF$_3$^{10}), AB-signal (δ_A = -113.26, δ_B = -114.63, J_{AB} = 297.4 Hz, additional coupling not resolved, signals broadened, CF$_2$^{17a}), AB-signal (δ_A = -115.22, δ_B = -116.19, J_{AB} = 291.8 Hz, additional coupling not resolved, signals broadened, CF$_2$^{17b}), -124.84 (s, CF$_2$), -126.12 (s, CF$_2$), -127.05 (s, CF$_2$), -130.44 (s, CF$_2$^{11}). Total integral of CF$_2$ region normalized with respect to the CF$_3$^{10} group = 14.

FAB – MS [m/z] (relative intensity): 750.1 (90%) [M]$^+$, 620.1 (17%) [Fragment A]$^+$, 250.1 (33%) [Fragment B]$^+$, 120.1 [Fragment C]$^+$.

HRMS – FAB [m/z]: [M]$^+$ calculated for $^{12}C_{29}{}^{1}H_{19}{}^{16}O_5{}^{14}N_2{}^{19}F_{17}$, 750.1017; found, 750.1018; Δ = 0.13 mmu.

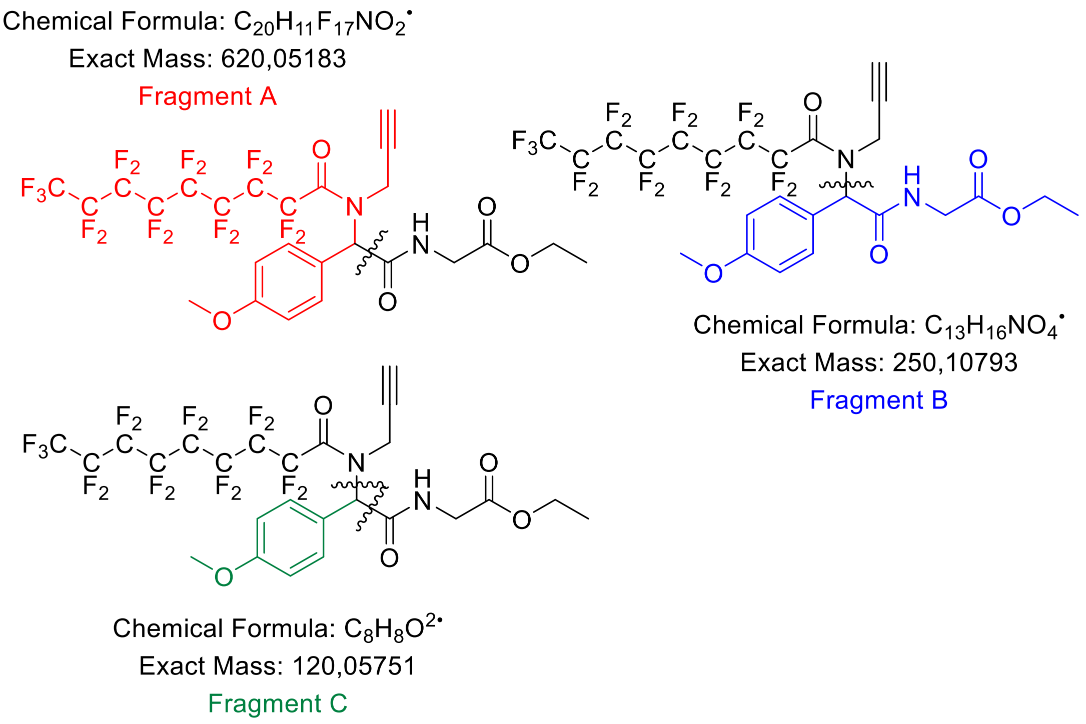

Supplementary Figure 89 | Proposed fragments observed in FAB-MS.

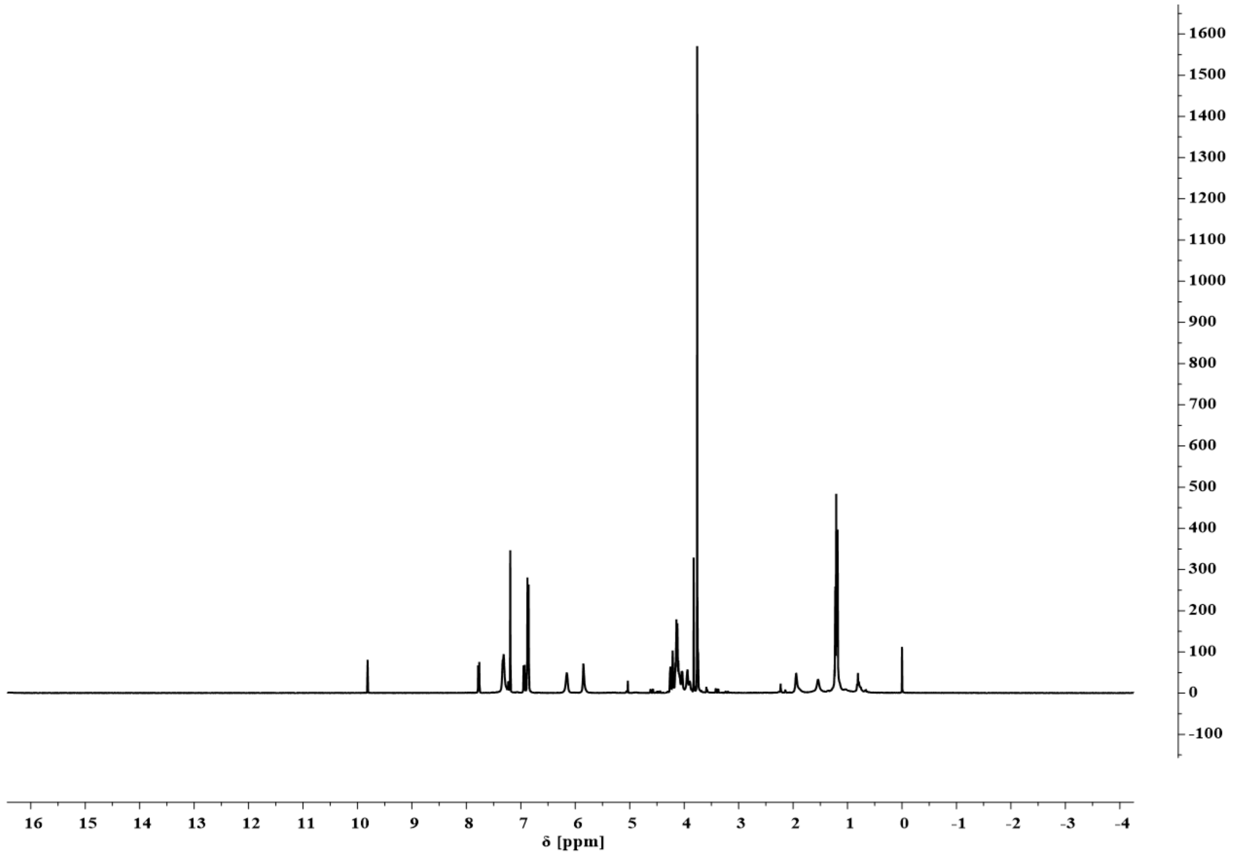

Supplementary Figure 90 | ^{1}H NMR experiment of the title compound recorded in $CDCl_3$.

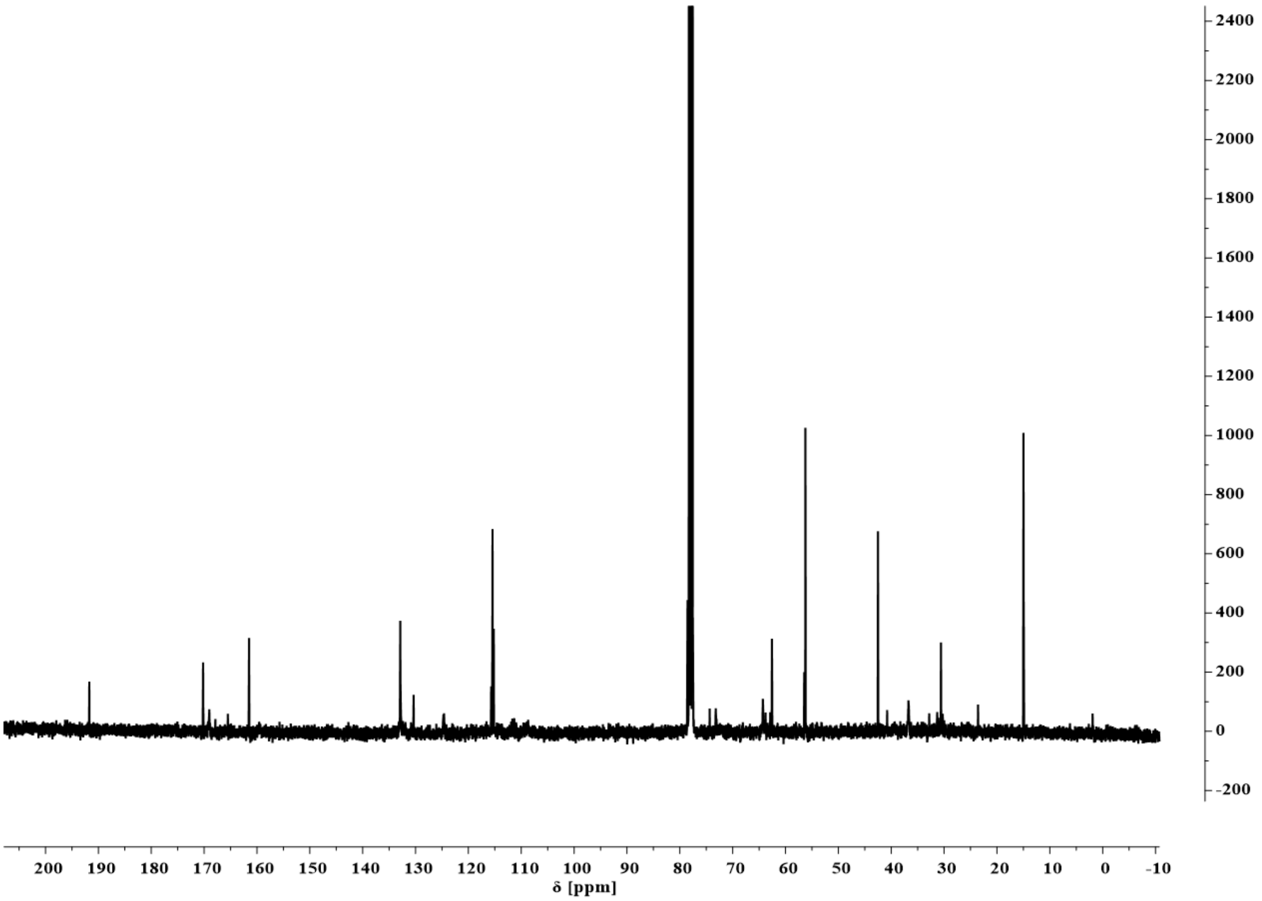

Supplementary Figure 91 | ^{13}C NMR experiment of the title compound recorded in $CDCl_3$.

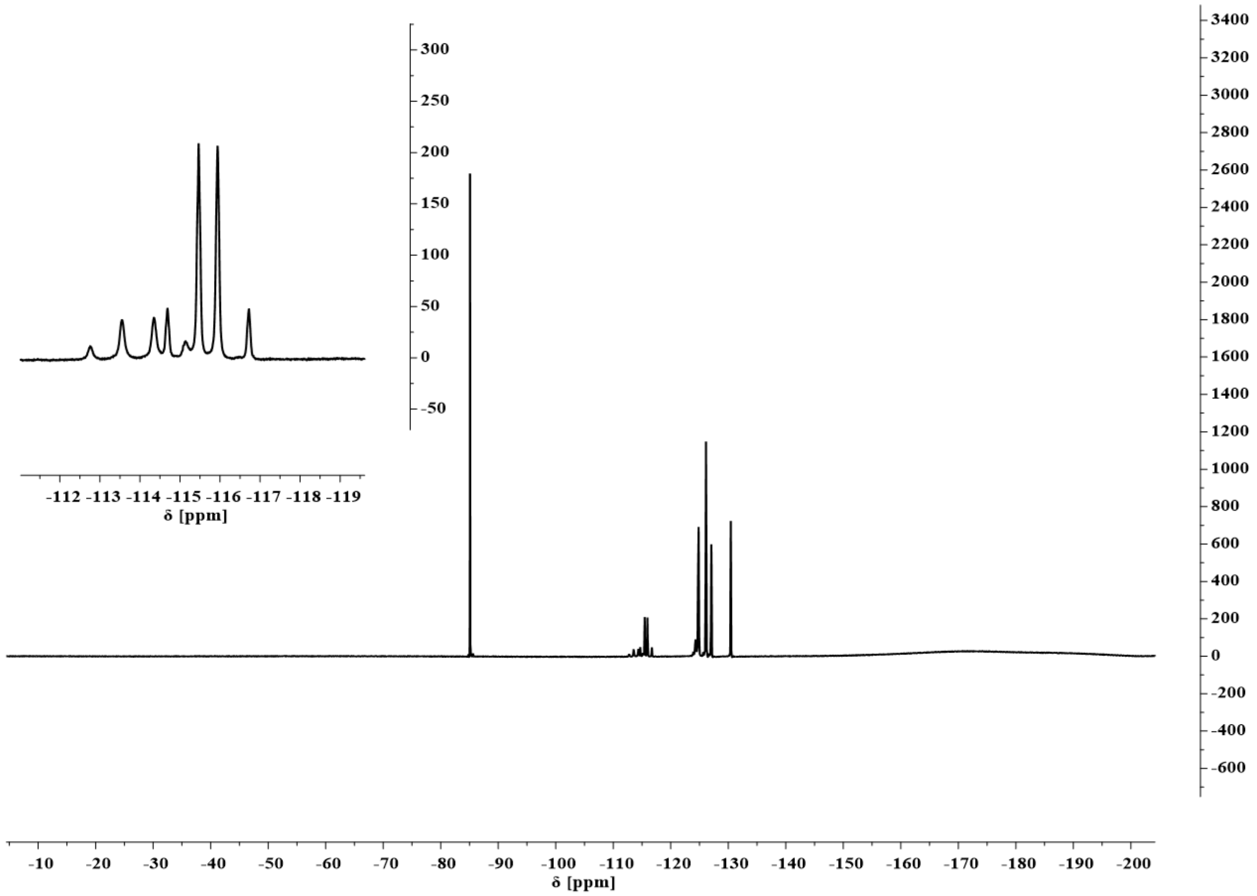

Supplementary Figure 92 | ^{19}F NMR experiment of the title compound recorded in $CDCl_3$.

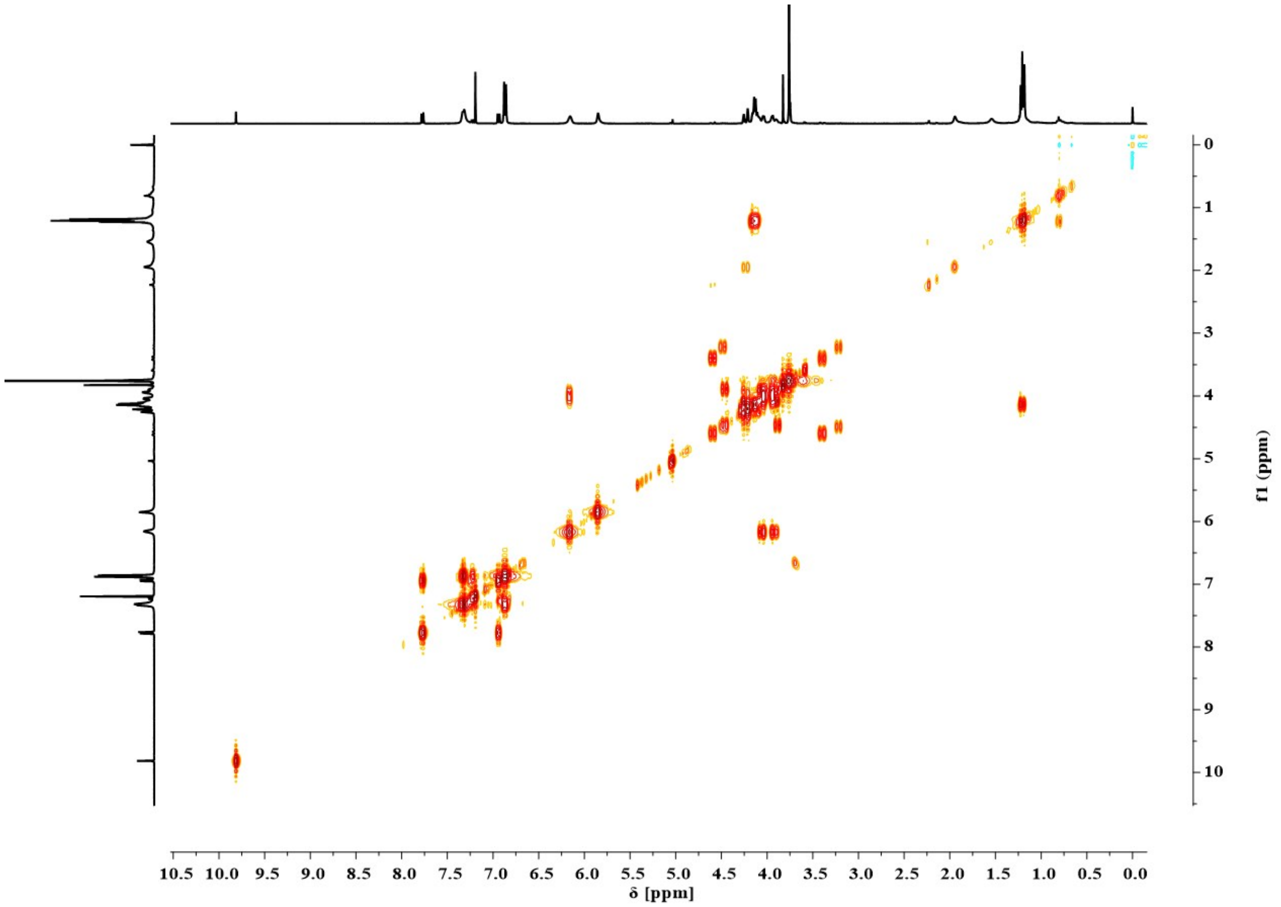

Supplementary Figure 93 | COSY experiment of the title compound recorded in $CDCl_3$.

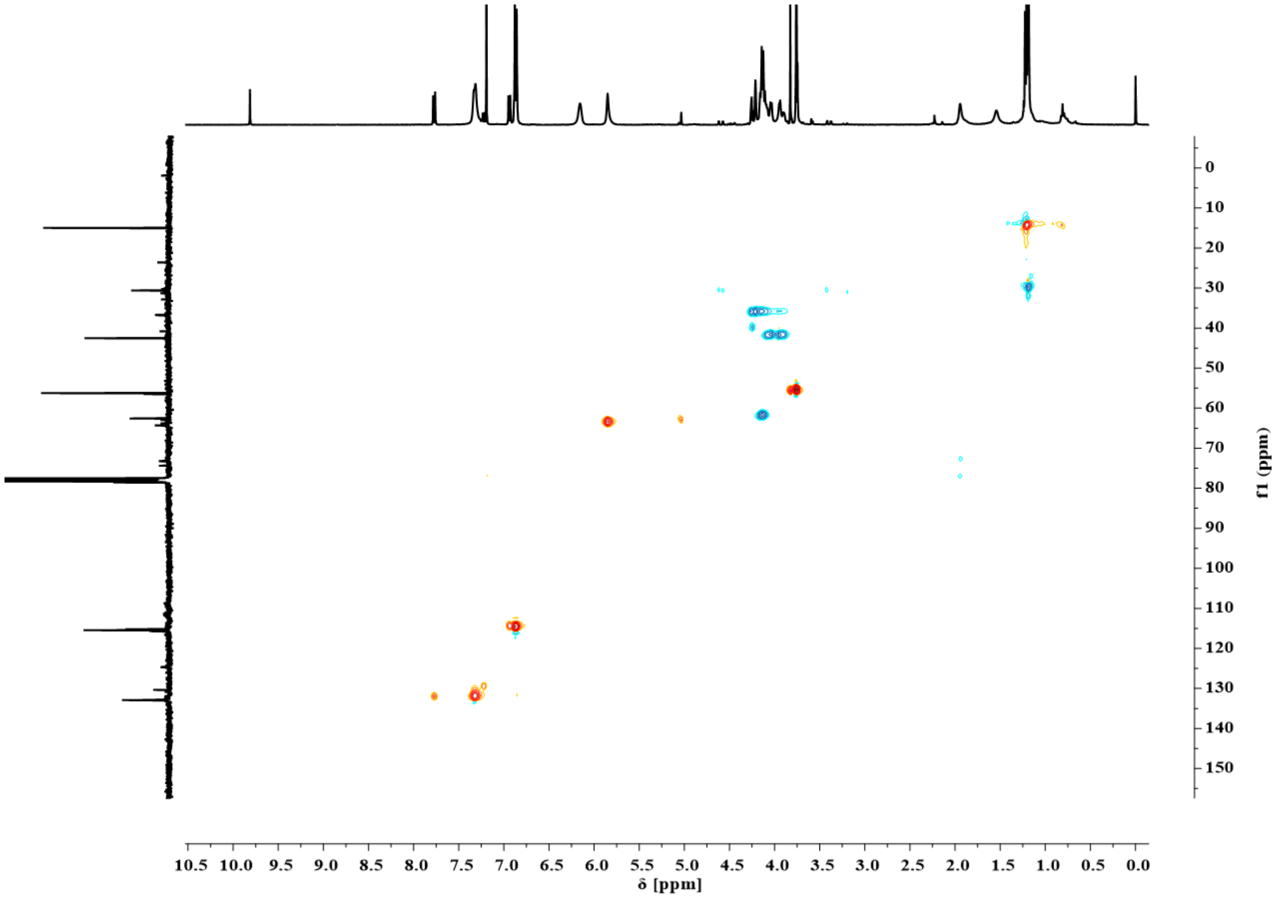

Supplementary Figure 94 | Multiplicity-edited HSQC experiment of the title compound recorded in $CDCl_3$.

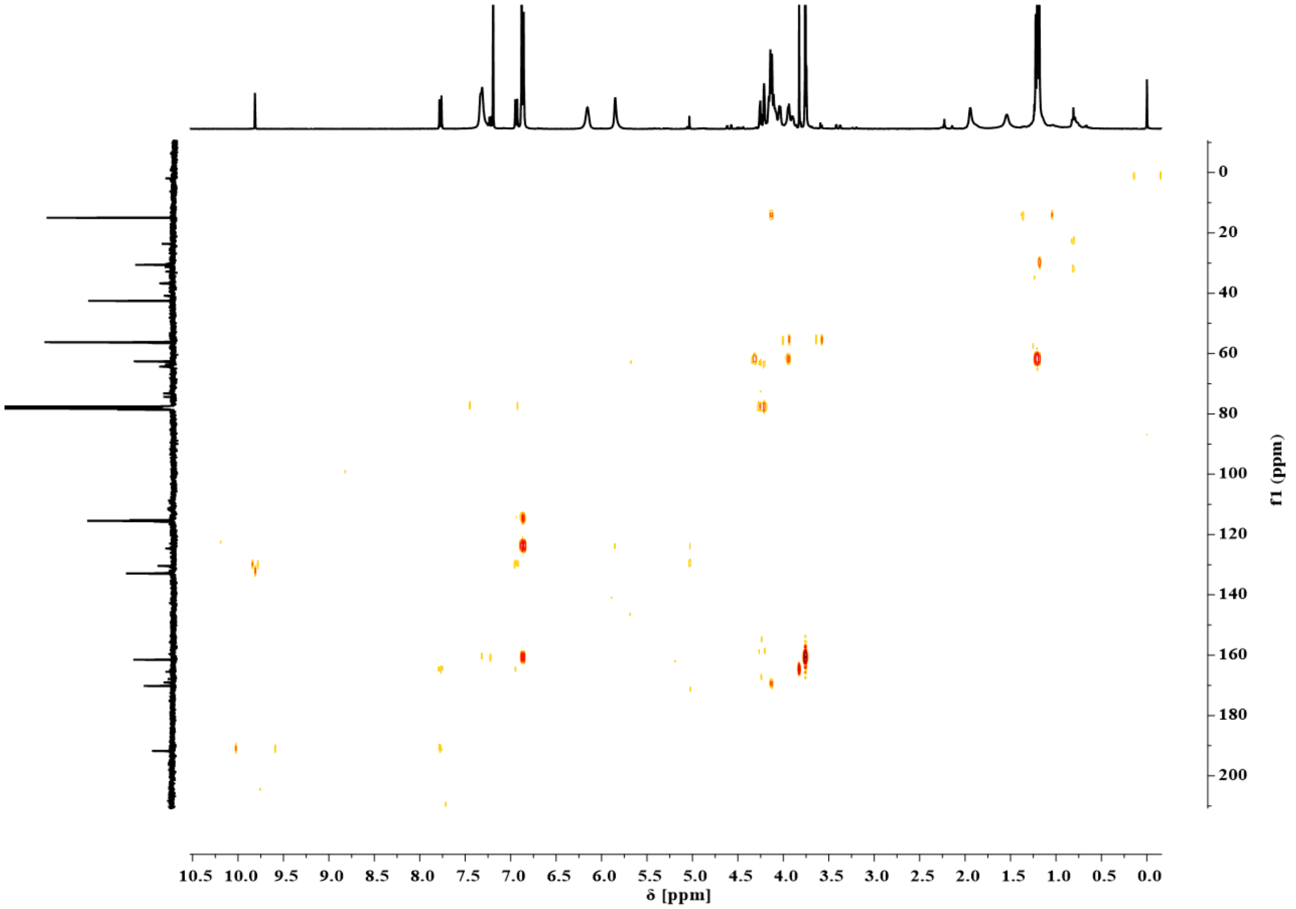

Supplementary Figure 95 | HMBC experiment of the title compound recorded in $CDCl_3$.

Ugi reaction of perfluorononanoic acid, valeraldehyde, *tert*-butylisocyanide and pentylamine

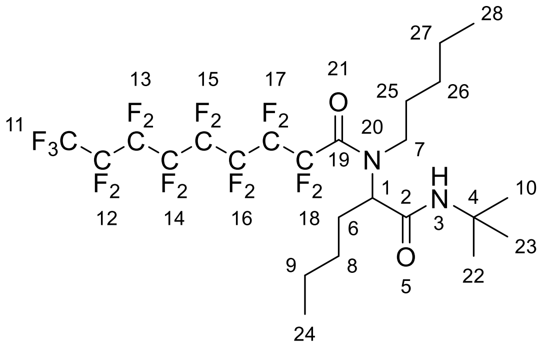

In a 25 mL round bottom flask valeraldehyde (42.2 mg, 490 µmol, 1.30 eq.) was dissolved in 1.5 mL methanol, subsequently pentylamine (56.6 µL, 42.7 mg, 490 µmol, 1.30 eq.) was added and the resulting mixture was stirred for 60 min over sodium sulfate. Afterwards, the mixture was filtrated. The solid was washed with 10 mL methanol three times. Subsequently, the filtrate was concentrated under reduced pressure. Perfluorononanoic acid (175 mg, 377 µmol, 1.00 eq.) dissolved in 1 mL methanol was added to the imine at room temperature and the resulting mixture was stirred for 2 min. Subsequently, *tert*-butylisocyanide (51.2 µL, 37.6 mg, 453 µmol, 1.20 eq.) was added to the stirring mixture. The reaction was stirred for 3 d at room temperature. The crude reaction mixture was dried under reduced pressure and purified *via* column chromatography employing Fluoro*Flash*® silica gel. The fluorous fraction was concentrated and the residue was adsorbed onto celite® and purified *via* column chromatography employing silica gel as stationary phase and eluting with a gradual solvent mixture of ethyl acetate and *c*-hexane (0:1 → 1:1) to yield the Ugi product as a highly viscous yellow oil (42.7 mg, 60.3 µmol, 16.0%).

R_f = 0.49 in *c*-hexane/ethyl acetate (6:1). Visualized *via* Seebach staining solution and permanganate staining.

IR (ATR): ν [cm^{-1}] = 3367.2 (br, ν(N-H)), 2962.9 (m, ν(C-H)), 2875.3 (w), 1661.9 (s, ν(C=O)), 1532.8 (m), 1456.6 (m), 1366.2 (m), 1204.6 (vs), 1147.5 (vs), 996.7 (w), 778.1 (m), 735.4 (m), 703.2 (m), 656.5 (m), 558.6 (m), 528.9 (m).

^{1}H NMR (400 MHz, CD_3OD): δ [ppm] = 7.43 (s, 1 H, NH^{3}), 4.57 (t, J = 7.6 Hz, 1 H, CH^{1}), 3.58 – 3.37 (m, 2 H, CH_2^{7}), 2.04 – 1.85 (m, 2 H, CH_2^{6}), 1.79 – 1.57 (m, 2 H, CH_2^{25}), 1.40 – 1.19 (m, 17 H, $CH_3^{10,22,23}$ + CH_2), 0.92 (t, J = 7.2 Hz, 6 H, $CH_3^{24,28}$).

^{13}C NMR (101 MHz, CD_3OD): δ [ppm] = 170.6 (s, CONR^{2}), 159.7 (s, CONR^{19}), 62.4 (s, CH^{1}), 52.4 (s, C^{4}), 47.1 (s, CH_2^{7}), 32.1 (s, CH_2^{25}), 31.5 (s, CH_2), 30.4 (s, CH_2), 30.0 (s, CH_2), 29.7 (s, CH_2^{6}), 29.4 (s, CH_2), 28.7 (s, $CH_3^{22,23,10}$), 23.2 (s, CH_2), 14.3 (s, $CH_3^{24\ or\ 28}$), 14.2 (s, $CH_3^{24\ or\ 28}$).

^{19}F NMR (376 MHz, CD_3OD): δ [ppm] = -88.27 (t, J = 10.3 Hz, 3 F, CF_3^{11}), AB-signal (δ_A = -116.39, δ_B = -117.10, J_{AB} = 288.0 Hz, A and B are split into t, J = 11.7 Hz, CF_2^{18a}), AB-signal (δ_A = -118.10, δ_B = -118.55, J_{AB} = 291.8 Hz, A and B are split into t, J = 12.5 Hz, CF_2^{18b}), -126.95 (s, CF_2), -128.68 (s, CF_2), -129.65 (s, CF_2), -133.16 (s, CF_2^{12}). Total integral of CF_2 region normalized with respect to the CF_3^{11} group = 14.

FAB – MS [*m/z*] (relative intensity): 703.2 (65%) $[M + H]^+$, 630.1 (25%) [Fragment A – H]$^+$, 560.5 (28%) [Fragment A – C_5H_{11}]$^+$.

HRMS – FAB [*m/z*]: $[M + H]^+$ calculated for $^{12}C_{24}{}^{1}H_{31}{}^{16}O_2{}^{14}N_2{}^{19}F_{17}$, 703.2187; found, 703.2188; Δ = 0.13 mmu.

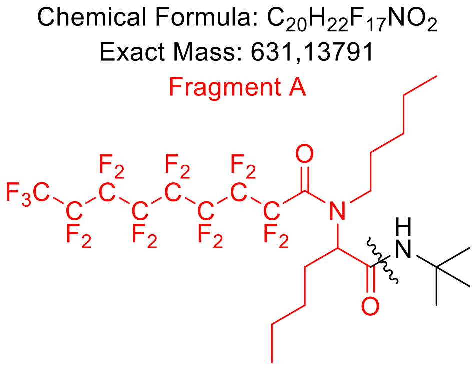

Supplementary Figure 96 | Proposed fragments observed in FAB-MS.

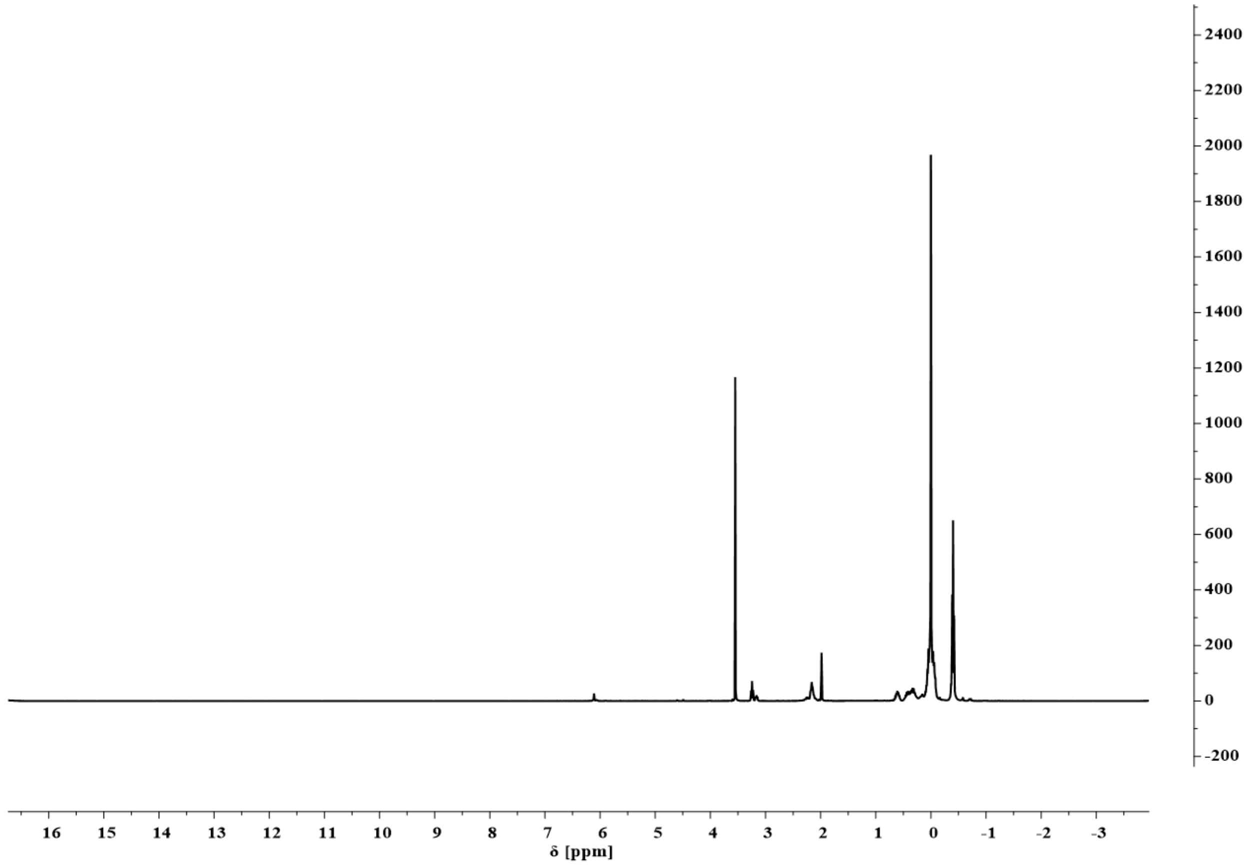

Supplementary Figure 97 | ^{1}H NMR experiment of the title compound recorded in $CDCl_3$.

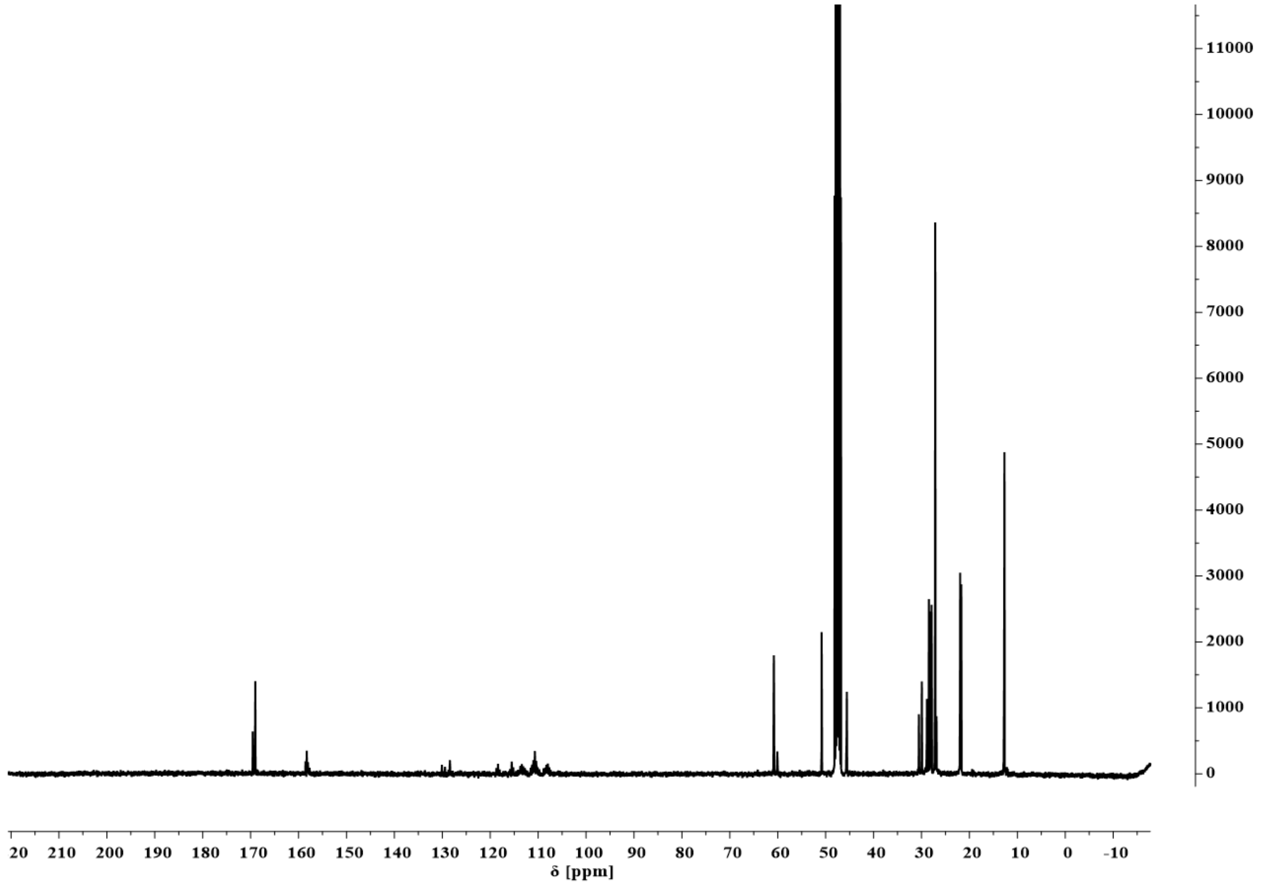

Supplementary Figure 98 | ^{13}C NMR experiment of the title compound recorded in $CDCl_3$.

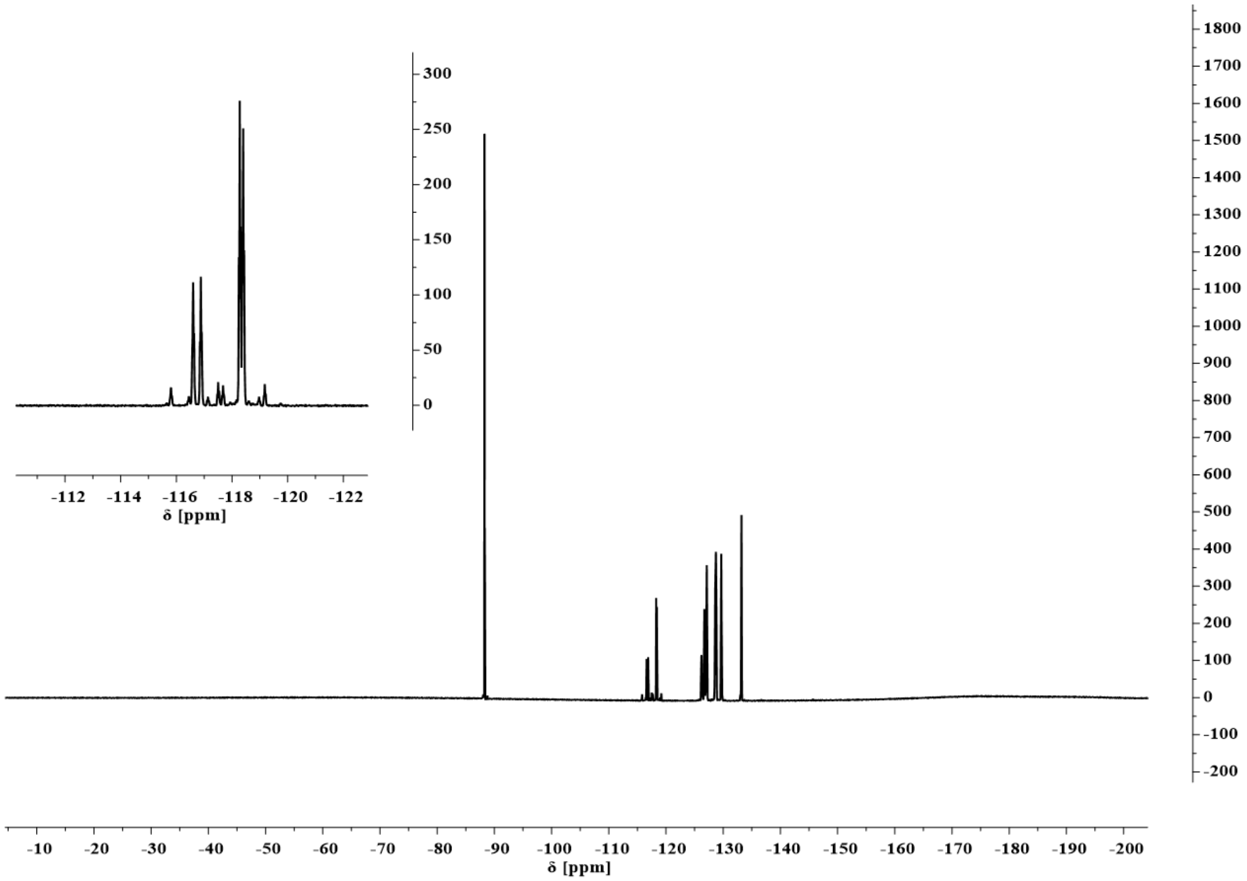

Supplementary Figure 99 | ^{19}F NMR experiment of the title compound recorded in $CDCl_3$.

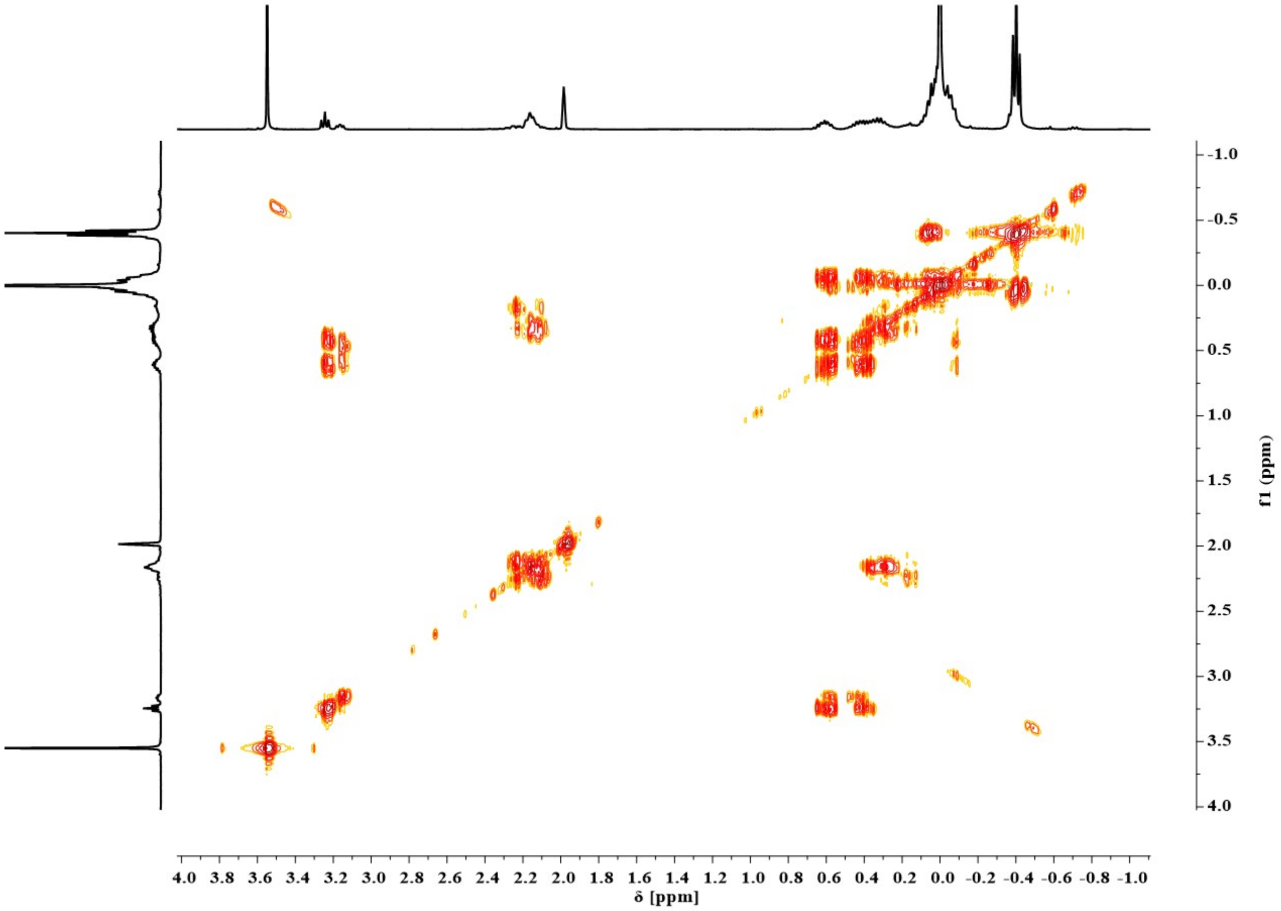

Supplementary Figure 100 | COSY experiment of the title compound recorded in $CDCl_3$.

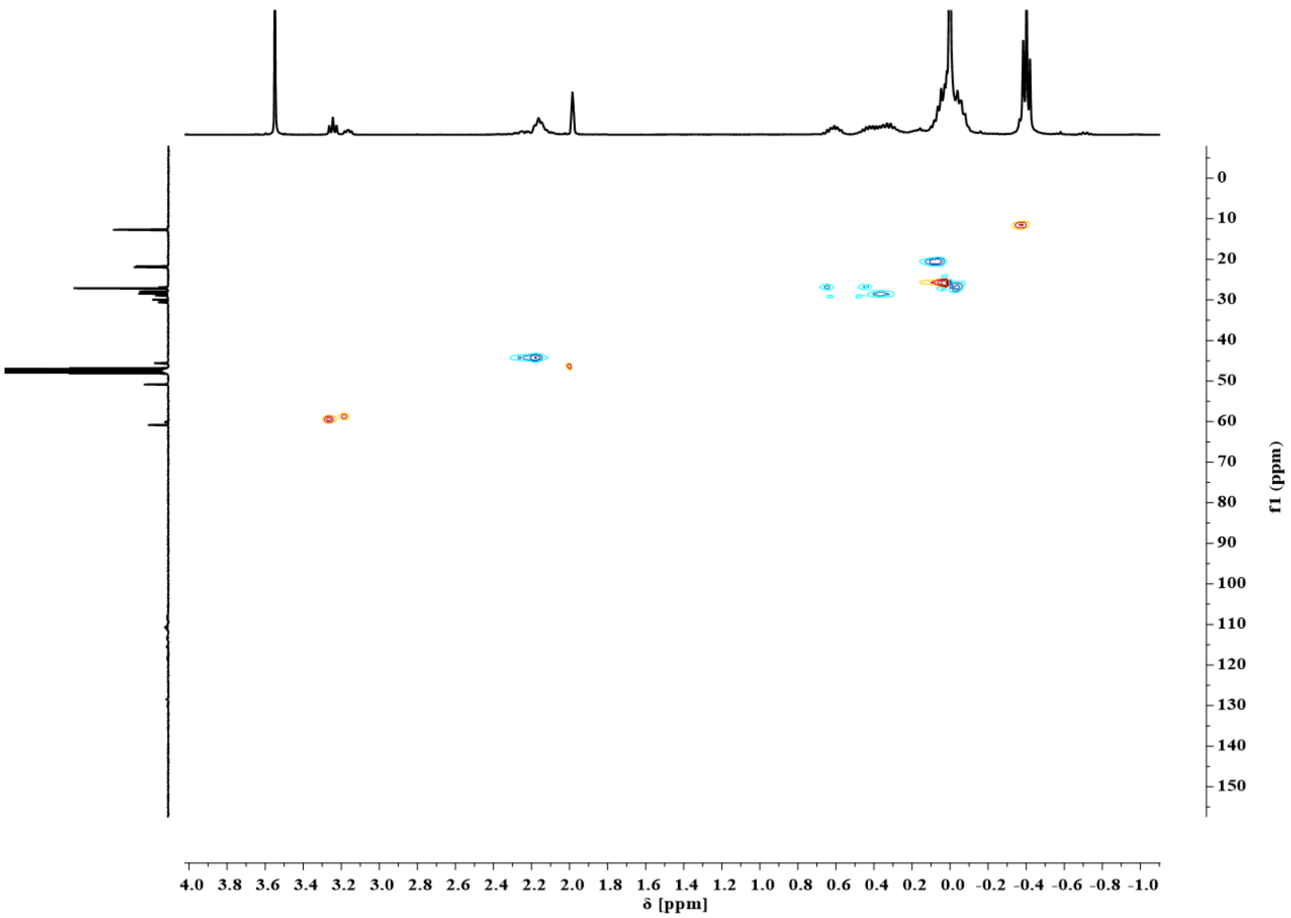

Supplementary Figure 101 | Multiplicity-edited HSQC experiment of the title compound recorded in $CDCl_3$.

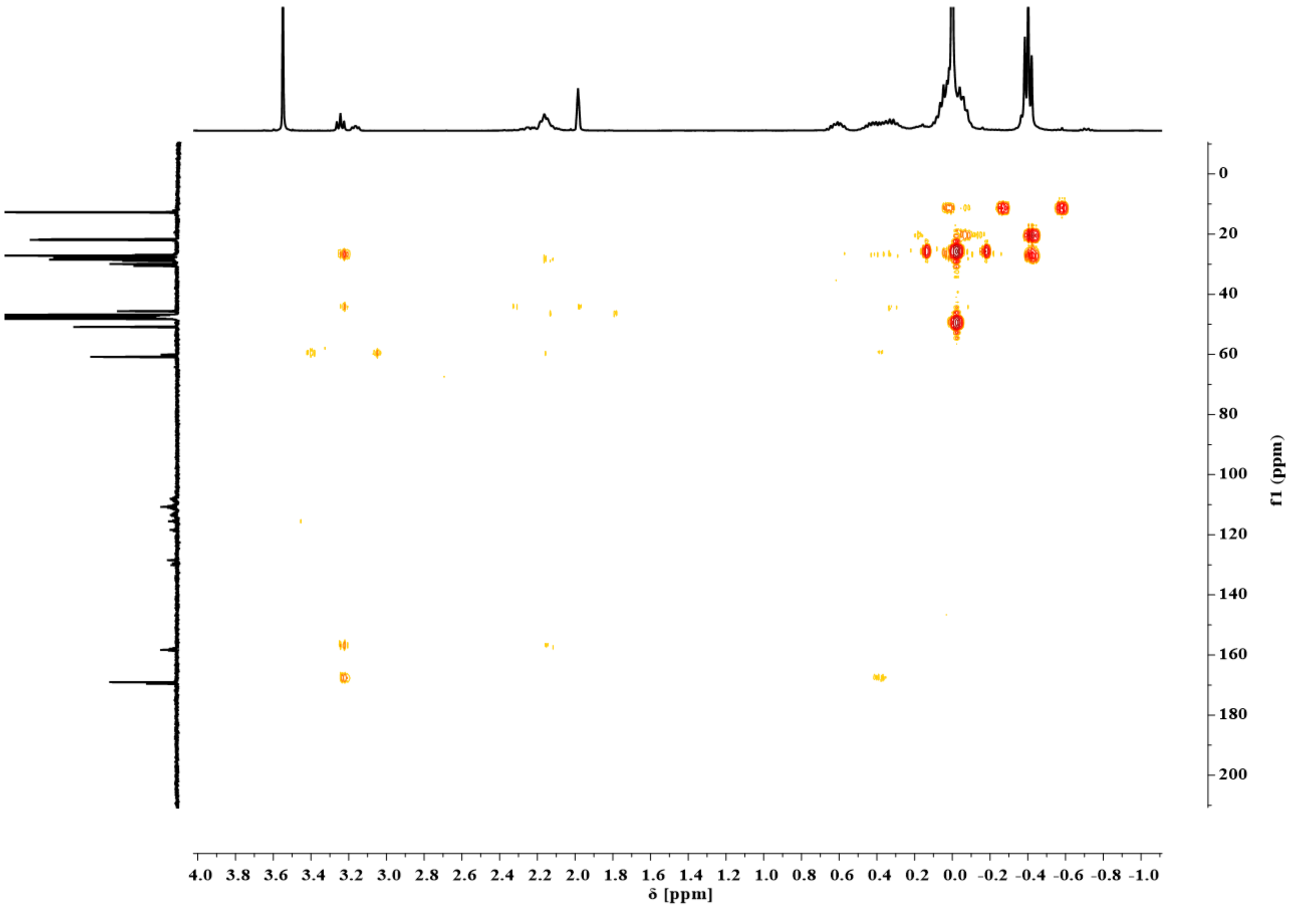

Supplementary Figure 102 | HMBC experiment of the title compound recorded in $CDCl_3$.

Ugi reaction of perfluorononanoic acid, valeraldehyde, *tert*-butylisocyanide and cyclohexylamine

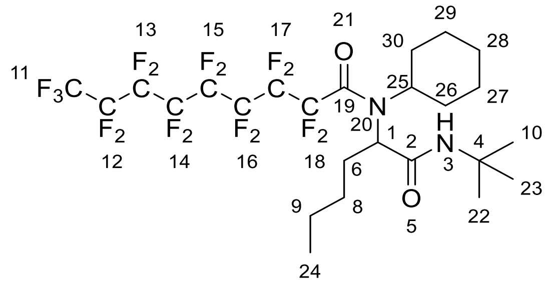

In a 25 mL round bottom flask valeraldehyde (126 mg, 1.46 mmol, 1.70 eq.) was stirred with cyclohexylamine (145 mg, 1.46 mmol, 1.70 eq.) for 60 min over sodium sulfate. Perfluorononanoic acid (400 mg, 862 µmol, 1.00 eq.) dissolved in 1 mL methanol was added to the imine at room temperature and the resulting mixture was stirred for 2 min. Subsequently, *tert*-butylisocyanide (165 µL, 122 mg, 1.46 mmol, 1.70 eq.) was added to the stirring mixture. The reaction was stirred for 4 d at room temperature. The crude reaction mixture was dried under reduced pressure and purified *via* column chromatography employing Fluoro*Flash*® silica gel. The fluorous fraction was concentrated and the residue was adsorbed onto celite® and purified *via* column chromatography employing silica gel as stationary phase and eluting with a gradual solvent mixture of ethyl acetate and *c*-hexane (0:1 → 1:1) to yield the Ugi product as a highly viscous yellow oil (42.2 mg, 61.2 µmol, 7.1%).

R_f = 0.52 in *c*-hexane/ethyl acetate (6:1). Visualized *via* Seebach staining solution and permanganate staining.

IR (ATR): ν[cm^{-1}] = 3337.4 (br, ν(N-H)), 2933.4 (s, ν(C-H)), 2875.3 (m), 1675.6 (s, ν(C=O)), 1534.8 (s), 1453.0 (m), 1238.8 (vs), 1206.8 (vs), 1148.2 (vs), 1109.0 (s), 999.9 (w), 896.1 (w), 785.3 (w), 735.0 (m), 702.4 (m), 668.5 (m), 557.5 (m), 528.0 (m).

^{1}H NMR (400 MHz, CD_3OD): δ [ppm] = 4.02 – 3.66 (m, 1 H, CH^{1}), 2.80 – 1.69 (m, 7 H, CH_2 + CH^{25}), 1.59 – 1.05 (m, 19 H, $CH_3^{10,22,23}$ + CH_2), 1.00 – 0.83 (m, 3 H, CH_3^{24}).

^{13}C NMR (101 MHz, CD_3OD): δ [ppm] = 180.2 (s, $CONR^{2}$), 171.8 (s, $CONR^{19}$), 63.5 (s, CH^{1a}), 59.7 (s, CH^{1b}), 32.5 (s, CH_2), 32.2 (s, CH_2), 30.6 (s, CH_2), 30.5 (s, CH_2), 30.4 (s, CH^{25}), 28.7 (s, CH_2), 28.6 (s, $CH_3^{10,22,23}$), 26.8 (s, CH_2), 26.6 (s, CH_2), 26.0 (s, CH_2), 23.6 (s, CH_2), 14.2 (s, CH_3^{24}).

^{19}F NMR (376 MHz, CD_3OD): δ [ppm] = -86.69 (t, J = 10.6 Hz, 3 F, CF_3^{11}), -114.08 – -114.49 (m, CF_2^{18a}), AB-signal (δ_A = -115.38, δ_B = -116.76, J_{AB} = 293.6 Hz, CF_2^{18b}, additional coupling not resolved, signals broadened), -124.02 – -124.67 (m, CF_2), -124.86 – -125.21 (m,

CF_2), -125.30 – -125.50 (m, CF_2), -126.98 (s, CF_2), -127.23 (s, CF_2), -128.08 (s, CF_2), -131.58 (s, CF_2[12]). Total integral of CF_2 region normalized with respect to the CF_3[11] group = 14.

ESI-MS [*m/z*]: $[M + Na]^+$ calculated for $^{12}C_{26}{}^{1}H_{31}{}^{16}O_2{}^{14}N_2{}^{19}F_{17}{}^{23}Na$, 737.2006; found, 737.2008, Δ = 0.20 mmu.

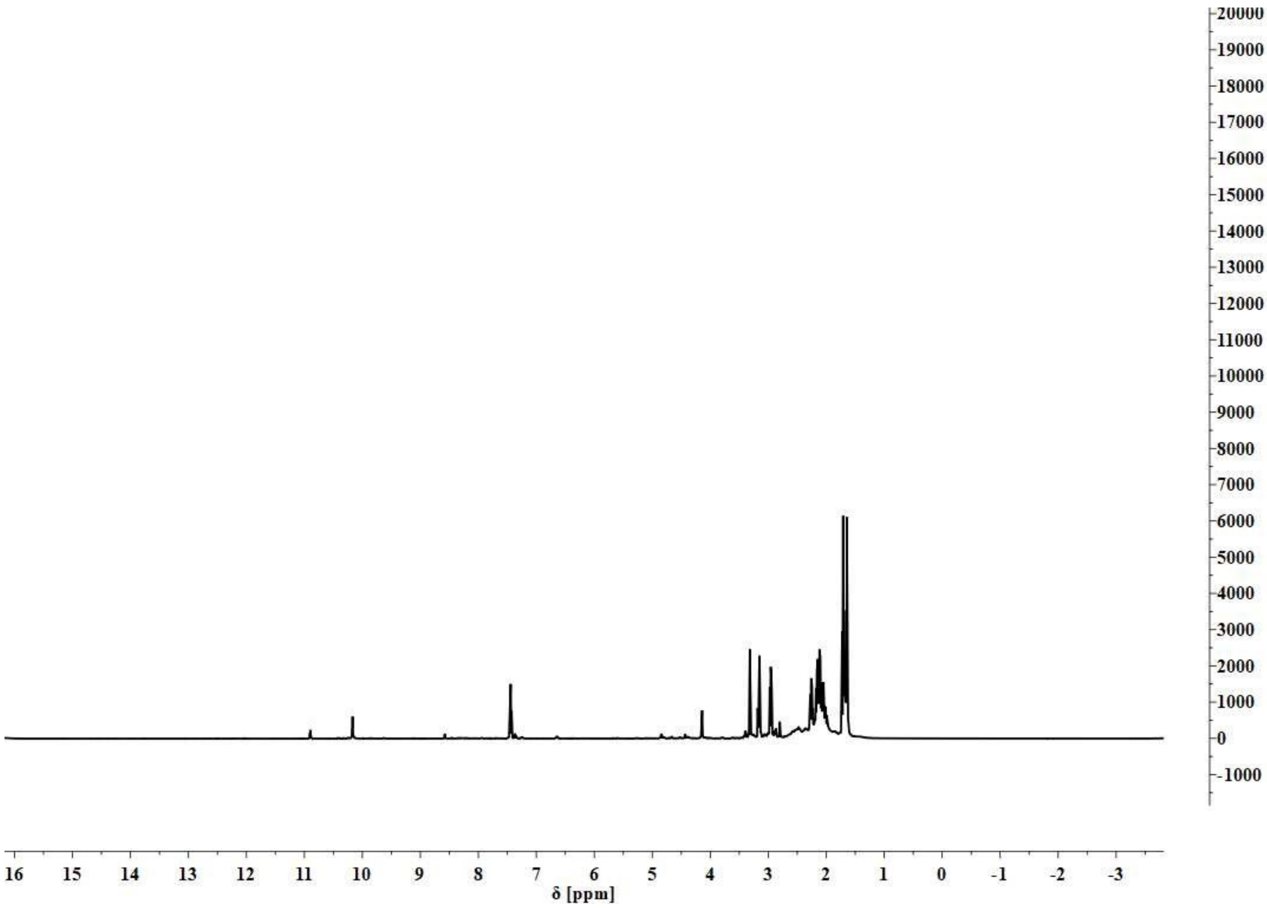

Supplementary Figure 103 | ^{1}H NMR experiment of the title compound recorded in CD_3OD.

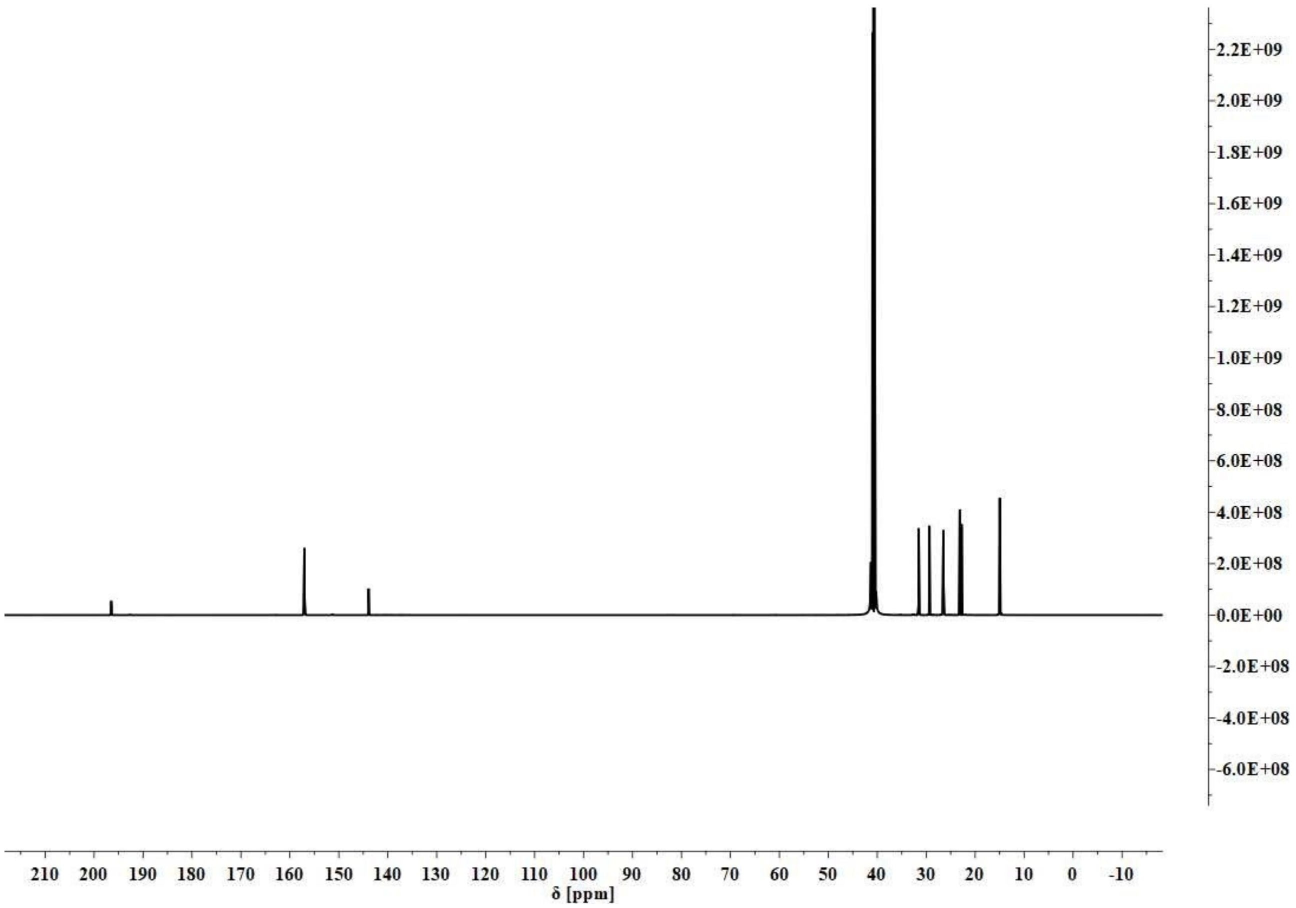

Supplementary Figure 104 | ^{13}C NMR experiment of the title compound recorded in CD_3OD.

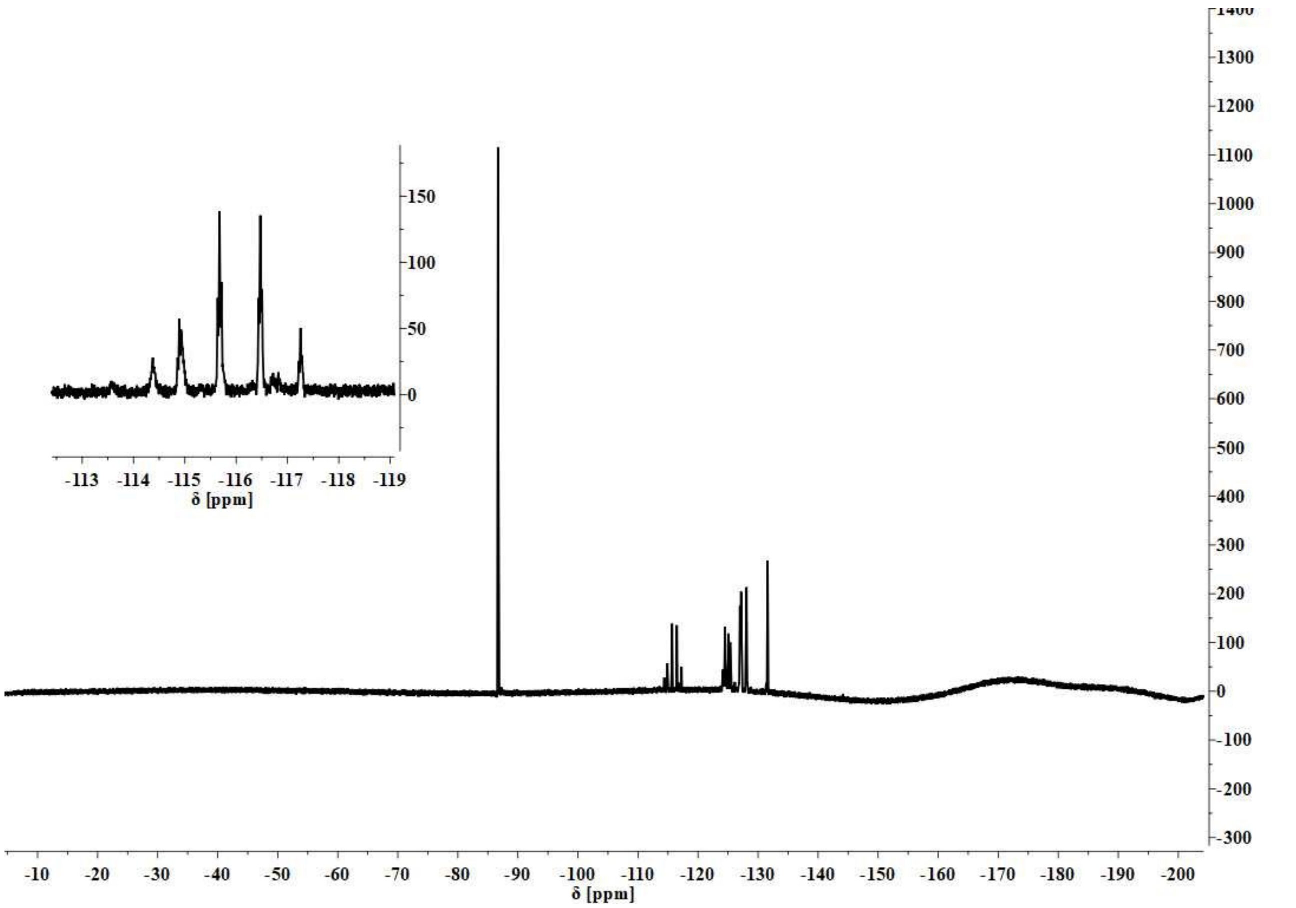

Supplementary Figure 105 | ^{19}F NMR experiment of the title compound recorded in CD_3OD.

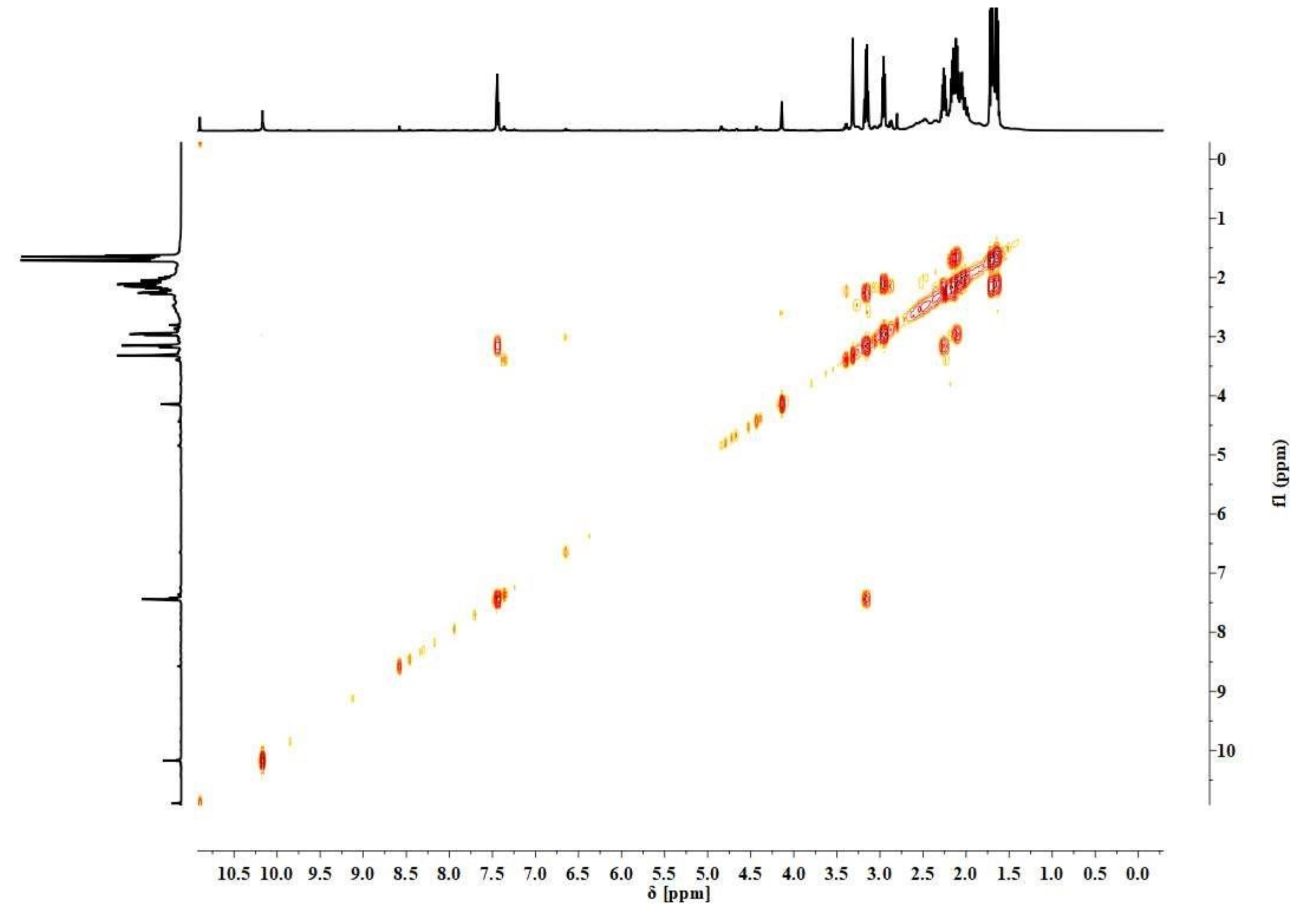

Supplementary Figure 106 | COSY experiment of the title compound recorded in CD_3OD.

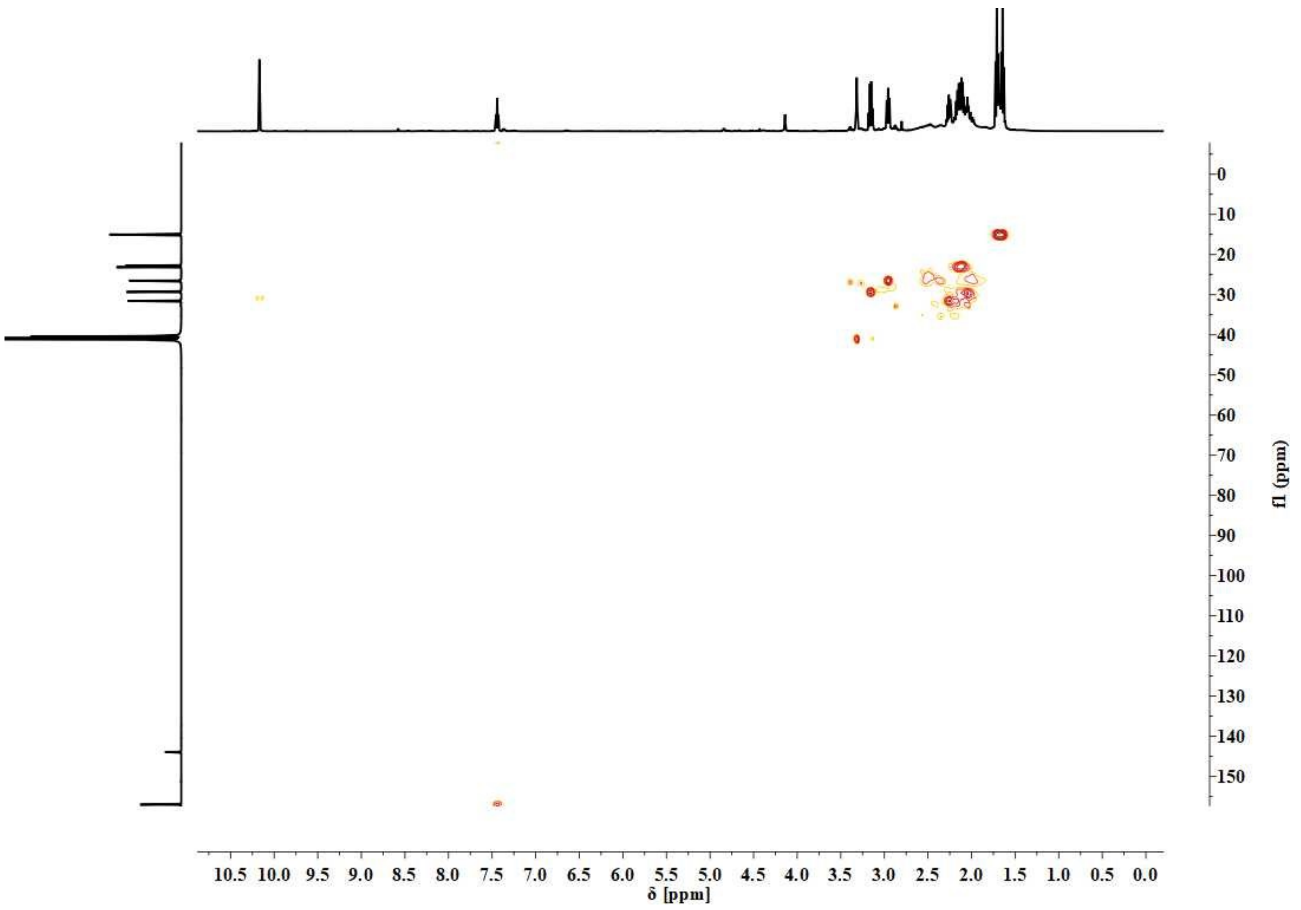

Supplementary Figure 107 | HSQC experiment of the title compound recorded in CD_3OD.

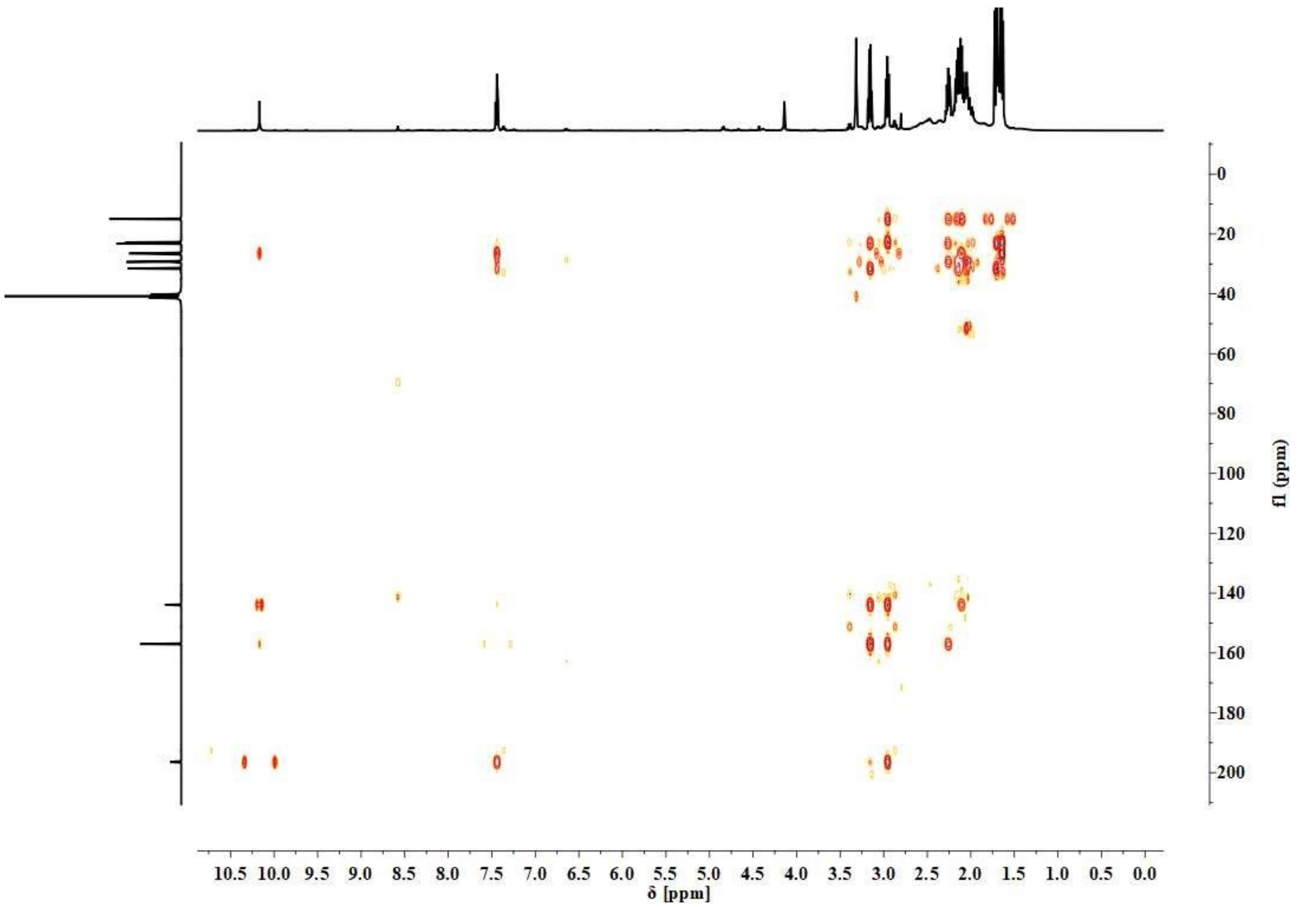

Supplementary Figure 108 | HMBC experiment of the title compound recorded in CD₃OD.

Ugi reaction of perfluorononanoic acid, valeraldehyde, *tert*-butylisocyanide and *tert*-butylamine

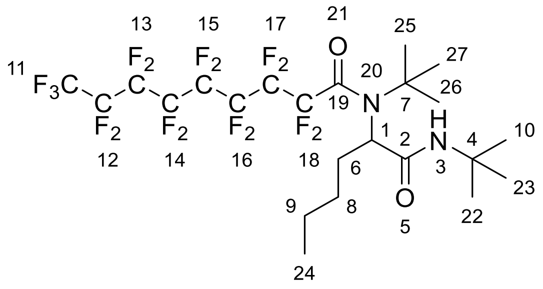

In a 25 mL round bottom flask valeraldehyde (126 mg, 1.46 mmol, 1.70 eq.) and *tert*-butylamine (107 mg, 1.46 mmol, 1.70 eq.) were stirred for 60 min over sodium sulfate. Perfluorononanoic acid (400 mg, 826 µmol, 1.00 eq.) dissolved in 1 mL methanol was added to the imine at room temperature and the resulting mixture was stirred for 2 min. Subsequently, *tert*-butylisocyanide (165 µL, 122 mg, 1.46 mmol, 1.70 eq.) was added to the stirring mixture. The reaction was stirred for 5 d at room temperature. The crude reaction mixture was dried under reduced pressure and purified *via* column chromatography employing Fluoro*Flash*® silica gel. The fluorous fraction was concentrated and the residue was adsorbed onto celite® and purified *via* column chromatography employing silica gel as stationary phase and eluting with a gradual solvent mixture of ethyl acetate and *c*-hexane (0:1 → 1:1) to yield the Ugi product as a highly viscous yellow oil (109 mg, 141 µmol, 17.3%).

R_f = 0.58 in *c*-hexane/ethyl acetate (6:1). Visualized *via* Seebach staining solution and permanganate staining.

IR (ATR): ν [cm^{-1}] = 2965.2 (m, ν(C-H)), 1683.9 (s, ν(C=O)), 1509.6 (m), 1456.9 (m), 1394.9 (m), 1366.9 (m), 1205.7 (s), 1146.3 (s), 1040.6 (w), 985.7 (w), 879.4 (w), 821.3 (w), 783.1 (w), 735.7 (m), 710.2 (m), 669.4 (m), 635.8 (m), 559.7 (m), 530.2 (m), 473.4 (w).

^{1}H NMR (400 MHz, CD_3OD): δ [ppm] = 4.34 (t, J = 6.8 Hz, 1 H, CH^{1}), 2.40 – 2.14 (m, 1 H, CH_2^{6a}), 1.86 – 1.66 (m, 1 H, CH_2^{6b}), 1.55 – 1.05 (m, 22 H, $CH_2^{8,9}$ + $CH_3^{10,22,23,25\text{-}27}$), 0.95 (t, J = 7.1 Hz, 3 H, CH_3^{24}).

^{13}C NMR (101 MHz, CD_3OD): δ [ppm] = 169.5 (s, CONR^{2}), 160.8 (s, CONR^{19}), 62.1 (s, C$^{4\ or\ 7}$), 61.3 (s, CH^{6}), 51.3 (s, C$^{4\ or\ 7}$), 32.2 (s, CH_2), 30.0 (s, CH_2), 28.3 (s, $CH_3^{10,22,23\ or\ 25\text{-}27}$), 27.2 (s, $CH_3^{10,22,23\ or\ 25\text{-}27}$), 22.3 (s, CH_2), 12.8 (s, CH_3^{24}).

^{19}F NMR (376 MHz, CD_3OD): δ [ppm] = -88.25 (t, J = 10.2 Hz, 3 F, CF_3^{11}), -113.23 – -116.89 (m, CF_2^{18}), -125.75 (s, CF_2), -128.64 (d, J = 64.9 Hz, CF_2), -129.65 (s, CF_2), -133.15 (s, CF_2^{12}). Total integral of CF_2 region normalized with respect to the CF_3^{11} group = 14.

ESI-MS [*m/z*]: [M + Na]$^+$ calculated for $^{12}C_{23}{}^{1}H_{29}{}^{16}O_2{}^{14}N_2{}^{19}F_{17}{}^{23}Na$, 711.1850 found, 711.18064, Δ = 1.35 mmu.

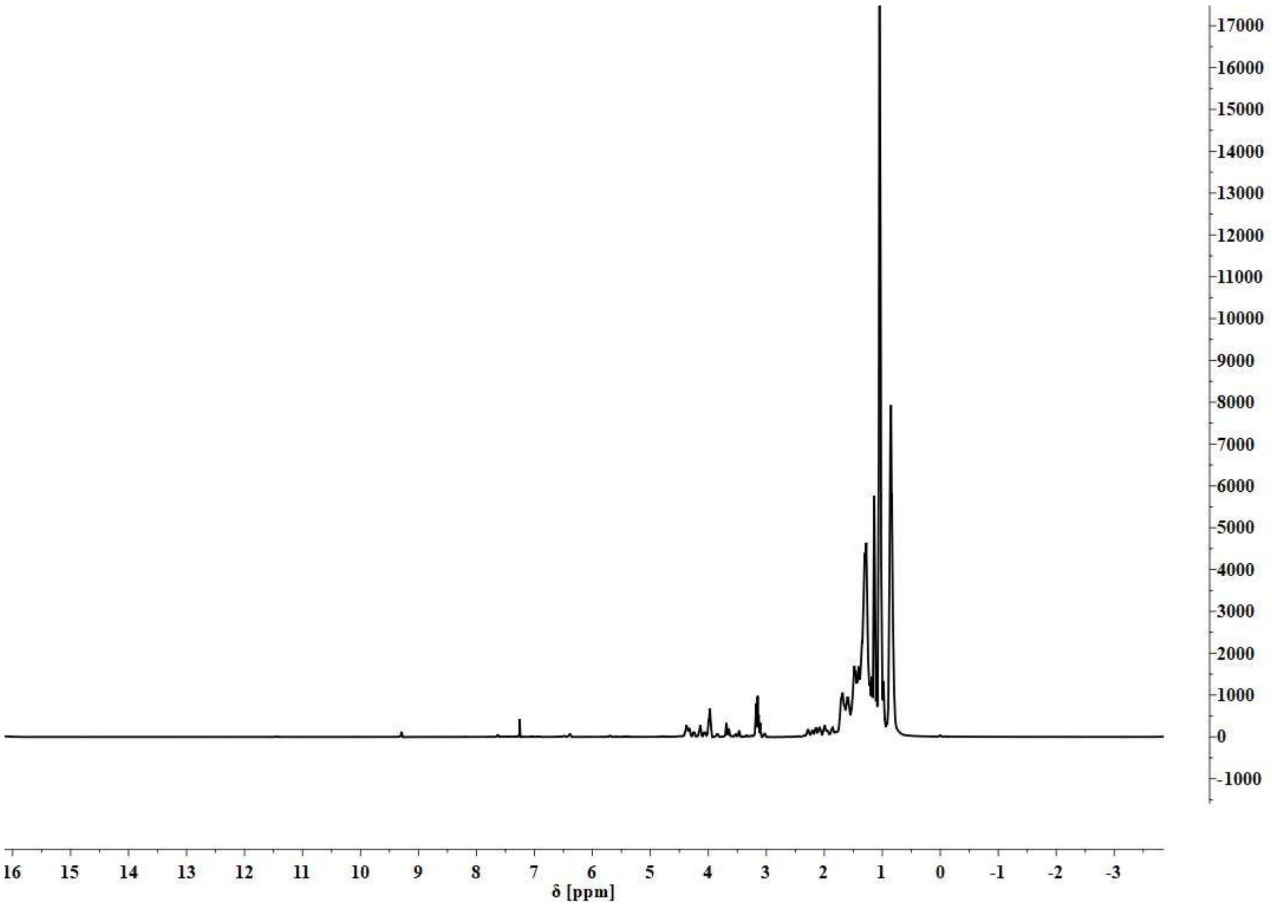

Supplementary Figure 109 | ^{1}H NMR experiment of the title compound recorded in CD_3OD.

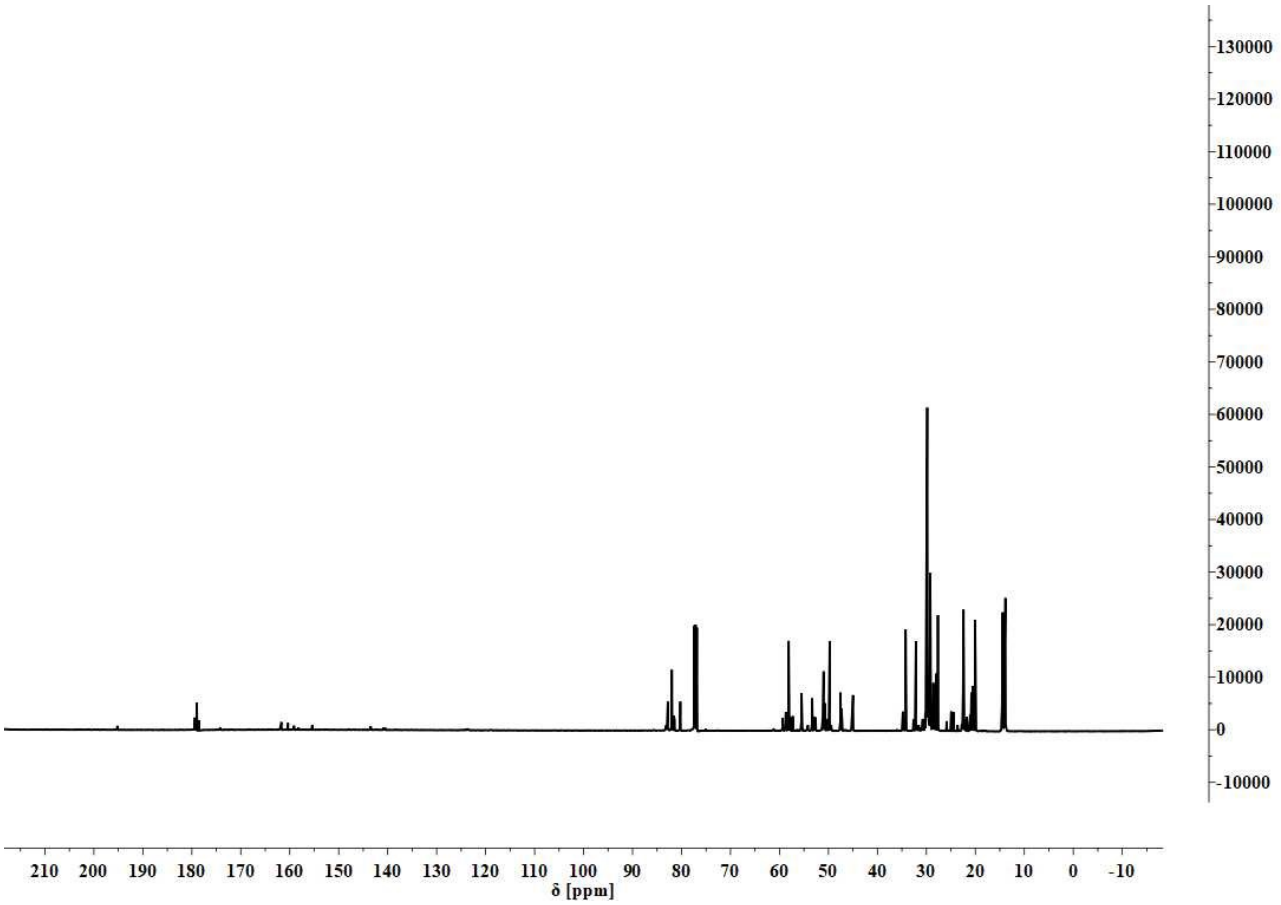

Supplementary Figure 110 | ^{13}C NMR experiment of the title compound recorded in CD_3OD.

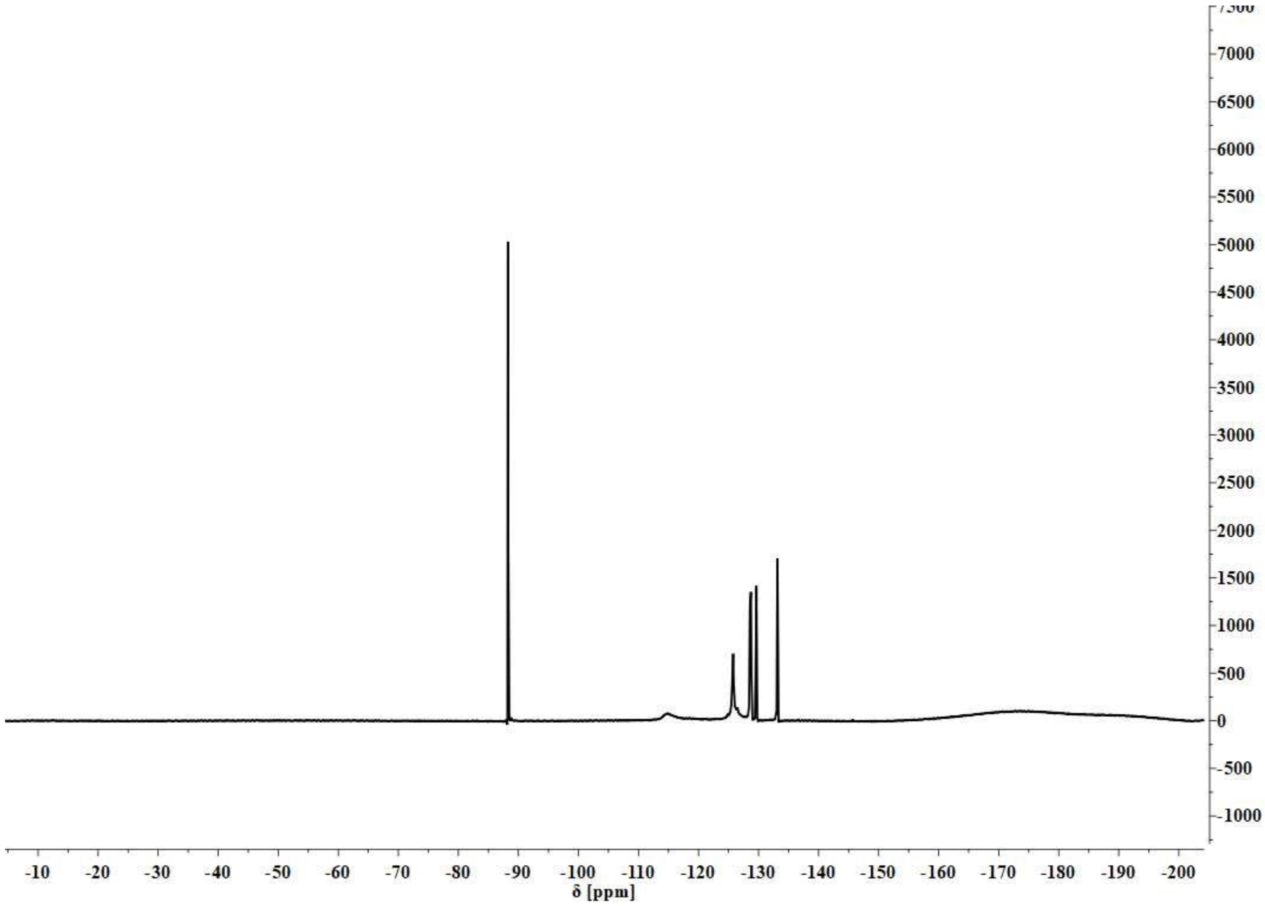

Supplementary Figure 111 | ^{19}F NMR experiment of the title compound recorded in CD_3OD.

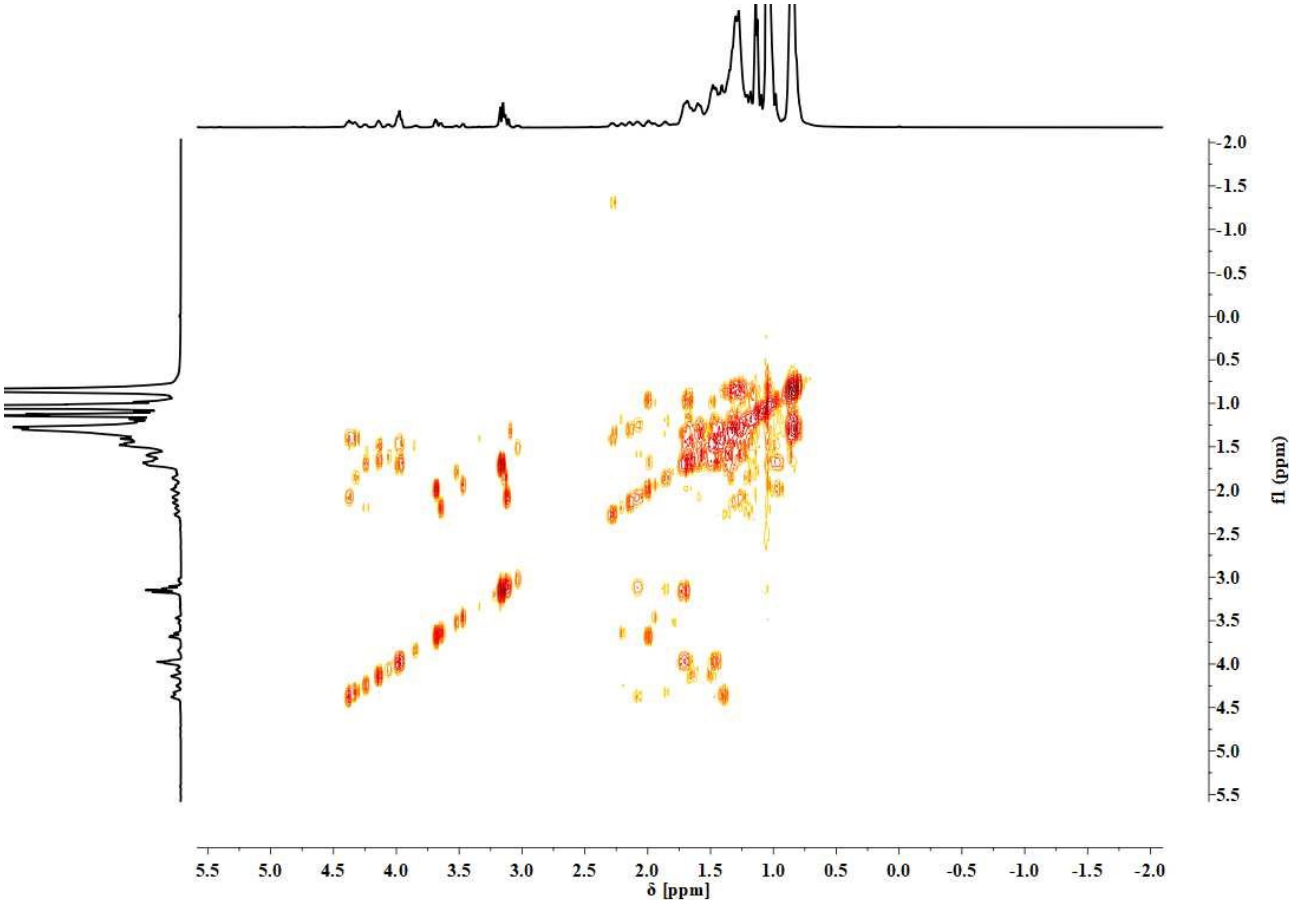

Supplementary Figure 112 | COSY experiment of the title compound recorded in CD_3OD.

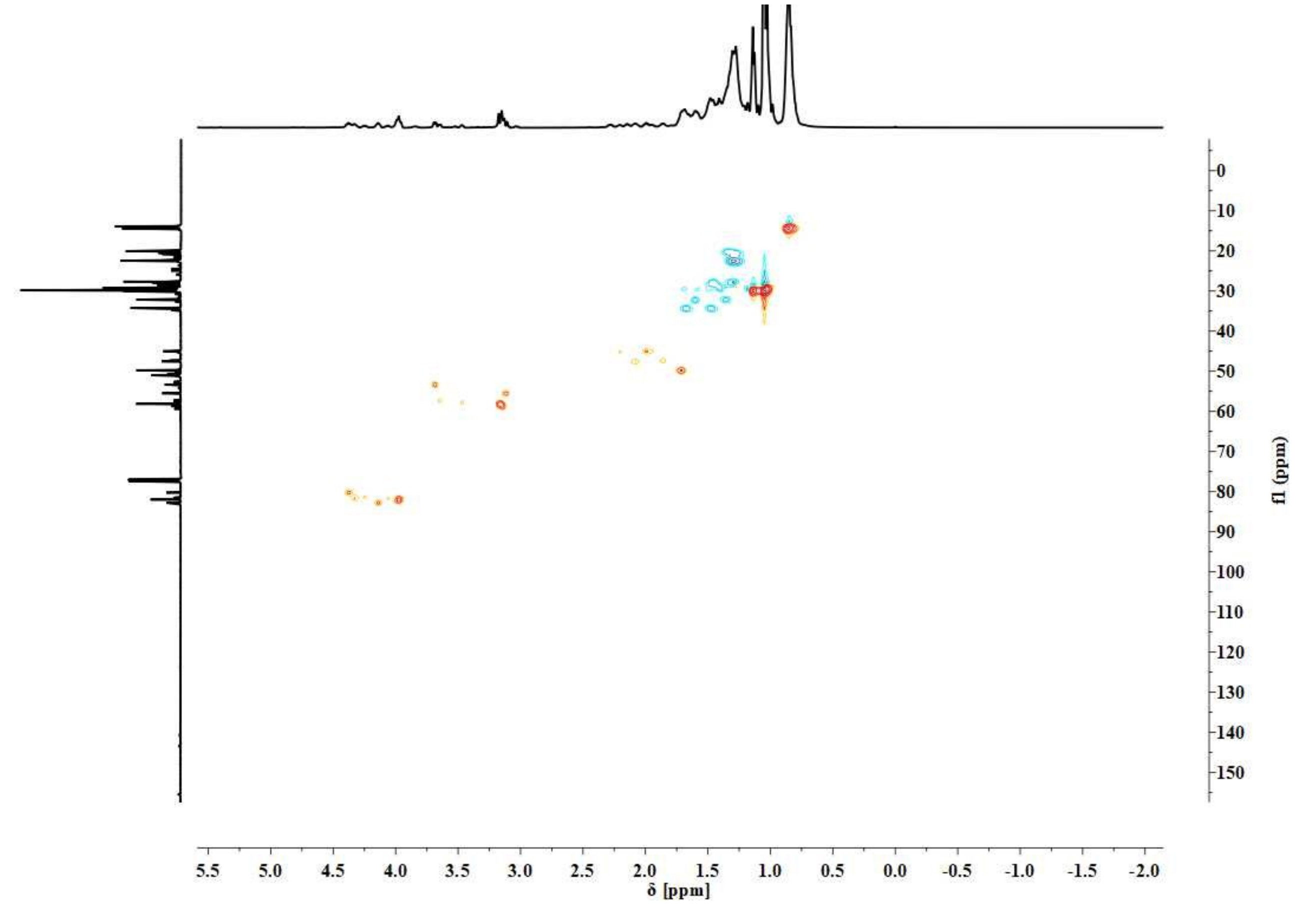

Supplementary Figure 113 | Multiplicity-edited HSQC experiment of the title compound recorded in CD_3OD.

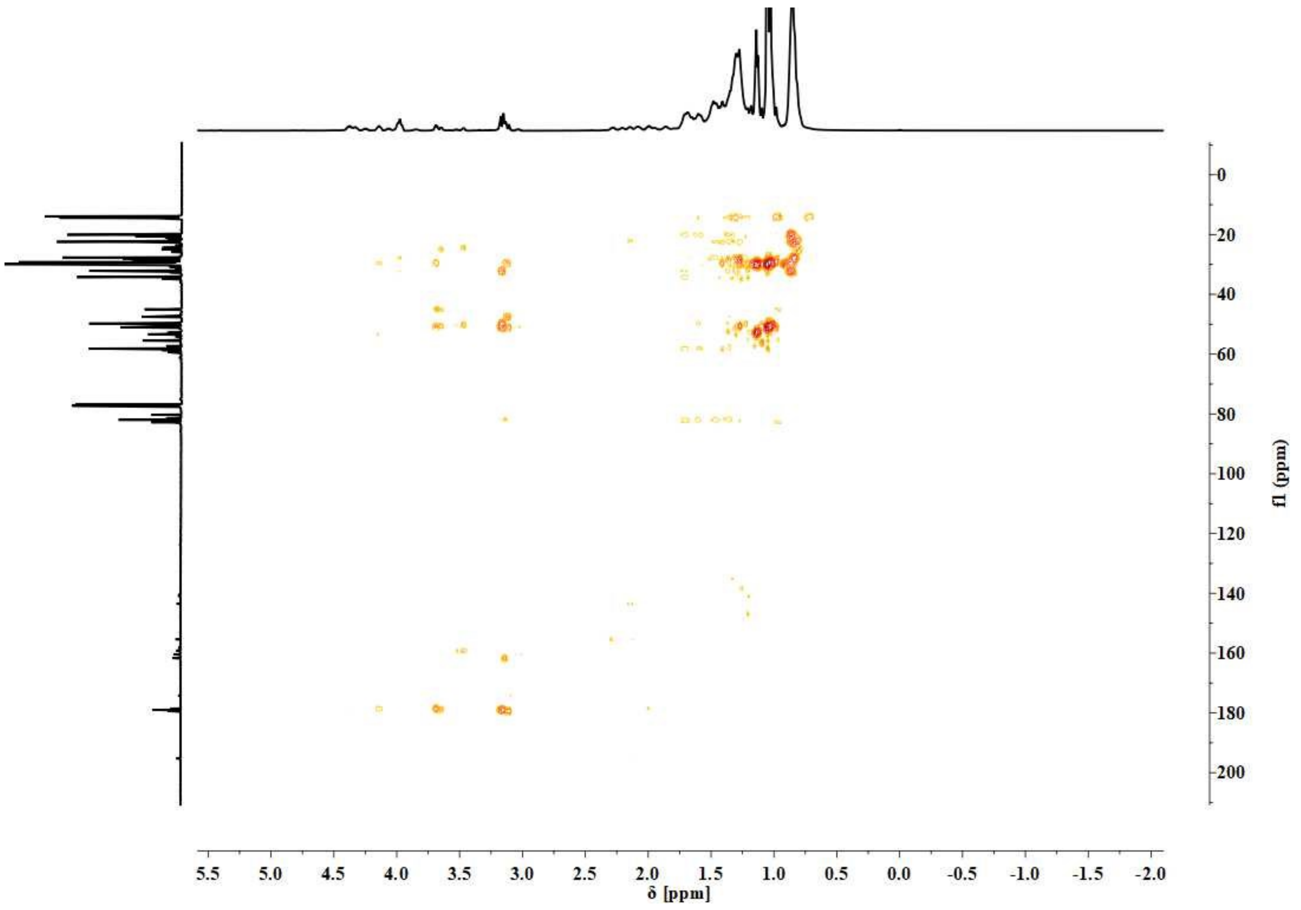

Supplementary Figure 114 | HMBC experiment of the title compound recorded in CD_3OD.

Ugi reaction of perfluorononanoic acid, isobutyraldehyde, cyclohexylisocyanide and pentylamine

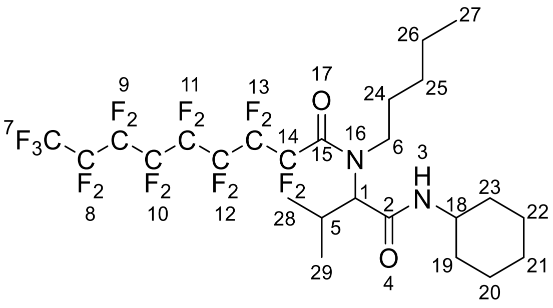

In a 25 mL round bottom flask isobutyraldehyde (83.6 µL, 66.0 mg, 916 µmol, 1.70 eq.) and pentylamine (106 µL, 79.8 mg, 916 µmol, 1.70 eq.) were added and the resulting mixture was stirred for 60 min over sodium sulfate. Perfluorononanoic acid (250 mg, 539 µmol, 1.00 eq.) dissolved in 0.5 mL methanol was added to the imine at room temperature and the resulting mixture was stirred for 2 min. Subsequently, cyclohexylisocyanide (114 µL, 100 mg, 916 µmol, 1.70 eq.) was added to the stirring mixture. The reaction was stirred for 3 d at room temperature. The crude reaction mixture was dried under reduced pressure. The residue was adsorbed onto celite® and purified *via* column chromatography employing silica gel as stationary phase and eluting with a gradual solvent mixture

of ethyl acetate and *c*-hexane (1:10 → 1:3) to yield the Ugi product as a yellow oil (95.1 mg, 133 mmol, 24.7%).

R_f = 0.54 in *c*-hexane/ethyl acetate (6:1). Visualized *via* permanganate staining solution.

IR (ATR): ν [cm^{-1}] = 3303.7 (br, ν(N-H)), 2927.3 (s, ν(C-H)), 2855.9 (m), 1764.4 (w), 1707.2 (s, ν(C=O)), 1673.4 (m), 1626.5 (s), 1538.8 (m), 1451.3 (m), 1429.5 (s), 1378.9 (m), 1239.5 (vs), 1211.9 (vs), 1146.6 (s), 1088.2 (m), 891.7 (w), 726.2 (w), 626.7 (m), 557.7 (w), 529.1 (w), 481.3 (w), 402.1 (w).

^{1}H NMR (400 MHz, CD_3OD): δ [ppm] = 3.71 – 3.30 (m, 2 H, CH^{1} + CH^{18}), 3.27 – 3.21 (m, 2 H, CH_2^{6}), 2.88 – 2.79 (m, 2 H, CH_2), 2.00 – 1.70 (m, 3 H, CH^{5} + CH_2), 1.67 – 1.53 (m, 2 H, CH_2), 1.50 – 1.16 (m, 12 H, CH_2), 1.06 (d, J = 6.8 Hz, 3 H, $CH_3^{28, 29}$), 0.97 – 0.77 (m, 6 H, $CH_3^{28, 29}$ + CH_3^{27}).

^{13}C NMR (101 MHz, CD_3OD): δ [ppm] = 166.9 (s, $CONR^{2}$), 162.8 (s, $CONR^{15}$), 51.8 (s, CH^{1}), 50.2 (s, CH^{18}), 42.5 (s, CH_2^{6}), 40.7 (s, CH_2), 33.1 (s, CH^{5}), 32.5 (s, CH_2), 30.9 (s, CH_2), 30.5 (s, CH_2), 30.1 (s, CH_2), 29.6 (s, CH_2), 28.8 (s, CH_2), 25.5 (s, CH_2), 25.4 (s, CH_2), 20.0 (s, $CH_3^{28, 29}$), 19.9 (s, $CH_3^{28, 29}$), 14.3 (s, CH_3^{27}).

^{19}F NMR (376 MHz, CD_3OD): δ [ppm] = -86.72 (t, J = 10.3 Hz, 3F, CF_3^{7}), -122.25 (t, J = 12.5 Hz, CF_2^{14}), -126.96 (s, CF_2), -127.28 (s, CF_2), -127.92 (s, CF_2), -128.12 (s, CF_2), -131.66 (s, CF_2^{8}). Total integral of CF_2 region normalized with respect to the CF_3^{7} group = 14.

ESI-MS [*m/z*]: [M + Na]$^+$ calculated for $^{12}C_{25}{}^{1}H_{31}{}^{16}O_2{}^{14}N_2{}^{19}F_{17}{}^{23}Na$, 737.2011; found, 737.2006, Δ = 0.42 mmu.

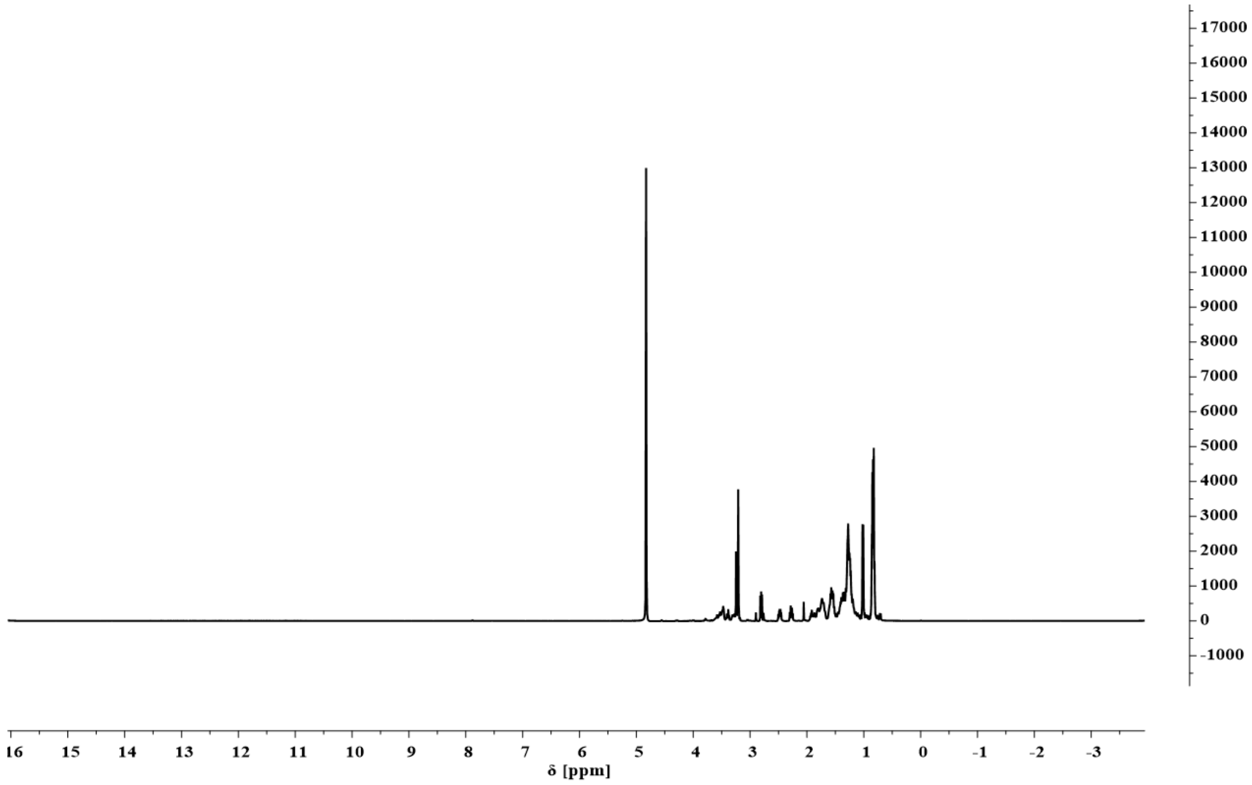

Supplementary Figure 115 | ^1H NMR experiment of the title compound recorded in CD_3OD.

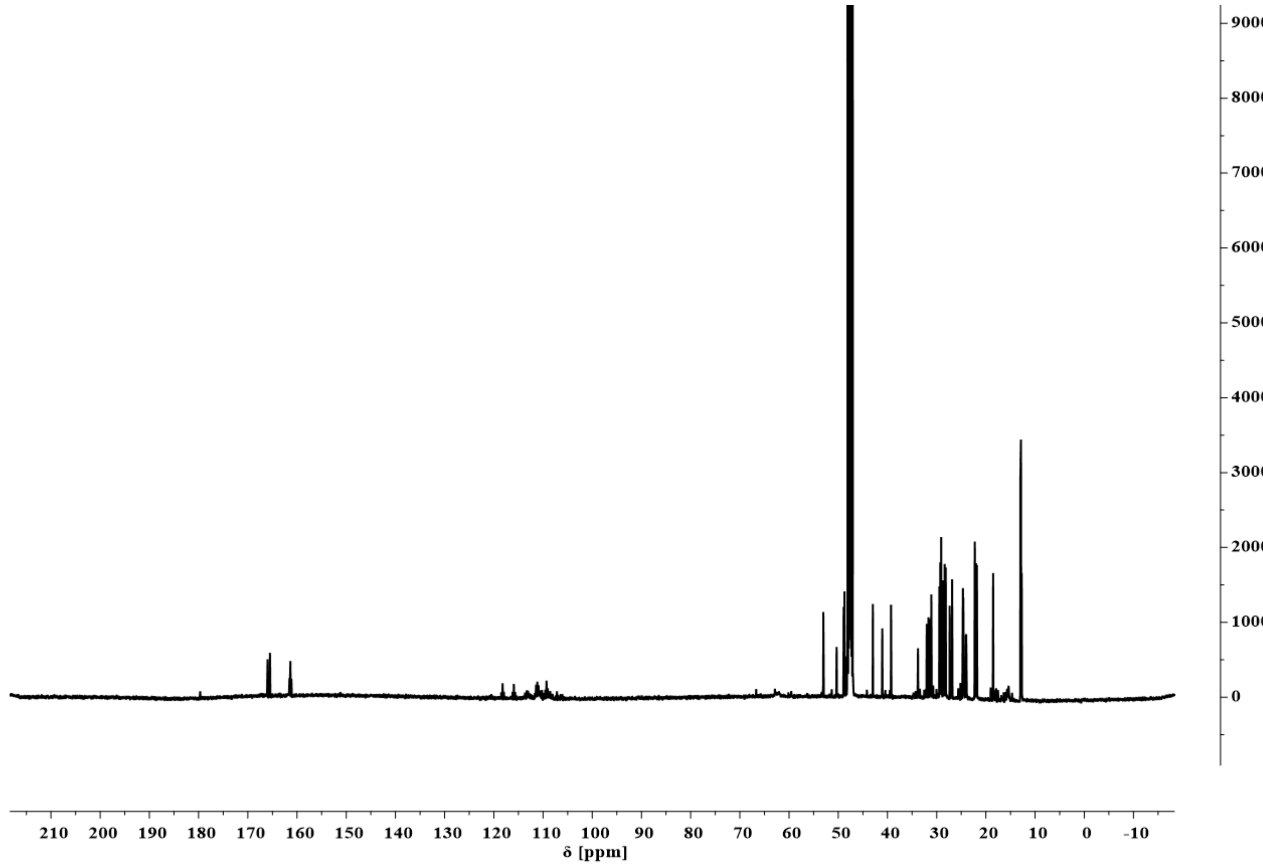

Supplementary Figure 116 | ^{13}C NMR experiment of the title compound recorded in CD_3OD.

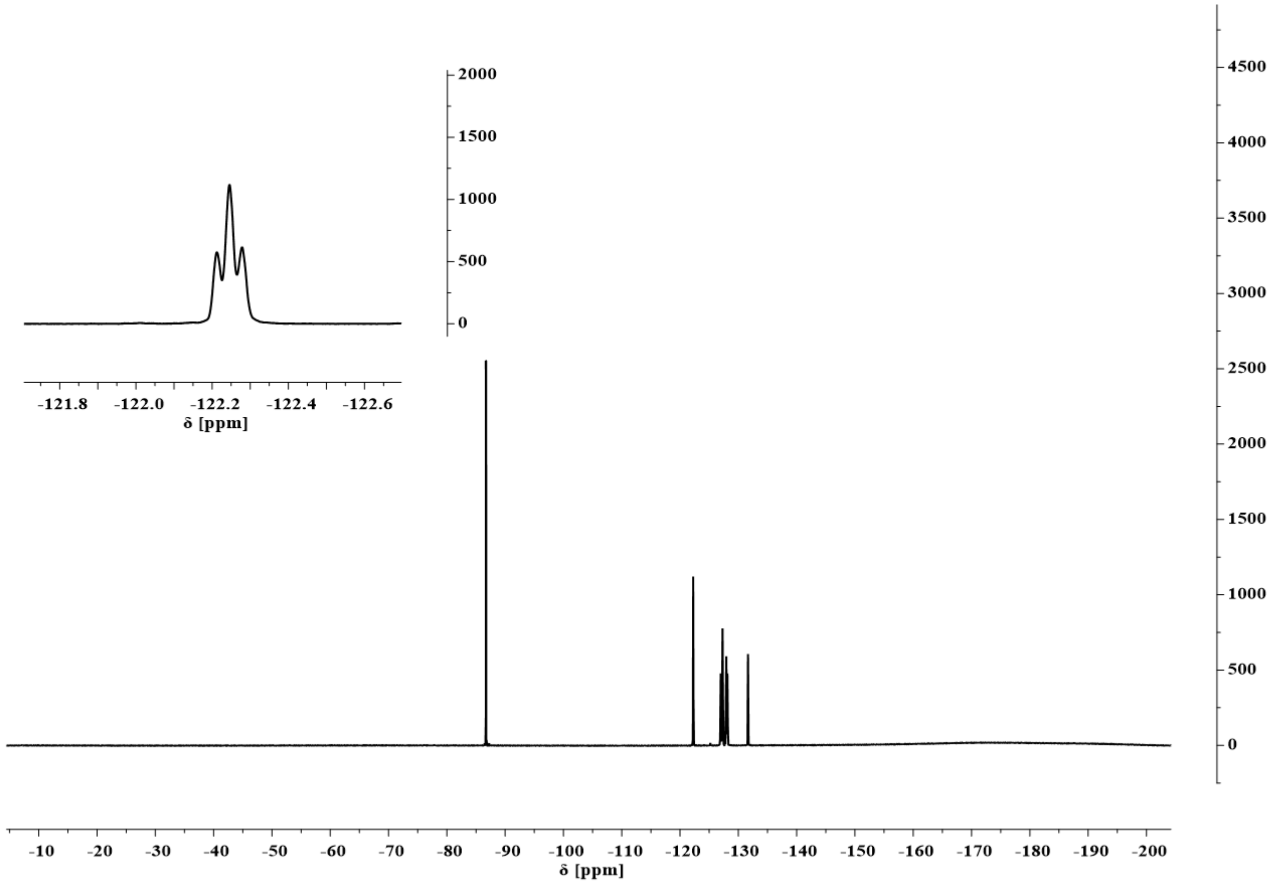

Supplementary Figure 117 | ^{19}F NMR experiment of the title compound recorded in CD_3OD.

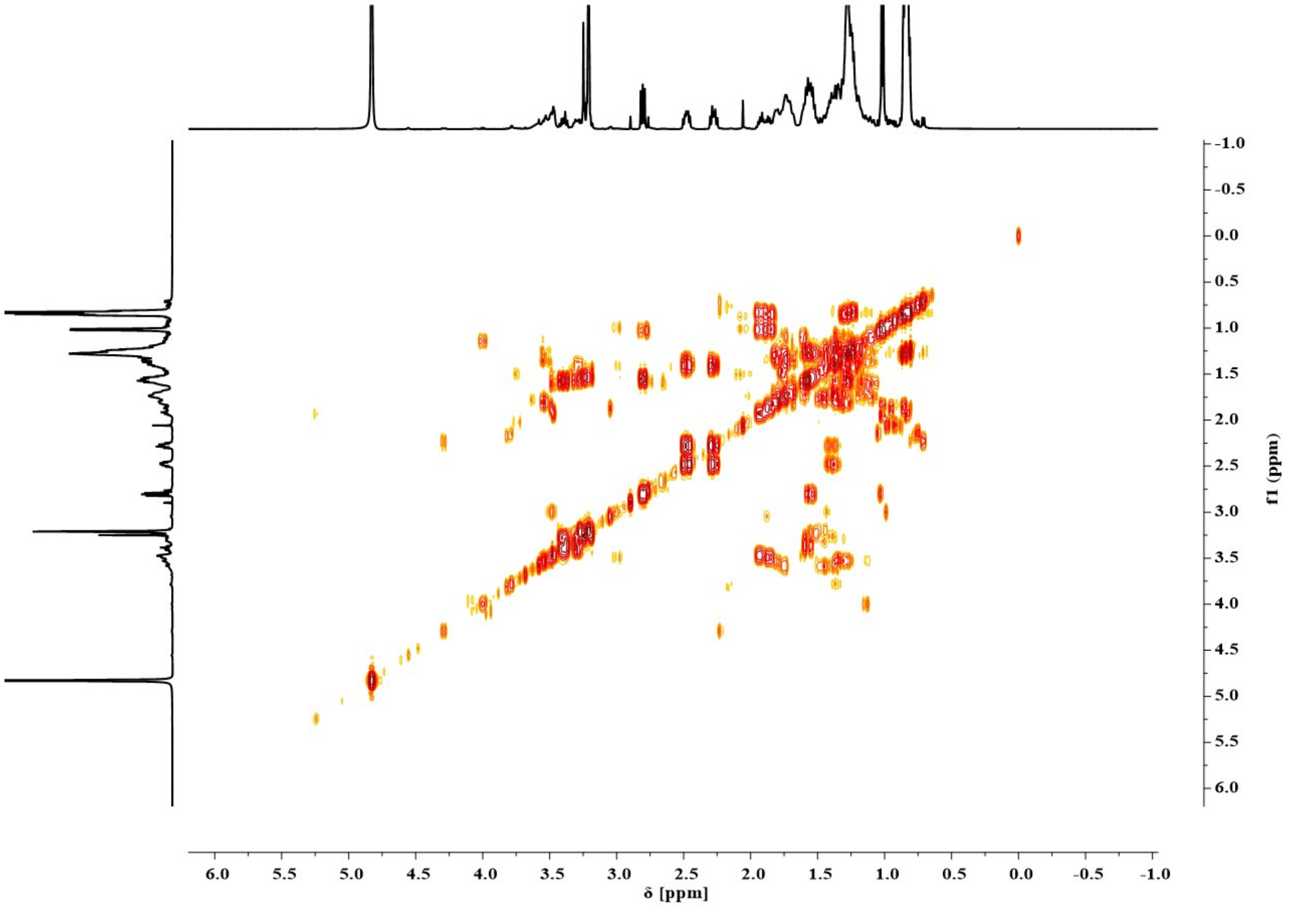

Supplementary Figure 118 | COSY experiment of the title compound recorded in CD_3OD.

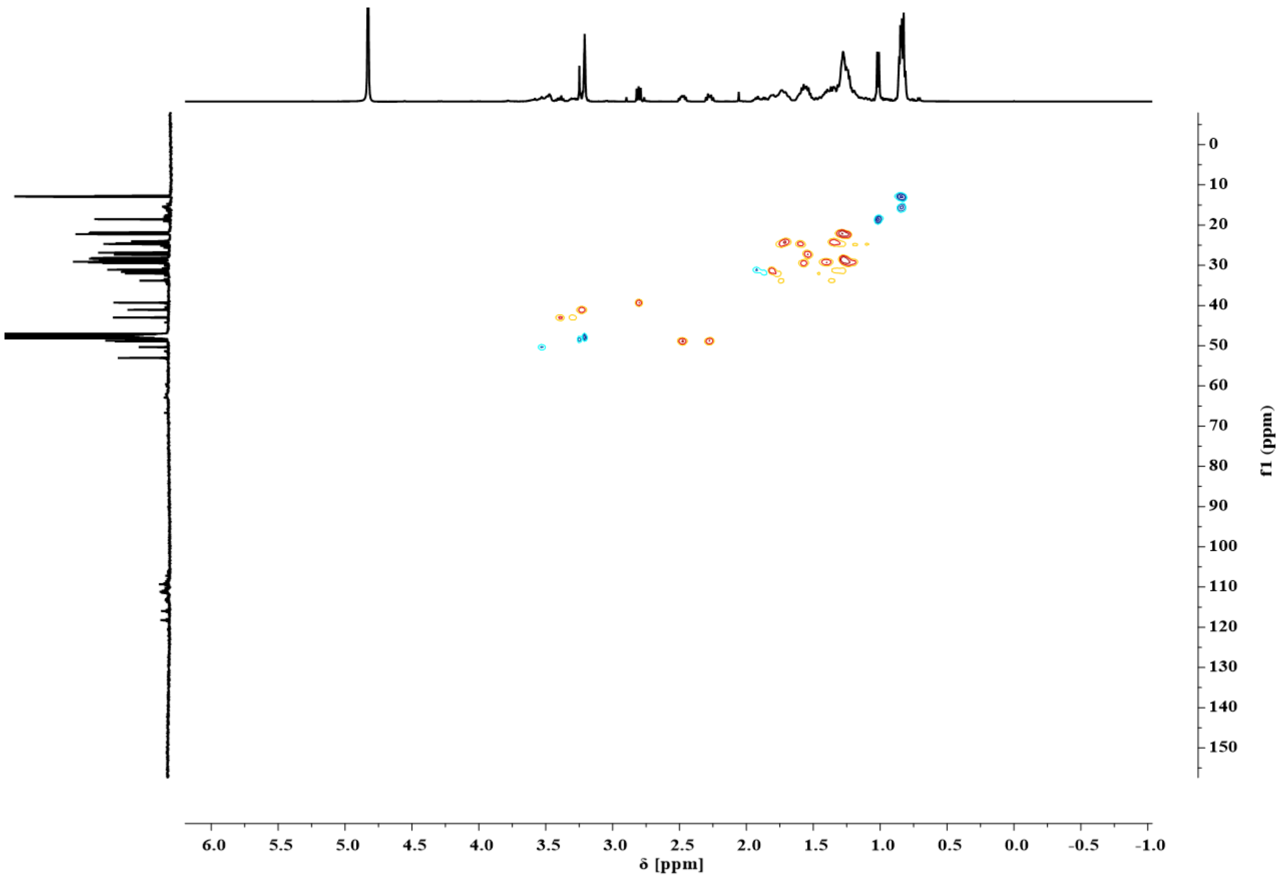

Supplementary Figure 119 | Multiplicity-edited HSQC experiment of the title compound recorded in CD_3OD.

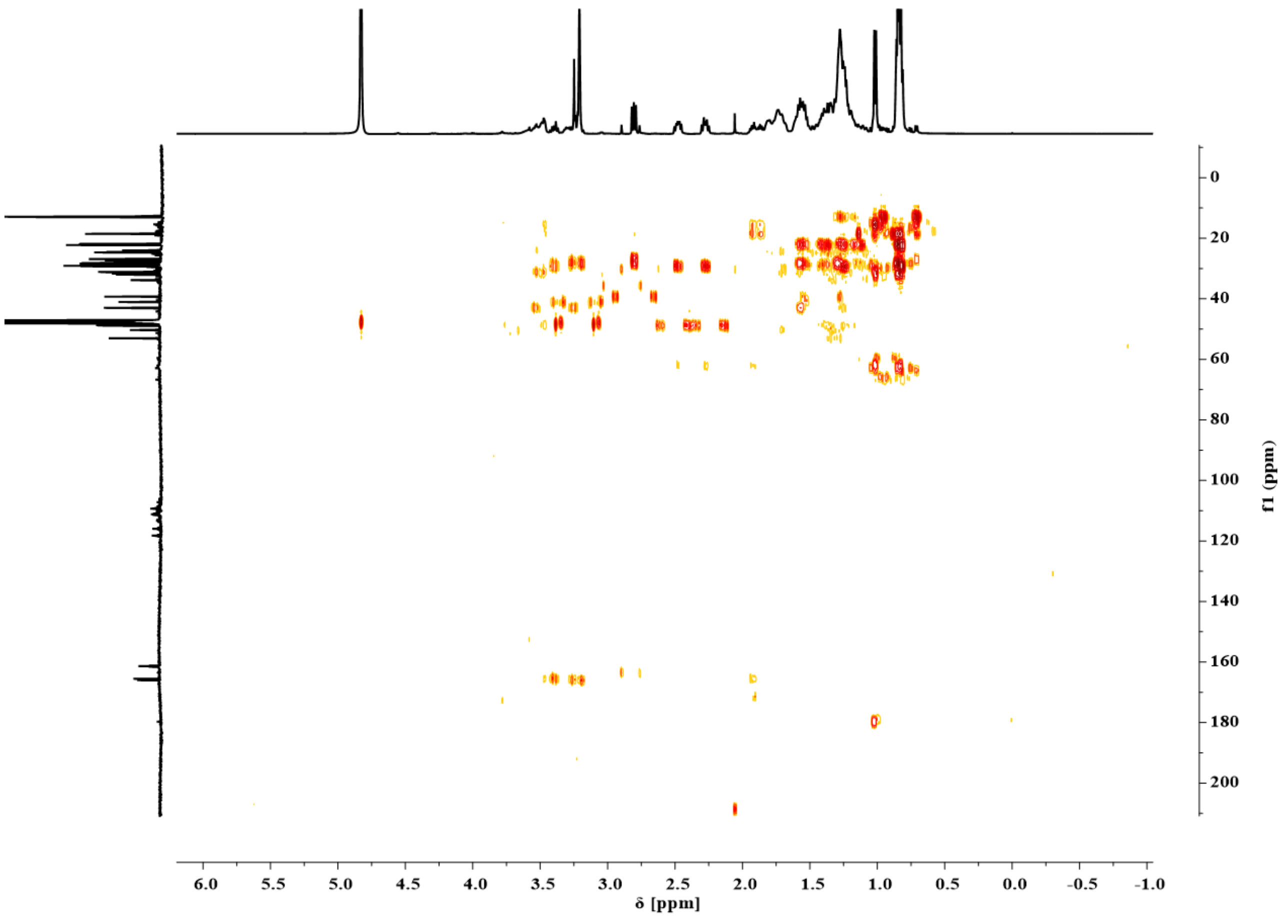

Supplementary Figure 120 | HMBC experiment of the title compound recorded in CD_3OD.

Ugi reaction of perfluorononanoic acid, valeraldehyde, cyclohexylisocyanide and pentylamine

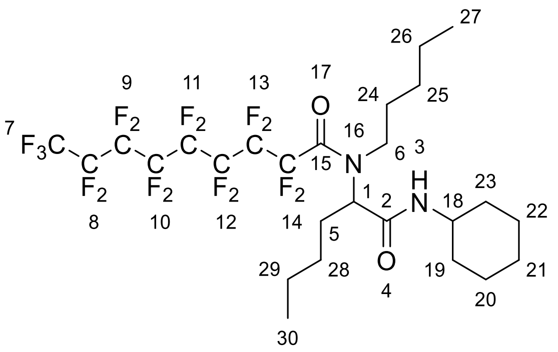

In a 25 mL round bottom flask valeraldehyde (52.1 µL, 42.2 mg, 490 µmol, 1.30 eq.) was dissolved in 1.5 mL methanol, subsequently pentylamine (56.6 µL, 42.7 mg, 490 µmol, 1.30 eq.) was added and the resulting mixture was stirred for 60 min over sodium sulfate. Afterwards, the mixture was filtrated and the solid was washed with 10 mL methanol three times. Subsequently, the filtrate was concentrated under reduced pressure. Perfluorononanoic acid (175 mg, 377 µmol, 1.00 eq.) dissolved in 1 mL methanol was added to the imine at room temperature and the resulting mixture was stirred for 2 min. Subsequently, cyclohexylisocyanide (56.3 µL, 59.4 mg, 453 µmol, 1.20 eq.) was added to the stirring mixture. The reaction was stirred for 4 d at room temperature. The crude reaction mixture

was dried under reduced pressure and purified *via* column chromatography employing Fluoro*Flash*® silica gel. The fluorous fraction was tested for purity *via* TLC and concentrated under reduced pressure. The remaining perfluoro acid was removed with a short silica gel filter column, eluting with *c*-hexane/ethyl acetate (3:1) to yield the Ugi product as a colorless solid (86.0 mg, 118 µmol, 31.4%).

R_f = 0.40 in *c*-hexane/ethyl acetate (6:1). Visualized *via* Seebach staining solution.

IR (ATR): ν [cm^{-1}] = 3325.5 (br, ν(N-H)), 2933.4 (m, ν(C-H)), 2859.3 (m, ν(C-H)), 1651.9 (s, ν(C=O)), 1534.7 (m), 1452.3 (m), 1367.9 (w), 1237.4 (vs), 1203.2 (vs), 1146.7 (vs), 1054.4 (w), 965.9 (w), 890.9 (w), 777.3 (m), 735.3 (m), 703.5 (m), 656.3 (m), 557.8 (m), 528.9 (m).

^{1}H NMR (400 MHz, CD_3OD): δ [ppm] = 4.59 (t, J = 7.6 Hz, CH^{1a}), 4.50 (t, J = 7.2 Hz, total integral of CH^{1} = 1 H, CH^{1b}), 3.69 – 3.43 (m, 3 H, $CH_2^{6\ or\ 5}$ + CH^{18}), 2.03 – 1.60 (m, 10 H, CH_2), 1.43 – 1.15 (m, 12 H, CH_2), 0.92 (t, J = 7.2 Hz, 6 H, $CH_3^{27,30}$).

^{13}C NMR (101 MHz, CD_3OD): δ [ppm] = 170.4 (s, $CONR^{2}$), 159.7 (s, $CONR^{15}$), 62.0 (s, CH^{1a}), 61.4 (s, CH^{1b}), 50.1 (s, CH^{18}), 47.2 (s, $CH_2^{6\ or\ 5}$), 33.6 (s, CH_2), 33.4 (s, CH_2), 32.0 (s, CH_2), 31.4 (s, CH_2), 30.4 (s, CH_2), 30.0 (s, CH_2), 29.6 (s, CH_2), 29.5 (s, CH_2), 28.4 (s, CH_2), 26.6 (s, CH_2), 26.0 (s, CH_2), 23.5 (s, CH_2), 23.3 (s, CH_2), 14.3 (s, $CH_3^{27\ or\ 30}$), 14.2 (s, $CH_3^{27\ or\ 30}$).

^{19}F NMR (376 MHz, CD_3OD): δ [ppm] = -88.27 (t, J = 10.4 Hz, 3 F, CF_3^{7}), AB-signal (δ_A = -116.17, δ_B = -116.99, J_{AB} = 301.2 Hz, A and B are split into t, J = 12.4 Hz, CF_2^{14a}), AB-signal (δ_A = -118.12, δ_B = -118.63, J_{AB} = 293.6 Hz, A and B are split into t, J = 12.3 Hz CF_2^{14b}), -126.20 (s, CF_2), -126.78 (s, CF_2), -127.15 (s, CF_2), -128.74 (s, CF_2), -129.66 (s, CF_2), -133.17 (s, CF_2^{8}). Total integral of CF_2 region normalized with respect to the CF_3^{7} group = 14.

FAB – MS [*m/z*] (relative intensity): 729.3 (55%) $[M + H]^+$, 631.2 (10%) [Fragment A]$^+$, 630.2 (28%) [Fragment A – H]$^+$, 281.3 (7%) [Fragment B]$^+$, 197.2 (16%) [Fragment C + H]$^+$.

HRMS – FAB [*m/z*]: $[M + H]^+$ calculated for $^{12}C_{26}{}^{1}H_{34}{}^{16}O_2{}^{14}N_2{}^{19}F_{17}$, 729.2343; found, 729.2342; Δ = 0.16 mmu.

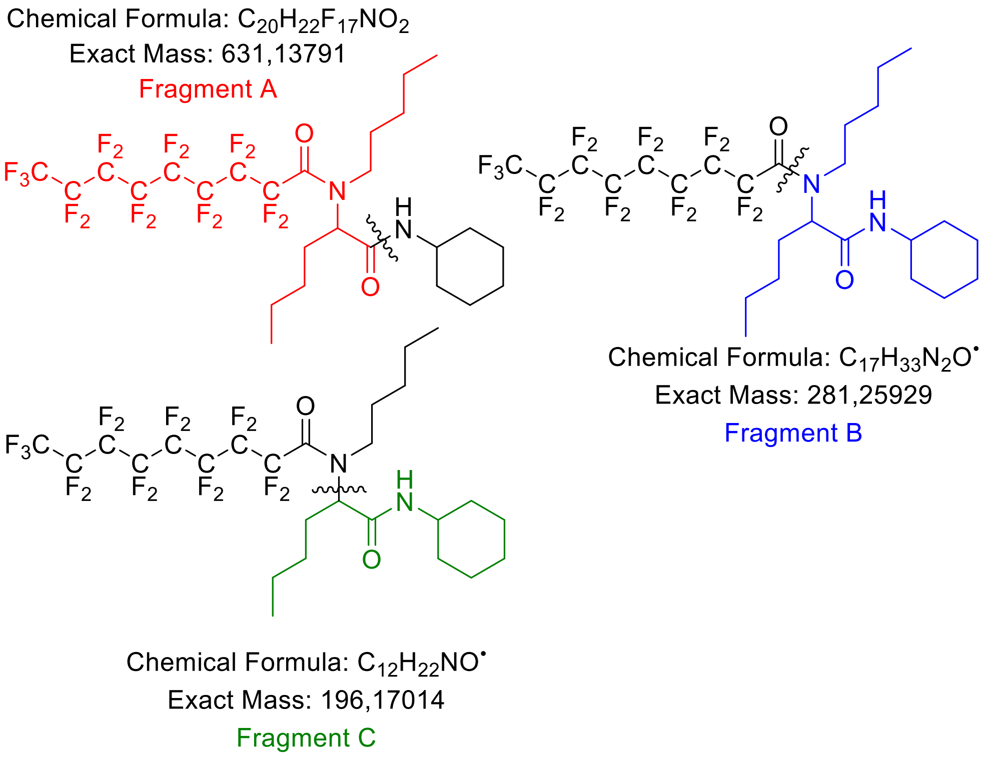

Supplementary Figure 121 | Proposed fragments observed in FAB-MS.

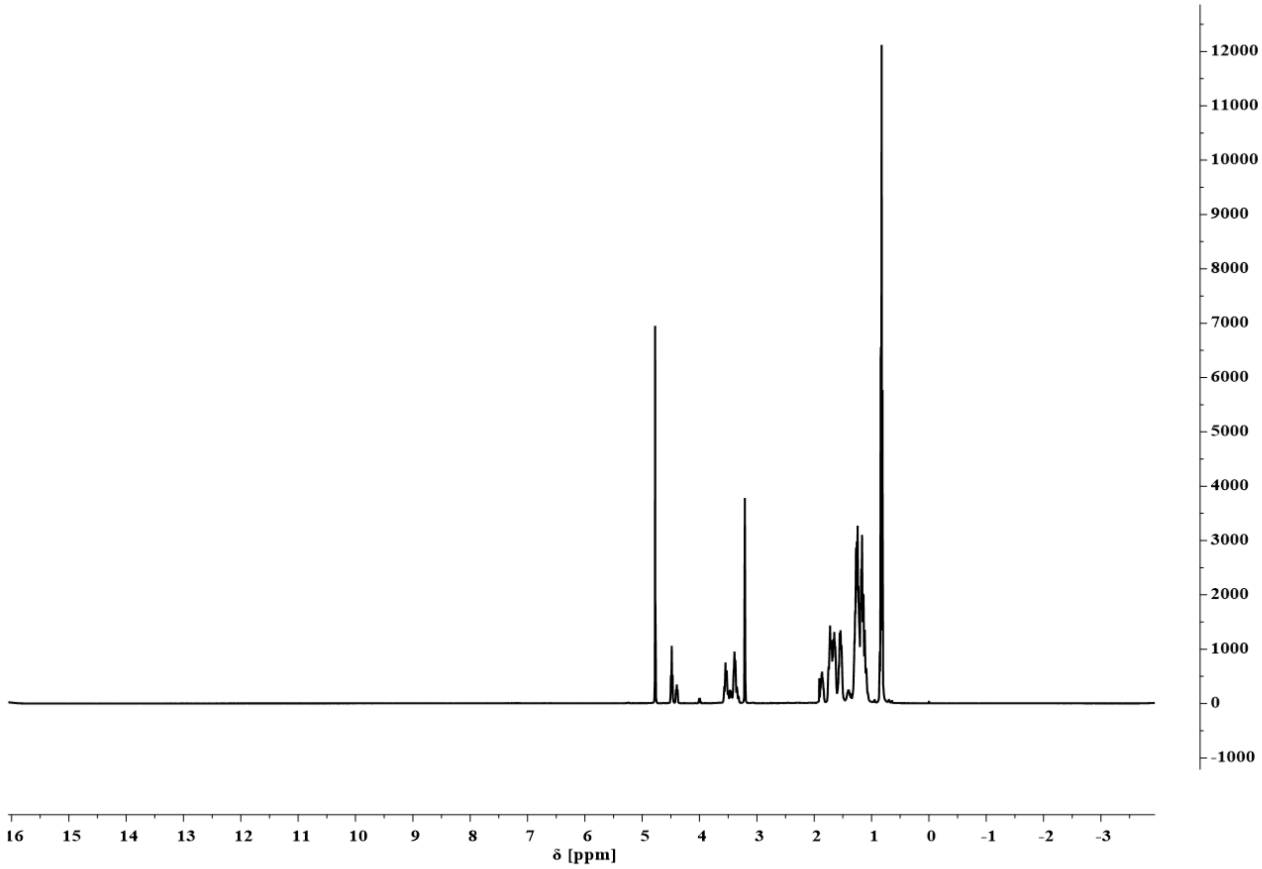

Supplementary Figure 122 | ^{1}H NMR experiment of the title compound recorded in CD_3OD.

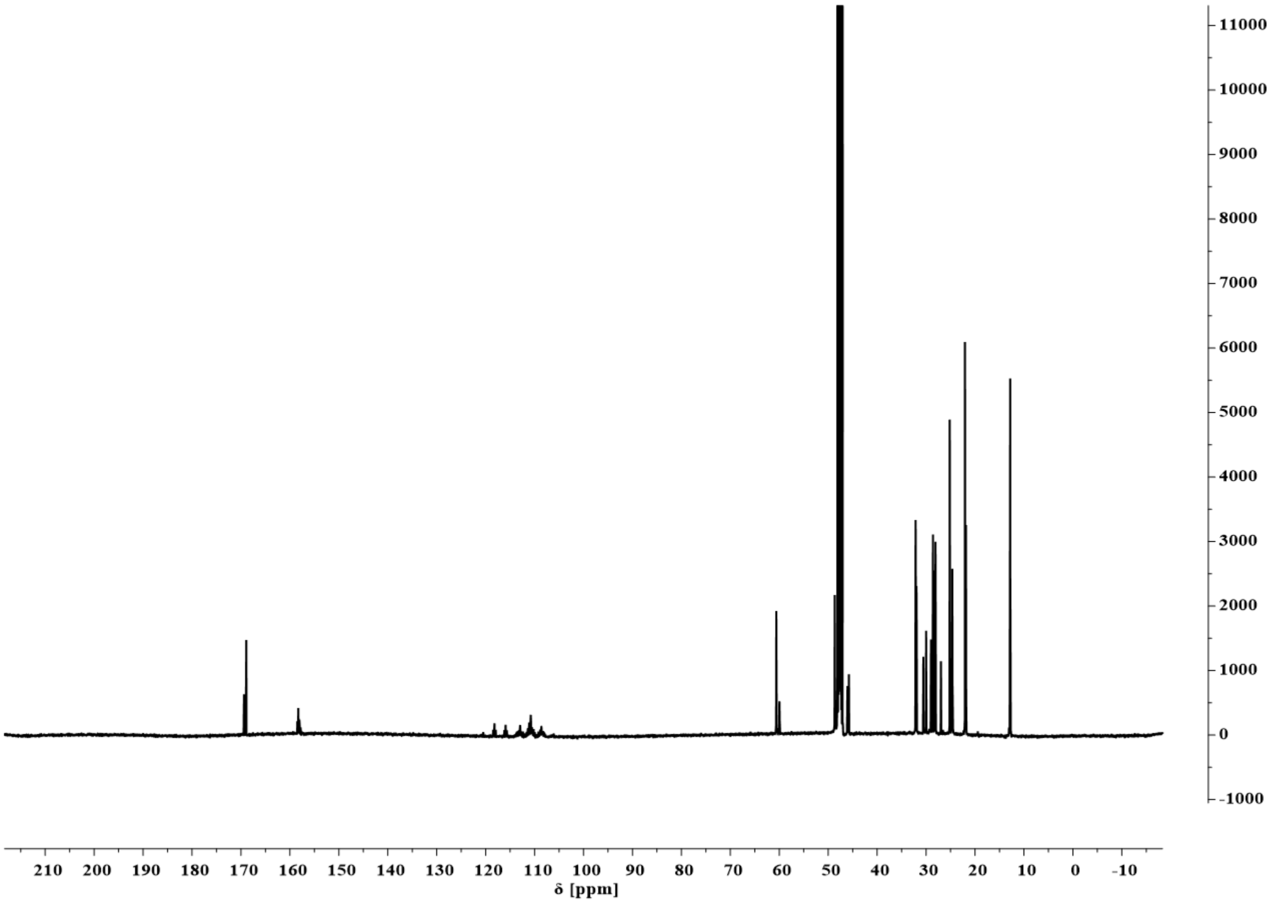

Supplementary Figure 123 | ^{13}C NMR experiment of the title compound recorded in CD_3OD.

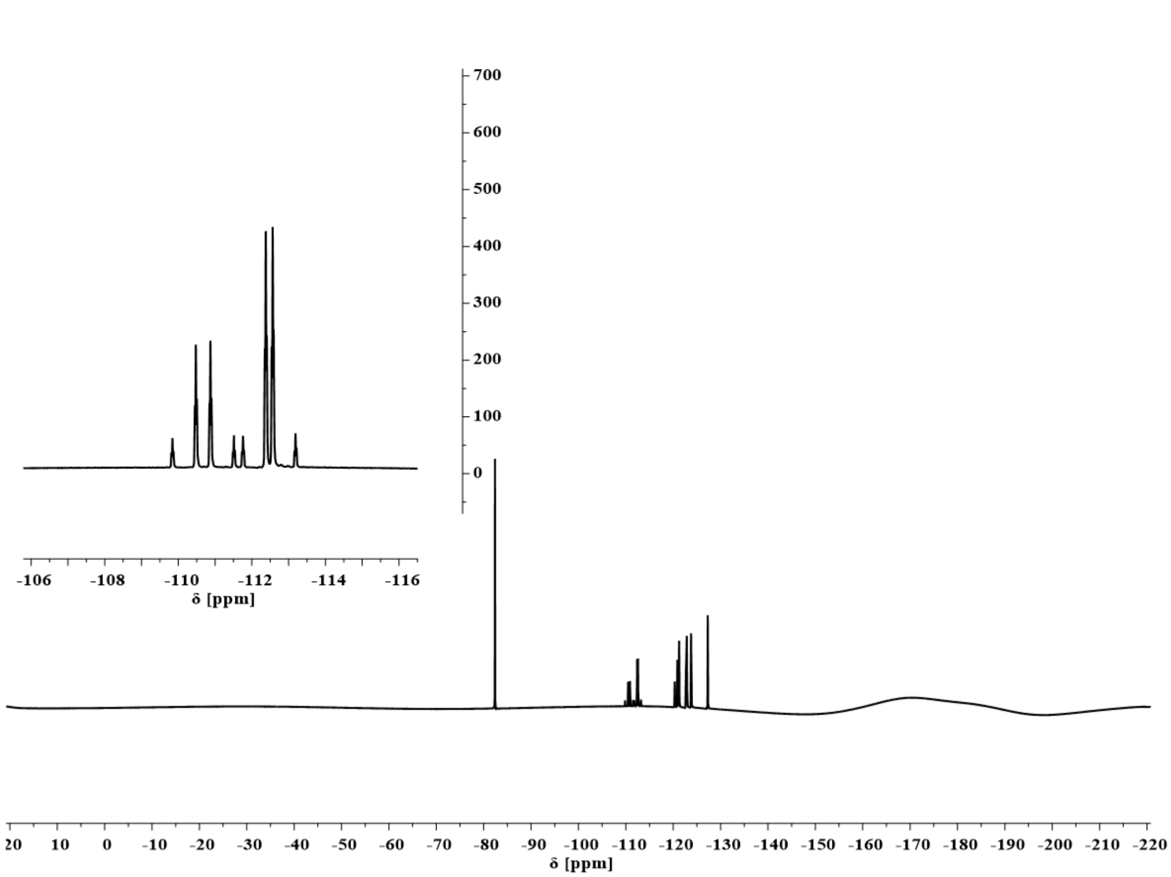

Supplementary Figure 124 | ^{19}F NMR experiment of the title compound recorded in CD_3OD.

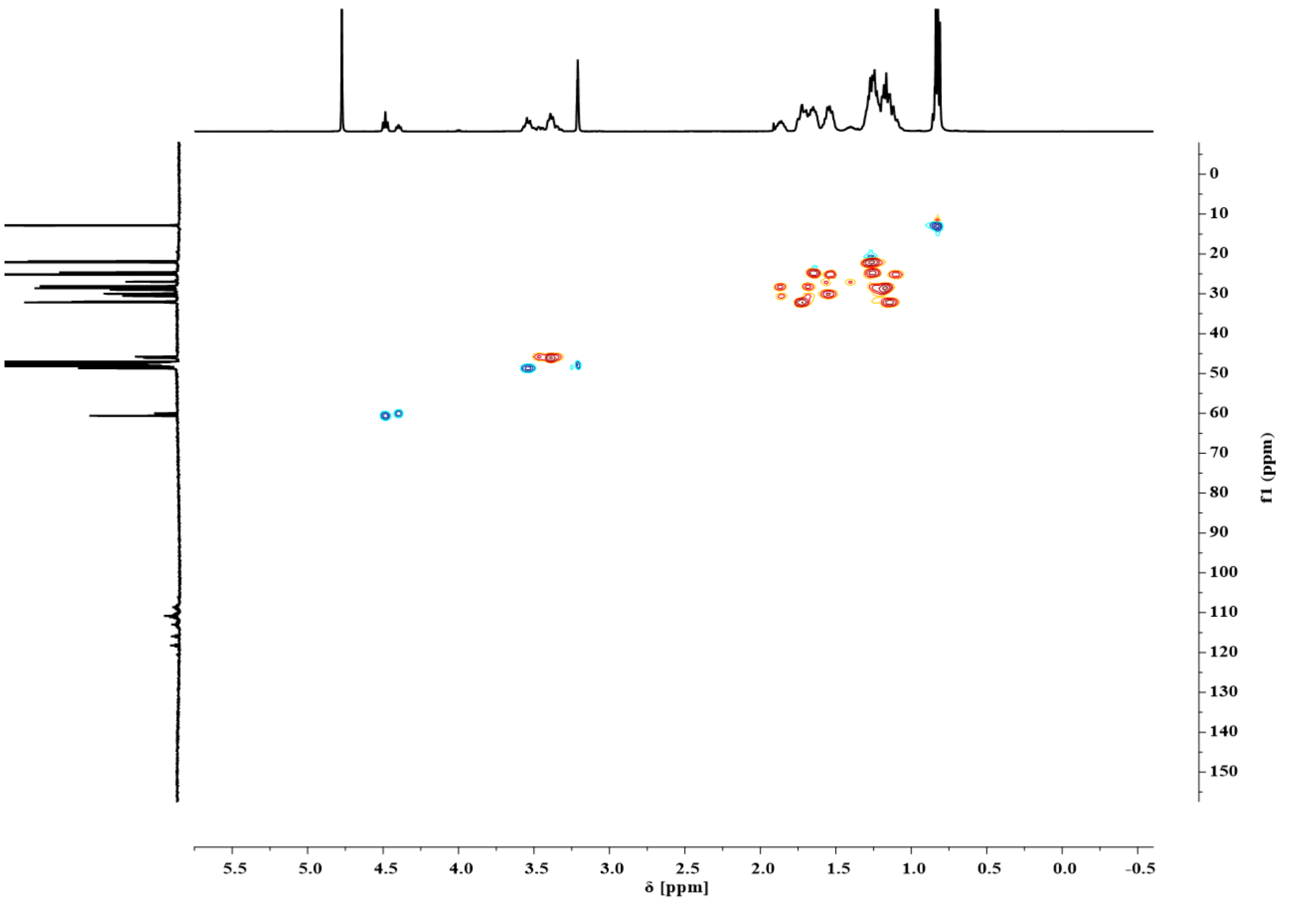

Supplementary Figure 125 | Multiplicity-edited HSQC experiment of the title compound recorded in CD_3OD.

Ugi reaction of perfluorononanoic acid, cyclohexancarboxaldehyde, cyclohexylisocyanide and pentylamine

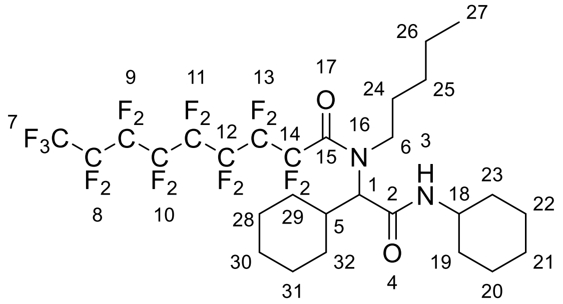

In a 25 mL round bottom flask cyclohexancarboxaldehyde (59.4 µL, 55.0 mg, 490 µmol, 1.30 eq.) was dissolved in 1.5 mL methanol, subsequently pentylamine (56.6 µL, 42.7 mg, 490 µmol, 1.30 eq.) was added and the resulting mixture was stirred for 60 min over sodium sulfate. Afterwards, the mixture was filtrated and the solid was washed with 10 mL methanol three times. Subsequently, the filtrate was concentrated under reduced pressure. Perfluorononanoic acid (175 mg, 377 µmol, 1.00 eq.) dissolved in 1 mL methanol was added to the imine at room temperature and the resulting mixture was stirred for 2 min. Subsequently, cyclohexylisocyanide (56.3 µL, 59.4 mg, 453 µmol, 1.20 eq.)

was added to the stirring mixture. The reaction was stirred for 4 d at room temperature. The crude reaction mixture was dried under reduced pressure and purified *via* column chromatography employing Fluoro*Flash*® silica gel. The fluorous fraction was tested for purity *via* TLC and concentrated under reduced pressure. The remaining perfluoro acid was removed with a short silica gel filter column, eluting with *c*-hexane/ethyl acetate (3:1) to yield the Ugi product as a colorless solid (103 mg, 137 µmol, 36.3%).

R_f = 0.45 in *c*-hexane/ethyl acetate (6:1). Visualized *via* Seebach staining solution and permanganate staining.

IR (ATR): ν [cm^{-1}] = 3317.7 (br, ν(N-H)), 2930.9 (s, ν(C-H)), 2856.6 (m, ν(C-H)), 1653.4 (s, ν(C=O)), 1536.7 (m), 1451.4 (m), 1351.0 (m), 1237.9 (vs), 1203.5 (vs), 1147.1 (vs), 1117.8 (s), 1053.5 (m), 962.2 (m), 891.1 (w), 777.5 (m), 735.4 (m), 703.4 (m), 656.3 (m), 557.6 (m), 528.6 (m).

^{1}H NMR (400 MHz, CD_3OD): δ [ppm] = 4.47 (d, J = 11.0 Hz, CH^{1a}), 4.19 – 3.90 (m, total integral of CH^1 = 1 H, CH^{1b}), 3.71 – 3.35 (m, 3 H, CH_2 + CH^{18}), 2.24 – 1.95 (m, 1 H, CH^5), 1.96 – 1.46 (m, 12 H, CH_2), 1.41 – 1.01 (m, 14 H, CH_2), 0.92 (t, J = 7.1 Hz, 3 H, CH_3^{27}).

^{13}C NMR (101 MHz, CD_3OD): δ [ppm] = 169.9 (s, $CONR^2$), 160.3 (s, $CONR^{15}$), 66.5 (s, CH^{1a}), 65.7 (s, CH^{1b}), 49.9 (s, CH^{18}), 46.3 (s, CH_2), 39.0 (s, CH^{5a}), 37.6 (s, CH^{5b}), 33.5 (s, CH_2), 33.3 (s, CH_2), 31.4 (s, CH_2), 31.1 (s, CH_2), 30.8 (s, CH_2), 30.1 (s, CH_2), 27.3 (s, CH_2), 26.6 (s, CH_2), 25.9 (s, CH_2), 23.2 (s, CH_2), 14.2 (s, CH_3^{27}).

^{19}F NMR (376 MHz, CD_3OD): δ [ppm] = -88.27 (t, J = 10.5 Hz, 3 F, CF_3^7), -115.58 (t, J = 10.8 Hz, CF_2^{14a}), AB-signal (δ_A = -117.67, δ_B = -118.82, J_{AB} = 293.6 Hz, A and B are split into t, J = 12.0 Hz, CF_2^{14b}), -125.59 (s, CF_2), -126.65 (s, CF_2), -126.92 (s, CF_2), -128.72 (s, CF_2), -129.66 (s, CF_2), -133.20 (s, CF_2^8). Total integral of CF_2 region normalized with respect to the CF_3^7 group = 14.

FAB – MS [*m/z*] (relative intensity): 755.3 (67%) $[M + H]^+$, 629.2 (27%) [Fragment A + H]$^+$, 307.3 (8%) [Fragment B]$^+$, 223.2 (23%) [Fragment C + H]$^+$.

HRMS – FAB [*m/z*]: $[M + H]^+$ calculated for $^{12}C_{28}{}^1H_{36}{}^{16}O_2{}^{14}N_2{}^{19}F_{17}$, 755.2501; found, 755.2500; Δ = 0.14 mmu.

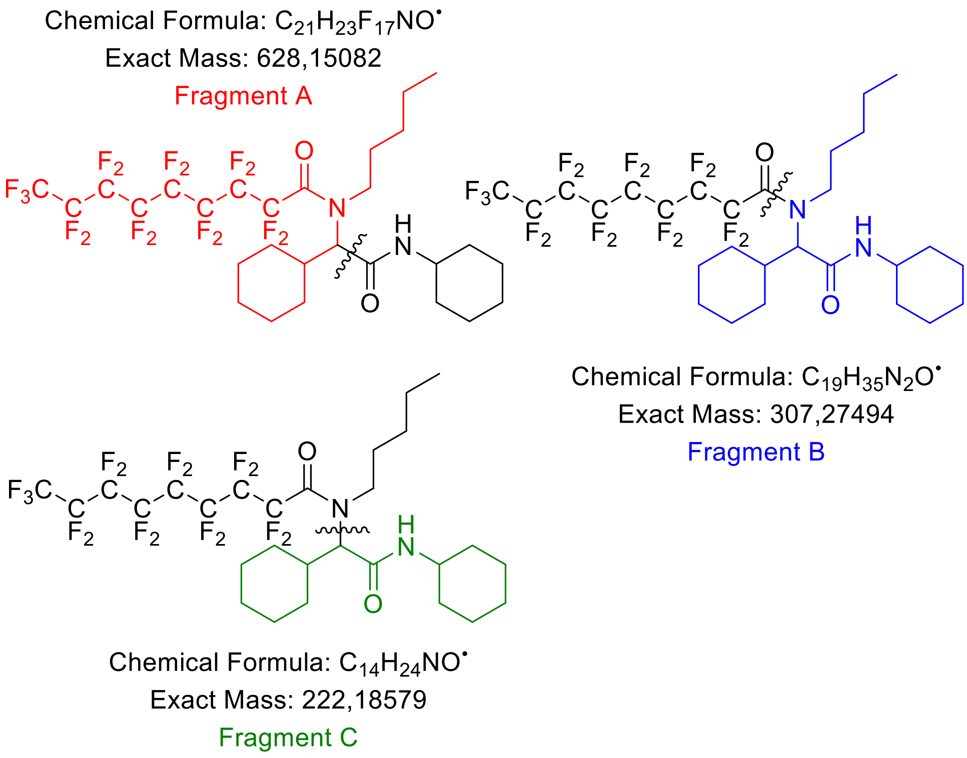

Supplementary Figure 126 | Proposed fragments observed in FAB-MS.

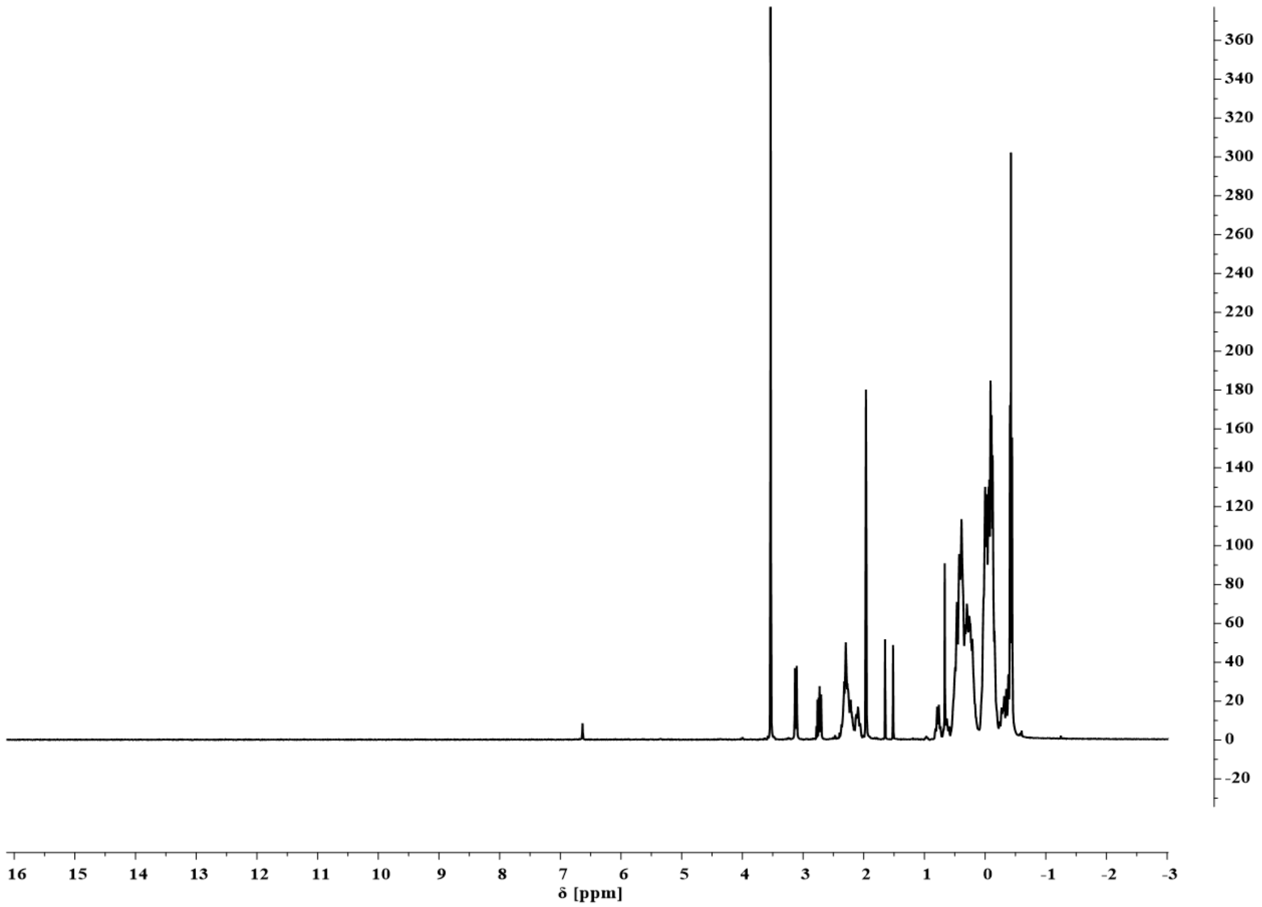

Supplementary Figure 127 | ^{1}H NMR experiment of the title compound recorded in CD_3OD.

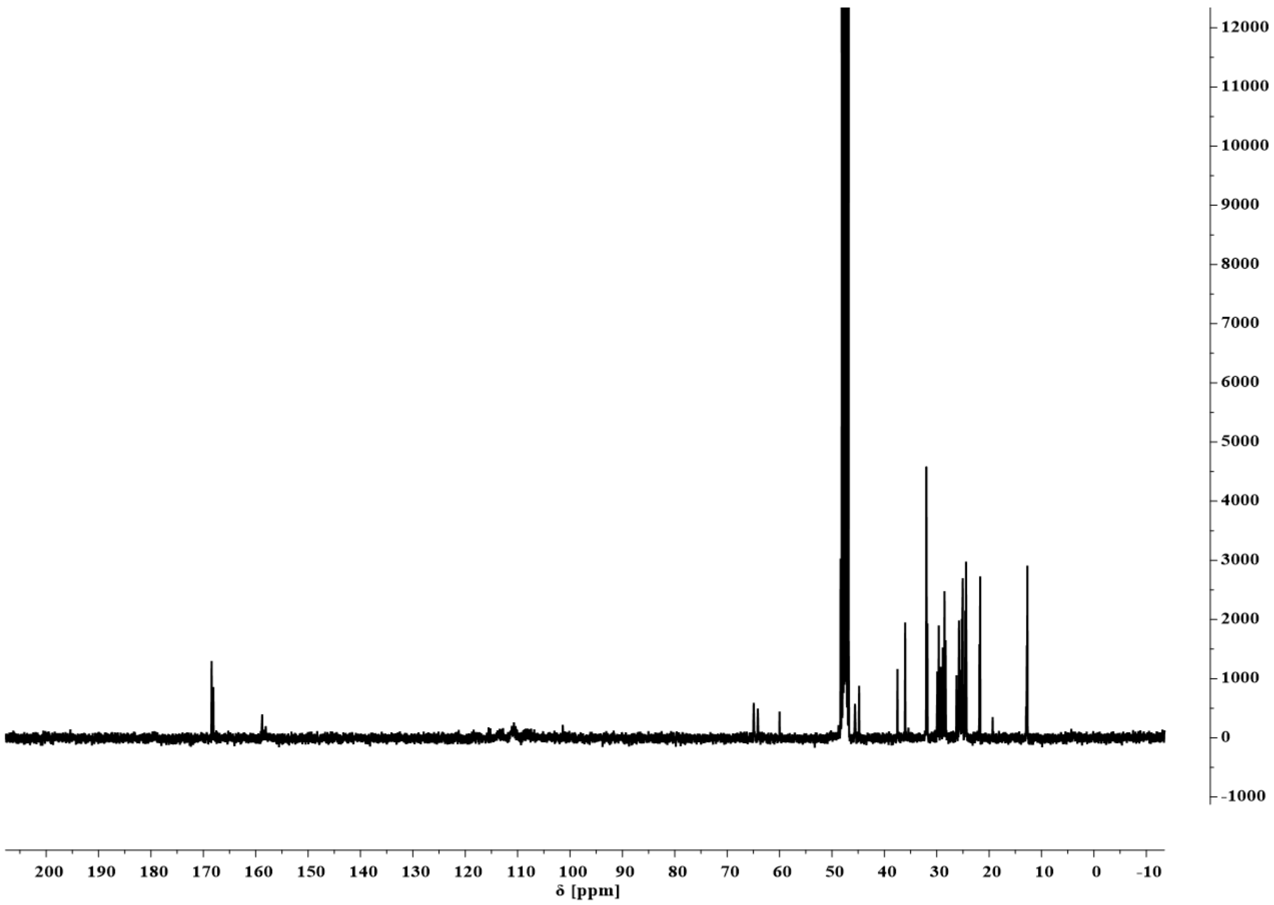

Supplementary Figure 128 | ^{13}C NMR experiment of the title compound recorded in CD_3OD.

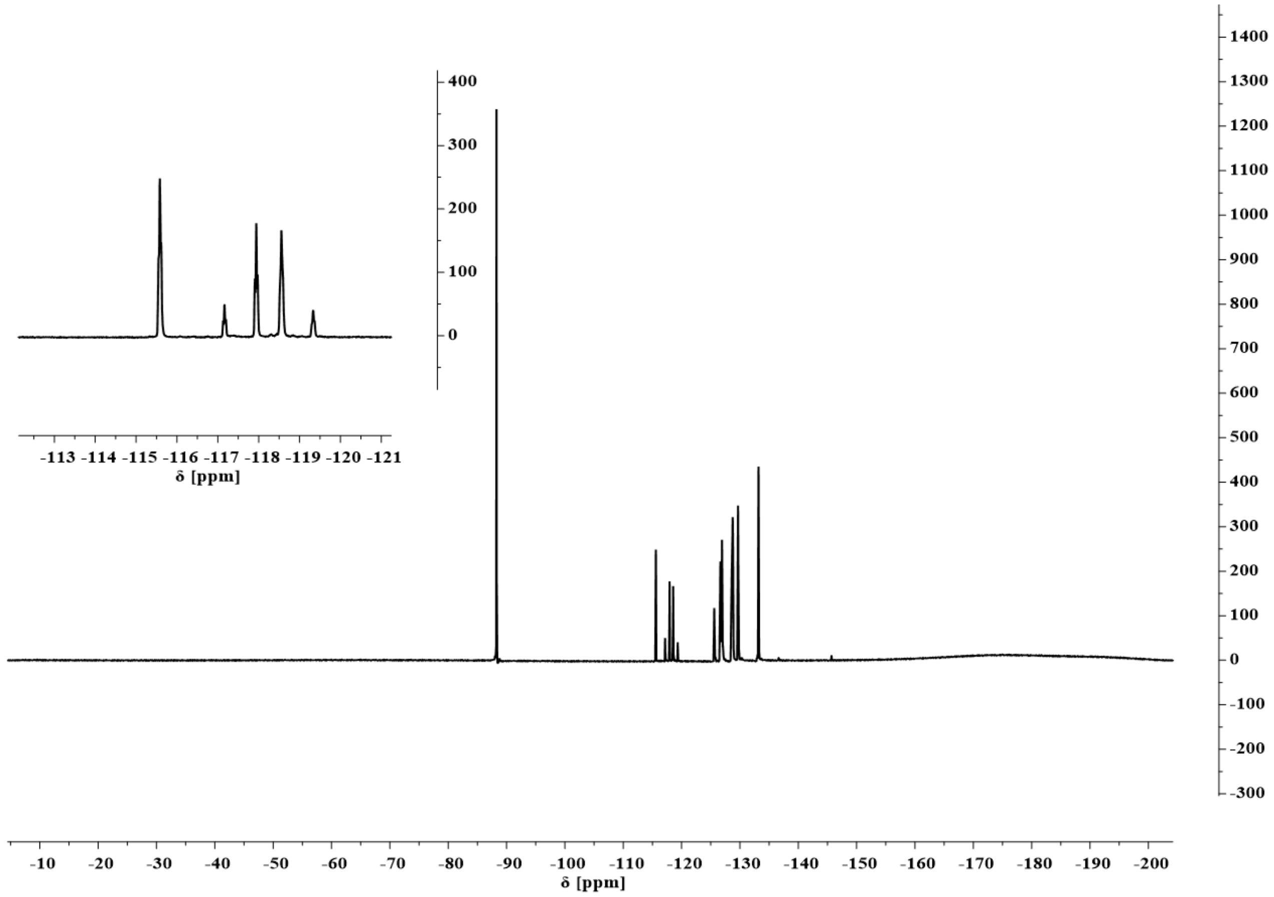

Supplementary Figure 129 | ^{19}F NMR experiment of the title compound recorded in CD_3OD.

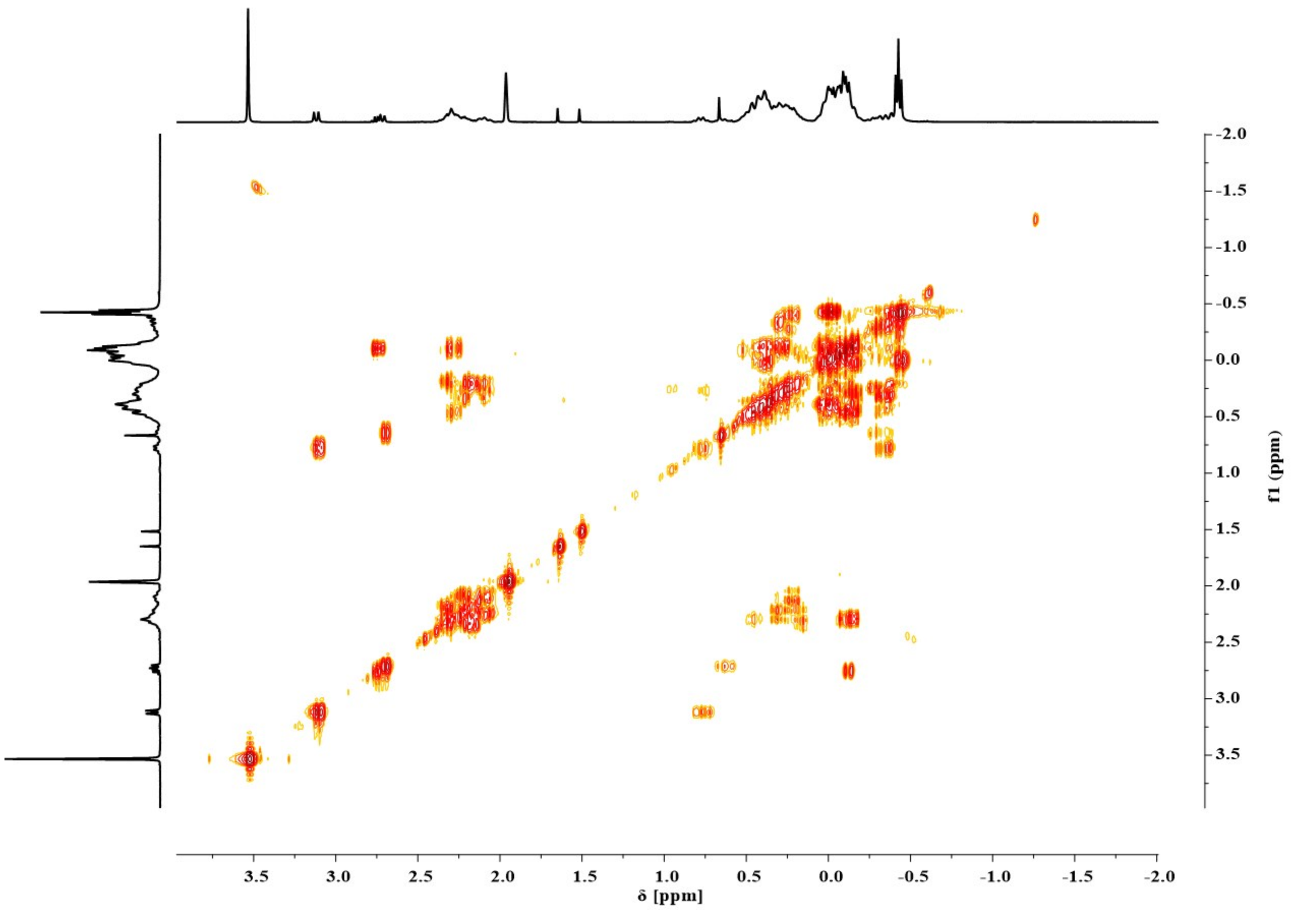

Supplementary Figure 130 |COSY experiment of the title compound recorded in CD_3OD.

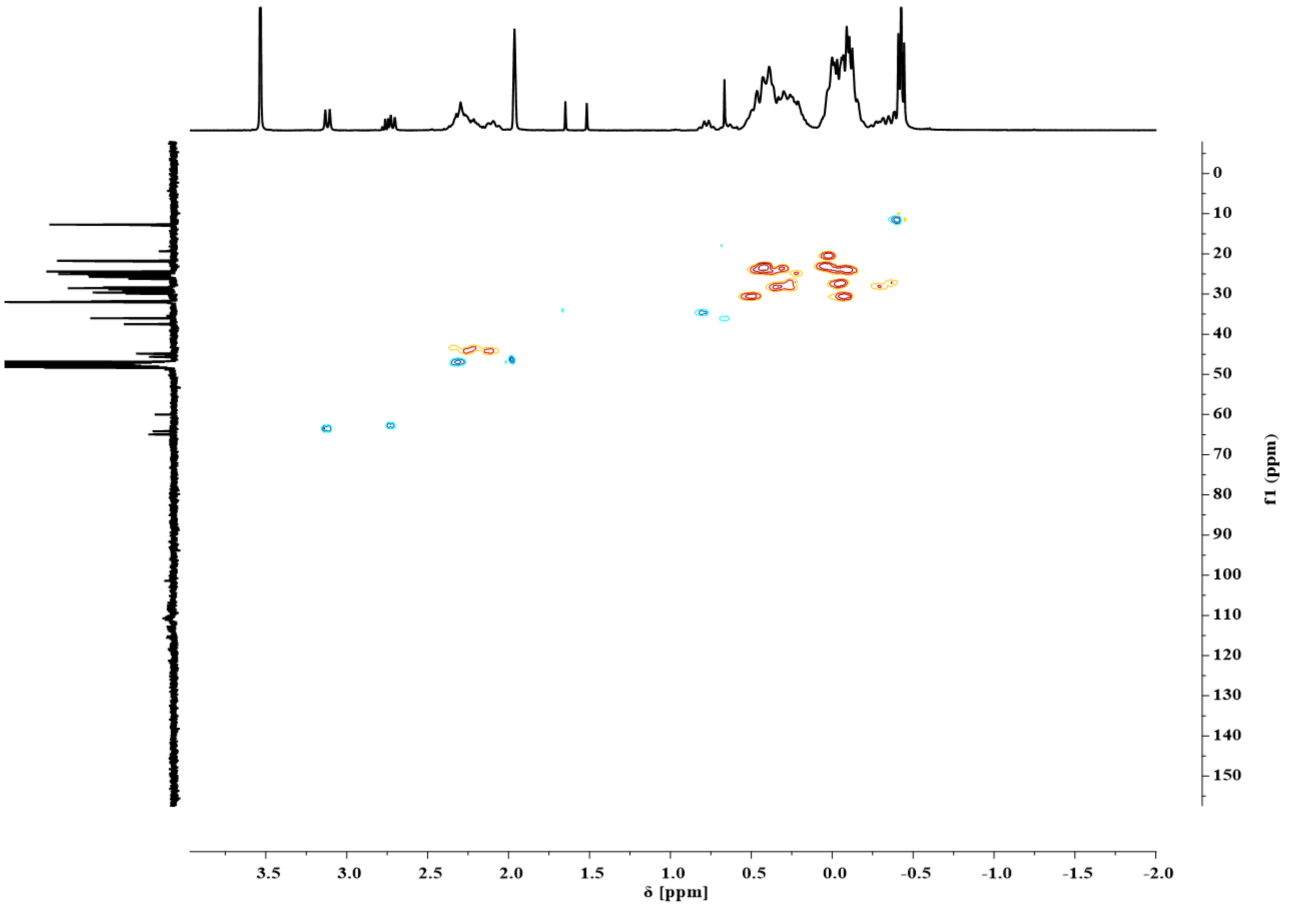

Supplementary Figure 131 | Multiplicity-edited HSQC experiment of the title compound recorded in CD_3OD.

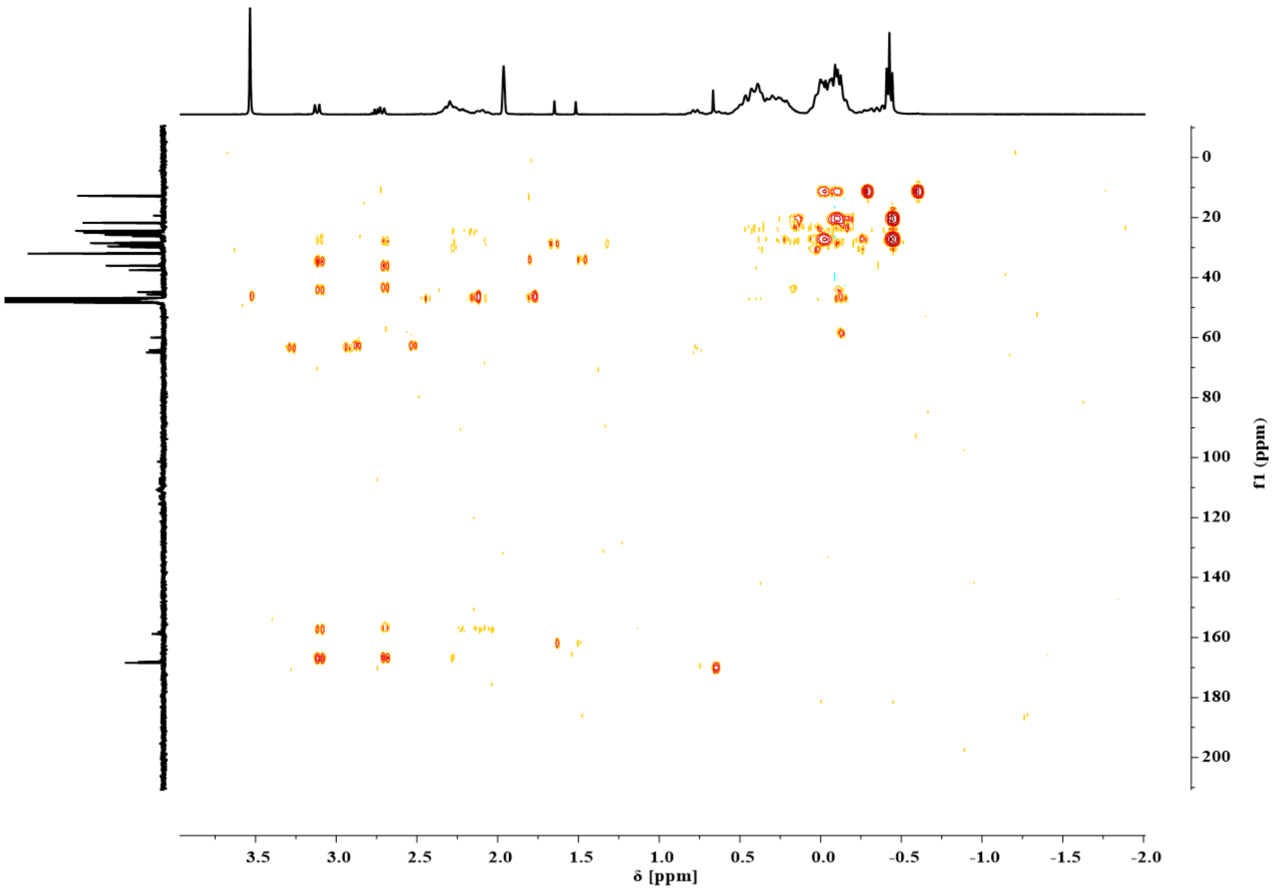

Supplementary Figure 132 | HMBC experiment of the title compound recorded in CD_3OD.

Ugi reaction of perfluorononanoic acid, isobutyraldehyde, *tert*-butylisocyanide and cyclohexylamine

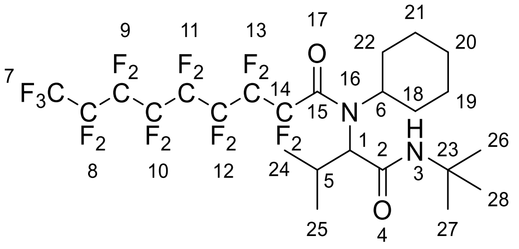

In a 25 mL round bottom flask isobutyraldehyde (83.6 µL, 66.0 mg, 916 µmol, 1.70 eq.) and cyclohexylamine (105 µL, 90.8 mg, 916 µmol, 1.70 eq.) were added and the resulting mixture was stirred for 60 min over sodium sulfate. Perfluorononanoic acid (250 mg, 539 µmol, 1.00 eq.) dissolved in 0.5 mL methanol was added to the solution at room temperature and the resulting mixture was stirred for 2 min. Subsequently, *tert*-butylisocyanide (104 µL, 76.1 mg, 916 µmol, 1.70 eq.) was added to the stirring mixture. The reaction was stirred for 3 d at room temperature. The crude reaction mixture was dried under reduced pressure. The residue was adsorbed onto celite® and purified *via* column chromatography on silica gel eluting with a gradual solvent mixture of ethyl acetate and *c*-hexane (1:10 → 1:3) to yield the Ugi product as a yellow solid (75.0 mg, 102 µmol, 19.1%).

R_f = 0.54 in *c*-hexane/ethyl acetate (6:1). Visualized *via* permanganate staining solution.

IR (ATR): ν[cm^{-1}] = 2927.2 (vs, ν(C-H)), 2854.9 (s, ν(C-H)), 1673.1 (s, ν(C=O)), 1596.0 (m), 1539.9 (m), 1450.7 (s), 1367.1 (m), 1349.4 (m), 1232.6 (vs), 1148.6 (s), 1130.7 (s), 991.2 (m), 890.6 (w), 802.1 (m), 721.0 (w), 701.0 (m), 660.6 (w), 553.3 (m).

^{1}H NMR (400 MHz, CD_3OD): δ [ppm] = 3.75 (s, CH^{1a}), 3.66 – 3.50 (m, 1 H, CH^6), 3.40 – 3.33 (m, total integral of CH^1 = 1 H, CH^{1b}), 2.38 – 1.98 (m, 1 H, CH^5), 1.95 – 1.49 (m, 8 H, CH_2), 1.47 – 1.11 (m, 11 H, CH_2 +$CH_3^{26,27,28}$), 1.04 – 0.79 (m, 6 H, $CH_3^{24,25}$).

^{13}C NMR (101 MHz, CD_3OD): δ [ppm] =170.2 (s, CONR^2), 164.1 (s, CONR^{15}), 57.5 (s, CH^1), 56.3 (s, CH^6), 54.8 (s, C^{23}), 35.0 (s, CH^1), 34.4 (s, CH_2), 31.7 (s, CH_2), 31.6 (s, CH_2), 30.8 (s, CH_2), 29.2 (s, CH^5), 25.5 (s, CH_3^{26-28}), 25.3 (s, CH_3^{26-28}), 22.2 (s, CH_2), 18.7 (s, $CH_3^{24,25}$), 18.5 (s, $CH_3^{24,25}$).

^{19}F NMR (376 MHz, CD_3OD): δ [ppm] = -88.25 (t, *J* = 10.4 Hz, 3 F, CF_3^7), AB-signal (δ_A = -116.39, δ_B = -117.11, J_{AB} = 301.2 Hz, A and B are split into t, *J* = 11.0 Hz, CF_2^{14a}), AB-signal (δ_A = -118.11, δ_B = -118.55, J_{AB} = 293.6 Hz, A and B are split into t, *J* = 12.3 Hz, CF_2^{14b}), -126.26 (s, CF_2), -126.73 (s, CF_2), -127.10 (s, CF_2), -128.75 (s, CF_2), -129.64 (s, CF_2), -133.22 (s, CF_2^8). Total integral of CF_2 region normalized with respect to the CF_3^7 group = 14.

ESI-MS [*m/z*]: $[M + Na]^+$ calculated for $^{12}C_{24}{}^1H_{29}{}^{16}O_2{}^{14}N_2{}^{19}F_{17}{}^{23}Na$, 723.18498; found, 723.18591, Δ = 1.02 mmu.

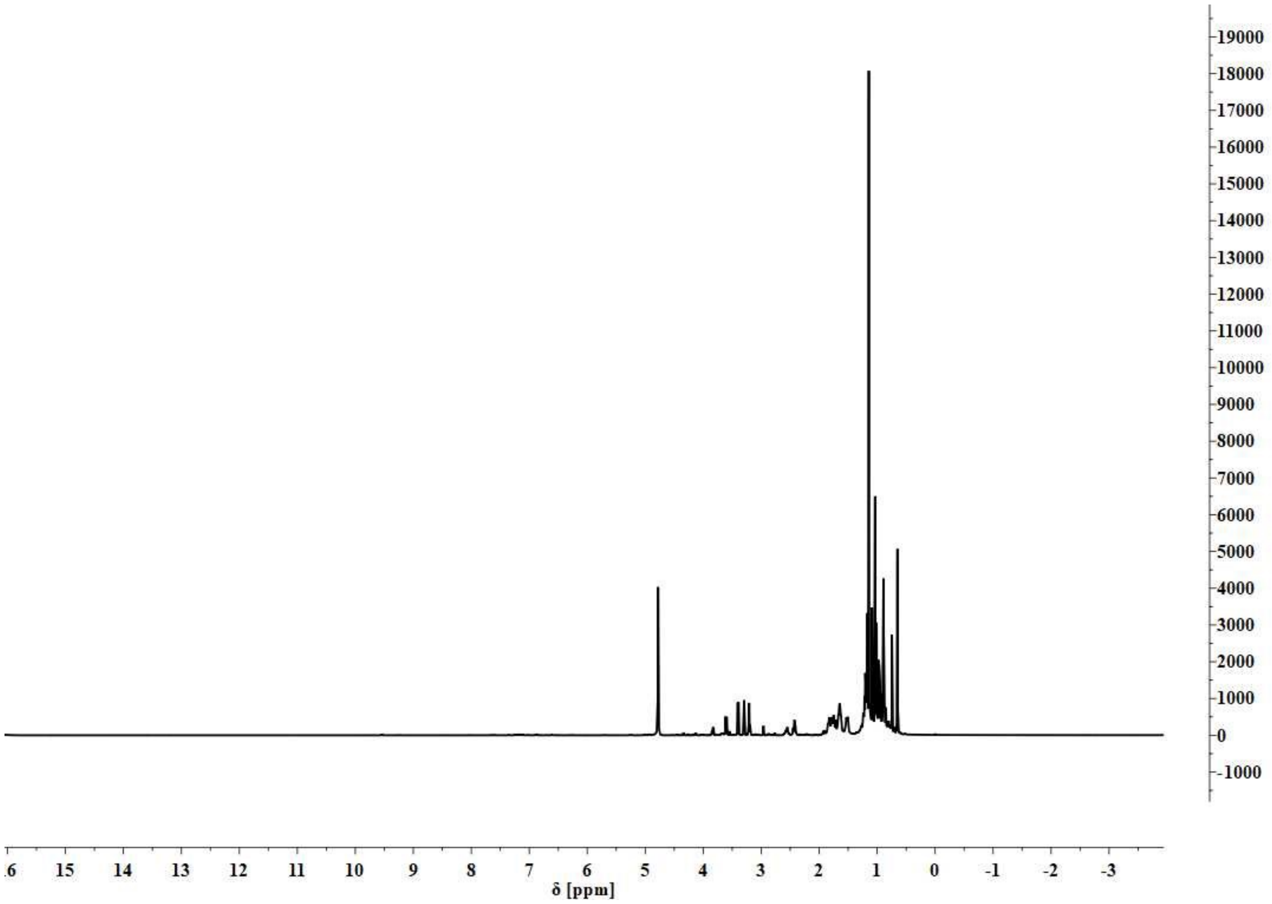

Supplementary Figure 133 | ^{1}H NMR experiment of the title compound recorded in CD_3OD.

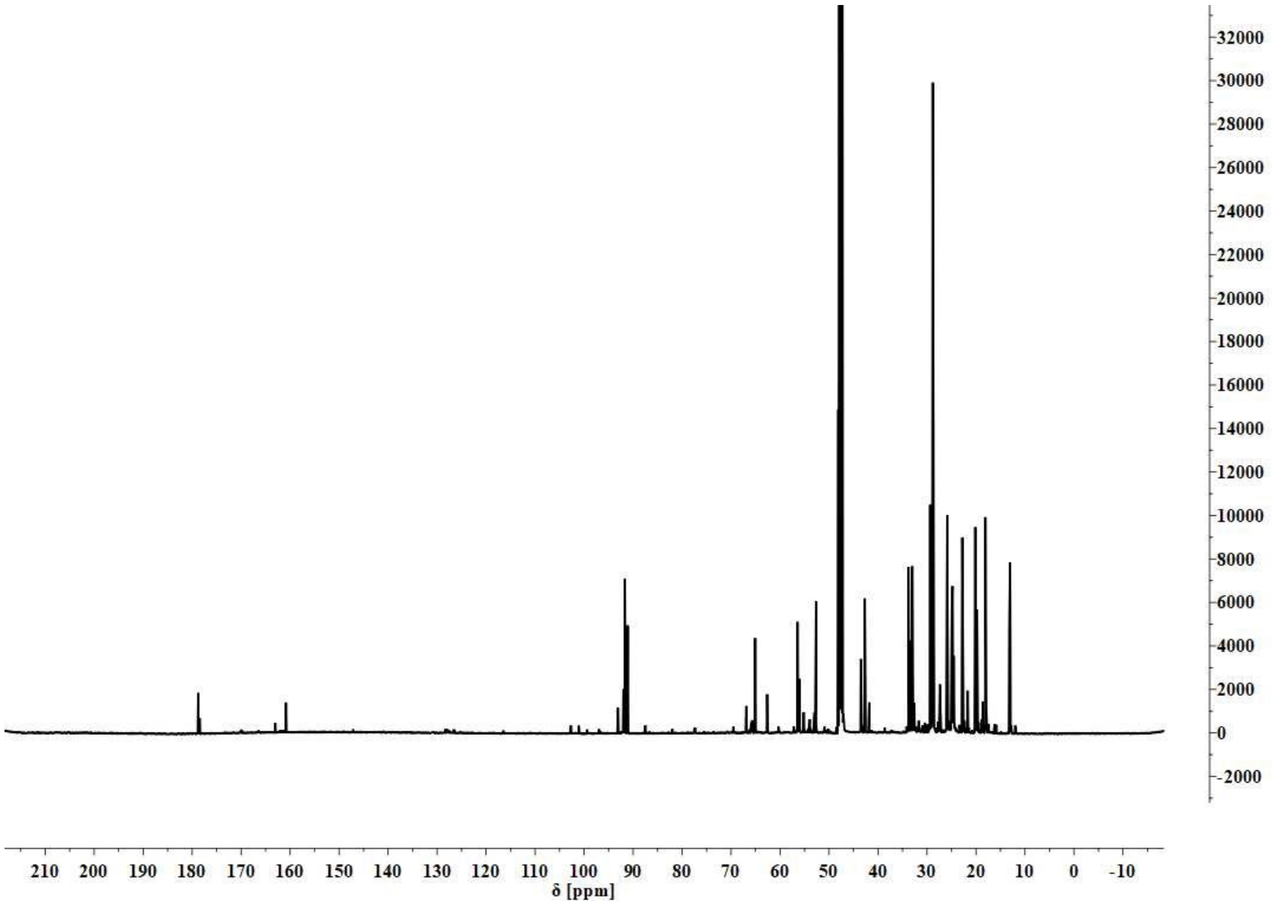

Supplementary Figure 134 | ^{13}C NMR experiment of the title compound recorded in CD_3OD.

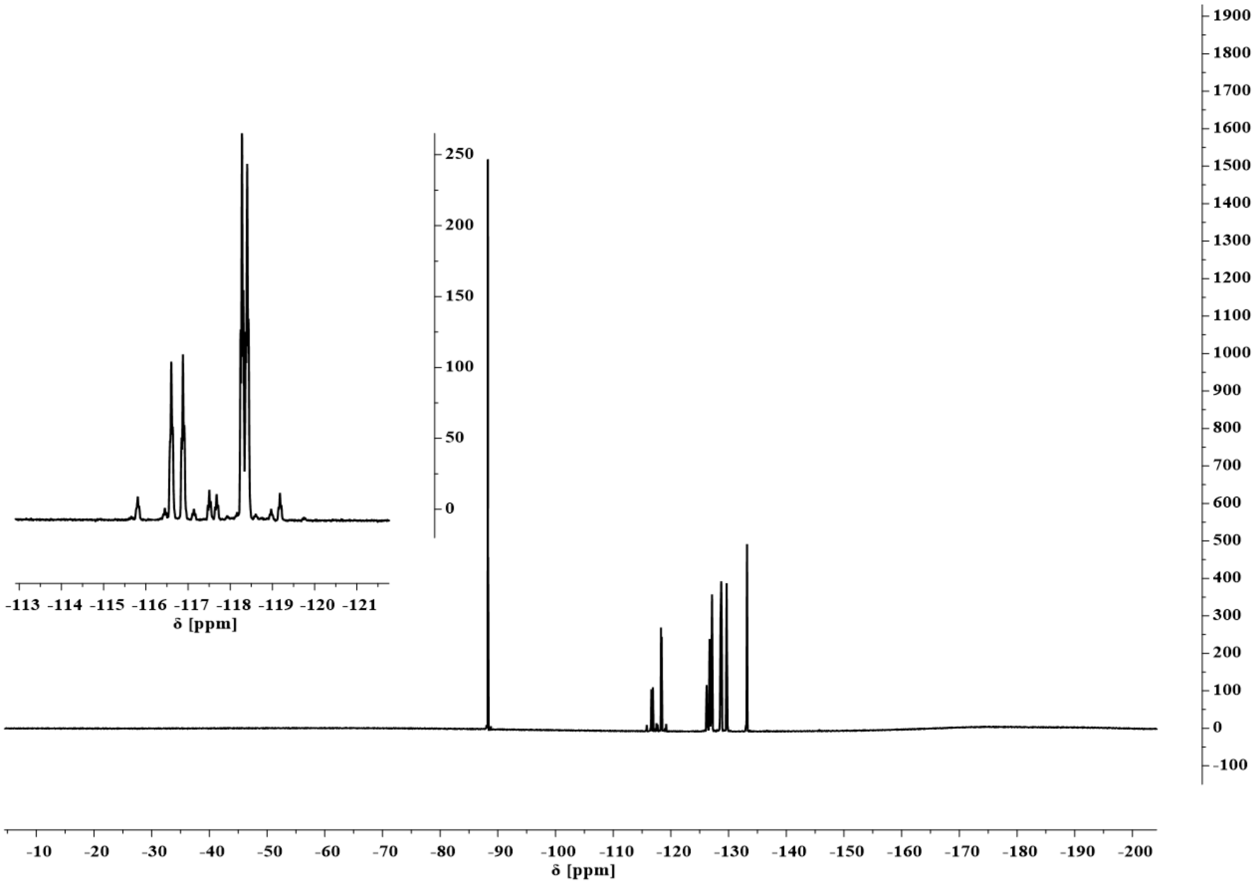

Supplementary Figure 135 | ^{19}F NMR experiment of the title compound recorded in CD_3OD.

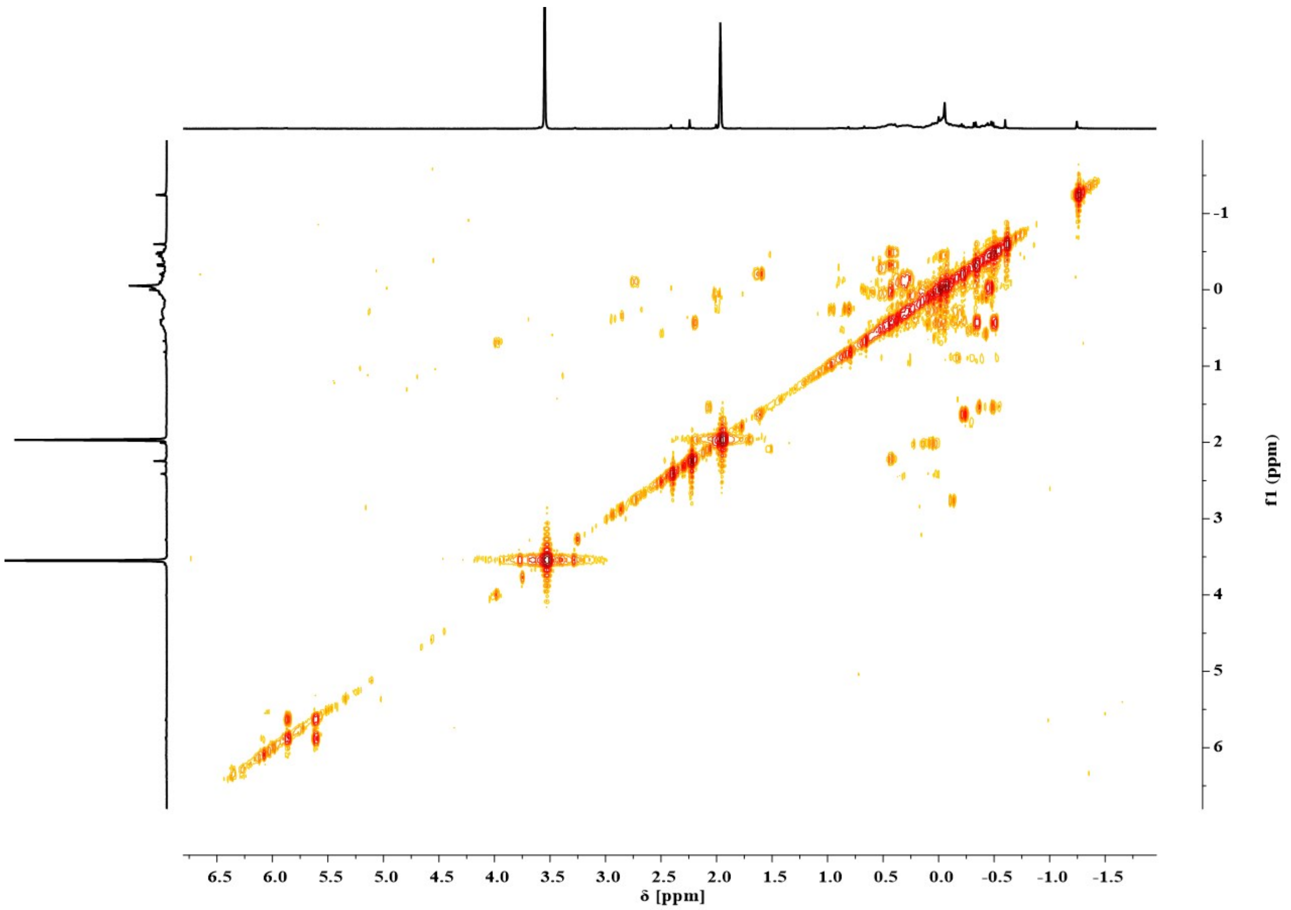

Supplementary Figure 136 | COSY experiment of the title compound recorded in CD_3OD.

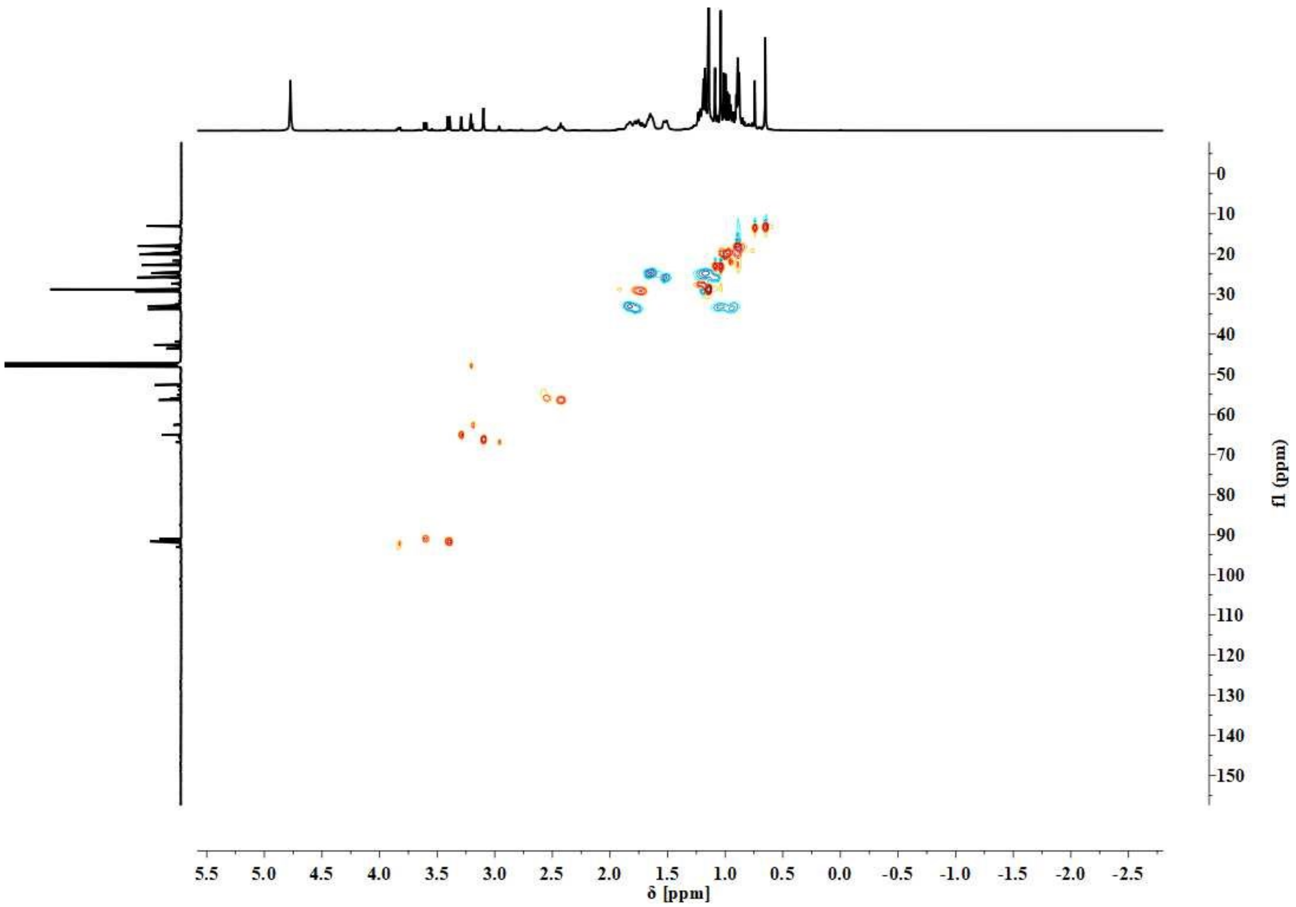

Supplementary Figure 137 | Multiplicity-edited HSQC experiment of the title compound recorded in CD_3OD.

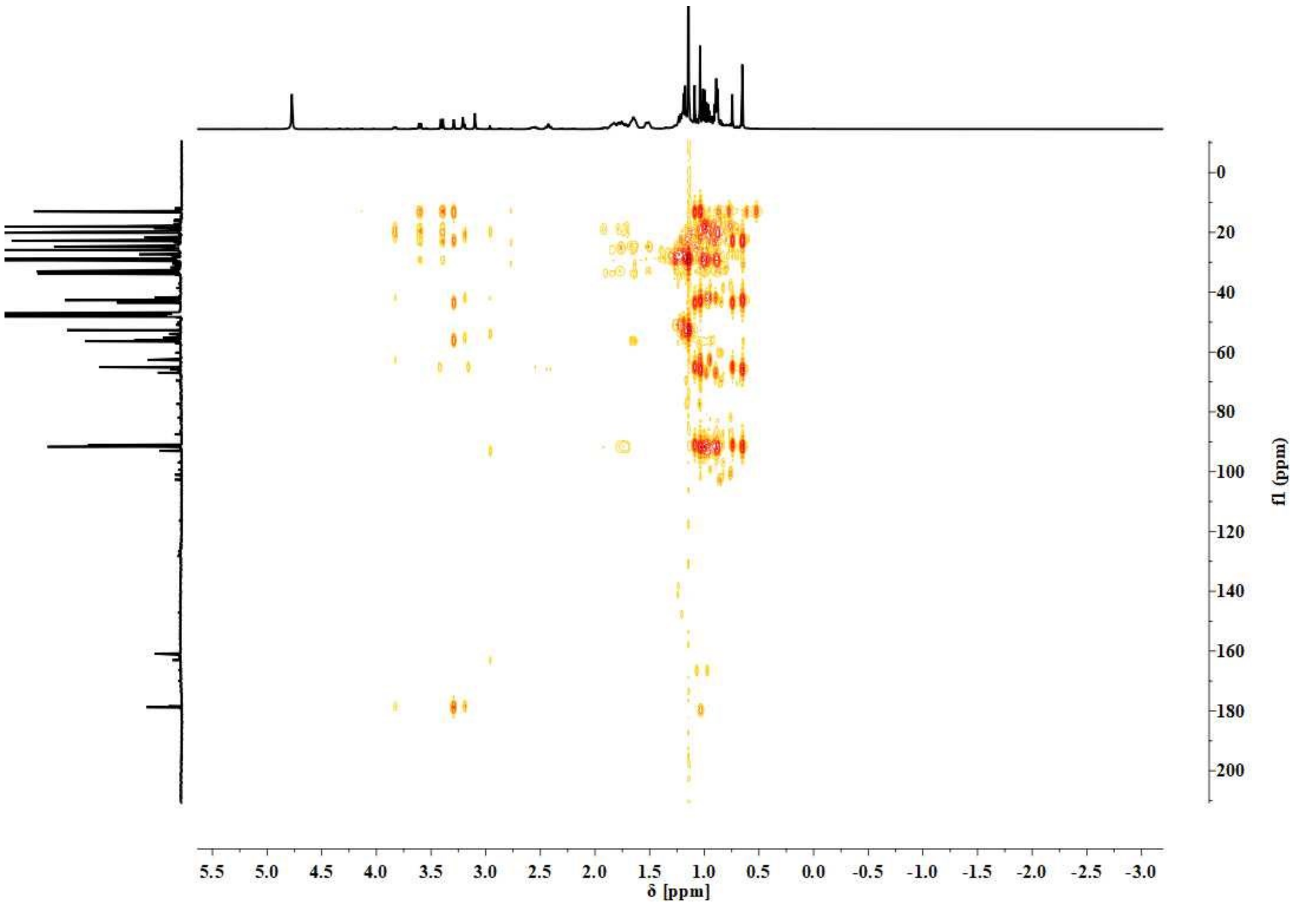

Supplementary Figure 138 | HMBC experiment of the title compound recorded in CD_3OD.

Ugi reaction of perfluorononanoic acid, isobutyraldehyde, pentylisocyanide and cyclohexylamine

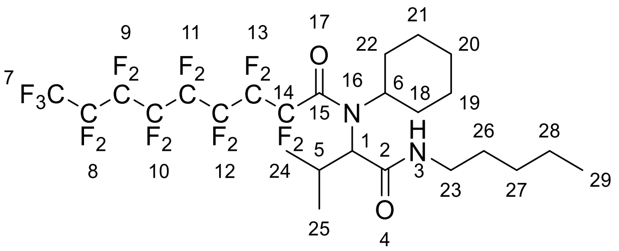

In a 25 mL round bottom flask isobutyraldehyde (46.2 mg, 641 µmol, 1.70 eq.) was dissolved in 1.5 mL methanol, subsequently cyclohexylamine (674 µL, 63.6 mg, 641 µmol, 1.70 eq.) was added and the resulting mixture was stirred for 60 min over sodium sulfate. Afterwards, the mixture was filtrated and the solid was washed with 10 mL methanol three times. Subsequently, the filtrate was concentrated under reduced pressure. Perfluorononanoic acid (175 mg, 377 µmol, 1.00 eq.) dissolved in 0.5 mL methanol was added to the imine at room temperature and the resulting mixture was stirred for 2 min. Subsequently, pentylisocyanide (80.6 µL, 62.2 mg, 641 µmol, 1.70 eq.) was added to the stirring mixture. The reaction was stirred for 6 d at room temperature. The crude reaction mixture was dried under reduced pressure and purified *via* column chromatography employing Fluoro*Flash*® silica gel. The fluorous fraction was tested for purity *via* TLC and concentrated under reduced pressure. The remaining perfluoro acid was removed with a short silica gel filter column, eluting with *c*-hexane/ethyl acetate (3:1) to yield the Ugi product as a highly viscous yellow oil (9.1 mg, 12.6 µmol, 3.34%).

R_f = 0.54 in *c*-hexane/ethyl acetate (6:1). Visualized *via* permanganate staining solution.

IR (ATR): ν [cm^{-1}] = 3338.2 (br, ν(N-H)), 2930.9 (s, ν(C-H)), 2859.9 (s, ν(C-H)), 1659.8 (s, ν(C=O)), 1540.8 (m), 1457.7 (m), 1369.4 (m), 1238.5 (vs), 1206.8 (vs), 1148.7 (vs), 998.0 (m), 777.8 (w), 735.1 (m), 703.1 (m), 656.7 (m), 558.1 (m), 528.8 (w).

^1H NMR (400 MHz, CD_3OD): δ [ppm] = 4.42 (d, J = 11.0 Hz, CH^{1a}), 4.10 (d, J = 7.1 Hz, total integral of CH^{1} = 1 H, CH^{1b}), 3.73 – 3.36 (m, 1 H, CH_2^{23a}), 3.22 – 3.04 (m, 1 H, CH_2^{23b}), 2.51 – 2.12 (m, 1 H, CH^{6}), 1.93 – 1.49 (m, 6 H, CH_2), 1.46 (s, 1 H, CH^{5}), 1.38 – 1.14 (m, 10 H, CH_2), 1.05 – 0.84 (m, 9 H, $CH_3^{24,25}$+ CH_3^{29}).

^{13}C NMR (101 MHz, CD_3OD): δ [ppm] = 170.9 (s, $CONR^{2}$), 67.4 (s, CH^{1}), 46.1 (s, CH_2^{23a}), 40.4 (s, CH_2^{23b}), 31.4 (s, CH_2), 30.9 (s, CH_2), 30.1 (s, CH_2), 30.0 (s, CH^{5}), 29.5 (s, CH_2), 28.2 (s, CH_2), 23.2 (s, CH_2), 20.9 (s, CH^{6}), 19.9 (s, $CH_3^{24, 25}$), 18.7 (s, $CH_3^{24, 25}$), 14.3 (s, CH_3^{29}).

^{19}F NMR (376 MHz, CD_3OD): δ [ppm] = -83.25 (s, CF_3), -88.25 (t, J = 10.3 Hz, 3 F, $CF_3{}^{7}$), AB-signal (δ_A = -114.98, δ_B = -115.37, J_{AB} = 207.1 Hz, A and B are split into A and B are split into t, J = 12.0 Hz, $CF_2{}^{14a}$), AB-signal (δ_A = -117,60, δ_B = -119,02, J_{AB} = 293.6 Hz, A and B are split into t, J = 12.6 Hz, $CF_2{}^{14b}$), -125.47 (s, CF_2), -126.86 (s, CF_2), -128.72 (s, CF_2), -129.63(s, CF_2), -133.14(s, $CF_2{}^{8}$). Total integral of CF_2 region normalized with respect to the $CF_3{}^{7}$ group = 14.

ESI-MS [*m/z*]: $[M + Na]^+$ calculated for $^{12}C_{25}{}^{1}H_{31}{}^{16}O_2{}^{14}N_2{}^{19}F_9{}^{23}Na$, 737.2006; found, 737.2013, Δ = 0.66 mmu.

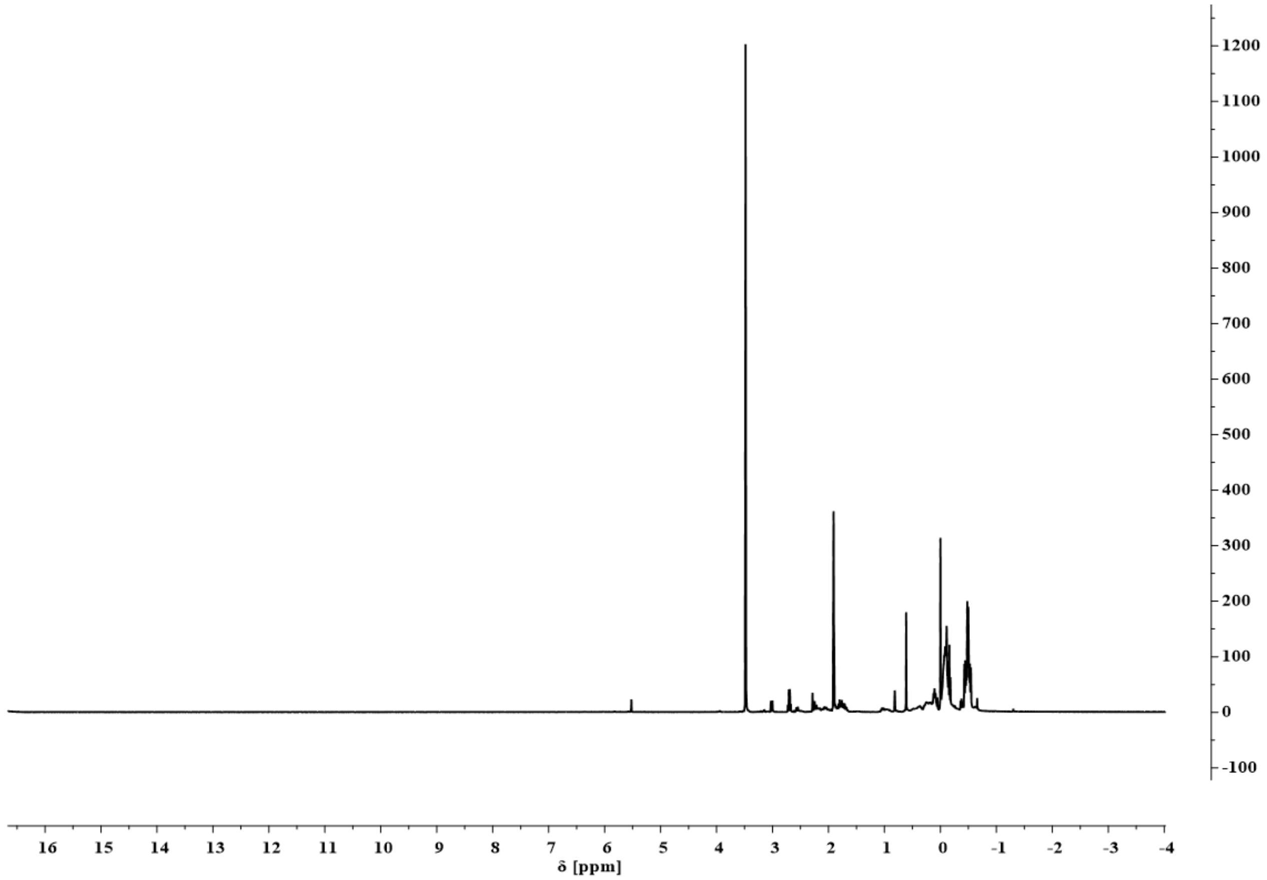

Supplementary Figure 139 | ^1H NMR experiment of the title compound recorded in CD_3OD.

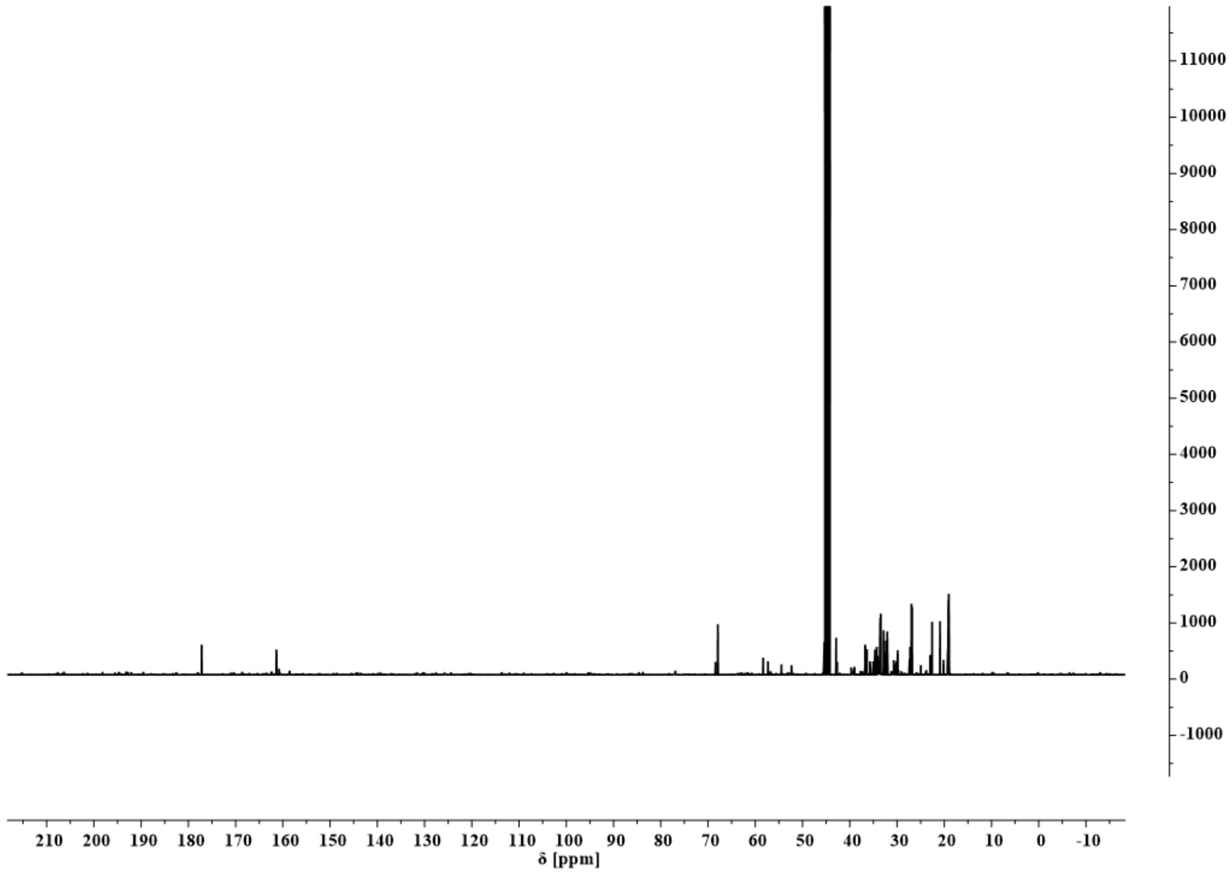

Supplementary Figure 140 | ^{13}C NMR experiment of the title compound recorded in CD_3OD.

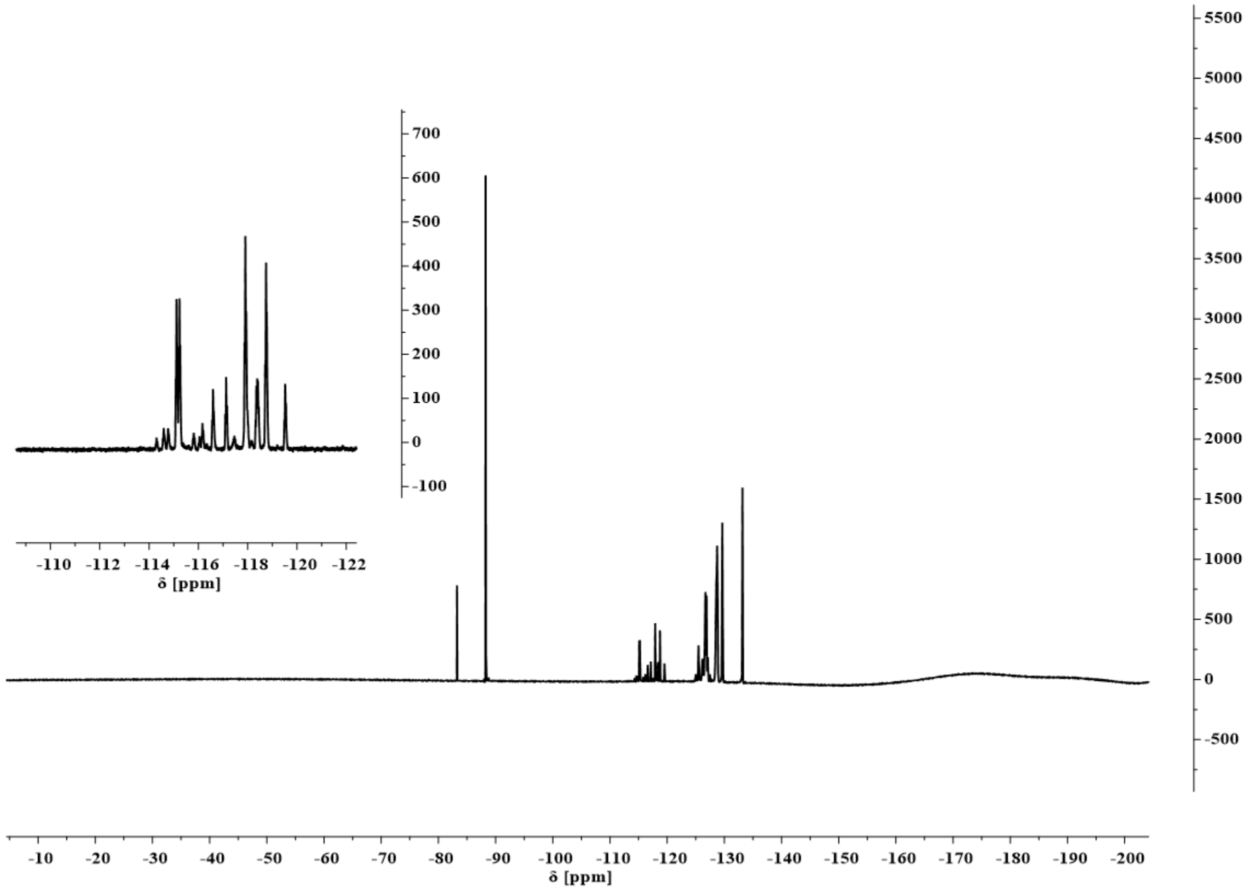

Supplementary Figure 141 | ^{19}F NMR experiment of the title compound recorded in CD_3OD.

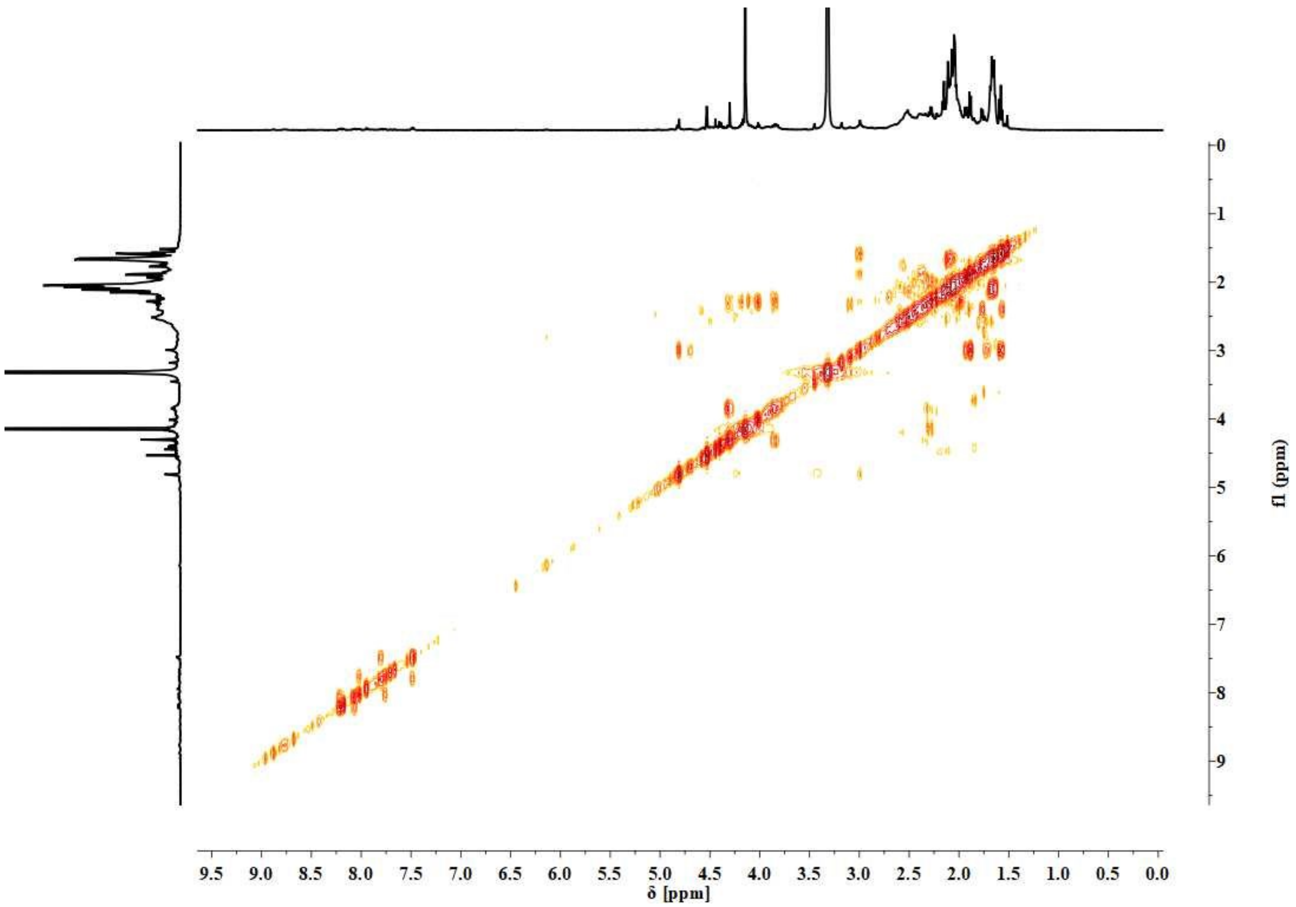

Supplementary Figure 142 | COSY experiment of the title compound recorded in CD_3OD.

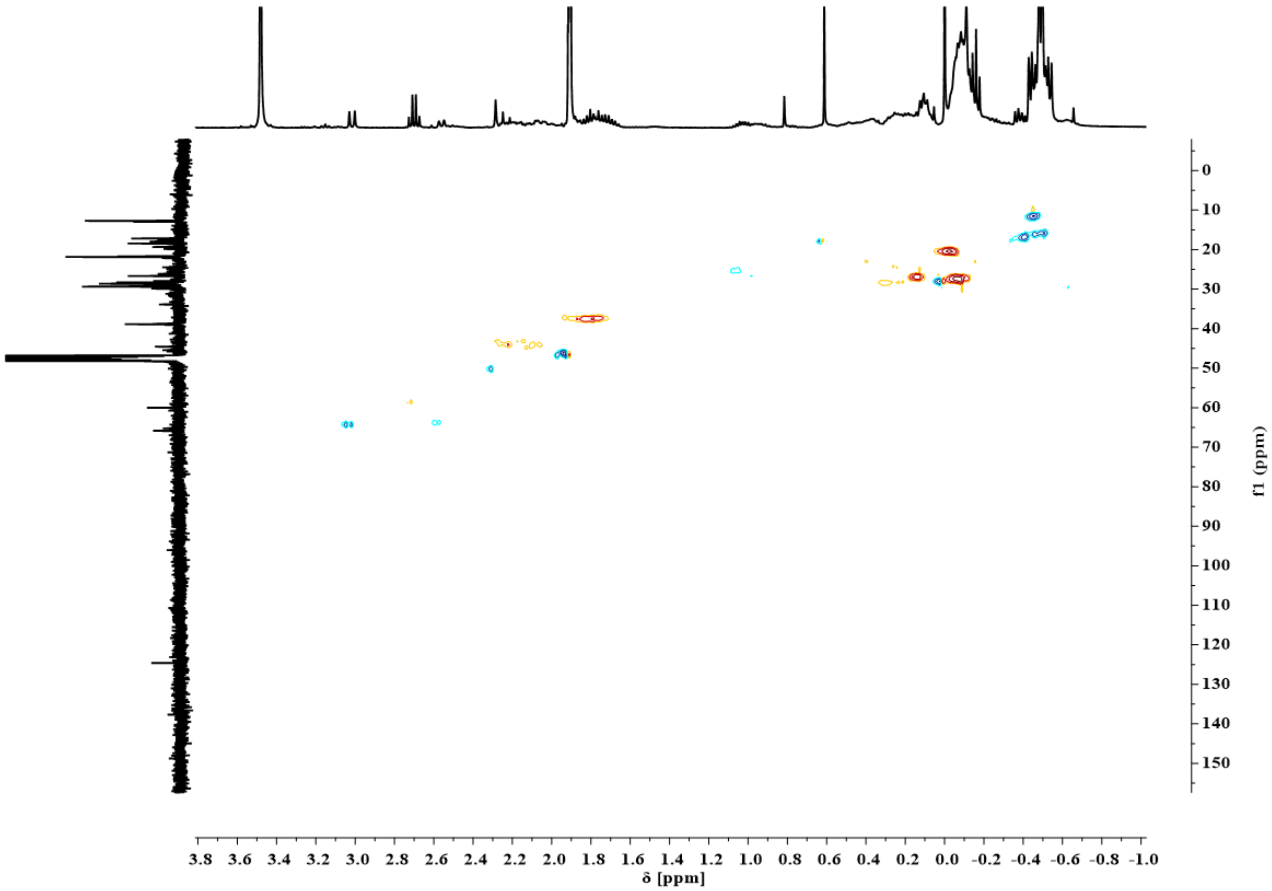

Supplementary Figure 143 | Multiplicity-edited HSQC experiment of the title compound recorded in CD_3OD.

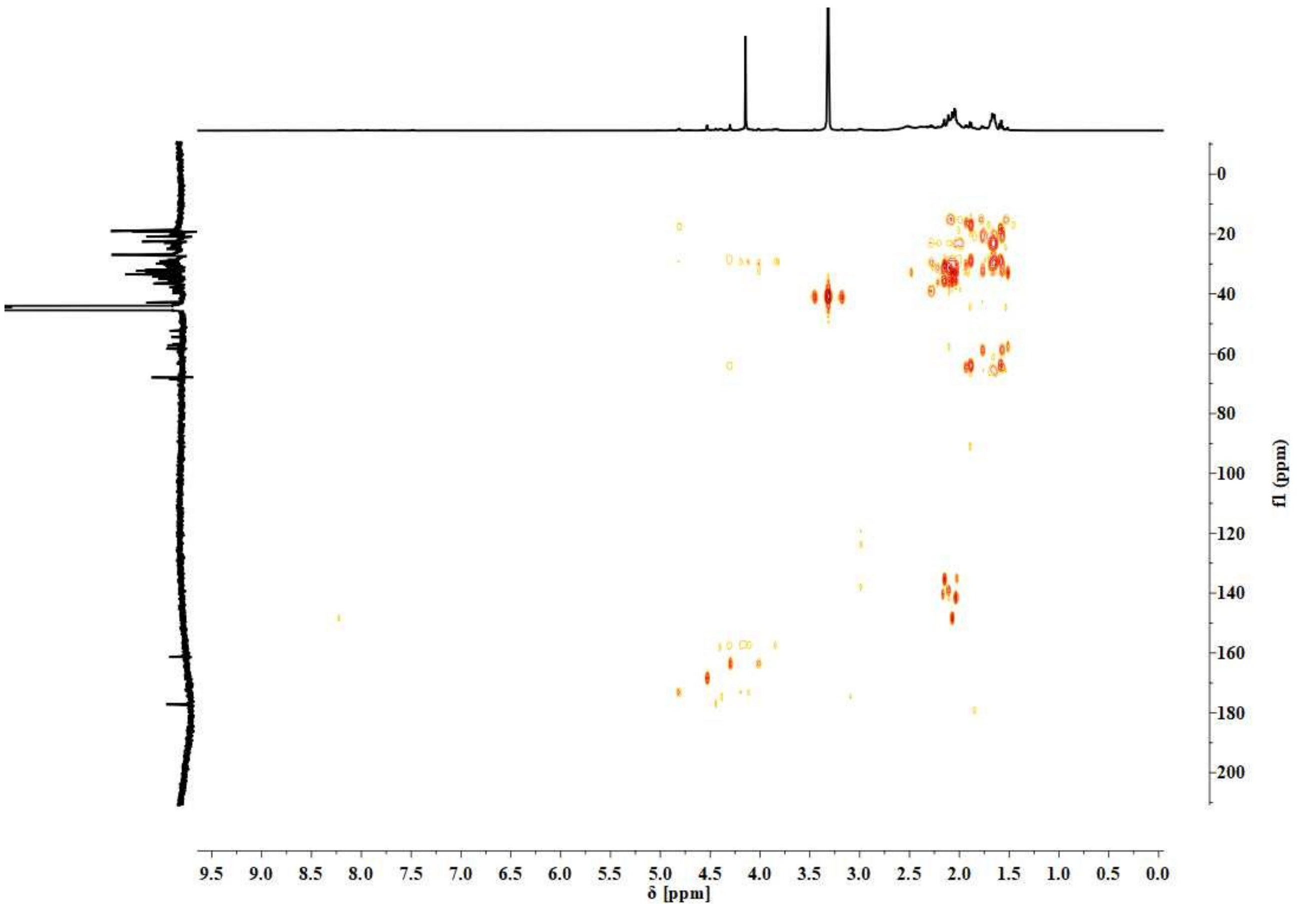

Supplementary Figure 144 | HMBC experiment of the title compound recorded in CD_3OD.

Ugi reaction of perfluorononanoic acid, isobutyraldehyde, cyclohexylisocyanide and cyclohexylamine

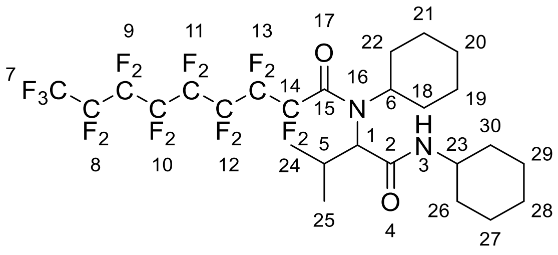

In a 25 mL round bottom flask isobutyraldehyde (46.2 mg, 641 µmol, 1.70 eq.) was dissolved in 1.5 mL methanol, subsequently cyclohexylamine (73.5 µL, 63.6 mg, 641 µmol, 1.70 eq.) was added and the resulting mixture was stirred for 60 min over sodium sulfate. Afterwards, the mixture was filtrated and the solid was washed with 10 mL methanol three times. Subsequently, the filtrate was concentrated under reduced pressure. Perfluorononanoic acid (175 mg, 377 µmol, 1.00 eq.) dissolved in 0.5 mL methanol was added to the imine at room temperature and the resulting mixture was stirred for 2 min. Subsequently, cyclohexylisocyanide (79.9 µL, 70.0 mg, 641 µmol, 1.70 eq.) was added to the stirring mixture. The reaction was stirred for 6 d at room temperature. The crude reaction mixture was dried under reduced pressure and purified *via* column chromatography employing Fluoro*Flash*®

silica gel. The fluorous fraction was tested for purity *via* TLC and concentrated under reduced pressure. The remaining perfluoro acid was removed with a short silica gel filter column, eluting with *c*-hexane/ethyl acetate (3:1) to yield the Ugi product as a highly viscous yellow oil (31.2 mg, 42.9 µmol, 11.4%).

R_f = 0.64 in *c*-hexane/ethyl acetate (6:1). Visualized *via* permanganate staining solution.

IR (ATR): ν [cm^{-1}] = 3343.1 (br, ν(N-H)), 2933.8 (m, ν(C-H)), 2856.3 (m, ν(C-H)), 1675.5 (s, ν(C=O)), 1535.2 (m), 1453.3 (m), 1325.8 (w), 1238.3 (s), 1205.6 (vs), 1148.5 (vs), 1109.9 (m), 1000.4 (w), 896.2 (w), 785.3 (w), 735.4 (m), 702.4 (m), 668.3 (m), 556.8 (w), 529.4 (w), 409.9 (w).

^{1}H NMR (400 MHz, CD_3OD): δ [ppm] = 3.98 – 3.73 (m, 1 H, CH^{1}), 3.72 – 3.59 (m, 1 H, CH^{23}), 3.44 (d, J = 11.1 Hz, 1 H, CH^{6}), 3.02 – 2.80 (m, 1 H, CH^{5}), 1.99 – 1.51 (m, 10 H, CH_2), 1.45 – 1.16 (m, 10 H, CH_2), 1.04 – 0.76 (m, 6 H, $CH_3^{24,25}$).

^{13}C NMR (101 MHz, CD_3OD): δ [ppm] = 172.6 (s, $CONR^{2}$), 160.6 (s, $CONR^{15}$), 71.6 (s, CH^{6}), 61.5 (s, CH^{1}), 49.9 (s, CH^{23}), 33.1 (s, CH_2), 32.7 (s, CH_2), 32.3 (s, CH_2), 27.8 (s, CH^{5}), 26.9 (s, CH_2), 26.6 (s, CH_2), 25.9 (s, CH_2), 25.2 (s, CH_2), 20.3 (s, $CH_3^{24,\,25}$), 20.0 (s, $CH_3^{24,\,25}$).

^{19}F NMR (376 MHz, CD_3OD): δ [ppm] = -88.29 (t, J = 9.9 Hz, 3 F, CF_3^{7}), -114.97 (s, CF_2^{14a}), AB-signal (δ_A = -116.37, δ_B = -118.34, J_{AB} = 293.6 Hz, A and B are split into t, J = 11.3 Hz CF_2^{14b}), -125.13 (s, CF_2), -126.01 (s, CF_2), -126.68 (d, J = 73.4 Hz, CF_2), -128.66 (d, J = 77.4 Hz, CF_2), -129.66 (s, CF_2), -133.16 (s, CF_2^{8}). Total integral of CF_2 region normalized with respect to the CF_3^{7} group = 14.

FAB – MS [*m/z*] (relative intensity): 755.3 (67%) $[M + H]^+$, 600.1 (31%) [Fragment A $]^+$, 518.0 (100%) [Fragment A – $C_6H_{10}]^+$, 98.1 (15%) [Fragment B – CHO$]^+$.

HRMS – FAB [*m/z*]: $[M + H]^+$ calculated for $^{12}C_{26}{}^{1}H_{32}{}^{16}O_2{}^{14}N_2{}^{19}F_{17}$, 727.2187; found, 727.2185; Δ = 0.22 mmu.

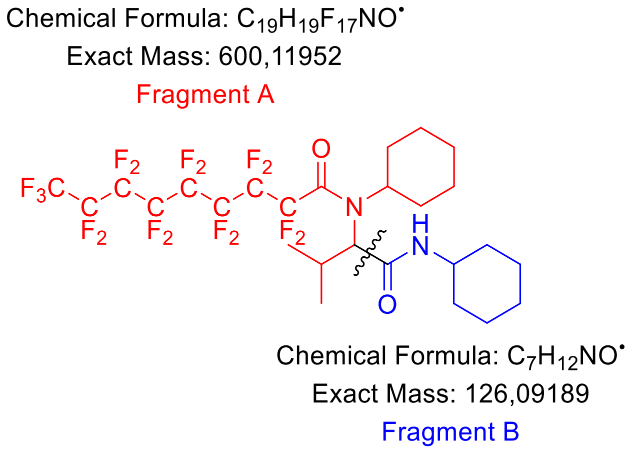

Supplementary Figure 145 | Proposed fragments observed in FAB-MS.

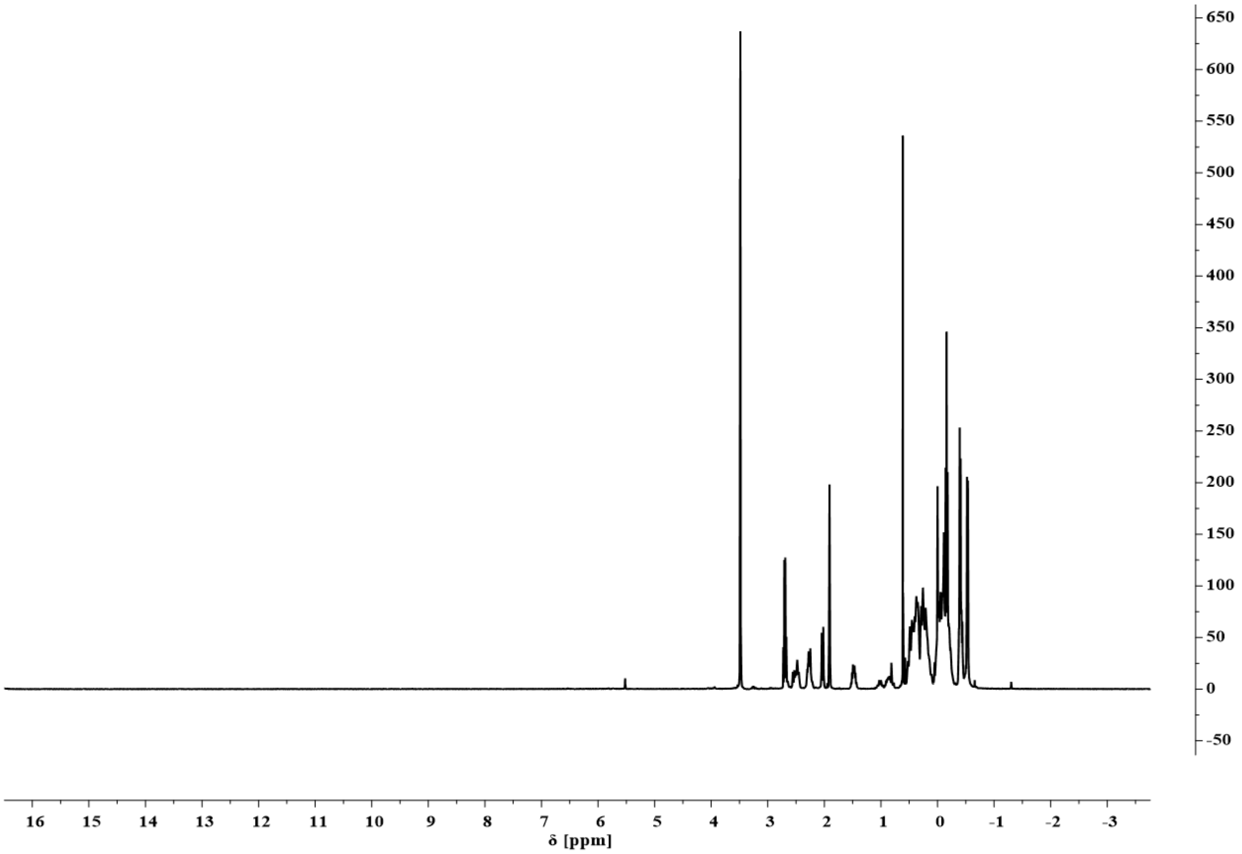

Supplementary Figure 146 | ^{1}H NMR experiment of the title compound recorded in CD_3OD.

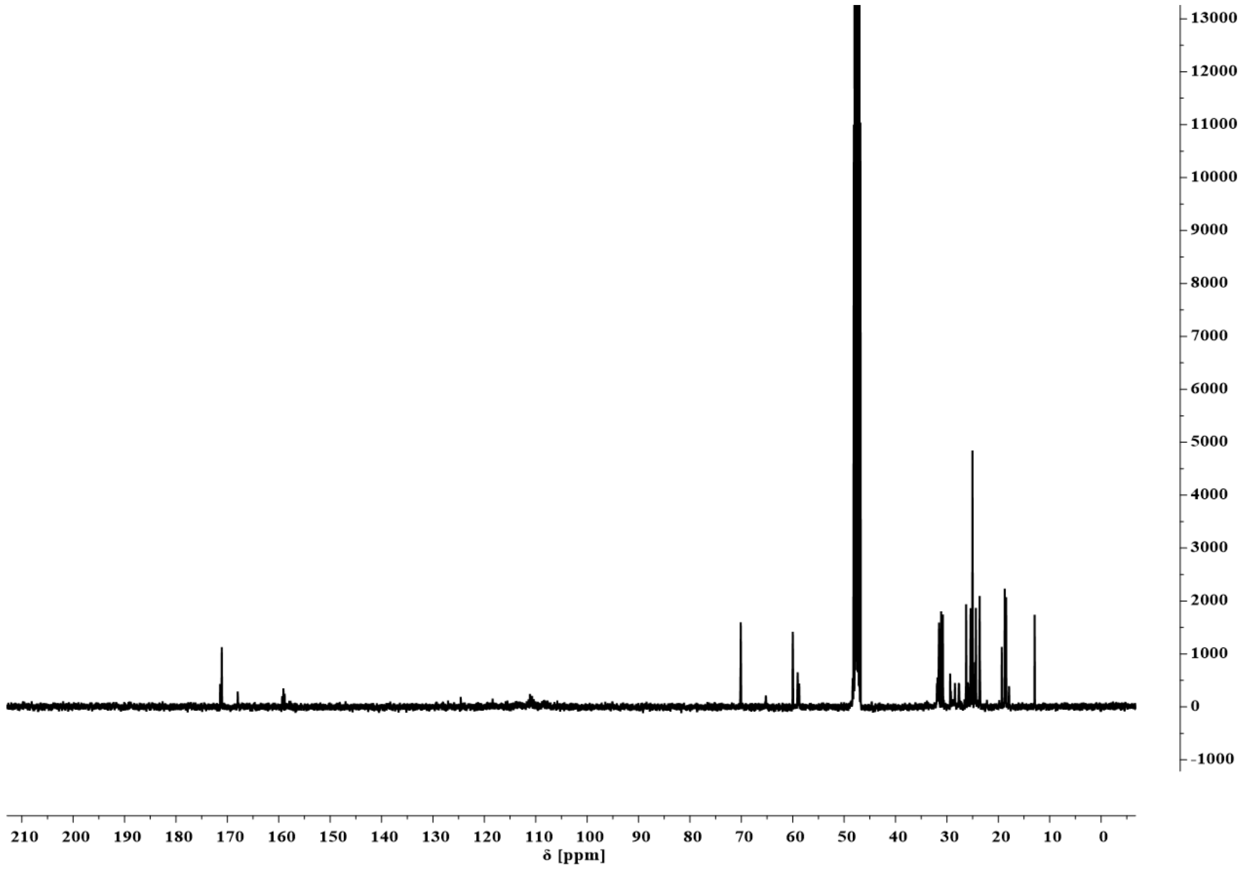

Supplementary Figure 147 | ^{13}C NMR experiment of the title compound recorded in CD_3OD.

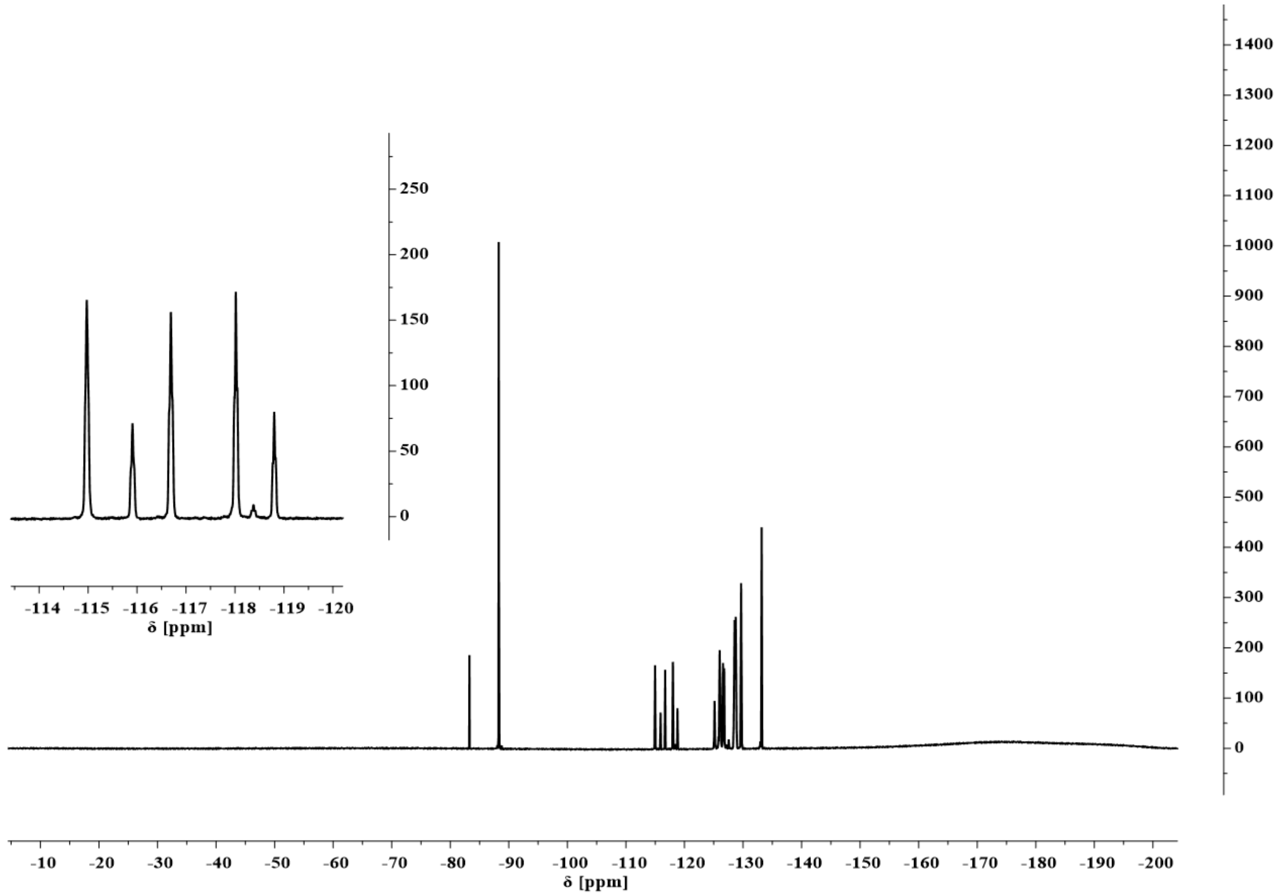

Supplementary Figure 148 | ^{19}F NMR experiment of the title compound recorded in CD_3OD.

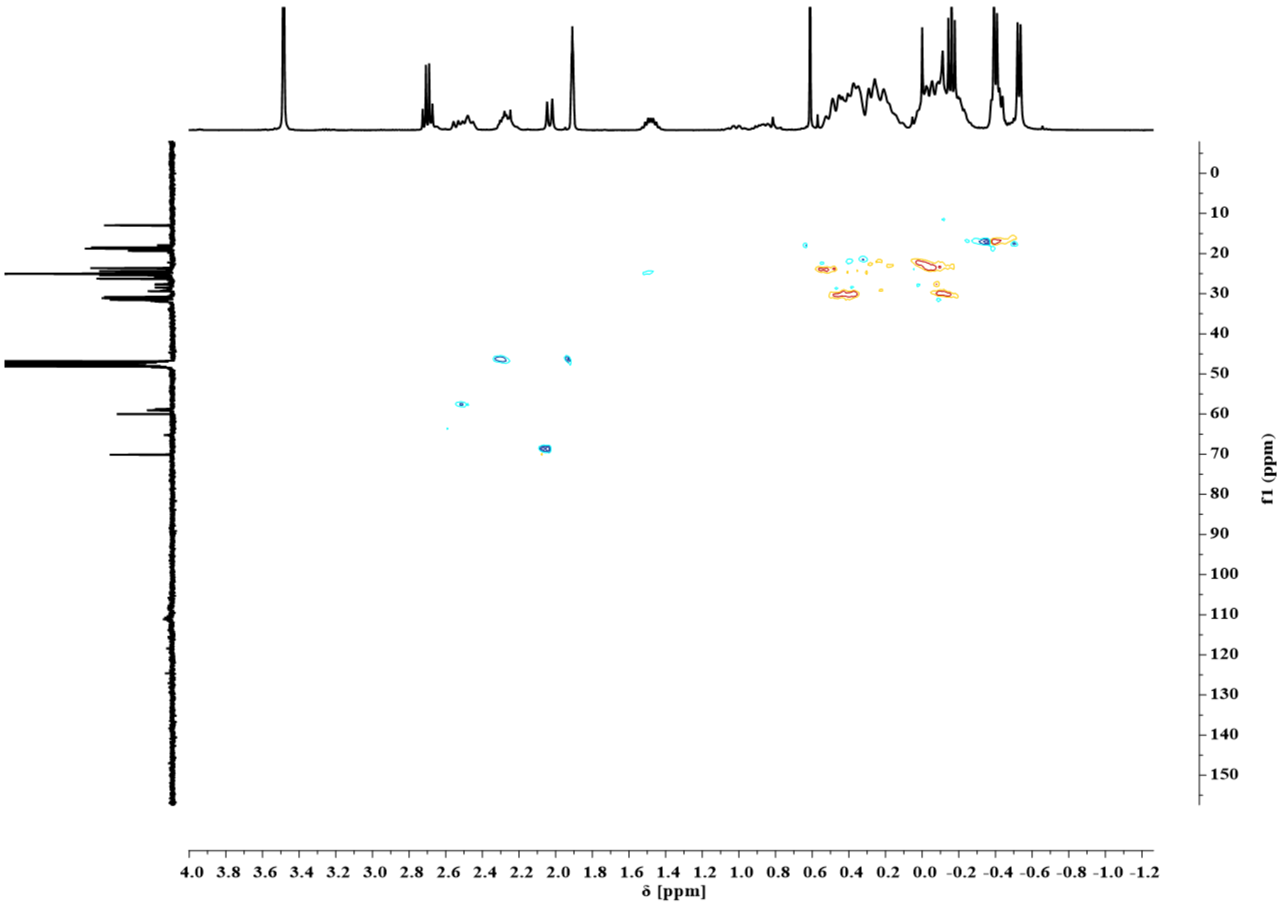

Supplementary Figure 149 | Multiplicity-edited HSQC experiment of the title compound recorded in CD_3OD.

Ugi reaction of perfluoropentanoic acid valeraldehyde, *4*-methoxyphenylisocyanide and butylamine

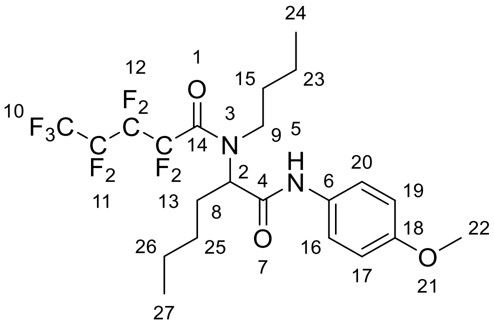

In a 25 mL round bottom flask valeraldehyde (83.2 mg, 966 µmol, 1.70 eq.) was dissolved in 1.5 mL methanol, subsequently butylamine (108 µL, 70.6 mg, 966 µmol, 1.70 eq.) was added and the resulting mixture was stirred for 60 min over sodium sulfate. Afterwards, the mixture was filtrated and the solid was washed with 10 mL methanol three times. Subsequently, the filtrate was concentrated under reduced pressure. Perfluoropentanoic acid (150 mg, 568 µmol, 1.00 eq.) dissolved in 1 mL methanol was added to the imine at room temperature and the resulting mixture was stirred for 2 min. Subsequently, *4*-methoxyphenylisocyanide (108 µL, 129 mg, 966 µmol, 1.70 eq.) was added to the stirring mixture. The reaction was stirred for 3 d at room temperature. The crude reaction mixture was dried under reduced pressure and purified *via* column chromatography employing Fluoro*Flash*® silica gel. The fluorous fraction was tested for purity *via* TLC and concentrated under reduced pressure. The remaining perfluoro acid was removed with a short silica gel filter column, eluting with *c*-hexane/ethyl acetate (3:1) to yield the Ugi product as a yellow powder (18.4 mg, 34.1 µmol, 6.01%).

R_f = 0.48 in *c*-hexane/ethyl acetate (5:1). Visualized *via* fluorescent quench and Seebach staining solution.

IR (ATR): ν [cm^{-1}] = 2957.5 (m, ν(C-H)), 2929.3 (s, ν(C-H)), 2858.8 (m,), 1795.3 (m), 1718.9 (s, ν(C=O)), 1606.3 (vs, ν(C=O)), 1506.0 (m), 1464.9 (m), 1351.9 (m), 1292.8 (vs), 1234.4 (vs), 1136.6 (s), 1099.4 (s), 1036.6 (s), 894.2 (m), 835.9 (s), 793.9 (m), 742.8 (m), 725.7 (m), 691.5 (m), 575.6 (w), 527.3 (w), 435.6 (w).

^{1}H NMR (400 MHz, CD_3OD): δ [ppm] = 7.47 – 7.35 (m, 2 H, $CH_{Ar}^{16,20}$), 6.94 – 6.80 (m, 2 H, $CH_{Ar}^{17,19}$), 4.75 (t, J = 7.6 Hz, 1 H, CH^{2}), 3.76 (s, 3 H, OCH_3^{22}), 3.67 – 3.47 (m, 2 H, CH_2^{9}), 2.15 – 2.00 (m, 1 H, CH_2^{8a}), 1.94 – 1.76 (m, 1 H, CH_2^{8b}), 1.72 – 1.57 (m, 2 H, CH_2), 1.45 – 1.22 (m, 6 H, CH_2), 1.02 – 0.81 (m, 6 H, $CH_3^{24,27}$).

^{13}C NMR (126 MHz, CD$_3$OD): δ [ppm] = 169.9 (s, CONR^{4}), 169.4 (s, CONR^{14}), 158.3 (s, C$_{Ar}$^{18}), 132.2 (s, C$_{Ar}$^{6}), 123.6 (s, CH$_{Ar}$^{16,20}), 115.0 (s, CH$_{Ar}$^{17,19}), 62.5 (s, CH^{2}), 55.8 (s, OCH$_3$^{22}), 47.1 (s, CH$_2$^{9}), 33.7 (s, CH$_2$), 29.7 (s, CH$_2$^{8}), 29.6 (s, CH$_2$), 23.5 (s, CH$_2$), 21.1 (s, CH$_2$), 14.3 (s, CH$_3$$^{24\ or\ 27}$), 13.9 (s, CH$_3$$^{24\ or\ 27}$).

^{19}F NMR (376 MHz, CD$_3$OD): δ [ppm] = -88.25 (t, J = 10.5 Hz, 3 F CF$_3$^{10}), -123.93 (s, CF$_2$^{13}), -125.69 – -127.15 (m, CF$_2$), -133.18 (s, CF$_2$), -133.32 (s, CF$_2$), -134.92 (s, CF$_2$^{11}). Total integral of CF$_2$ region normalized with respect to the CF$_3$^{10} group = 6.

FAB – MS [m/z] (relative intensity): 538.3 (28%) [M + H]$^+$, 523.3 (34%) [M – CH$_3$]$^+$, 220.2 (28%) [Fragment A]$^+$, 122.1 (53%) [Fragment B]$^+$.

HRMS – FAB [m/z]: [M]$^+$ calculated for $^{12}C_{22}{}^{1}H_{27}{}^{16}O_3{}^{14}N_2{}^{19}F_9$, 538.1872; found, 538.1870; Δ = 0.26 mmu.

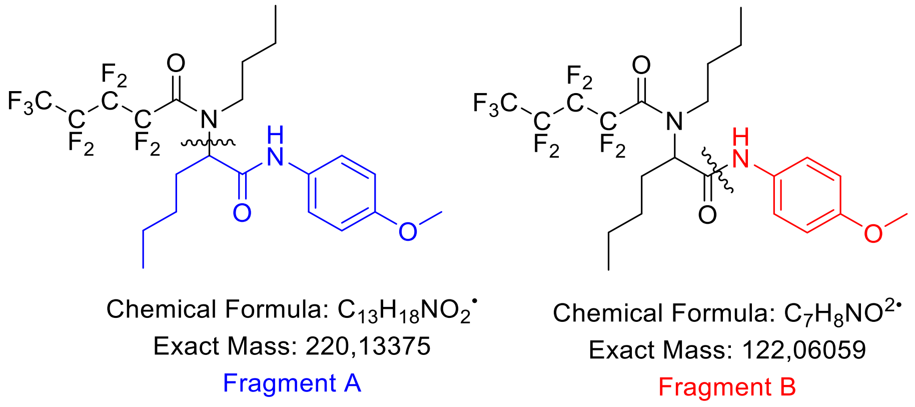

Supplementary Figure 150 | Proposed fragments observed in FAB-MS.

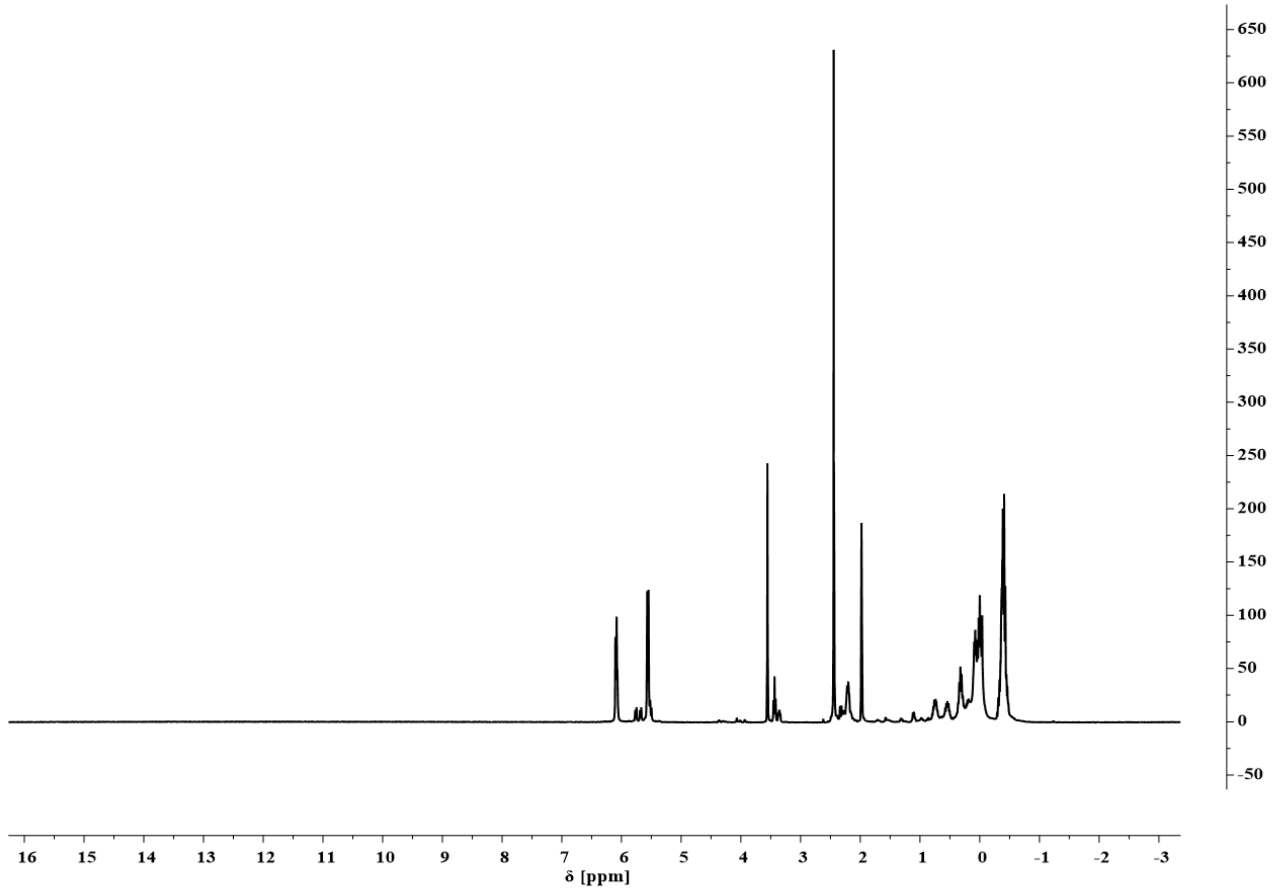

Supplementary Figure 151 | ^{1}H NMR experiment of the title compound recorded in CD_3OD.

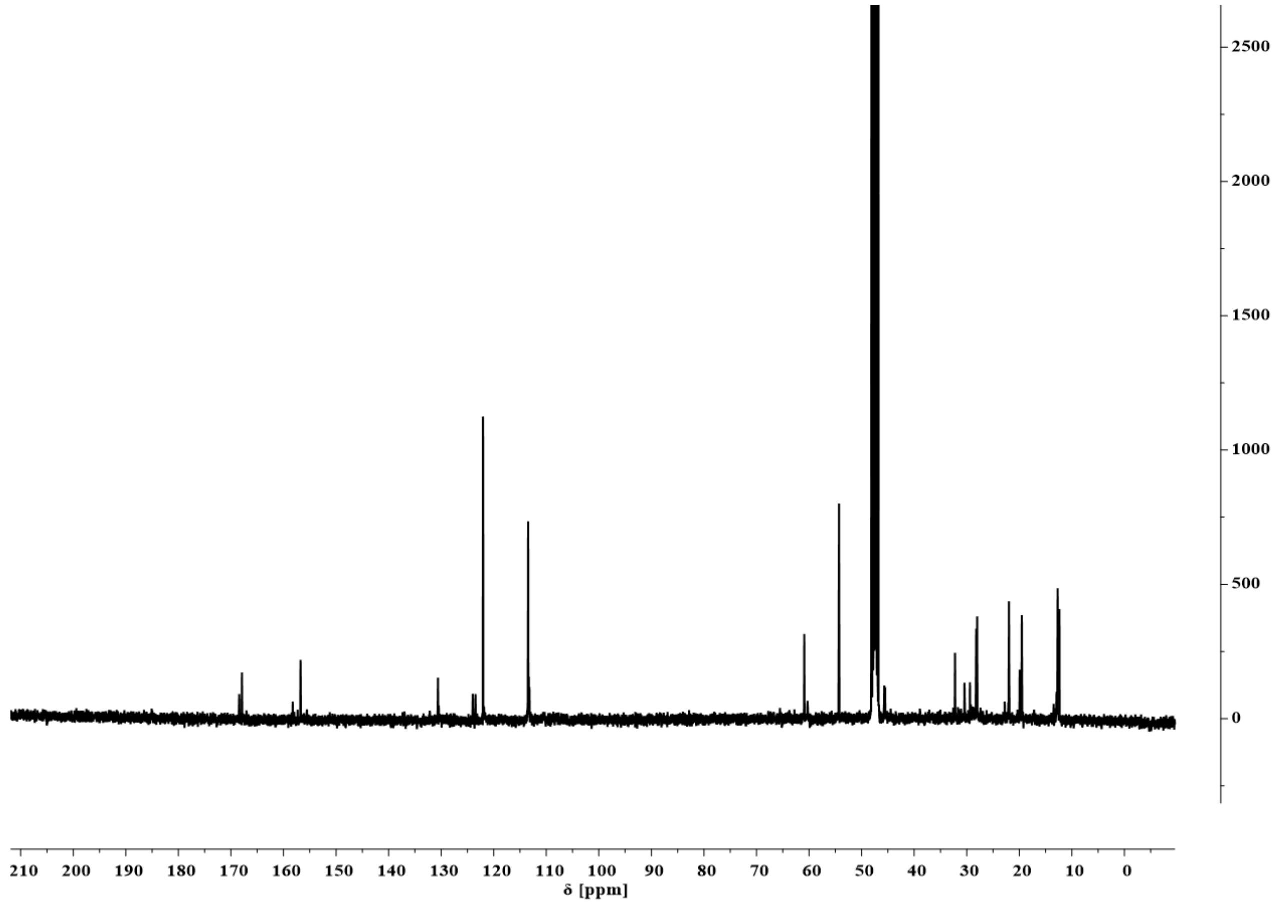

Supplementary Figure 152 | ^{13}C NMR experiment of the title compound recorded in CD_3OD.

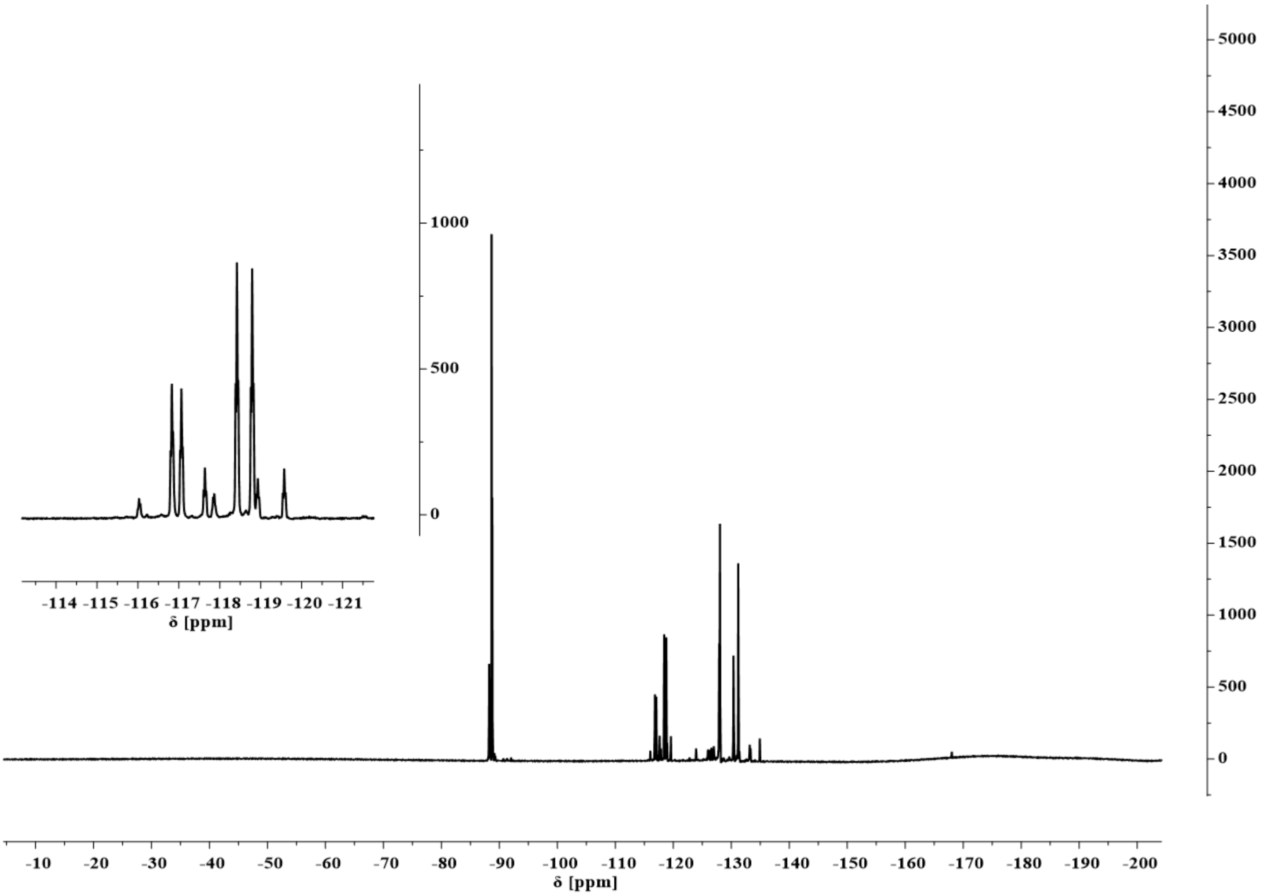

Supplementary Figure 153 | ^{19}F NMR experiment of the title compound recorded in CD_3OD.

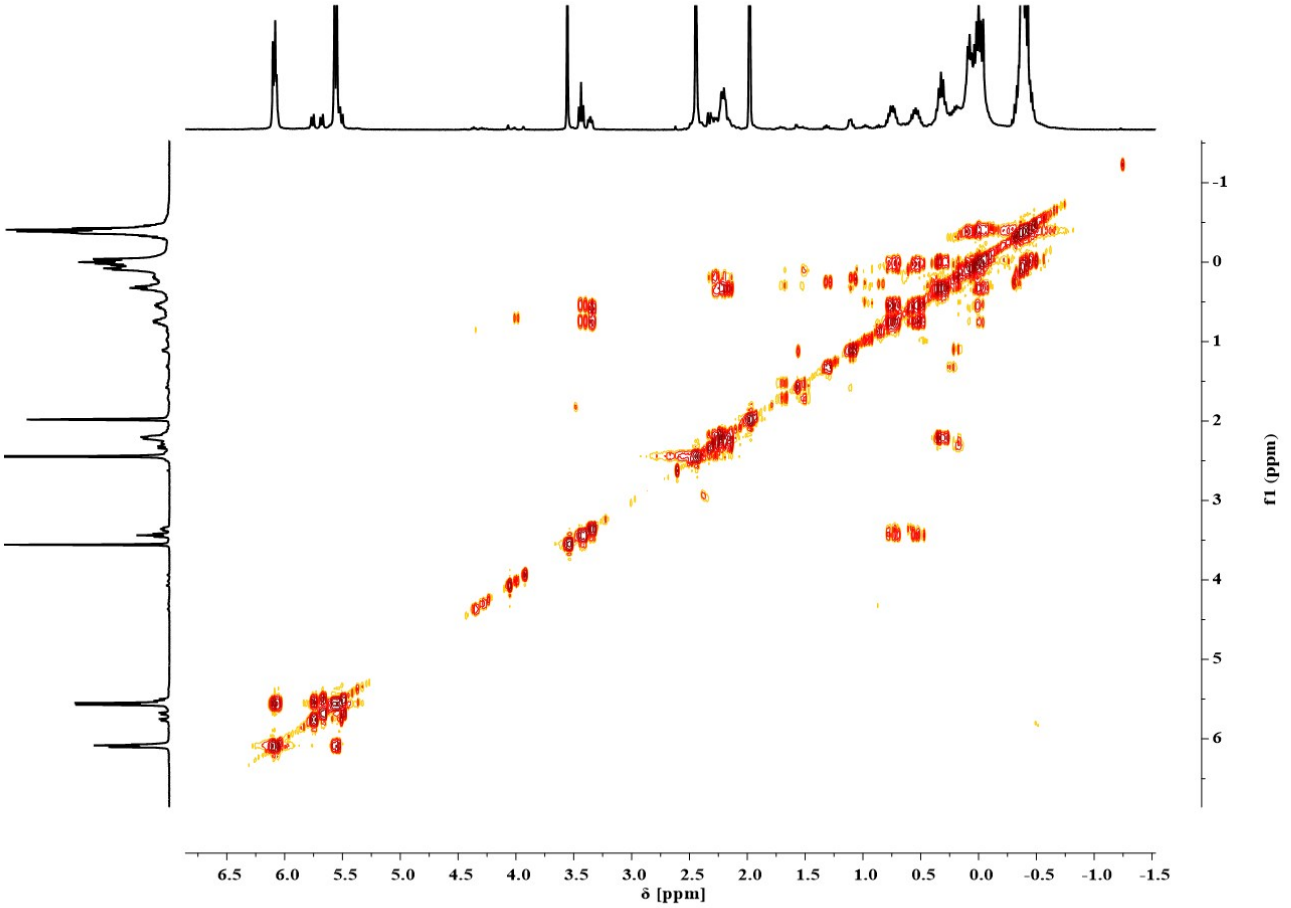

Supplementary Figure 154 | COSY experiment of the title compound recorded in CD_3OD.

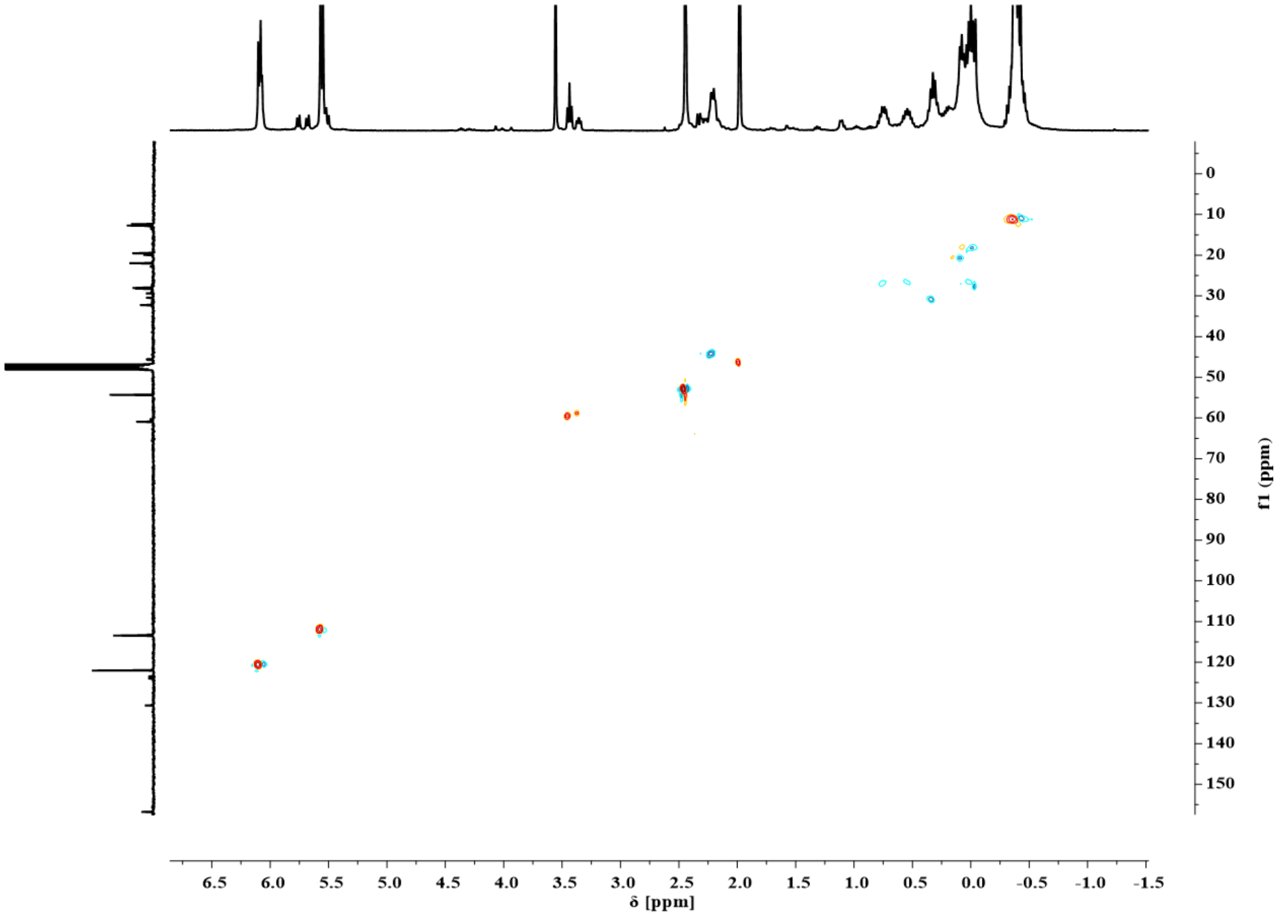

Supplementary Figure 155 | Multiplicity-edited HSQC experiment of the title compound recorded in CD3OD.

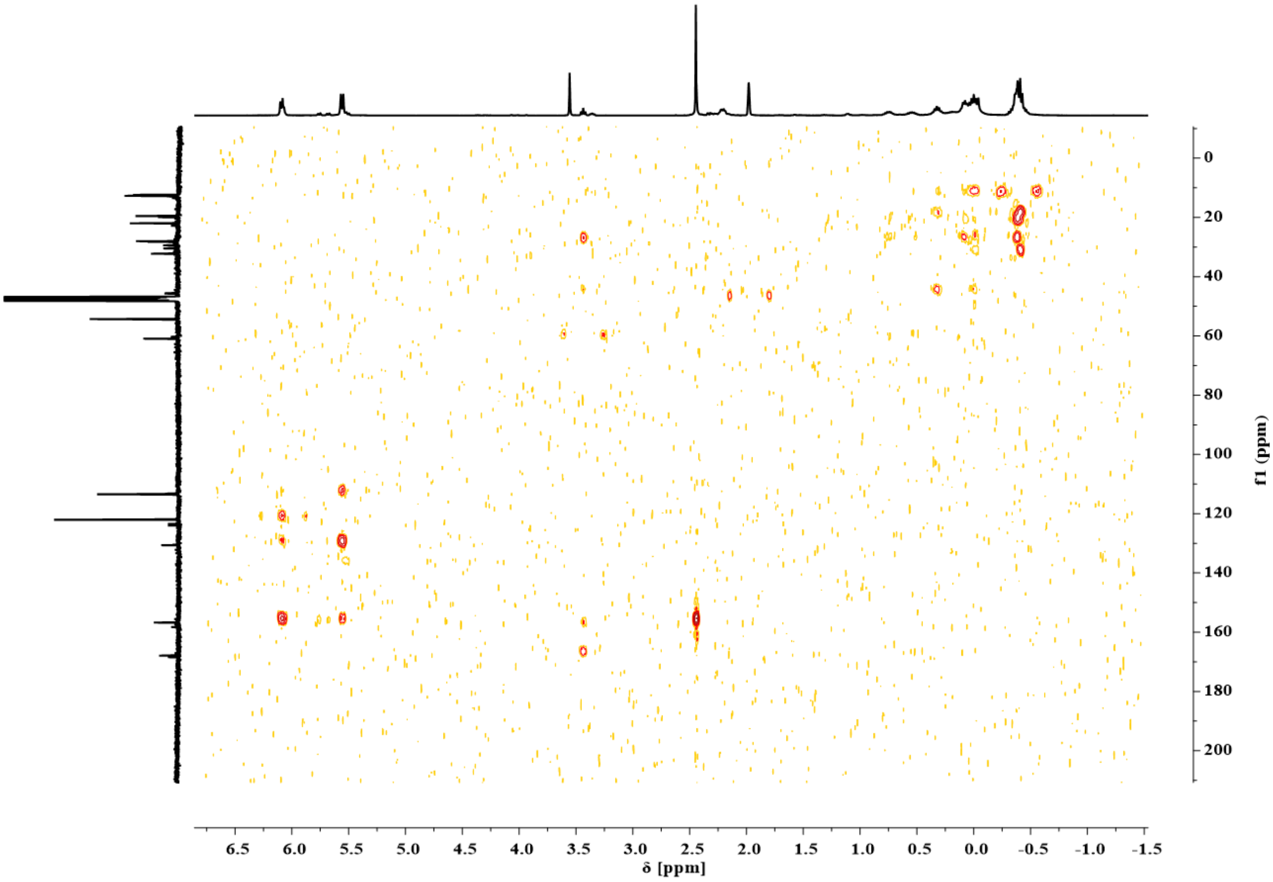

Supplementary Figure 156 | HMBC experiment of the title compound recorded in CD3OD.

Ugi reaction of perfluorotetradecanoic acid, benzaldehyde, *tert*-butylisocyanide and butylamine

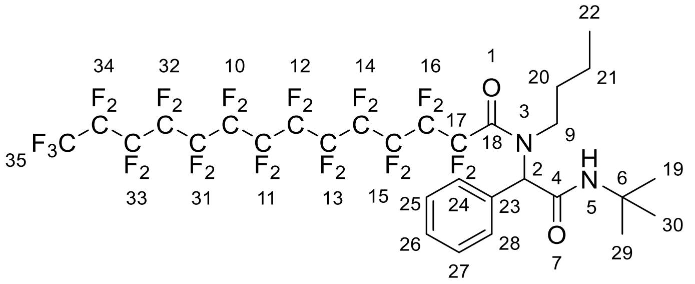

In a 25 mL round bottom flask benzaldehyde (97.2 µL, 101 mg, 952 µmol, 1.70 eq.) was dissolved in 1.5 mL methanol, subsequently butylamine (94.1 µL, 69.6 mg, 952 µmol, 1.70 eq.) was added and the resulting mixture was stirred for 60 min over sodium sulfate. Perfluorotetradecanoic acid (400 mg, 560 µmol, 1.00 eq.) dissolved in 1 mL methanol was added at room temperature and the resulting mixture was stirred for 2 min. Subsequently, *tert*-butylisocyanide (108 µL, 79.2 mg, 952 µmol, 1.70 eq.) was added to the stirring mixture. After 4 h a precipitate was formed and 2 mL tetrahydrofuran were added to homogenize the reaction mixture. The resulting solution was stirred for 5 d at room temperature. The crude reaction mixture was dried under reduced pressure. The residue was adsorbed onto celite® and purified *via* column chromatography employing silica gel and eluting with a gradual solvent mixture of ethyl acetate and *c*-hexane (1:10 → 1:3) to yield the Ugi product as a yellow solid (98.7 mg, 103 mmol, 18.4%).

R_f = 0.50 in *c*-hexane/ethyl acetate (5:1). Visualized *via* fluorescent quench and Seebach staining solution.

IR (ATR): ν [cm^{-1}] = 3321.1 (w, ν(N-H)), 2968.1 (w, ν(C-H)), 1679.5 (s, ν(C=O)), 1654.5 (s, ν(C=O)), 1553.7 (m), 1452.7 (w), 1429.0 (w), 1363.3 (w), 1021.7 (vs), 1149.1 (vs), 1113.3 (s), 1095.2 (m), 1042.1 (m), 987.1 (w), 968.3 (w), 938.3 (w), 873.6 (w), 827.5 (m), 761.6 (m), 729.6 (m), 699.6 (m), 645.8 (s), 549.9 (s), 524.9 (s), 436.8 (w).

^{1}H NMR (400 MHz, CDCl$_3$): δ [ppm] = 7.79 – 7.25 (m, 5 H, $CH_{Ar}^{24\text{-}28}$), 5.83 – 5.10 (m, 2 H, NH^5 + CH^2), 3.79 – 3.16 (m, 2 H, CH_2^9), 1.43 (s, 1 H, CH_2^{20a}), 1.40 – 1.22 (m, 9 H, $CH_3^{19,29,30}$), 1.16 – 0.82 (m, 3 H, CH_2^{20b} + CH_2^{21}), 0.77 – 0.56 (m, 3 H, CH_3^{22}).

^{13}C NMR (101 MHz, CDCl$_3$): δ [ppm] = 166.2 (s, $CONR^4$), 159.8 (s, $CONR^{18}$), 132.6 (s, C_{Ar}^{23}), 129.4 (s, CH_{Ar}), 128.6 (s, CH_{Ar}), 127.6 (s, CH_{Ar}), 64.8 (s, CH^{2a}), 62.1 (s, CH^{2b}), 50.8 (s, C^6), 46.3 (s, CH_2^9), 30.8 (s, $CH_2^{20\ or\ 21}$), 27.4 (s, $CH_3^{18,\ 29,\ 30}$), 18.7 (s, $CH_2^{20\ or\ 21}$), 12.3 (s, CH_3^{22}).

^{19}F NMR (376 MHz, CDCl$_3$): δ [ppm] = -80.78 (t, J = 9.7 Hz, 3 F, CF_3^{35}), AB-signal (δ_A = -108.81, δ_B = -109.78, J_{AB} = 237.2 Hz, A and B are split into t, J = 13.1 Hz, CF_2^{17a}), AB-signal (δ_A = -111.27,

δ_B = -112.34, J_{AB} = 233.4 Hz, CF_2[17b], additional coupling not resolved, signals broadened), -120.31 (s, CF_2), -121.76 (s, CF_2), -122.77(s, CF_2), -126.18 (s, CF_2[34]). Total integral of CF_2 region normalized with respect to the CF_3[35] group = 24.

FAB – MS [*m/z*] (relative intensity): 959.1 (25%) $[M + H]^+$, 886.0 (27%) $[\text{Fragment A}]^+$, 858.0 (43%) $[\text{Fragment B}]^+$, 802.0 $[\text{Fragment B} - C_4H_9]^+$.

HRMS – FAB [*m/z*]: $[M + H]^+$ calculated for $^{12}C_{30}{}^{1}H_{26}{}^{16}O_2{}^{14}N_2{}^{19}F_{27}$, 959.1558; found, 959.1557; Δ = 0.09 mmu.

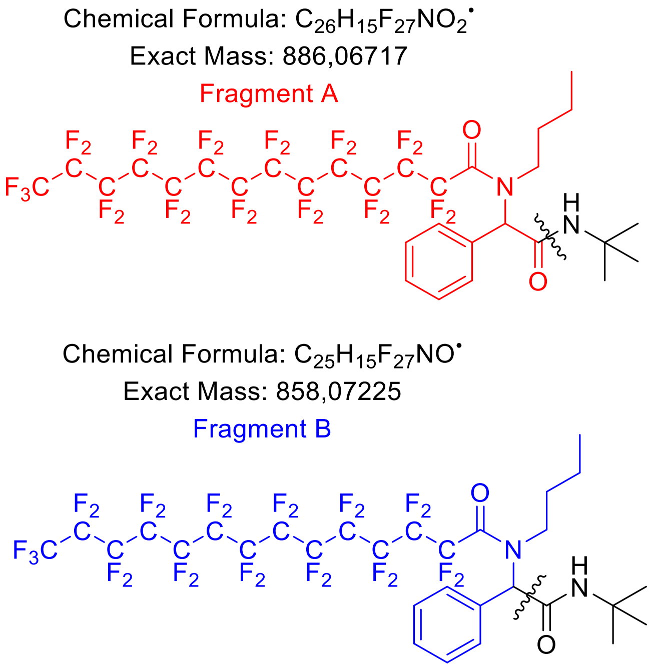

Supplementary Figure 157 | Proposed fragments observed in FAB-MS.

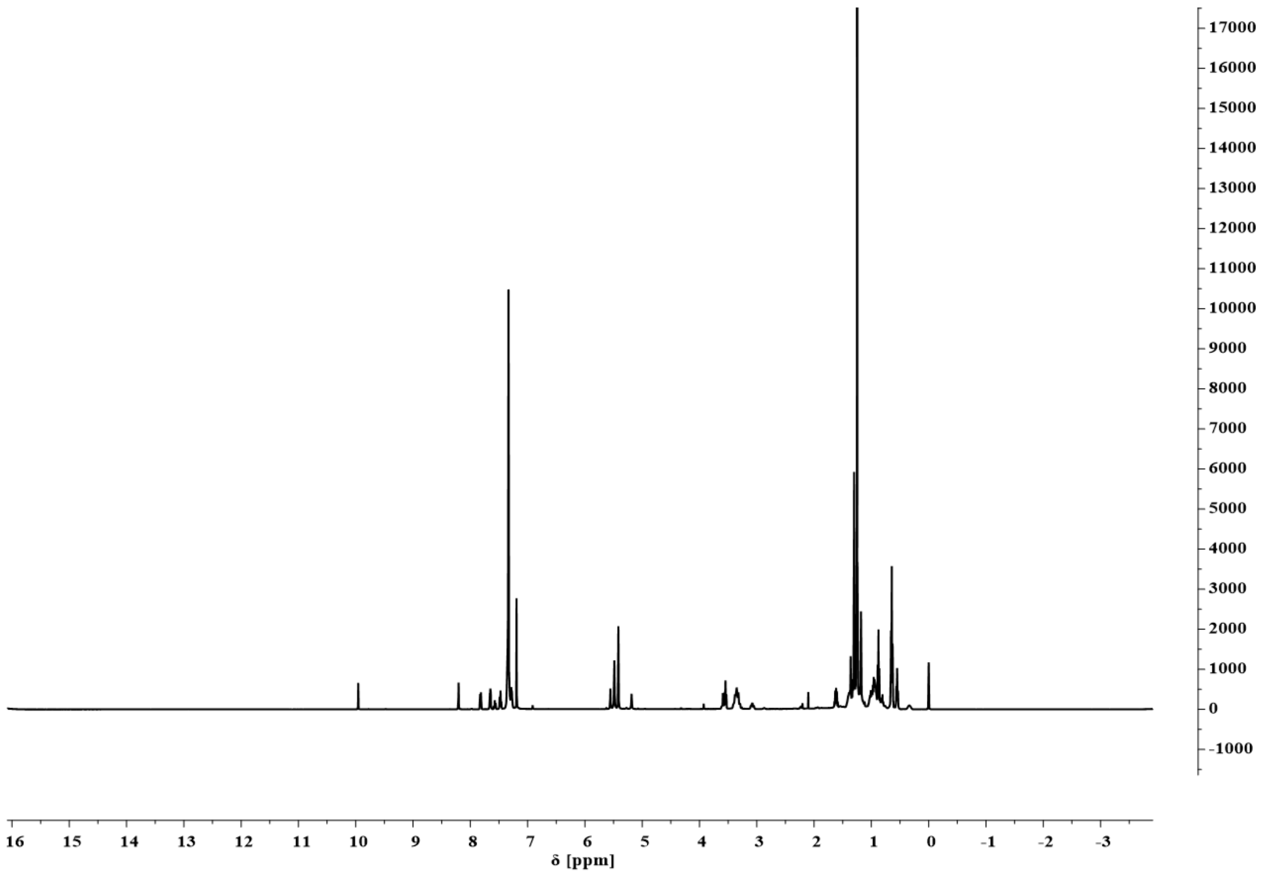

Supplementary Figure 158 | ^{1}H NMR experiment of the title compound recorded in CD_3OD.

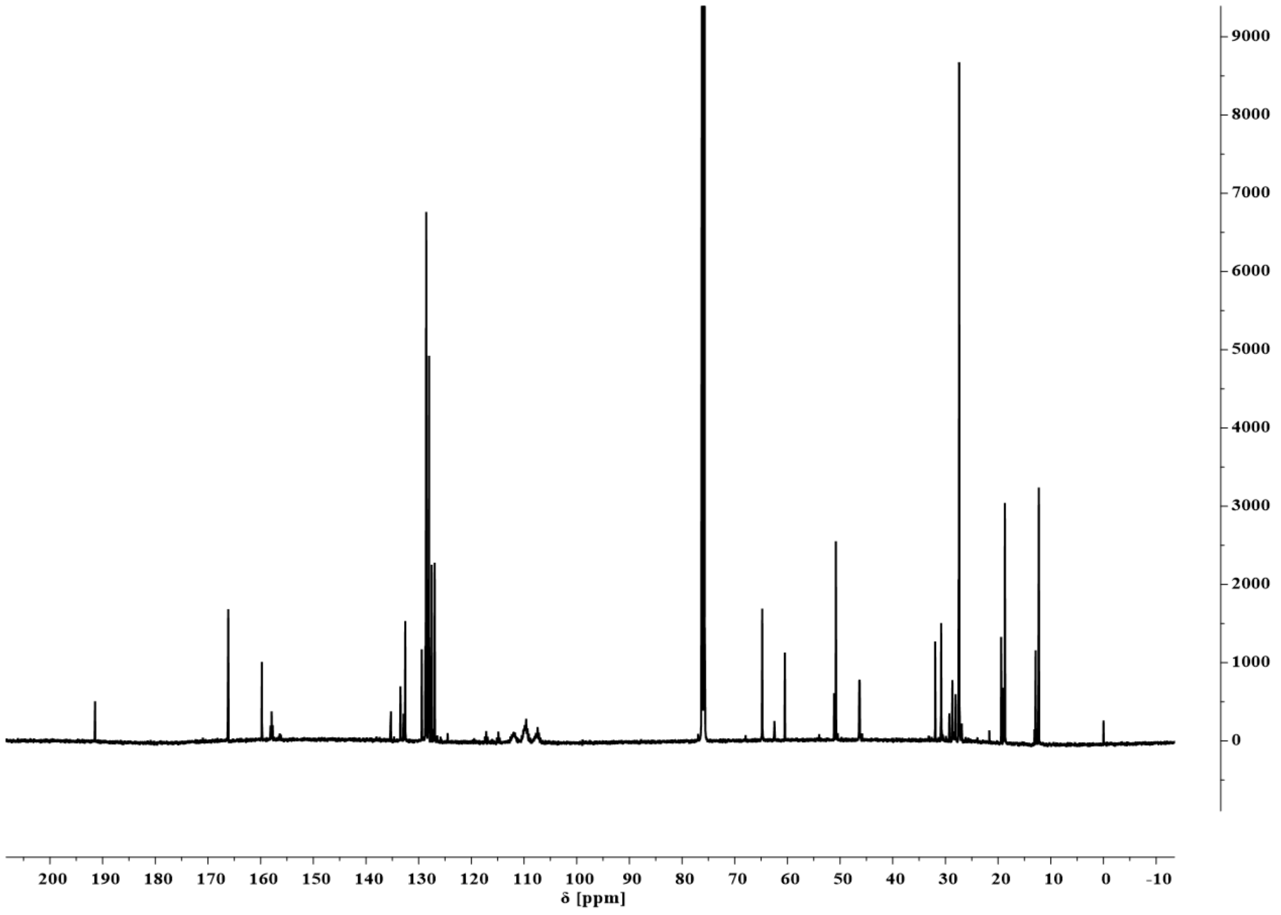

Supplementary Figure 159 | ^{13}C NMR experiment of the title compound recorded in CD_3OD.

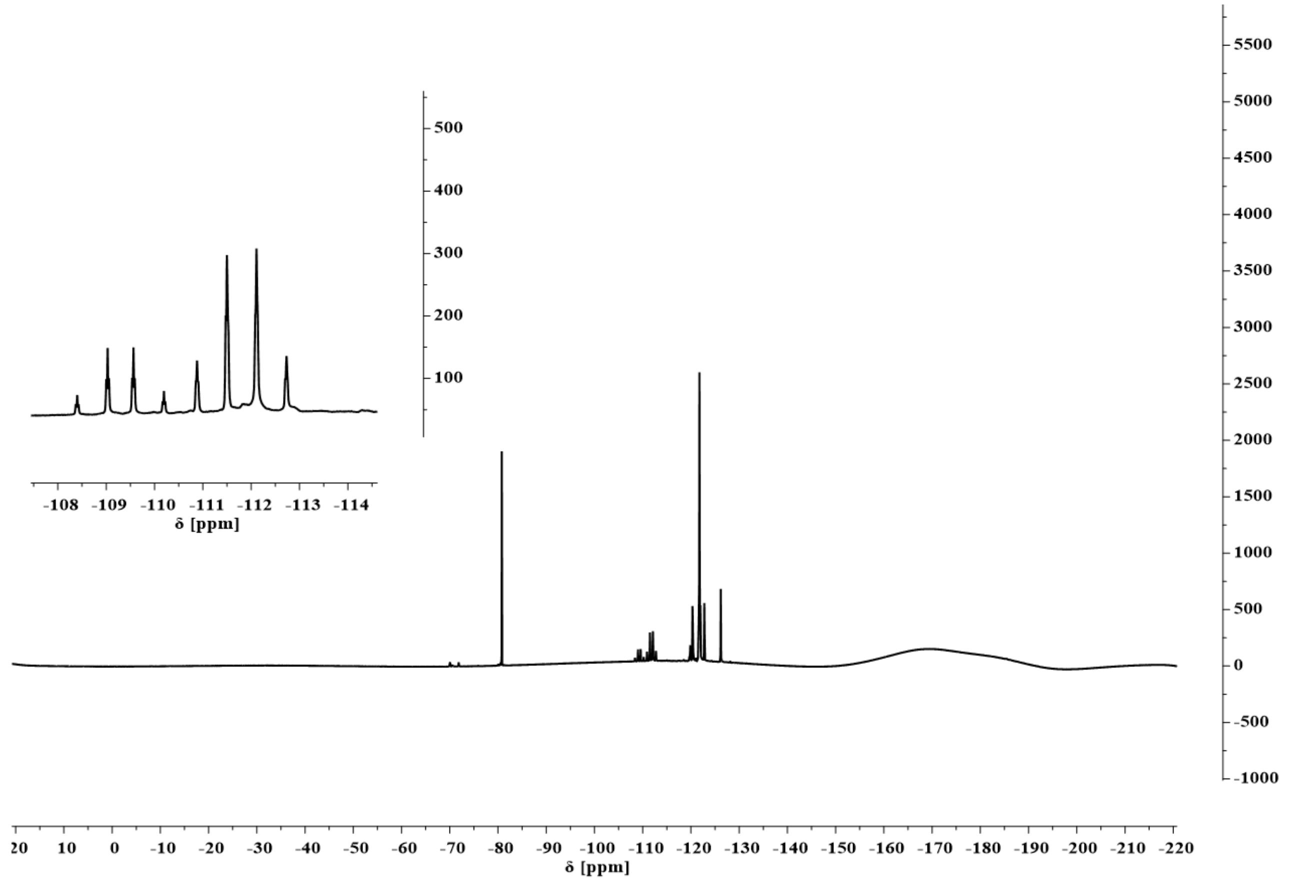

Supplementary Figure 160 | ^{19}F NMR experiment of the title compound recorded in CD_3OD.

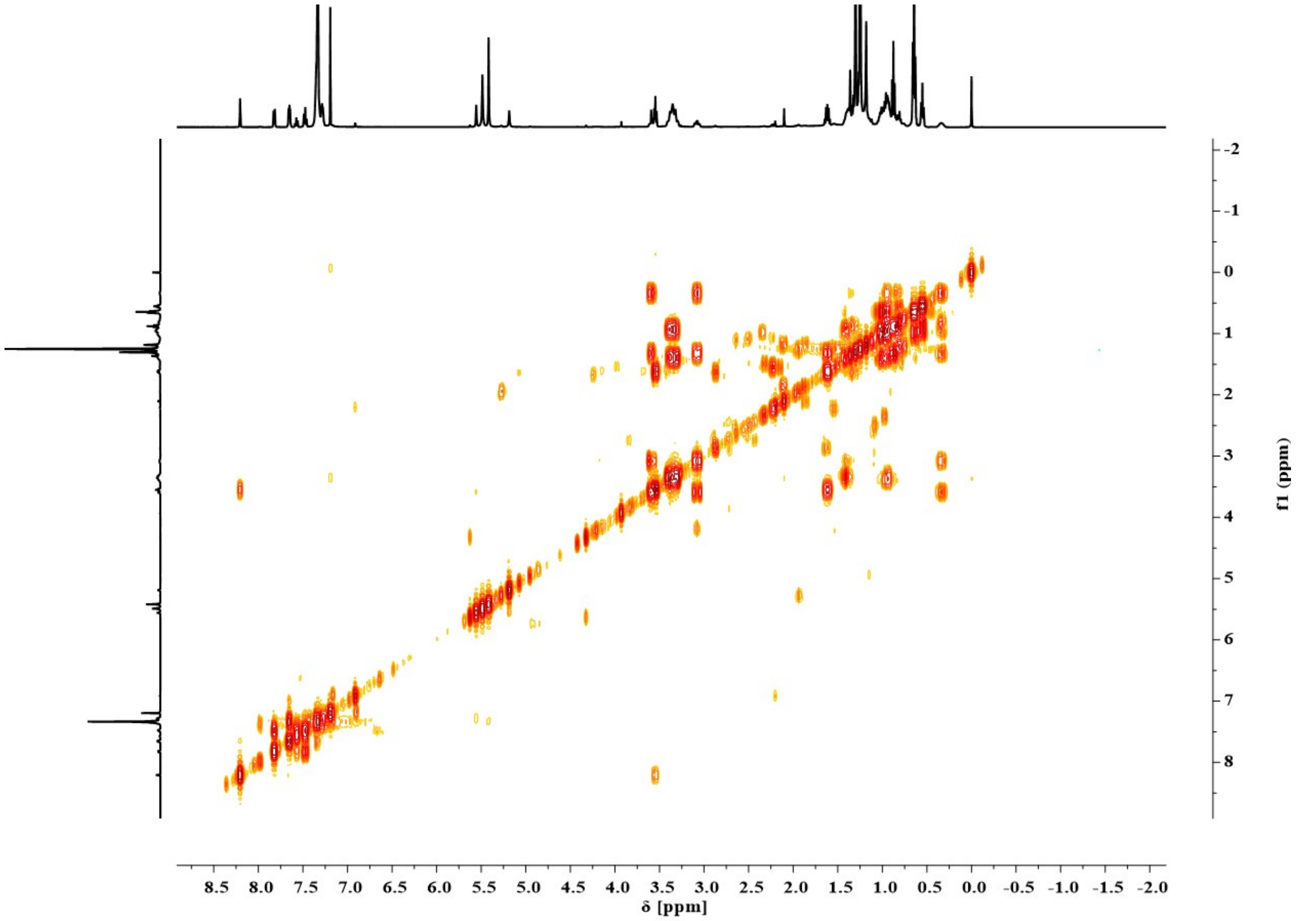

Supplementary Figure 161 | COSY experiment of the title compound recorded in CD_3OD.

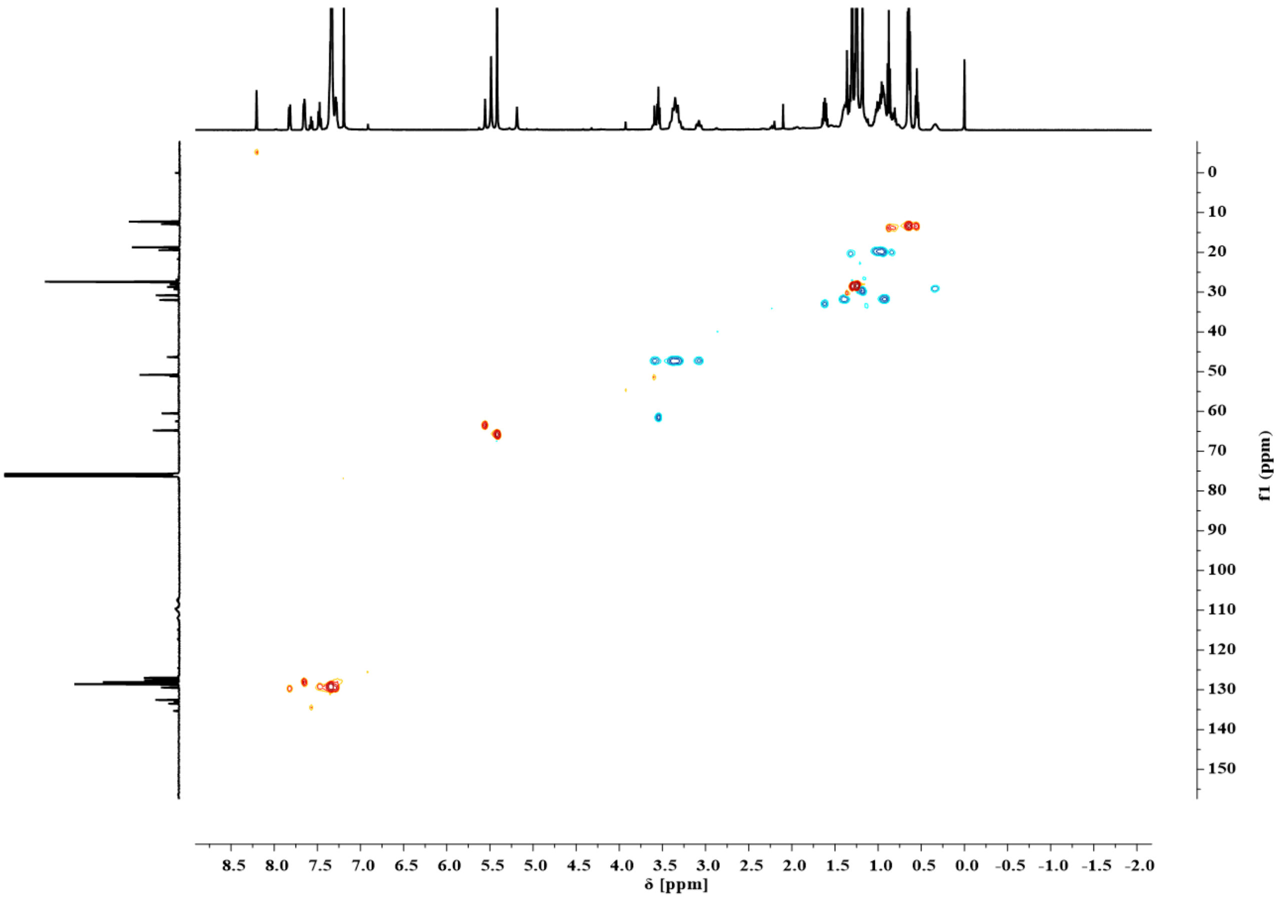

Supplementary Figure 162 | Multiplicity-edited HSQC experiment of the title compound recorded in CD_3OD.

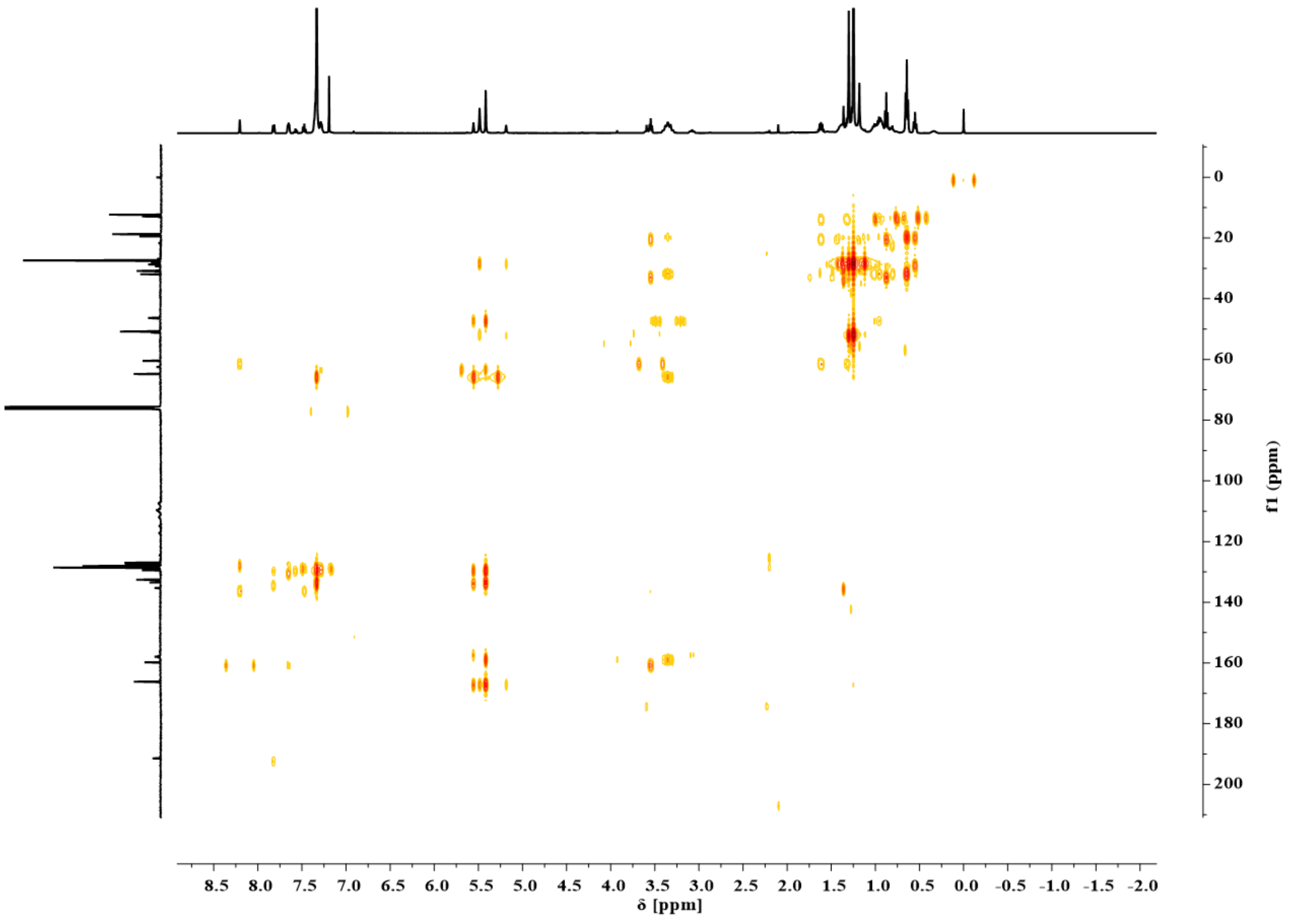

Supplementary Figure 163 | HMBC experiment of the title compound recorded in CD_3OD.

Ugi reaction of perfluoropentanoic acid benzaldehyde, *4*-methoxyphenylisocyanide and pentylamine

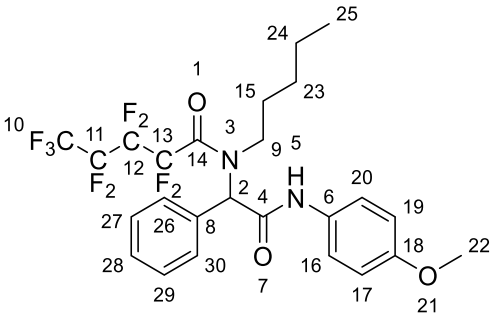

In a 25 mL round bottom flask benzaldehyde (115 µL, 119 mg, 1.12 mmol, 1.70 eq.) was dissolved in 1.5 mL methanol, subsequently butylamine (114 µL, 82.4 mg, 1.12 mmol, 1.70 eq.) was added and the resulting mixture was stirred for 60 min over sodium sulfate. Afterwards, the mixture was filtrated and the solid was washed with 10 mL methanol three times. Subsequently, the filtrate was concentrated under reduced pressure. Perfluoropentanoic acid (175 mg, 663 µmol, 1.00 eq.) dissolved in 2 mL methanol was added to the imine at room temperature and the resulting mixture was stirred for 2 min. Subsequently, *tert*-butylisocyanide (127 µL, 93.7 mg, 1.12 mmol, 1.70 eq.) was added to the stirring mixture. The reaction was stirred for 4 d at room temperature. The crude reaction mixture was dried under reduced pressure and purified *via* column chromatography employing fluoro flash silica gel. The fluorous fraction was tested for purity via TLC and concentrated under reduced pressure. The remaining perfluoro acid was removed with a short silica gel filter column, eluting with *c*-hexane/ethyl acetate (3:1) to yield the Ugi product as a colorless powder (258 mg, 451 µmol, 68.1%).

R_f = 0.29 in *c*-hexane/ethyl acetate (4:1). Visualized *via* fluorescent quench and Seebach staining solution.

IR (ATR): ν [cm^{-1}] = 3307.9 (br, ν(N-H)), 2962.1 (m, ν(C-H)), 2932.8 (m, ν(C-H)), 1673.9 (vs, ν(C=O)), 1657.4 (s, ν(C=O)), 1599.4 (m), 1544.4 (s), 1513.9 (w), 1494.6 (w), 1477.9 (m), 1463.6 (m), 1452.6 (m), 1431.4 (m), 1417.4 (m), 1381.8 (w), 1353.1 (m), 1298.5 (m), 1284.8 (m), 1262.9 (m), 1234.3 (s), 1211.9 (vs), 1197.1 (vs), 1185.5 (s), 1175.2 (s),1136.8 (vs), 1126.6 (s), 1110.5 (vs), 1034.0 (s), 974.4 (w), 950.7 (s), 931.0 (w), 870.8 (w), 849.6 (w), 829.7 (s), 812.4 (s), 802.4 (s), 760.1 (m), 745.8 (m), 722.2 (m), 704.7 (vs), 632.3 (m), 612.4 (m), 574.9 (w), 548.1 (m), 524.7 (m), 512.3(s), 474.4 (m), 436.9 (w).

^{1}H NMR (400 MHz, CDCl$_3$): δ [ppm] = 7.44 (d, J = 2.6 Hz, 5 H, CH$_{Ar}$$^{26\text{-}30}$), 7.31 (d, J = 9.0 Hz, 2 H, CH$_{Ar}$^{16,20}), 6.79 (d, J = 8.9 Hz, 2 H, CH$_{Ar}$^{17,19}), 5.74 (s, 1 H, CH^2), 3.88 – 3.66 (m, 3 H, OCH$_3$^{22}), 3.58 – 3.25 (m, 2 H, CH$_2$^{9}), 1.57 (d, J = 58.6 Hz, 2 H, CH$_2$), 1.19 – 0.91 (m, 4 H, CH$_2$), 0.75 (t, J = 7.0 Hz, 3 H, CH$_3$^{25}).

^{13}C NMR (126 MHz, $CDCl_3$): δ [ppm] = 166.6 (s, $CONR^{4}$), 159.7 (s, C_{Ar}^{18}), 157.1 (s, $CONR^{14}$), 133.4 (s, C_{Ar}^{8}), 130.8 (s, C_{Ar}^{6}), 130.1 (s, CH_{Ar}), 129.9 (s, CH_{Ar}), 129.7 (s, CH_{Ar}), 122.3 (s, $CH_{Ar}^{16,20}$), 114.5 (s, $CH_{Ar}^{17,19}$), 66.6 (s, CH^{2}), 55.9 (s, OCH_3^{22}), 48.2 (s, CH_2^{9}), 29.9 (s, CH_2), 29.1 (s, CH_2), 22.3 (s, CH_2), 14.2 (s, CH_3^{25}).

^{19}F NMR (376 MHz, $CDCl_3$): δ [ppm] = -85.38 (t, J = 9.9 Hz, 3 F, CF_3^{10}), AB-signal (δ_A = -113.13, δ_B = -114.21, J_{AB} = 301.20 Hz, A and B are split into t, J = 12.3 Hz, CF_2^{13a}), AB-signal (δ_A = -115.78, δ_B = -116.72, J_{AB} = 291.8 Hz, A and B are split into t, J = 12.4 Hz, CF_2^{13a}), -125.48 (s, CF_2), -128.89 (s, CF_2^{11}). Total integral of CF_2 region normalized with respect to the CF_3^{10} group = 6.

ESI-MS [m/z]: $[M + Na]^+$ calculated for $^{12}C_{25}{}^{1}H_{25}{}^{16}O_3{}^{14}N_2{}^{19}F_9{}^{23}Na$, 595.1614; found, 595.1615, Δ = 0.13 mmu.

ESI-MS [m/z]: $[2M + Na]^+$ calculated for $^{12}C_{50}{}^{1}H_{50}{}^{16}O_6{}^{14}N_4{}^{19}F_{18}{}^{23}Na$, 1167.3335; found, 1167.3348, Δ = 1.32 mmu.

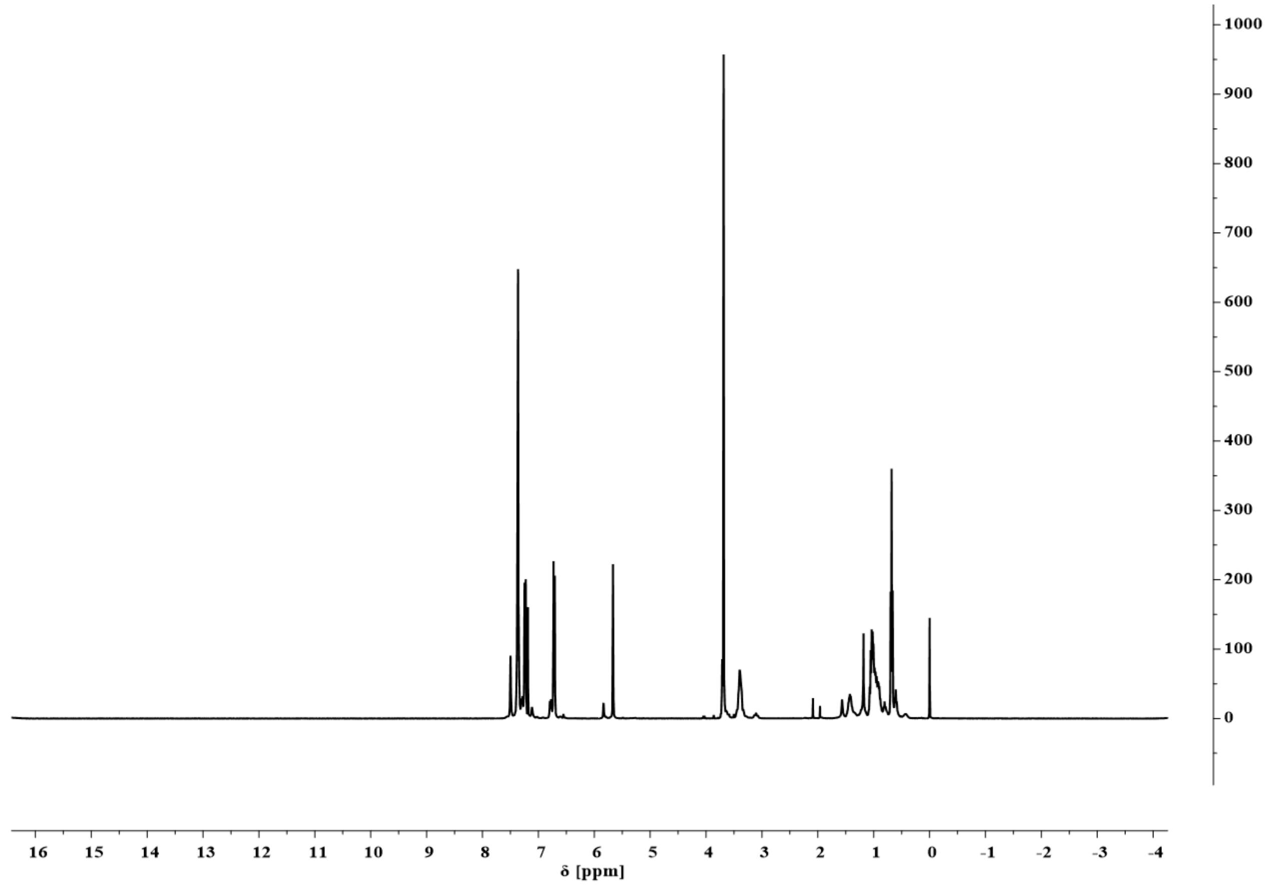

Supplementary Figure 164 | ^{1}H NMR experiment of the title compound recorded in CD_3OD.

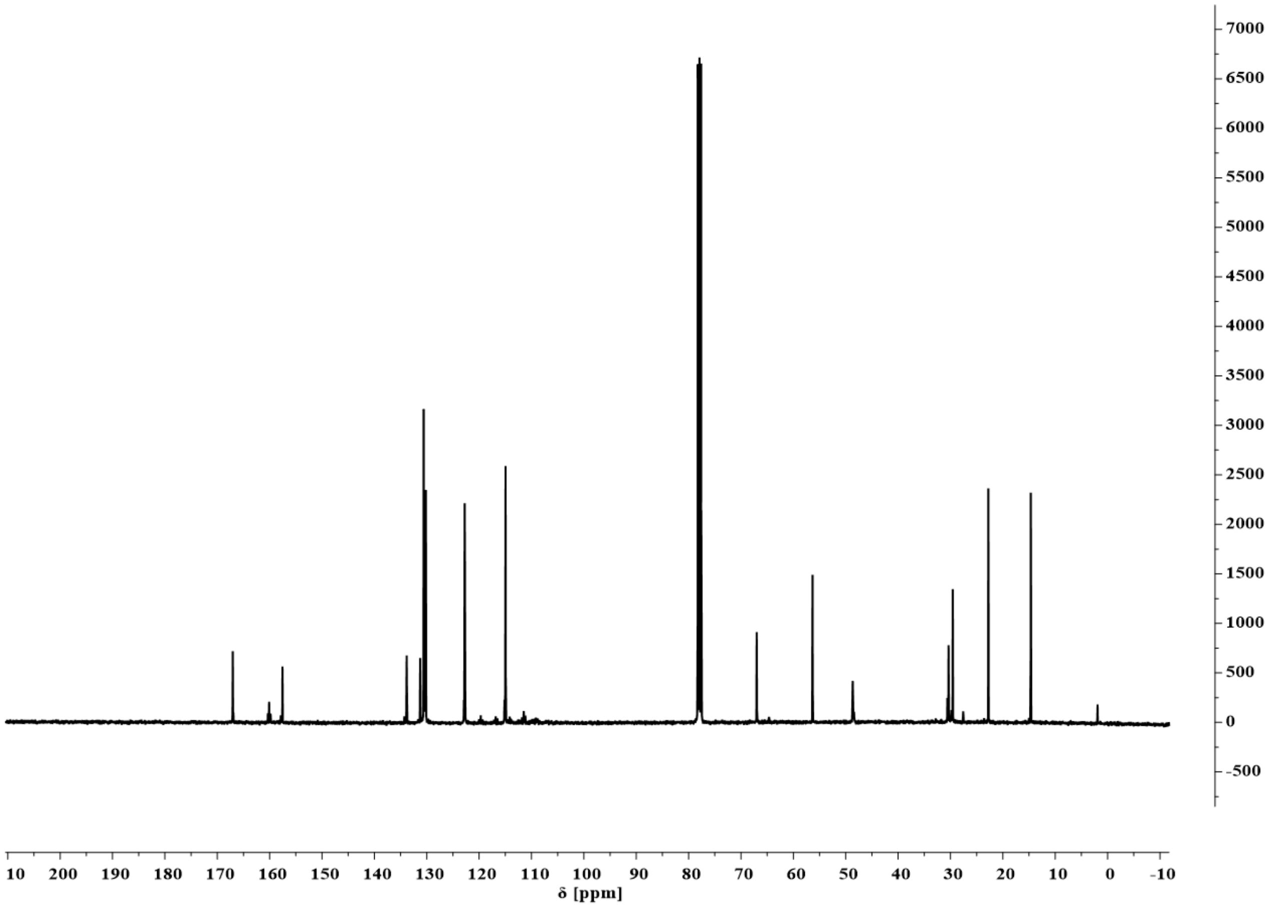

Supplementary Figure 165 | ^{13}C NMR experiment of the title compound recorded in CD_3OD.

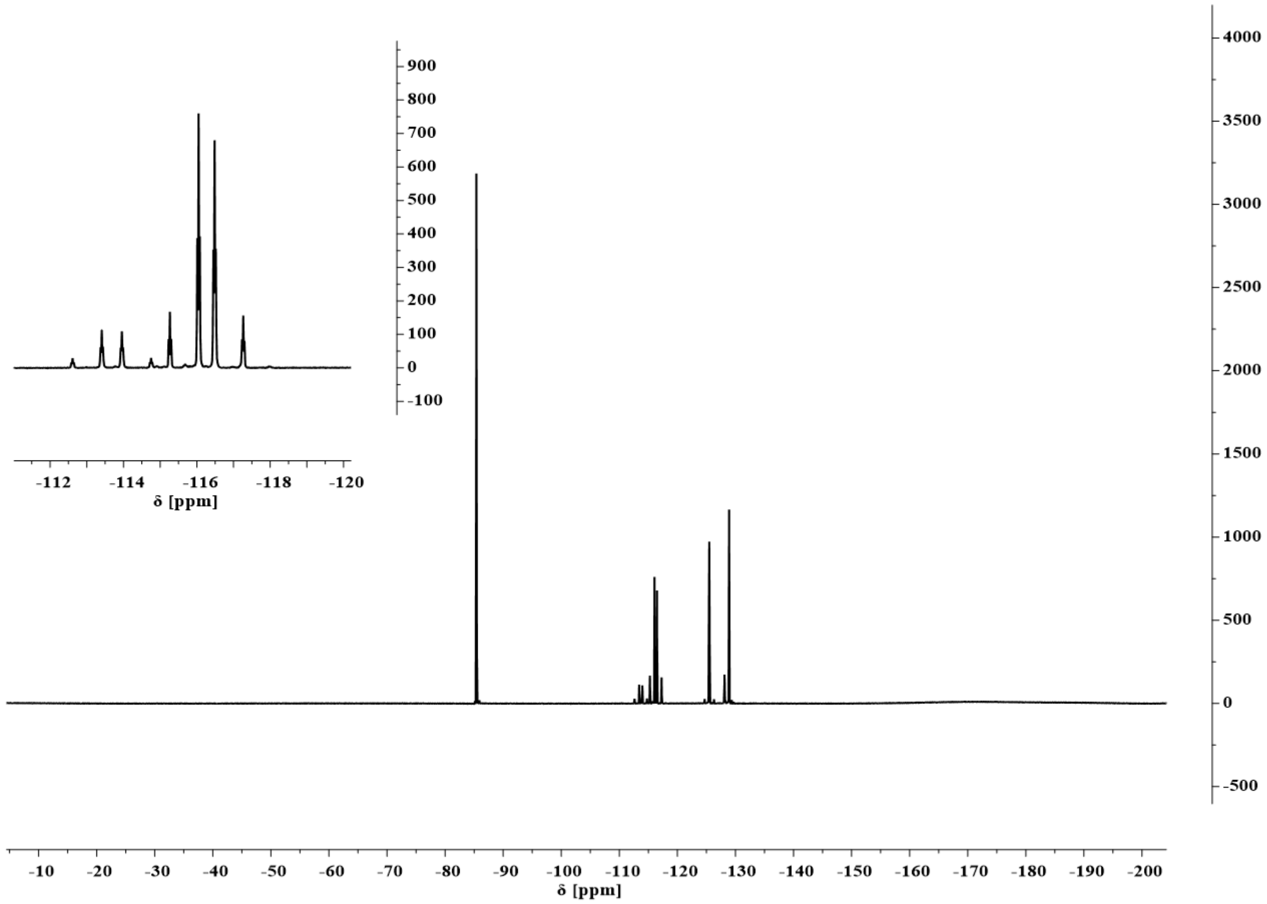

Supplementary Figure 166 | ^{19}F NMR experiment of the title compound recorded in CD_3OD.

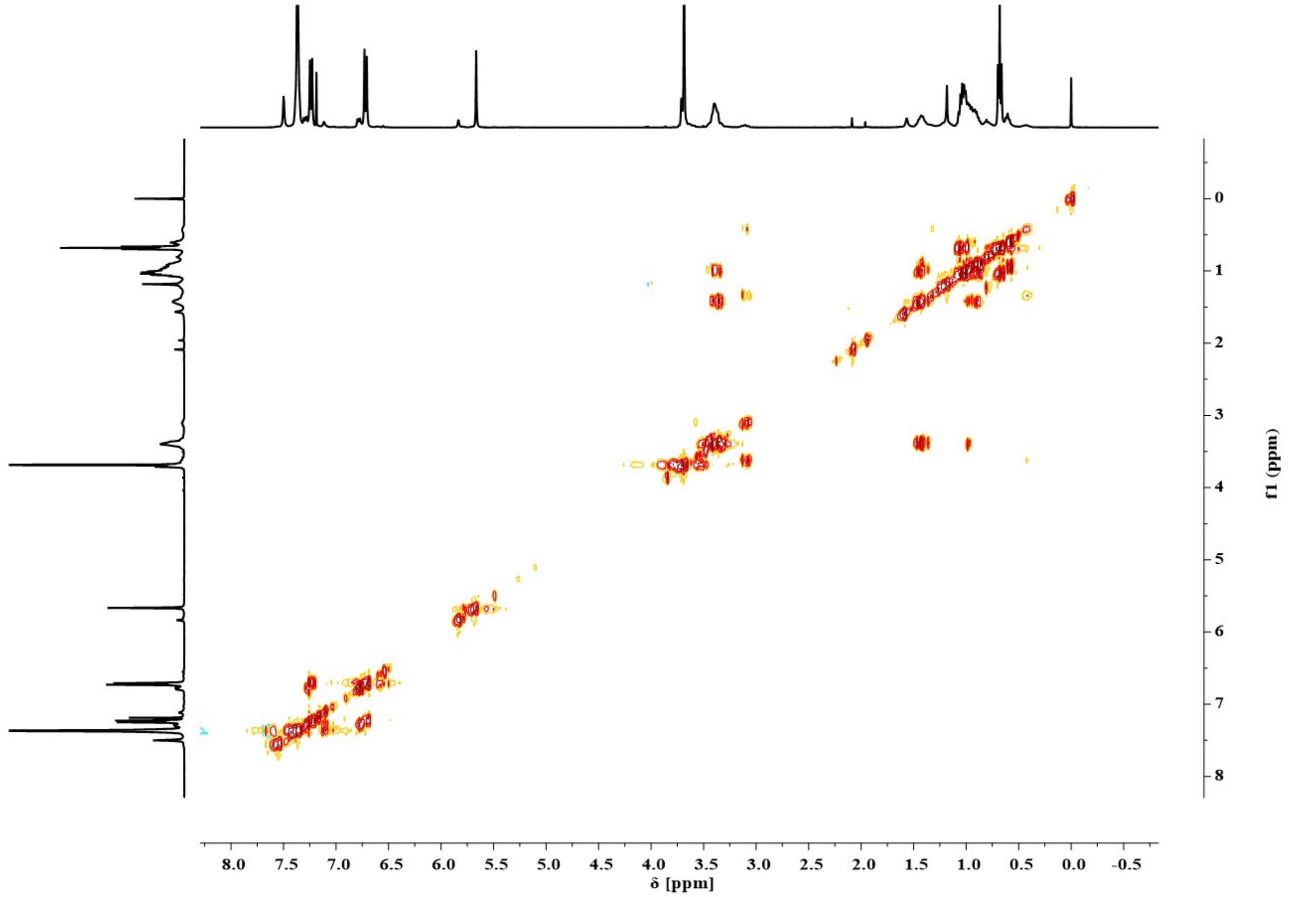

Supplementary Figure 167 | COSY experiment of the title compound recorded in CD_3OD.

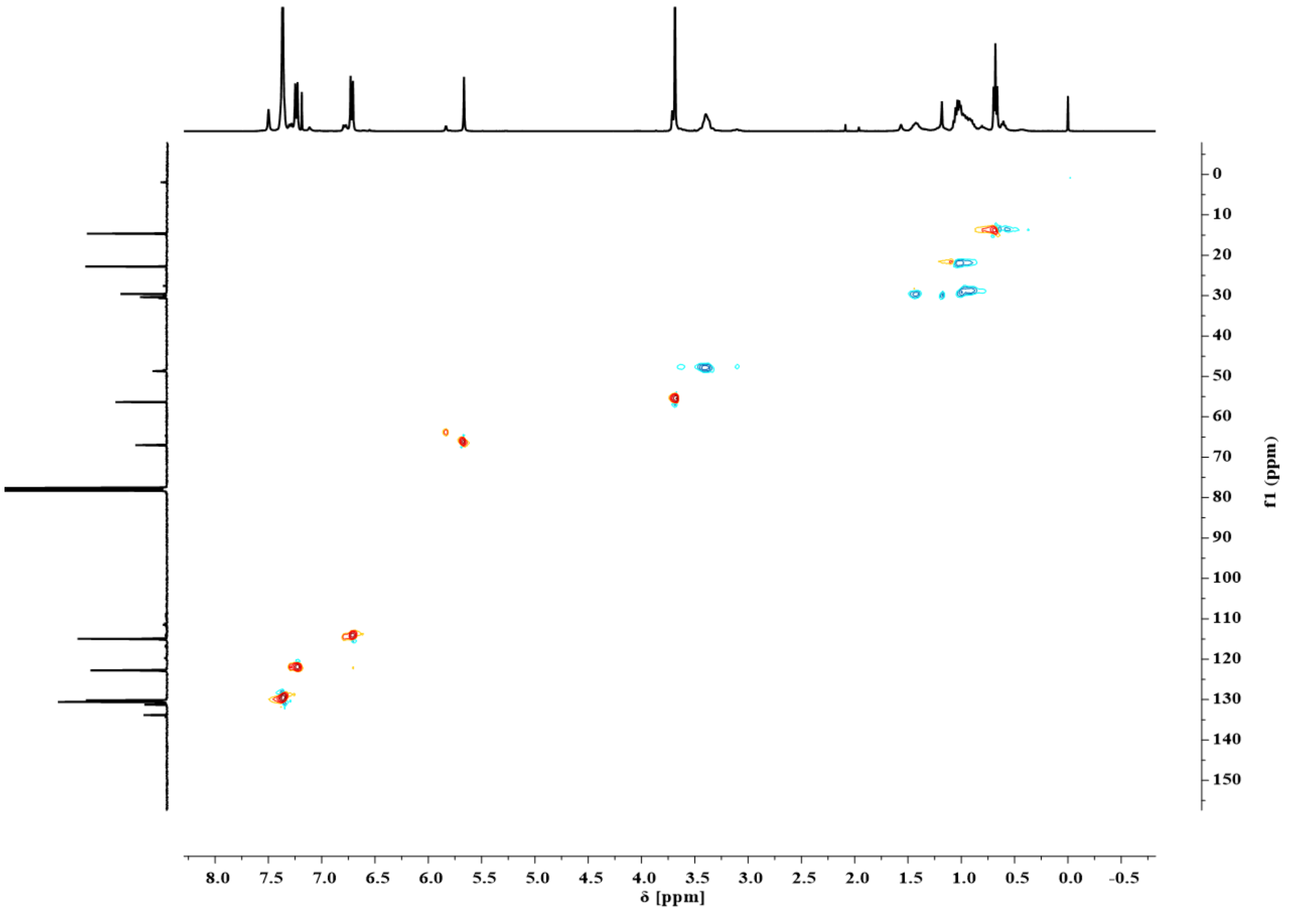

Supplementary Figure 168 | Multiplicity-edited HSQC experiment of the title compound recorded in CD_3OD.

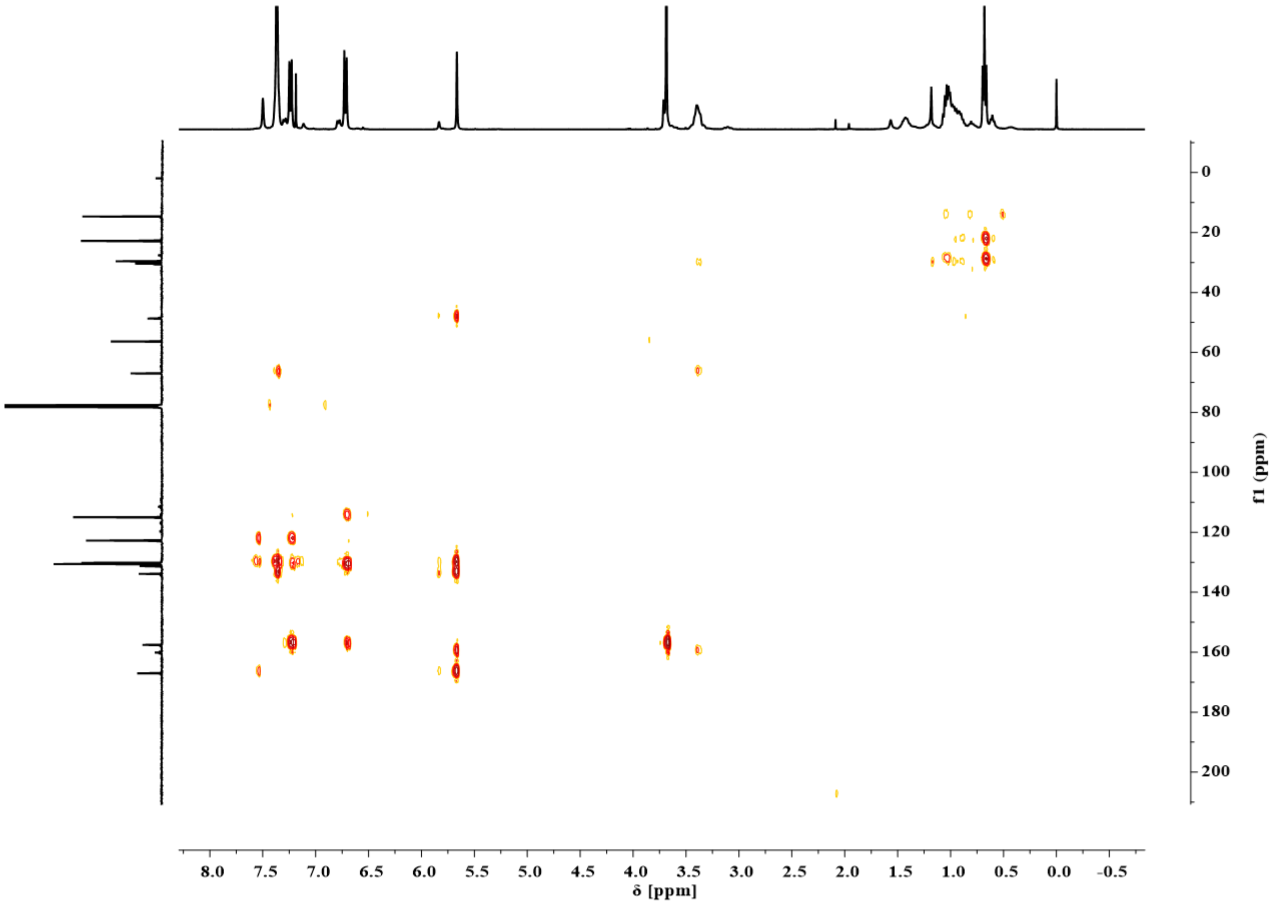

Supplementary Figure 169 | HMBC experiment of the title compound recorded in CD_3OD.

Ugi reaction of perfluorononanoic acid, dodecanal, pentylisocyanide and benzylamine

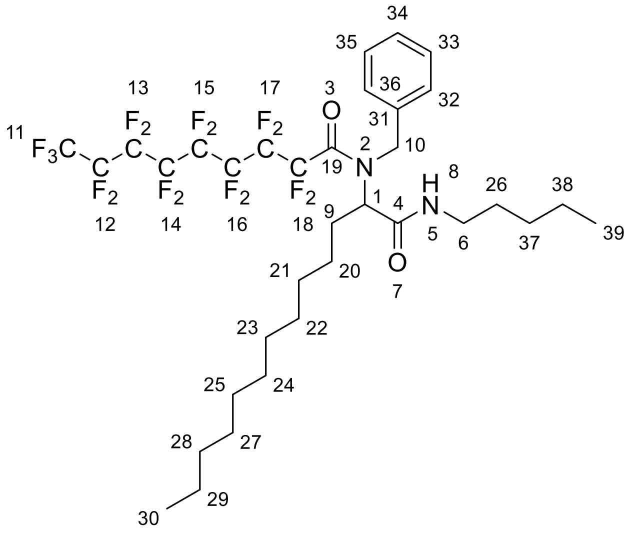

In a 25 mL round bottom flask dodecyl aldehyde (90.4 mg, 490 µmol, 1.30 eq.) was dissolved in 1.5 mL methanol, subsequently benzylamine (56.0 µL, 52.5 mg, 490 µmol, 1.30 eq.) was added and the resulting mixture was stirred for 60 min over sodium sulfate. Afterwards, the mixture was filtrated and the solid was washed with 10 mL methanol three times. Subsequently, the filtrate was concentrated under reduced pressure. Perfluorononanoic acid (175 mg, 377 µmol, 1.00 eq.) dissolved

in 1 mL methanol was added to the imine at room temperature and the resulting mixture was stirred for 2 min. Subsequently, pentylisocyanide (56.9 µL, 43.9 mg, 453 µmol, 1.20 eq.) was added to the stirring mixture. The reaction was stirred for 3 d at room temperature. The crude reaction mixture was dried under reduced pressure and purified *via* column chromatography employing Fluoro*Flash*® silica gel. The fluorous fraction was tested for purity *via* TLC and concentrated under reduced pressure. The remaining perfluoro acid was removed with a short silica gel filter column, eluting with *c*-hexane/ethyl acetate (3:1) to yield the Ugi product as a highly viscous yellow oil (57.1 mg, 68.4 µmol, 18.1%).

R_f = 0.69 in *c*-hexane/ethyl acetate (6:1). Visualized *via* fluorescent quench and Seebach staining solution.

IR (ATR): ν [cm^{-1}] = 3327.8 (br, ν(N-H)), 2924.6 (m, ν(C-H)), 2854.8 (w, ν(C-H)), 1659.9 (m, ν(C=O)), 1539.8 (w, ν(N-H)), 1455.2 (w), 1364.5 (s), 1239.1 (s), 1209.2 (vs), 1148.2 (s), 956.2 (w), 722.9 (m), 699.5 (m), 657.1 (m), 559.4 (w), 529.2 (w), 463.1 (w).

^{1}H NMR (400 MHz, $CDCl_3$): δ [ppm] = 7.38 – 7.13 (m, 5 H, $CH_{Ar}^{32\text{-}36}$), 6.17 (t, J = 5.5 Hz, 1 H, NH^{8}), 5.04 – 4.55 (m, 2 H, CH_2^{10}), 4.43 (t, J = 14.8, 1 H, CH^{1}), 3.34 – 2.92 (m, 2 H, CH_2^{6}), 1.96 – 1.67 (m, 2 H, CH_2^{9}), 1.66 – 1.37 (m, 2 H, $CH_2^{38\ or\ 29}$), 1.36 – 1.06 (m, 22 H, CH_2), 0.87 (s, 6 H, $CH_3^{30\ +\ 39}$).

^{13}C NMR (126 MHz, $CDCl_3$): δ [ppm] = 168.3 (s, $CONR^{4}$), 159.0 (s, $CONR^{19}$), 135.9 (s, C_{Ar}^{31}), 127.9 (s, CH_{Ar}), 127.3 (s, CH_{Ar}), 126.5 (s, CH_{Ar}), 60.7 (s, CH^{1}), 40.3 (s, CH_2^{6}), 31.6 (s, CH_2^{26}), 28.4 (s, CH_2), 28.3 (s, CH_2), 22.3 (s, CH_2), 21.9 (s, CH_2), 13.8 (s, $CH_3^{30\ or\ 39}$), 13.6 (s, $CH_3^{30\ or\ 39}$).

^{19}F NMR (376 MHz, $CDCl_3$) δ [ppm] = -85.10 (t, J = 9.9 Hz, 3 F, CF_3^{11}), -112.37 – -114.22 (m, CF_2^{18a}), AB-signal (δ_A = -114.15, δ_B = -115.22, J_{AB} = 291.8 Hz, A and B are split into t, J = 13.1 Hz, CF_2^{18b}), 124.25 (s, CF_2), -126.13 (s, CF_2), -127.05 (s, CF_2), -130.44 (s, CF_2^{12}). Total integral of CF_2 region normalized with respect to the CF_3^{11} group = 14.

FAB – MS [*m/z*] (relative intensity): 835.4 (65%) $[M + H]^+$, 387.3 (10%) [Fragment A]$^+$, 283.2 (32%) [Fragment B + H]$^+$.

HRMS – FAB [*m/z*]: $[M + H]^+$ calculated for $^{12}C_{34}{}^{1}H_{44}{}^{16}O_2{}^{14}N_2{}^{19}F_{17}$, 835.3126; found, 835.3125; Δ = 0.06 mmu.

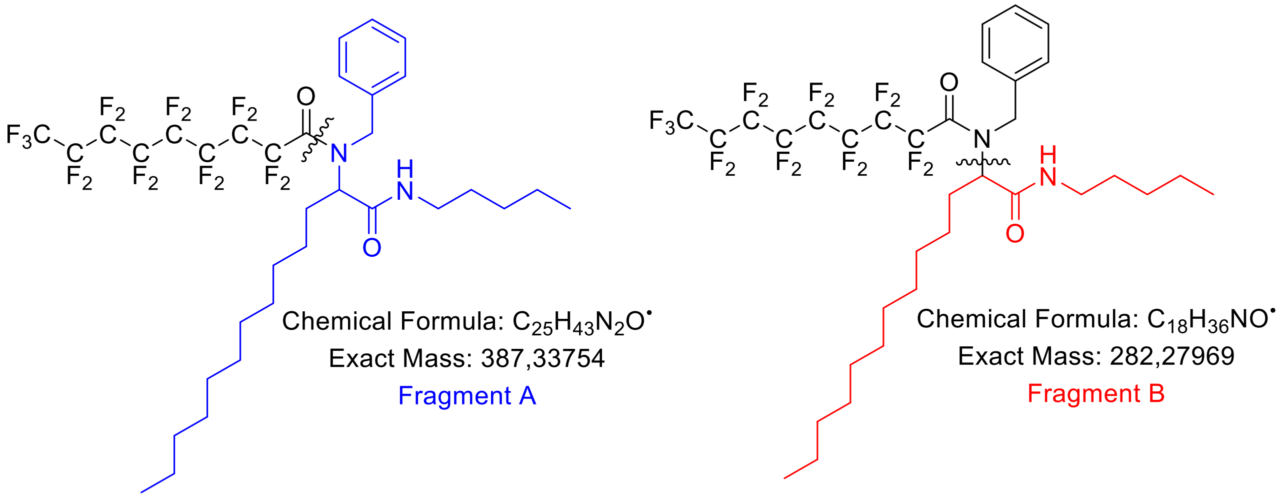

Supplementary Figure 170 | Proposed fragments observed in FAB-MS.

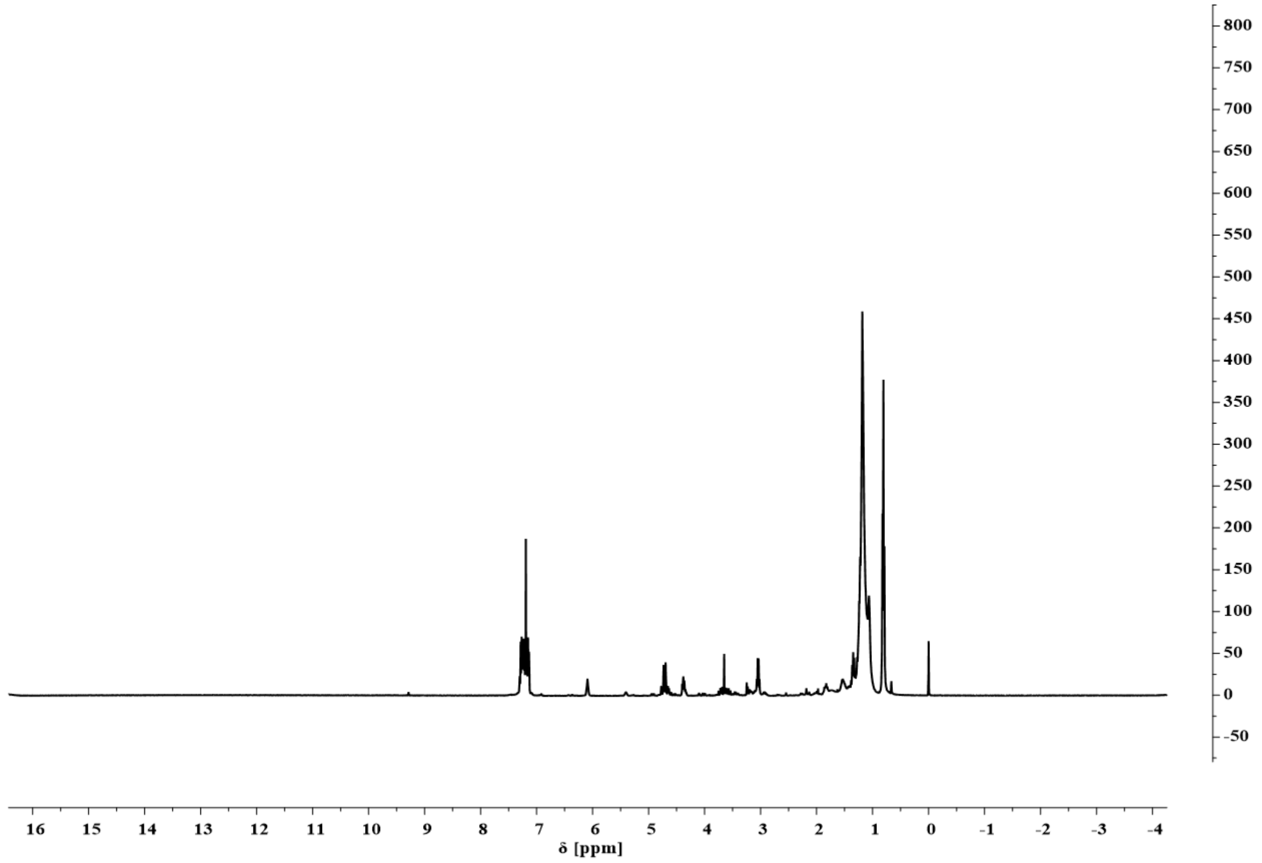

Supplementary Figure 171 | ¹H NMR experiment of the title compound recorded in CD₃OD.

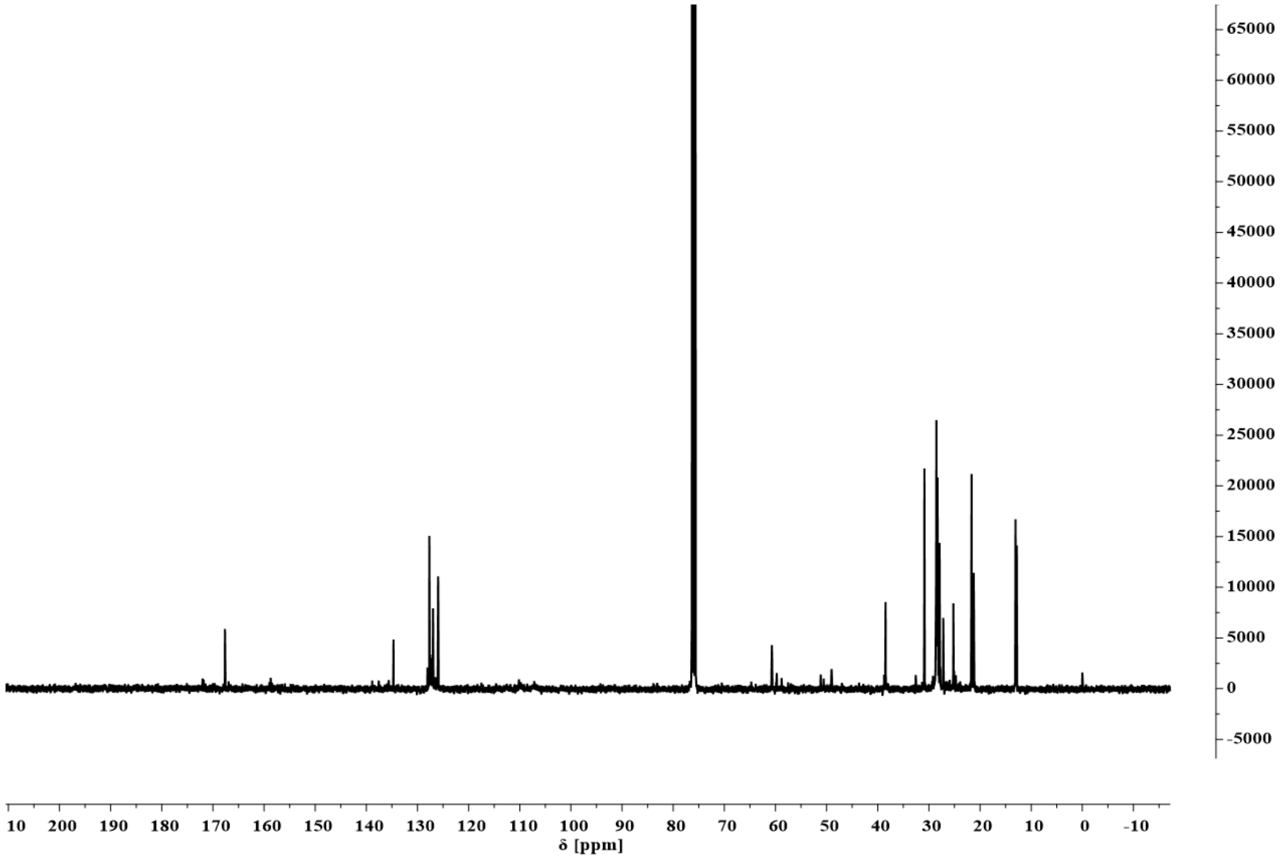

Supplementary Figure 172 | ^{13}C NMR experiment of the title compound recorded in CD_3OD.

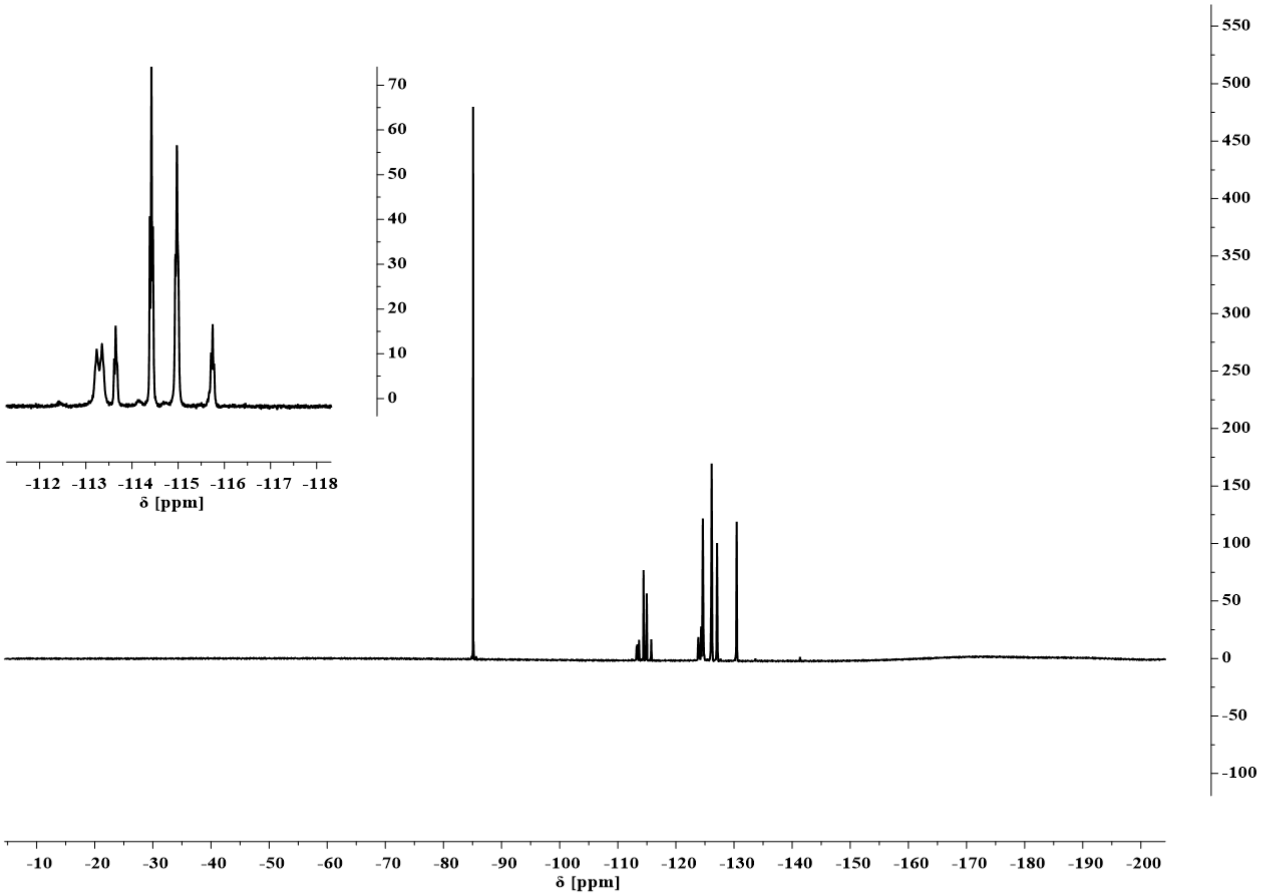

Supplementary Figure 173 | ^{19}F NMR experiment of the title compound recorded in CD_3OD.

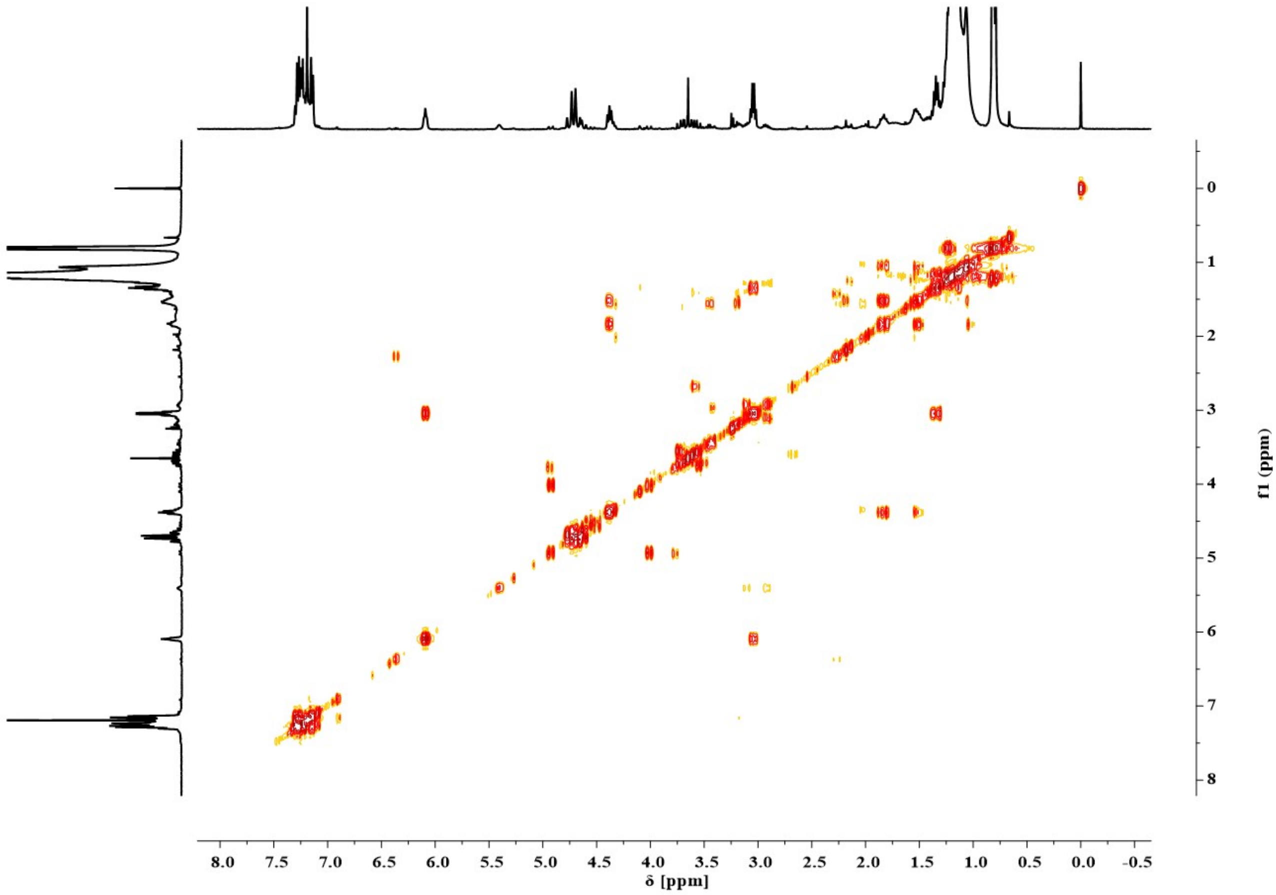

Supplementary Figure 174 |COSY experiment of the title compound recorded in CD_3OD.

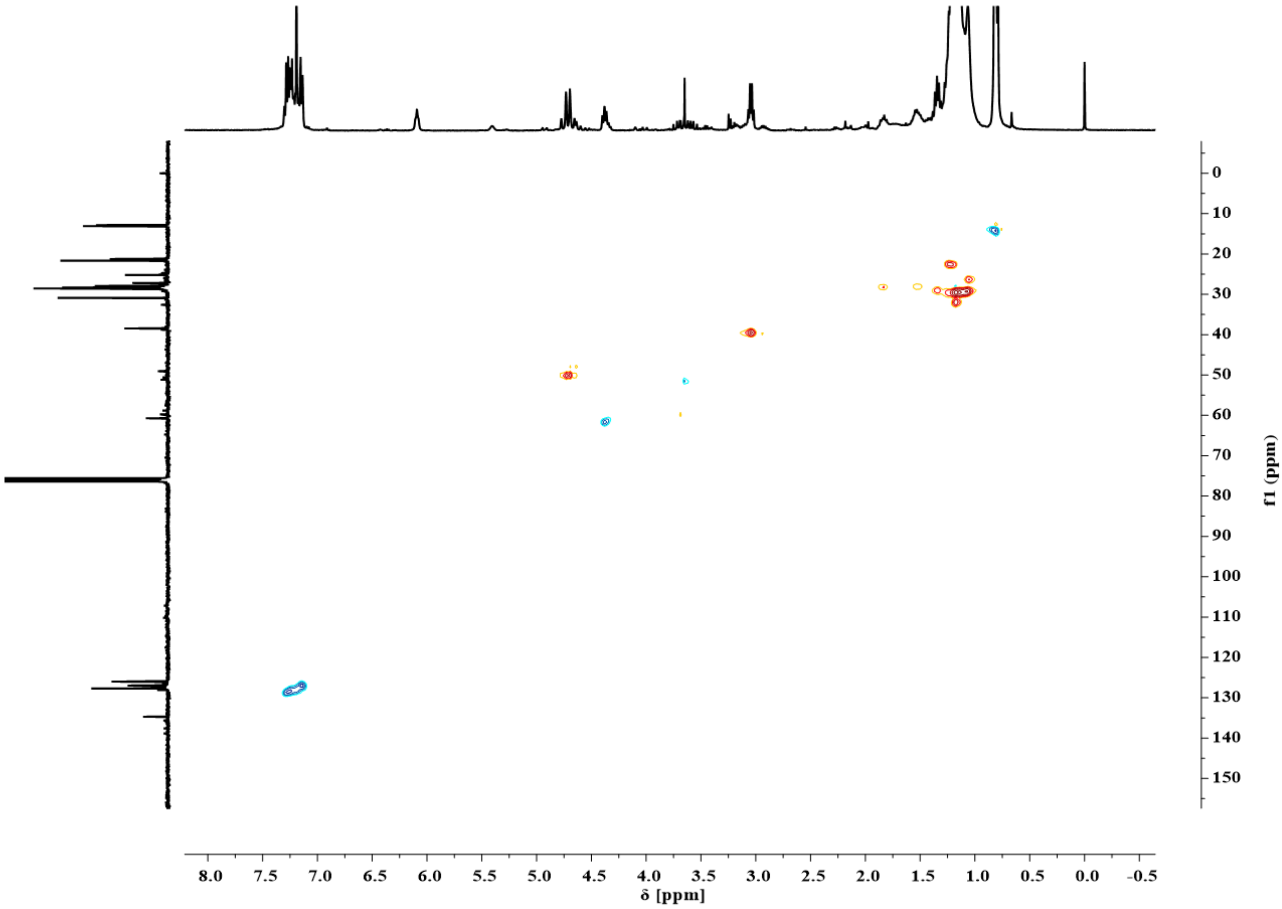

Supplementary Figure 175 | Multiplicity-edited HSQC experiment of the title compound recorded in CD_3OD.

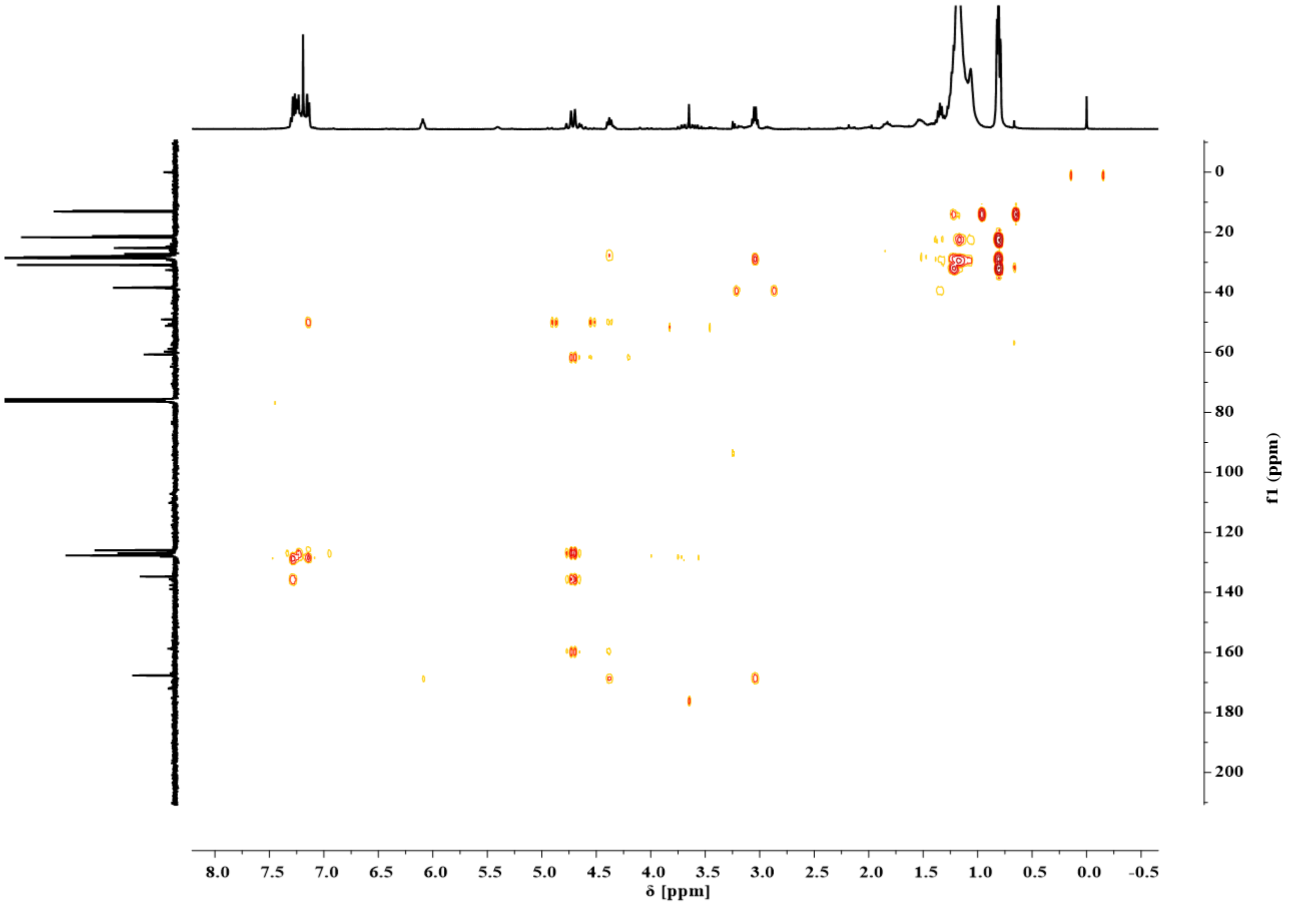

Supplementary Figure 176 | HMBC experiment of the title compound recorded in CD_3OD.

Ugi reaction of perfluorononanoic acid, undec-10-enal, cyclohexylamine and benzylisocyanide

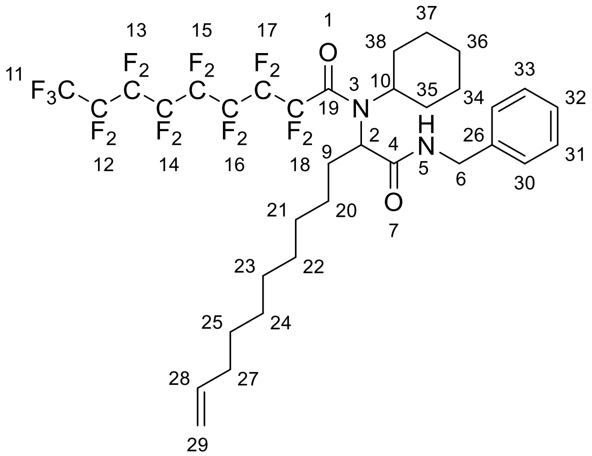

In a 25 mL round bottom flask undec-10-enal (97.6 µL, 84.5 mg, 490 µmol, 1.30 eq.) was dissolved in 1.5 mL methanol, subsequently cyclohexylamine (56.5 µL, 48.9 mg, 490 µmol, 1.30 eq.) was added and the resulting mixture was stirred for 60 min over sodium sulfate. Afterwards the mixture was filtrated and the solid was washed with 10 mL methanol three times. Subsequently, the filtrate was concentrated under reduced pressure. Perfluorononanoic acid (175 mg, 377 µmol, 1.00 eq.) was

added at room temperature and the resulting mixture stirred for 2 min. Subsequently, benzylisocyanide (53.9 µL, 53.0 mg, 453 µmol, 1.20 eq.) was added to the stirring mixture. The reaction was stirred for 5 d at room temperature. The crude reaction mixture was dried under reduced pressure and purified *via* column chromatography employing silica gel and eluted with a gradual solvent mixture of ethyl acetate and *c*-hexane (0:1 → 1:3) to remove the remaining perfluoro acid. The product containing fractions were collected and further purified *via* column chromatography employing Fluoro*Flash*® silica gel to yield the Ugi product as a highly viscous yellow oil (59.4 mg, 71.5 µmol, 19.0%).

R_f = 0.50 in *c*-hexane/ethyl acetate (6:1). Visualized *via* fluorescent quench and Seebach staining solution.

IR (ATR): ν [cm^{-1}] = 3324.4 (br, ν(N-H)), 2926.4 (s, ν(C-H)), 2855.8 (m, ν(C-H)), 1663.4 (s, ν(C=O)), 1528.8 (w, ν(C=C)), 1455.2 (w), 1364.2 (w), 1238.6 (vs), 1208.1 (vs), 1147.9 (s), 1029.0 (w), 992.2 (w), 909.4 (m), 723.2 (s), 698.2 (s), 655.9 (m), 559.2 (m), 528.9 (m).

^{1}H NMR (400 MHz, $CDCl_3$): δ [ppm] = 7.65 – 6.99 (m, 5 H, CH_{Ar}^{30-34}), 6.70 (t, J = 5.7 Hz, 1 H, NH^{5}), 5.95 – 5.64 (m, 2 H, CH_2^{29}), 5.11 – 4.83 (m, 1 H, CH^{28}), 4.58 (s, 1 H, CH^{2}), 4.50 – 4.26 (m, 3 H, CH_2^{6} + CH^{10}), 3.61 – 3.16 (m, 2 H, CH_2^{9}) 2.17 – 1.94 (m, 4 H, CH_2), 1.88 – 1.42 (m, 4 H, CH_2), 1.41 – 1.03 (m, 16 H, CH_2).

^{13}C NMR (101 MHz, $CDCl_3$): δ [ppm] = 170.4 (s, $CONR^{4}$), 160.5 (s, $CONR^{19}$), 140.4 (s, CH_2^{29}), 138.6 (s, C_{Ar}^{26}), 129.6 (s, CH_{Ar}), 128.5 (s, CH_{Ar}), 128.4 (s, CH_{Ar}), 115.0 (s, CH^{28}), 62.8 (s, $CH^{2\ or\ 10}$), 61.9 (s, $CH^{2\ or\ 10}$), 47.5 (s, CH_2), 44.5 (s, CH_2^{6}), 34.7 (s, CH_2), 32.1 (s, CH_2), 30.9 (s, CH_2), 30.6 (s, CH_2), 30.3 (s, CH_2), 30.2 (s, CH_2), 29.9 (s, CH_2), 29.5 (s, CH_2), 28.7 (s, CH_2), 27.7 (s, CH_2), 27.4 (s, CH_2), 26.9 (s, CH_2).

^{19}F NMR (376 MHz, $CDCl_3$): δ [ppm] = -85.11 (t, J = 10.3 Hz, 3 F, CF_3^{11}), AB-signal (δ_A = -112.77, δ_B = -113.30, J_{AB} = 299.3 Hz, A and B are split into t, J = 12.7 Hz, CF_2^{18a}), AB-signal (δ_A = -115.50, δ_B = -115.86, J_{AB} = 289.9 Hz, A and B are split into t, J = 13.2 Hz, CF_2^{18b}), -124.62 (s, CF_2), -126.11 (s, CF_2), -127.05 (s, CF_2), -130.44 (s, CF_2^{12}). Total integral of CF_2 region normalized with respect to the CF_3^{11} group = 14.

FAB – MS [*m/z*] (relative intensity): 831.4 (45%) $[M + H]^+$, 726.3 (73%) $[\text{Fragment A} + H]^+$, 106.0 (17%) $[\text{Fragment B}]^+$.

HRMS – FAB [*m/z*]: $[M + H]^+$ calculated for $^{12}C_{34}{}^{1}H_{40}{}^{16}O_2{}^{14}N_2{}^{19}F_{17}$, 831.2813; found, 821.2814; Δ = 0.13 mmu.

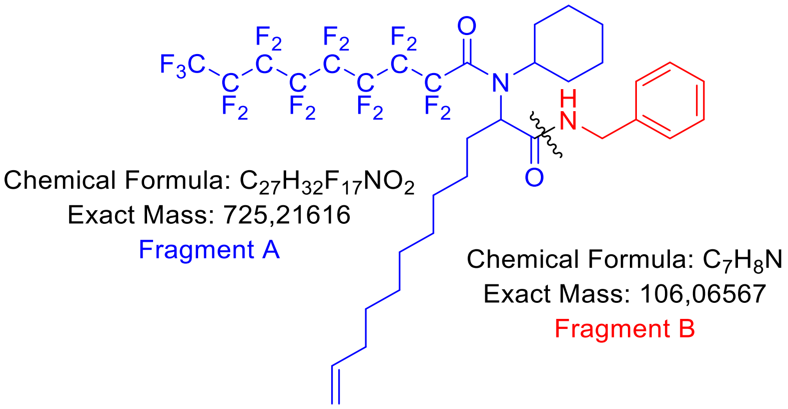

Supplementary Figure 177 | Proposed fragments observed in FAB-MS.

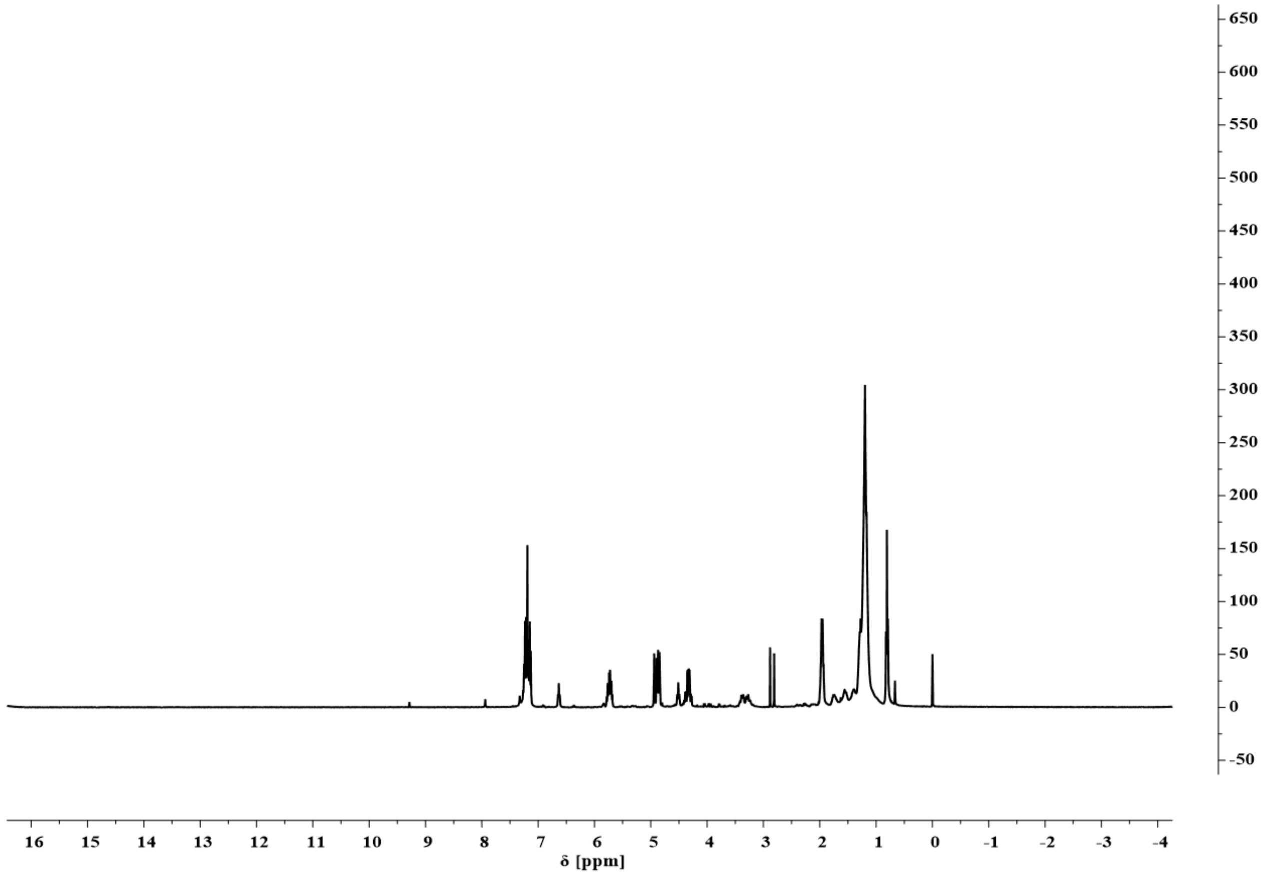

Supplementary Figure 178 | ^{1}H NMR experiment of the title compound recorded in $CDCl_3$.

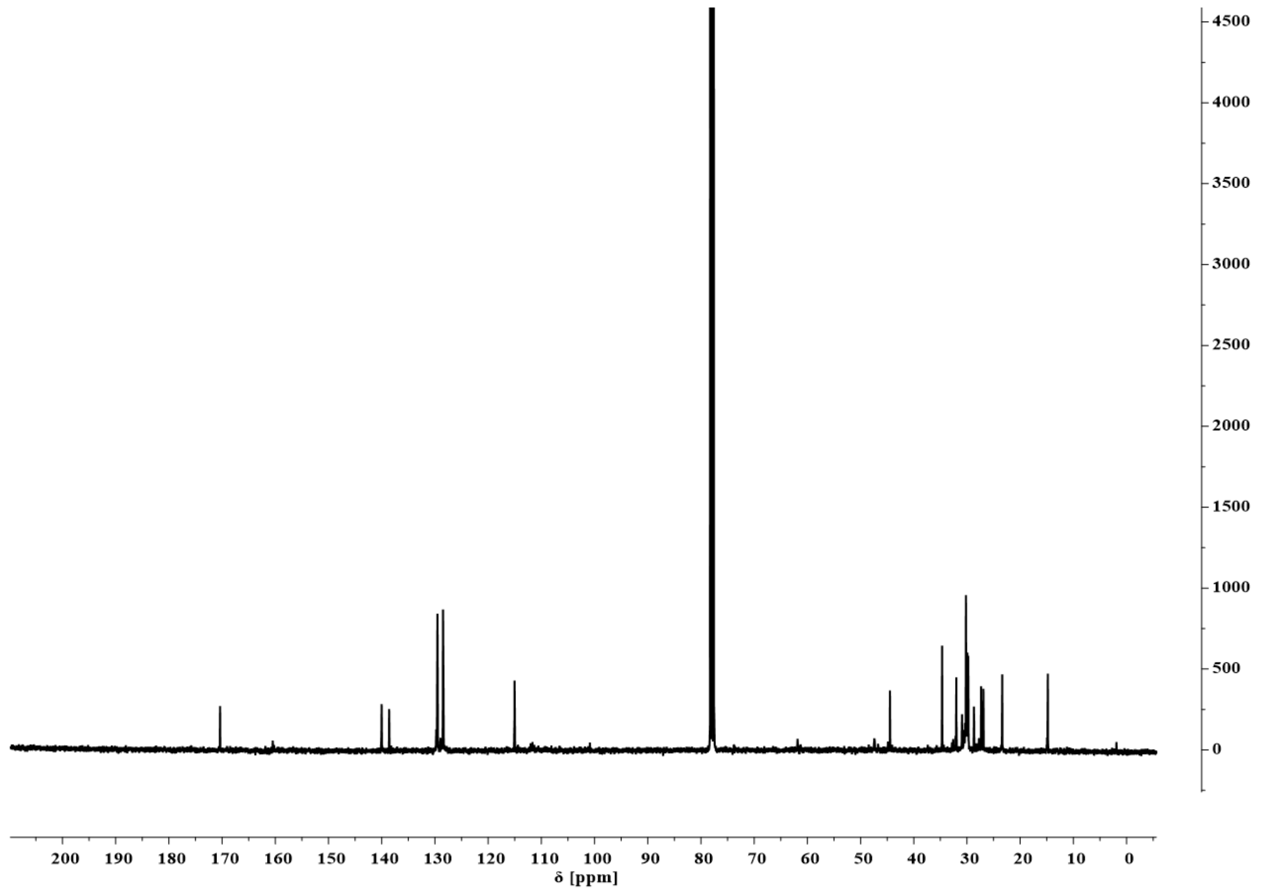

Supplementary Figure 179 | ^{13}C NMR experiment of the title compound recorded in $CDCl_3$.

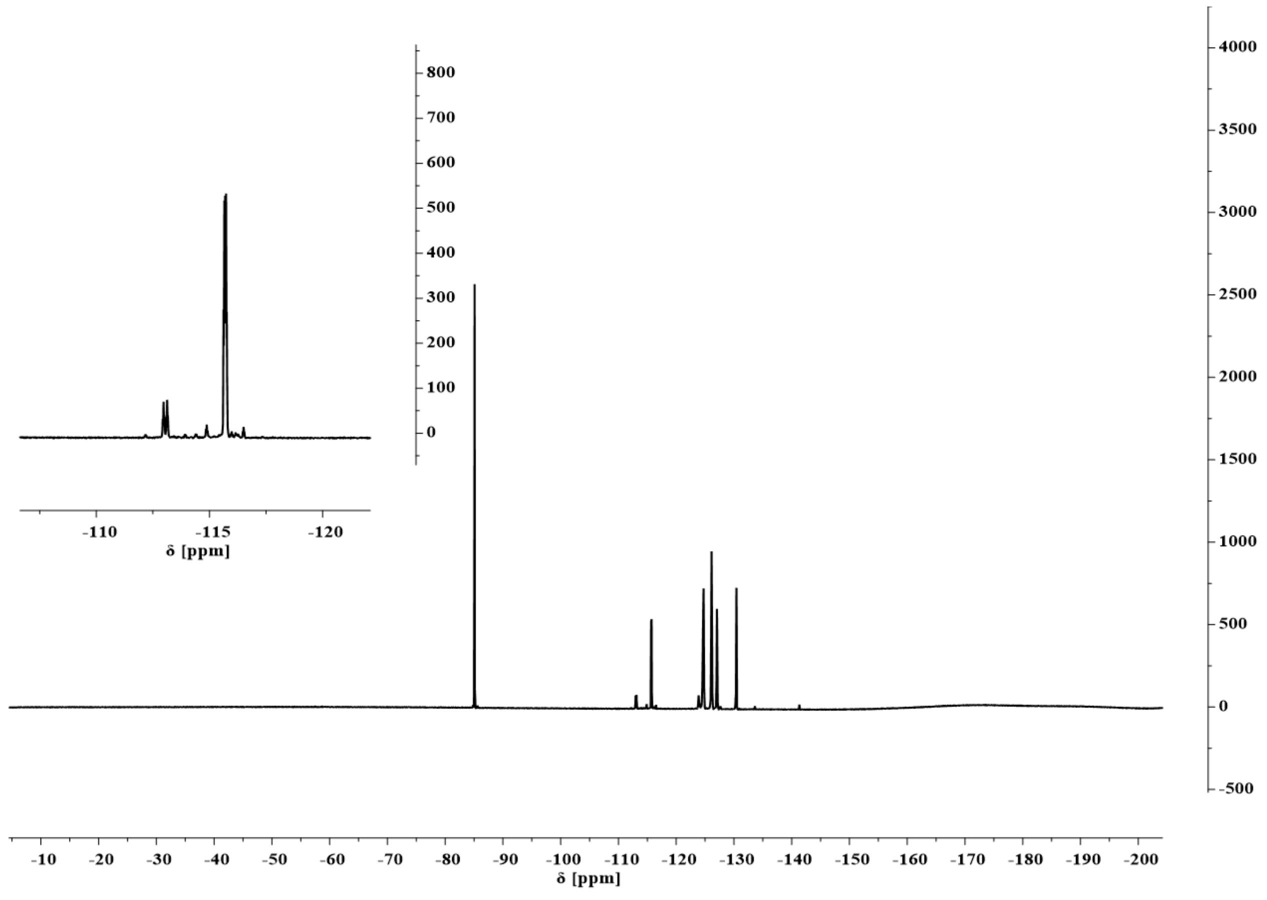

Supplementary Figure 180 | ^{19}F NMR experiment of the title compound recorded in $CDCl_3$.

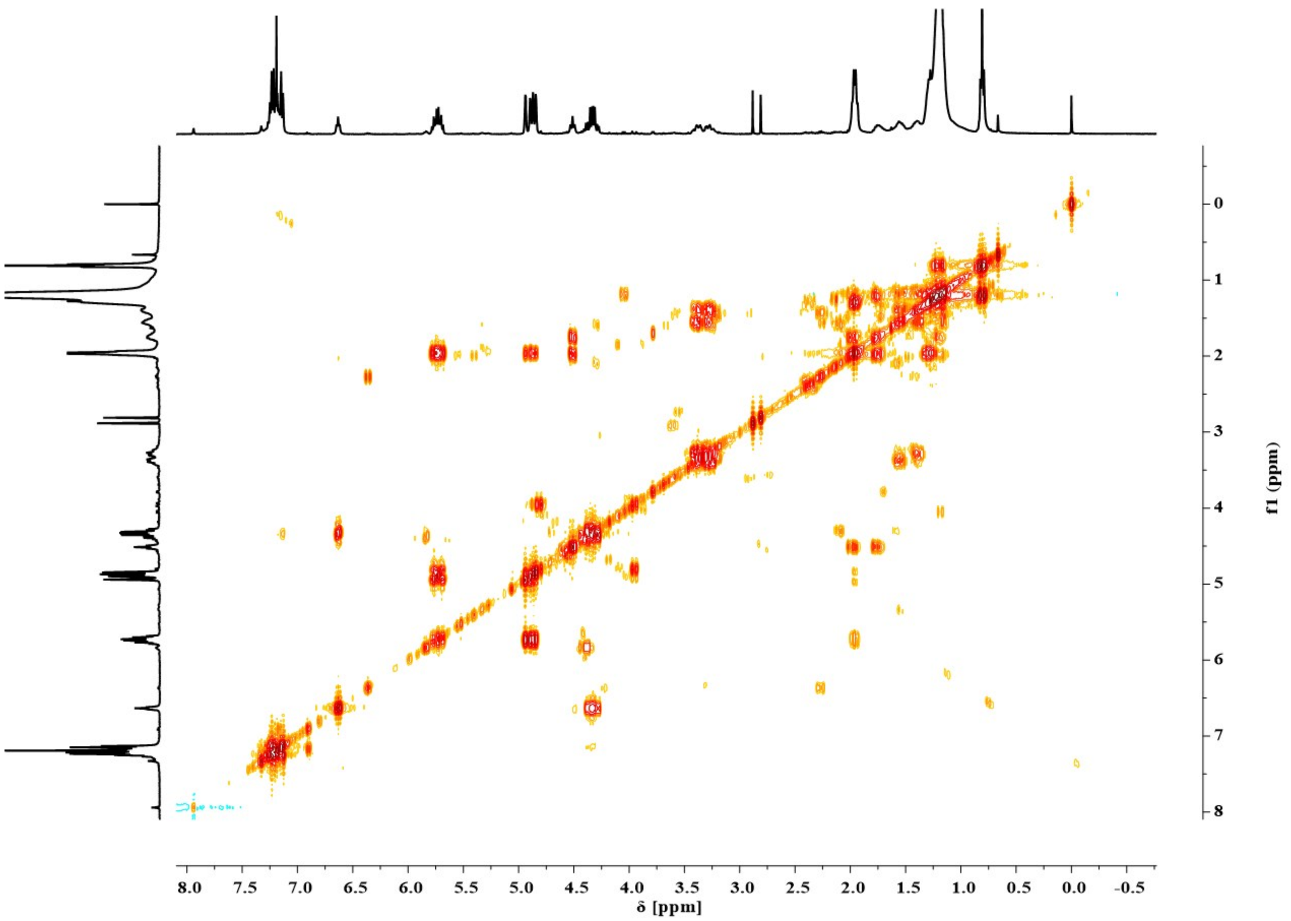

Supplementary Figure 181 | COSY experiment of the title compound recorded in $CDCl_3$.

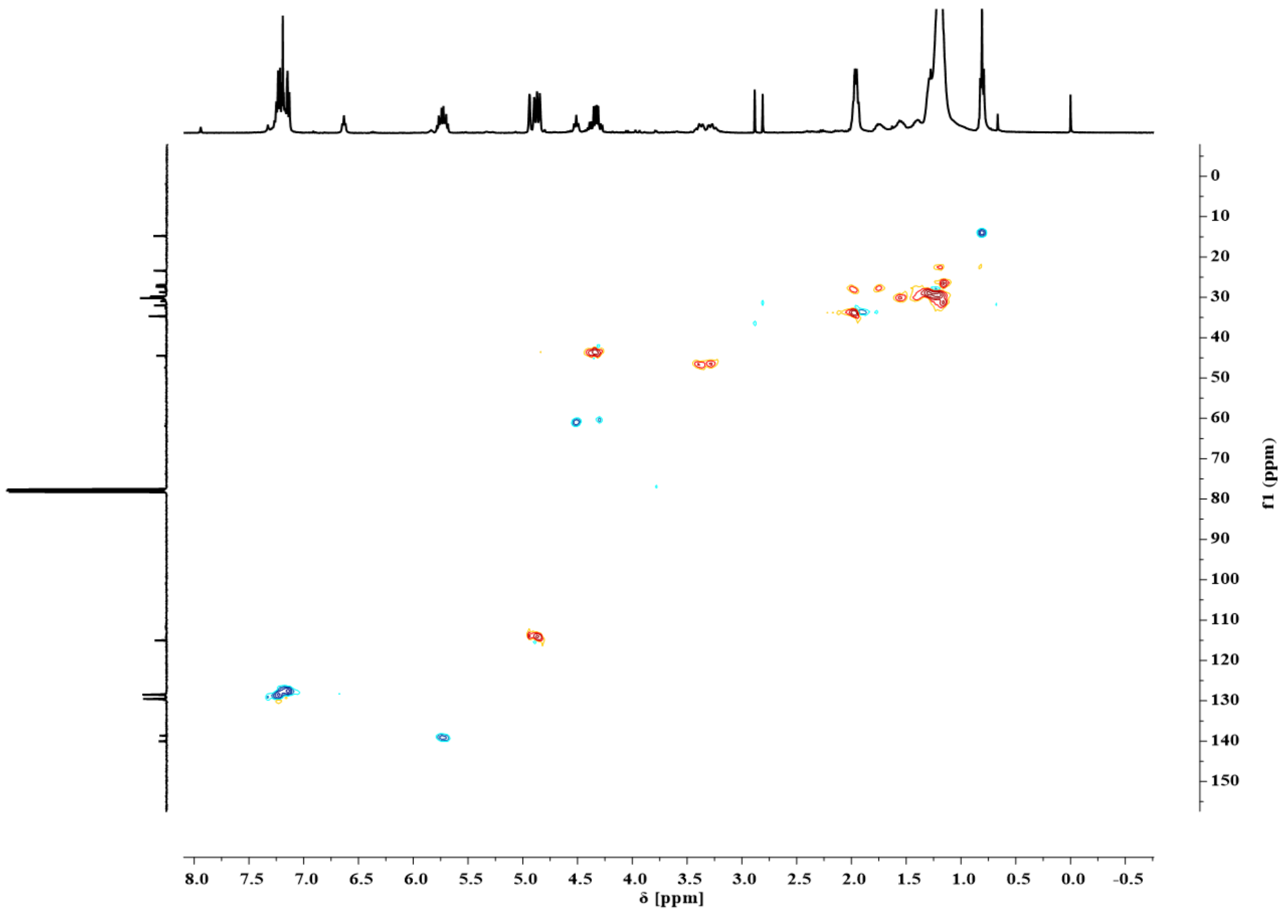

Supplementary Figure 182 | Multiplicity-edited HSQC experiment of the title compound recorded in $CDCl_3$.

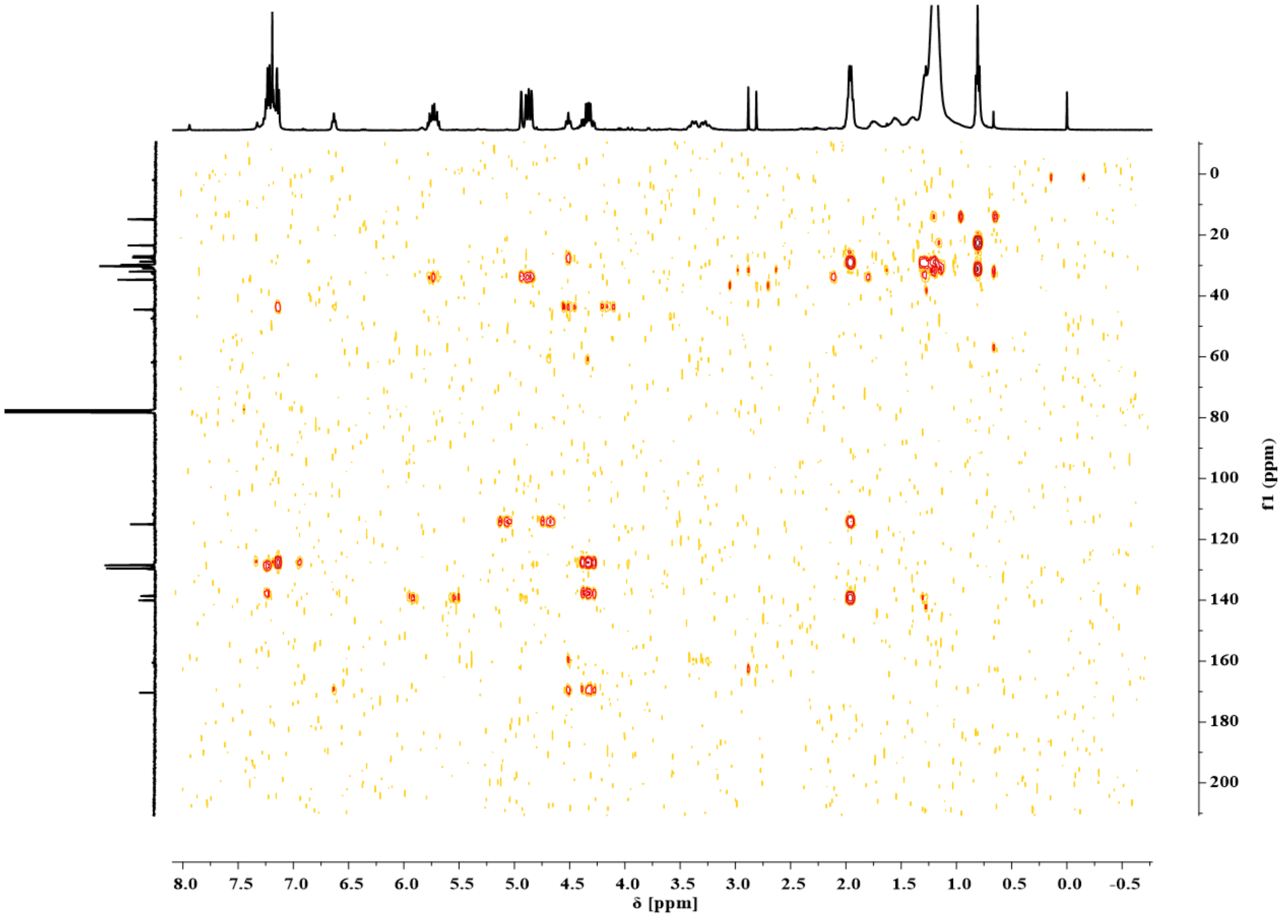

Supplementary Figure 183 | HMBC experiment of the title compound recorded in CD_3OD.

Ugi reaction of perfluorononanoic acid, 4-hydroxybenzaldehyde, cyclohexylisocyanide and propargylamine

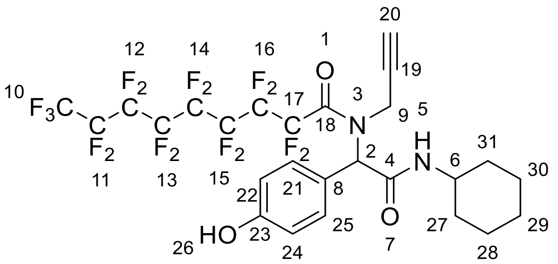

In a 25 mL round bottom flask 4-hydroxybenzaldehyd (59.6 mg, 489 µmol, 1.30 eq.) was dissolved in 1.5 mL methanol, subsequently propargylamine (31.4 µL, 27.0 mg, 490 µmol, 1.30 eq.) was added and the resulting mixture was stirred for 60 min over sodium sulfate. Afterwards, the mixture was filtrated. The solid was washed with 10 mL methanol three times. Subsequently, the filtrate was concentrated under reduced pressure. Perfluorononanoic acid (175 mg, 377 µmol, 1.00 eq.) dissolved in 1 mL methanol was added to the imine at room temperature and the resulting mixture was stirred for 2 min. Subsequently, cyclohexylisocyanide (56.3 µL, 49.4 mg, 453 µmol, 1.20 eq.) was added to the stirring mixture. The reaction was stirred for 1 d at room temperature. The crude reaction mixture

was dried under reduced pressure and purified *via* column chromatography employing Fluoro*Flash*® silica gel. The fluorous fraction was tested for purity *via* TLC and concentrated under reduced pressure. The Ugi product was obtained as a yellow oil (93.9 mg, 128 µmol, 34.1%).

R_f = 0.66 in *c*-hexane/ethyl acetate (6:1). Visualized *via* fluorescent quench and Seebach staining solution.

IR (ATR): ν [cm^{-1}] = 3301.6 (br, ν(N-H)), 3103.4 (br, ν(O-H)), 2928.2 (w, ν(C-H)), 2855.1 (w, ν(C-H)), 1650.1 (m, ν(C=O)), 1614.5 (w), 1598.5 (w), 1566.03 (m), 1514.2 (m), 1452.4 (w), 1425.6 (w), 1367.3 (w), 1347.2 (w), 1200.5 (s), 1149.3 (vs), 988.9 (w), 945.4 (m), 891.3 (w), 864.1 (w), 837.2 (w), 821.1 (w), 806.3 (w), 806.3 (w), 769.8 (w), 712.8 (m), 678.2 (m), 638.9 (s), 558.5 (m), 544.7 (m), 515.6 (s), 451.2 (w), 440.3 (w), 415.3 (w).

^{1}H NMR (400 MHz, CD_3OD): δ [ppm] = 7.20 (dd, J = 25.7, 7.3 Hz, 2 H, $CH_{Ar}^{22,24}$), 6.86 (d, J = 7.3 Hz, 2 H, $CH_{Ar}^{21,25}$), 5.89 (d, J = 17.6 Hz, 1 H, CH^{2}), 4.41 – 3.93 (m, 2 H, CH_2^{9}), 3.77 – 3.57 (m, 1 H, CH^{6}), 1.97 – 1.06 (m, 11 H, CH^{20} + CH_2).

^{13}C NMR (126 MHz, CD_3OD): δ [ppm] = 170.1 (s, $CONR^{4}$), 159.8 (s, $CONR^{18}$), 141.0 (s, C_{Ar}^{23}), 133.2 (s, $CH_{Ar}^{22, 24}$), 125.0 (s, C_{Ar}^{8}), 116.7 (s, $CH_{Ar}^{21, 25}$), 73.0 (s, C^{19}), 64.5 (s, CH^{2}), 48.8 (s, CH^{6}), 36.9 (s, CH_2^{9}), 33.5 (s, CH_2), 26.6 (s, 2 CH_2), 26.0 (s, 2 CH_2), 24.4 (s, CH^{20}).

^{19}F NMR (376 MHz, CD_3OD): δ [ppm] = -86.69 (t, J = 10.3 Hz, 3 F, CF_3^{10}), AB-signal (δ_A = -114.86, δ_B = -116.45, J_{AB} = 286.1 Hz, A and B are split into t, CF_2^{17a}, additional coupling not resolved, signals broadened), AB-signal (δ_A = -116.24, δ_B = -117.50, J_{AB} = 293.6 Hz, A and B are split into t, CF_2^{17b}, additional coupling not resolved, signals broadened), - 125.00 (s, CF_2), -125.59 (m, CF_2), -127.10 (s, CF_2), -128.07 (s, CF_2), -131.60 (s, CF_2^{11}). Total integral of CF_2 region normalized with respect to the CF_3^{10} group = 14.

FAB – MS [*m/z*] (relative intensity): 733.2 (65%) $[M + H]^+$, 606.0 (75%) [Fragment A]$^+$, 568.0 (22%) [Fragment A + H – C_3H_3]$^+$, 232.1 (83%) [Fragment B]$^+$.

HRMS – FAB [*m/z*]: $[M + H]^+$ calculated for $^{12}C_{26}{}^{1}H_{22}{}^{16}O_{3}{}^{14}N_{2}{}^{19}F_{17}$, 733.1353; found, 733.1352; Δ = 0.14 mmu.

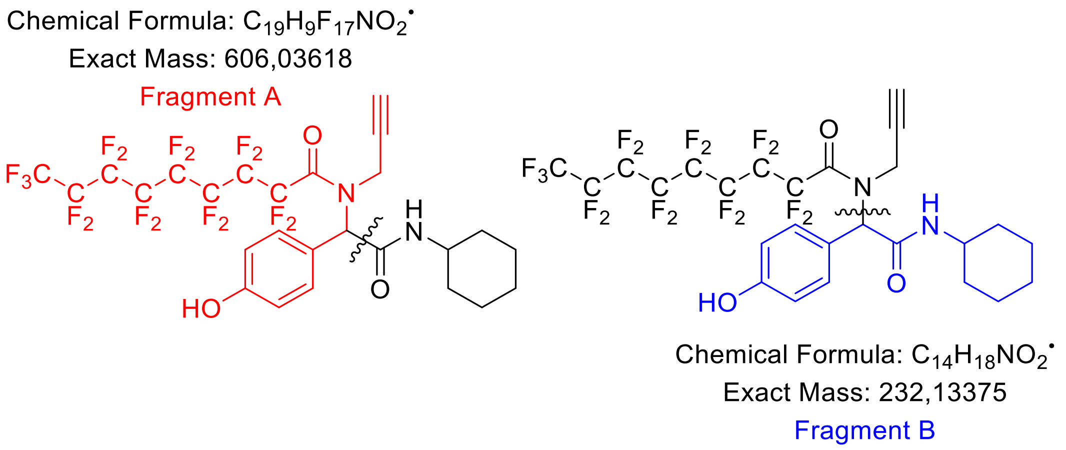

Supplementary Figure 184 | Proposed fragments observed in FAB-MS.

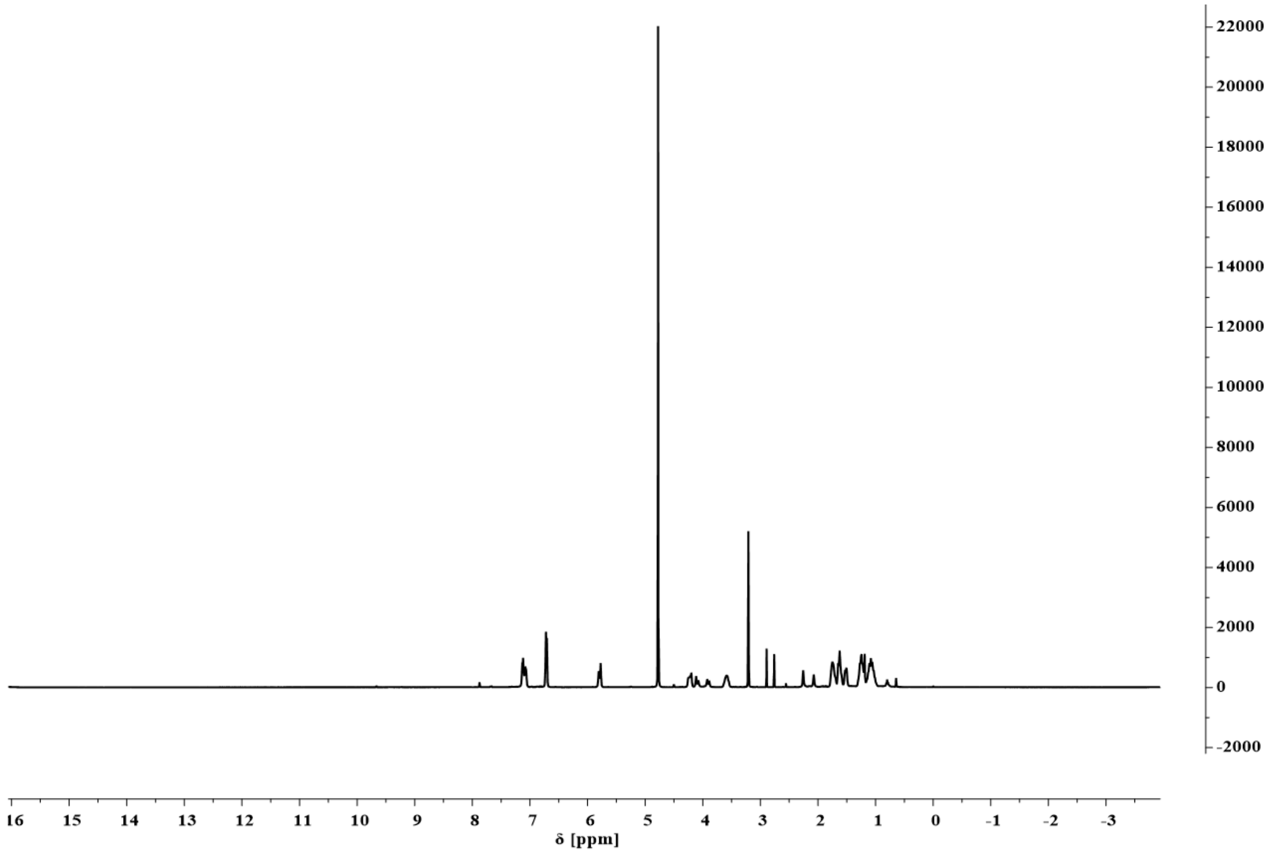

Supplementary Figure 185 | ^{1}H NMR experiment of the title compound recorded in $CDCl_3$.

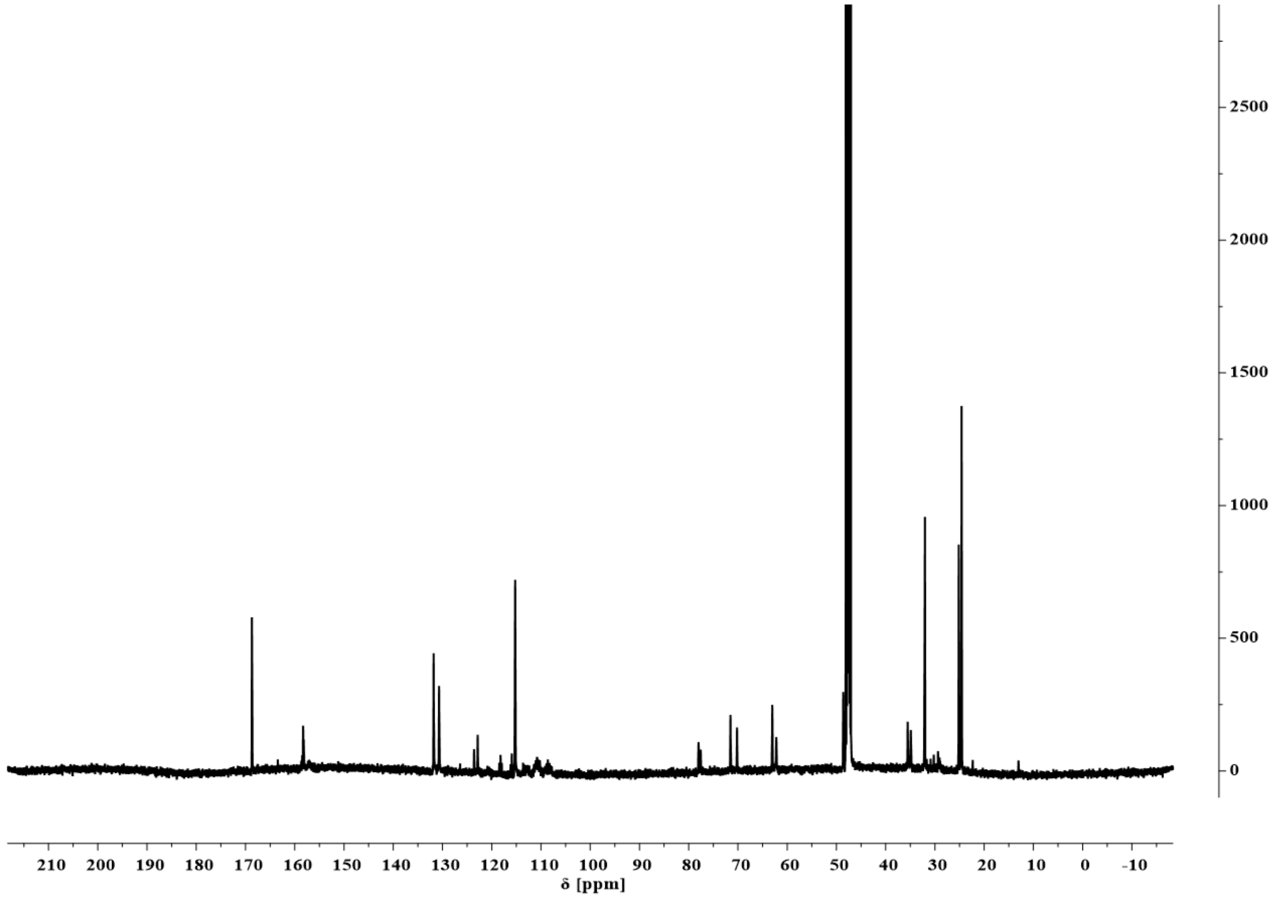

Supplementary Figure 186 | ^{13}C NMR experiment of the title compound recorded in $CDCl_3$.

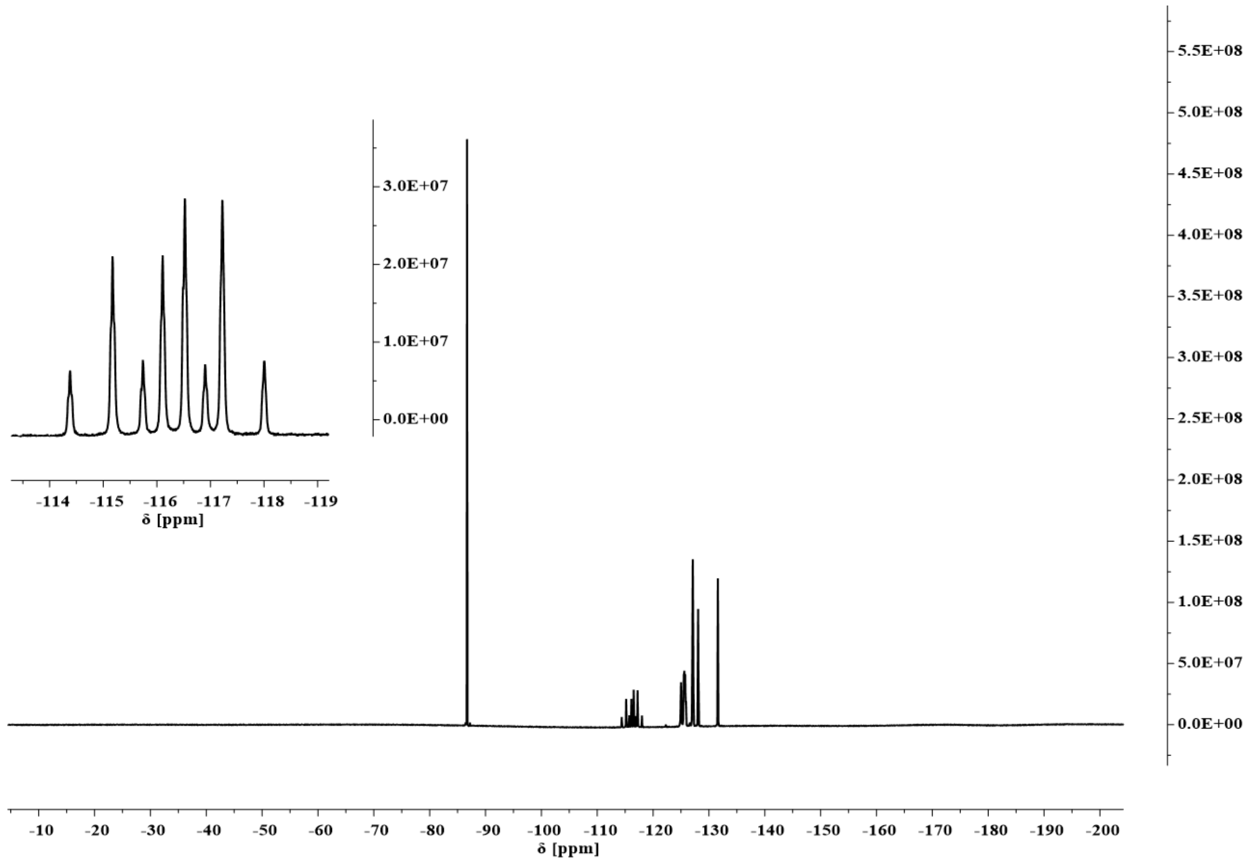

Supplementary Figure 187 | ^{19}F NMR experiment of the title compound recorded in $CDCl_3$.

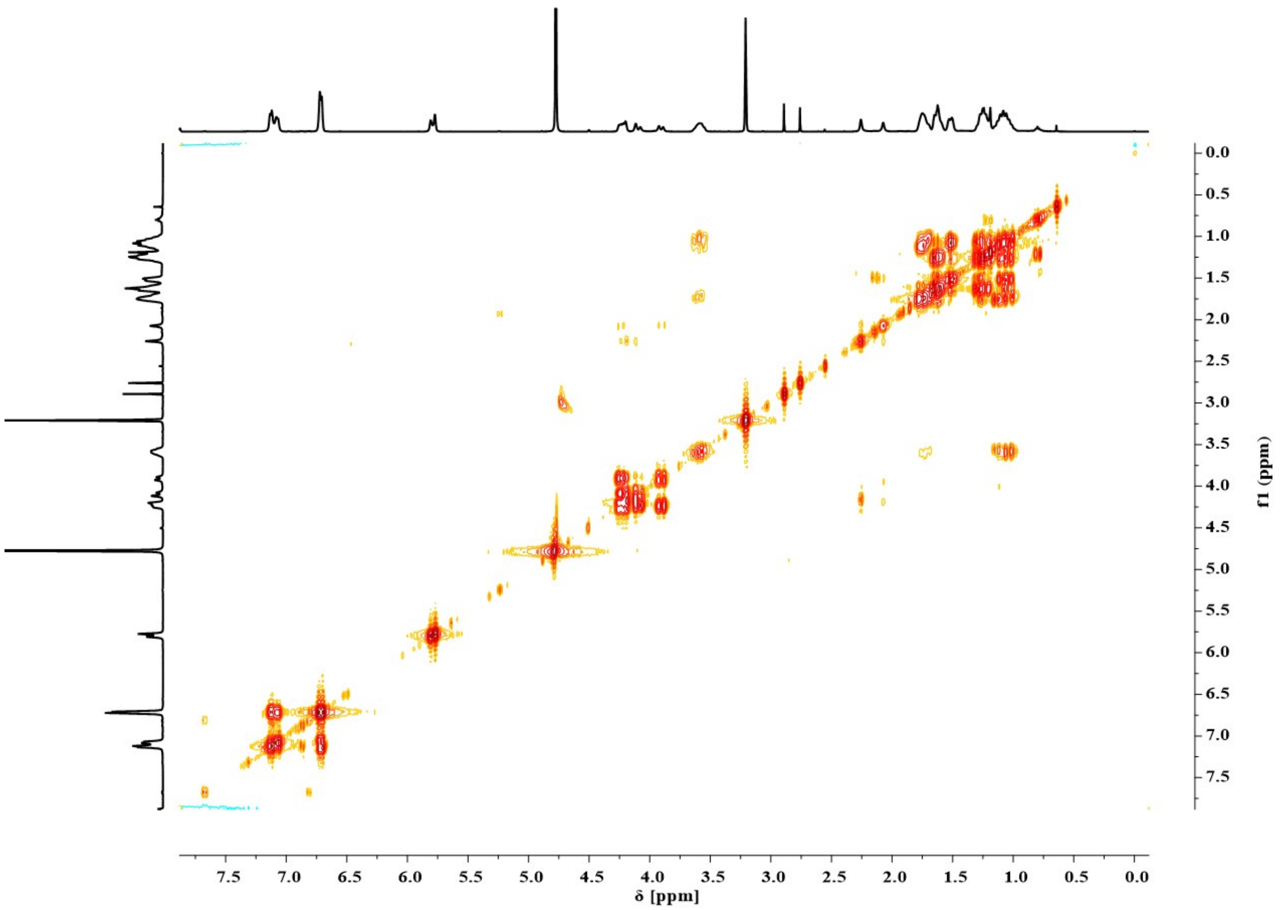

Supplementary Figure 188 | COSY experiment of the title compound recorded in $CDCl_3$.

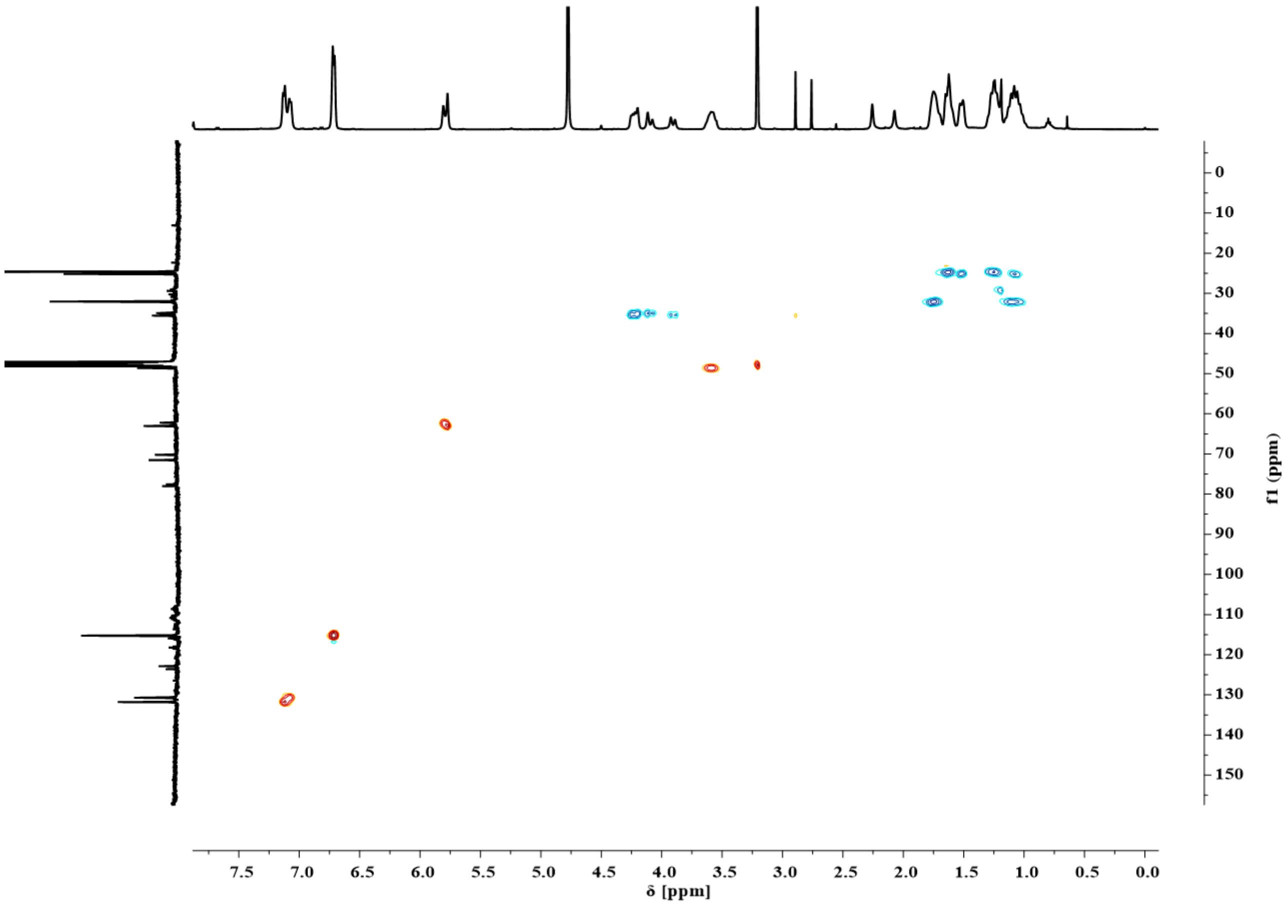

Supplementary Figure 189 | Multiplicity-edited HSQC experiment of the title compound recorded in

CDCl₃.

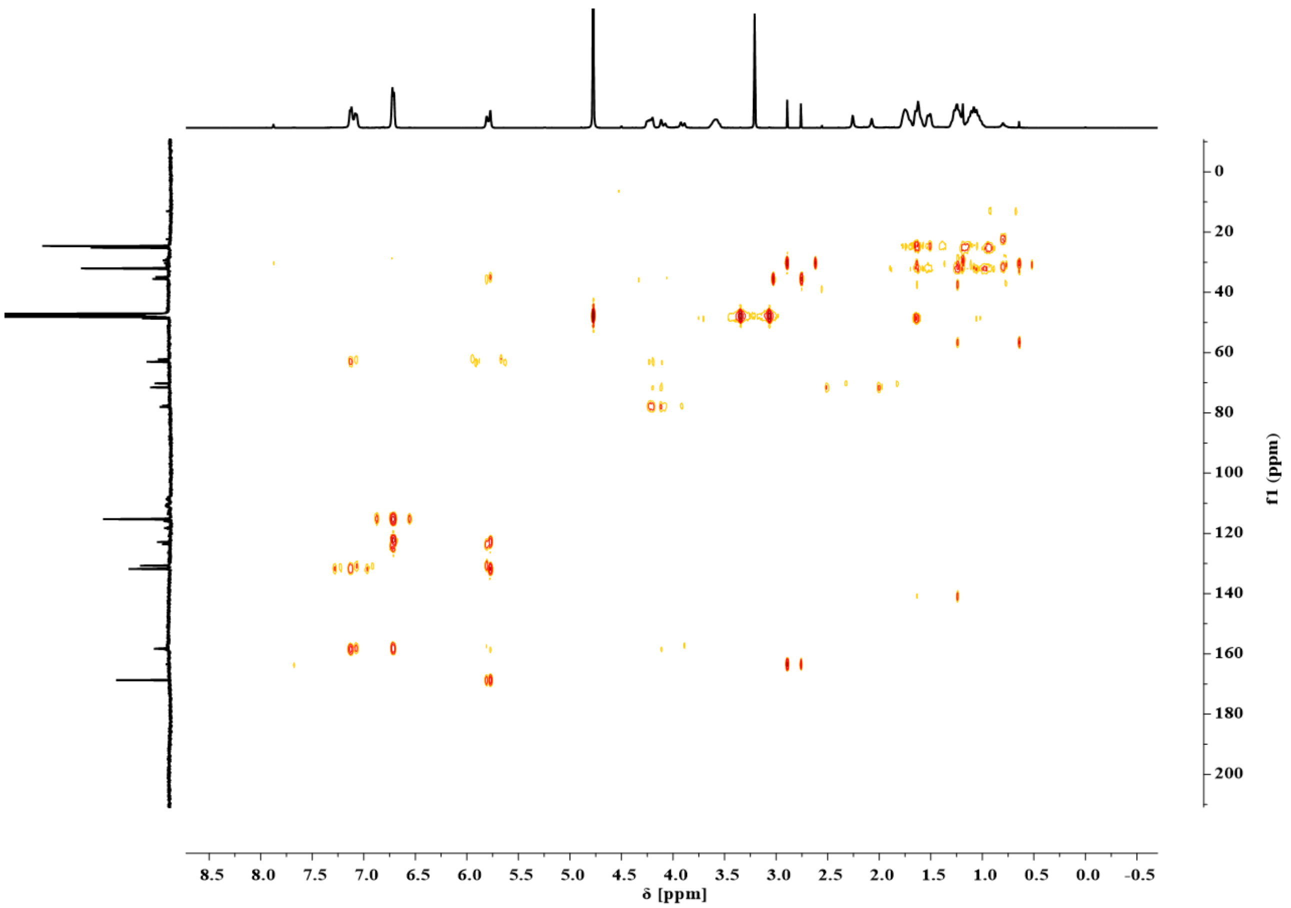

Supplementary Figure 190 | HMBC experiment of the title compound recorded in $CDCl_3$.

Ugi reaction of perfluorononanoic acid, heptanal, 4-methoxyphenylisocyanide and butylamine

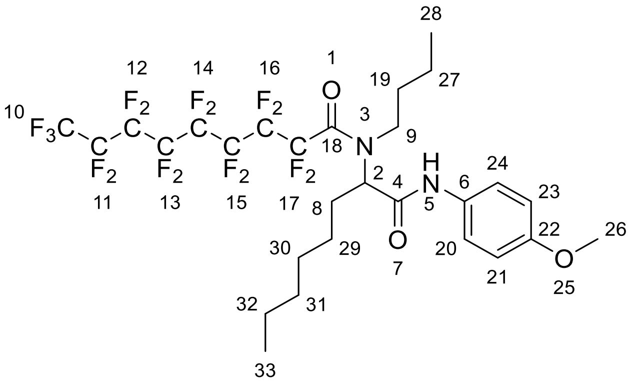

In a 25 mL round bottom flask heptanal (71.0 µL, 56.0 mg, 490 µmol, 1.30 eq.) was dissolved in 1.5 mL methanol, subsequently butylamine (48.5 µL, 35.9 mg, 490 µmol, 1.30 eq.) was added and the resulting mixture was stirred for 60 min over sodium sulfate. Afterwards, the mixture was filtrated and the solid was washed with 10 mL methanol three times. Subsequently, the filtrate was concentrated under reduced pressure. Perfluorononanoic acid (175 mg, 377 µmol, 1.00 eq.) dissolved in 1 mL methanol was added to the imine at room temperature and the resulting mixture was stirred

for 2 min. Subsequently, 4-methoxypehnylisocyanide (50.4 µL, 60.3 mg, 453 µmol, 1.20 eq.) was added to the stirring mixture. The reaction was stirred for 3 d at room temperature. The crude reaction mixture was dried under reduced pressure and purified *via* column chromatography employing Fluoro*Flash*® silica gel. The fluorous fraction was tested for purity *via* TLC and concentrated under reduced pressure. The remaining perfluoro acid was removed with a short silica gel filter column, eluting with *c*-hexane/ethyl acetate (3:1) to yield the Ugi product as a highly viscous yellow oil (53.9 mg, 70.3 µmol, 18.6%).

R_f = 0.45 in *c*-hexane/ethyl acetate (6:1). Visualized *via* fluorescent quench and Seebach staining solution.

IR (ATR): ν [cm^{-1}] = 3320.9 (br, ν(N-H)), 2959.5 (m, ν(C-H)), 2932.7 (w, ν(C-H)), 2861.1 (w, ν(C-H)), 1794.9 (w, ν(C=O)), 1665.5 (s), 1605.3 (w), 1511.7 (m), 1466.2 (s), 1414.5 (m), 1298.9 (m), 1236.4 (vs), 1205.4 (vs), 1147.3 (vs), 1037.6 (s), 936.0 (w), 829.4 (m), 722.4 (m), 703.9 (m), 659.9 (m), 559.0 (m), 528.0 (m).

^{1}H NMR (400 MHz, $CDCl_3$): δ [ppm] = 8.25 (s, 1 H, NH^5), 7.53 – 7.27 (m, 2 H, $CH_{Ar}^{20,24}$), 7.13 – 6.68 (m, 2 H, $CH_{Ar}^{21,23}$), 4.69 (t, J = 7.5 Hz, 1 H, CH^2), 3.79 (s, 3 H, OCH_3^{26}), 3.63 – 3.28 (m, 2 H, CH_2^9), 2.42 – 1.68 (m, 2 H, CH_2^8), 1.64 – 1.42 (m, 2 H, CH_2^{19}), 1.39 – 1.19 (m, 10 H, CH_2), 1.02 – 0.77 (m, 6 H, CH_3^{28+33}).

^{13}C NMR (126 MHz, $CDCl_3$): δ [ppm] = 167.7 (s, CONR^4), 160.3 (s, CONR^{18}), 156.8 (s, C_{Ar}^{22}), 130.7 (s, C_{Ar}^6), 121.8 (s, $CH_{Ar}^{20,24}$), 114.3 (s, $CH_{Ar}^{21,23}$), 62.0 (s, CH^2), 55.6 (s, OCH_3^{26}), 45.6 (s, CH_2^9), 31.7 (s, CH_2), 29.1 (s, CH_2), 27.8 (s, CH_2^8), 26.1 (s, CH_2), 22.6 (s, CH_2), 20.1 (s, CH_2), 14.1(s, $CH_3^{33\ or\ 28}$), 13.6 (s, $CH_3^{33\ or\ 28}$).

^{19}F NMR (376 MHz, $CDCl_3$): δ [ppm] = -85.08 (t, J = 9.9 Hz, 3 F, CF_3^{10}), -112.11 – -113.63 (m, CF_2^{17a}), AB-signal (δ_A = -115.34, δ_B = -115.68, J_{AB} = 331.3 Hz, A and B are split into t, J = 12.8 Hz, CF_2^{17b}), -124.74 (s, CF_2), -126.11 (s, CF_2), -127.03 (s, CF_2), -130.42 (s, CF_2^{11}). Total integral of CF_2 region normalized with respect to the CF_3^{10} group = 14.

FAB – MS [*m/z*] (relative intensity): 766.3 (50%) [M]$^+$, 617.2 (85%) [Fragment A + H]$^+$.

HRMS – FAB [*m/z*]: [M]$^+$ calculated for $^{12}C_{28}{}^1H_{31}{}^{16}O_3{}^{14}N_2{}^{19}F_{17}$, 766.2058; found, 766.2058; Δ = 0.04 mmu.

Chemical Formula: $C_{20}H_{23}F_{17}NO^{\bullet}$

Exact Mass: 616,15082

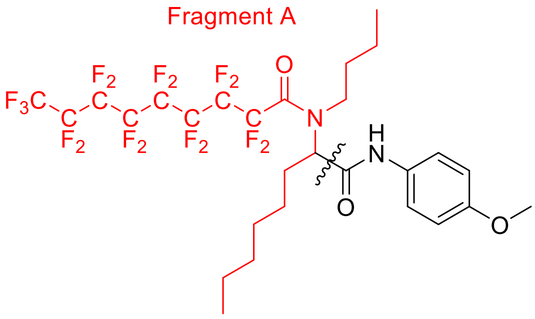

Supplementary Figure 191 | Proposed fragments observed in FAB-MS.

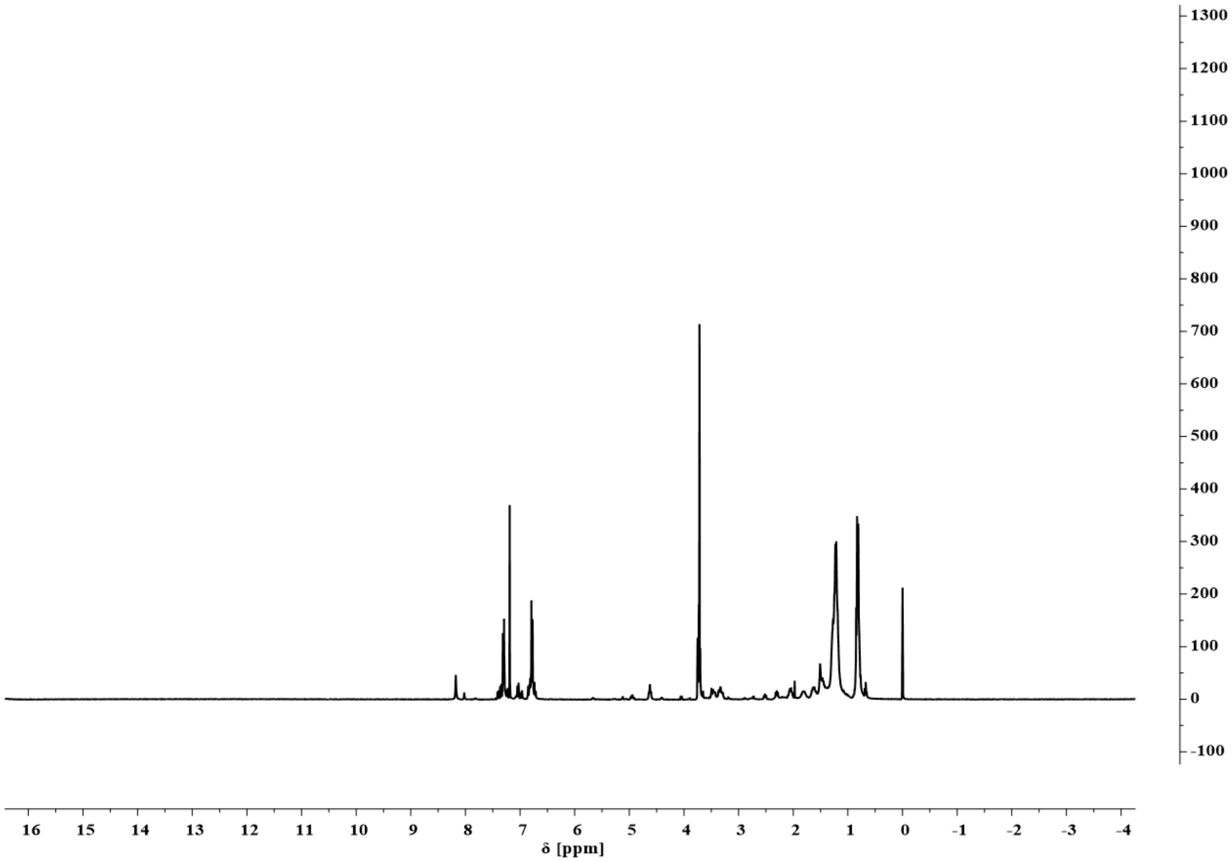

Supplementary Figure 192 | ^{1}H NMR experiment of the title compound recorded in $CDCl_3$.

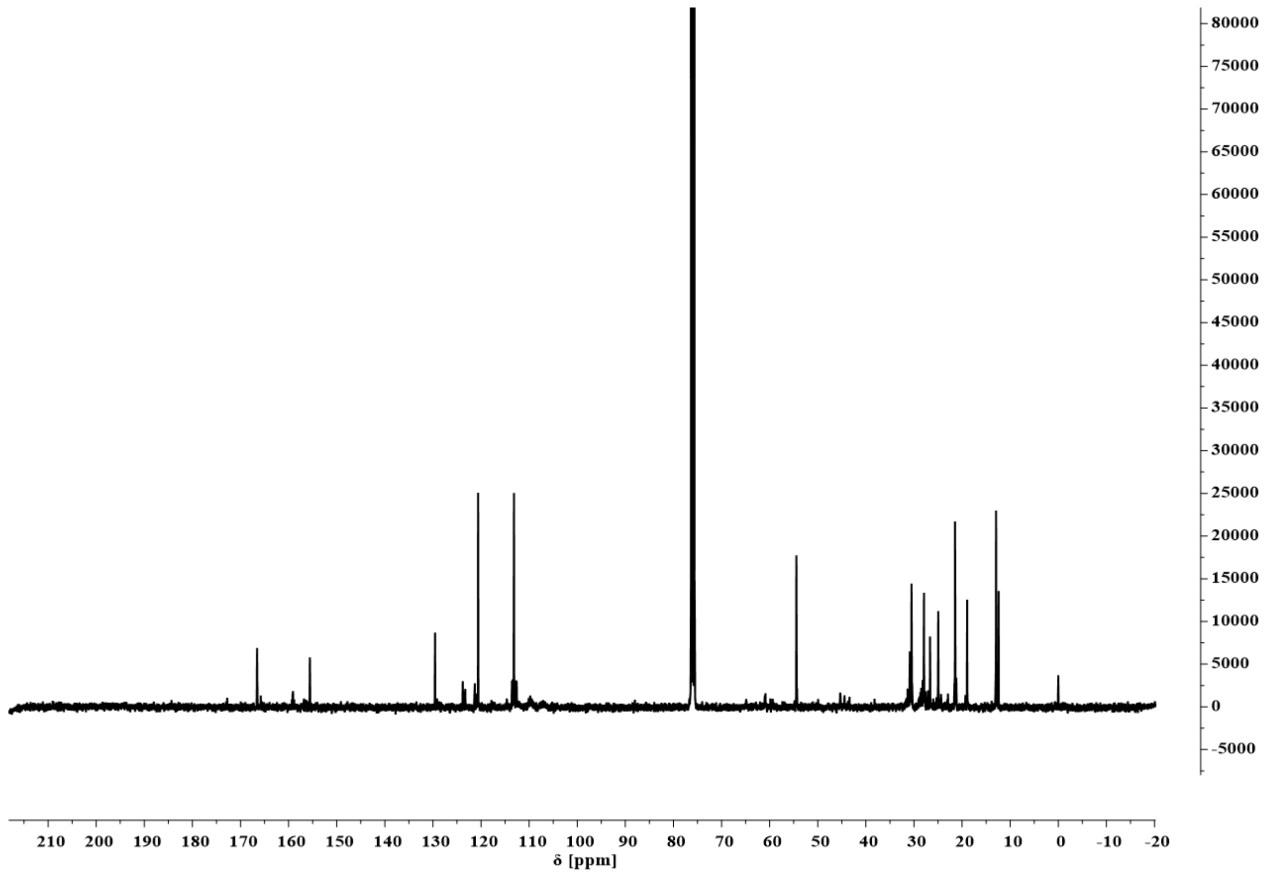

Supplementary Figure 193 | ^{13}C NMR experiment of the title compound recorded in $CDCl_3$.

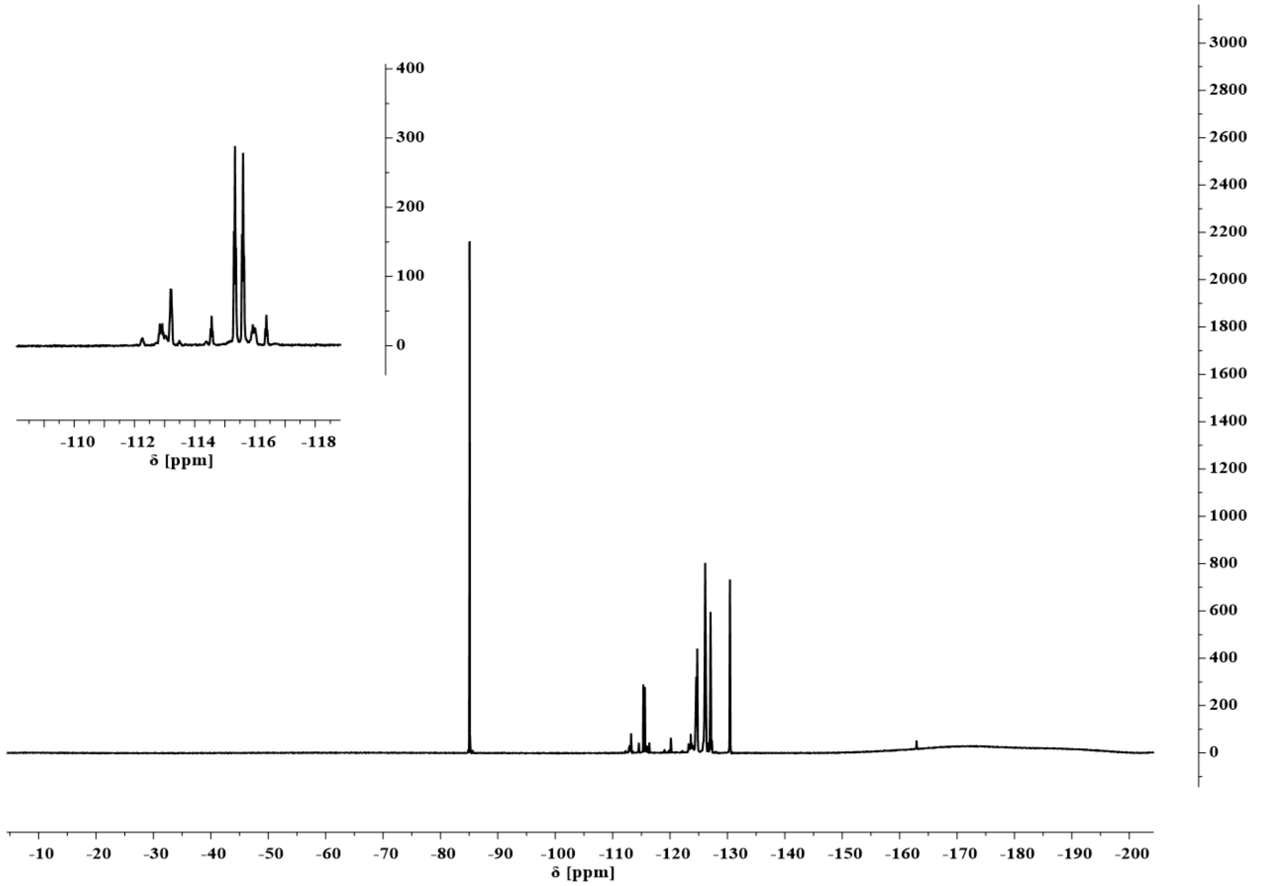

Supplementary Figure 194 | ^{19}C NMR experiment of the title compound recorded in $CDCl_3$.

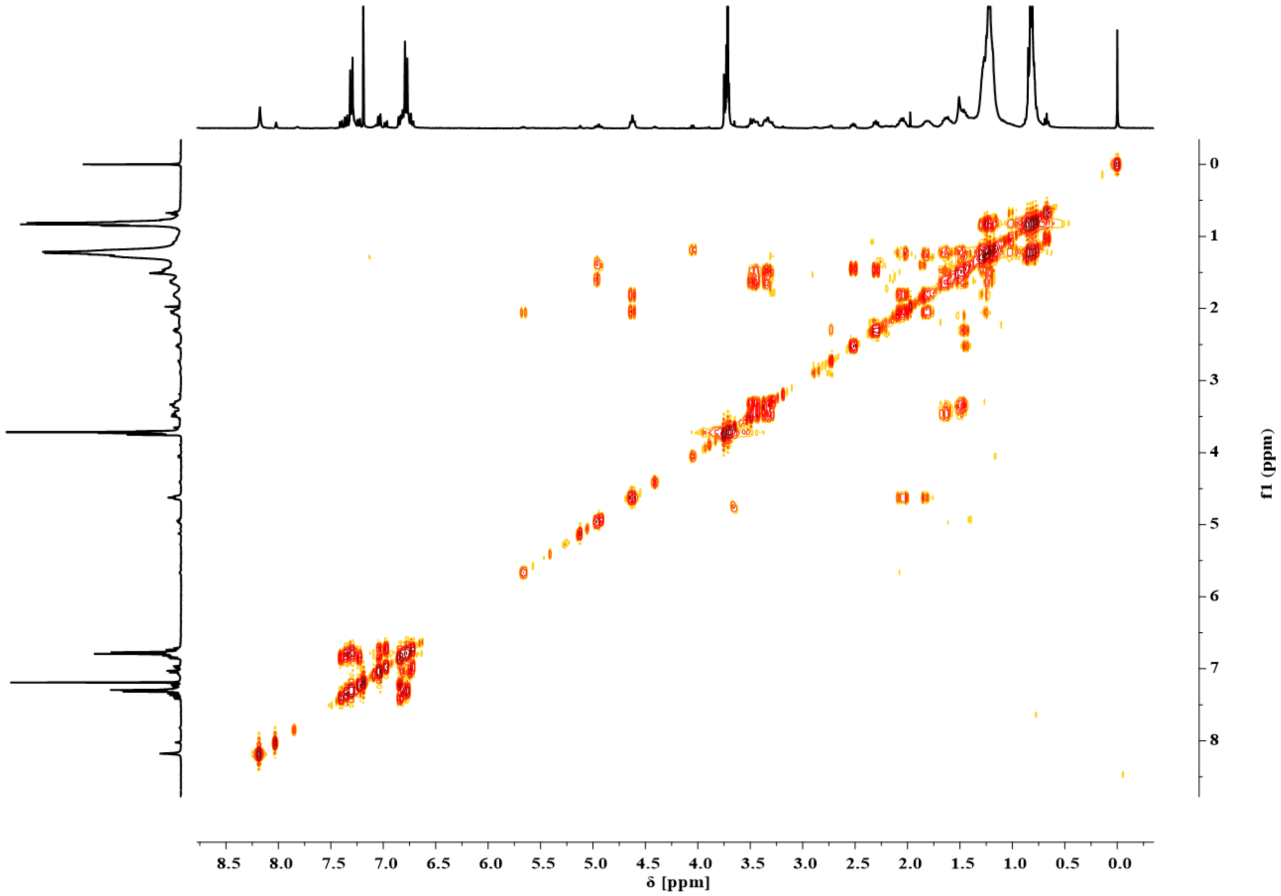

Supplementary Figure 195 | COSY experiment of the title compound recorded in $CDCl_3$

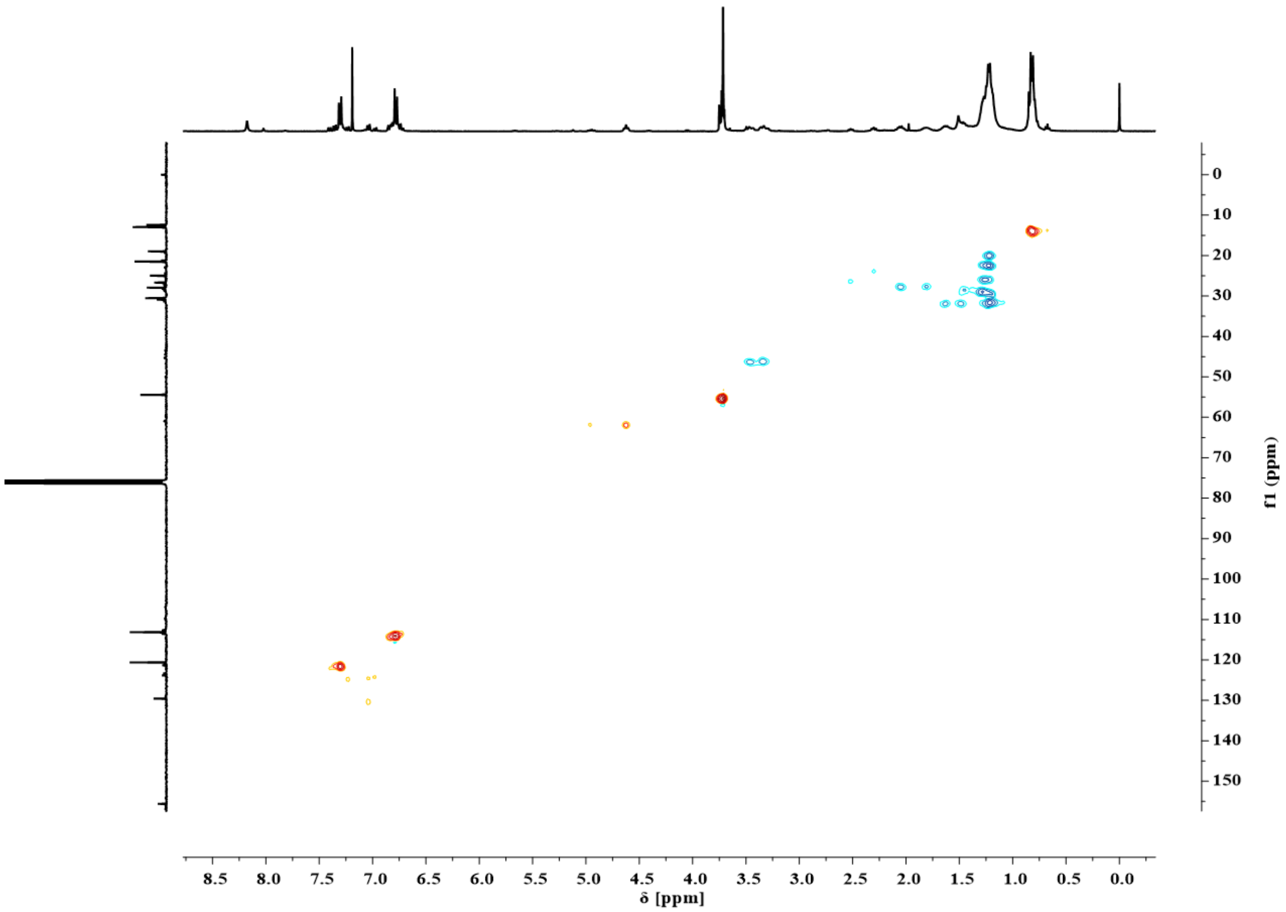

Supplementary Figure 196 | Multiplicity-edited HSQC experiment of the title compound recorded in $CDCl_3$

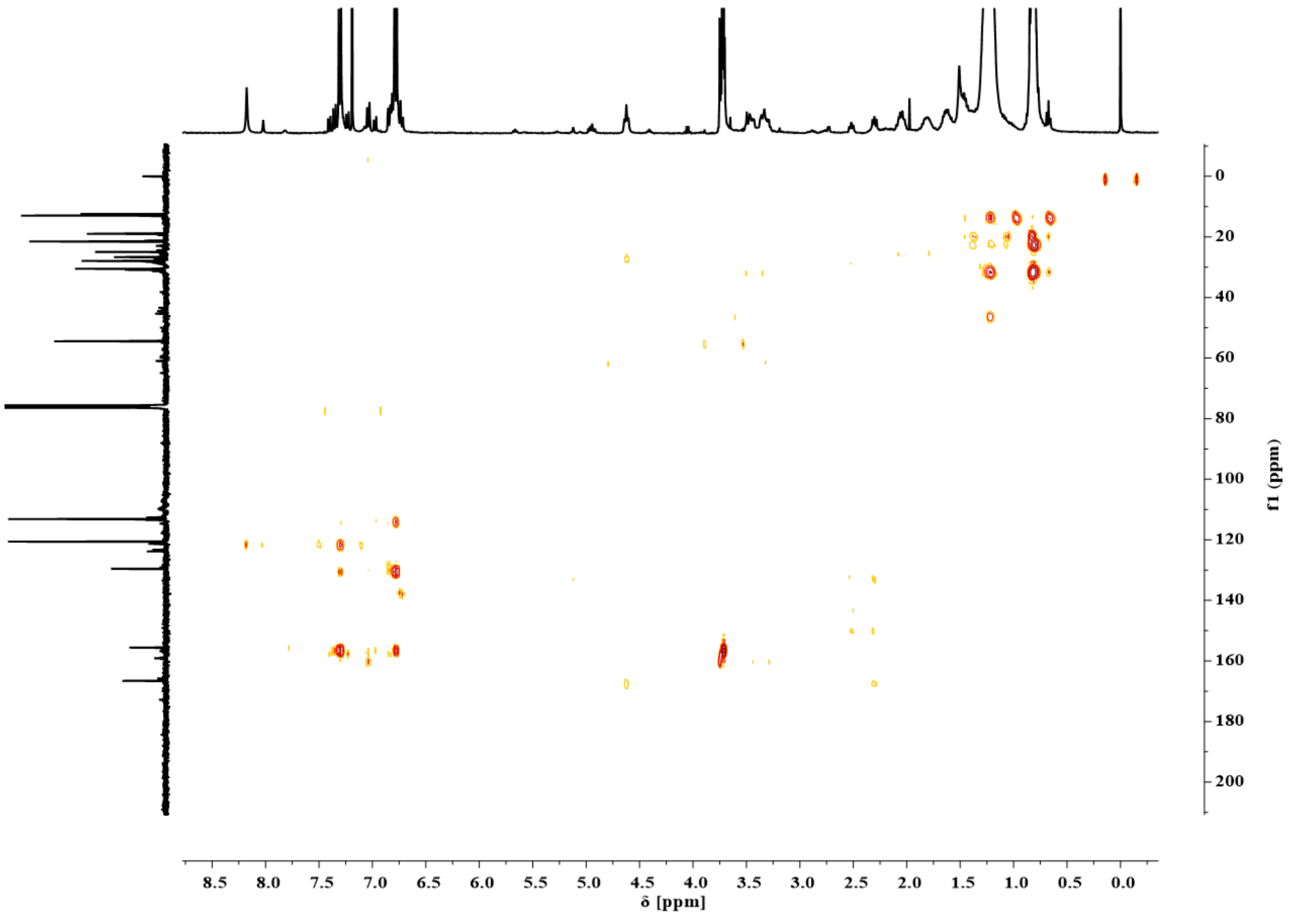

Supplementary Figure 197 | HMBC experiment of the title compound recorded in $CDCl_3$

Ugi reaction of perfluorononanoic acid, benzaldehyde, cyclohexylisocyanide and pentylamine

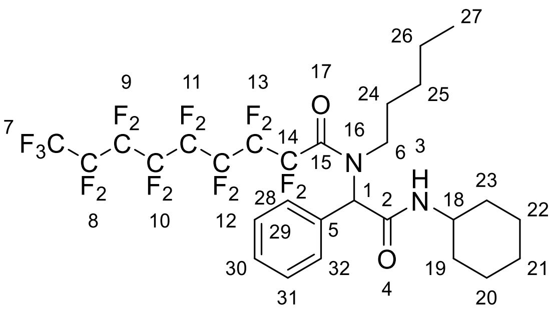

In a 25 mL round bottom flask benzaldehyde (56.3 µL, 49.4 mg, 453 µmol, 1.20 eq.) was dissolved in 1.5 mL methanol, subsequently pentylamine (56.6 µL, 42.7 mg, 490 µmol, 1.30 eq.) was added and the resulting mixture was stirred for 60 min over sodium sulfate. Afterwards, the mixture was filtrated and the solid was washed with 10 mL methanol three times. Subsequently, the filtrate was concentrated under reduced pressure. Perfluorononanoic acid (175 mg, 377 µmol, 1.00 eq.) dissolved in 1 mL methanol was added to the imine at room temperature and the resulting mixture was stirred for 2 min. Subsequently, cyclohexylisocyanide (56.3 µL, 59.4 mg, 453 µmol, 1.20 eq.) was added to the stirring mixture. The reaction was stirred for 4 d at room temperature. The crude reaction mixture was dried under reduced pressure and purified *via* column chromatography employing Fluoro*Flash*®

silica gel. The fluorous fraction was tested for purity *via* TLC and concentrated under reduced pressure. The remaining perfluoro acid was removed with a short silica gel filter column, eluting with *c*-hexane/ethyl acetate (3:1) to yield the Ugi product as a colorless solid (106 mg, 140 µmol, 42.3%).

R_f = 0.47 in *c*-hexane/ethyl acetate (6:1). Visualized *via* fluorescent quench and Seebach staining solution.

IR (ATR): ν [cm^{-1}] = 3306.5 (br, ν(N-H)), 2922.9 (m, ν(C-H)), 2851.1 (w), 2186.6 (vw), 2044.9 (vw), 1971.1 (vw), 1672.8 (s, ν(C=O)), 1654.2 (s, ν(C=O)), 1556.4 (m), 1451.0 (w), 1428.8 (m), 1369.9 (m), 1234.2 (s), 1193.9 (s), 1145.5 (s), 1119.6 (s), 1062.2 (m), 975.9 (m), 923.8 (m), 859.2 (w), 762.6 (w), 709.5 (s), 683.4 (m), 666.9 (m), 643.2 (m), 559.2 (m), 516.4 (m), 463.7 (w), 437.2 (w).

^{1}H NMR (400 MHz, CD_3OD): δ [ppm] = 7.54 – 7.22 (m, 5 H, $CH_{Ar}^{28\text{-}32}$), 5.87 (d, J = 29.9 Hz, 1 H, CH^{1}), 3.84 – 3.52 (m, 1 H, CH_2^{6a}), 3.41 – 3.35 (m, 1 H, CH^{18}), 3.24 – 3.13 (m, 1 H, CH_2^{6b}), 1.90 – 1.52 (m, 6 H, CH_2), 1.44 – 1.24 (m, 4 H, CH_2), 1.21 – 1.04 (m, 4 H, CH_2), 1.04 – 0.68 (m, 2 H, CH_2^{26}), 0.66 – 0.53 (m, 3 H, CH_3^{27}).

^{13}C NMR (101 MHz, CD_3OD): δ [ppm] = 169.9 (s, $CONR^{2}$), 158.8 (s, $CONR^{15}$), 135.7 (s, C_{Ar}^{5}), 131.7 (s, CH_{Ar}), 130.9 (s, CH_{Ar}), 130.1 (s, CH_{Ar}), 65.5 (s, CH^{1a}), 64.1 (s, CH^{1b}), 50.0 (s, CH^{18}), 47.3 (s, CH_2^{6}), 33.5 (s, CH_2), 30.3 (s, CH_2), 26.6 (s, CH_2), 26.0 (s, CH_2), 21.0 (s, CH_2), 20.7 (s, CH_2^{26}), 13.7 (s, CH_3^{27}).

^{19}F NMR (376 MHz, CD_3OD): δ [ppm] = -88.27 (t, J = 9.4 Hz, 3 F, CF_3^{7}), AB-signal (δ_A = -116.01, δ_B = -117.30, J_{AB} = 299.3 Hz, A and B are split into t, J = 12.8 Hz, CF_2^{14a}), AB-signal (δ_A = -117.97, δ_B = -119.28, J_{AB} = 291.8 Hz, A and B are split into t, J = 11.3 Hz, CF_2^{14b}), -126.38 (s, CF_2), -127.08 (s, CF_2), -128.67 (s, CF_2), -129.65 (s, CF_2), -133.20 (s, CF_2^{8}). Total integral of CF_2 region normalized with respect to the CF_3^{7} group = 14.

FAB – MS [*m/z*] (relative intensity): 749.2 $[M + H]^+$ (80%), 552.0 (92%) [Fragment A + H]$^+$, 217.1 (68%) [Fragment B + H]$^+$.

HRMS – FAB [*m/z*]: $[M + H]^+$ calculated for $^{12}C_{28}{}^{1}H_{29}{}^{16}O_2{}^{14}N_2{}^{19}F_{17}$, 749.2030; found, 749.2032; Δ = 0.17 mmu.

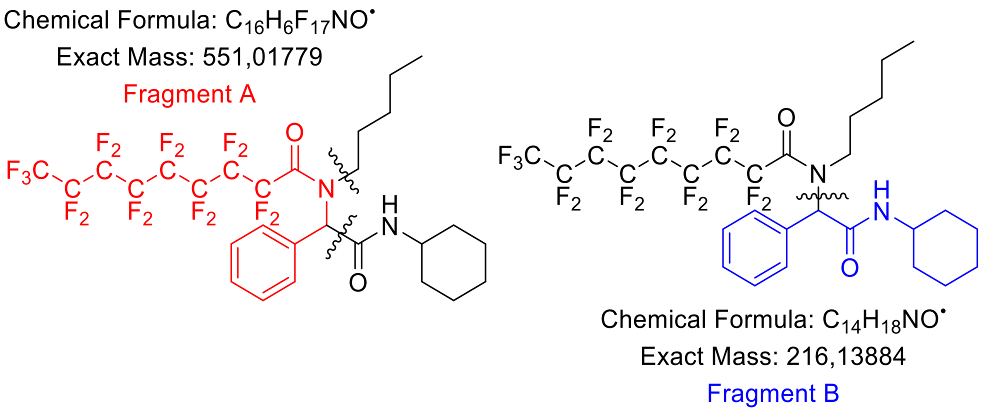

Supplementary Figure 198 | Proposed fragments observed in FAB-MS.

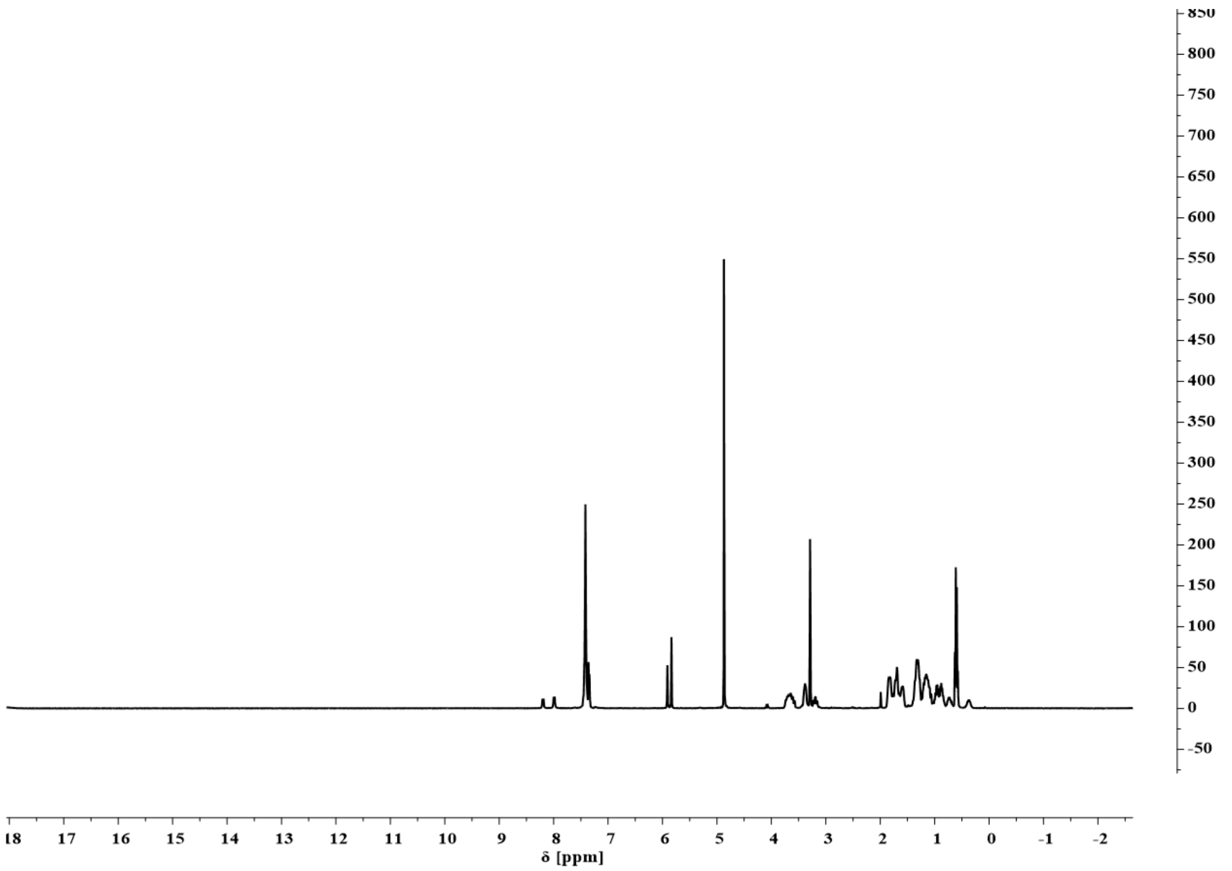

Supplementary Figure 199 | ^{1}H NMR experiment of the title compound recorded in CD_3OD.

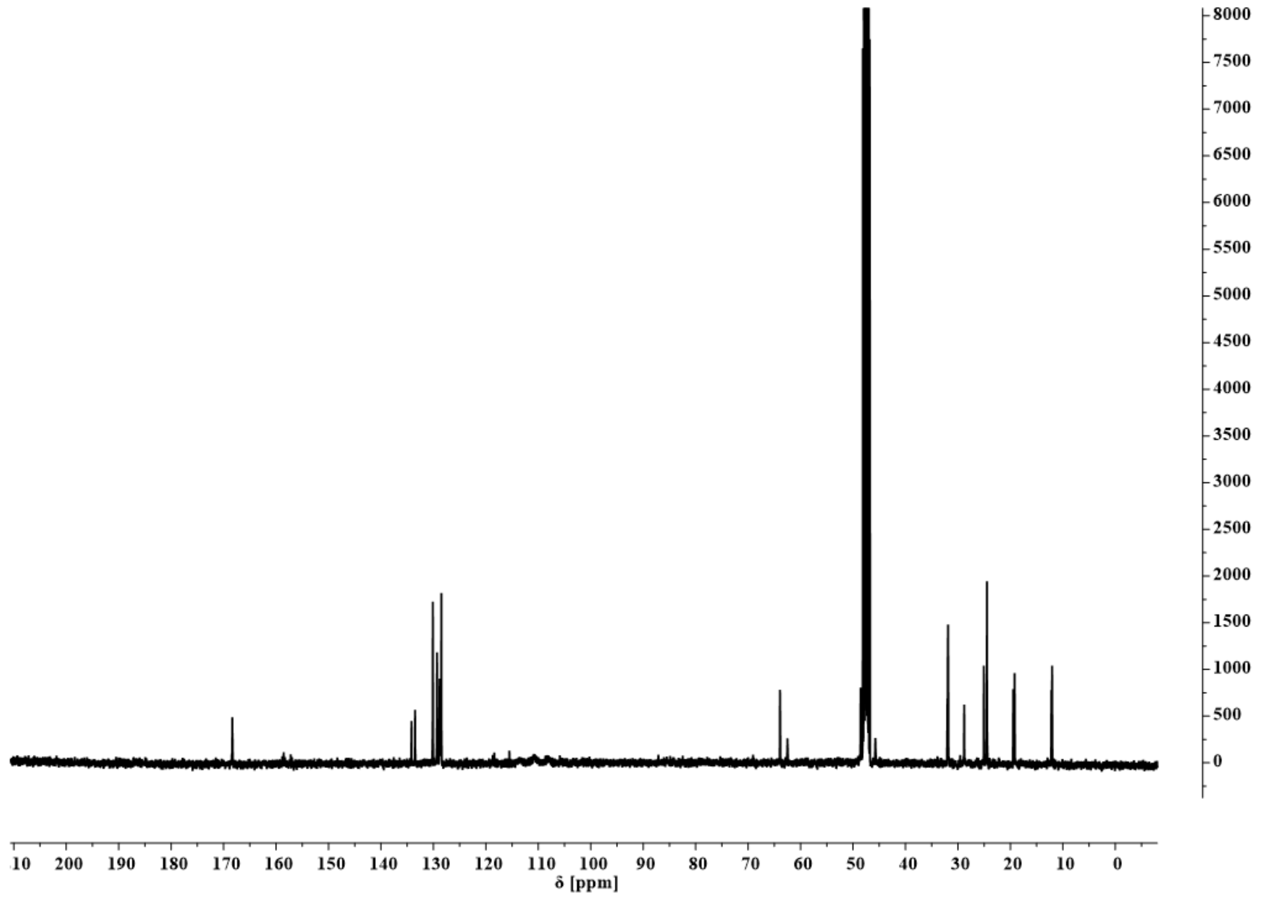

Supplementary Figure 200 | ^{13}C NMR experiment of the title compound recorded in CD_3OD.

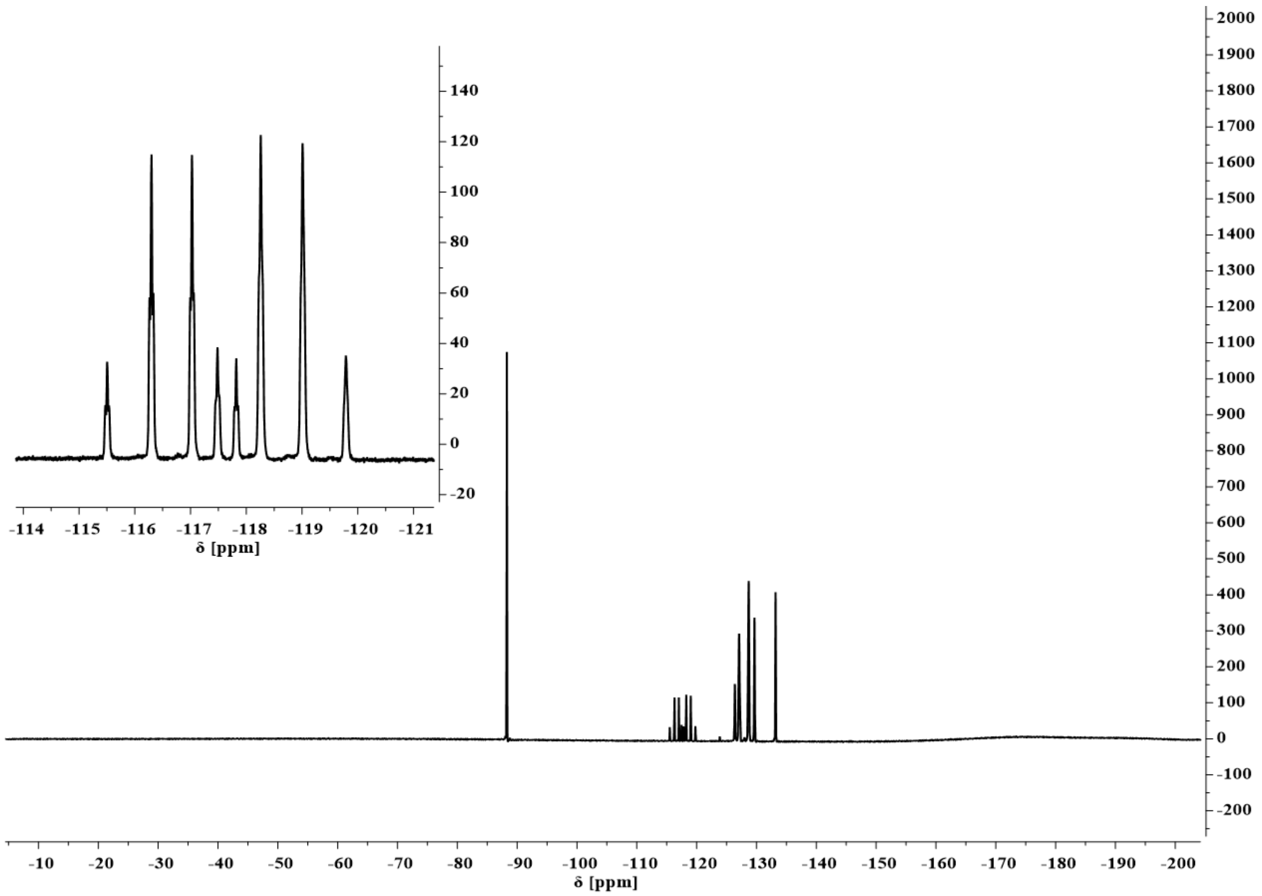

Supplementary Figure 201 | ^{19}F NMR experiment of the title compound recorded in CD_3OD.

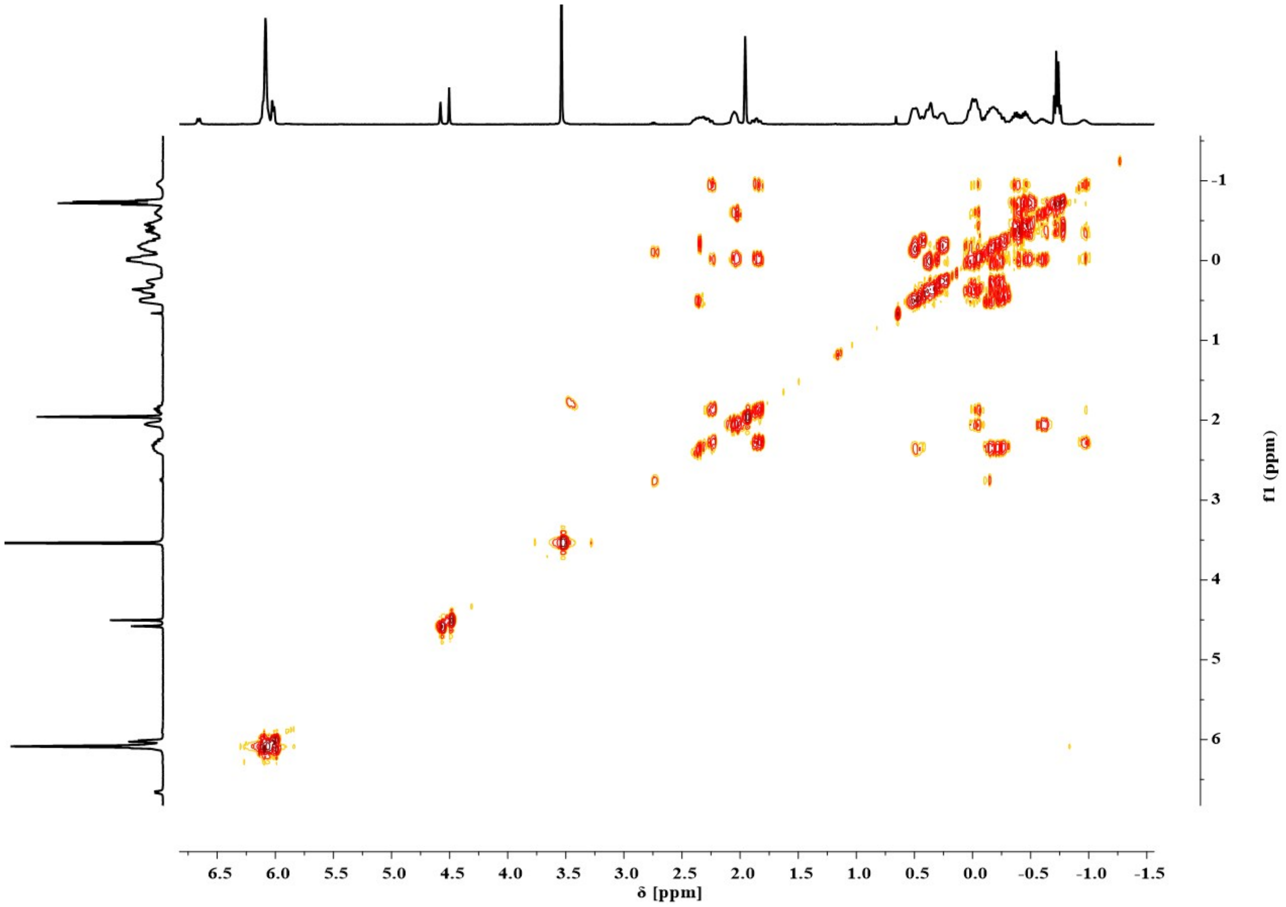

Supplementary Figure 202 | COSY experiment of the title compound recorded in CD_3OD.

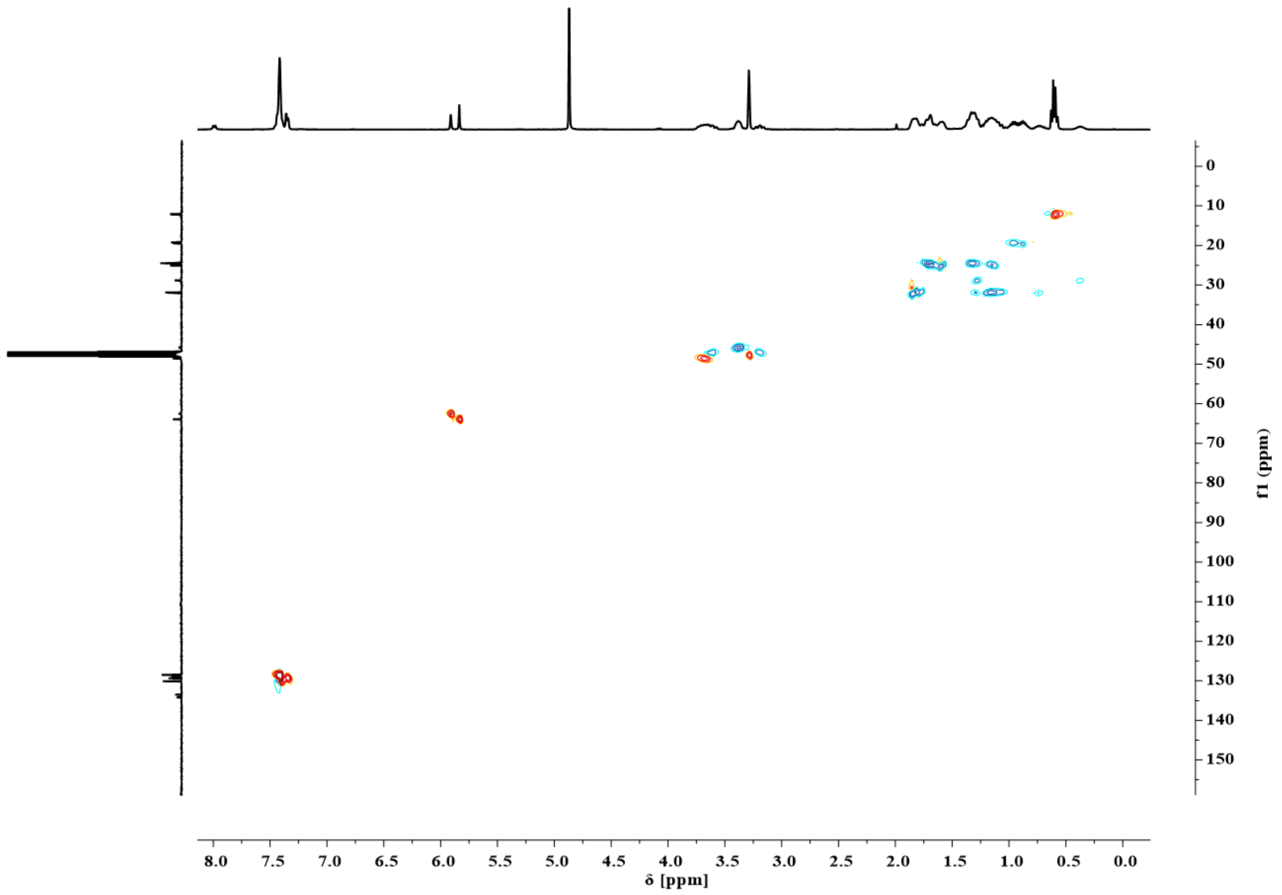

Supplementary Figure 203 | Multiplicity-edited HSQC experiment of the title compound recorded in CD_3OD.

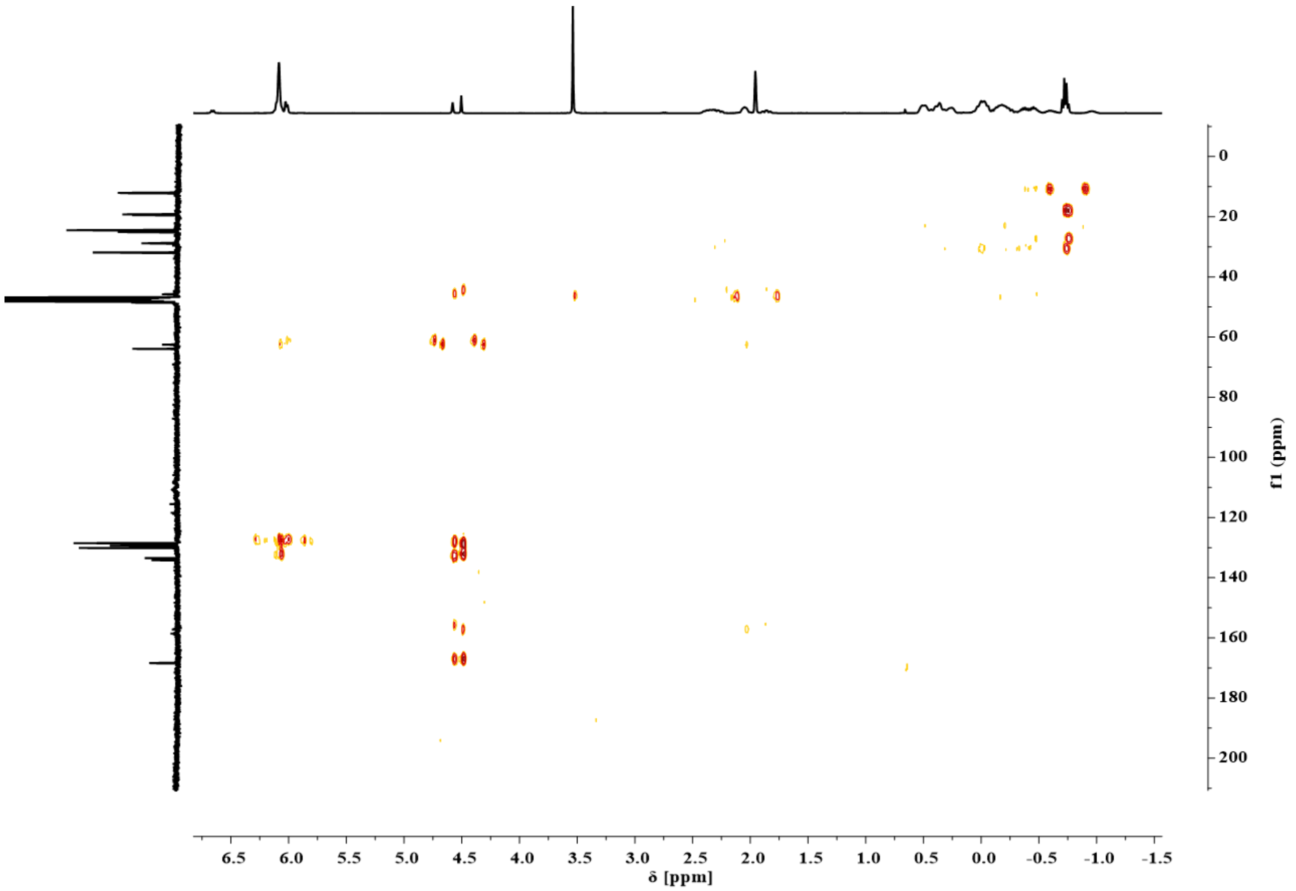

Supplementary Figure 204 | HMBC experiment of the title compound recorded in CD_3OD.

Ugi reaction of perfluorononanoic acid, cyclamen aldehyde, *tert*-butylisocyanide and 2-pentylamine

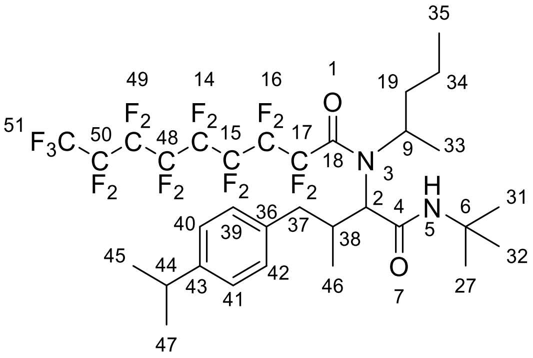

In a 25 mL round bottom flask cylcamenaldehyde (906 µL, 861 mg, 4.53 mmol, 3.00 eq.) and 2-pentylamine (493 µL, 394 mg, 4.53 mmol, 3.00 eq.) were stirred for 60 min over sodium sulfate. The mixture was diluted with 0.5 mL methanol and perfluorononaic acid (700 mg, 1.51 mmol, 1.00 eq.) was added at room temperature. Subsequently, *tert*-butylisocyanide (512 µL, 376 mg, 4.53 mmol, 3.00 eq.) was added to the stirring mixture. The reaction was stirred for 4 d at room temperature. The crude reaction mixture was dried under reduced pressure and purified *via* column chromatography employing Fluoro*Flash*® silica gel and eluting with 8 mL methanol/water (8:2) to elute the organic fraction, subsequently the fluorous fraction was eluted with pure methanol. The remaining perfluouro acid was removed with a short silica gel filter column, eluting with *c*-hexane/ethyl acetate (3:1). After drying under reduced pressure, the fluoro-tagged product (diastereomer mixture) was obtained as a yellow oil (209 mg, 251 µmol, 16.7%).

R_f = 0.75 in *c*-hexane/ethyl acetate (3:1). Visualized *via* fluorescent quench and Seebach staining solution.

IR (ATR): ν [cm^{-1}] = 3305.2 (br, ν(N-H)), 2959.7 (vs, ν(C-H)), 2929.3 (s, ν(C-H)), 2870.8 (m, ν(C-H)), 2711.1 (m), 1725.1 (vs, ν(C=O)), 1674.4 (s, ν(C=O)), 1512.6 (m), 1457.4 (m), 1419.9 (w), 1382.2 (w), 1363.0 (w), 1282.5 (w), 1217.7 (m), 1114.2 (s), 1050.7 (m), 1019.5 (w), 923.8 (w), 879.9 (m), 837.5 (w), 704.4 (w), 548.6 (m).

^{1}H NMR (500 MHz, CD_3OD): δ [ppm] = 7.20 – 6.95 (m, 4 H, CH_{Ar}^{39-42}), 4.87 (s, 1 H), 4.30 (dd, J = 5.0, 2.3 Hz, 1 H, CH^{2}), 3.04 – 2.50 (m, 5 H, CH_2^{19+37} + CH^{9}), 2.31 (ddd, J = 13.4, 9.4, 2.6 Hz, 2 H, CH_2^{34}), 1.94 – 1.80 (m, 2 H, CH^{38} + CH^{44}), 1.42 – 1.12 (m, 18 H, $CH_3^{45,47}$ + $CH_3^{27,31,32}$ + CH_3^{33}), 1.03 – 0.82 (m, 6 H, CH_3^{35+46}).

^{13}C NMR (126 MHz, CD_3OD): δ [ppm] = 147.3 (s, $CONR^{4}$), 139.3 (s, $CONR^{18}$), 139.2 (s, C_{Ar}), 130.2 (s, C_{Ar}), 127.4 (s, CH_{Ar}), 127.1 (s, CH_{Ar}), 102.1 (s, CH^{2}), 42.7 (s, $CH^{38\ or\ 44}$), 42.4 (s, $CH^{38\ or\ 44}$), 38.9 (s, CH_2), 38.7 (s, CH_2), 35.5 (s, CH_2), 34.9 (s, CH^{9}), 24.6 (s, CH_3), 24.5 (s, CH_3), 14.2 (s, CH_3^{35or46}), 14.1 (s, CH_3^{35or46}).

^{19}F NMR (376 MHz, CD_3OD): δ [ppm] = -86.41 (t, J = 10.4 Hz, 3 F, CF^{51}), -113.47 – -116.61 (m, CF_2^{17}), -124.05 – -125.24 (m, CF_2), -126.48 (s, CF_2), -126.67 – -127.14 (m, CF_2), -127.72 (d, J = 17.1 Hz, CF_2), -127.84 (s, CF_2), -130.95 – -131.61 (m, CF_2^{50}). Total integral of CF_2 region normalized with respect to the CF_3^{51} group = 14.

ESI – MS [m/z]: $[M + Na]^+$ calculated for $^{12}C_{32}{}^{1}H_{39}{}^{16}O_2{}^{14}N_2{}^{19}F_{17}Na_1$, 829.2638; found, 829.2636; Δ = 0.19 mmu.

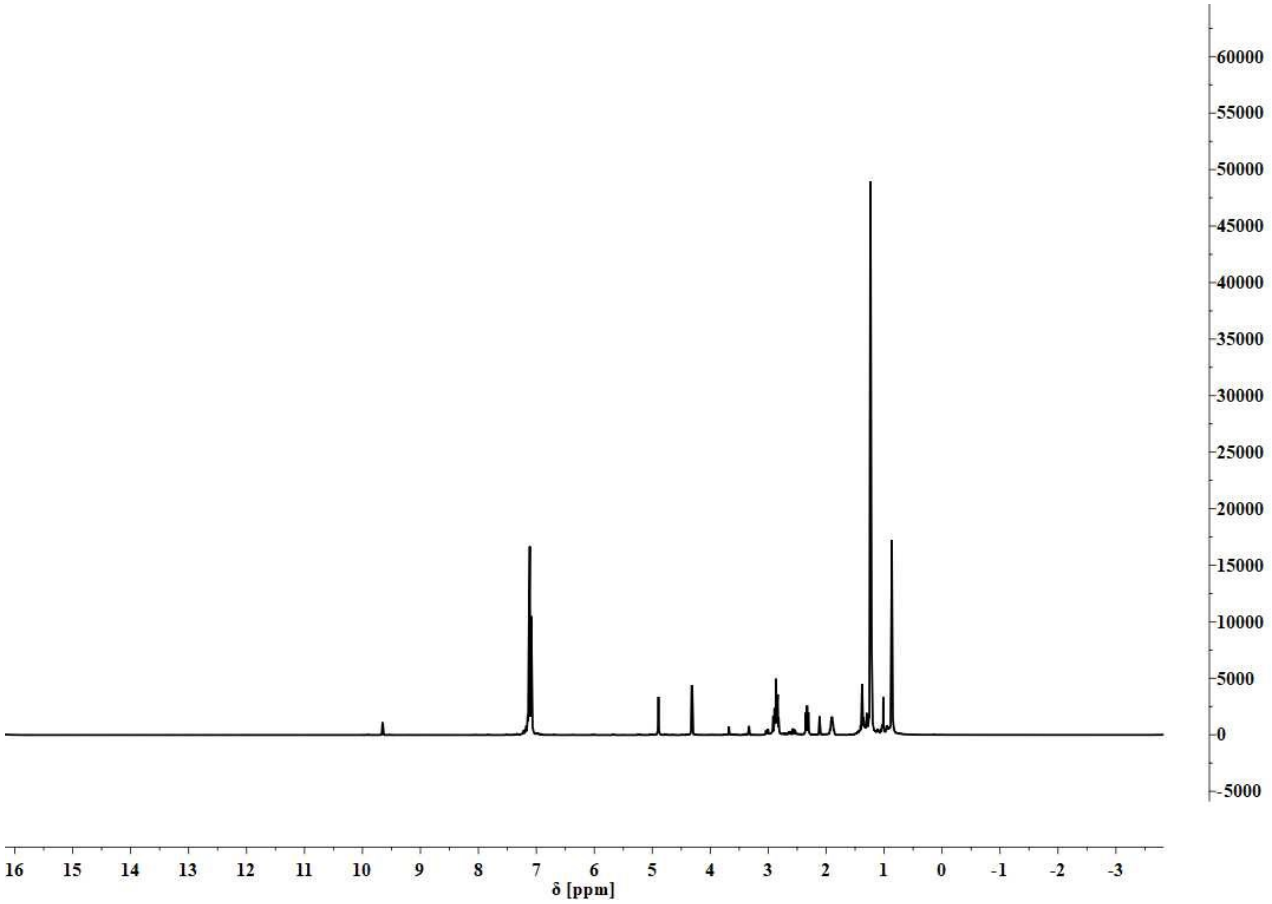

Supplementary Figure 205 | ^{1}H NMR experiment of the title compound recorded in CD_3OD.

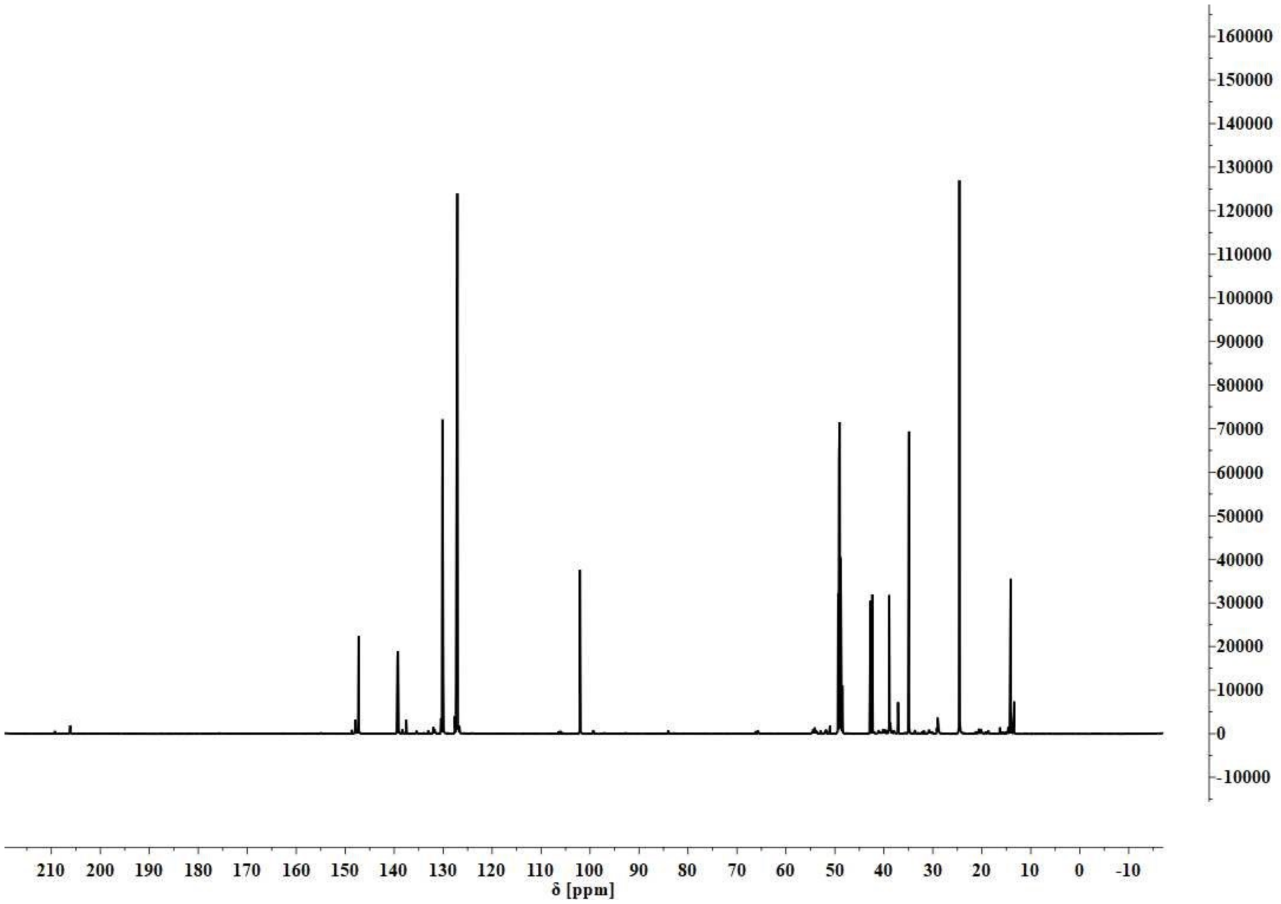

Supplementary Figure 206 | ^{13}C NMR experiment of the title compound recorded in CD_3OD.

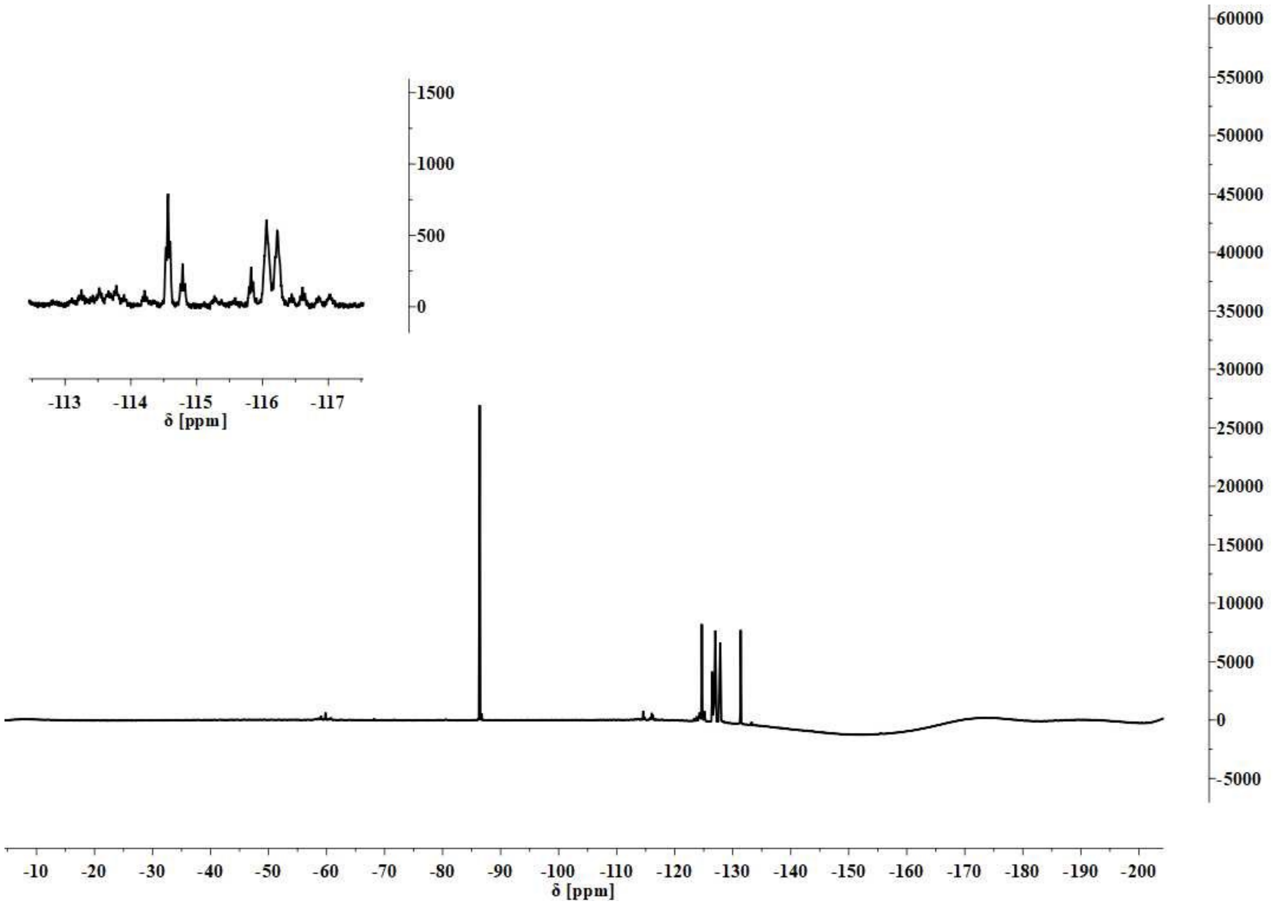

Supplementary Figure 207 | ^{19}F NMR experiment of the title compound recorded in CD_3OD.

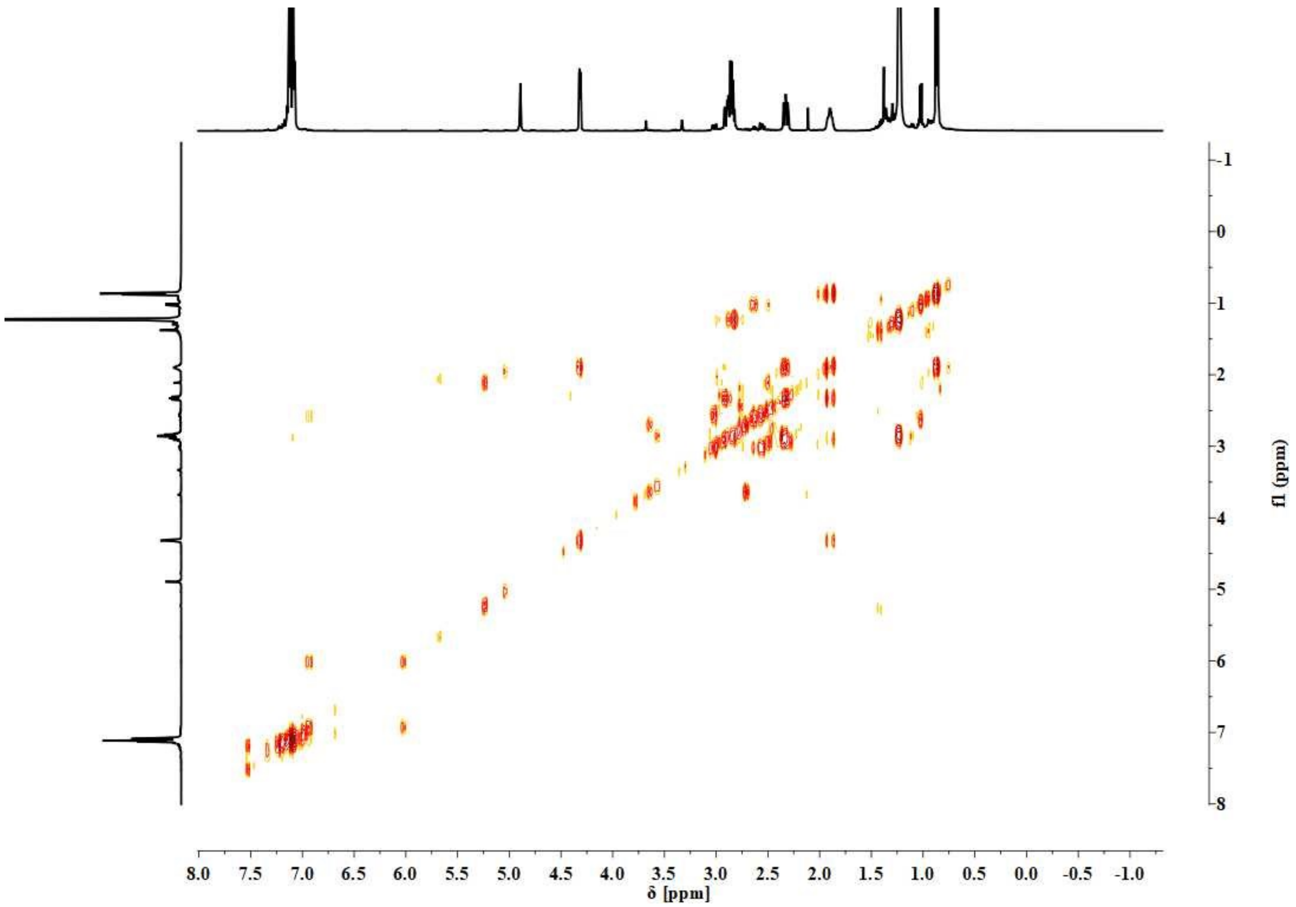

Supplementary Figure 208 | COSY experiment of the title compound recorded in CD_3OD.

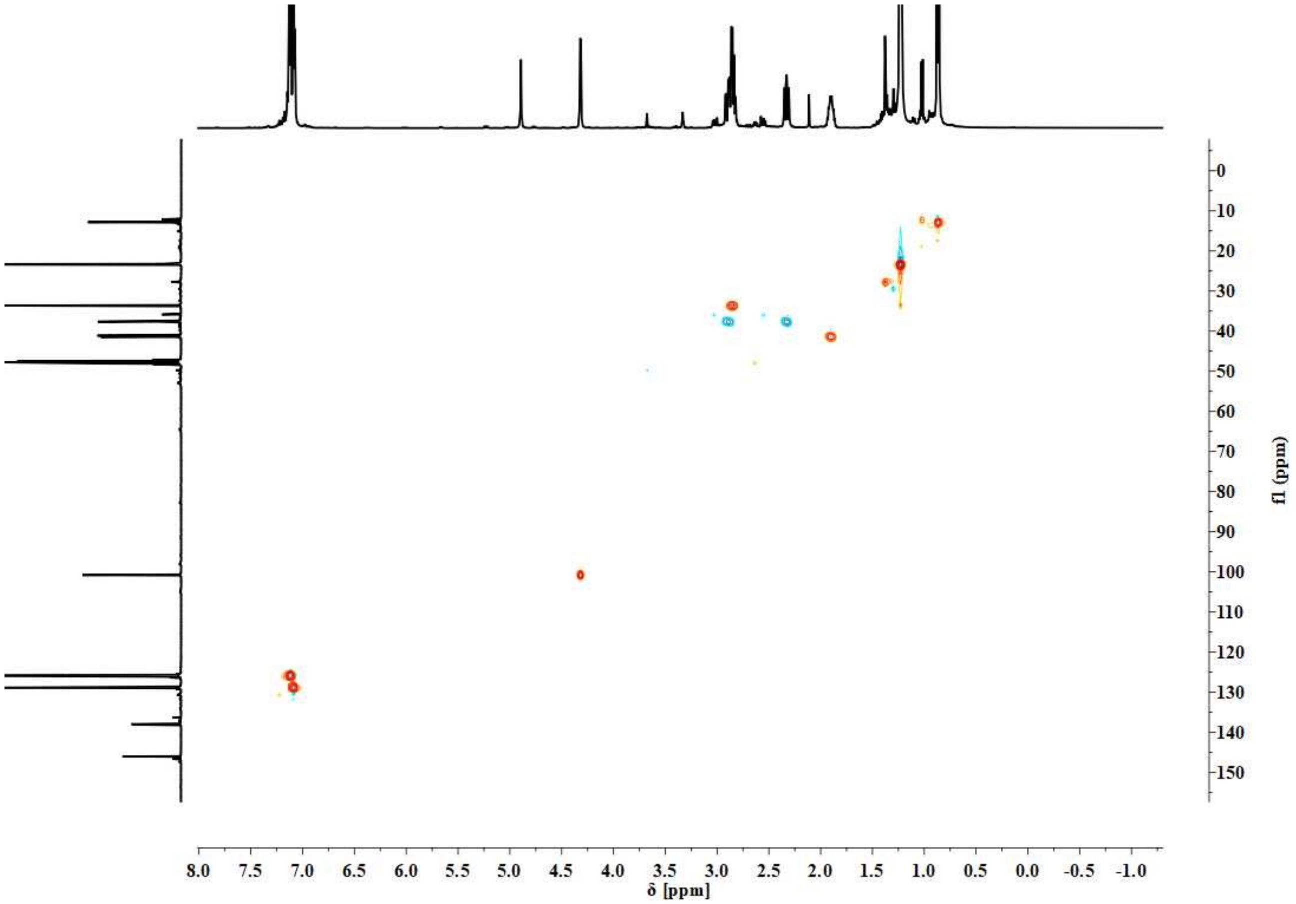

Supplementary Figure 209 | Multiplicity-edited HSQC experiment of the title compound recorded in CD_3OD.

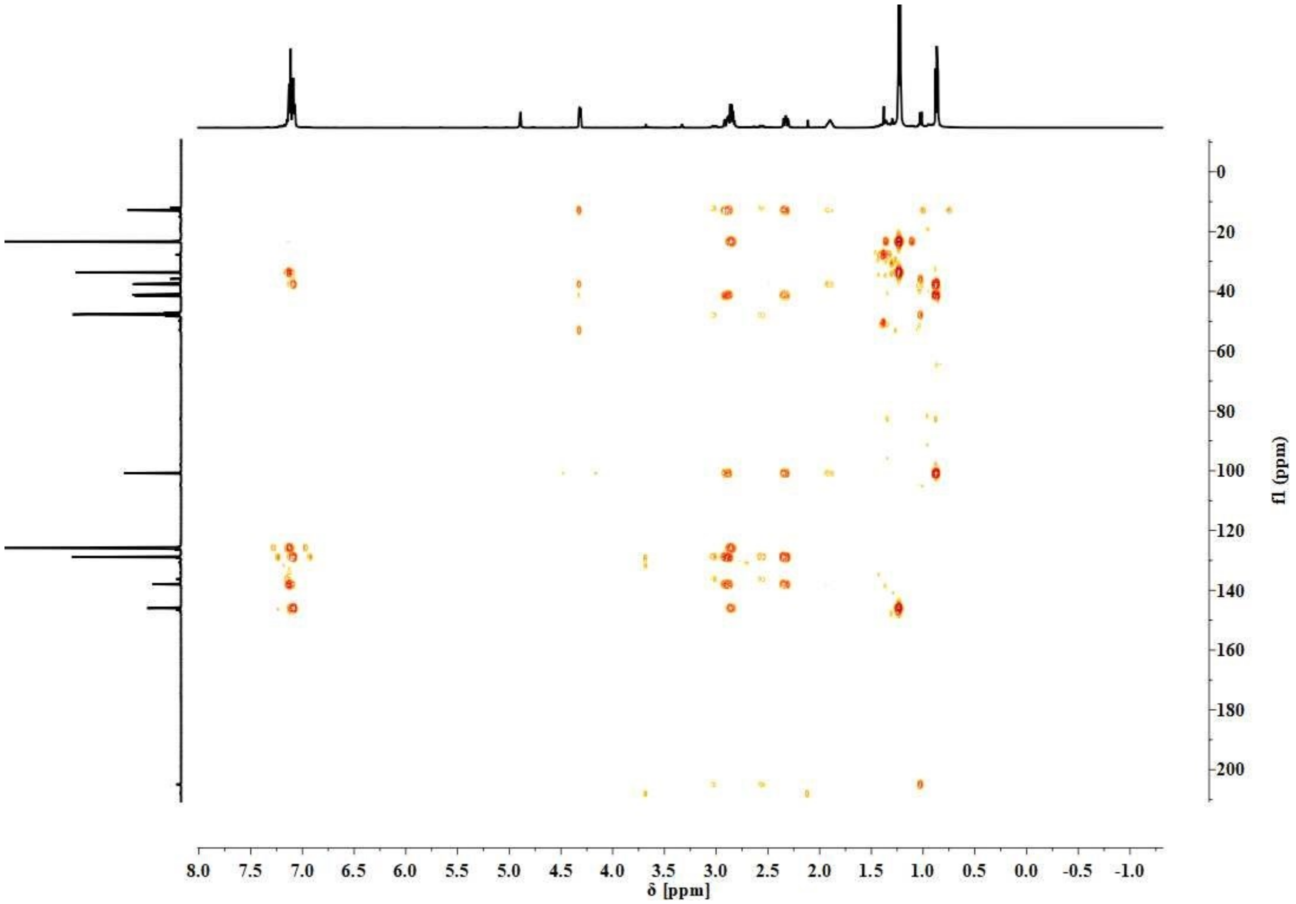

Supplementary Figure 210 | HMBC experiment of the title compound recorded in CD_3OD.

Influence of stereochemistry

During the Ugi reaction a new chiral center is formed, which is not controlled in the present protocol. However, if chiral precursor components are utilized, diastereomeric product mixtures will result. In order to study if diastereoisomers have an influence on the separation protocol or the MS/MS fragmentation behavior, a model substrate was synthesized, purified *via* F-SPE and fragmented *via* ESI-MS/MS.

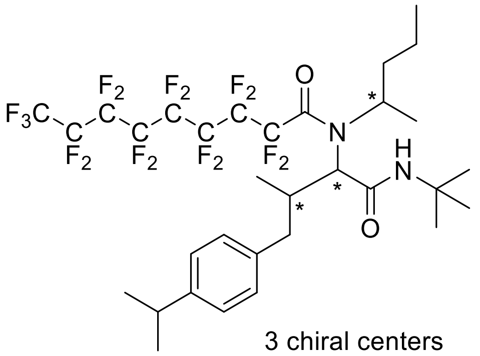

Supplementary Figure 211 | Model substrate for studying the influence of stereochemistry. The structure presented above was synthesized from the racemic precursor components. The reaction product will thus be a mixture of four different diastereoisomers.

The F-SEP purification protocol retains perfluorinated compounds selectively and is thus unaffected by diastereoisomers. This is a great advantage of F-SPE and was utilized in the field of so called fluorous mixture synthesis (FMS).[2] FMS separation was also applied successfully for the synthesis of diastereomer mixtures, wherein F-tagged diastereomers were synthesized stepwise and separated from non-fluorinated contaminations *via* F-SPE.[3] The synthetic procedures for the diastereomeric molecular key mixture presented above is included at the end of the chapter Synthetic procedures (page 154). If a diastreomeric mixture is subjected to a tandem-MS experiment, it can be assumed that the required fragments for the readout will still be observed, since, even if theoretically different fragmentation pathways may occur, the favored fragments presented in the manuscript will still be observed, at least for some of the fragmentation pathways (maybe with a somewhat lower probability, but the required fragments should still be present and detectable). The observed mass of the fragments is independent from the stereochemical information. The fragmentation behavior of diastereoisomers in MS/MS methods and was previously studied, confirming that the fragmentation leads to a different intensity distribution pattern but all relevant fragments were observed.[4,5] In the Supplementary Figure 11 the fragmentation of the diastereomeric product mixture presented above is illustrated, indicating that diastereomeric mixtures of molecular keys can be unambiguously read out similar to diastereomeric pure molecular keys.

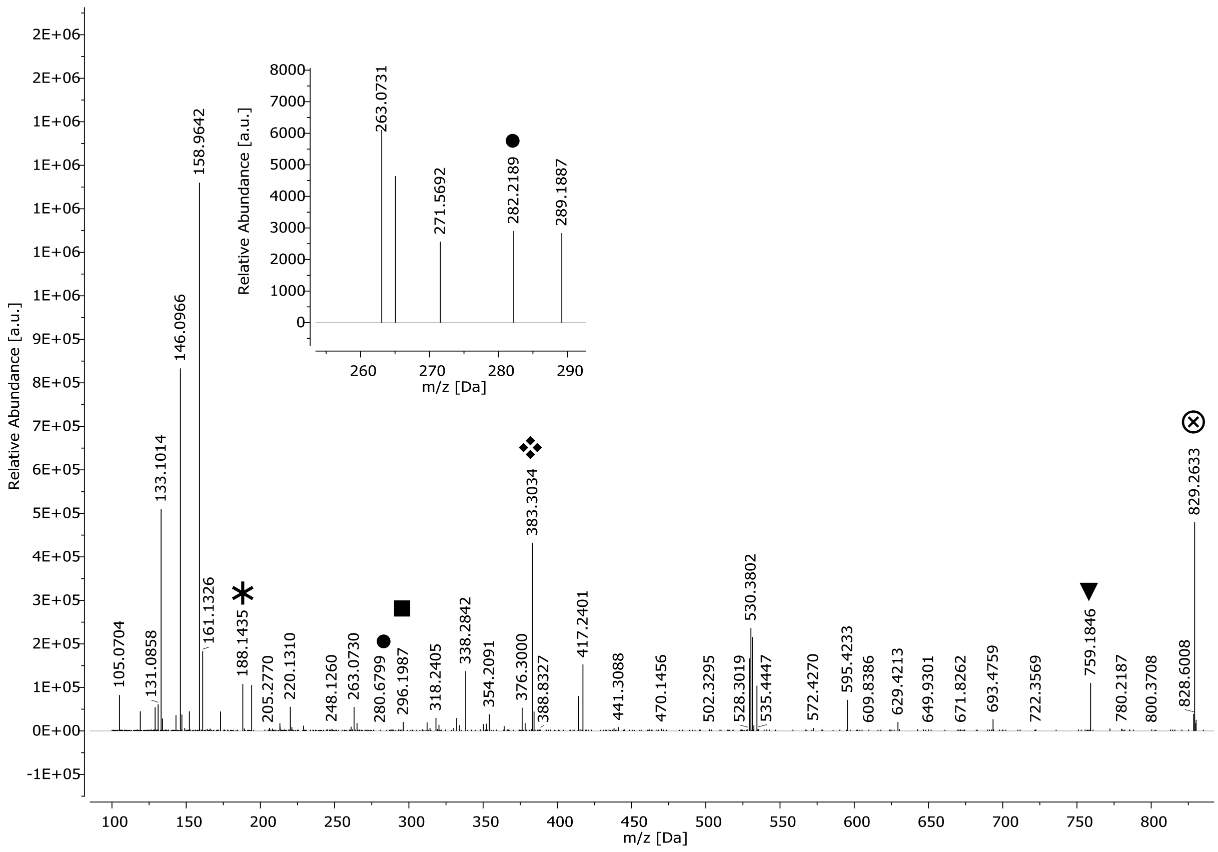

Entry	Label	Resolution	*m/z*(exp)	*m/z*(theo)	Δ*m/z*	Formula	Structure
1	⊗	73800	829.2633	829.2638	0.0005	$C_{32}H_{39}O_2N_2F_{17}Na_1$	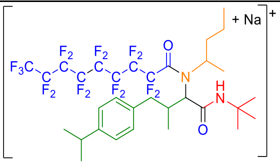
2	▼	71000	759.1846	759.1850	0.0004	$C_{27}H_{29}O_2N_2F_{17}Na_1$	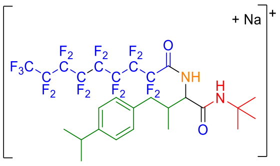
3	❖	106000	383.3034	383.3033	0.0001	$C_{23}H_{40}O_1N_2Na_1$	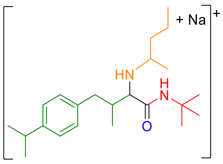
4	■	122000	296.1987	296.1985	0.0002	$C_{18}H_{27}O_1N_1Na_1$	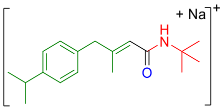
5	●	124000	282.2189	282.2192	0.0003	$C_{18}H_{29}N_1Na_1$	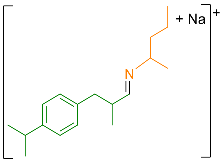
6	✱	165000	188.1435	188.1434	0.0001	$C_{13}H_{18}N_1$	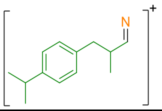

Supplementary Figure 212 | Fragmentation of a molecular key diastereomeric mixture. ESI-MS/MS of a single charged species at 829 *m/z* (⊗). HCD = 35 eV. **Table (bottom):** Fragment assignment. In conclusion the diastereomeric mixture can be read out in the same fashion.

Supplementary Note 1

Solutions for encrypted messages

In this chapter, the solutions for obtaining the encryption keys from the above presented tandem-MS spectra are included.

Solution for example 1

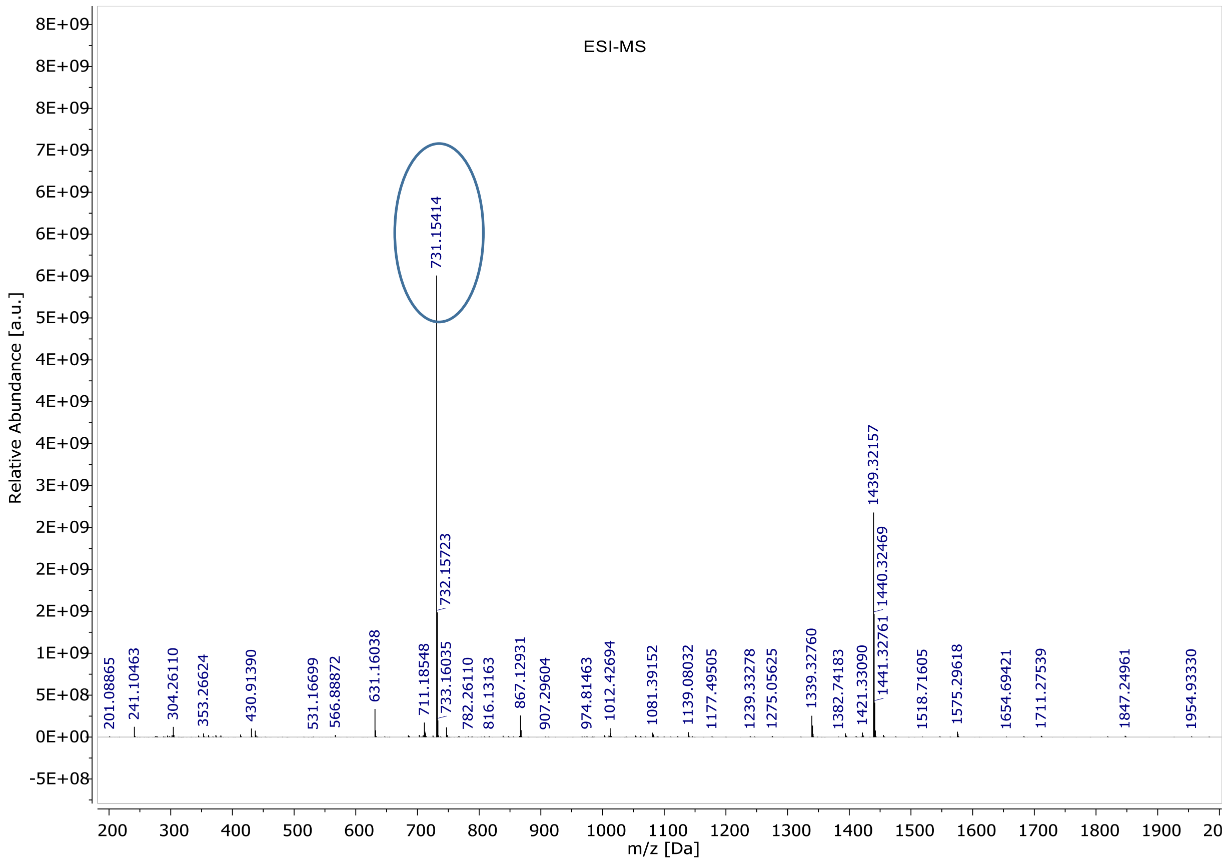

Supplementary Figure 213 | Solution for example 1. ESI-MS.

Enter $[M+Na]^+$ = 731.15414, choose dM = 0.002

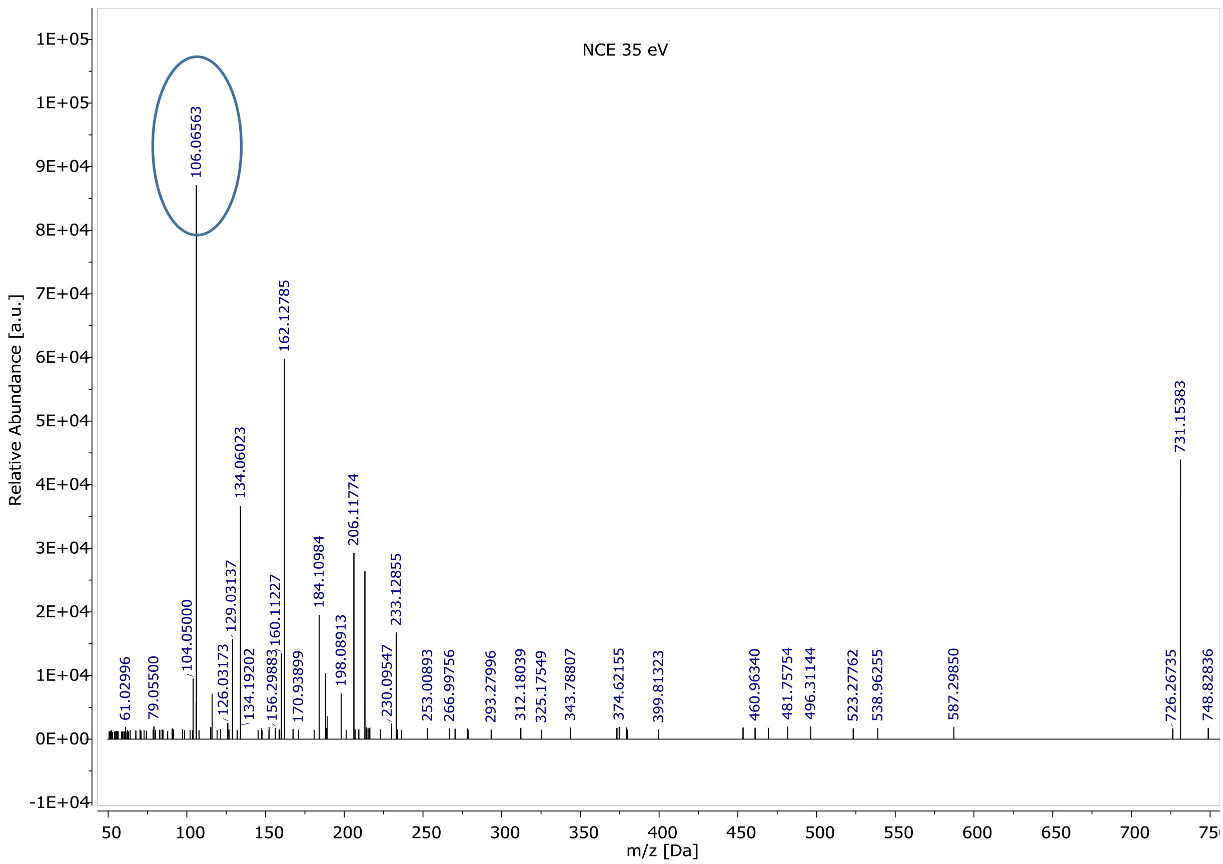

Supplementary Figure 214 | Solution for example 1. ESI-MS/MS. NEC = 35 eV.

Enter 106.06

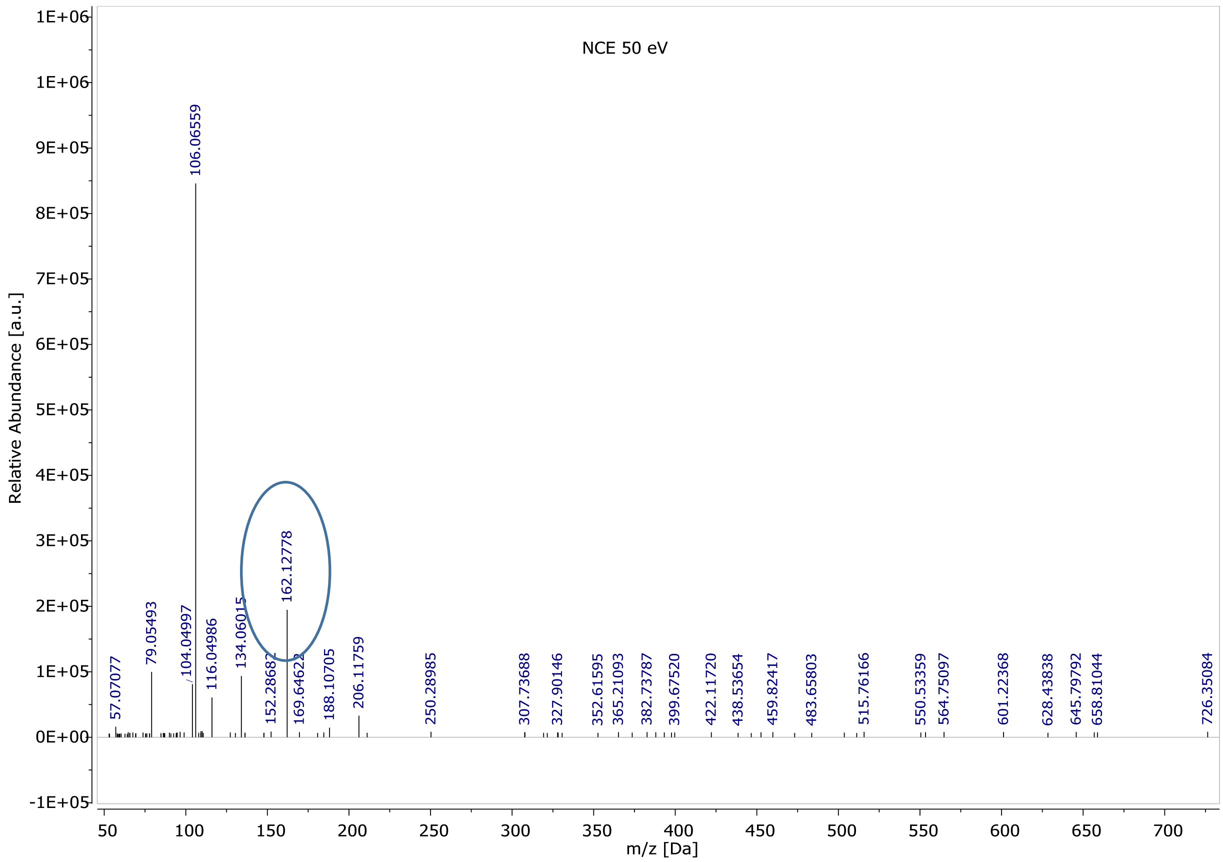

Supplementary Figure 215 | Solution for example 1. ESI-MS/MS. NEC = 50 eV.

Enter 162.12

Solution: A(005)-B(002)-C(004)-D(007)

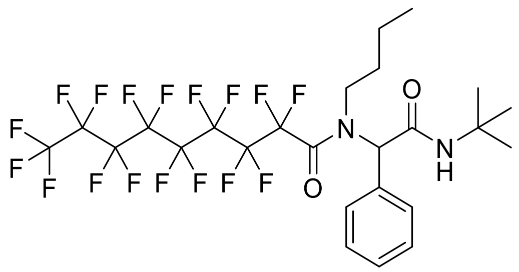

O=C(C(C(C(C(F)(C(F)(C(F)(C(F)(C(F)(F)F)F)F)F)F)(F)F)(F)F)(F)F)N(CCCC)C(C1=CC=CC=C1)C(NC(C)(C)C)=O

Originally encoded text:

Dear reader, congratulations!

You successfully encoded the following message:

The α-addition of immonium ions and anions (OH^{-}, SeH^{-}, $S_2O_3^{2-}$, N_3^{-} NCO^{-} NCS^{-}, R-CO_2^{-}, RO-CO_2^{-}) to isonitriles, accompanied by secondary reactions provides a means for the one-stage synthesis of organic nitrogen compounds starting with two to five different components. Thus, by the condensations of amines (ammonia, primary, and secondary aliphatic and aromatic amines, hydrazines) and aldehydes or ketones with isonitriles and acids, a number of α-aminocarboxylic acid amides, thioamides, selenoamides, 1,5-disubstituted tetrazoles, hydantoin imides, thiohydantoin imides, α-acylamino carboxylic acid amides, oligopeptide derivatives, β-lactams, derivatives of penicillanic acid, urethanes, diacylimides, and various hydrazine derivatives, can be prepared. The reactions are easily carried out and take place under mild conditions. Yields of more than 90% are frequently encountered.

This is the abstract of the original publication from Ivar Ugi describing the herein utilized Ugi reaction.

Ugi, I. (1962), The α-Addition of Immonium Ions and Anions to Isonitriles Accompanied by Secondary Reactions. Angew. Chem. Int. Ed. Engl., 1: 8–21. doi:10.1002/anie.196200081.

Solution for example 2

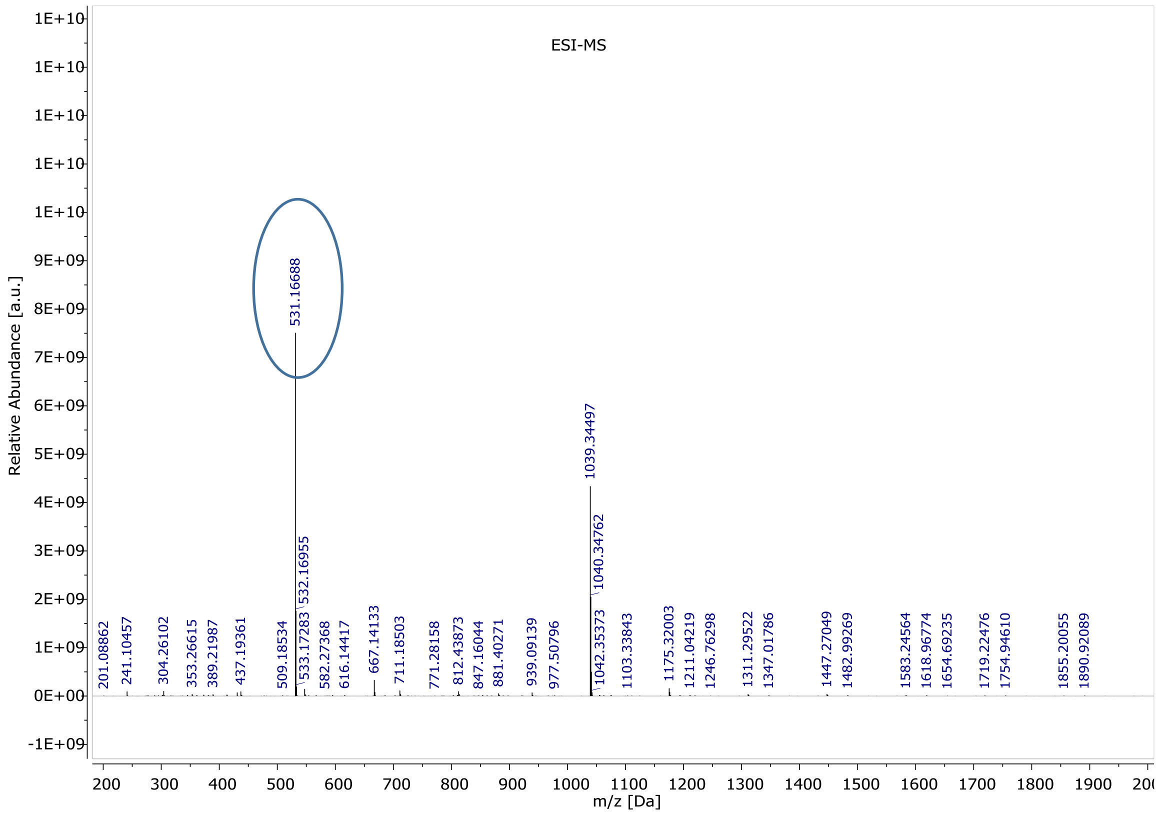

Supplementary Figure 216 | Solution for example 2. ESI-MS.

Enter $[M+Na]^+$ = 531.16688, choose dM = 0.002

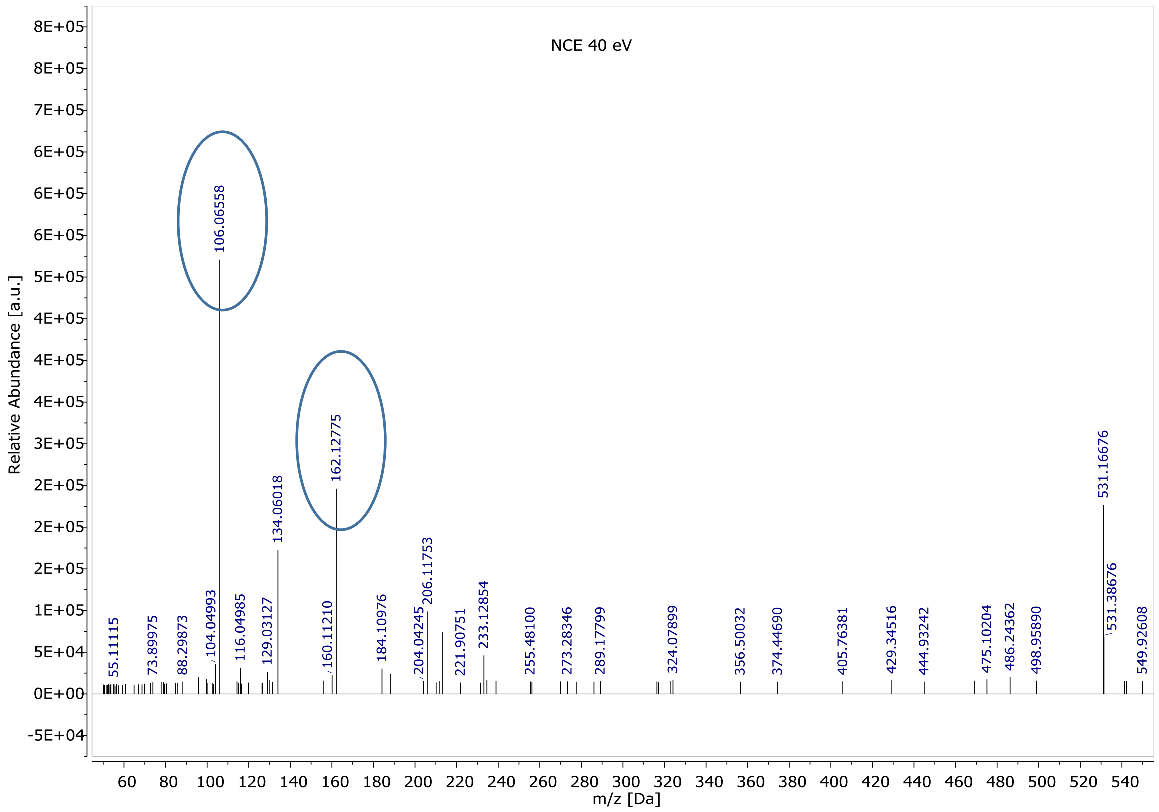

Supplementary Figure 217 | Solution for example 2. ESI-MS/MS. NCE = 40 eV

Enter 106.06 than enter 162.12

Solution: A(001)-B(002)-C(004)-D(007)

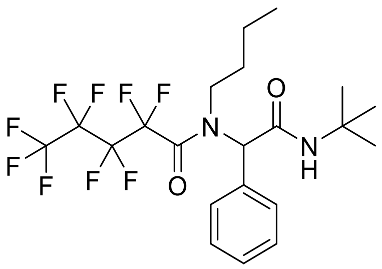

O=C(C(F)(C(F)(C(F)(C(F)(F)F)F)F)F)N(CCCC)C(C1=CC=CC=C1)C(NC(C)(C)C)=O

Originally encoded text:

Dear reader, congratulations!

You successfully encoded the following message:

Wer reitet so spät durch Nacht und Wind?

Es ist der Vater mit seinem Kind;

Er hat den Knaben wohl in dem Arm,

Er fasst ihn sicher, er hält ihn warm.

Mein Sohn, was birgst du so bang dein Gesicht? –

Siehst, Vater, du den Erlkönig nicht?

Den Erlenkönig mit Kron’ und Schweif? –

Mein Sohn, es ist ein Nebelstreif. –

„Du liebes Kind, komm, geh mit mir!

Gar schöne Spiele spiel’ ich mit dir;

Manch’ bunte Blumen sind an dem Strand,

Meine Mutter hat manch gülden Gewand.“ –

Mein Vater, mein Vater, und hörest du nicht,
Was Erlenkönig mir leise verspricht? –
Sei ruhig, bleibe ruhig, mein Kind;
In dürren Blättern säuselt der Wind. –

„Willst, feiner Knabe, du mit mir gehn?
Meine Töchter sollen dich warten schön;
Meine Töchter führen den nächtlichen Reihn
Und wiegen und tanzen und singen dich ein.“ –

Mein Vater, mein Vater, und siehst du nicht dort
Erlkönigs Töchter am düstern Ort? –
Mein Sohn, mein Sohn, ich seh’ es genau:
Es scheinen die alten Weiden so grau. –

„Ich liebe dich, mich reizt deine schöne Gestalt;
Und bist du nicht willig, so brauch’ ich Gewalt.“ –
Mein Vater, mein Vater, jetzt faßt er mich an!
Erlkönig hat mir ein Leids getan! –

Dem Vater grauset’s; er reitet geschwind,
Er hält in Armen das ächzende Kind,
Erreicht den Hof mit Mühe und Not;
In seinen Armen das Kind war tot.

This is the famous German poem „Erlkönig” from Johann Wolfgang von Goethe (1782).

https://en.wikipedia.org/wiki/Erlk%C3%B6nig_(Goethe), accessed june 2017.

Solution for example 3

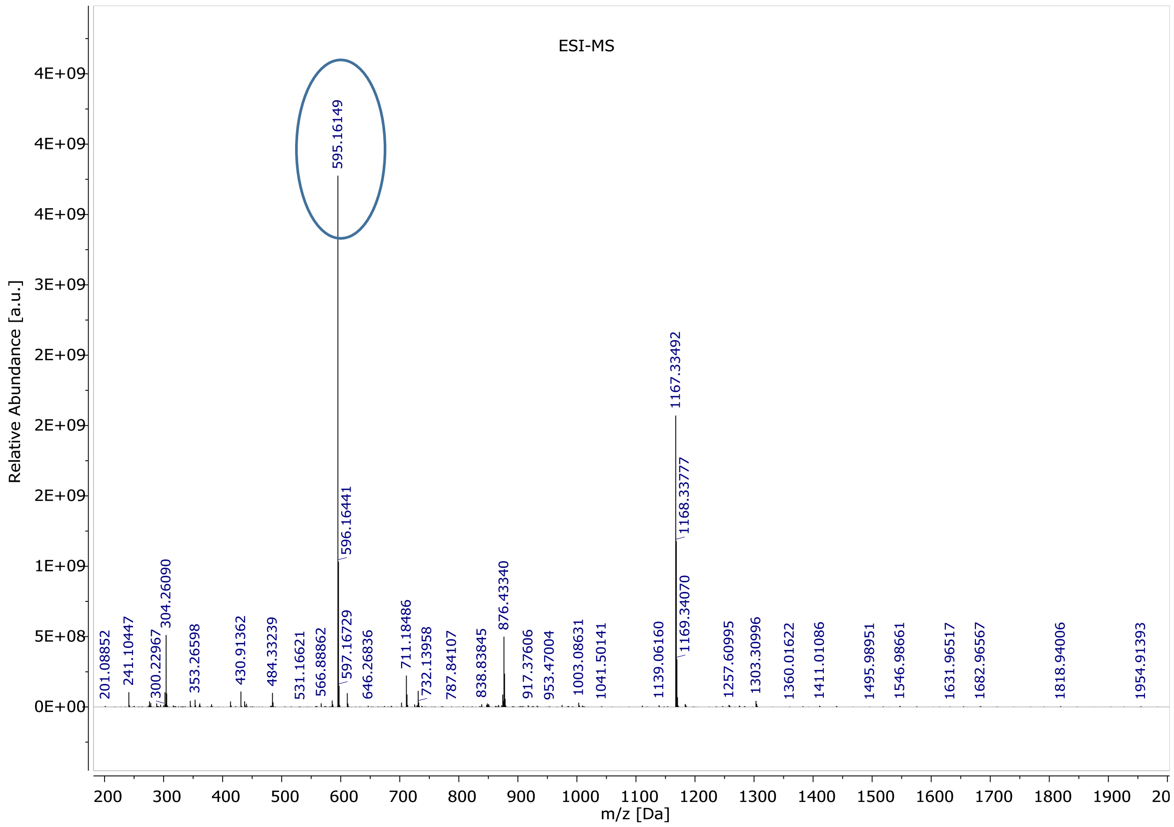

Supplementary Figure 218 | Solution for example 3. ESI-MS.

Enter $[M+Na]^+$ = 595.16149, choose dM = 0.002

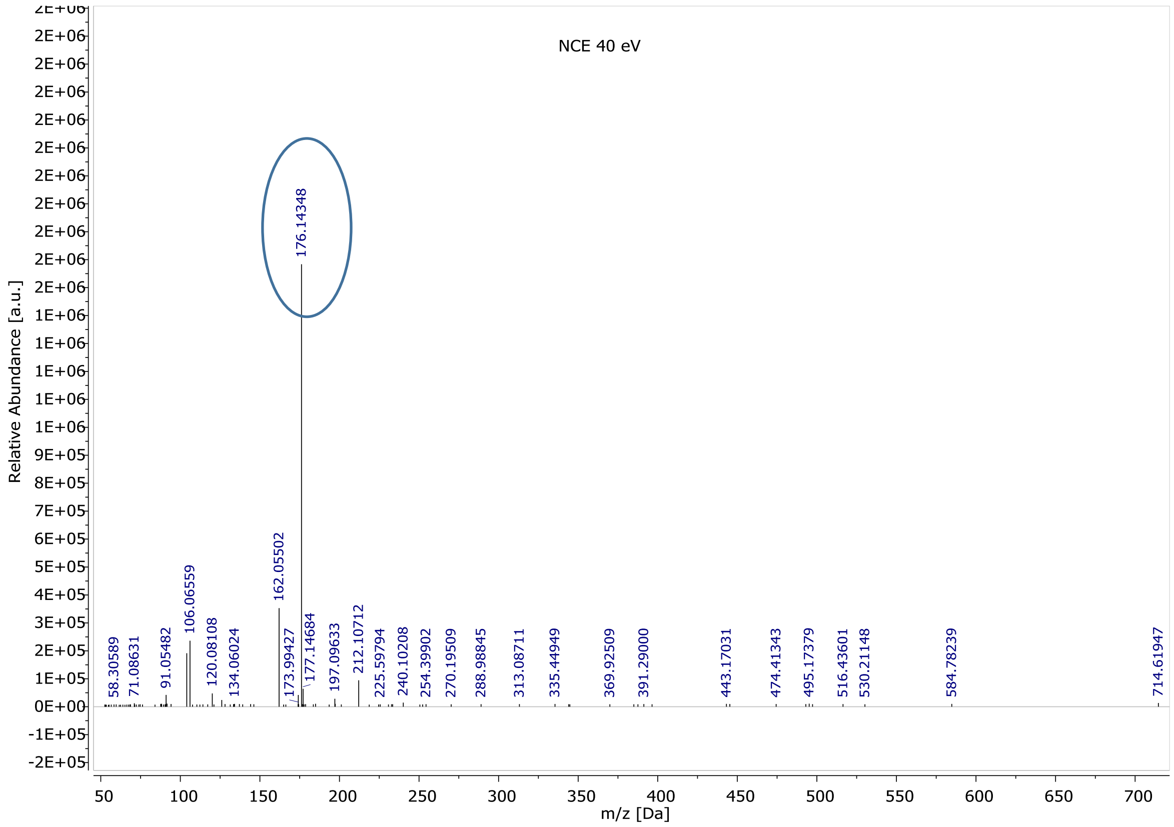

Supplementary Figure 219 | Solution for example 3. ESI-MS/MS. NCE = 40 eV

Enter 176.14

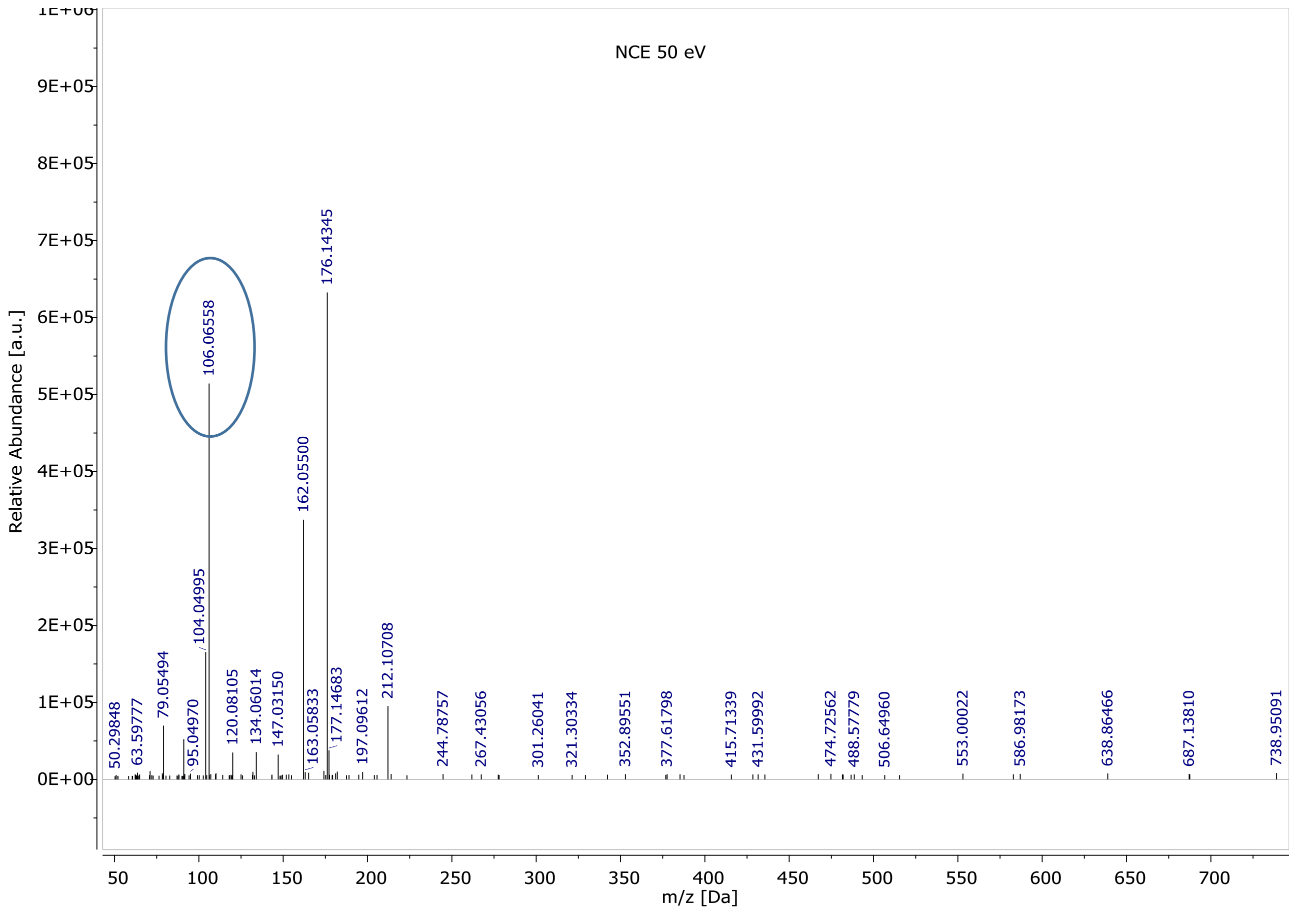

Supplementary Figure 220 | Solution for example 3. ESI-MS/MS. NCE = 50 eV

Enter 106.06

Solution: A(001)-B(012)-C(007)-D(007)

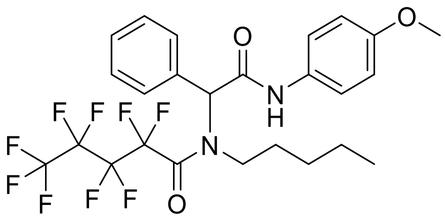

O=C(C(F)(C(F)(C(F)(C(F)(F)F)F)F)F)N(CCCCC)C(C1=CC=CC=C1)C(NC1=CC=C(OC)C=C1)=O

Originally encoded text:

Dear reader, congratulations!

You successfully encoded the following message:

Tell me, Muse, of the man of many ways, who was driven far journeys, after he had sacked Troy's sacred citadel. Many were they whose cities he saw, whose minds he learned of, many the pains he suffered in his spirit on the wide sea, struggling for his own life and the homecoming of his companions. Even so he could not save his companions, hard though he strove to; they were destroyed by their own wild recklessness, fools, who devoured the oxen of Helios, the Sun God, and he took away the day of their homecoming. From some point here, goddess, daughter of Zeus, speak, and begin our story. Then all the others, as many as fled sheer destruction, were at home now, having escaped the sea and the fighting. This one alone, longing for his wife and his homecoming, was detained by the queen nympf Kalypso, bright among goddess, in der hollowed caverns, desiring that he should be her husband.

The Odyssey of Homer, book 1, opening lines. Translated by Richmond Lattimore (1965).

Solution for deciphering the filecontainer

Enter the three molecular keys as follows:

A(005)-B(002)-C(004)-D(007)

A(001)-B(002)-C(004)-D(007)

A(001)-B(012)-C(007)-D(007)

Save as *.zip file.

Supplementary References

1. Still, W. C., Kahn, M. & Mitra, A. Rapid chromatographic technique for preparative separations with moderate resolution. *J. Org. Chem.* **43,** 2923–2925 (1978).

2. Luo, Z. ., Zhang, Q., Oderaotoshi, Y. & Curran, D. P. Fluorous Mixture Synthesis: A Fluorous-Tagging Strategy for the Synthesis and Separation of Mixtures of Organic Compounds. *Science (80-.).* **291,** 1766–1769 (2001).

3. Lu, Y. *et al.* Fluorous diastereomeric mixture synthesis (FDMS) of hydantoin-fused hexahydrochromeno[4,3-b]pyrroles. *Chem. Commun.* **46,** 7578 (2010).

4. Madhusudanan, K. P. Tandem mass spectra of ammonium adducts of monosaccharides: differentiation of diastereomers. *J. Mass Spectrom.* **41,** 1096–1104 (2006).

5. Drabik, E. *et al.* Differentiation of Diastereoisomers of Protected 1,2-Diaminoalkylphosphonic Acids by EI Mass Spectrometry and Density Functional Theory. *J. Am. Soc. Mass Spectrom.* **24,** 388–398 (2013).
