# Supplementary material for: Multicomponent reactions provide key molecules for secret communication
Source: Nat Commun. 2018 Apr 12;9:1439. doi: 10.1038/s41467-018-03784-x (PMC5897361; doi:10.1038/s41467-018-03784-x)
Supplement: Supplementary file 3 — Description of Additional Supplementary Files [file 41467_2018_3784_MOESM3_ESM.pdf]

Description of Additional Supplementary Files

File Name: Supplementary Data 1
Description: The “list of components” used to create the library of compounds.

File Name: Supplementary Data 2
Description: The encrypted Texts/Files.

File Name: Supplementary Software 1
Description: The “Analysis Script”.

File Name: Supplementary Software 2
Description: Executable of the “Molecular Encryption Script – AES”.
